# Screening Libraries to Discover Molecular Design Principles for the Targeted Delivery of mRNA with One-Component Ionizable Amphiphilic Janus Dendrimers Derived from Plant Phenolic Acids †

**DOI:** 10.3390/pharmaceutics15061572

**Published:** 2023-05-23

**Authors:** Juncheng Lu, Elena N. Atochina-Vasserman, Devendra S. Maurya, Muhammad Irhash Shalihin, Dapeng Zhang, Srijay S. Chenna, Jasper Adamson, Matthew Liu, Habib Ur Rehman Shah, Honey Shah, Qi Xiao, Bryn Queeley, Nathan A. Ona, Erin K. Reagan, Houping Ni, Dipankar Sahoo, Mihai Peterca, Drew Weissman, Virgil Percec

**Affiliations:** 1 Roy & Diana Vagelos Laboratories, Department of Chemistry, University of Pennsylvania, Philadelphia, PA 19104-6323, USA; junchlu@sas.upenn.edu (J.L.); maurya@sas.upenn.edu (D.S.M.); shalihin@sas.upenn.edu (M.I.S.); zhdp9114@126.com (D.Z.); srijay.chenna@gmail.com (S.S.C.); jasper.adamson@kbfi.ee (J.A.); liumatt@sas.upenn.edu (M.L.); habib.ur.rehman@iub.edu.pk (H.U.R.S.); honey@sas.upenn.edu (H.S.); qxiao@ethealthcareinc.com (Q.X.); dipankar.sahoo@pennmedicine.upenn.edu (D.S.); mpeterca@gmail.com (M.P.); 2 Department of Medicine, Perelman School of Medicine, University of Pennsylvania, Philadelphia, PA 19104-6323, USA; atochina@pennmedicine.upenn.edu (E.N.A.-V.); queeleyb@pennmedicine.upenn.edu (B.Q.); nathan.ona@pennmedicine.upenn.edu (N.A.O.); ereagan@pennmedicine.upenn.edu (E.K.R.); houping@pennmedicine.upenn.edu (H.N.)

**Keywords:** targeted mRNA delivery, one-component vector, ionizable Janus dendrimer, dendrimersome nanoparticles, molecular design principles, vaccines, nanotherapeutics, plant phenolic acids, simplified preparation and handling, reduced vaccine price

## Abstract

Viral and synthetic vectors to deliver nucleic acids were key to the rapid development of extraordinarily efficient COVID-19 vaccines. The four-component lipid nanoparticles (LNPs), containing phospholipids, PEG-conjugated lipids, cholesterol, and ionizable lipids, co-assembled with mRNA via a microfluidic technology, are the leading nonviral delivery vector used by BioNTech/Pfizer and Moderna to access COVID-19 mRNA vaccines. LNPs exhibit a statistical distribution of their four components when delivering mRNA. Here, we report a methodology that involves screening libraries to discover the molecular design principles required to realize organ-targeted mRNA delivery and mediate activity with a one-component ionizable multifunctional amphiphilic Janus dendrimer (IAJD) derived from plant phenolic acids. IAJDs co-assemble with mRNA into monodisperse dendrimersome nanoparticles (DNPs) with predictable dimensions, via the simple injection of their ethanol solution in a buffer. The precise location of the functional groups in one-component IAJDs demonstrated that the targeted organs, including the liver, spleen, lymph nodes, and lung, are selected based on the hydrophilic region, while activity is associated with the hydrophobic domain of IAJDs. These principles, and a mechanistic hypothesis to explain activity, simplify the synthesis of IAJDs, the assembly of DNPs, handling, and storage of vaccines, and reduce price, despite employing renewable plant starting materials. Using simple molecular design principles will lead to increased accessibility to a large diversity of mRNA-based vaccines and nanotherapeutics.

## 1. Introduction

The efficient delivery of nucleic acids via viral [1,2,3] and synthetic [4,5,6,7,8,9,10,11,12] vectors has remarkably impacted genetic nanomedicine, as illustrated by the great success of COVID-19 vaccines [13,14,15,16,17,18]. Recent reviews [19,20,21,22,23] and three publications from our laboratories [24,25,26] summarized in great detail the advantages and disadvantages of viral and nonviral delivery systems. Therefore, some of these advantages and disadvantages will only be briefly repeated here. This recapitulation has as its main goal a clarification of the scope, properties, and limitations of viral and synthetic vectors for the delivery of DNA and RNA, and of the main functions of the nucleic acids. DNA must be delivered to the nucleus, while RNA is delivered to the cytoplasm. At the same time, due to enzymatic degradation, neither DNA nor RNA is stable, although RNA needs more careful handling since it is single-stranded and is much less stable than DNA. This dissimilar stability of nucleic acids and location of delivery endows the difference between the methodology principles for the delivery of DNA and RNA. 

Viral vectors have high transfection efficacy (95%) and higher specificity for cell targeting, including for unnatural cells. The drawbacks of viral gene delivery include immunogenicity, cytotoxicity, the difficulty of assembly, inflammatory responses to repeated administration, and potential risk for insertional mutagenesis [24,25,26]. 

Delivery via nonviral vectors is safer and exhibits lower toxicity and immunogenicity but exhibits much lower transfection efficiency (1–2%), and the synthetic vectors are less stable than the viral vectors. The unlimited synthetic capabilities of synthetic vectors represent, most probably, their main advantage over viral vectors. Taking all these features into account, we can understand why covalent and supramolecular dendrimers, as well as other permanently charged synthetic vectors complexed on their cationic periphery groups with nucleic acids, have pioneered the field of nonviral vectors for the cell transfection of DNA. Due to the relatively higher stability of DNA, no protection–deprotection methodology is required during the delivery of DNA. However, a protection–deprotection methodology is required for the delivery of RNA. More details on these principles and methodologies will be briefly summarized in a later part of this report. 

**Figure 1:**
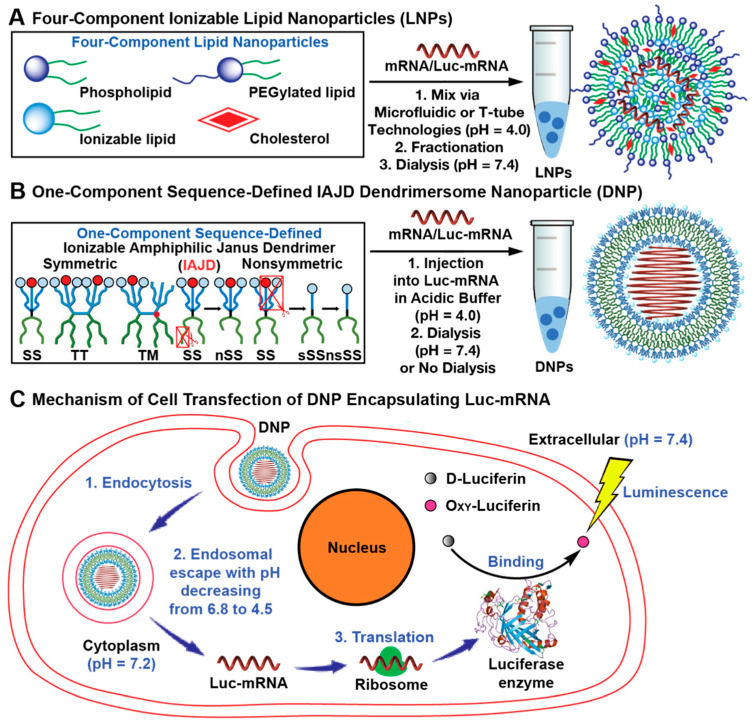
Schematic representation of four-component LNPs, one-component IAJD-based DNPs, and the cell transfection mechanism of LNPs and DNPs encapsulating Luc-mRNA: (**A**) four-component LNPs; (**B**) one-component IAJDs and their DNPs; (**C**) cell transfection mechanism of LNPs and DNPs encapsulating Luc-mRNA.

Four-component lipid nanoparticles (LNPs) [27,28,29] consisting of ionizable lipids [27,28], phospholipids [29], cholesterol, and a PEG-conjugated lipid [26,27,28], represent the state-of-the-art technology employed by BioNTech/Pfizer [16] and Moderna [17] for the production of their mRNA vaccines (Figure 1A). The protection–deprotection is accomplished in this case through a reversible encapsulation of the mRNA mediated by an ionizable lipid rather than by the permanently charged cationic components employed in the delivery of DNA. The stability of the nanoparticle is aided through co-assembly with cholesterol and PEG-conjugated lipids. The statistical distribution of the four components in the LNPs is responsible for some of their limitations and difficulties of molecular design since the exact location of their four components within LNPs is not known. For example, the segregation of the neutral ionizable lipid as droplets in the core of LNPs [28,30] and the “PEG dilemma” [31,32,33,34] are two of the main outcomes induced by the lack of their precise location. An additional deficiency is optimal stability at temperatures of about −70 °C. The detailed structure of the LNP is also not yet known in great detail, but it will be discussed, together with alternative methods of delivery and their evolution, in a later subsection. 

Recently, we took advantage of the unlimited synthetic capabilities of nonviral vectors and elaborated a simple one-component sequence-defined ionizable amphiphilic Janus dendrimer (IAJD) [24,25,26] concept that relies not only on precise composition but also, most importantly, on the exact location and placement of its functional groups (Figure 1B) [24,25,26]. In addition, one-component IAJDs do not require the microfluidic or T-tube technology employed by four-component LNPs to co-assemble with mRNA. One-component IAJDs are co-assembled with mRNA into vesicles named dendrimersome nanoparticles (DNPs). They exhibit 97% nucleic acid encapsulation efficiency [24] and are assembled via the simple injection of their ethanol solution into an acidic buffer containing mRNA rather than via the microfluidic or T-tube technology required using LNPs. This simple assembly methodology provides rapid access to screening libraries of IAJDs with different compositions and sequences, both in their hydrophilic and hydrophobic parts. 

Here, we will elaborate on a methodology for screening libraries to discover, and later even predict, the molecular design principles required to accomplish organ-targeted mRNA delivery and engineer activity with a one-component ionizable multifunctional amphiphilic Janus dendrimer (IAJD) derived from plant phenolic acids. Quantitative experiments on this topic are outside the scope of this report but will become available soon. 

Since this is an invited publication by the Guest Editors of a Special Issue dedicated to the 85th anniversary of Donald A. Tomalia, we decided to organize a more unusual manuscript that represents a combination of an original research paper, as the title indicates, in the first part, combined with a perspective on the discovery, evolution, and the immense role of Don on the discovery, evolution, and the current status of covalent and supramolecular dendrimers, in the second part.

## 2. Materials and Methods

### 2.1. Materials

3,5-Dihydroxybenzoic acid (Acros, 97%), 1-bromononane (Lancaster, 99%), 1-bromododecane (Alfa Aesar, 99%),1-bromotetradecane (Acros, 98%), 1-bromohexadecane (TCI, 96%), 1-heptadecanol (TCI, 97%) (*rac*)-3-(bromomethyl)heptane, 1-bromooctadecane (Acros, 96%), 2-ethylhexyl bromide, (Aldrich, 95%), 1-bromooctane (Aldrich, 99%), 1-bromodecane (Acros, 98%), 1-bromoundecane (Aldrich, 99%), 1-bromopentadecane (Aldrich, 98%), benzyl chloride (Alfa Aesar, 99%), 4-toluenesulfonyl chloride (Alfa Aesar, 98%), palladium on activated carbon catalyst (Spectrum, 10 wt% loading), lithium aluminum hydride (LiAlH_4_, TCI, 95%), 4-bromobutyric acid (Acros, 98%), thionyl chloride (Alfa Aesar, 99+%), potassium phthalimide (Chem Impex, 99.8%), 1-methylpiperazine (Alfa Aesar, 98%), 1-(2-hydroxyethyl)piperazine (Acros, 99%), and triethylamine (TCI, 99%) were used as received. Heptadecyl 4-methylbenzenesulfonate (C_17_H_35_OTs) was synthesized according to a literature procedure reported by our laboratory [26]. All other reagents and solvents were obtained from commercial sources and were used as received. CH_2_Cl_2_ (DCM) was dried over CaH_2_ and distilled before use. 4-(Dimethylamino)pyridinium 4-toluenesulfonate (DPTS) was prepared according to a literature method [35]. Acetate buffer (10 mM) was prepared by dissolving sodium acetate (2.3 mM) and acetic acid (7.7 mM) in ultra-pure water. The final pH of the buffer was adjusted with 0.1 M HCl or 0.1 M NaOH solution. Nucleoside-modified messenger RNA encoding firefly luciferase (Luc-mRNA) was produced as reported [36]. Human embryonic kidney (HEK) 293T cells (American Type Culture Collection, Manassas, VA, USA) were cultured in Dulbecco’s modified Eagle’s medium (DMEM) supplemented with 10% inactivated fetal bovine serum (FBS) (Gemini Bio-Products, West Sacramento, CA, USA), 2 mM L–glutamine and 100 U/mL penicillin/streptomycin (Life Technologies, Carlsbad, CA, USA). DPBS (Corning, Corning, NY, USA), OptiMEM (Gibco, Carlsbad, CA, USA), UltraPure DNase/RNase-free distilled water (Invitrogen, Carlsbad, CA, USA ), Trypsin-EDTA (0.25%, Gibco, Carlsbad CA, USA), Trypan Blue (Sigma-Aldrich, St. Louis, MO, USA), Cell Culture Lysis 5X Reagent (Promega, Madison, WI, USA), Luciferase Assay System (Promega, Madison, WI, USA), and D-luciferin sodium salt (Regis Technologies, Morton Grove, IL, USA) were used as received.

### 2.2. Characterization and Methods

The purity and structure of intermediate compounds and final products were determined using a combination of techniques, including thin-layer chromatography (TLC), ^1^H and ^13^C NMR, high-pressure liquid chromatography (HPLC), and matrix-assisted laser desorption ionization–time-of-flight (MALDI-TOF) mass spectrometry.

#### 2.2.1. ^1^H and ^13^C NMR

^1^H and ^13^C NMR spectra were recorded at 400 MHz and 101 MHz, respectively, on a Bruker (Billerica, MA, USA) NEO (400 MHz) NMR spectrometer equipped with an autosampler, or 500 MHz and 126 MHz, respectively, on a Bruker DRX (500 MHz) NMR spectrometer. All NMR values were measured at 23 °C in CDCl_3_. Chemical shifts (*δ*) are reported in ppm. The resonance multiplicities in the ^1^H NMR spectra are indicated as “s” (singlet), “d” (doublet), “t” (triplet), “m” (multiplet), and “br” (broad resonance). A residual protic solvent of CDCl_3_ (^1^H, *δ* 7.26 ppm; ^13^C, *δ* 77.16 ppm) and tetramethylsilane (TMS, *δ* 0 ppm) were used as the internal reference in the ^1^H and ^13^C NMR spectra. NMR spectra were analyzed using MNova 14 or TopSpin 4.07 (Bruker). More details are available in previous publications from our laboratory [24,25,26].

#### 2.2.2. Thin-Layer Chromatography (TLC) 

TLC was used to monitor the evolution of the extent of the reaction by using silica gel 60 F_254_ precoated plates (E. Merck, Darmstadt, Germany). The individual compounds with aromatic groups were visualized using UV light (*λ* = 254 nm). For compounds without aromatic groups, the TLC plate was stained with iodine vapor to help visualization. Purification via flash column chromatography (SiO_2_) was performed using silica gel from Silicycle (60 Å, 40–63 μm), with the eluent partially mentioned in the experimental part for each compound. More details are available in previous publications from our laboratory [24,25,26].

#### 2.2.3. High-Pressure Liquid Chromatography (HPLC)

The determination of the purity of individual compounds via HPLC was performed by using a Shimadzu LC-20AD high-performance liquid chromatograph pump, a PE Nelson Analytical 900 Series integration data station, a Shimadzu SPD-10A VP (UV–vis, *λ* = 254 nm), and three AM gel columns (a guard column, two 500 Å, 10 μm columns). THF with 5% of NEt3 was used as the solvent, and characterization was carried out at 23 °C. The detection of the compounds was determined using UV absorbance (*λ* = 254 nm) or an RI (refractive index) detector. More details are available in previous publications from our laboratory [24,25,26].

#### 2.2.4. Matrix-Assisted Laser Desorption Ionization–Time-of-Flight (MALDI-TOF) Mass Spectrometry

The molar mass of all molecules was determined via MALDI-TOF mass spectrometry employing a Perseptive Biosystem-Voyager-DE (Framingham, MA, USA) mass spectrometer equipped with a nitrogen laser (337 nm) and operating in linear mode. Angiotensin II and Bombesin were used as standards for calibration. For the preparation of the sample solution, the corresponding compound was first dissolved in THF (5–10 mg/mL). Subsequently, the matrix (2,5-dihydroxybenzoic acid) was dissolved in THF 10 mg/mL, and the two solutions were mixed with a 1/5 (*v*/*v*, compound solution/matrix solution) ratio. Then, one drop of solution was placed on the MALDI plate and dried at 23 °C. Afterward, the plate was inserted into the vacuum chamber of the instrument for analysis. The laser intensity and voltages applied for the analysis were adjusted based on the molar mass and nature of each compound. More details are available in previous publications from our laboratory [24,25,26].

#### 2.2.5. Dynamic Light Scattering (DLS)

DLS for the dimensions (sizes and polydispersities) of DNPs was performed on a Malvern Instruments particle sizer (Zetasizer Nano S, Malvern Instruments, Malvern, UK) equipped with 4 mW He–Ne laser 633 nm and avalanche photodiode positioned at 175° to the beam and temperature-controlled cuvette holder. Instrument parameters were set up automatically along with measurement times. The sample solution (c.a. 0.4 mL) was placed in a semi-micro cuvette (1.6 mL, polystyrene, 10 × 10 × 45 mm, Greiner Bio-One, Monroe, NC, USA), and the experiments were performed at 23 °C. More details are available in previous publications from our laboratory [24,25,26].

#### 2.2.6. pKa Measurements of Individual IAJDs

IAJDs were dissolved in ethanol (sat. with NaCl) at a concentration of 1.5 mg/mL and in a volume of 3 mL. Then, a 0.1 M HCl solution was added to the ethanol solution, with an increment of 7.5 μL. The resulting pH, after each addition of HCl solution, was measured using a Thermo Scientific Orion Star A121(Waltham, MA, USA) meter with a Thermo Scientific Orion 8220BNWP pH probe. pK_a_ was determined using the half equivalence point titration. More details are available in previous publications from our laboratory [24,25,26].

#### 2.2.7. Production of Nucleoside-Modified Luc-mRNA

mRNA was produced as previously described [36] using T7 RNA polymerase on linearized DNA encoding codon-optimized firefly luciferase and a 101nt poly(A) tail. 1-methylpseudouridine-5′-triphosphate was used instead of UTP. A trinucleotide cap1 analog was added co-transcriptionally. Purification was performed as previously described [37]. mRNA was analyzed for RNase, dsRNA, endotoxin, and other forms of contamination and stored frozen at −20 °C.

#### 2.2.8. Formulation of DNPs Co-Assembled from IAJDs and Luc-mRNA

Nucleoside-modified mRNA encoding firefly luciferase (Luc-mRNA) was dissolved in UltraPure DNase/RNase-free distilled water with an initial concentration of 4.0 mg/mL. IAJDs were dissolved in ethanol at an initial concentration of 80 mg/mL. Luc-mRNA solution (12.5 μL) was placed into a clean RNA-free Eppendorf (1.5 mL), and 463 μL of acetate buffer (10 mM, pH 4.0) was added. The IAJD stock solution in ethanol was taken (25 μL) and rapidly injected into the above Luc-mRNA solution in acetate buffer, followed by vortex for 5 s. More details are available in previous publications from our laboratory [24,25,26].

#### 2.2.9. Luminescence Characterization for In Vivo Transfection Experiments 

Bioluminescence imaging was performed with an IVIS Spectrum imaging system (PerkinElmer, Waltham, MA, USA). Mice were anesthetized with 3% of isoflurane (Piramal Healthcare Limited) and intraperitoneally (i.p.) administered with D-luciferin (Regis Technologies) at a dose of 150 mg/kg of body weight. Ten minutes after the administration of D-luciferin, mice were placed on the imaging platform while being maintained on isoflurane via a nose cone and imaged using a certain exposure time (60, 30, or 15 s). Bioluminescence values were quantified by measuring photon flux (photons/second, p/s) in the region of interest (ROI) on mice, where the bioluminescence signal emanated was analyzed using the Living Image Software (PerkinElmer). To quantify luminescent flux, an oval ROI was placed over each organ of interest and analyzed. More details are available in previous publications from our laboratory [24,25,26].

#### 2.2.10. In Vivo mRNA Delivery in Mice with DNPs 

All the mice used were in accordance with the guidelines and approval from the Pennsylvania University Institution of Animal Care and Use Committee. Female or male BALB/c mice (6–8 weeks old, from Charles River Laboratories, Wilmington, MA, USA) were anesthetized with isoflurane (Piramal Healthcare Limited) and injected via retro-orbital sinus with 100 μL of a DNP solution containing 10 μg of Luc-mRNA. Then, 4 to 6 h after injection, mice were i.p. injected with D-luciferin (150 mg/kg of body weight, Regis Technologies) and imaged on a PerkinElmer IVIS Spectrum CT system (PerkinElmer, Waltham, MA). The tissue luminescence signal was measured on the IVIS imaging system using a certain exposure time (60, 30, or 15 s) and medium binning (binning = 8) to ensure that the signal obtained was within the operative detection range. For the IVIS imaging of the organs, mice were sacrificed, and the heart, lungs, liver, and spleen were immediately collected; then, bioluminescence imaging was performed as described above. Image analysis was conducted with the Living Image software (PerkinElmer). Bioluminescence values were quantified by measuring photon flux (photons/second) in the region of interest (ROI) using the Living Image software. More details are available in previous publications from our laboratory [24,25,26].

#### 2.2.11. Molecular Modelling

Molecular models of the IAJD bilayers were drawn using the DS ViewerPro (version 5.0) software. The Material Studio Modeling (version 3.1) software from Accelrys was used to perform energy minimization analyses of the built models on supramolecular structures. BIOVIA Discovery Studio Visualizer (version 2019) was used for display style and coloring. Color codes were used similarly to those in the ChemDraw (Waltham, MA, USA) structure (hydrophilic part in blue; hydrophobic part in light and dark green; oxygen and OH in pink; carbons in the aromatic ring in gray; and H groups on aromatic in white).

### 2.3. Synthesis of IAJDs

#### 2.3.1. Synthesis of the Monoprotected Benzyl Ether of Methyl 3,5-Dihydroxybenzoate, 2

**Scheme 1:**
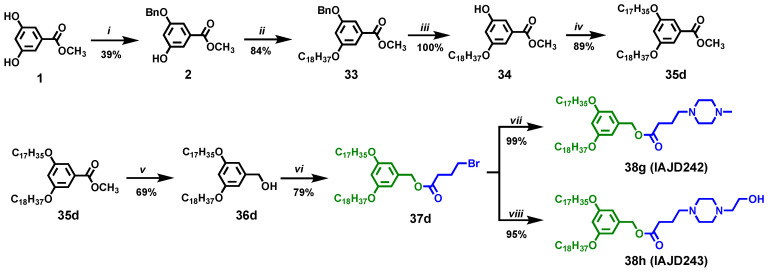
Synthesis of 3,5-nonsymmetric nsSS IAJDs: (i) BnCl, K_2_CO_3_, KI, DMF, 80 °C, 5 h; (ii) C_18_H_37_Br, K_2_CO_3_, DMF, 120 °C, 2 h; (iii) H_2_, Pd/C, DCM, MeOH, 12 h; (iv) C_17_H_35_OTs, K_2_CO_3_, DMF, 120 °C, 2 h; (v) LiAlH_4_, THF, 0–23 °C, 1 h; (vi) DCC, DPTS, DCM 12 h; (vii) methylpiperazine, K_2_CO_3_, MeCN, 95 °C, 3 h; (viii) 1-(2-hydroxyethyl)piperazine, K_2_CO_3_, MeCN, 95 °C, 3 h.

Methyl 3,5-dihydroxybenzoate, **1**, (10.00 g, 59.5 mmol, 1.0 equiv) (Scheme 1), benzyl chloride (8.30 g, 65.4 mmol, 1.1 equiv), KI (1.23 g, 7.43 mmol, 0.125 equiv), K_2_CO_3_ (9.06 g, 65.4 mmol, 1.1 equiv), and DMF (50 mL) were added into a 100 mL round-bottom flask. The reaction mixture was stirred at 600 rpm, at 80 °C under N_2_ while monitored for conversion using TLC. After 5 h, methyl 3,5-dihydroxybenzoate was completely consumed, and the reaction mixture was poured into ice/water (150 mL). The aqueous layer was extracted with ethyl acetate 4 times. The organic phase was dried over MgSO_4_, and the solvent was removed in a rotary evaporator. The solid product was purified via column chromatography on silica gel with hexane/ethyl acetate = 5/1 as mobile phase to obtain 6.00 g of white crystals (isolated yield, 39%). Mp = 96 °C. ^1^H NMR (400 MHz, CDCl_3_) *δ* 7.46–7.34 (m, 5 H, Ph*H*), 7.28 (s, 1 H, Ph*H*), 7.17 (s, 1 H, Ph*H*), 6.71 (s, 1 H, Ph*H*), 5.10 (s, 2 H, PhC*H*_2_O-), 3.93 (s, 3 H, PhCOOC*H*_3_). ^13^C NMR (101 MHz, CDCl_3_) *δ* 167.6, 160.0, 157.2, 136.5, 131.8, 128.7, 128.2, 127.7, 109.8, 108.0, 107.7, 70.3, 52.6. Purity by HPLC: 99+%. MALDI-TOF MS *m*/*z* of [M + H]^+^ calculated for C_15_H_15_O_4_: 259.1; found: 259.4. [M + Na]^+^ calculated for C_15_H_14_NaO_4_: 281.1; found: 281.3. [M + K]^+^ calculated for C_15_H_14_KO_4_: 297.1; found: 297.2 (Figure S1). 

#### 2.3.2. Synthesis of Methyl 3-(benzyloxy)-5-(octadecyloxy)benzoate, **33**

Methyl 3-(benzyloxy)-5-hydroxybenzoate, 2, (2.00 g, 7.74 mmol, 1 equiv), 1-bromooctadecane (2.84 g, 8.52 mmol, 1.1 equiv), K_2_CO_3_ (2.14 g, 15.48 mmol, 2 equiv), and 30 mL DMF were heated at 120 °C and stirred under a N_2_ atmosphere for 2 h. The reaction mixture was cooled to 23 °C poured into 150 mL ice water. The resulting white precipitate was filtered and collected. Then, the precipitate was recrystallized from minimum acetone to afford the title compound as a white solid (3.28 g, 84%). Mp = 65 °C. ^1^H NMR (400 MHz, CDCl_3_) *δ* 7.48–7.29 (m, 5 H, PhH), 7.27 (br, 1 H, PhH), 7.20 (br, 1 H, PhH), 6.73 (t, 1 H, PhH), 5.08 (s, 2 H, PhCH_2_O-), 3.97 (t, 2 H, PhOCH_2_-), 3.90 (s, 3 H, PhCOOCH_3_), 1.78 (m, 2 H, PhOCH_2_CH_2_CH_2_(CH_2_)_14_CH_3_), 1.44 (m, 2 H, PhOCH_2_CH_2_CH_2_(CH_2_)_14_CH_3_), 1.28 (m, 28 H, PhOCH_2_CH_2_CH_2_(CH_2_)_14_CH_3_), 0.89 (t, 3 H, PhO(CH_2_)_17_CH_3_). ^13^C NMR (101 MHz, CDCl_3_) *δ* 167.0, 160.3, 159.9, 136.7, 132.1, 128.7, 128.2, 127.7, 108.3, 108.0, 107.1, 77.4, 70.4, 68.5, 52.3, 32.1, 29.8, 29.7, 29.7, 29.5, 29.3, 26.4, 26.1, 22.9, 22.8, 14.2. Purity by HPLC: 99+%. MALDI-TOF MS *m*/*z* of [M + H]^+^ calculated for C_33_H_51_O_4_: 511.4; found: 512.5. [M + Na]^+^ calculated for C_33_H_50_NaO_4_: 533.4; found: 534.5. [M + K]^+^ calculated for C_33_H_50_KO_4_: 549.3; found: 550.4 (Figure S2).

#### 2.3.3. Synthesis of the Methyl 3-hydroxy-5-(octadecyloxy)benzoate, **34**

Methyl 3-(benzyloxy)-5-(octadecyloxy)benzoate, **33**, (3.26 g, 6.42 mmol), DCM (20 mL), and methanol (10 mL) were added in a 50 mL round-bottom flask. Then, Pd/C (0.16 g, 5 wt%) was added to the solution, and the flask was evacuated and filled with hydrogen three times. Afterward, the mixture was stirred at 23 °C under a hydrogen atmosphere for 12 h. The reaction mixture was filtered through Celite, and the filter cake was washed with DCM. The evaporation of the solvent yielded the title compound as a white solid (2.70 g, 100%). Mp = 99 °C. ^1^H NMR (400 MHz, CDCl_3_) *δ* 7.13 (d, 2 H, Ph*H*), 6.64 (t, 1 H, Ph*H*), 3.95 (t, 2 H, PhOC*H*_2_-), 3.89 (s, 3 H, PhCOOC*H*_3_), 1.75 (m, 2 H, PhOCH_2_C*H*_2_CH_2_(CH_2_)_14_CH_3_), 1.44 (m, 2 H, PhOCH_2_CH_2_C*H*_2_(CH_2_)_14_CH_3_), 1.26 (m, 28 H, PhOCH_2_CH_2_CH_2_(C*H*_2_)_14_CH_3_), 0.88 (t, 3 H, PhO(CH_2_)_17_C*H*_3_). ^13^C NMR (101 MHz, CDCl_3_) *δ* 167.0, 160.4, 157.0, 131.9, 109.0, 107.7, 107.1, 68.4, 52.2, 31.9, 29.7, 29.7, 29.6, 29.6, 29.4, 29.4, 29.2, 26.0, 22.7, 14.2. Purity by HPLC: 99+%. MALDI-TOF MS *m*/*z* of [M + H]^+^ calculated for C_26_H_45_O_4_: 421.3; found: 421.5. [M + Na]^+^ calculated for C_26_H_44_NaO_4_: 443.3; found: 443.4. [M + K]^+^ calculated for C_26_H_44_KO_4_: 459.7; found: 459.2 (Figure S3).

#### 2.3.4. Synthesis of Methyl 3-(heptadecyloxy)-5-(octadecyloxy)benzoate (**35d**)

Methyl 3-hydroxy-5-(octadecyloxy)benzoate, **34**, (0.50 g, 1.19 mmol, 1 equiv), C_17_H_35_OTs (0.54 g, 1.31 mmol, 1.1 equiv), and K_2_CO_3_ (0.33 g, 2.38 mmol, 2 equiv) were heated at 120 °C and stirred under a N_2_ atmosphere for 2 h. The reaction mixture was cooled to 23 °C and poured into ice/water (150 mL). The resulting white precipitate was filtered and collected. Then, the precipitate was recrystallized from minimum acetone to afford the title compound as a white solid (0.70 g, 89%). Mp = 72 °C. ^1^H NMR (400 MHz, CDCl_3_) *δ* 7.15 (br, 2 H, Ph*H*), 6.63 (t, 1 H, Ph*H*), 3.96 (t, 4 H, PhOC*H*_2_CH_2_CH_2_-), 3.89 (s, 3 H, PhCOOC*H*_3_), 1.77 (m, 4 H, PhOCH_2_C*H*_2_CH_2_-), 1.43 (m, 4 H, PhOCH_2_CH_2_C*H*_2_-), 1.26 (m, 54 H, PhOCH_2_CH_2_CH_2_(C*H*_2_)_13_CH_3_ and PhOCH_2_CH_2_CH_2_(C*H*_2_)_14_CH_3_), 0.88 (t, 6 H, PhO(CH_2_)_16_C*H*_3_ and PhO(CH_2_)_17_C*H*_3_). ^13^C NMR (101 MHz, CDCl_3_) *δ* 167.1, 160.3, 132.0, 107.8, 106.7, 68.5, 52.3, 34.1, 33.0, 32.1, 29.9, 29.8, 29.8, 29.8, 29.7, 29.7, 29.6, 29.5, 29.3, 28.9, 28.3, 26.2, 22.8, 14.3. Mp = 72 °C. Purity by HPLC: 99+%. MALDI-TOF MS *m*/*z* of [M + H]^+^ calculated for C_43_H_79_O_4_: 659.6; found: 660.3. [M + Na]^+^ calculated for C_43_H_78_NaO_4_: 681.6; found: 682.4. [M + K]^+^ calculated for C_43_H_78_KO_4_: 697.6; found: 699.5 (Figure S4). 

#### 2.3.5. Synthesis of (3-(Heptadecyloxy)-5-(octadecyloxy)phenyl)methanol (**36d**)

Methyl 3-(heptadecyloxy)-5-(octadecyloxy)benzoate, **35d**, (0.70 g, 1.06 mmol, 1 equiv) dissolved in 5 mL dry THF, which was added dropwise to a slurry of LiAlH_4_ (40 mg, 1.06 mmol, 1 equiv) in dry THF (5 mL) at 0 °C under a N_2_ atmosphere. Afterward, the resulting mixture was stirred at 23 °C for 1 h. Finally, the reaction was quenched via the successive addition of water (0.2 mL), a 15% NaOH aqueous solution (0.2 mL), and water (1.0 mL). The white precipitate was filtered. The solid filtrate was dissolved in DCM, dried over anhydrous MgSO_4_, and filtered. The solvent was removed under reduced pressure to afford the title compound as a white solid (0.46 g, 69%). Mp = 62 °C. ^1^H NMR (400 MHz, CDCl_3_) *δ* 6.49 (br, 2 H, Ph*H*), 6.37 (t, 1 H, Ph*H*), 4.61 (s, 2 H, PhC*H*_2_OH), 3.93 (t, 4 H, PhOC*H*_2_CH_2_CH_2_-), 1.76 (m, 4 H, PhOCH_2_C*H*_2_CH_2_-), 1.44 (m, 4 H, PhOCH_2_CH_2_C*H*_2_-), 1.29 (m, 54 H, PhOCH_2_CH_2_CH_2_(C*H*_2_)_13_CH_3_ and PhOCH_2_CH_2_CH_2_(C*H*_2_)_14_CH_3_), 0.88 (t, 6 H, PhO(CH_2_)_16_C*H*_3_ and PhO(CH_2_)_17_C*H*_3_). ^13^C NMR (101 MHz, CDCl_3_) *δ* 160.7, 160.6, 143.4, 125.7, 105.2, 100.7, 68.2, 65.6, 32.1, 30.5, 29.9, 29.8, 29.8, 29.7, 29.6, 29.5, 29.5, 29.4, 26.2, 25.9, 22.8, 14.3. Mp = 62 °C. Purity by HPLC: 99+%. MALDI-TOF MS *m*/*z* [M + H]^+^ calculated for C_42_H_77_O_4_: 630.6; found: 631.3. [M + Na]^+^ calculated for C_42_H_78_NaO_4_: 653.6; found: 653.0. [M + K]^+^ calculated for C_42_H_78_KO_4_: 670.6; found: 670.6 (Figure S5).

#### 2.3.6. Synthesis of 3-(Heptadecyloxy)-5-(octadecyloxy)benzyl 4-bromobutanoate, **37d**

A solution of (3-(heptadecyloxy)-5-(octadecyloxy)phenyl)methanol, **36d**, (0.46 g, 0.73 mmol, 1 equiv), 4-bromobutyric acid (0.13 g, 0.80 mmol, 1.1 equiv), and DPTS (0.24 g, 0.80 mmol, 1.1 equiv) was dissolved in 8 mL DCM. DCC (0.30 g, 1.46 mmol, 2 equiv) was added to the above mixture. The reaction mixture was stirred at 23 °C for 12 h. Afterward, the resulting precipitate (urea) was filtered out from the reaction mixture. The filtrate was concentrated to around 3 mL and purified via column chromatography (SiO_2_) with hexane/DCM = 1/1 as the mobile phase to give the title compound as a white solid (0.45 g, 79%). Mp = 45 °C. ^1^H NMR (400 MHz, CDCl_3_) *δ* 6.46 (br, 2 H, Ph*H*), 6.40 (t, 1 H, Ph*H*), 5.05 (s, 2 H, PhC*H*_2_OCO), 3.93 (t, 4 H, PhOC*H*_2_CH_2_CH_2_-), 3.47 (t, 2 H, -OCOCH_2_CH_2_C*H*_2_Br), 2.54 (t, 2 H, -OCOC*H*_2_CH_2_CH_2_Br), 2.20 (m, 2 H, -OCOCH_2_C*H*_2_CH_2_Br), 1.76 (m, 4 H, PhOCH_2_C*H*_2_CH_2_-), 1.45 (m, 4 H, PhOCH_2_CH_2_C*H*_2_-), 1.27 (m, 54 H, PhOCH_2_CH_2_CH_2_(C*H*_2_)_13_CH_3_ and PhOCH_2_CH_2_CH_2_(C*H*_2_)_14_CH_3_), 0.88 (t, 6 H, PhO(CH_2_)_16_C*H*_3_ and PhO(CH_2_)_17_C*H*_3_). ^13^C NMR (101 MHz, CDCl_3_) *δ* 172.4, 160.6, 137.9, 106.5, 101.2, 68.2, 66.5, 32.8, 32.6, 32.1, 32.0, 29.8, 29.8, 29.8, 29.7, 29.7, 29.7, 29.5, 29.5, 29.5, 29.4, 27.9, 26.2, 22.8, 14.3. Purity by HPLC: 99+%. MALDI-TOF MS *m*/*z* of [M + H]^+^ calculated for C_46_H_84_BrO_4_: 779.6; found: 780.5. [M + Na]^+^ calculated for C_46_H_83_BrNaO_4_: 801.5; found: 802.6. [M + K]^+^ calculated for C_46_H_83_BrKO_4_: 817.5; found: 818.6 (Figure S6). 

#### 2.3.7. Synthesis of 3-(Heptadecyloxy)-5-(octadecyloxy)benzyl 4-(4-methylpiperazin-1-yl)butanoate, **38g**, IAJD242

3-(Heptadecyloxy)-5-(octadecyloxy)benzyl 4-(4-methylpiperazin-1-yl)butanoate, **38g,** IAJD242, was synthesized using a modified literature procedure [38]. A mixture of 3-(heptadecyloxy)-5-(octadecyloxy)benzyl 4-bromobutanoate, **37d**, (0.18 g, 0.23 mmol), 1-methylpiperazine (25 mg, 0.25 mmol), K_2_CO_3_ (39 mg, 0.28 mmol), and MeCN (10 mL) was stirred at 95 °C for 3 h. The reaction mixture was cooled to 23 °C, and MeCN was removed under reduced pressure. Then, water (20 mL) was added, and the resulting mixture was extracted with DCM (20 mL) three times. The organic phases were collected and dried over anhydrous MgSO_4_. After filtration, the obtained filtrate was concentrated to around 3 mL and purified via column chromatography (SiO_2_) with DCM/MeOH = 30/1 and 15/1 as the eluent. Then, the obtained product was dissolved in DCM (20 mL), which was washed using a NaHCO_3_ solution (2%, 20 mL). The aqueous phase was extracted via DCM (20 mL) another two times. The organic phases were combined and dried over anhydrous MgSO_4_. The filtration and evaporation of the solvent yielded the title compound as a colorless oil (0.18 g, 99%). ^1^H NMR (400 MHz, CDCl_3_) *δ* 6.44 (br, 2 H, Ph*H*), 6.37 (t, 1 H, Ph*H*), 5.01 (s, 2 H, PhC*H*_2_-), 3.90 (t, 4 H, PhOC*H*_2_-), 2.59–2.25 (m, 15 H, -N(C*H*_2_C*H*_2_)_2_N-, -NC*H*_2_CH_2_C*H*_2_COO- and -NC*H*_3_), 1.83 (m, 2 H, -OCOCH_2_C*H*_2_CH_2_-), 1.77 (m, 4 H, PhOCH_2_C*H*_2_-), 1.42 (m, 4 H, PhOCH_2_CH_2_C*H*_2_-), 1.27 (br, 54 H, PhOCH_2_CH_2_CH_2_(C*H*_2_)_13_CH_3_ and PhOCH_2_CH_2_CH_2_(C*H*_2_)_14_CH_3_), 0.86 (t, 6 H, -CH_2_CH_2_C*H*_3_). ^13^C NMR (101 MHz, CDCl_3_) *δ* 173.3, 160.5, 138.2, 106.5, 101.0, 68.2, 66.2, 57.6, 55.1, 52.9, 45.9, 32.3, 32.0, 29.8, 29.8, 29.7, 29.7, 29.5, 29.5, 29.4, 26.2, 22.8, 22.2, 14.2. Purity by HPLC: 99+%. MALDI-TOF MS *m*/*z* of [M + H]^+^ calculated for C_51_H_95_N_2_O_4_: 799.7; found: 799.8. [M + Na]^+^ calculated for C_51_H_94_N_2_NaO_4_: 821.7; found: 821.7. [M + K]^+^ calculated for C_51_H_94_KN_2_O_4_: 837.6; found: 837.6 (Figure S7).

#### 2.3.8. Synthesis of 3-(Heptadecyloxy)-5-(octadecyloxy)benzyl 4-(4-(2-hydroxyethyl)piperazin-1-yl)butanoate, **38h**, IAJD243

3-(Heptadecyloxy)-5-(octadecyloxy)benzyl 4-(4-(2-hydroxyethyl)piperazin-1-yl)butanoate, **38h,** IAJD243, was synthesized using a modified literature procedure [38]. A mixture of 3-(heptadecyloxy)-5-(octadecyloxy)benzyl 4-bromobutanoate, **37d**, (0.18 g, 0.23 mmol), 1-(2-hydroxyethyl)piperazine (33 mg, 0.25 mmol), K_2_CO_3_ (39 mg, 0.28 mmol), and MeCN (10 mL) was stirred at 95 °C for 3 h. The reaction mixture was cooled to 23 °C, and MeCN was removed under reduced pressure. Then, water (20 mL) was added, and the resulting mixture was extracted with DCM (20 mL) three times. The organic phases were collected and dried over anhydrous MgSO_4_. After filtration, the obtained filtrate was concentrated to around 3 mL and purified via column chromatography (SiO_2_) with DCM/MeOH = 30/1 and 15/1 as the eluent. Then, the obtained product was dissolved in DCM (20 mL), which was washed using a NaHCO_3_ solution (2%, 20 mL). The aqueous phase was extracted via DCM (20 mL) another two times. The organic phases were combined and dried over anhydrous MgSO_4_. The filtration and evaporation of the solvent yielded the title compound as a colorless oil (0.18 g, 95%). ^1^H NMR (400 MHz, CDCl_3_) *δ* 6.45 (br, 2 H, Ph*H*), 6.38 (t, 1 H, Ph*H*), 5.02 (s, 2 H, PhC*H*_2_-), 3.91 (t, 4 H, PhOC*H*_2_-), 3.60 (t, 2 H, -CH_2_C*H*_2_OH), 2.63–2.31 (m, 14 H, -N(C*H*_2_C*H*_2_)_2_N-, -NC*H*_2_CH_2_C*H*_2_COO- and -C*H*_2_CH_2_OH), 1.84 (m, 2 H, -OCOCH_2_C*H*_2_CH_2_-), 1.74 (m, 4 H, PhOCH_2_C*H*_2_-), 1.43 (m, 4 H, PhOCH_2_CH_2_C*H*_2_-), 1.25 (br, 54 H, PhOCH_2_CH_2_CH_2_(C*H*_2_)_13_CH_3_ and PhOCH_2_CH_2_CH_2_(C*H*_2_)_14_CH_3_), 0.87 (t, 6 H, -CH_2_CH_2_C*H*_3_). ^13^C NMR (101 MHz, CDCl_3_) *δ* 173.4, 160.6, 138.2, 106.5, 101.0, 68.2, 66.3, 59.5, 57.8, 57.6, 53.0, 53.0, 32.3, 32.0, 29.8, 29.8, 29.7, 29.7, 29.5, 29.5, 29.4, 26.2, 22.8, 22.2, 14.2. Purity by HPLC: 99+%. MALDI-TOF MS *m*/*z* of [M + H]^+^ calculated for C_52_H_97_N_2_O_5_: 829.7; found: 829.8. [M + Na]^+^ calculated for C_52_H_96_N_2_NaO_5_: 851.7; found: 852.0. [M + K]^+^ calculated for C_52_H_96_KN_2_O_5_: 867.7; found: 867.9 (Figure S8).

#### 2.3.9. Synthesis of Methyl 3,5-bis(heptadecyloxy)benzoate), **64d**

**Scheme 2:**
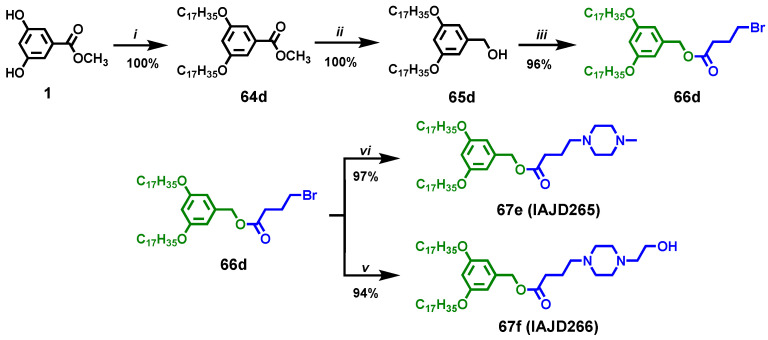
Synthesis of 3,5-symmetric sSS IAJDs: (i) C_17_H_35_OTs, K_2_CO_3_, DMF, 120 °C, 2 h; (ii) LiAlH_4_, THF, 0–23 °C, 1 h; (iii) DCC, DPTS, DCM 12 h; (iv) methylpiperazine, K_2_CO_3_, MeCN, 95 °C, 3 h; (v) 1-(2-hydroxyethyl)piperazine, K_2_CO_3_, MeCN, 95 °C, 3 h.

Methyl 3,5-dihydroxybenzoate, **1**, (0.40 g, 2.38 mmol, 1 equiv) (Scheme 2), C_17_H_35_OTs (2.30 g, 5.23 mmol, 2.2 equiv), K_2_CO_3_ (0.82 g, 5.95 mmol, 2.5 equiv), and DMF (20 mL) was heated to 120 °C and stirred under a N_2_ atmosphere for 2 h. The reaction mixture was cooled to 23 °C and poured into ice water (150 mL). The resulting white precipitate was filtered and collected. The solid precipitate was recrystallized from minimum acetone to afford the title compound as a white solid (1.33 g, 87%). Mp = 70 °C. ^1^H NMR (400 MHz, CDCl_3_) *δ* 7.16 (br, 2 H, Ph*H*), 6.63 (t, 1 H, Ph*H*), 3.96 (t, 4 H, PhOC*H*_2_CH_2_CH_2_-), 3.89 (s, 3 H, PhCOOC*H*_3_), 1.77 (m, 4 H, PhOCH_2_C*H*_2_CH_2_-), 1.45 (m, 4 H, PhOCH_2_CH_2_C*H*_2_-), 1.26 (m, 52 H, PhOCH_2_CH_2_CH_2_(C*H*_2_)_13_CH_3_), 0.88 (t, 6 H, PhO(CH_2_)_16_C*H*_3_). ^13^C NMR (101 MHz, CDCl_3_) *δ* 167.0, 160.2, 131.8, 107.6, 106.6, 77.2, 68.3, 64.1, 52.2, 31.9, 29.7, 29.7, 29.6, 29.6, 29.4, 29.2, 26.0, 25.8, 22.7, 14.1. Purity by HPLC: 99+%. MALDI-TOF MS *m*/*z* of [M + H]^+^ calculated for C_42_H_77_O_4_: 645.8; found: 646.6. [M + Na]^+^ calculated for C_42_H_76_NaO_4_: 667.6; found: 668.7. [M + K]^+^ calculated for C_42_H_76_KO_4_: 683.5; found: 682.6 (Figure S9).

#### 2.3.10. Synthesis of (3,5-Bis(heptadecyloxy)phenyl)methanol, **65d**

Methyl 3,5-bis(heptadecyloxy)benzoate), **64d**, (0.66 g, 1.02 mmol, 1 equiv) was dissolved in 10 mL dry THF, which was added dropwise to a slurry of LiAlH_4_ (38 mg, 1.02 mmol, 1 equiv) in dry THF (5 mL) at 0 °C under a N_2_ atmosphere. Afterward, the resulting mixture was stirred at 23 °C for 1 h. Finally, the reaction was quenched via the successive addition of water (0.2 mL), a 15% NaOH aqueous solution (0.2 mL), and water (1.0 mL). The white precipitate was filtered. The solid filtrate was dissolved in DCM, dried over anhydrous MgSO_4_, and filtered. The solvent was removed under reduced pressure to afford the title compound as a white solid (0.62 g, 100%). Mp = 64 °C. ^1^H NMR (400 MHz, CDCl_3_) *δ* 6.50 (br, 2 H, Ph*H*), 6.37 (t, 1 H, Ph*H*), 4.61 (s, 2 H, PhC*H*_2_OH), 3.93 (t, 4 H, PhOC*H*_2_CH_2_CH_2_-), 1.76 (m, 4 H, PhOCH_2_C*H*_2_CH_2_-), 1.45 (m, 4 H, PhOCH_2_CH_2_C*H*_2_-), 1.26 (m, 52 H, PhOCH_2_CH_2_CH_2_(C*H*_2_)_13_CH_3_), 0.88 (t, 6 H, PhO(CH_2_)_16_C*H*_3_). ^13^C NMR (101 MHz, CDCl_3_) *δ* 160.6, 143.2, 105.1, 100.6, 77.2, 68.1, 65.5, 34.0, 32.9, 31.9, 29.7, 29.7, 29.6, 29.6, 29.6, 29.5, 29.4, 29.4, 29.3, 28.8, 28.2, 26.1, 22.7, 14.1. Purity by HPLC: 99+%. MALDI-TOF MS *m*/*z* of [M + H]^+^ calculated for C_41_H_75_O_4_: 617.6; found: 618.3. [M + Na]^+^ calculated for C_41_H_76_NaO_4_: 639.6; found: 640.3. [M + K]^+^ calculated for C_41_H_76_KO_4_: 655.5; found: 656.6 (Figure S10).

#### 2.3.11. Synthesis of 3,5-Bis(heptadecyloxy)benzyl 4-bromobutanoate, **66d**

(3,5-Bis(heptadecyloxy)phenyl)methanol, **65d**, (0.62 g, 1.02 mmol, 1 equiv), 4-bromobutyric acid (0.20 g, 1.22 mmol, 1.2 equiv), DPTS (0.36 g, 1.22 mmol, 1.2 equiv) were dissolved in 10 mL DCM. *N*,*N*′-Dicyclohexylcarbodiimide, DCC, (0.42 g, 2.04 mmol, 2 equiv) was added into the above mixture. The reaction mixture was stirred at 23 °C for 12 h. Afterward, the resulting precipitate (urea) was filtered from the reaction mixture. The solid filtrate was concentrated to around 3 mL and purified via column chromatography (SiO_2_) with hexane/DCM = 1/1 as the mobile phase to give the title compound as a white solid (0.75 g, 96%). Mp = 55 °C. ^1^H NMR (400 MHz, CDCl_3_)*δ* 6.47 (br, 2 H, Ph*H*), 6.40 (t, 1 H, Ph*H*), 5.05 (s, 2 H, PhC*H*_2_OCO), 3.93 (t, 4 H, PhOC*H*_2_CH_2_CH_2_-), 3.47 (t, 2 H, -OCOCH_2_CH_2_C*H*_2_Br), 2.57 (t, 2 H, -OCOC*H*_2_CH_2_CH_2_Br), 2.20 (m, 2 H, -OCOCH_2_C*H*_2_CH_2_Br), 1.77 (m, 4 H, PhOCH_2_C*H*_2_CH_2_-), 1.45 (m, 4 H, PhOCH_2_CH_2_C*H*_2_-), 1.28 (m, 52 H, PhOCH_2_CH_2_CH_2_(C*H*_2_)_13_CH_3_), 0.88 (t, 6 H, PhO(CH_2_)_16_C*H*_3_). ^13^C NMR (101 MHz, CDCl_3_) *δ* 172.3, 160.5, 137.8, 106.4, 101.1, 77.2, 68.1, 66.4, 64.8, 32.7, 32.6, 32.5, 32.5, 31.9, 30.3, 29.7, 29.7, 29.6, 29.6, 29.6, 29.5, 29.4, 29.4, 29.3, 27.8, 27.8, 26.1, 25.9, 22.7, 14.1. Purity by HPLC: 99+%. MALDI-TOF MS *m*/*z* of [M + H]^+^ calculated for C_45_H_82_BrO_4_: 765.5; found: 767.0. [M + Na]^+^ calculated for C_45_H_81_BrNaO_4_: 787.5; found: 790.0. [M + K]^+^ calculated for C_45_H_81_BrKO_4_: 803.5; found: 805.9 (Figure S11).

#### 2.3.12. Synthesis of 3,5-Bis(pentadecyloxy)benzyl 4-(4-methylpiperazin-1-yl)butanoate. **67e**, IAJD265 

3,5-Bis(pentadecyloxy)benzyl 4-(4-methylpiperazin-1-yl)butanoate, **67e**, IAJD265, was synthesized using a modified literature procedure [38]. A solution of 3,5-bis(heptadecyloxy)benzyl 4-bromobutanoate, **66d**, IAJD 264, (300 mg, 0.39 mmol, 1 equiv), 1-methylpiperazine (47 mg, 0.47 mmol, 1.2 equiv), and K_2_CO_3_ (81 mg, 0.59 mmol, 1.5 equiv) in 20 mL MeCN was heated to 95 °C and stirred for 3 h. The reaction mixture was cooled to 23 °C, and MeCN was removed under reduced pressure. Then, water (20 mL) was added, and the resulting mixture was extracted with DCM (20 mL) three times. The organic phases were collected and dried over anhydrous MgSO_4_. After filtration, the obtained filtrate was concentrated to around 3 mL and purified via column chromatography (SiO_2_) with DCM/MeOH = 30/1 and 15/1 as the eluent. The obtained product was dissolved in DCM (20 mL) and washed using a NaHCO_3_ solution (2%, 20 mL). The aqueous phase was extracted via DCM (20 mL) another two times. The organic phases were combined and dried over anhydrous MgSO_4_. The filtration and evaporation of the solvent yielded the title compound as a colorless oil (0.50 g, 97%). ^1^H NMR (400 MHz, CDCl_3_) *δ* 6.46 (br, 2 H, Ph*H*), 6.39 (t, 1 H, Ph*H*), 5.02 (s, 2 H, PhC*H*_2_-), 3.92 (t, 4 H, PhOC*H*_2_-), 2.63–2.25 (m, 15 H, -N(C*H*_2_C*H*_2_)_2_N-, -NC*H*_2_CH_2_C*H*_2_COO- and -NC*H*_3_), 1.83 (m, 2 H, -OCOCH_2_C*H*_2_CH_2_-), 1.73 (m, 4 H, PhOCH_2_C*H*_2_-), 1.42 (m, 4 H, PhOCH_2_CH_2_C*H*_2_-), 1.26 (br, 52 H, PhOCH_2_CH_2_CH_2_(C*H*_2_)_13_CH_3_), 0.88 (t, 6 H, -CH_2_CH_2_C*H*_3_). ^13^C NMR (101 MHz, CDCl_3_) *δ* 173.3, 160.5, 138.1, 106.4, 100.9, 77.2, 68.1, 66.2, 57.6, 55.2, 53.1, 46.1, 32.3, 31.9, 29.7, 29.7, 29.6, 29.6, 29.4, 29.4, 29.3, 26.1, 22.7, 22.2, 14.1. Purity by HPLC: 99+%. MALDI-TOF MS *m*/*z* of [M + H]^+^ calculated for C_50_H_93_N_2_O_4_: 785.7; found: 785.9. [M + Na]^+^ calculated for C_50_H_92_N_2_NaO_4_: 807.7; found: 808.9. [M + K]^+^ calculated for C_50_H_92_KN_2_O_4_: 823.7; found: 835.6 (Figure S12).

#### 2.3.13. Synthesis of 3,5-Bis(pentadecyloxy)benzyl 4-(4-(2-hydroxyethyl)piperazin-1-yl)butanoate, **67f**, IAJD266

3,5-Bis(pentadecyloxy)benzyl 4-(4-(2-hydroxyethyl)piperazin-1-yl)butanoate, **67f**, **IAJD266**, was synthesized using a modified literature procedure [38]. A solution of 3,5-bis(heptadecyloxy)benzyl 4-bromobutanoate, **66d**, IAJD 264, (300 mg, 0.39 mmol, 1 equiv), 1-(2-hydroxyethyl)piperazine (62 mg, 0.47 mmol, 1.2 equiv), and K_2_CO_3_ (81 mg, 0.59 mmol, 1.5 equiv) in 20mL MeCN was heated to 95 °C and stirred for 3 h. The reaction mixture was cooled to 23 °C, and MeCN was removed under reduced pressure. Then, water (20 mL) was added, and the resulting mixture was extracted with DCM (20 mL) three times. The organic phases were collected and dried over anhydrous MgSO_4_. After filtration, the obtained filtrate was concentrated to around 3 mL and purified via column chromatography (SiO_2_) with DCM/MeOH = 30/1 and 15/1 as the eluent. The obtained product was dissolved in DCM (20 mL) and washed using a NaHCO_3_ solution (2%, 20 mL). The aqueous phase was extracted via DCM (20 mL) another two times. The organic phases were combined and dried over anhydrous MgSO_4_. The filtration and evaporation of the solvent yielded the title compound as a colorless oil (0.50 g, 94%). ^1^H NMR (400 MHz, CDCl_3_) *δ* 6.46 (br, 2 H, Ph*H*), 6.39 (t, 1 H, Ph*H*), 5.01 (s, 2 H, PhC*H*_2_-), 3.89 (t, 4 H, PhOC*H*_2_-), 3.61 (t, 2H, -CH_2_C*H*_2_OH), 2.62–2.31 (m, 14 H, -N(C*H*_2_C*H*_2_)_2_N-, -NC*H*_2_CH_2_C*H*_2_COO- and -C*H*_2_CH_2_OH), 1.82 (m, 2 H, -OCOCH_2_C*H*_2_CH_2_-), 1.72 (m, 4 H, PhOCH_2_C*H*_2_-), 1.40 (m, 4 H, PhOCH_2_CH_2_C*H*_2_-), 1.28 (br, 52 H, PhOCH_2_CH_2_CH_2_(C*H*_2_)_13_CH_3_), 0.86 (t, 6 H, -CH_2_CH_2_C*H*_3_). ^13^C NMR (101 MHz, CDCl_3_) *δ* 173.3, 160.5, 138.1, 106.4, 100.9, 77.2, 68.1, 66.2, 59.3, 57.6, 57.5, 53.0, 52.8, 32.2, 31.9, 31.8, 29.5, 29.4, 29.4, 29.3, 29.2, 26.1, 22.7, 22.1, 14.1. Purity by HPLC: 99+%. MALDI-TOF MS *m*/*z* of [M + H]^+^ calculated for C_51_H_95_N_2_O_5_: 815.7; found: 815.6. [M + Na]^+^ calculated for C_51_H_94_N_2_NaO_5_: 837.7; found: 837.7. [M + K]^+^ calculated for C_51_H_94_KN_2_O_5_: 853.7; found: 853.7 (Figure S13).

## 3. Results and Discussion

### 3.1. Concept, Strategy, and Methodology

IAJDs are stable for a long time after being stored in a chemistry laboratory at room temperature in air, while their DNPs are stable and remain active for at least 5 months at the normal refrigerator temperature of 5 °C [24,25,26]. The original architecture of one-component IAJDs [24,25,26] was inspired by the structure of amphiphilic Janus dendrimers (JDs) [39,40,41,42,43,44,45,46,47,48,49,50,51], Janus glycodendrimers (JGDs) [52,53,54,55,56,57,58,59,60,61,62,63,64,65,66,67,68,69,70], and sequence-defined JGDs [71,72] and will be discussed in more detail later.

JDs and JGDs are self-assembled through the simple injection of their ethanol solution into water or buffer to produce monodisperse vesicles known as dendrimersomes and glycodendrimersomes, with predictable dimensions [40]. The physical properties of dendrimersomes are as good as those of three-component stealth liposomes co-assembled from phospholipids, PEG-conjugated lipids, and cholesterol [73,74,75]. The three-component concept of stealth liposomes [73,74,75,76] inspired the elaboration of the four-component LNPs (Figure 1A) currently employed to deliver mRNA. The mechanism for the delivery of mRNA is most probably closely related to the mechanisms of both four-component LNPs and one-component DNPs (Figure 1C), although their structures are quite different (Figure 1A,B). 

LNPs contain the mRNA encapsulated together with ionizable lipids in the center of a stealth liposome (Figure 1A), while DNPs may contain the mRNA encapsulated as a spherical helix (Figure 1B) that provides the largest number of contacts between the nucleic acid and dendrimersome. 

The ability to molecularly design both the hydrophilic and the hydrophobic parts of one-component IAJDs provides synthetic advantages that will be demonstrated in this publication. Oligooxyethylene fragments were originally employed to design the multifunctional sequence-defined hydrophilic region and generate single–single, (SS, single hydrophilic dendron connected to single hydrophobic dendron), twin–twin (TT, two hydrophilic dendrons connected to two hydrophobic dendrons), and twin–mixed (TM, two different hydrophilic dendrons connected to two hydrophobic dendrons) IAJDs (Figure 1B) [24]. A simplified SS, sSS architecture was also generated, which resulted from the removal of the oligooxyethylene fragments from the hydrophilic dendron while maintaining only its ionizable amine, which, after protonation, becomes hydrophilic and active to mRNA binding (Figure 1B) [24]. 

In all cases, hydrophobic dendrons were constructed mostly from 3,5-substituted, with only very few preliminary examples of 3,4- and 3,4,5-substituted plant phenolic acids or trisubstituted pentaerythritol containing identical alkyl groups. Sequence-defined JGDs were demonstrated to be extraordinarily important in providing the highest activity of their glycan when binding to sugar-binding proteins known as lectins and galectins [52,53,54,55,56,57]. Sequence-defined self-assembling dendronized perylenebisimides (PBIs) were also demonstrated to dramatically change their rate of self-organization process via a cogwheel mechanism accompanied by deracemization in a crystal state [76,77,78,79,80]. In addition, phospholipids forming the bilayer of natural cell membranes are generated by a nonsymmetric substitution of their glycerol with alkyl groups containing a different number of *cis*-double bonds to introduce their homochiral stereocenter [81]. This arrangement provides dissimilar chain lengths in the bilayer of vesicles even for an identical number of carbons in their alkyl groups but different numbers of *cis*-double bonds. With the currently available results, the mechanism of the self-assembly of JDs into DNPs was shown to be preferred by the 3,5- rather than 3,4- or 3,4,5-substitution pattern since the 3,5-substitution pattern favors the interdigitation of its alkyl groups in the hydrophobic part of the bilayer of the vesicle and stabilizes the mechanical integrity of the assembly [40]. 

These literature data prompted us to advance the hypothesis that a sequence-defined arrangement in the hydrophobic part of IAJDs could also be influential, as it was shown to be important in the hydrophilic part, on the activity of targeted delivery of mRNA mediated by IAJDs [24,25,26]. This was already indicated with preliminary experiments [26] by replacing their identical alkyl group lengths with dissimilar alkyl group lengths in their hydrophobic part to create nonsymmetric SS (nSS) and nonsymmetric simplified SS (nsSS) (Figure 1B) and testing them for their in vitro and in vivo delivery of mRNA. 

Originally, this combination of dissimilar alkyl groups in their hydrophobic part was hypothesized to decrease the crystallization tendency of the hydrophobic part of the bilayer containing long alkyl groups and increase the solubility of the IAJD in ethanol. The decreased rate of crystallization and increased solubility of IAJD were indeed accomplished using this methodology, but unexpectedly, it was also accompanied by increased activity of mRNA delivery by the corresponding DNP to specific organs [24,25,26]. 

Figure 1A outlines the structure and co-assembly with the mRNA of four-component LNPs [25]. Figure 1B shows the structures of sSS, SS, and TM IAJDs with dissimilar (nsSS, nSS) and similar alkyl groups in their hydrophobic parts and their co-assembly with mRNA into DNPs. Figure 1C illustrates the current mechanism accepted for the in vivo delivery of mRNA with both four-component LNPs and one-component DNPs. This mechanism involves the endocytosis of LNPs or DNPs, followed by the endosomal escape of mRNA and the synthesis of a protein in collaboration with the ribosome. 

Since normal mRNA experiments can take extended periods of time, especially in the case of vaccine experiments, we employed firefly luciferase mRNA (Luc-mRNA) that provides important screening results in several hours [24,25,26]. In this case, the protein translated in the cell is the luciferase enzyme that interacts with D-luciferin to generate oxy-luciferin emitting light that helps to identify rapidly the organ targeted. 

### 3.2. Accelerated Modular–Orthogonal Synthesis of Two Libraries of nsSS IAJDs

**Figure 2:**
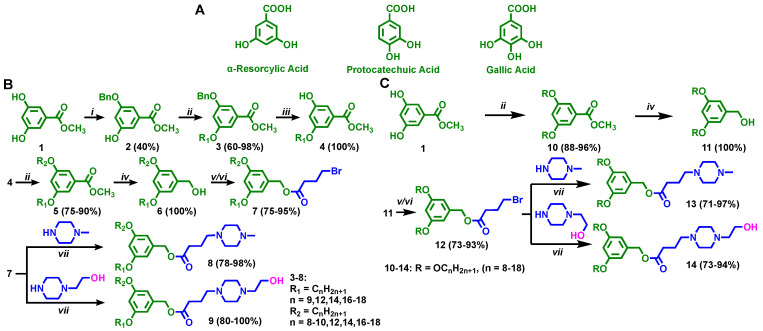
Structures of plant phenolic acids: α-resorcylic acid, protocatechuic acid, and gallic acid and synthesis of nsSS IAJDs containing 3,5-nonsymmetric alkyl groups as well as of sSS IAJDs containing 3,5-symmetric alkyl groups: (**A**) structures of plant phenolic acids employed as starting materials in the synthesis of IAJDs; (**B**) accelerated modular–orthogonal synthesis of nsSS IAJDs based on 3,3-dihydroxybenzoate; (**C**) accelerated modular–orthogonal synthesis of sSS IAJDs based on 3,4-dihydroxybenzoate. Reagents and Conditions: (i) BnCl, K_2_CO_3_, KI, DMF, 80 °C, 5 h; (ii) RBr, K_2_CO_3_, DMF, 120 °C, 2 h; (iii) H_2_, Pd/C, DCM, MeOH, 12 h; (iv) LiAlH_4_, THF, 0–23 °C, 1 h; (v) 4-bromobutyric acid, SOCl_2_, DMF (cat.), DCM, 23 °C, 1 h, then DMAP, NEt_3_, DCM, 0–23 °C, 2 h; (vi) DCC, DPTS, 12 h; (vii) methylpiperazine or hydroxyethyl piperazine, K_2_CO_3_, MeCN, 95 °C, 3 h.

Figure 2A shows the structures of the 3,5-dihydroxy, 3,4-dihydroxy, and 3,4,5-trihydroxy plant phenolic acids known by common names as α-resorcylic, protocatechuic, and gallic acid, respectively [82], that were used by our laboratory in the synthesis of a larger diversity of complex systems [50] including IAJDs [24,25,26]. Due to their antioxidant activity, some of these phenolic acid derivatives are used as food additives and are present in vegetable oils, butter, fats, meat, and many other daily components of our food [83]. 

Here, using the accelerated modular–orthogonal methodology outlined in Figure 2B, we will first report the synthesis of two libraries of nsSS IAJDs containing 78 molecules based on 3,5-dihydroxy phenolic acid containing methyl piperazine and hydroxyethyl piperazine as ionizable amines. The synthesis of a single nSS IAJD-131 (Supporting Scheme S10) is also reported. nsSS IAJDs with various combinations of *n*-alkyl groups containing 18, 17, 16, 14, 12, 10, 9, 8, and 2-ethyl hexyl (EH) were synthesized. Linear and branched alkyl groups were employed since they do not oxidize and therefore dramatically increase the stability of IAJDs by comparison with that of four-component systems containing unsaturated phospholipids. 

In the first step of their synthesis, the 3-benzylether of 3,5-dihydroxy methyl benzoate was produced in 40% isolated yield in 5 h via the etherification of **1** with BnCl at 80 °C in DMF with K_2_CO_3_ base. Subsequently, **2** was alkylated with a 1-bromoalkyl group in DMF, with K_2_CO_3_ base at 120 °C, to produce a 60% to 98% isolated yield of **3**. The hydrogenolysis of **3** (H_2_/Pd, DCM/MeOH, 12 h) generated **4** in 100% isolated yield. The alkylation of **4** with various linear and branched alkyl bromides from 1-bromooctane to 1-bromooctadecane in DMF, with K_2_CO_3_ base at 120 °C, created the libraries of the nonsymmetric compounds **5** in 75–90% isolated yield.

The reduction of compounds **5** with LiAlH_4_ in THF (0–23 °C, 1 h) generated the benzyl alcohols **6** in 100% isolated yield. Compounds **6** were esterified with 4-bromobutyric acid either via its acid chloride generated with SOCl_2_ catalyzed via DMF in CH_2_Cl_2_ at 23 °C followed by reaction in the presence of NEt_3_/DMAP (0–23 °C, 2 h) or via the direct esterification of **6** with DCC/DPTS in 12 h to produce compounds **7** in 75% to 95% isolated yield. Compounds **7** were reacted with methyl piperazine or hydroxyethyl piperazine (K_2_CO_3_, MeCN, 95 °C, 3 h) to give rise to IAJDs **8** (78–98% isolated yields) and **9** (80–100% isolated yields). Their purity using a combination of thin-layer chromatography (TLC), high-pressure liquid chromatography (HPLC), matrix-assisted laser desorption/ionization–time-of-flight (MALDI-TOF) mass spectroscopy, ^1^H-NMR, and ^13^C-NMR was higher than 99%.

### 3.3. Accelerated Modular–Orthogonal Synthesis of a Complete Library of sSS IAJDs

In order to finalize the few experiments reported previously on sSS IAJDs [25,26] and provide a complete library, we employed the methodology from Figure 2C for their synthesis. The dialkylation of **1** was accomplished at 120 °C in DMF with K_2_CO_3_ as the base in 2 h to produce compounds **10** in 88% to 96% isolated yields. The reduction of **10** with LiAlH_4_ for 1 h in THF at 0–23 °C generated **11** in 100% yield. Compound **12** (73–93% isolated yields) was prepared as in the case of **7** (Figure 2B,C). Compounds **13** (71–97% isolated yields) and **14** (73–94% isolated yields) were obtained under similar reaction conditions to **8** and **9**. Their structures are shown in Figure 2C.

**Figure 3:**
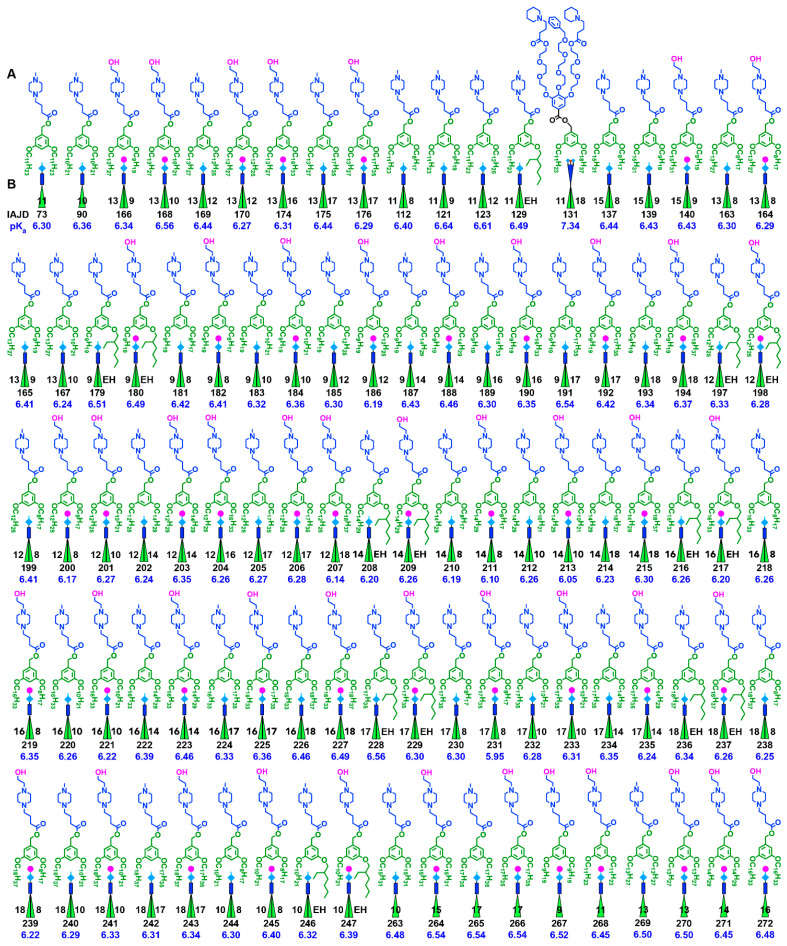
The structures of nsSS IAJDs with 3,5-nonsymmetric alkyl chains and of nSS IAJD 131. IAJD numbers and pK_a_ values are shown under corresponding schematic representations of nsSS and nSS IAJDs: (**A**) structures of IAJDs; (**B**) schematic representations of IAJDs with the number of carbons in the alkyl groups from their hydrophobic part on the left and right sides of the structure; IAJD numbers in black under the schematic of IAJD, and pKa values in blue under the number of IAJD.

The structures of all 98 nsSS and sSS IAJDs are shown in Figure 3 (IAJDs 112 to 272) together with IAJD 131. Figure 3 also outlines a simplified cartoon illustration of the structure of each IAJD together with the number of carbons in the pair of their alkyl groups, the number of the IAJD structure, and the pKa values shown in blue under the IAJD numbers in black. The schematic illustration from Figure 3 will simplify the discussion in the rest of this publication. Two libraries of IAJDs containing 85 nsSS, 43 containing methylpiperazine, and 42 hydroxyethyl piperazine ionizable amines were synthesized using the methodology elaborated in Figure 2B. All of them are shown in Figure 3, together with the nSS IAJD 131, whose synthesis is outlined in Supporting Schemes S9 and S11 sSS that were synthesized as outlined in Figure 2B. Therefore, a total of 98 IAJDs together with their complexes with mRNA known as dendrimersome nanoparticles (DNPs) will be discussed in the following subsections. We must clarify again that DNPs are in fact vesicles with a stability closely related to that of viral capsids rather than to the stability of LNPs. As we can see from the structures of Figure 3, all these IAJDs are based on first-generation dendrons that were defined by our laboratory, via analogy with minipeptides and miniproteins as minidendrons and minidendrimers [50]. In this particular case, they contain one or two branching points in their hydrophobic part, while the hydrophilic part contains branching points derived from their ionizable amine.

### 3.4. Co-Assembly of IAJDs with Luc-mRNA into Dendrimersome Nanoparticles (DNPs) via Simple Injection

**Figure 4:**
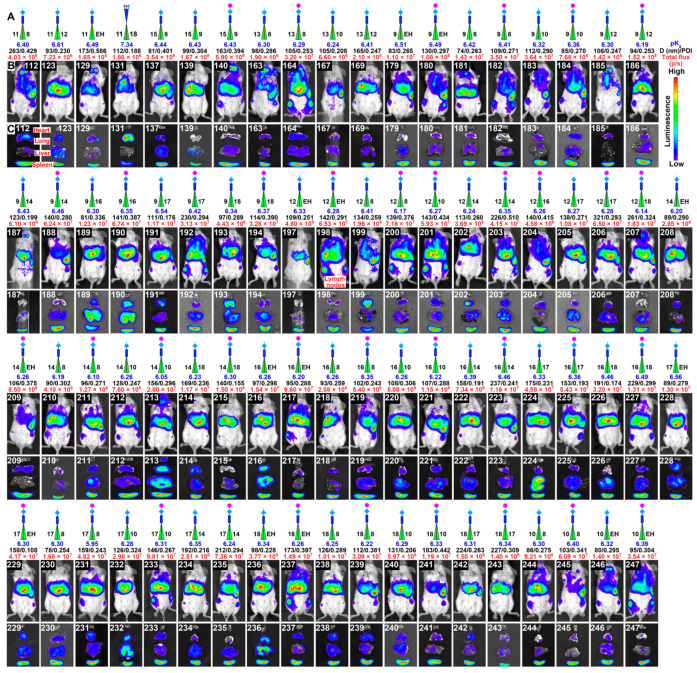
Delivery of Luc-mRNA in vivo by DNPs assembled from IAJDs with 3,5-nonsymmetric alkyl chains: (**A**) schematic representation of the IAJD structures with the number of the carbons in the hydrophobic part on the left and right sides of IAJD, pKa values under the schematic in blue, dimensions and polydispersity under pKa in black, and total flux luminescence activity (p/s) in red; (**B**) the representative image of luciferase expression in the whole body or organs 4–6 h post-intravenous (i.v.) injection of Luc-mRNA co-assembled with various IAJDs; (**C**) the images of organs with luciferase activity shown in the color code from the top right column. Color code: pink is hydroxyethyl, the light blue square is piperazine, and the perpendicular line on the square in blue is methyl, dark blue is the hydrophilic fragment, and green is the hydrophobic fragment of IAJD.

The injection method elaborated for the self-assembly of dendrimersomes from amphiphilic Janus dendrimers [39] and glycodendrimersomes from Janus glycodendrimers [52] was adopted for the self-assembly of IAJDs [24,25,26] and their co-assembly with mRNA into DNPs [24,25,26]. Narrow polydispersity DNPs [24,25,26] with predictable dimensions, as obtained in the case of dendrimersomes [39] and glycodendrimersomes [52], are accessible via the simple injection of an ethanol solution of IAJD into an acidic solution of Luc-mRNA of molar mass 661,050 daltons. This method facilitates fast access to the DNPs employed for in vivo mice transfection experiments. We must stress that our DNPs are in fact dendrimersome vesicles containing mRNA encapsulated in their interior most probably in a spherical helix conformation (Figure 1B). Their vesicular structure was demonstrated previously [24]. However, the mechanism of mRNA encapsulation and the structure of the DNPs containing mRNA are under investigation. The details of the co-assembly method via injection are described as follows: Nucleoside-modified mRNA encoding firefly luciferase (Luc-mRNA) was dissolved in UltraPure DNase/RNase-free distilled water with an initial concentration of 4.0 mg/mL. IAJDs were dissolved in ethanol with an initial concentration of 80 mg/mL. The Luc-mRNA solution (12.5 μL) was placed into a clean RNA-free Eppendorf tube (1.5 mL). Then, 463 μL of acetate buffer (10 mM, pH 4.0) was added. Afterward, 25 μL of IAJD in the ethanol stock solution was taken and rapidly injected above the Luc-mRNA solution in acetate buffer, followed by vortex for 5 s. The size and the size distribution of the resulting nanoparticles were determined using DLS and are reported in Figure 4 under the schematic illustration of the corresponding IAJDs. More than 98% of Luc-mRNA was encapsulated in the interior of DNPs that are stable and active at 5 °C for more than 135 days. These data will be reported soon.

### 3.5. In Vivo Delivery of Mice with DNPs Containing Luc-mRNA 

Female or male BALB/c mice, 6–8 weeks old, from Charles River Laboratories, were anesthetized with 3% isoflurane from Piramal Healthcare Limited and injected via retro-orbital sinus with 100 μL of DNP solution containing 10 μg of Luc-mRNA. At 4–6 h post-injection, mice were intraperitoneally (i.p.) injected with D-luciferin at a dose of 150 mg/kg of body weight, from Regis Technologies and imaged on a PerkinElmer In Vivo Imaging System (IVIS) Spectrum CT. Ten minutes after the administration of D-luciferin, mice were placed on the imaging platform while being maintained on isoflurane via a nose cone, and whole-body luminescence signal was measured on the IVIS imaging using a certain exposure time (60, 30, or 15 s). The tissue luminescence signal was measured on the IVIS imaging system using a certain exposure time (60, 30, or 15 s) and medium binning (binning = 8) to ensure that the signal obtained was within the operative detection range. For the IVIS imaging of the organs, mice were sacrificed; the heart, lungs, liver, and spleen were immediately collected; and bioluminescence imaging was performed as described above. Image analysis was conducted with the Living Image software from PerkinElmer. Bioluminescence values were quantified by measuring photon flux (photons/second) in the region of interest (ROI) using the Living Image software and were then analyzed. Figure 4 reports the in vivo transfection results for all 78 IAJDs synthesized, together with their organ analysis and total flux luminescence results. The remarkable first conclusion of the complex Figure 4 is that all the IAJDs are active for the delivery of Luc-mRNA, even if these are only fast screening experiments. We must stress that both the co-assembly with mRNA and transfection experiments performed in vivo were not yet optimized.

### 3.6. In Vivo Delivery of Luc-mRNA with nsSS DNPs to Spleen and Liver

**Figure 5:**
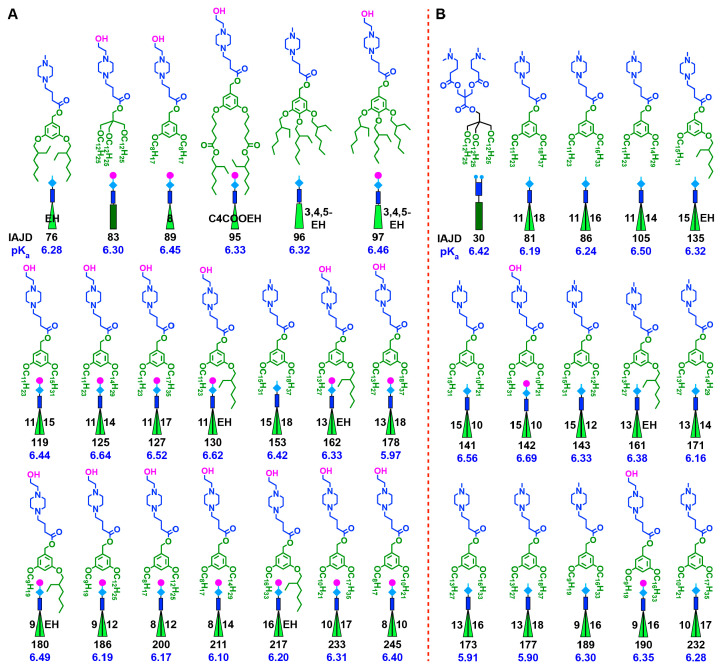
Structures of 15 most active IAJDs for selective delivery of mRNA to the liver and 20 most active IAJDs for selective delivery of mRNA to the spleen: (**A**) IAJDs delivering to the spleen; (**B**) IAJDs delivering to the liver. Legend: schematic representations of IAJDs with the number of carbons in the alkyl groups from their hydrophobic part on the left and right sides of the structure, IAJD numbers in black under the schematic of IAJD, and pKa values in blue under the number of IAJD.

A detailed discussion of these results is provided by grouping these results into targeted delivery to specific organs and correlating the molecular structure of the IAJD with the specific target and activity. We decided to group these experiments in specific delivery to the spleen, lymph nodes, liver, and lung. The twenty most active nsSS and sSS IAJDs that deliver predominantly to the spleen are shown in Figure 5A, while the fifteen most active nsSS and SS IAJDs delivering to the liver are presented in Figure 5B. There is a striking difference between the nsSS and sSS IAJDs delivering to the spleen (Figure 5A) vs. the one delivering to the liver (Figure 5B). Seventeen out of the twenty (85%) nsSS IAJDs that deliver to the spleen contain hydroxyethyl piperazine as an ionizable amine in their hydrophilic part, while the other three contain methyl piperazine (Figure 5A). By contrast, twelve out of the fifteen (80%) nsSS JAJDs that deliver to the liver contain the methyl piperazine ionizable amine in their hydrophilic part, two carry hydroxyethyl piperazine, and only one contains dimethyl amino group as ionizable amine (Figure 5B). 

The pKa values of the IAJDs delivering to the spleen are between 6.10 and 6.64, while the pKa values of the compounds targeting the liver are between 5.90 and 6.69. At the extracellular pH of 7.4 (Figure 1C), the exterior of DNPs co-assembled from these IAJDs with Luc-mRNA becomes deprotonated. These results demonstrate that the target of the delivery is provided via the structure of the ionizable amine from the hydrophilic part of the IAJD.

**Figure 6:**
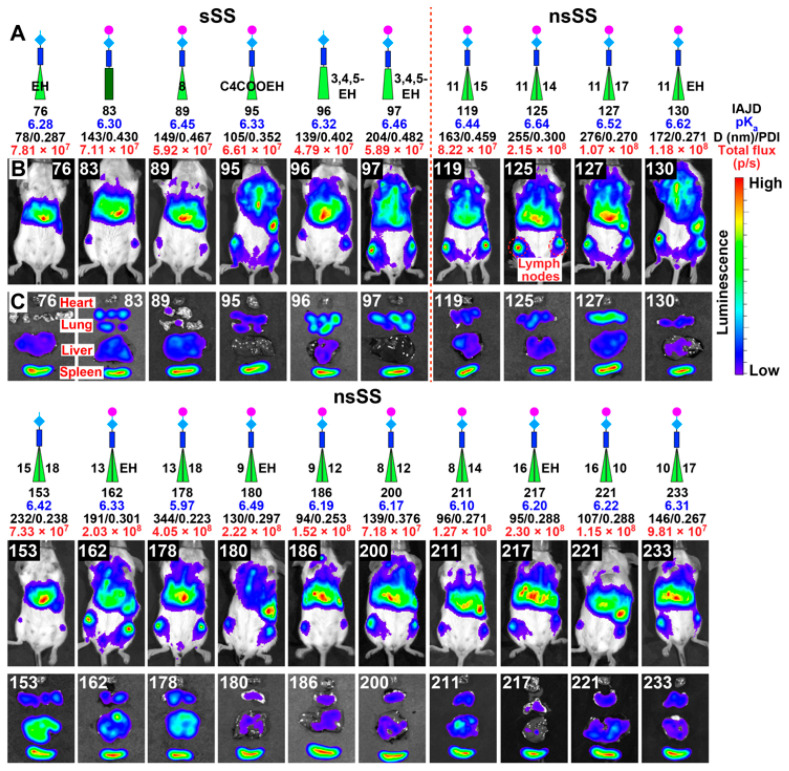
Selective delivery of Luc-mRNA in vivo by 20 most active DNPs to the spleen: (**A**) schematic representation of the IAJD structures with the number of the carbons in the hydrophobic part on the left and right sides of IAJD, pKa values under the schematic in blue, dimensions and polydispersity under pKa in black, and total flux luminescence activity (p/s) in red; (**B**) the representative images of luciferase expression in the whole body or organs after i.v. injection of Luc-mRNA co-assembled with IAJDs; (**C**) the images of organs with luciferase activity shown in color coding from the top right column; color code: pink is hydroxyethyl, the light blue square is piperazine, and the perpendicular line on the square in blue is methyl, dark blue is the hydrophilic fragment, and green is the hydrophobic fragment of IAJD.

**Figure 7:**
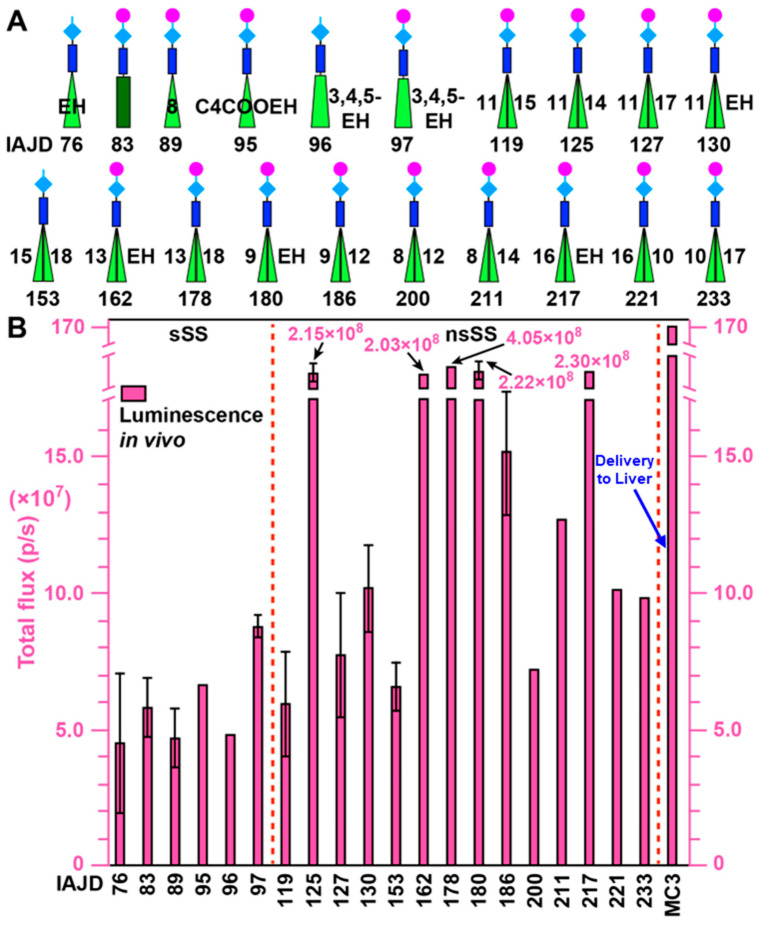
Comparison of the activities of the 20 most active DNPs that deliver mRNA to the spleen: (**A**) schematic representations of IAJDs with the number of carbons in the alkyl groups from their hydrophobic part on the left and right sides of the structure, and IAJD numbers in black under the schematic of IAJD. MC3 delivers mRNA to the liver; (**B**) plots of total flux luminescence (p/s) for the DNPs assembled from the IAJDs from (**A**).

The activity of the IAJDs from Figure 5A in their delivery to the spleen is shown in Figure 6. Most of these IAJDs deliver mRNA to the spleen and lymph nodes. Without any optimization, eleven nsSS IAJDs (55% of them), namely 125, 127, 130, 162, 178, 180, 186, 211, 217, 221, and 233, exhibit the total flux of their luminescence very close to or higher than 10^8^ p/s. All the other sSS and nsSS IAJDs display the total flux of their luminescence in the range of high 10^7^ p/s values. It is very interesting to observe that the most active of all the IAJDs contain between three and six methylenic units difference between the number of carbons in their nonsymmetric 3,5-disubstituted hydrophobic part. This is a quite remarkable result considering that most of these results were obtained only from screening experiments without any optimization, as shown in the plots from Figure 7. 

**Figure 8:**
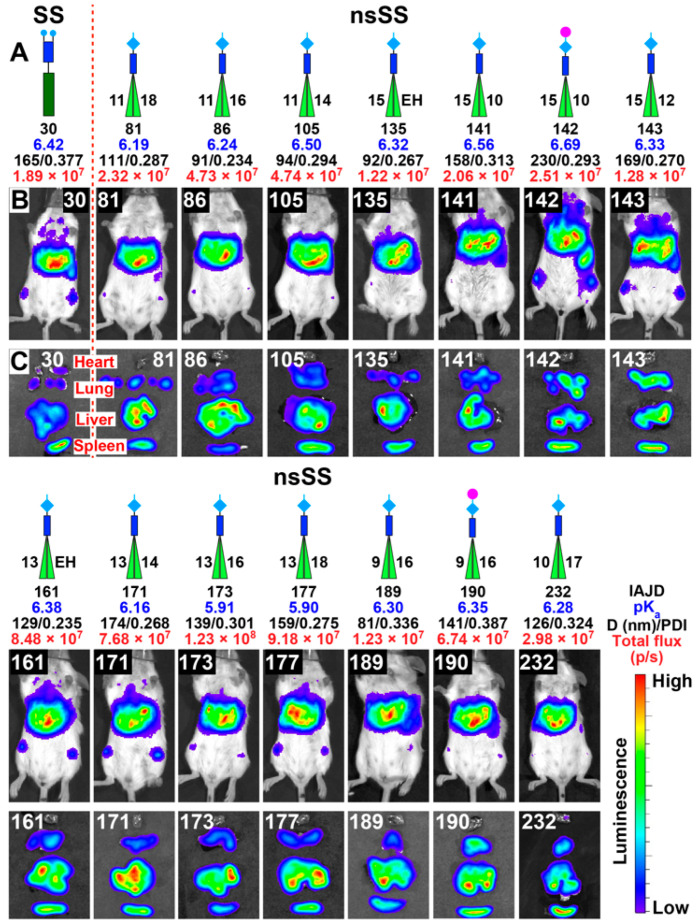
Selective delivery of Luc-mRNA in vivo by 15 most active DNPs to the liver: (**A**) schematic representation of the IAJD structures with the number of the carbons in the hydrophobic part on the left and right sides of IAJD, pKa values under the schematic in blue, dimensions and polydispersity under pKa in black, and total flux luminescence activity (p/s) in red; (**B**) the images of luciferase expression in the whole body 4–6 h after the i.v. injection of Luc-mRNA co-assembled with IAJDs; (**C**) the images of organs with their activity shown using the color code from the bottom right column.

Excellent agreement for duplicated or triplicated experiments is shown by the error bars of the statistical analysis illustrated in Figure 7. The experiments, the results of which are shown in Figure 7, revealed the most active synthetic vectors for the delivery of mRNA to the spleen regardless of whether they were generated from four-component LNPs or from one-component DNPs. ***MC3 shows slightly higher activity in delivery to the liver and must not be compared with the spleen since MC3 delivers only to the liver***. Figure 8 illustrates the in vivo delivery of Luc-mRNA with the fifteen most active nsSS IAJDs that deliver to the liver. Delivery to the liver is accomplished successfully with four-component LNPs and is currently part of the technology of BioNTech/Pfizer and Moderna COVID-19 vaccines. 

Consequently, to date, we have not invested special effort in delivery experiments to the liver since delivery to the spleen and lymph nodes is more efficient for the design of vaccines. Figure 8 summarizes the most active in vivo mice results accomplished with the nsSS IAJDs from Figure 5B. It is interesting to observe from Figure 8 that the delivery of mRNA to the liver is also accompanied by delivery to lymph nodes. Although only screening experiments were performed in all cases, a total flux of luminescence of 10^8^ p/s was observed for the nsSS IAJD 173, which has a combination of 13 and 16 carbons in its nonsymmetric hydrophobic part. This IAJD has a three-carbon difference between the length of the two alkyl groups. This result is in agreement with the results observed for the activity of the delivery to the spleen. Four nsSS IAJDs, i.e., 161, 171, 177, and 190, display almost 10^8^ p/s total flux luminescence activity. Without any optimization, screening experiments show that all the other nsSS IAJDs, including the SS IAJD 30, display the total flux of their luminescence in the range of 10^7^ p/s. 

**Figure 9:**
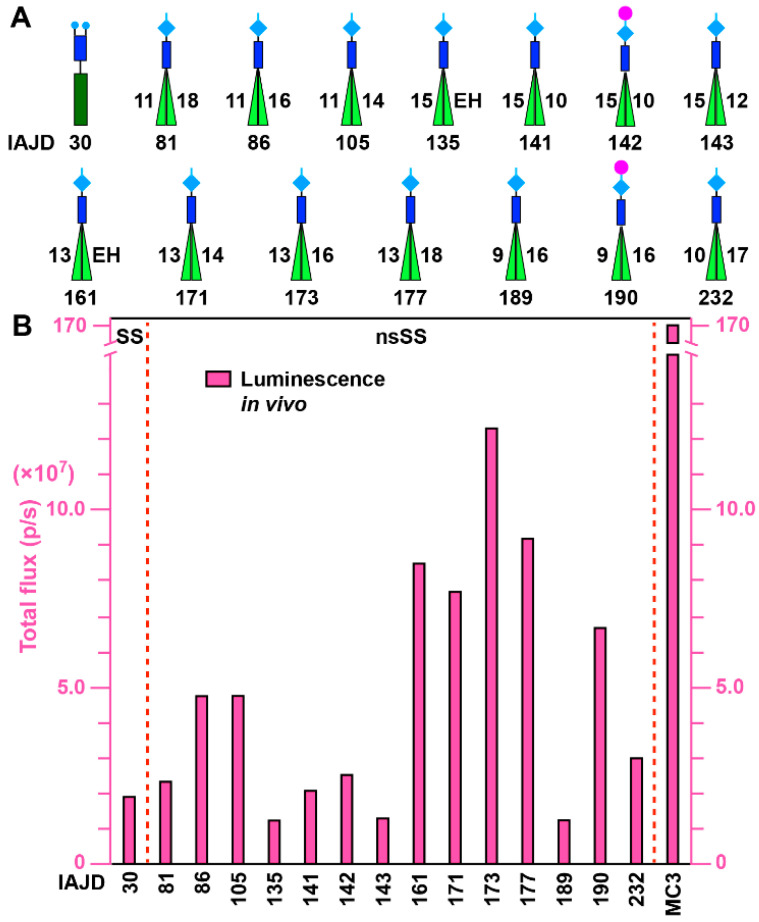
Comparison of the activities of the 15 most active DNPs which deliver mRNA to the liver: (**A**) schematic representations of IAJDs with the number of carbons in the alkyl groups from their hydrophobic part on the left and right sides of the structure, and IAJD numbers in black under the schematic of IAJD; (**B**) plots of total flux luminescence (p/s) for the DNPs assembled from the IAJDs from (**A**).

It is interesting to observe that the two pairs of nsSS IAJDs that show activity for delivery to the lung and contain both hydroxyethyl piperazine and methyl piperazine ionizable amines with the identical pairs of alkyl groups (15 and 10 for 141 and 142; 9 and 16 for 189 and 190) follow the same trend of their total flux. The highest activity is for the IAJD containing hydroxyethyl piperazine. The summary of the total flux as a function of IAJD structure is summarized in Figure 9.

### 3.7. In Vivo Delivery of Luc-mRNA with DNPs to Lung

In order to provide a comprehensive discussion on the concept of screening libraries to discover the molecular design principles of IAJDs and correlate their structure with the targeted activity, in this report, we decided to also summarize the twenty IAJDs that display the most active activity in their delivery to the lung [24].

**Figure 10:**
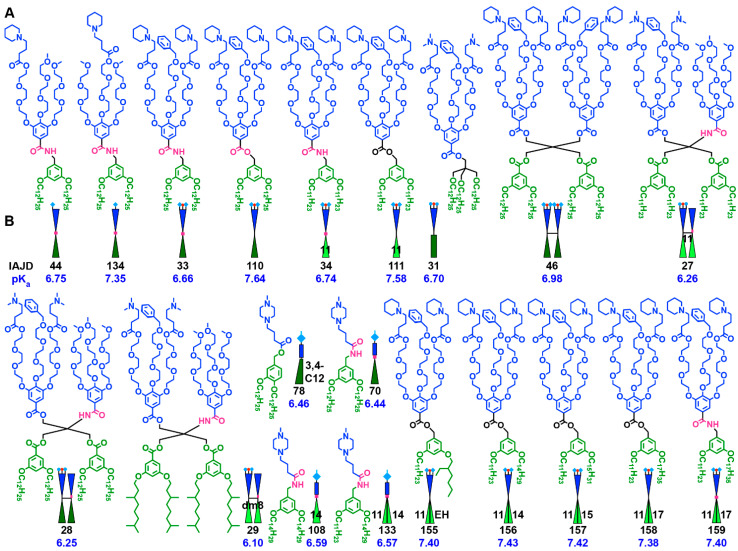
Structures of the 20 most active IAJDs for selective delivery of mRNA to the lung. (**A**) Legend: Schematic representations of IAJDs with the number of carbons in the alkyl groups from their hydrophobic part on the left and right sides of the structure, IAJD numbers in black, under the schematic of IAJD and pKa values in blue under the number of IAJD (**B**).

These data represent a combination of results, reported first time here and in our previous publications [24,25,26]. Figure 10 shows the structures of all these IAJDs. They represent a large diversity of IAJD structures, including SS, TT, TM, sSS, nsSS, and sSS. 

**Figure 11:**
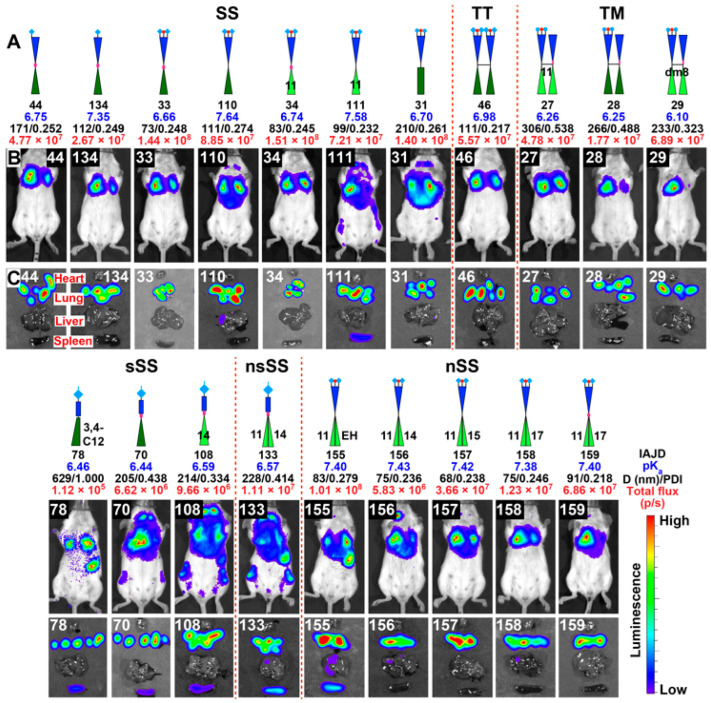
Selective delivery of Luc-mRNA in vivo by 20 most active DNPs to the lung: (**A**) schematic representation of the IAJD structures with the number of the carbons in the hydrophobic part on the left and right sides of IAJD, pKa values under the schematic in blue, dimensions and polydispersity under pKa in black, and total flux luminescence activity (p/s) in red; (**B**) the images of luciferase expression in the whole body 4–6 h after the i.v. injection of Luc-mRNA formulated with IAJDs; (**C**) the images of organs with luciferase activity are shown using color coding from the bottom right column.

**Figure 12:**
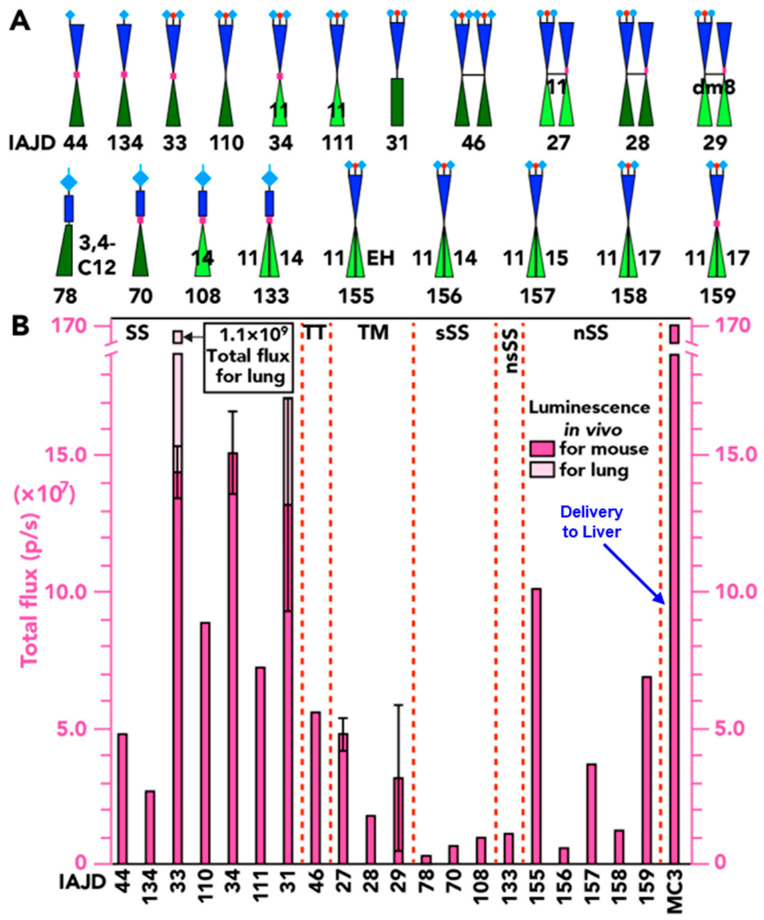
Comparison of the activities of 20 best DNPs that deliver mRNA to the lung: (**A**) schematic representations of IAJDs with the number of carbons in the alkyl groups from their hydrophobic part on the left and right sides of the structure, and IAJD numbers in black under the schematic of IAJD. MC3 delivers mRNA to the liver; (**B**) plots of total flux luminescence (p/s) for the DNPs assembled from the IAJDs from (**A**).

Figure 11 shows the mice images for all in vivo experiments performed with the IAJDs from Figure 10. Figure 12 plots the activity data for the IAJDs from Figure 10 with their in vivo mice experiments from Figure 11. Without discussing the details, we see that the hydrophilic part of IAJDs determines the delivery target, while the hydrophobic part of the IAJDs is responsible for the level of activity.

IAJDs 33, 34, 31, and 155 display activity in the range of 10^8^ p/s, while IAJD 33 shows a total flux for the lung of 10^9^ p/s, which is similar to that of MC3 to the liver. The comparison with MC3 from Figure 12 is not valid since MC3 delivers mRNA only to the liver. IAJDs 110, 111, 29, and 159 show activity almost equal to 10^8^ p/s. We can also compare the SS IAJD 111 containing 11 carbons in its equal alkyl groups of the hydrophobic part and activity of 7.4 × 10^7^ p/s, with nSS IAJD 155 containing a combination of 11 and ethyl hexyl alkyl groups that provide 1.01 × 10^8^ p/s total flux activity. 

These unoptimized results demonstrate that the targeted organ is selected according to the structure and sequence of the ionizable amine in the hydrophilic part of the IAJD, while the overall activity is determined mostly based on the structure, composition, and sequence of the hydrophobic part of the IAJD. 

### 3.8. In Vivo Delivery of Luc-mRNA with sSS DNPs

**Figure 13:**
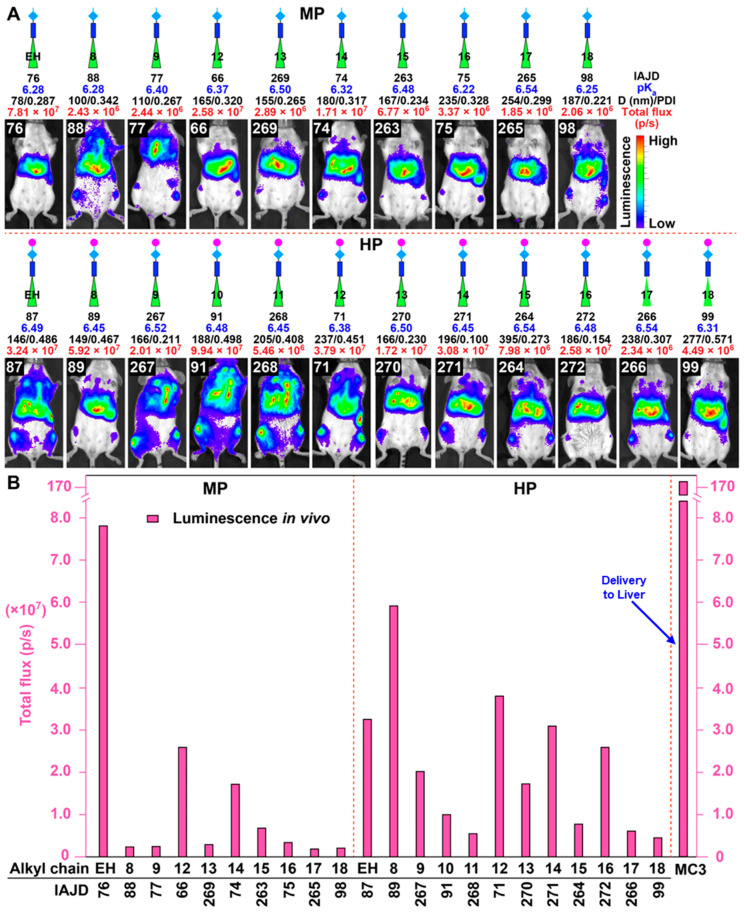
Selective delivery of Luc-mRNA in vivo via all 22 sSS IAJDs-based DNPs: (**A**) the schematic structures of all sSS IAJDs, the number of carbons in their alkyl groups, their numbers, pKa, dimensions and polydispersity of their DNPs and whole-body mice images of delivered Luc-mRNA; (**B**) whole-body luciferase activity (total flux in p/s) as a function of the number of carbons in the alkyl groups of sSS IAJDs derived DNPs.

So far, we discussed experiments in which the delivery of mRNA was performed with nsSS-based DNPs. Figure 13A shows the delivery experiments of mRNA with all the 22 sSS IAJD-forming DNPs containing the number of carbons in their alkyl groups that were employed in the construction of the nsSS IAJDs reported in the previous subsections. All the 22 sSS IAJD-based DNPs deliver to the spleen. It is interesting to observe from Figure 13B that the whole-body total flux of the luciferase activity of all the DNPs assembled from sSS IAJDs is in the range of 10^7^ p/s, and therefore, it is lower than 10^8^ p/s. This demonstrates, as it will be shown more efficiently in three-dimensional figures in the following subsections, that substitution with nonsymmetric alkyl groups in the hydrophobic part of nsSS IAJDs decreases the crystallization and increases the solubility in ethanol of IAJDs with long alkyl groups through comparison with that of sSS IAJDs. At the same time, this concept facilitates a substantial enhancement of the activity of their DNPs. 

### 3.9. A Hypothetic Mechanism Explaining the Activity of mRNA Delivery via the Hydrophobic Part of IAJDs 

A model describing the structure of the bilayer of the vesicles named dendrimersomes assembled via the injection of Janus dendrimers in water and in the buffer as well as the prediction of their dimensions was elaborated by one of our laboratories [41]. This method relies on the idea that dendrimersome vesicles are closed bilayer films with identical bilayer structure and bilayer planarity in a solid state that is only slightly curved in the wall of the dendrimersome assembly [84,85,86].

**Figure 14:**
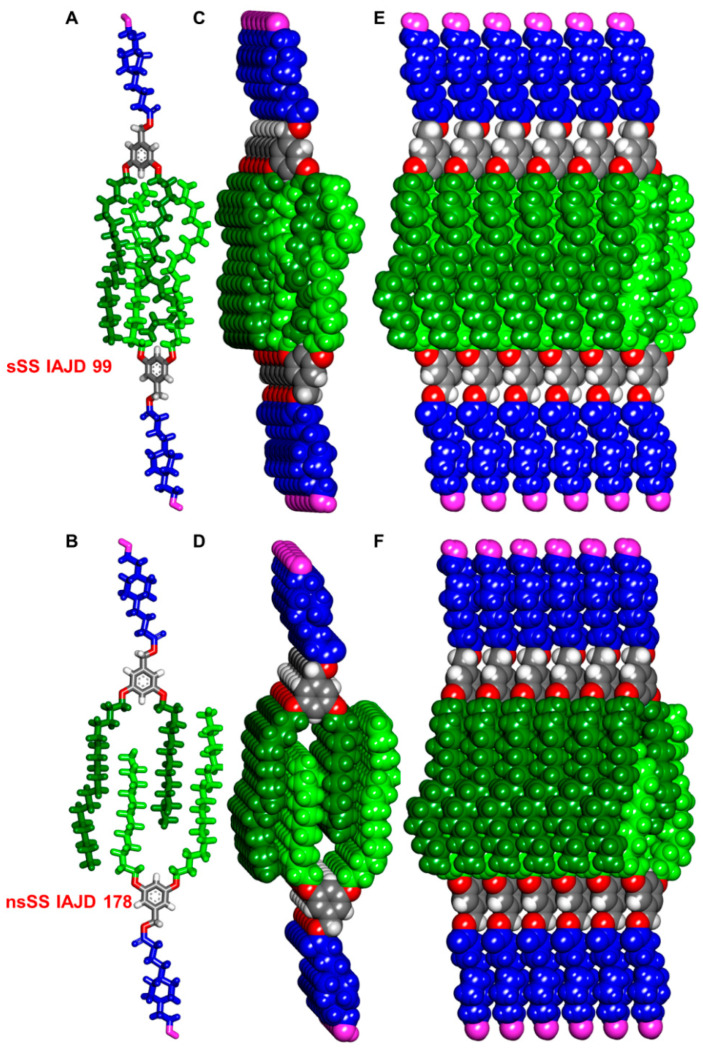
Molecular models of the bilayer structure of sSS IAJD 99 and of nsSS IAJD 178: (**A**,**B**) dimers of sSS IAJD 99 containing 18 carbons in both alkyls and of nsSS IAJD 178 based on 18 and 13 carbons in its alkyls (stick models, side view for both IAJDs); (**C**,**D**) dimers of sSS IAJD 99 and of nsSS IAJD 178 (space-filling models, side view for both IAJDs); (**E**,**F**) bilayers of sSS IAJD 99 and of nsSS IAJD 178 (space-filling models, side views for both IAJDs). Color legends: hydrophilic part in blue; hydrophobic part in light and dark green; oxygen and OH in pink; aromatic ring in gray; H groups on aromatic in white.

It has been demonstrated that amphiphilic Janus dendrimers self-assembled from 3,5-disubstituted phenolic acids interdigitate their aliphatic alkyl groups forming dendrimersome vesicles with mechanical properties and prolonged circulation time as good as those of stealth liposomes [73,75,87] and polymersomes [39]. Figure 14A illustrates the interdigitation of the octadecyl alkyl groups of sSS IAJD 99. A perfect bilayer is self-organized during this self-assembly process. This process generates a very compact bilayer of the dendrimersome nanoparticle that in fact is a vesicle with maximum hydrophobic interactions between its alkyl groups. The transition from sSS IAJD 99 to nsSS IAJD 178 and to other nsSS IAJDs was selected originally to improve the solubility of sSS IAJD 99 containing two octadecyl alkyl groups and of other sSS IAJDs containing long alkyl groups. This increased solubility was expected due to the two different alkyl chain lengths in the hydrophobic part of the new nsSS IAJD 178 that will increase the entropy of the bilayer. The expected increased solubility due to decreased tendency towards the crystallization of the IAJDs was indeed accomplished at the transition from sSS IAJDs to nsSS IAJDs, as it was in the case of sSS IAJD 99 to nsSS IAJD 178. This mechanism of increasing solubility by decreasing the tendency towards crystallization provides an excellent opportunity to design IAJDs with long alkyl groups that do not contain *cis*-double bonds as phospholipids contain and, therefore, are oxidatively stable. However, at the time of these preliminary experiments, we could not predict that an increase in DNP activity for the delivery of mRNA from 4.49 × 10^6^ to 4 × 10^8^ will occur by changing the self-assembly of DNPs from sSS IAJD 99 to nsSS IAJD 178. The model for the bilayer of nsSS IAJD 178 responsible for this two orders of magnitude increase in DNP activity is shown in Figure 14B,D,F. This model demonstrates that an increase in nsSS IAJD 178 solubility was accompanied by a decrease in the perfection of the bilayer packing that decreases the hydrophobic interactions between the alkyl groups in the hydrophobic part of the DNP bilayer self-organized from nsSS IAJD 178. This less perfect DNP does not contain empty space in its bilayer, as shown in the model from Figure 14B,D. This “empty space” is in fact filled up with distorted rather than almost all-*trans* alkyl groups. Nevertheless, the “empty space” from Figure 14 helps to indicate the number of methylenes that have to change their all-*trans* conformation, which most probably facilitates a more efficient endosomal escape of the mRNA in the cytoplasm (Figure 1C) due to lower hydrophobic interaction in the bilayer of nsSS IAJDs based DNPs. These preliminary results will be complemented via a comprehensive investigation of this process, which will be published soon. Regardless of the outcome of these more detailed investigations, the model from Figure 14 indicates one of the key contributions of this report: “*an unexpectedly simple design principle for the control of delivery activity by the molecular engineering of the hydrophobic part of DNPs*”. 

As already mentioned, a quantitative approach to these molecular design principles requires additional research. For example, the mechanism may be completely different for the case of IAJDs derived from 3,4-dihydroxy and 3,4,5-trihydroxy phenolic acids from the case of the similar structure based on 3,5-dihydroxy homolog since the former do not interdigitate in their bilayer structure. Nevertheless, the synthetic capability of one-component IAJD and DNP delivery systems definitively relies on the ability of molecular design principles based on the precise location of its functional groups rather than on the statistical distribution of its four components from the case of four-component LNPs.

### 3.10. The Current Status of the Molecular Design Principles to Target the Delivery of mRNA Mediated via IAJDs

**Figure 15:**
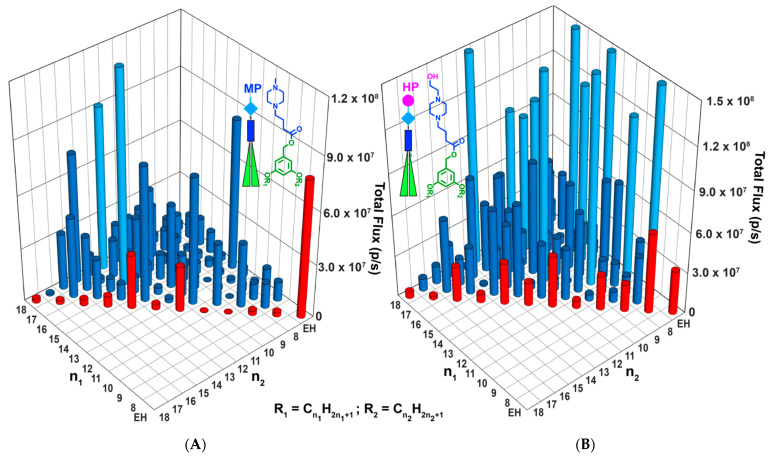
Comparison of the Luc-mRNA delivery activities (whole-body total flux in p/s) of DNPs exhibiting in the hydrophilic part the methyl piperazine group ((**A**) left side) and hydroxyethylpiperazine group ((**B**) right side). The red cylinders represent the activity of the symmetric sSS IAJDs employed in the assembly of DNPs, and the blue cylinders are the nonsymmetric nsSS IAJDs used to assemble in the hydrophobic part. Cylinders in cyan color represent activity higher than 10^8^ (p/s).

A three-dimensional (3D) representation of the transfection activity accomplished in vivo with DNPs co-assembled from sSS IAJDs and nsSS IAGDs with mRNA is illustrated in Figure 15 for the case of delivery to the lung (Figure 15A) and to the spleen and lymph nodes (Figure 15B) as a function of the number of carbons in their alkyl groups. The red cylinders from the diagonals of the two 3D plots contain equal numbers of carbons in the alkyl groups located at the 3- and 5- positions of the sSS IAJDs, while the blue and cyan cylinders show the activity results for different numbers of carbons in the same two positions of the nsSS IAJDs derived DNPs. 

A remarkable conclusion results from these 3D plots. The red cylinders that correspond to sDD IAJD-derived DNPs exhibit the lowest activity, while the blue cylinders that correspond to nsSS IAJD-based DNPs display the highest activity. The cyan cylinders exhibit activities that are higher than 10^8^ p/s. This structure of IAJD activity of DNP dependence demonstrates the ability to discover molecular design principles for the targeted delivery of mRNA via the rapid screening of DNPs co-assembled through the simple injection of IAJDs with different primary structures with mRNA and to predict their activity. No dialysis and fractionation of the DNP assemblies, as needed in the case of LNPs, is required for these rapid screening experiments.

## 4. The Early Days of Nucleic Acid Delivery and Their Evolution to the Current Methodologies

**Figure 16:**
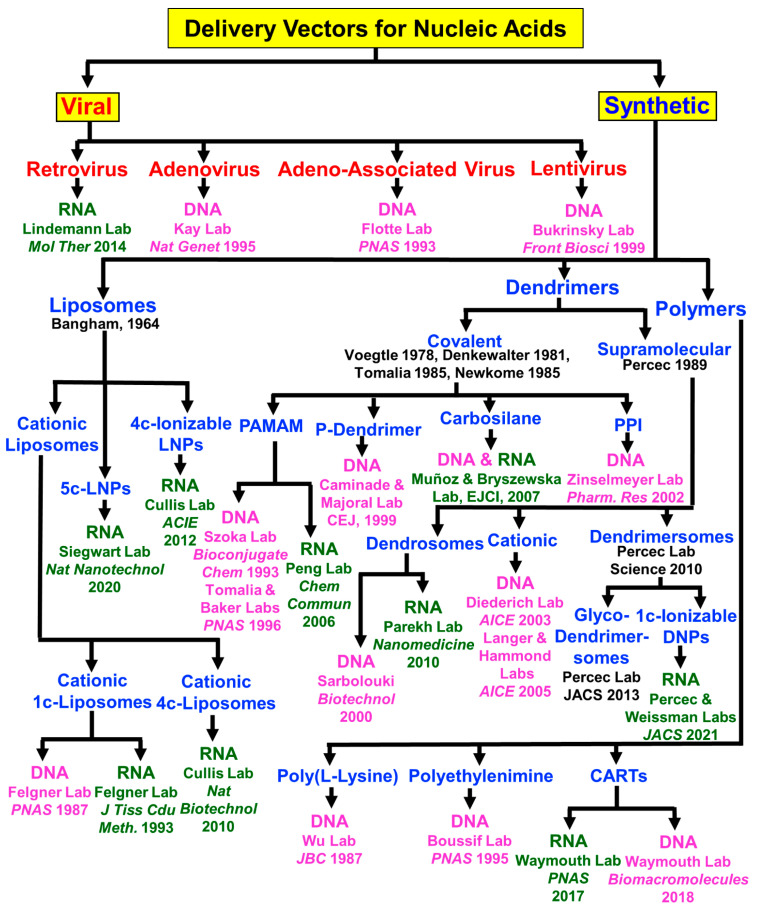
Summary of the viral and nonviral vectors for the delivery of nucleic acids and the evolution of methodology development [2,12,24,29,39,52,88,90,91,95,96,97,98,99,100,101,102,103,104,105,106,107,108,109,110,111,112,113,114,115,116,117].

According to our knowledge, Figure 16 organizes for the first time the historical development of the viral and synthetic vectors for the delivery of DNA and RNA starting from the early days to the currently employed methodologies. This is not a comprehensive review, and therefore, we apologize to the authors that may have discovered methodologies that we may have missed incorporating in Figure 16. However, the goal of this figure is to illustrate, in a very brief fashion, the conceptual development of this field and the rationale for the direction of its evolution. As shown in the title of Figure 16, delivery vectors for nucleic acids can be classified in the simplest way as viral and nonviral or synthetic. Some of the viruses employed in nucleic acid delivery are the retrovirus which was first used for the delivery of RNA in 2014 [88]. Adenovirus [89], adeno-associated virus [90], and lentivirus [91] were some of the most frequently used viruses for the delivery of DNA that started as early as 1995, 1993, and 1999, respectively. Viral vectors are currently used in commercial vaccines [92,93,94,95,96]. Viral vectors are very efficient, but synthetic vectors rely on their unlimited synthetic capabilities. 

The discussion on synthetic vectors will rely on demonstrating their synthetic capabilities. The simplest classification of synthetic vectors relies on liposomes, covalent and supramolecular dendrimers, and polymers. The early days of nucleic acid delivery with liposomes employed cationic one-component liposomes. Cationic one-component liposomes were pioneered by the laboratory of Felgner for the delivery of DNA in 1987 [95] and by the same laboratory for the delivery of RNA in 1993 [96]. Cationic liposomes are toxic and bind both DNA and RNA via electrostatic interactions on their exterior surface and in their interior. The nucleic acid from their exterior surface undergoes enzymatic degradation, and therefore, an ideal vector would contain the nucleic acid only in its interior where it is protected against enzymatic degradation. Toxicity and degradation were the limiting features that did not facilitate, according to our knowledge, the commercial development of cationic one-component liposomes in vaccines, although the cationic lipid component is commercially available under the name Lipofectamine transfection reagent and Transfectamine 5000 transfection reagent. 

In 2010, Cullis laboratory developed a four-component cationic liposome for the delivery of RNA [97] that is affected by the same features as the one-component cationic liposomes. The solution to the one-component and four-component cationic liposomes was solved in an elegant way also by Cullis laboratory in 2012 [29]. This laboratory elaborated four-component ionizable lipid nanoparticles (LNPs) by expanding the three-component stealth liposomes into four-component lipid nanoparticles. LNPs rely on a combination of phospholipids, cholesterol, ionizable lipids, and PEG-conjugated lipids. The elegance of this concept consists of the presence of an ionizable lipid that, at a certain pH, becomes protonated and, therefore, binds RNA, while at the physiological pH, it becomes deprotonated and, therefore, releases the RNA from the periphery of LNPs but not from its interior and eliminates cationic-derived toxicity. Therefore, LNPs are nontoxic. 

However, the ionizable lipid and the PEG-conjugated lipid have their own deficiencies in a four-component LNP, which were briefly mentioned in the introductory part of this paper and in our previous publications [24,25,26]. Details of these developments and the deficiencies resulting from the statistical distribution of the four components of the LNPs will be discussed in the next subsection. LNPs are currently the most successful RNA delivery vector used in commercial mRNA-based vaccines, although they deliver mostly to the liver. A five-component LNP system was recently developed by Siegwart Laboratory in 2020 [12]. The fifth component of this LNP has either permanently positive or negative charges that can facilitate delivery to organs other than the liver. Cationic covalent dendrimers made their entry into the delivery of nucleic acids in 1993 [98] and 1996 [99], when Szoka and Tomalia, Baker laboratories, reported the delivery of DNA with the cationic covalent PAMAM dendrimers. Both laboratories investigated different generations and observed a strong dependence on the delivery in vitro as a function of generation number. In 2006, Peng Laboratory also employed cationic PAMAM dendrimers to deliver RNA [100]. In 1999, Caminade and Majoral Lab used phosphorus (P)-containing dendrimers to deliver DNA [101]. Cationic carbosilanes were employed to deliver DNA and RNA by Muñoz and Bryszewska laboratories in 2005 [102,118,119,120,121,122], while cationic PPI was used by Sinselmeyer Lab in 2002 [103,123]. Both covalent cationic dendrimers are associated with some of the negative features of cationic liposomes. 

A solution to this problem was generated by Sarbolouki’s laboratory in 2000 [104]. His laboratory encapsulated the complex of cationic dendrimers with DNA into liposomes named dendrosomes. In 2010, Parekh Lab employed dendrosomes for the delivery of RNA [105]. Dendrosomes eliminate the toxicity and enzymatic degradation of cationic dendrimers but require extensive additional processes for encapsulation in liposomes. 

Cationic supramolecular dendrimers obtained via the self-assembly of cationic amphiphilic dendrimers were employed by the laboratory of Diederich starting in 2003 [106] and Langer and Hammond in 2005 [107] for the delivery of DNA. Supramolecular cationic dendrimers are also toxic. Therefore, the Langer and Anderson laboratories elaborated on two- to four-component LNPs in which the ionizable lipid was replaced with amphiphilic ionizable dendrimers based on PAMAM or polyethylene imine (PEI) [124,125]. 

The most recent progress in this field was in 2021, when Percec and Weissman laboratories [24,25,26] developed one-component multifunctional sequence-defined ionizable amphiphilic Janus dendrimers (IAJDs) that co-assemble with mRNA into dendrimersome nanoparticles (DNPs). This co-assembly process proceeds by the simple injection of the IAJD into the acidic buffer solution of mRNA. The IAJD concept was developed, as already mentioned in the early part of this paper, based on inspiration from research on amphiphilic Janus dendrimers (JDs) that self-assemble dendrimersomes (DS) [39,40,41,42,43,44,45,46,47,48,49,50,51] and sequence-defined amphiphilic Janus glycodendrimers (JGD) that self-assemble glycodendrimersomes (GDSs) [52,53,54,55,56,57,58,59,60,61,62,63,64,65,66,67,68,69,70]. 

As discovered in this report, the structure of the hydrophilic part of IAJD determines the organ targeted, while the structure of the IAJD in its hydrophobic part determines the activity of the in vivo transfection. With these synthetic vector technologies, delivery to the spleen and lymph nodes, liver and lymph nodes, and lung became accessible. One of the most difficult challenges in this field remains the delivery to the nervous system, including the brain, which is under extensive investigation in the laboratory of Cena [126,127,128,129,130,131,132,133,134,135,136,137,138,139,140,141].

**Figure 17:**
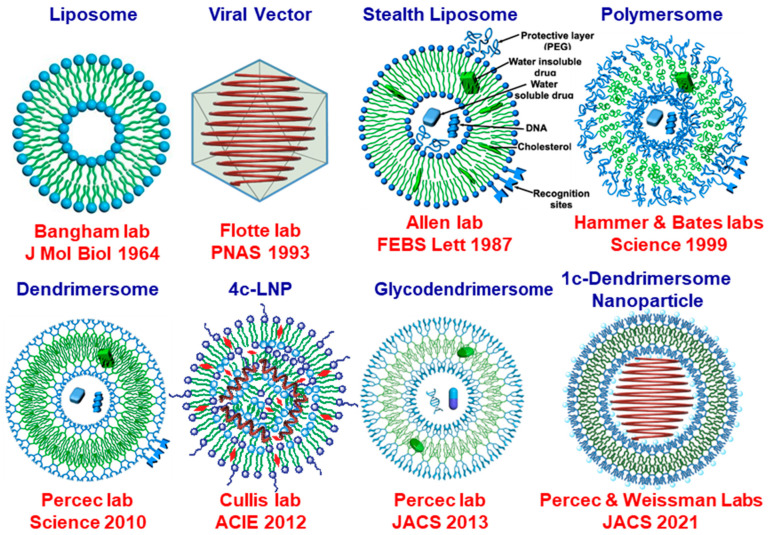
A brief summary of the evolution, development, and discovery of ionizable LNPs and DNPs [24,25,29,39,52,73,90,112,142].

The final group of synthetic vectors that have been developed are based on synthetic polymers. Cationic poly(L-lysine) and polyethyleneimine were, respectively, both employed for the delivery of DNA in 1987 by Wu’s laboratory [108] and in 1995 by Boussif’s laboratory [109]. We must recall that polyethyleneimine is a very high-molecular-weight branched polymer obtained via the cationic ring-opening polymerization of ethylene imine. A very elegant strategy was recently developed by Waymouth’s laboratory, where Charge–Altering Releasable Transporters (CARTs) were employed for the delivery of RNA in 2017 and for DNA in 2018 [110,111]. A brief illustration of the most important historical developments ranging from liposomes, viral vectors, stealth liposomes, polymersomes, dendrimersomes, four-component LNPs, glycodendrimersomes, and 1c-DNPs is schematically illustrated in Figure 17 [90,112].

The rationale of Figure 17 is first to illustrate their three-dimensional structures and their structures containing the encapsulated mRNA. Briefly, we can observe that, in the absence of mRNA liposomes, stealth liposomes, polymersomes, dendrimersomes, 4c-LNPs, glycodendrimersomes, and 1c-DNPs are all vesicles. Liposomes are unstable and require cholesterol and PEG-conjugated lipids to increase their stability and circulation time. Polymersomes, 4c-LNPs, glycodendrimersomes, and 4c-DNPs are as stable, or even more stable than, stealth liposomes. Regardless of the method of preparation, liposomes, stealth liposomes, polymersomes, and 4c-LNPs are polydisperse and must be fractionated in order to obtain the desired dimension and polydispersity. Viral vectors are monodispersed, and dendrimersomes, glycodendrimersomes, and 1c-LNPs are obtained with narrow polydispersity and predictable dimensions through simple injection in water or buffer. Therefore, viral vectors and 1c-DNPs are more closely related in both the absence and presence of mRNA. Notably, 4c-LNPs require dialysis after the encapsulation of mRNA. During this process, the neutral ionizable lipids get encapsulated together with the mRNA inside of their external vesicle, generating a complex internal mixture containing ionizable lipids, mRNA, and cholesterol [28,142,143,144,145,146,147]. Therefore, an ideal synthetic nonviral vector would have to be closely related to the structure of viral vectors in order to provide the very high activity of the viral vectors and the flexibility of the synthetic vectors. Although substantial research is required to accomplish a system like this, we believe that the strategy of 1c-IAJD-DNP is conceptually suitable to accomplish it. Alternative dendrimer fragments such as carbosilane, PPI, and others can also be incorporated into IAJDs [101,102,118,119,120,121,122].

## 5. Inspiration from and Collaboration with Donald A. Tomalia

The history of the discovery of covalent dendrimers was published repeatedly, and it is well known to all contributors and readers of this Special Issue dedicated to the 85th anniversary of Donald A. Tomalia. However, in spite of being well known, we will repeat it briefly here. Dendrimers were discovered independently in four different laboratories. In 1978, Fritz Voegtle from the University of Bonn [113] reported the bis-cyanoethylation of primary aliphatic amines and of bifunctional secondary amines, followed by the reduction of the cyano groups and the subsequent iteration of these two steps to synthesize two generations of “**cascade**” molecules. In a series of US patents starting in 1981, Denkewalter, Kolc, and Lukasavage of Allied Corporation [114] produced branching monodisperse spherical poly(lysine)s that were found by Aharoni from the same company to be indeed globular and monodisperse [148]. In 1985, Tomalia, Baker, Dewald, Hall, Kallos, Martin, Roeck, Ryder, and Smith from Dow Chemical Company reported the synthesis and characterization of a new class of polymers named **starburst–dendritic macromolecules** [115]. Seven generations of starburst–dendritic macromolecules were synthesized via the iterative Michael addition of methyl acrylate to ethylenediamine or ammonia, followed by extensive amidation with excess ethylene diamine and reiteration of this methodology. Additionally, in 1985, Newkome, Yao, Baker, and Gupta reported a new “**cascade**” molecule that provided a synthetic approach to unimolecular micelles. This molecule was named **arborol** [116]. These laboratories reported excellent synthesis methods, identified side reactions to structural imperfections in the designed compound, and employed careful analysis of the final products. 

However, neither “**cascades**” nor “**arborols**” but rather “**dendrimers**” provided the name for the new field of perfectly branched and monodisperse synthetic macromolecules. In fact, **The First International Dendrimer Symposium** (**IDS-1**), a conference that became biannual, was organized by Professor Fritz Voegtle, the person who invented “**cascade**” molecules, between 3 and 5 October 1999, in Frankfurt, Germany [149]. 

Many review articles and books on covalent dendrimers were published by Tomalia, Voegtle, and Newkome laboratories and by many other groups that joined the field soon after [7,8,149,150,151,152,153,154,155,156,157,158,159,160,161,162,163,164,165,166]. 

In an attempt to synthesize a biaxial nematic thermotropic liquid crystal, in the late 1980s, Percec’s laboratory discovered self-assembling dendrons, self-assembling dendrimers, and self-organizable dendronized polymers [167]. Percec’s laboratory, in collaboration with top experts in the field, developed and employed a combination of structural analysis through X-ray diffraction, transmission electron microscopy, electron density maps, and reconstruction of the X-ray diffractograms using the method elaborated by Watson and Crick to solve the structure of DNA, to elucidate the three-dimensional (3D) structure of supramolecular dendrimers [167,168,169,170,171,172,173,174]. This combination of diffraction methodologies added a new dimension to the characterization of dendrimers and created the complementary subfield of supramolecular dendrimers. Helical chirality resembling the mechanism used by rod-like or columnar and polygonal including icosahedral viruses [175,176,177,178], Frank–Kasper phases [175,176,178,179,180,181,182,183,184,185,186,187,188,189,190,191,192,193,194,195,196,197,198,199,200,201,202,203], liquid quasicrystals [173,204,205,206,207,208,209], amphiphilic Janus dendrimers [39,40,41,42,43,44,45,46,47,48,49,50,51], Janus glycodendrimers [52,53,54,55,56,57,58,59,60,61,62,63,64,65,66,67,68,69,70] and one-component sequence-defined ionizable amphiphilic Janus dendrimers (IAJDs) [24,25,26] are just a few of the new concepts via which supramolecular dendrimers impacted a diversity of disciplines of soft and living matter. 

**Figure 18:**
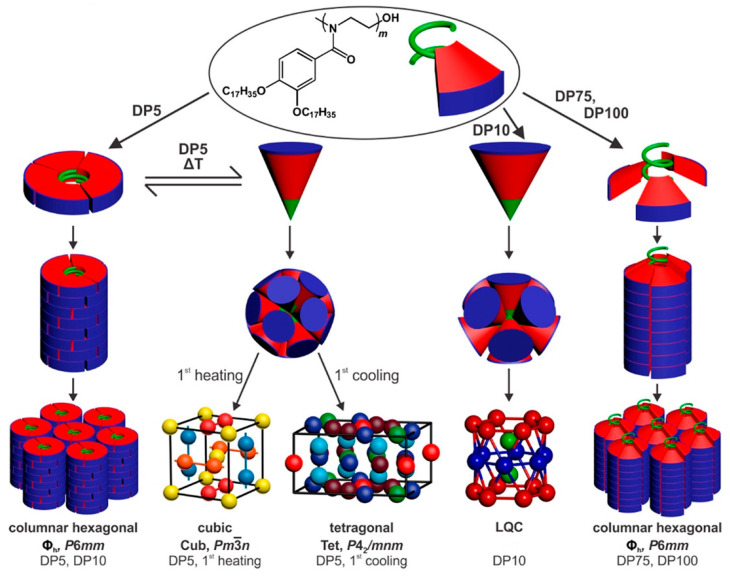
Summary of periodic and quasiperiodic arrays self-organized from assemblies of poly[(3,4)17G1-Oxz] at different degrees of polymerization (DP) and temperature. This Figure is reproduced with permission from reference [205]. Copyright © 2018, American Chemical Society.

Tomalia’s leading role, characterized by enthusiasm, creativity, and modesty, inspired, encouraged, and attracted new contributors to the field of dendrimers. He supported us when we discovered that supramolecular dendrimers can form conventional thermotropic liquid crystal phases [210,211,212], rod-like or columnar shape assemblies [117,213,214,215,216,217,218,219,220,221,222,223,224,225,226,227,228,229,230,231,232,233,234,235,236,237,238,239,240,241,242,243,244,245,246,247,248,249,250], and ultimately, spherical and even chiral spherical or spherical helical shapes generating Frank–Kasper [175,176,178,179,180,181,182,183,184,185,186,187,188,189,190,191,192,193,194,195,196,197,198,199,200,201,202] and quasicrystal [173,203,204,205,206,207,208,209] phases. He also inspired and encouraged us to generate the first nanoperiodic tables of supramolecular dendrimers [198] and to continue the pathway to the discovery of new concepts in supramolecular dendrimers by solving their 3D structure, elucidating the mechanism, and ultimately predicting additional primary structures behaving in a similar way. Don also inspired Percec’s laboratory to select the name dendrimersome out of several other options when we discovered the self-assembly of amphiphilic Janus dendrimers into monodisperse vesicles with predictable dimensions [39]. While the readers of this Special Issue are familiar with the contributions of Tomalia to dendrimers, not many of them may know that in 1966, the laboratories of Tomalia, Kagia, Seeliger, and Litt, independently from each other, discovered the cationic ring-opening polymerization of 2-substituted-2-ozazolines and their living polymerization [205,251,252,253,254,255,256,257]. Self-organizable dendronized polymers obtained through the living cationic ring-opening polymerization of dendronized 2-oxazolines allowed Percec’s laboratory to establish some of the most fundamental dependences of primary structure–tertiary structure [255,256,257], including the discovery of liquid quasicrystal, A15, and σ Frank–Kasper phases within only five monomer repeat units of the degree of polymerization of the dendronized polymer [205] (Figure 18).

A series of self-accelerated [187,189] and self-interrupted processes [190] during the synthesis of supramolecular dendrimers, some of them inspired by Tomalia’s work [149], culminated in the first methodology for the synthesis of monodisperse macromolecules via the self-interrupted living ring-opening polymerization of a dendronized monomer [190]. We would like to state that our laboratory never wrote a joint paper with Tomalia. However, one of the corresponding authors (V.P.) has never been turned down by Don when he was invited as a plenary or invited speaker at the numerous conferences organized by V.P. 

**Figure 19:**
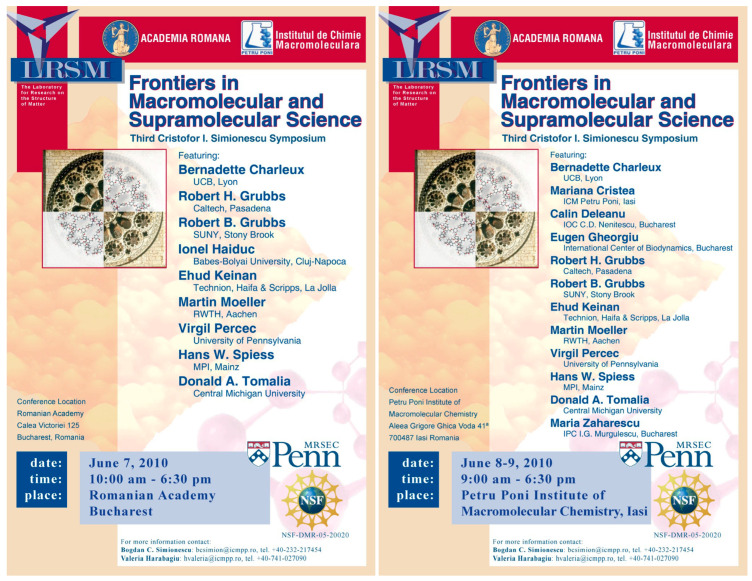
The posters with the names of the invited speakers including Donald A. Tomalia from the 2010 Frontiers in Macromolecular and Supramolecular Science Symposia co-organized by one of us (V.P.) at the Romanian Academy in Bucharest and in Iasi.

**Figure 20:**
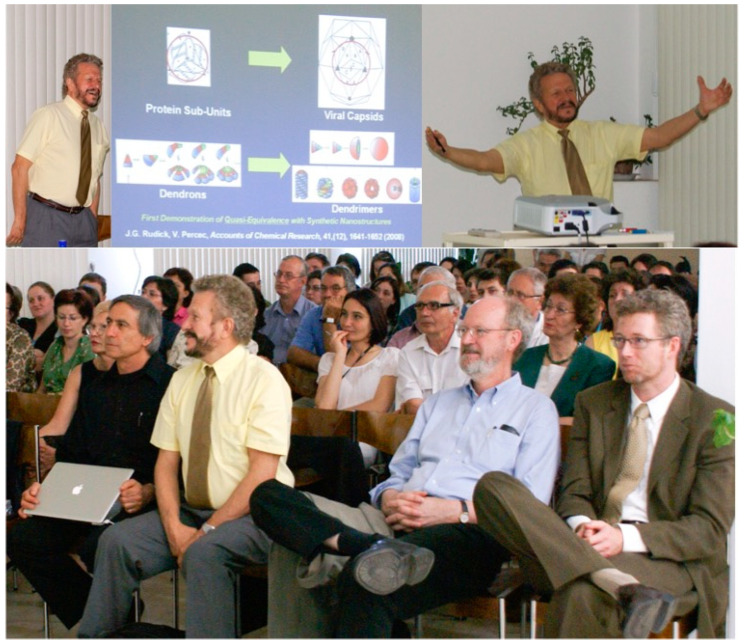
Top part shows the enthusiastic Donald Tomalia giving his lecture at the Romanian Academy in Iasi in 2010. Bottom part, first row from right to left: Robert (Barney) Grubbs, the Nobel Laureate Robert (Bob) Grubbs, Donald Tomalia, and Ehud Keinan, the President of the Israel Chemical Society.

The posters shown in Figure 19 present the list of the invited speakers from the third Frontiers in Macromolecular and Supramolecular Science Symposium dedicated to the memory of V.P.’s PhD mentor that took place at the Romanian Academy between 7 and 9 June 2010 in Bucharest and Iasi. Donald Tomalia is an illuminating and enthusiastic lecturer and a contagiously positive example for the young and even for senior generations of scientists (Figure 20). 

## 6. Conclusions

Ninety-eight one-component IAJDs based on, mostly plant 3,5-dihydroxy phenolic acid, with different primary structures in both their hydrophilic and hydrophobic parts were synthesized via an accelerated modular–orthogonal methodology. These very simple first-generation amphiphilic Janus dendrimers are constructed from minidendrons, as defined by our laboratory [50,167]. Therefore, they can be named also Janus minidendrimers containing one or two branching points in the hydrophobic part and one or two in the hydrophilic ionizable amine part. In spite of their synthetic simplicity, they are extremely efficient in the construction of IAJDs [24,25,26]. Their co-assembly with Luc-mRNA was accomplished via injection in an acidic buffer to generate DNPs of well-defined dimensions and narrow polydispersity. This methodology provided rapid access to in vivo transfection experiments in mice, without the need for fractionation or dialysis, and demonstrated that all the IAJDs provided DNPs active in transfection experiments in vivo. This methodology also allowed us to determine that their activity could be grouped in terms of delivery to the spleen and lymph nodes, liver and lymph nodes, and lung. 

A primary structure–activity analysis generated by screening libraries allowed the discovery of molecular design principles to target the delivery of mRNA and demonstrate, for this class of IAJDs, that their hydrophilic part determines, through the structure of their ionizable amine and their sequence the targeted organ, with hydroxyethyl piperazine favoring the spleen and methyl piperazine favoring the liver. Piperidine and dimethyl amine favor delivery to the lung. It seems that sterically hindered cyclic ionizable amines display high organ selectivity. The primary structure of the hydrophilic part was shown to determine their activity. A change from sSS IAJDs to nsSS IAJDs demonstrated a mechanism to increase the delivery of mRNA activity by up to two orders of magnitude. 

A mechanism explaining the increase in activity via the hydrophobic part was provided. This report generated the first approach to molecular design principles required for mRNA delivery to targeted organs with one-component IAJDs derived from plant phenolic acids and supports the preliminary data reported in previous communications [25,26]. Most experiments reported here were performed with the symmetric 3,5-dihydroxy plant phenolic acid regardless of the substitution pattern in the hydrophobic part. The corresponding 3,4-dihydroxy plant phenolic acids provide two constitutional isomers under the same conditions, thus doubling the discovery capability. The 3,4,5-trihydroxy plant phenolic acid provides four-constitutional isomers, thus quadrupling the power of discovery. 

However, in addition to other natural phenolic acids, the 3 phenolic acids reported in Figure 2A were already employed to design and synthesize 30 additional constitutional isomeric symmetric and nonsymmetric AB_2_ to AB_9_ phenolic acids. All of them can be employed in these IAJD experiments using molecular design principles [83]. Aside from providing access to elucidate some of the most complex systems of fundamental and applied interest for today’s society [167,173,174,258,259,260], we expect that the discovery of the molecular design principle methodology reported here will bestow rapid access to a large diversity of mRNA vaccines and other nanotherapeutics that will require much simpler preparation, handling, and storage at a reduced price, and lower concentration of mRNA while employing renewable plant starting materials. 

These results also represent a first step towards the generation of a nanoperiodic table of IAJDs for the delivery of mRNA, similar to those of proteins [261,262,263], self-assembling dendrons, and dendrimers [198,264,265], that may be able to help expand the fields of genetic medicine and nanomedicine [34,266,267,268,269]. 

We expect that the development of new architectural concepts, including polygonal supramolecular dendrimers and new synthetic methodologies [270,271,272,273,274,275,276,277,278,279,280,281,282,283,284,285,286,287,288,289,290,291], as well as the attraction of new creative minds to this field, through a methodology similar to that pioneered by the very enthusiastic Donald A. Tomalia, will help impact this field and take it to a new level of discovery and even prediction that, together with practical applications, will reward education and society. All co-authors of this paper thank the Guest Editors for their kind invitation to contribute, with this special combination of research articles and brief review-perspective to the 85th anniversary of Donald A. Tomalia, one of the most influential inventors of this field.

## Data Availability

Data are contained within the article and Supplementary Materials. Additional data will from the lead contact be available upon request.

## Supplementary Materials

The following supporting information can be downloaded at: https://www.mdpi.com/article/10.3390/pharmaceutics15061572/s1, Scheme S1: Synthesis of Nonane-Based IAJDs with 3,5-Nonsymmetric Alkyl Chains; Scheme S2: Synthesis of Dodecane-Based IAJDs with 3,5-Nonsymmetric Alkyl Chains; Scheme S3: Synthesis of Tetradecane-Based IAJDs with 3,5-Nonsymmetric Alkyl Chains; Scheme S4: Synthesis of Hexadecane-Based IAJDs with 3,5-Nonsymmetric Alkyl Chains; Scheme S5: Synthesis of Heptadecane-Based IAJDs with 3,5-Nonsymmetric Alkyl Chains; Scheme S6: Synthesis of Octadecane-Based IAJDs with 3,5-Nonsymmetric Alkyl Chains; Scheme S7: Synthesis of Undecane and Pentadecane Based IAJDs with 3,5-Nonsymmetric Alkyl Chains; Scheme S8: Synthesis of Tridecane Based IAJDs with 3,5-Nonsymmetric Alkyl Chains; Scheme S9: Synthesis of IAJD131; Scheme S10: Synthesis of Decane-Based IAJDs with 3,5-Nonsymmetric Alkyl Chains; Scheme S11: Synthesis of IAJDs with 3,5-symmetric Alkyl Chains; Figure S1: ^1^H NMR spectrum (top), MALDI-TOF MS spectrum (bottom left) and HPLC trace (bottom right) of Monoprotected Benzyl Ether of Methyl 3,5-Dihydroxybenzoate **2**; Figure S2: ^1^H NMR spectrum (top), MALDI-TOF MS spectrum (bottom left) and HPLC trace (bottom right) of Methyl 3-(benzyloxy)-5-(octadecyloxy)benzoate, **33**; Figure S3: ^1^H NMR spectrum (top), MALDI-TOF MS spectrum (bottom left) and HPLC trace (bottom right) of Methyl 3-hydroxy-5-(octadecyloxy)benzoate, **34**; Figure S4: ^1^H NMR spectra (top), MALDI-TOF MS spectra (bottom left) and HPLC trace (bottom right) of Methyl 3-(heptadecyloxy)-5-(octadecyloxy)benzoate, **35d**; Figure S5: ^1^H NMR spectrum (top), MALDI-TOF MS spectrum (bottom left) and HPLC trace (bottom right) of (3-(Heptadecyloxy)-5-(octadecyloxy)phenyl)methanol, **36d**; Figure S6: ^1^H NMR spectrum (top), MALDI-TOF MS spectrum (bottom left) and HPLC trace (bottom right) of 3-(Heptadecyloxy)-5-(octadecyloxy)benzyl 4-bromobutanoate, **37d**; Figure S7: ^1^H NMR spectrum (top), MALDI-TOF MS spectrum (bottom left) and HPLC trace (bottom right) of 3-(heptadecyloxy)-5-(octadecyloxy)benzyl 4-(4-methylpiperazin-1-yl)butanoate, **38g**, IAJD242; Figure S8: ^1^H NMR spectrum (top), MALDI-TOF MS spectrum (bottom left) and HPLC trace (bottom right) of 3-(Heptadecyloxy)-5-(octadecyloxy)benzyl 4-(4-(2-hydroxyethyl)piperazin-1-yl)butanoate, **38h**, IAJD243; Figure S9: ^1^H NMR spectrum (top), MALDI-TOF MS spectrum (bottom left) and HPLC trace (bottom right) of methyl 3,5-bis(heptadecyloxy)benzoate), **64d**; Figure S10: ^1^H NMR spectrum (top), MALDI-TOF MS spectrum (bottom left) and HPLC trace (bottom right) of (3,5-bis(heptadecyloxy)phenyl)methanol, **65d**; Figure S11: ^1^H NMR spectrum (top), MALDI-TOF MS spectrum (bottom left) and HPLC trace (bottom right) of 3,5-bis(heptadecyloxy)benzyl 4-bromobutanoate, **66d**; Figure S12: ^1^H NMR spectrum (top), MALDI-TOF MS spectrum (bottom left) and HPLC trace (bottom right) of 3,5-bis(pentadecyloxy)benzyl 4-(4-methylpiperazin-1-yl)butanoate, **67e**, IAJD265; Figure S13: ^1^H NMR spectrum (top), MALDI-TOF MS spectrum (bottom left) and HPLC trace (bottom right) of 3,5-Bis(pentadecyloxy)benzyl 4-(4-(2-hydroxyethyl)piperazin-1-yl)butanoate, **67f**, IAJD266; Figure S14: DLS data of DNPs assembled from IAJDs 112, 123, 129, 131, 137, 139 and 140; Figure S15: DLS data of DNPs assembled from IAJDs 163, 164, 166–170, 174, 175; Figure S16: DLS data of DNPs assembled from IAJDs 176, 179–185; Figure S17: DLS data of DNPs assembled from IAJDs 186–194; Figure S18: DLS data of DNPs assembled from IAJDs 197–205; Figure S19: DLS data of DNPs assembled from IAJDs 206–214; Figure S20: DLS data of DNPs assembled from IAJDs 224–232; Figure S21: DLS data of DNPs assembled from IAJDs 233–241; Figure S22: DLS data of DNPs assembled from IAJDs 242–247, 263–265; Figure S23: DLS data of DNPs assembled from IAJDs 266–272; Figure S24: Titration curves showing changes in solution pH in response to addition of a strong acid and calculated pKa for IAJDs 73, 90, 112, 121, 123, 129, 131, 137; Figure S25: Titration curves showing changes in solution pH in response to addition of a strong acid and calculated pKa for IAJDs 139, 140, 163–168; Figure S26: Titration curves showing changes in solution pH in response to addition of a strong acid and calculated pKa for IAJDs 169, 170, 174–176, 179–181; Figure S27: Titration curves showing changes in solution pH in response to addition of a strong acid and calculated pKa for IAJDs 182–189; Figure S28: Titration curves showing changes in solution pH in response to addition of a strong acid and calculated pKa for IAJDs 190–194, 197–199; Figure S29: Titration curves showing changes in solution pH in response to addition of a strong acid and calculated pKa for IAJDs 200–207; Figure S30: Titration curves showing changes in solution pH in response to addition of a strong acid and calculated pKa for IAJDs 208–215; Figure S31: Titration curves showing changes in solution pH in response to addition of a strong acid and calculated pKa for IAJDs 261–223; Figure S32: Titration curves showing changes in solution pH in response to addition of a strong acid and calculated pKa for IAJDs 224–231; Figure S33: Titration curves showing changes in solution pH in response to addition of a strong acid and calculated pKa for IAJDs 232–239; Figure S34: Titration curves showing changes in solution pH in response to addition of a strong acid and calculated pKa for IAJDs 240–247; Figure S35: Titration curves showing changes in solution pH in response to addition of a strong acid and calculated pKa for IAJDs 263–270; Figure S36: Titration curves showing changes in solution pH in response to addition of a strong acid and calculated pKa for IAJDs 271, 272. References [24,25,26,35,36,38] are cited in the supplementary materials.
