# Supplementary material for: Screening Libraries to Discover Molecular Design Principles for the Targeted Delivery of mRNA with One-Component Ionizable Amphiphilic Janus Dendrimers Derived from Plant Phenolic Acids
Source: Pharmaceutics. 2023 May 23;15(6):1572. doi: 10.3390/pharmaceutics15061572 (PMC10303357; doi:10.3390/pharmaceutics15061572)
Supplement: Supplementary file 1 [file pharmaceutics-15-01572-s001.zip › pharmaceutics-2390918-supplementary.pdf]

Supporting Information for

Screening Libraries to Discover Molecular Design Principles for the Targeted Delivery of mRNA with One-Component Ionizable Amphiphilic Janus Dendrimers Derived from Plant Phenolic Acids †

Juncheng Lu[1,2,‡], Elena N. Atochina-Vasserman[2,‡], Devendra S. Maurya[1,‡], Muhammad Irhash Shalihin[1], Dapeng Zhang[1], Srijay S. Chenna[1], Jasper Adamson[1], Matthew Liu[1], Habib Ur Rehman Shah[1], Honey Shah[1], Qi Xiao[1], Bryn Queeley[2], Nathan A. Ona[2], Erin K. Reagan[2], Houping Ni[2], Dipankar Sahoo[1,2], Mihai Peterca[1], Drew Weissman[2,*] and Virgil Percec[1,*]

[1]Roy& Diana Vagelos Laboratories, Department of Chemistry, University of Pennsylvania, Philadelphia, PA 19104-6323, USA

[2]Department of Medicine, Perelman School of Medicine, University of Pennsylvania, Philadelphia, PA 19104-6323, USA

*Correspondence: dreww@pennmedicine.upenn.edu (D.W.); percec@sas.upenn.edu (V.P.)
† Dedicated to the 85th Anniversary of Donald A. Tomalia.
‡ These authors contributed equally to this work.

Table of Contents

1. Materials S3
2. Methods and Techniques S3
  2.1 ^{1}H and ^{13}C NMR S3
  2.2 Thin-Layer Chromatography (TLC) S4
  2.3 High-Pressure Liquid Chromatography (HPLC) S4
  2.4 Matrix Assisted Laser Desorption Ionization-Time of Flight (MALDI-TOF) Mass Spectrometry S4
  2.5 Dynamic Light Scattering (DLS) S5
  2.6 pK_a Measurements of Individual IAJDs S5
  2.7 Formulation of DNPs Co-assembled from IAJDs and Luc-mRNA S5
  2.8 Luminescence Characterization for *In Vivo* Transfection Experiments S5
  2.9 *In Vivo* mRNA Delivery in Mice with DNPs S6
  2.10 Molecular Modelling S6
3. Synthesis S7
  3.1 Synthesis of Nonane-Based IAJDs with 3,5-Nonsymmetric Alkyl Chains S7
  3.2 Synthesis of Dodecane-Based IAJDs with 3,5-Nonsymmetric Alkyl Chains S26
  3.3 Synthesis of Tetradecane-Based IAJDs with 3,5-Nonsymmetric Alkyl Chains S42
  3.4 Synthesis of Hexadecane-Based IAJDs with 3,5-Nonsymmetric Alkyl Chains S52
  3.5 Synthesis of Heptadecane-Based IAJDs with 3,5-Nonsymmetric Alkyl Chains S67
  3.6 Synthesis of Octadecane-Based IAJDs with 3,5-Nonsymmetric Alkyl Chains S78
  3.7 Synthesis of Undecane and Pentadecane Based IAJDs with 3,5-Nonsymmetric Alkyl Chains S88
  3.8 Synthesis of Tridecane Based IAJDs with 3,5-Nonsymmetric Alkyl Chains S93
  3.9 Synthesis of IAJD131 .... S107
  3.10 Synthesis of Decane-Based IAJDs with 3,5-Nonsymmetric Alkyl Chains S109
  3.11 Synthesis of IAJDs with 3,5-symmetric Alkyl Chains S116
4. DLS Data of DNPs Co-Assembled from IAJDs and Luc-mRNA S143
5. pK_a Measurements of Individual IAJD Molecules S153
6. Supporting References S166

1. Materials

3,5-Dihydroxybenzoic acid (Acros, 97%), 1-bromononane (Lancaster, 99%), 1-bromododecane (Alfa Aesar, 99%),1- bromotetradecane (Acros, 98%), 1-bromohexadecane (TCI, 96%), 1-heptadecanol (TCI, 97%) (*rac*)-3-(bromomethyl)heptane, 1-bromooctadecane (Acros, 96%), 2-ethylhexyl bromide, (Aldrich, 95%), 1-bromooctane (Aldrich, 99%), 1-bromodecane (Acros, 98%), 1-bromoundecane (Aldrich, 99%), 1-bromopentadecane (Aldrich, 98%), benzyl chloride (Alfa Aesar, 99%), 4- toluenesulfonyl chloride (Alfa Aesar, 98%), palladium on activated carbon catalyst (Spectrum, 10 wt% loading), lithium aluminium hydride ($LiAlH_4$, TCI, 95%), 4-bromobutyric acid (Acros, 98%), thionyl chloride (Alfa Aesar, 99+%), potassium phthalimide (Chem Impex, 99.8%), 1-methylpiperazine (Alfa Aesar, 98%), 1-(2-hydroxyethyl)piperazine (Acros, 99%), triethylamine (TCI, 99%) were used as received. All other reagents and solvents were obtained from commercial sources and were used as received. CH_2Cl_2 (DCM) was dried over CaH_2 and distilled before use. 4-(Dimethylamino)pyridinium 4- toluenesulfonate (DPTS) was prepared according to a literature method.[1] Acetate buffer (10 mM) was prepared by dissolving sodium acetate (2.3 mM) and acetic acid (7.7 mM) in ultra-pure water. Final pH of the buffer was adjusted with 0.1 M HCl or 0.1 M NaOH solution. Nucleoside-modified messenger RNA encoding firefly luciferase (Luc-mRNA) was produced as reported.[2] Human embryonic kidney (HEK) 293T cells (American Type Culture Collection) were cultured in Dulbecco's modified Eagle's medium (DMEM) supplemented with 10% inactivated fetal bovine serum (FBS) (Gemini Bio- Products), 2 mM L–glutamine and 100 U/mL penicillin/streptomycin (Life Technologies). DPBS (Corning), OptiMEM (Gibco), UltraPure DNase/RNase-Free Distilled Water (Invitrogen), Trypsin-EDTA (0.25%, Gibco), Trypan Blue (Sigma-Aldrich), Cell Culture Lysis 5X Reagent (Promega), Luciferase Assay System (Promega) and D-luciferin sodium salt (Regis Technologies) were used as received.

2. Methods and Techniques

The purity and structure of intermediate compounds and final products were determined by a combination of techniques including thin-layer chromatography (TLC), ^1H and ^{13}C NMR, high-pressure liquid chromatography (HPLC), and matrix assisted laser desorption ionization-time of flight (MALDI-TOF) mass spectrometry.

2.1 ^1H and ^{13}C NMR. [3,4,5] ^1H and ^{13}C NMR spectra were recorded at 400 MHz and 101 MHz respectively, on a Bruker NEO (400 MHz) NMR spectrometer equipped with an autosampler, or 500 MHz

and 126 MHz respectively, on a Bruker DRX (500 MHz) NMR spectrometer. All NMR were measured at 23 °C in $CDCl_3$. Chemical shifts (δ) are reported in ppm. The resonance multiplicities in the ^1H NMR spectra are indicated as "s" (singlet), "d" (doublet), "t" (triplet), "m" (multiplet) and "br" (broad resonance). Residual protic solvent of $CDCl_3$ (^1H, δ 7.26 ppm; ^{13}C, δ 77.16 ppm) and tetramethylsilane (TMS, δ 0 ppm) are used as the internal reference in the ^1H and ^{13}C NMR spectra. NMR spectra were analyzed by MNova 14 or TopSpin 4.07 (Bruker).

2.2 Thin-Layer Chromatography (TLC). [3,4,5] TLC was used to monitor the evolution of the extent of reaction by using silica gel 60 F_{254} precoated plates (E. Merck). Individual compounds with aromatic groups were visualized by UV light (λ = 254 nm). For compounds without aromatic groups, the TLC plate was stained with iodine vapor to help visualization. Purification by flash column chromatography (SiO_2) was performed using silica gel from Silicycle (60 Å, 40–63 µm) with the eluent mentioned in the experimental part for each compound in part.

2.3 High-Pressure Liquid Chromatography (HPLC). [3,4,5] The determination of the purity of individual compounds by HPLC was performed by using a Shimadzu LC-20AD high-performance liquid chromatograph pump, a PE Nelson Analytical 900 Series integration data station, a Shimadzu SPD-10A VP (UV-vis, λ = 254 nm) and three AM gel columns (a guard column, two 500 Å, 10 µm columns). THF with 5% of NEt_3 was used as the solvent and the characterization was carried out at 23 °C. Detection of compounds was determined by UV absorbance (λ = 254 nm) or RI (refractive index) detector.

2.4 Matrix Assisted Laser Desorption Ionization-Time of Flight (MALDI-TOF) Mass Spectrometry. [3,4,5] The molar mass of all molecules was determined by MALDI-TOF mass spectrometry employing a PerSeptive Biosystem-Voyager-DE (Framingham, MA) mass spectrometer equipped with nitrogen laser (337 nm) and operating in linear mode. Angiotensin II and Bombesin were used as standards for calibration. For the preparation of sample solution, the corresponding compound was first dissolved in THF (5–10 mg/mL). Subsequently, the matrix (2,5-dihydroxybenzoic acid) was dissolved in THF 10 mg/mL and the two solutions were mixed with a 1/5 (v/v, compound solution/matrix solution) ratio. Then one drop of solution was placed on the MALDI plate and dried at 23 °C. Afterwards, the plate was inserted into the vacuum chamber of the instrument for analysis. The laser intensity and voltages applied for the analysis were adjusted based on the molar mass and nature of each compound.

2.5 Dynamic Light Scattering (DLS). [3,4,5] DLS for the dimensions (sizes and polydispersities) of DNPs was performed on a Malvern Instruments particle sizer (Zetasizer Nano S, Malvern Instruments, UK) equipped with 4 mW He−Ne laser 633 nm and avalanche photodiode positioned at 175° to the beam and temperature-controlled cuvette holder. Instrument parameters were set up automatically along with measurement times. Sample solution (c.a. 0.4 mL) was placed in a semi-micro cuvette (1.6 mL, polystyrene, 10 × 10 × 45 mm, Greiner Bio-One) and the experiments were performed at 23 °C.

2.6 pKa Measurements of Individual IAJDs. [3,4,5] IAJDs were dissolved in ethanol (sat. with NaCl) at a concentration of 1.5 mg/mL and in a volume of 3 mL. Then 0.1 M HCl solution was added to the ethanol solution with an increment of 7.5 µL. The resulting pH, after each addition of HCl solution, was measured by an Thermo Scientific Orion Star A121 meter with Thermo Scientific Orion 8220BNWP pH probe. pK_a was determined using the half equivalence point titration.

2.7 Formulation of DNPs Co-assembled from IAJDs and Luc-mRNA. [3,4,5] Nucleoside-modified mRNA encoding firefly luciferase (Luc-mRNA) was dissolved in UltraPure DNase/RNase-free distilled water with an initial concentration of 4.0 mg/mL. IAJDs were dissolved in ethanol at an initial concentration of 80 mg/mL. Luc-mRNA solution (12.5 µL) was placed into a clean RNAs free eppendorf (1.5 mL and 463 µL of acetate buffer (10 mM, pH 4.0) was added. IAJD stock solution in ethanol was taken (25 µL) and rapidly injected into the above Luc-mRNA solution in acetate buffer followed by vortex for 5 seconds.

2.8 Luminescence Characterization for *In Vivo* Transfection Experiments. [3,4,5] Bioluminescence imaging was performed with an IVIS Spectrum imaging system (PerkinElmer, Waltham, MA). Mice were anesthetized with 3% of isoflurane (Piramal Healthcare Limited) and intraperitoneally (i.p.) administered with D-luciferin (Regis Technologies) at a dose of 150 mg/kg of body weight. Ten min post administration of D-luciferin, mice were placed on the imaging platform while being maintained on isoflurane *via* a nose cone and imaged using a certain exposure time (60, 30, or 15 seconds). Bioluminescence values were quantified by measuring photon flux (photons/second, p/s) in the region of interest (ROI) on mice where bioluminescence signal emanated using the Living Image Software (PerkinElmer). To quantify luminescent flux, an oval ROI was placed over each organ of interest and analyzed.

2.8 Production of nucleoside-modified Luc-mRNA mRNA was produced as previously described[2] using T7 RNA polymerase on linearized DNA encoding codon-optimized Firefly Luciferase and a 101nt poly(A) tail. 1-methylpseudouridine-5'-triphosphate was used instead of UTP. A trinucleotide cap1 analog was added cotranscriptionally. Purification was performed as previously described.[6] mRNA was analyzed for RNase, dsRNA, endotoxin, and other forms of contamination and stored frozen at -20°C.

2.9 *In Vivo* mRNA Delivery in Mice with DNPs. [3,4,5] All mice used were in accordance with the guidelines and approval from the Pennsylvania University Institution of Animal Care and Use Committee. Female or male BALB/c mice (6-8 weeks old, from Charles River Laboratories) were anesthetized with isoflurane (Piramal Healthcare Limited) and injected *via* retro-orbital sinus with 100 µL of DNP solution containing 10 µg of Luc-mRNA. After 4 to 6 hours post injection, mice were i.p. injected with D-Luciferin (150 mg/kg of body weight, Regis Technologies) and imaged on a PerkinElmer IVIS Spectrum CT system (PerkinElmer, Waltham, MA). Tissue luminescence signal was measured on the IVIS imaging system using a certain exposure time (60, 30 or 15 seconds) and medium binning (binning = 8) to ensure that the signal obtained was within operative detection range. For IVIS imaging of the organs, mice were sacrificed, and heart, lungs, liver, and spleen were immediately collected, and bioluminescence imaging was performed as described above. Image analysis was conducted with the Living Image software (PerkinElmer). Bioluminescence values were quantified by measuring photon flux (photons/second) in the region of interest (ROI) using Living Image software.

2.10 Molecular Modelling. Molecular models of the IAJD bilayers were drawn using DS ViewerPro (version 5.0) software. Material Studio Modeling (version 3.1) software from Accelrys was used to perform the energy minimizations of the built models on the supramolecular structures. BIOVIA Discovery Studio Visualizer (version 2019) was used for display style and coloring. Color codes were used as in the ChemDraw structure (hydrophilic part in blue; hydrophobic part in light and dark green; oxygen and OH in pink; carbons in aromatic ring in gray; H groups on aromatic in white).

3. Synthesis

3.1 Synthesis of Nonane-Based IAJDs with 3,5-Nonsymmetric Alkyl Chains

Scheme S1. Synthesis of Nonane-Based IAJDs with 3,5-Nonsymmetric Alkyl Chains

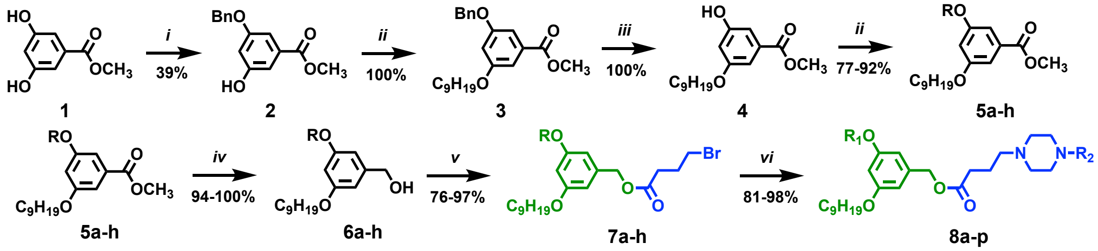

5-7a: R = 2-ethylhexyl
5-7b: R = C_8H_{17}
5-7c: R = $C_{10}H_{21}$
5-7d: R = $C_{12}H_{25}$
5-7e: R = $C_{14}H_{29}$
5-7f: R = $C_{16}H_{33}$
5-7g: R = $C_{17}H_{35}$
5-7h: R = $C_{18}H_{37}$

8a (IAJD179): R_1 = 2-ethylhexyl; R_2 = CH_3
8b (IAJD180): R_1 = 2-ethylhexyl; R_2 = CH_2CH_2OH
8c (IAJD181): R_1 = C_8H_{17}; R_2 = CH_3
8d (IAJD182): R_1 = C_8H_{17}; R_2 = CH_2CH_2OH
8e (IAJD183): R_1 = $C_{10}H_{21}$; R_2 = CH_3
8f (IAJD184): R_1 = $C_{10}H_{21}$; R_2 = CH_2CH_2OH
8g (IAJD185): R_1 = $C_{12}H_{25}$; R_2 = CH_3
8h (IAJD186): R_1 = $C_{12}H_{25}$; R_2 = CH_2CH_2OH
8i (IAJD187): R_1 = $C_{14}H_{29}$; R_2 = CH_3
8j (IAJD188): R_1 = $C_{14}H_{29}$; R_2 = CH_2CH_2OH
8k (IAJD189): R_1 = $C_{16}H_{33}$; R_2 = CH_3
8l (IAJD190): R_1 = $C_{16}H_{33}$; R_2 = CH_2CH_2OH
8m (IAJD191): R_1 = $C_{17}H_{35}$; R_2 = CH_3
8n (IAJD192): R_1 = $C_{17}H_{35}$; R_2 = CH_2CH_2OH
8o (IAJD193): R_1 = $C_{18}H_{37}$; R_2 = CH_3
8p (IAJD194): R_1 = $C_{18}H_{37}$; R_2 = CH_2CH_2OH

Reagents and conditions: (*i*) K_2CO_3, KI, DMF, 80 °C, 5 h; (*ii*) RBr/ROTs, K_2CO_3, DMF, 120 °C, 2 h; (*iii*) H_2, Pd/C, DCM, MeOH, 12 h; (*iv*) $LiAlH_4$, THF, 0–23 °C, 1 h; (*v*) DCC, DPTS, DCM, 12 h; (*vi*) K_2CO_3, MeCN, 95 °C, 3 h.

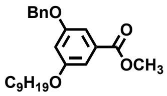

3

Methyl 3-(benzyloxy)-5-(nonyloxy)benzoate (3). The synthesis of methyl 3-(benzyloxy)-5-hydroxybenzoate (**2**) was adapted from literature procedures reported by our laboratory.[3] A mixture of compound **2** (6.50 g, 25.17 mmol, 1 equiv), 1-bromononane (5.74 g, 27.68 mmol, 1.1 equiv), K_2CO_3 (6.96 g, 50.34 mmol, 2 equiv) and DMF (35 mL) was heated to 120 °C and stirred under N_2 atmosphere for 2 h. The reaction mixture was cooled to 23 °C and poured into ice/water (150 mL). The resulted white precipitates in the solution were filtered and collected. Then the precipitates were recrystallized from minimum acetone to afford the title compound as a white solid (7.53 g, 78%). ^1H NMR (400 MHz, $CDCl_3$) δ 7.48–7.31 (m, 5 H, Ph*H*), 7.28 (br, 1 H, Ph*H*), 7.22 (br, 1 H, Ph*H*), 6.74 (t, 1 H, Ph*H*), 5.08 (s, 2 H, $PhCH_2O$-), 3.97 (t, 2 H, $PhOCH_2$-), 3.90 (s, 3 H, $PhCOOCH_3$), 1.78 (m, 2 H, $PhOCH_2CH_2CH_2(CH_2)_5CH_3$), 1.45 (m, 2 H, $PhOCH_2CH_2CH_2(CH_2)_5CH_3$), 1.27 (m, 10 H, $PhOCH_2CH_2CH_2(CH_2)_5CH_3$), 0.89 (t, 3 H, $PhO(CH_2)_8CH_3$). ^{13}C NMR (101 MHz, $CDCl_3$) δ 167.0, 161.3, 160.3, 159.9, 136.7, 132.1, 128.7, 128.2,

127.7, 108.3, 107.9, 107.0, 77.4, 70.3, 68.5, 64.2, 52.3, 32.0, 29.6, 29.5, 29.4, 29.3, 28.6, 26.1, 25.9, 22.8, 14.2. Mp = 31°C.

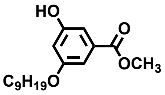

4

Methyl 3-hydroxy-5-(nonyloxy)benzoate (4). Compound **3** (7.53 g, 19.58 mmol) was dissolved in a mixture of DCM (40 mL) and methanol (20 mL). Then Pd/C (0.38 g, 5 wt%) was added to the solution and the flask was evacuated and filled with hydrogen for three times. Afterwards, the mixture was stirred at 23 °C under hydrogen atmosphere for 12 h. The reaction mixture was filtered through Celite, and the filter cake was washed with DCM. Evaporation of the solvent yielded the title compound as a white solid (5.46 g, 100%). ^{1}H NMR (400 MHz, $CDCl_3$) δ 7.12 (d, 2 H, Ph*H*), 6.64 (t, 1 H, Ph*H*), 5.99 (s, 1 H, PhO*H*), 3.93 (t, 2 H, $PhOCH_2$-), 3.90 (s, 3 H, $PhCOOCH_3$), 1.74 (m, 2 H, $PhOCH_2CH_2CH_2(CH_2)_5CH_3$), 1.41 (m, 2 H, $PhOCH_2CH_2CH_2(CH_2)_5CH_3$), 1.27 (m, 10 H, $PhOCH_2CH_2CH_2(CH_2)_5CH_3$), 0.88 (t, 3 H, $PhO(CH_2)_8CH_3$). ^{13}C NMR (101 MHz, $CDCl_3$) δ 167.0, 161.9, 160.5, 157.2, 131.7, 109.3, 107.8, 107.4, 68.5, 52.5, 32.6, 32.0, 31.9, 29.6, 29.5, 29.5, 29.4, 29.3, 29.2, 28.9, 26.1, 22.8, 14.2. Mp = 85 °C.

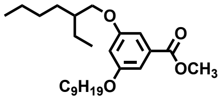

5a

Methyl 3-((2-ethylhexyl)oxy)-5-(nonyloxy)benzoate (5a). A mixture of compound **4** (0.70 g, 2.38 mmol, 1 equiv), 2-ethylhexyl bromide (0.51 g, 2.66 mmol, 1.1 equiv), K_2CO_3 (0.66 g, 4.76 mmol, 2 equiv) and DMF (15 mL) was heated to 80 °C and stirred under N_2 atmosphere for 12 h. The reaction mixture was cooled to 23 °C and DMF was removed under reduced pressure. Then water (20 mL) was added, and the resulted mixture was extracted with DCM (20 mL) for three times. The organic phases were collected and dried over anhydrous $MgSO_4$. After filtration, the obtained filtrate was concentrated to around 3 mL and purified by column chromatography (SiO_2) with hexane/EtOAc = 20/1 as the eluent to give the title compound as a light-yellow oil (0.61 g, 63%). ^{1}H NMR (400 MHz, $CDCl_3$) δ 7.16 (br, 2 H, Ph*H*), 6.63 (t, 1 H, Ph*H*), 3.97 (t, 2 H, $PhOCH_2(CH_2)_{13}CH_3$), 3.89 (s, 3 H, $PhCOOCH_3$), 3.85 (m, 2 H, $PhOCH_2CH(CH_2CH_3)$-), 1.86–1.65 (m, 3 H, $PhOCH_2CH_2(CH_2)_6CH_3$ and $PhOCH_2CH(CH_2CH_3)(CH_2)_3CH_3$), 1.57–1.18 (m, 20 H, $PhOCH_2CH_2(CH_2)_6CH_3$ and $PhOCH_2CH(CH_2CH_3)(CH_2)_3CH_3$), 1.00–0.82 (m, 9 H, $PhO(CH_2)_8CH_3$ and $PhOCH_2CH(CH_2CH_3)(CH_2)_3CH_3$). ^{13}C NMR (101 MHz, $CDCl_3$) δ 167.1, 160.6, 160.3, 131.9, 107.9,

107.6, 106.7, 70.9, 68.4, 52.3, 39.5, 32.0, 30.7, 29.7, 29.5, 29.4, 29.3, 29.2, 26.2, 24.0, 23.2, 22.8, 14.2, 14.2, 11.2.

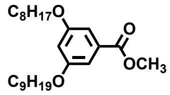

5b

Methyl 3-(octyloxy)-5-(nonyloxy)benzoate (5b). A mixture of compound **4** (0.70 g, 2.38 mmol, 1 equiv), 1-bromooctane (0.51 g, 2.66 mmol, 1.1 equiv), K_2CO_3 (0.66 g, 4.76 mmol, 2 equiv) and DMF (10 mL) was heated to 120 °C and stirred under N_2 atmosphere for 2 h. The reaction mixture was cooled to 23 °C and poured into ice/water (150 mL). The resulted white precipitates in the solution were filtered and collected. Then the precipitates were recrystallized from minimum acetone to afford the title compound as a white solid (0.64 g, 66%). ^{1}H NMR (400 MHz, $CDCl_3$) δ 7.16 (br, 2 H, Ph*H*), 6.64 (t, 1 H, Ph*H*), 3.96 (t, 4 H, $PhOCH_2CH_2CH_2$-), 3.89 (s, 3 H, $PhCOOCH_3$), 1.77 (m, 4 H, $PhOCH_2CH_2CH_2$-), 1.45 (m, 4 H, $PhOCH_2CH_2CH_2$-), 1.28 (m, 18 H, $PhOCH_2CH_2CH_2(CH_2)_4CH_3$ and $PhOCH_2CH_2CH_2(CH_2)_5CH_3$), 0.89 (t, 6 H, $PhO(CH_2)_7CH_3$ and $PhO(CH_2)_8CH_3$). ^{13}C NMR (101 MHz, $CDCl_3$) δ 167.1, 160.3, 132.0, 107.8, 106.7, 77.4, 68.5, 52.3, 32.0, 29.7, 29.5, 29.5, 29.4, 29.4, 29.3, 26.2, 22.8, 14.2. Mp = 36 °C.

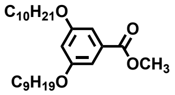

5c

Methyl 3-(dectyloxy)-5-(nonyloxy)benzoate (**5c**). Compound **5c** was synthesized from compound **4** (0.70 g, 2.38 mmol, 1 equiv), 1-bromodecane (0.58 g, 2.66 mmol, 1.1 equiv) and K_2CO_3 (0.66 g, 4.76 mmol, 2 equiv) following a procedure similar to that used for the synthesis of compound **5b**. The title compound was obtained as a white solid (0.68 g, 66%). ^{1}H NMR (400 MHz, $CDCl_3$) δ 7.16 (br, 2 H, Ph*H*), 6.63 (t, 1 H, Ph*H*), 3.96 (t, 4 H, $PhOCH_2CH_2CH_2$-), 3.89 (s, 3 H, $PhCOOCH_3$), 1.77 (m, 4 H, $PhOCH_2CH_2CH_2$-), 1.45 (m, 4 H, $PhOCH_2CH_2CH_2$-), 1.29 (m, 22 H, $PhOCH_2CH_2CH_2(CH_2)_6CH_3$ and $PhOCH_2CH_2CH_2(CH_2)_5CH_3$), 0.88 (t, 6 H, $PhO(CH_2)_9CH_3$ and $PhO(CH_2)_8CH_3$). ^{13}C NMR (101 MHz, $CDCl_3$) δ 167.1, 160.3, 131.9, 107.8, 106.7, 68.4, 52.3, 32.0, 32.0, 29.7, 29.7, 29.7, 29.5, 29.4, 29.4, 29.3, 26.1, 22.8, 14.2. Mp = 41 °C.

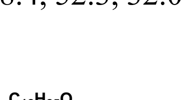

5d

Methyl 3-(dodectyloxy)-5-(nonyloxy)benzoate (**5d**). Compound **5d** was synthesized from compound **4** (0.70 g, 2.38 mmol, 1 equiv), 1-bromododecane (0.63 g, 2.66 mmol, 1.1 equiv) and K_2CO_3 (0.66 g, 4.76 mmol, 2 equiv) following a procedure similar to that used for the synthesis of compound **5b**. The title

compound was obtained as a white solid (0.74 g, 67%). ^{1}H NMR (400 MHz, $CDCl_3$) δ 7.16 (br, 2 H, Ph*H*), 6.63 (t, 1 H, Ph*H*), 3.96 (t, 4 H, $PhOCH_2CH_2CH_2$-), 3.89 (s, 3 H, $PhCOOCH_3$), 1.77 (m, 4 H, $PhOCH_2CH_2CH_2$-), 1.45 (m, 4 H, $PhOCH_2CH_2CH_2$-), 1.28 (m, 26 H, $PhOCH_2CH_2CH_2(CH_2)_8CH_3$ and $PhOCH_2CH_2CH_2(CH_2)_5CH_3$), 0.89 (t, 6 H, $PhO(CH_2)_{11}CH_3$ and $PhO(CH_2)_8CH_3$). ^{13}C NMR (101 MHz, $CDCl_3$) δ 167.1, 160.3, 132.0, 107.8, 106.7, 68.5, 52.3, 32.1, 32.0, 29.8, 29.8, 29.7, 29.7, 29.5, 29.4, 29.3, 26.2, 22.8, 14.2. Mp = 48 °C.

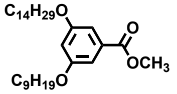

5e

Methyl 3-(nonyloxy)-5-(tetradecyloxy)benzoate (5e). Compound **5e** was synthesized from compound **4** (0.70 g, 2.38 mmol, 1 equiv), 1-bromotetradecane (727 mg, 2.62 mmol, 1.1 equiv) and K_2CO_3 (0.66 g, 4.76 mmol, 2 equiv) following a procedure similar to that used for the synthesis of compound **5b**. The title compound was obtained as a white solid (1.05 g, 90%). ^{1}H NMR (400 MHz, $CDCl_3$) δ 7.15 (br, 2 H, Ph*H*), 6.63 (t, 1 H, Ph*H*), 3.96 (t, 4 H, $PhOCH_2CH_2CH_2$-), 3.89 (s, 3 H, $PhCOOCH_3$), 1.77 (m, 4 H, $PhOCH_2CH_2CH_2$-), 1.44 (m, 4 H, $PhOCH_2CH_2CH_2$-), 1.27 (m, 30 H, $PhOCH_2CH_2CH_2(CH_2)_{10}CH_3$ and $PhOCH_2CH_2CH_2(CH_2)_5CH_3$), 0.89 (t, 6 H, $PhO(CH_2)_{13}CH_3$ and $PhO(CH_2)_8CH_3$). ^{13}C NMR (101 MHz, $CDCl_3$) δ 167.0, 160.2, 131.8, 107.7, 106.6, 77.4, 68.3, 52.2, 31.9, 31.9, 29.7, 29.7, 29.6 29.6, 29.5, 29.4, 29.3, 26.2, 25.9, 22.7, 22.7, 14.2. Mp = 53 °C.

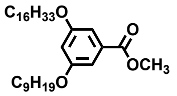

5f

Methyl 3-(hexadecyloxy)-5-(nonyloxy)benzoate (5f). Compound **5f** was synthesized from compound **4** (0.70 g, 2.38 mmol, 1 equiv), 1-bromohexadecane (0.80 g, 2.66 mmol, 1.1 equiv) and K_2CO_3 (0.66 g, 4.76 mmol, 2 equiv) following a procedure similar to that used for the synthesis of compound **5b**. The title compound was obtained as a white solid (1.24 g, 100%). ^{1}H NMR (400 MHz, $CDCl_3$) δ 7.15 (br, 2 H, Ph*H*), 6.63 (t, 1 H, Ph*H*), 3.96 (t, 4 H, $PhOCH_2CH_2CH_2$-), 3.89 (s, 3 H, $PhCOOCH_3$), 1.77 (m, 4 H, $PhOCH_2CH_2CH_2$-), 1.44 (m, 4 H, $PhOCH_2CH_2CH_2$-), 1.27 (m, 34 H, $PhOCH_2CH_2CH_2(CH_2)_{12}CH_3$ and $PhOCH_2CH_2CH_2(CH_2)_5CH_3$), 0.89 (t, 6 H, $PhO(CH_2)_{15}CH_3$ and $PhO(CH_2)_8CH_3$). ^{13}C NMR (101 MHz, $CDCl_3$) δ 167.1, 160.3, 132.0, 107.8, 106.7, 77.4, 68.5, 52.3, 32.1, 32.0, 29.8, 29.8, 29.8, 29.7, 29.6, 29.5, 29.4, 29.3, 26.2, 25.9, 22.8, 22.8, 14.3. Mp = 55 °C.

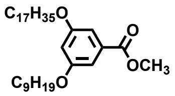

5g

Methyl 3-(heptadectyloxy)-5-(nonyloxy)benzoate (5g). Heptadecyl 4-methylbenzenesulfonate ($C_{17}H_{35}OTs$) was synthesized according to a literature procedure reported by our laboratory.[5] Compound **5g** was synthesized from compound **4** (0.70 g, 2.38 mmol, 1 equiv), $C_{17}H_{35}OTs$ (1.08 g, 2.66 mmol, 1.1 equiv) and K_2CO_3 (0.66 g, 4.76 mmol, 2 equiv) following a procedure similar to that used for the synthesis of compound **5b**. The title compound was obtained as a white solid (1.05 g, 83%). ^1H NMR (400 MHz, $CDCl_3$) δ 7.15 (br, 2 H, Ph*H*), 6.63 (t, 1 H, Ph*H*), 3.96 (t, 4 H, PhOCH_2CH$_2$CH$_2$-), 3.89 (s, 3 H, PhCOOCH_3), 1.77 (m, 4 H, PhOCH$_2$CH_2CH$_2$-), 1.44 (m, 4 H, PhOCH$_2$CH$_2$CH_2-), 1.28 (m, 36 H, PhOCH$_2$CH$_2$CH$_2$(CH_2)$_{13}$CH$_3$ and PhOCH$_2$CH$_2$CH$_2$(CH_2)$_5$CH$_3$), 0.88 (t, 6 H, PhO(CH$_2$)$_{16}$CH_3 and PhO(CH$_2$)$_8$CH_3). ^{13}C NMR (101 MHz, $CDCl_3$) δ 167.1, 160.3, 132.0, 107.8, 106.7, 77.4, 68.5, 63.1, 52.3, 32.9, 32.1, 32.0, 29.8, 29.8, 29.8, 29.7, 29.7, 29.6, 29.5, 29.4, 29.3, 26.2, 22.8, 22.8, 14.3. Mp = 58 °C.

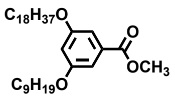

5h

Methyl 3-(octadectyloxy)-5-(nonyloxy)benzoate (5h). Compound **5h** was synthesized from compound **4** (0.63 g, 1.50 mmol, 1 equiv), 1-bromoctadecanene (0.34 g, 1.65 mmol, 1.1 equiv) and K_2CO_3 (0.42 g, 3.00 mmol, 2 equiv) following a procedure similar to that used for the synthesis of compound **5b**. The title compound was obtained as a white solid (0.76 g, 94%). ^1H NMR (400 MHz, $CDCl_3$) δ 7.16 (br, 2 H, Ph*H*), 6.63 (t, 1 H, Ph*H*), 3.97 (t, 4 H, PhOCH_2CH$_2$CH$_2$-), 3.89 (s, 3 H, PhCOOCH_3), 1.77 (m, 4 H, PhOCH$_2$CH_2CH$_2$-), 1.45 (m, 4 H, PhOCH$_2$CH$_2$CH_2-), 1.28 (m, 38 H, PhOCH$_2$CH$_2$CH$_2$(CH_2)$_{14}$CH$_3$ and PhOCH$_2$CH$_2$CH$_2$(CH_2)$_5$CH$_3$), 0.89 (t, 6 H, PhO(CH$_2$)$_{17}$CH_3 and PhO(CH$_2$)$_8$CH_3). ^{13}C NMR (101 MHz, $CDCl_3$) δ 167.1, 160.3, 132.0, 107.8, 106.7, 68.5, 52.3, 32.1, 32.0, 29.8, 29.7, 29.7, 29.5, 29.4, 29.3, 26.2, 22.8, 22.8, 14.2. Mp = 60 °C.

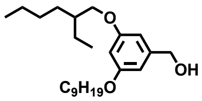

6a

(3-(2-Ethylhexyl)oxy)-5-(nonyloxy)phenyl)methanol (6a). Compound **5a** (0.61 g, 1.50 mmol, 1 equiv) was dissolved in 5 mL dry THF, which was added dropwise to a slurry of $LiAlH_4$ (57 mg, 1.50 mmol, 1 equiv) in dry THF (5 mL) at 0 °C under N_2 atmosphere. Afterwards, the resulted mixture was stirred at 23 °C for 1 h. Finally, the reaction was quenched by the successive addition of water (0.2 mL), 15% NaOH aqueous solution (0.2 mL) and water (1.0 mL). White precipitates were formed in the mixture and filtered

out. The filtrate was dried over anhydrous $MgSO_4$ and filtered. The solvent was removed under reduced pressure to afford the title compound as a colorless oil (0.52 g, 93%). ^{1}H NMR (400 MHz, $CDCl_3$) δ 6.50 (d, 2 H, Ph*H*), 6.38 (t, 1H, Ph*H*), 4.61 (s, 2 H, $PhCH_2OH$), 3.95 (t, 2 H, $PhOCH_2(CH_2)_7CH_3$), 3.82 (m, 2 H, $PhOCH_2CH(CH_2CH_3)$-), 1.83–1.62 (m, 3 H, $PhOCH_2CH_2(CH_2)_6CH_3$ and $PhOCH_2CH(CH_2CH_3)(CH_2)_3CH_3$), 1.60–1.20 (m, 20 H, $PhOCH_2CH_2(CH_2)_6CH_3$ and $PhOCH_2CH(CH_2CH_3)(CH_2)_3CH_3$), 0.91 (m, 9 H, $PhO(CH_2)_8CH_3$ and $PhOCH_2CH(CH_2CH_3)(CH_2)_3CH_3$). ^{13}C NMR (101 MHz, $CDCl_3$) δ 160.9, 160.7, 143.3, 107.9, 105.3, 105.2, 105.1, 100.7, 70.7, 68.5, 68.2, 65.6, 39.5, 32.9, 32.0, 30.7, 30.5, 29.7, 29.6, 29.5, 29.5, 29.4, 29.4, 29.2, 26.2, 25.9, 24.0, 23.2, 22.8, 14.2, 14.2, 11.2.

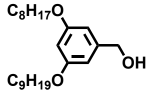

6b

(3-(Octyloxy)-5-(nonyloxy)phenyl)methanol (6b). Compound **6b** was synthesized from compound **5b** (0.68 g, 1.48 mmol, 1 equiv) and $LiAlH_4$ (56 mg, 1.48 mmol, 1 equiv) following a procedure similar to that used for the synthesis of compound **6a**. The title compound was obtained as a colorless oil (0.53 g, 95%). ^{1}H NMR (400 MHz, $CDCl_3$) δ 6.50 (br, 2 H, Ph*H*), 6.38 (t, 1 H, Ph*H*), 4.61 (s, 2 H, $PhCH_2OH$), 3.93 (t, 4 H, $PhOCH_2CH_2CH_2$-), 1.77 (m, 4 H, $PhOCH_2CH_2CH_2$-), 1.44 (m, 4 H, $PhOCH_2CH_2CH_2$-), 1.28 (m, 18 H, $PhOCH_2CH_2CH_2(CH_2)_4CH_3$ and $PhOCH_2CH_2CH_2(CH_2)_5CH_3$), 0.88 (t, 6 H, $PhO(CH_2)_7CH_3$ and $PhO(CH_2)_8CH_3$). ^{13}C NMR (101 MHz, $CDCl_3$) δ 160.7, 160.7, 143.3, 105.2, 100.7, 68.2, 65.6, 32.0, 32.0, 29.7, 29.5, 29.5, 29.4, 29.4, 26.2, 22.8, 14.2.

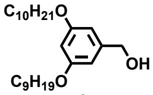

6c

(3-(Decyloxy)-5-(nonyloxy)phenyl)methanol (6c). Compound **6c** was synthesized from compound **5c** (0.68 g, 1.56 mmol, 1 equiv) and $LiAlH_4$ (60 mg, 1.56 mmol, 1 equiv) following a procedure similar to that used for the synthesis of compound **6a**. The title compound was obtained as a colorless oil (0.54 g, 85%). ^{1}H NMR (400 MHz, $CDCl_3$) δ 6.50 (br, 2 H, Ph*H*), 6.38 (t, 1 H, Ph*H*), 4.60 (s, 2 H, $PhCH_2OH$), 3.93 (t, 4 H, $PhOCH_2CH_2CH_2$-), 1.76 (m, 4 H, $PhOCH_2CH_2CH_2$-), 1.44 (m, 4 H, $PhOCH_2CH_2CH_2$-), 1.29 (m, 22 H, $PhOCH_2CH_2CH_2(CH_2)_6CH_3$ and $PhOCH_2CH_2CH_2(CH_2)_5CH_3$), 0.89 (t, 6 H, $PhO(CH_2)_9CH_3$ and $PhO(CH_2)_8CH_3$). ^{13}C NMR (101 MHz, $CDCl_3$) δ 160.7, 143.4, 143.4, 105.2, 100.7, 77.4, 68.2, 65.5, 65.5, 32.0, 32.0, 29.7, 29.7, 29.7, 29.5, 29.5, 29.4, 26.2, 22.8, 14.2.

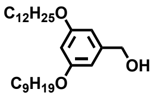
6d

(3-(Decyloxy)-5-(nonyloxy)phenyl)methanol (6d). Compound **6d** was synthesized from compound **5d** (0.71 g, 1.53 mmol, 1 equiv) and $LiAlH_4$ (59 mg, 1.53 mmol, 1 equiv) following a procedure similar to that used for the synthesis of compound **6a**. The title compound was obtained as a colorless oil (0.58 g, 87%). ^1H NMR (400 MHz, $CDCl_3$) δ 6.50 (br, 2 H, Ph*H*), 6.37 (t, 1 H, Ph*H*), 4.60 (s, 2 H, $PhCH_2OH$), 3.93 (t, 4 H, $PhOCH_2CH_2CH_2$-), 1.76 (m, 4 H, $PhOCH_2CH_2CH_2$-), 1.44 (m, 4 H, $PhOCH_2CH_2CH_2$-), 1.29 (m, 26 H, $PhOCH_2CH_2CH_2(CH_2)_8CH_3$ and $PhOCH_2CH_2CH_2(CH_2)_5CH_3$), 0.89 (t, 6 H, $PhO(CH_2)_{11}CH_3$ and $PhO(CH_2)_8CH_3$). ^{13}C NMR (101 MHz, $CDCl_3$) δ 160.7, 143.3, 105.2, 100.7, 77.5, 77.4, 77.2, 76.8, 68.2, 65.5, 32.1, 32.0, 29.8, 29.8, 29.8, 29.7, 29.7, 29.5, 29.5, 29.4, 26.2, 22.8, 22.8, 14.2.

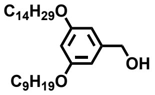
6e

(3-(Nonyloxy)-5-(tetradecyloxy)phenyl)methanol (6e). Compound **6e** was synthesized from compound **5e** (0.60 g, 0.22 mmol, 1 equiv) and $LiAlH_4$ (46 mg, 1.22 mmol, 1 equiv) following a procedure similar to that used for the synthesis of compound **6a**. The title compound was obtained as a colorless oil (0.55 g, 97%). ^1H NMR (400 MHz, $CDCl_3$) δ 6.50 (br, 2 H, Ph*H*), 6.37 (t, 1 H, Ph*H*), 4.60 (s, 2 H, $PhCH_2OH$), 3.93 (t, 4 H, $PhOCH_2CH_2CH_2$-), 1.75 (m, 4 H, $PhOCH_2CH_2CH_2$-), 1.43 (m, 4 H, $PhOCH_2CH_2CH_2$-), 1.26 (m, 30 H, $PhOCH_2CH_2CH_2(CH_2)_{10}CH_3$ and $PhOCH_2CH_2CH_2(CH_2)_5CH_3$), 0.88 (t, 6 H, $PhO(CH_2)_{13}CH_3$ and $PhO(CH_2)_8CH_3$). ^{13}C NMR (101 MHz, $CDCl_3$) δ 160.6, 143.2, 105.1, 100.6, 77.4, 77.4, 77.0, 76.7, 68.2, 65.5, 32.1, 32.0, 29.8, 29.8, 29.8, 29.7, 29.7, 29.6, 29.4, 29.3, 26.1, 22.7, 22.7, 14.2.

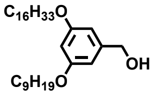
6f

(3-(hexadecyloxy)-5-(nonyloxy)phenyl)methanol (6f). Compound **6f** was synthesized from compound **5f** (1.7 g, 2.25 mmol, 1 equiv) and $LiAlH_4$ (85 mg, 2.25 mmol, 1 equiv) following a procedure similar to that used for the synthesis of compound **6a**. The title compound was obtained as a colorless oil (1.10 g, 100%). ^1H NMR (400 MHz, $CDCl_3$) δ 6.51 (br, 2 H, Ph*H*), 6.38 (t, 1 H, Ph*H*), 4.60 (s, 2 H, $PhCH_2OH$), 3.93 (t, 4 H, $PhOCH_2CH_2CH_2$-), 1.75 (m, 4 H, $PhOCH_2CH_2CH_2$-), 1.44 (m, 4 H, $PhOCH_2CH_2CH_2$-), 1.26 (m, 34 H, $PhOCH_2CH_2CH_2(CH_2)_{12}CH_3$ and $PhOCH_2CH_2CH_2(CH_2)_5CH_3$), 0.88 (t, 6 H, $PhO(CH_2)_{15}CH_3$ and $PhO(CH_2)_8CH_3$). ^{13}C NMR (101 MHz, $CDCl_3$) δ 160.6, 143.2, 105.1, 100.6, 77.4, 77.4, 77.0, 76.7, 68.2, 65.5, 32.1, 32.0, 29.8, 29.8, 29.8, 29.7, 29.7, 29.6, 29.4, 29.3, 26.1, 22.7, 22.7, 14.2.

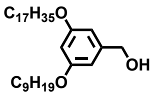

6g

(3-(Heptadectyloxy)-5-(nonyloxy)phenyl)methanol (6g). Compound **6g** was synthesized from compound **5g** (1.01 g, 1.90 mmol, 1 equiv) and $LiAlH_4$ (44 mg, 1.90 mmol, 1 equiv) following a procedure similar to that used for the synthesis of compound **6a**. The title compound was obtained as a white solid (0.91 g, 95%). ^{1}H NMR (400 MHz, $CDCl_3$) δ 6.50 (br, 2 H, Ph*H*), 6.38 (t, 1 H, Ph*H*), 4.61 (s, 2 H, $PhCH_2OH$), 3.93 (t, 4 H, $PhOCH_2CH_2CH_2$-), 1.76 (m, 4 H, $PhOCH_2CH_2CH_2$-), 1.43 (m, 4 H, $PhOCH_2CH_2CH_2$-), 1.27 (m, 22 H, $PhOCH_2CH_2CH_2(CH_2)_{13}CH_3$ and $PhOCH_2CH_2CH_2(CH_2)_5CH_3$), 0.89 (t, 6 H, $PhO(CH_2)_{16}CH_3$ and $PhO(CH_2)_8CH_3$). ^{13}C NMR (101 MHz, $CDCl_3$) δ 160.7, 143.4, 105.2, 100.7, 77.4, 68.2, 65.5, 63.2, 32.9, 32.1, 32.0, 29.8, 29.8, 29.8, 29.7, 29.7, 29.6, 29.5, 29.5, 29.4, 26.2, 25.9, 22.8, 22.8, 14.2. Mp = 40 °C.

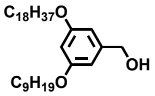

6h

(3-(Octadectyloxy)-5-(nonyloxy)phenyl)methanol (6h). Compound **6h** was synthesized from compound **5h** (0.75 g, 1.14 mmol, 1 equiv) and $LiAlH_4$ (44 mg, 1.14 mmol, 1 equiv) following a procedure similar to that used for the synthesis of compound **6a**. The title compound was obtained as a white solid (0.51 g, 97%). ^{1}H NMR (400 MHz, $CDCl_3$) δ 6.49 (br, 2 H, Ph*H*), 6.38 (t, 1 H, Ph*H*), 4.61 (s, 2 H, $PhCH_2OH$), 3.93 (t, 4 H, $PhOCH_2CH_2CH_2$-), 1.76 (m, 4 H, $PhOCH_2CH_2CH_2$-), 1.44 (m, 4 H, $PhOCH_2CH_2CH_2$-), 1.29 (m, 22 H, $PhOCH_2CH_2CH_2(CH_2)_6CH_3$ and $PhOCH_2CH_2CH_2(CH_2)_5CH_3$), 0.89 (t, 6 H, $PhO(CH_2)_9CH_3$ and $PhO(CH_2)_8CH_3$). ^{13}C NMR (101 MHz, $CDCl_3$) δ 160.7, 143.3, 105.2, 100.7, 68.2, 65.6, 32.1, 32.0, 29.8, 29.8, 29.8, 29.7, 29.7, 29.5, 29.5, 29.4, 26.2, 22.8, 22.8, 14.2. Mp = 41 °C.

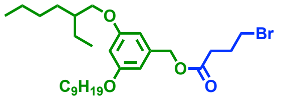

7a

(3-(2-Ethylhexyl)oxy)-5-(nonyloxy)benzyl 4-bromobutanoate (7a). Compound **6a** (0.50 g, 1.33 mmol, 1 equiv), 4-bromobutyric acid (0.33 g, 1.98 mmol, 1.5 equiv) and DPTS (0.58 g, 1.98 mmol, 1.5 equiv) were dissolved in 10 mL DCM. *N,N'*-Dicyclohexylcarbodiimide (DCC, 0.82 g, 3.96 mmol, 3 equiv) was added into the above mixture. The reaction mixture was stirred at 23 °C for 12 h. Afterwards, the resulted precipitates (urea) were filtered out from the reaction mixture. The filtrate was concentrated to around 3 mL and purified by column chromatography (SiO_2) with hexane/DCM = 1/1 as the mobile phase to give

the title compound as a colorless oil (0.52 g, 75%). ^{1}H NMR (400 MHz, $CDCl_3$) δ 6.47 (d, 2 H, PhH), 6.41 (t, 1H, PhH), 5.05 (s, 2 H, $PhC\mathit{H}_2OCO$), 3.93 (t, 2 H, $PhOC\mathit{H}_2(CH_2)_7CH_3$), 3.82 (m, 2 H,$PhOC\mathit{H}_2CH(CH_2CH_3)$-), 3.47 (t, 2 H, -$OCOCH_2CH_2C\mathit{H}_2Br$), 2.56 (t, 2 H, -$OCOC\mathit{H}_2CH_2CH_2Br$), 2.20 (m, 2 H, -$OCOCH_2C\mathit{H}_2CH_2Br$), 1.87–1.67 (m, 3 H, $PhOCH_2C\mathit{H}_2(CH_2)_6CH_3$ and $PhOCH_2C\mathit{H}(CH_2CH_3)(CH_2)_3CH_3$), 1.57–1.21 (m, 20 H, $PhOCH_2CH_2(C\mathit{H}_2)_6CH_3$ and $PhOCH_2CH(C\mathit{H}_2CH_3)(C\mathit{H}_2)_3CH_3$), 0.91 (m, 9 H, $PhO(CH_2)_8C\mathit{H}_3$ and $PhOCH_2CH(CH_2C\mathit{H}_3)(CH_2)_3C\mathit{H}_3$). ^{13}C NMR (101 MHz, $CDCl_3$) δ 172.4, 160.9, 160.6, 137.9, 106.6, 106.4, 101.2, 70.7, 68.2, 66.6, 39.5, 32.8, 32.6, 32.0, 30.7, 29.7, 29.5, 29.5, 29.4, 29.2, 27.9, 26.2, 24.0, 23.2, 22.8, 14.2, 14.2, 11.3.

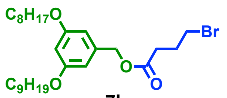

7b

3-(Octyloxy)-5-(nonyloxy)benzyl 4-bromobutanoate (7b). Compound **7b** was synthesized from compound **6b** (0.51 g, 1.35 mmol, 1 equiv), 4-bromobutyric acid (0.34 g, 2.02 mmol, 1.5 equiv), DPTS (0.60 g, 2.02 mmol, 1.5 equiv) and DCC (0.84 g, 4.05 mmol, 3 equiv) following a procedure similar to that used for the synthesis of compound **7a**. The title compound was obtained as a colorless oil (0.70 g, 98%). ^{1}H NMR (400 MHz, $CDCl_3$) δ 6.47 (br, 2 H, PhH), 6.41 (t, 1 H, PhH), 5.05 (s, 2 H, $PhC\mathit{H}_2OCO$), 3.93 (t, 4 H, $PhOC\mathit{H}_2CH_2CH_2$-), 3.47 (t, 2 H, -$OCOCH_2CH_2C\mathit{H}_2Br$), 2.57 (t, 2 H, -$OCOC\mathit{H}_2CH_2CH_2Br$), 2.20 (m, 2 H, -$OCOCH_2C\mathit{H}_2CH_2Br$), 1.77 (m, 4 H, $PhOCH_2C\mathit{H}_2CH_2$-), 1.44 (m, 4 H, $PhOCH_2CH_2C\mathit{H}_2$-), 1.28 (m, 18 H, $PhOCH_2CH_2CH_2(C\mathit{H}_2)_4CH_3$ and $PhOCH_2CH_2CH_2(C\mathit{H}_2)_5CH_3$), 0.89 (t, 6 H, $PhO(CH_2)_7C\mathit{H}_3$ and $PhO(CH_2)_8C\mathit{H}_3$). ^{13}C NMR (101 MHz, $CDCl_3$) δ 172.4, 172.4, 160.6, 137.9, 106.5, 101.2, 68.2, 66.5, 32.7, 32.6, 32.0, 32.0, 29.7, 29.5, 29.5, 29.4, 27.9, 26.2, 22.8, 14.2.

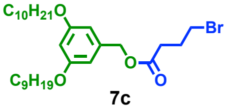

7c

3-(Dectyloxy)-5-(nonyloxy)benzyl 4-bromobutanoate (7c). Compound **7c** was synthesized from compound **6c** (0.52 g, 1.27 mmol, 1 equiv), 4-bromobutyric acid (0.30 g, 1.90 mmol, 1.5 equiv), DPTS (0.53 g, 1.90 mmol, 1.5 equiv) and DCC (0.75 g, 3.80 mmol, 3 equiv) following a procedure similar to that used for the synthesis of compound **7a**. The title compound was obtained as a colorless oil (0.61 g, 92%). ^{1}H NMR (400 MHz, $CDCl_3$) δ 6.46 (br, 2 H, PhH), 6.40 (t, 1 H, PhH), 5.05 (s, 2 H, $PhC\mathit{H}_2OCO$), 3.93 (t, 4 H, $PhOC\mathit{H}_2CH_2CH_2$-), 3.47 (t, 2 H, -$OCOCH_2CH_2C\mathit{H}_2Br$), 2.56 (t, 2 H, -$OCOC\mathit{H}_2CH_2CH_2Br$), 2.20 (m, 2 H, -$OCOCH_2C\mathit{H}_2CH_2Br$), 1.76 (m, 4 H, $PhOCH_2C\mathit{H}_2CH_2$-), 1.44 (m, 4 H, $PhOCH_2CH_2C\mathit{H}_2$-), 1.28 (m, 22 H, $PhOCH_2CH_2CH_2(C\mathit{H}_2)_6CH_3$ and $PhOCH_2CH_2CH_2(C\mathit{H}_2)_5CH_3$), 0.89 (t, 6 H,

$PhO(CH_2)_9CH_3$ and $PhO(CH_2)_8CH_3$). ^{13}C NMR (101 MHz, $CDCl_3$) δ 172.4, 172.4, 160.6, 137.9, 106.5, 101.2, 77.4, 68.2, 66.5, 32.8, 32.6, 32.0, 32.0, 29.7, 29.7, 29.7, 29.5, 29.5, 29.4, 27.9, 26.2, 22.8, 14.2.

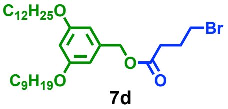

7d

3-(Dodectyloxy)-5-(nonyloxy)benzyl 4-bromobutanoate (7d). Compound **7d** was synthesized from compound **6d** (0.60 g, 1.30 mmol, 1 equiv), 4-bromobutyric acid (0.54 g, 1.86 mmol, 1.5 equiv), DPTS (0.55 g, 1.86 mmol, 1.5 equiv) and DCC (0.71 g, 3.42 mmol, 3 equiv) following a procedure similar to that used for the synthesis of compound **7a**. The title compound was obtained as a colorless oil (0.74 g, 100%). ^{1}H NMR (400 MHz, $CDCl_3$) δ 6.47 (br, 2 H, Ph*H*), 6.41 (t, 1 H, Ph*H*), 5.04 (s, 2 H, $PhCH_2OCO$), 3.93 (t, 4 H, $PhOCH_2CH_2CH_2$-), 3.47 (t, 2 H, -$OCOCH_2CH_2CH_2Br$), 2.56 (t, 2 H, -$OCOCH_2CH_2CH_2Br$), 2.20 (m, 2 H, -$OCOCH_2CH_2CH_2Br$), 1.76 (m, 4 H, $PhOCH_2CH_2CH_2$-), 1.44 (m, 4 H, $PhOCH_2CH_2CH_2$-), 1.28 (m, 26 H, $PhOCH_2CH_2CH_2(CH_2)_8CH_3$ and $PhOCH_2CH_2CH_2(CH_2)_5CH_3$), 0.88 (t, 6 H, $PhO(CH_2)_{11}CH_3$ and $PhO(CH_2)_8CH_3$). ^{13}C NMR (101 MHz, $CDCl_3$) δ 172.4, 160.6, 137.9, 106.5, 101.2, 77.4, 68.2, 66.5, 64.9, 32.7, 32.6, 32.1, 32.0, 29.8, 29.8, 29.7, 29.7, 29.7, 29.5, 29.5, 29.4, 28.7, 27.9, 26.2, 26.0, 22.8, 22.8, 14.2.

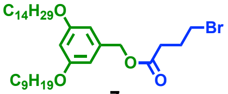

7e

3-(Nonyloxy)-5-(tetradecyloxy)benzyl 4-bromobutanoate (7e). Compound **7e** was synthesized from compound **6e** (0.55 g, 1.19 mmol, 1 equiv), 4-bromobutyric acid (297 g, 1.78 mmol, 1.5 equiv), DPTS (382 mg, 1.78 mmol, 1.5 equiv) and DCC (737 mg, 3.57 mmol, 3 equiv) following a procedure similar to that used for the synthesis of compound **7a**. The title compound was obtained as a colorless oil (0.43 g, 59%). ^{1}H NMR (400 MHz, $CDCl_3$) δ 6.47 (br, 2 H, Ph*H*), 6.40 (t, 1 H, Ph*H*), 5.01 (s, 2 H, $PhCH_2OCO$), 3.93 (t, 4 H, $PhOCH_2CH_2CH_2$-), 3.44 (t, 2 H, -$OCOCH_2CH_2CH_2Br$), 2.55 (t, 2 H, -$OCOCH_2CH_2CH_2Br$), 2.20 (m, 2 H, -$OCOCH_2CH_2CH_2Br$), 1.77 (m, 4 H, $PhOCH_2CH_2CH_2$-), 1.43 (m, 4 H, $PhOCH_2CH_2CH_2$-), 1.27 (m, 30 H, $PhOCH_2CH_2CH_2(CH_2)_{10}CH_3$ and $PhOCH_2CH_2CH_2(CH_2)_5CH_3$), 0.88 (t, 6 H, $PhO(CH_2)_{13}CH_3$ and $PhO(CH_2)_8CH_3$). ^{13}C NMR (101 MHz, $CDCl_3$) δ 172.3, 160.5, 137.8, 106.4, 101.2, 68.1, 66.4, 32.6, 32.5, 31.9, 31.8, 29.6, 29.6, 29.4, 29.4, 29.4, 29.3, 29.3, 27.8, 26.1, 22.7, 22.7, 14.1.

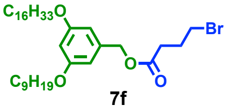

7f

3-(Hexadecyloxy)-5-(nonyloxy)benzyl 4-bromobutanoate (7f). Compound **7f** was synthesized from compound **6f** (0.55 g, 1.02 mmol, 1 equiv), 4-bromobutyric acid (256 g, 1.53 mmol, 1.5 equiv), DPTS (450 mg, 1.53 mmol, 1.5 equiv) and DCC (631 mg, 3.06 mmol, 3 equiv) following a procedure similar to that used for the synthesis of compound **7a**. The title compound was obtained as a colorless oil (0.53 g, 83%). ^{1}H NMR (400 MHz, $CDCl_3$) δ 6.46 (br, 2 H, Ph*H*), 6.39 (t, 1 H, Ph*H*), 5.01 (s, 2 H, $PhCH_2OCO$), 3.92 (t, 4 H, $PhOCH_2CH_2CH_2$-), 3.44 (t, 2 H, -$OCOCH_2CH_2CH_2Br$), 2.55 (t, 2 H, -$OCOCH_2CH_2CH_2Br$), 2.24 (m, 2 H, -$OCOCH_2CH_2CH_2Br$), 1.76 (m, 4 H, $PhOCH_2CH_2CH_2$-), 1.37 (m, 4 H, $PhOCH_2CH_2CH_2$-), 1.27 (m, 34 H, $PhOCH_2CH_2CH_2(CH_2)_{12}CH_3$ and $PhOCH_2CH_2CH_2(CH_2)_5CH_3$), 0.88 (t, 6 H, $PhO(CH_2)_{13}CH_3$ and $PhO(CH_2)_8CH_3$). ^{13}C NMR (101 MHz, $CDCl_3$) δ 172.3, 160.5, 137.8, 106.4, 101.2, 68.1, 66.4, 32.6, 32.5, 31.9, 31.8, 29.6, 29.6, 29.4, 29.4, 29.4, 29.3, 29.3, 27.8, 26.1, 22.7, 22.7, 14.1.

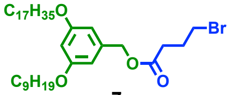

7g

3-(Heptectyloxy)-5-(nonyloxy)benzyl 4-bromobutanoate (7g). Compound **7g** was synthesized from compound **6g** (0.45 g, 0.89 mmol, 1 equiv), 4-bromobutyric acid (0.23 g, 1.34 mmol, 1.5 equiv), DPTS (0.38 g, 1.34 mmol, 1.5 equiv) and DCC (0.55 g, 2.67 mmol, 3 equiv) following a procedure similar to that used for the synthesis of compound **7a**. The title compound was obtained as a colorless oil (0.60 g, 75%). ^{1}H NMR (400 MHz, $CDCl_3$) δ 6.46 (br, 2 H, Ph*H*), 6.40 (t, 1 H, Ph*H*), 5.04 (s, 2 H, $PhCH_2OCO$), 3.93 (t, 4 H, $PhOCH_2CH_2CH_2$-), 3.47 (t, 2 H, -$OCOCH_2CH_2CH_2Br$), 2.56 (t, 2 H, -$OCOCH_2CH_2CH_2Br$), 2.20 (m, 2 H, -$OCOCH_2CH_2CH_2Br$), 1.76 (m, 4 H, $PhOCH_2CH_2CH_2$-), 1.44 (m, 4 H, $PhOCH_2CH_2CH_2$-), 1.28 (m, 36 H, $PhOCH_2CH_2CH_2(CH_2)_{13}CH_3$ and $PhOCH_2CH_2CH_2(CH_2)_5CH_3$), 0.88 (t, 6 H, $PhO(CH_2)_{16}CH_3$ and $PhO(CH_2)_8CH_3$). ^{13}C NMR (101 MHz, $CDCl_3$) δ 172.4, 160.6, 137.9, 106.5, 101.2, 68.2, 66.6, 64.9, 32.9, 32.8, 32.7, 32.6, 32.1, 32.0, 29.8, 29.8, 29.8, 29.7, 29.7, 29.5, 29.5, 29.4, 28.8, 28.0, 27.9, 26.2, 26.1, 22.8, 22.8, 14.3.

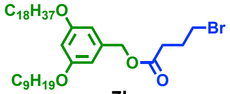

7h

3-(Octadectyloxy)-5-(nonyloxy)benzyl 4-bromobutanoate (7h). Compound **7h** was synthesized from compound **6h** (0.36 g, 0.68 mmol, 1 equiv), 4-bromobutyric acid (0.17 g, 1.04 mmol, 1.5 equiv), DPTS (0.31 g, 1.04 mmol, 1.5 equiv) and DCC (0.43 g, 2.08 mmol, 3 equiv) following a procedure similar to that used for the synthesis of compound **7a**. The title compound was obtained as a colorless oil (0.46 g, 100%). ^{1}H NMR (400 MHz, $CDCl_3$) δ 6.46 (br, 2 H, Ph*H*), 6.40 (t, 1 H, Ph*H*), 5.04 (s, 2 H, $PhCH_2OCO$),

3.93 (t, 4 H, PhOCH_2CH$_2$CH$_2$-), 3.46 (t, 2 H, -OCOCH$_2$CH$_2$CH_2Br), 2.56 (t, 2 H, -OCOCH_2CH$_2$CH$_2$Br), 2.20 (m, 2 H, -OCOCH$_2$CH_2CH$_2$Br), 1.76 (m, 4 H, PhOCH$_2$CH_2CH$_2$-), 1.44 (m, 4 H, PhOCH$_2$CH$_2$CH_2-), 1.28 (m, 38 H, PhOCH$_2$CH$_2$CH$_2$(CH_2)$_{14}$CH$_3$ and PhOCH$_2$CH$_2$CH$_2$(CH_2)$_5$CH$_3$), 0.88 (t, 6 H, PhO(CH$_2$)$_{17}$CH_3 and PhO(CH$_2$)$_8$CH_3). ^{13}C NMR (101 MHz, CDCl$_3$) δ 172.4, 172.4, 160.6, 137.9, 106.5, 101.2, 77.4, 68.2, 66.6, 32.8, 32.6, 32.1, 32.0, 29.8, 29.8, 29.8, 29.7, 29.7, 29.5, 29.5, 29.4, 27.9, 26.2, 22.8, 22.8, 14.3.

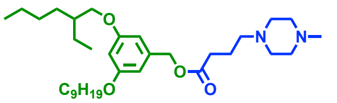

8a (IAJD179)

3-((2-Ethylhexyl)oxy)-5-(nonyloxy)benzyl 4-(4-methylpiperazin-1-yl)butanoate (8a, IAJD179). A mixture of compound **7a** (0.26 g, 0.50 mmol, 1.0 equiv), 1-methylpiperazine (60 mg, 0.60 mmol, 1.2 equiv), K_2CO_3 (83 mg, 0.60 mmol, 1.2 equiv) and MeCN (15 mL) was heated to 95 °C and stirred for 3 h. The reaction mixture was cooled to 23 °C and MeCN was removed under reduced pressure. Then water (20 mL) was added, and the resulted mixture was extracted with DCM (20 mL) for three times. The organic phases were collected and dried over anhydrous $MgSO_4$. After filtration, the obtained filtrate was concentrated to around 3 mL and purified by column chromatography (SiO_2) with DCM/MeOH = 30/1 and 15/1 as the eluent. Then the obtained product was dissolved in DCM (20 mL), which was washed by $NaHCO_3$ solution (2%, 20 mL). The aqueous phase was extracted by DCM (20 mL) for another two times. The organic phases were combined and dried over anhydrous $MgSO_4$. Filtration and evaporation of the solvent yielded the title compound as a light-yellow oil (0.25 g, 95%). ^{1}H NMR (400 MHz, CDCl$_3$) δ 6.46 (br, 2 H, PhH), 6.40 (t, 1 H, PhH), 5.03 (s, 2 H, PhCH_2-), 3.92 (t, 4 H, PhOCH_2-), 3.81 (m, 2 H, PhOCH_2CH(CH$_2$CH$_3$)-), 2.70–2.16 (m, 15 H, -N(CH_2CH_2)$_2$N-, -NCH_2CH$_2$CH_2COO- and -NCH_3), 1.82 (m, 2 H, -OCOCH$_2$CH_2CH$_2$-), 1.79–1.64 (m, 3 H, PhOCH$_2$CH_2(CH$_2$)$_6$CH$_3$ and PhOCH$_2$CH(CH$_2$CH$_3$)(CH$_2$)$_3$CH$_3$), 1.55–1.14 (m, 20 H, PhOCH$_2$CH$_2$(CH_2)$_6$CH$_3$ and PhOCH$_2$CH(CH_2CH$_3$)(CH_2)$_3$CH$_3$), 0.88 (m, 9 H, PhO(CH$_2$)$_8$CH_3 and PhOCH$_2$CH(CH$_2$CH_3)(CH$_2$)$_3$CH_3). ^{13}C NMR (101 MHz, CDCl$_3$) δ 173.5, 160.7, 160.6, 138.2, 106.6, 106.4, 101.0, 70.7, 68.2, 66.3, 57.7, 55.3, 53.3, 46.2, 39.5, 32.4, 32.0, 30.7, 29.8, 29.7, 29.5, 29.4, 29.2, 26.2, 24.0, 23.4, 22.8, 22.4, 14.2, 14.2, 11.2. Purity by HPLC: 99+%. MALDI-TOF MS m/z of $[M + H]^+$ calculated for $C_{33}H_{59}N_2O_4$: 547.4; Found: 547.6.

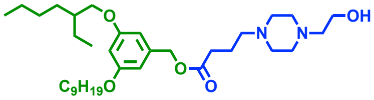

8b (IAJD180)

3-((2-Ethylhexyl)oxy)-5-(nonyloxy)benzyl 4-(4-(2-hydroxyethyl)piperazin-1-yl)butanoate (8b, IAJD180). Compound **8b** was synthesized from compound **7a** (0.26 g, 0.50 mmol), 1-(2-hydroxyethyl)piperazine (78 mg, 0.60 mmol) and K_2CO_3 (83 mg, 0.60 mmol) following a procedure similar to that used for the synthesis of compound **8a**. The title compound was obtained as a colorless oil (0.21 g, 72%). ^1H NMR (400 MHz, $CDCl_3$) δ 6.46 (br, 2 H, Ph*H*), 6.40 (t, 1 H, Ph*H*), 5.03 (s, 2 H, PhCH_2-), 3.92 (t, 4 H, PhOCH_2-), 3.81 (m, 2 H, PhOCH_2CH(CH_2CH_3)-), 3.60 (t, 2 H, -CH_2CH_2OH), 2.82–2.16 (m, 14 H, -N(CH_2CH_2)$_2$N-, -NCH_2CH$_2$CH_2COO- and -NCH_2-), 1.83 (m, 2 H, -OCOCH$_2$CH_2CH$_2$-), 1.74 (m, 3 H, PhOCH$_2$CH_2(CH$_2$)$_6$CH$_3$ and PhOCH$_2$C*H*(CH$_2$CH$_3$)(CH$_2$)$_3$CH$_3$), 1.56–1.17 (m, 20 H, PhOCH$_2$CH$_2$(CH_2)$_6$CH$_3$ and PhOCH$_2$CH(CH_2CH$_3$)(CH_2)$_3$CH$_3$), 0.88 (m, 9 H, PhO(CH$_2$)$_8$CH_3 and PhOCH$_2$CH(CH$_2$CH_3)(CH$_2$)$_3$CH_3). ^{13}C NMR (101 MHz, $CDCl_3$) δ 173.5, 160.7, 160.6, 138.2, 106.6, 106.5, 101.0, 70.7, 68.2, 66.3, 59.3, 57.8, 57.7, 53.3, 53.0, 39.5, 32.4, 32.0, 30.7, 29.8, 29.7, 29.5, 29.4, 29.2, 26.2, 24.0, 23.2, 22.8, 22.3, 14.2, 14.2, 11.3. Purity by HPLC: 99+%. MALDI-TOF MS m/z of $[M + H]^+$ calculated for $C_{34}H_{61}N_2O_5$: 577.5; Found: 577.2.

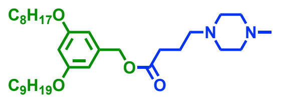

8c (IAJD181)

3-(Nonyloxy)-5-(octyloxy)benzyl 4-(4-methylpiperazin-1-yl)butanoate (8c, IAJD181). Compound **8c** was synthesized from compound **7b** (0.30 g, 0.57 mmol), 1-methylpiperazine (85 mg, 0.61 mmol) and K_2CO_3 (95 mg, 0.68 mmol) following a procedure similar to that used for the synthesis of compound **8a**. The title compound was obtained as a colorless oil (0.28 g, 90%). ^1H NMR (400 MHz, $CDCl_3$) δ 6.45 (br, 2 H, Ph*H*), 6.38 (t, 1 H, Ph*H*), 5.02 (s, 2 H, PhCH_2-), 3.91 (t, 4 H, PhOCH_2-), 2.67–2.21 (m, 15 H, -N(CH_2CH_2)$_2$N-, -NCH_2CH$_2$CH_2COO- and -NCH_3), 1.84 (m, 2 H, -OCOCH$_2$CH_2CH$_2$-), 1.74 (m, 4 H, PhOCH$_2$CH_2-), 1.42 (m, 4 H, PhOCH$_2$CH$_2$CH_2-), 1.28 (br, 18 H, PhOCH$_2$CH$_2$CH$_2$(CH_2)$_4$CH$_3$ and PhOCH$_2$CH$_2$CH$_2$(CH_2)$_5$CH$_3$), 0.88 (t, 6 H, -CH$_2$CH$_2$CH_3). ^{13}C NMR (101 MHz, $CDCl_3$) δ 173.3, 160.5, 138.1, 106.4, 100.9, 68.1, 66.1, 57.5, 55.0, 52.8, 45.8, 32.2, 31.9, 31.8, 29.5, 29.4, 29.4, 29.3, 29.2, 26.1, 22.7, 22.1, 14.1. Purity by HPLC: 99+%. MALDI-TOF MS m/z of $[M + H]^+$ calculated for $C_{33}H_{59}N_2O_4$: 547.4; Found: 548.5.

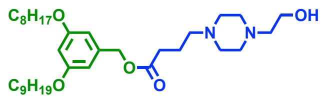

8d (IAJD182)

3-(Nonyloxy)-5-(octyloxy)benzyl 4-(4-(2-hydroxyethyl)piperazin-1-yl)butanoate (8d, IAJD182). Compound **8d** was synthesized from compound **7b** (0.38 g, 0.72 mmol), 1-(2-hydroxyethyl)piperazine

(141 mg, 1.08 mmol) and K_2CO_3 (119 mg, 0.86 mmol) following a procedure similar to that used for the synthesis of compound **8a**. The title compound was obtained as a colorless oil (0.30 g, 72%). ^{1}H NMR (400 MHz, $CDCl_3$) δ 6.46 (br, 2 H, Ph*H*), 6.39 (t, 1 H, Ph*H*), 5.02 (s, 2 H, PhC*H*$_2$-), 3.91 (t, 4 H, PhOC*H*$_2$-), 3.61 (t, 2H, -CH_2CH_2OH), 2.62–2.31 (m, 14 H, -N(CH_2CH_2)$_2$N-, -N$CH_2CH_2CH_2$COO- and -CH_2CH_2OH), 1.84 (m, 2 H, -OCO$CH_2CH_2CH_2$-), 1.75 (m, 4 H, PhOCH_2CH_2-), 1.43 (m, 4 H, PhO$CH_2CH_2CH_2$-), 1.28 (br, 18 H, PhO$CH_2CH_2CH_2(CH_2)_4CH_3$ and PhO$CH_2CH_2CH_2(CH_2)_5CH_3$), 0.89 (t, 6 H, -$CH_2CH_2CH_3$). ^{13}C NMR (101 MHz, $CDCl_3$) δ 173.3, 160.5, 138.1, 106.4, 100.9, 77.2, 68.1, 66.2, 59.3, 57.6, 57.5, 53.0, 52.8, 32.2, 31.9, 31.8, 29.5, 29.4, 29.4, 29.3, 29.2, 26.1, 22.7, 22.1, 14.1. Purity by HPLC: 99+%. MALDI-TOF MS m/z of $[M + H]^+$ calculated for $C_{34}H_{61}N_2O_5$: 577.5; Found: 578.3.

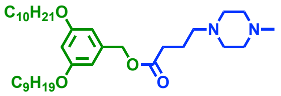

8e (IAJD183)

3-(Decyloxy)-5-(nonyloxy)benzyl 4-(4-methylpiperazin-1-yl)butanoate (8e, IAJD183). Compound **8e** was synthesized from compound **7c** (0.30 g, 0.54 mmol), 1-methylpiperazine (81 mg, 0.81 mmol) and K_2CO_3 (90 mg, 0.68 mmol) following a procedure similar to that used for the synthesis of compound **8a**. The title compound was obtained as a colorless (0.30 g, 97%). ^{1}H NMR (400 MHz, $CDCl_3$) δ 6.44 (br, 2 H, Ph*H*), 6.37 (t, 1 H, Ph*H*), 5.01 (s, 2 H, PhC*H*$_2$-), 3.90 (t, 4 H, PhOC*H*$_2$-), 2.67–2.21 (m, 15 H, -N(CH_2CH_2)$_2$N-, -N$CH_2CH_2CH_2$COO- and -NCH_3), 1.82 (m, 2 H, -OCO$CH_2CH_2CH_2$-), 1.74 (m, 4 H, PhOCH_2CH_2-), 1.42 (m, 4 H, PhO$CH_2CH_2CH_2$-), 1.28 (br, 22 H, PhO$CH_2CH_2CH_2(CH_2)_6CH_3$ and PhO$CH_2CH_2CH_2(CH_2)_5CH_3$), 0.86 (t, 6 H, -$CH_2CH_2CH_3$). ^{13}C NMR (101 MHz, $CDCl_3$) δ 173.2, 160.4, 138.0, 106.4, 100.9, 77.4, 77.1, 76.8, 68.0, 66.1, 59.5, 57.7, 57.5, 52.9, 32.1, 31.9, 31.9, 29.6, 29.5, 29.5, 29.4, 29.3, 29.2, 26.0, 22.7, 22.1, 14.1. Purity by HPLC: 99+%. MALDI-TOF MS m/z of $[M + H]^+$ calculated for $C_{35}H_{63}N_2O_4$: 575.5; Found: 576.2.

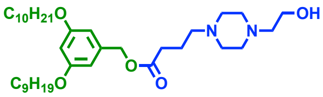

8f (IAJD184)

3-(Decyloxy)-5-(nonyloxy)benzyl 4-(4-(2-hydroxyethyl)piperazin-1-yl)butanoate (8f, IAJD184). Compound **8f** was synthesized from compound **7c** (0.30 g, 0.54 mmol), 1-(2-hydroxyethyl)piperazine (105 mg, 0.81 mmol) and K_2CO_3 (90 mg, 0.65 mmol) following a procedure similar to that used for the synthesis of compound **8a**. The title compound was obtained as a colorless oil (0.30 g, 95%). ^{1}H NMR (400 MHz, $CDCl_3$) δ 6.46 (br, 2 H, Ph*H*), 6.39 (t, 1 H, Ph*H*), 5.02 (s, 2 H, PhC*H*$_2$-), 3.91 (t, 4 H, PhOC*H*$_2$-), 3.61 (t, 2 H, -CH_2CH_2OH), 2.65–2.25 (m, 14 H, -N(CH_2CH_2)$_2$N-, -N$CH_2CH_2CH_2$COO- and -CH_2CH_2OH),

1.84 (m, 2 H, -OCOCH$_2$CH_2CH$_2$-), 1.75 (m, 4 H, PhOCH$_2$CH_2-), 1.43 (m, 4 H, PhOCH$_2$CH$_2$CH_2-), 1.28 (br, 22 H, PhOCH$_2$CH$_2$CH$_2$(CH_2)$_6$CH$_3$ and PhOCH$_2$CH$_2$CH$_2$(CH_2)$_5$CH$_3$), 0.87 (t, 6 H, -CH$_2$CH$_2$CH_3). ^{13}C NMR (101 MHz, CDCl$_3$) δ 173.3, 160.4, 138.1, 106.3, 100.8, 68.0, 66.1, 57.4, 54.9, 52.7, 45.7, 32.1, 31.9, 31.8, 29.5, 29.5, 29.5, 29.4, 29.3, 29.2, 26.0, 22.6, 22.0, 14.1. Purity by HPLC: 99+%. MALDI-TOF MS m/z of [M + H]$^+$ calculated for $C_{36}H_{65}N_2O_5$: 605.5; Found: 606.2.

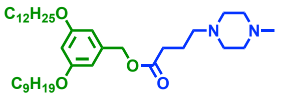

8g (IAJD185)

3-(Dodecyloxy)-5-(nonyloxy)benzyl 4-(4-methylpiperazin-1-yl)butanoate (8g, IAJD185). Compound **8g** was synthesized from compound **7d** (0.37 g, 0.63 mmol), 1-methylpiperazine (68 mg, 0.68 mmol) and K_2CO_3 (128 mg, 0.92 mmol) following a procedure similar to that used for the synthesis of compound **8a**. The title compound was obtained as a colorless (0.35 g, 92%). ^{1}H NMR (400 MHz, CDCl$_3$) δ 6.46 (br, 2 H, PhH), 6.39 (t, 1 H, PhH), 5.02 (s, 2 H, PhCH_2-), 3.91 (t, 4 H, PhOCH_2-), 2.63–2.25 (m, 15 H, -N(CH_2CH_2)$_2$N-, -NCH_2CH$_2$CH_2COO- and -NCH_3), 1.84 (m, 2 H, -OCOCH$_2$CH_2CH$_2$-), 1.74 (m, 4 H, PhOCH$_2$CH_2-), 1.44 (m, 4 H, PhOCH$_2$CH$_2$CH_2-), 1.28 (br, 26 H, PhOCH$_2$CH$_2$CH$_2$(CH_2)$_8$CH$_3$ and PhOCH$_2$CH$_2$CH$_2$(CH_2)$_5$CH$_3$), 0.88 (t, 6 H, -CH$_2$CH$_2$CH_3). ^{13}C NMR (101 MHz, CDCl$_3$) δ 173.3, 160.5, 138.1, 106.4, 100.9, 77.2, 68.1, 66.2, 57.5, 55.0, 52.8, 45.8, 32.2, 31.9, 31.9, 29.7, 29.6, 29.6, 29.6, 29.5, 29.4, 29.4, 29.3, 26.1, 22.7, 22.7, 22.1, 14.1. Purity by HPLC: 99+%. MALDI-TOF MS m/z of [M + H]$^+$ calculated for $C_{37}H_{67}N_2O_4$: 603.5; Found: 604.4.

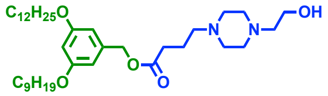

8h (IAJD186)

3-(Dodecyloxy)-5-(nonyloxy)benzyl 4-(4-(2-hydroxyethyl)piperazin-1-yl)butanoate (8h, IAJD186). Compound **8h** was synthesized from compound **7d** (0.30 g, 0.51 mmol), 1-(2-hydroxyethyl)piperazine (73 mg, 0.56 mmol) and K_2CO_3 (90 mg, 0.65 mmol) following a procedure similar to that used for the synthesis of compound **8a**. The title compound was obtained as a colorless oil (0.29 g, 91%). ^{1}H NMR (400 MHz, CDCl$_3$) δ 6.47 (br, 2 H, PhH), 6.39 (t, 1 H, PhH), 5.02 (s, 2 H, PhCH_2-), 3.91 (t, 4 H, PhOCH_2-), 3.61 (t, 2 H, -CH$_2$CH_2OH), 2.68–2.21 (m, 14 H, -N(CH_2CH_2)$_2$N-, -NCH_2CH$_2$CH_2COO- and -CH_2CH$_2$OH), 1.84 (m, 2 H, -OCOCH$_2$CH_2CH$_2$-), 1.76 (m, 4 H, PhOCH$_2$CH_2-), 1.44 (m, 4 H, PhOCH$_2$CH$_2$CH_2-), 1.28 (br, 26 H, PhOCH$_2$CH$_2$CH$_2$(CH_2)$_8$CH$_3$ and PhOCH$_2$CH$_2$CH$_2$(CH_2)$_5$CH$_3$), 0.88 (t, 6 H, -CH$_2$CH$_2$CH_3). ^{13}C NMR (101 MHz, CDCl$_3$) δ 173.3, 160.4, 138.1, 106.3, 100.9, 68.0, 66.1, 57.4, 54.9, 52.8, 45.7, 32.1, 31.9,

31.7, 29.6, 29.5, 29.4, 29.4, 29.4, 29.2, 26.0, 22.6, 22.0, 14.2. Purity by HPLC: 99+%. MALDI-TOF MS m/z of $[M + H]^+$ calculated for $C_{38}H_{69}N_2O_5$: 633.5; Found: 634.1.

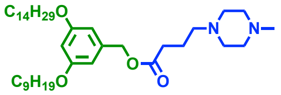

8i (IAJD187)

3-(Nonyloxy)-5-(tetradecyloxy)benzyl 4-(4-methylpiperazin-1-yl)butanoate (8i, IAJD187). Compound **8i** was synthesized from compound **7e** (0.22 g, 0.35 mmol), 1-methylpiperazine (53 mg, 0.53 mmol) and K_2CO_3 (58 mg, 0.42 mmol) following a procedure similar to that used for the synthesis of compound **8a**. The title compound was obtained as a colorless (0.10 g, 46%). ^{1}H NMR (400 MHz, $CDCl_3$) δ 6.48 (br, 2 H, Ph*H*), 6.40 (t, 1 H, Ph*H*), 5.04 (s, 2 H, $PhCH_2$-), 3.93 (t, 4 H, $PhOCH_2$-), 2.90–2.18 (m, 15 H, -$N(CH_2CH_2)_2N$-, -$NCH_2CH_2CH_2COO$- and -NCH_3), 1.85 (m, 2 H, -$OCOCH_2CH_2CH_2$-), 1.76 (m, 4 H, $PhOCH_2CH_2$-), 1.44 (m, 4 H, $PhOCH_2CH_2CH_2$-), 1.29 (br, 30 H, $PhOCH_2CH_2CH_2(CH_2)_{10}CH_3$ and $PhOCH_2CH_2CH_2(CH_2)_5CH_3$), 0.86 (t, 6 H, -$CH_2CH_2CH_3$). ^{13}C NMR (101 MHz, $CDCl_3$) δ 173.3, 160.5, 138.1, 106.4, 100.9, 68.1, 66.2, 57.4, 54.9, 52.6, 45.7, 32.2, 31.9, 31.9, 29.6, 29.6, 29.4, 29.4, 29.3, 29.3, 29.2, 26.1, 22.7, 22.7, 22.1, 14.1. Purity by HPLC: 99+%. MALDI-TOF MS m/z of $[M + H]^+$ calculated for $C_{39}H_{71}N_2O_4$: 631.5; Found: 632.0.

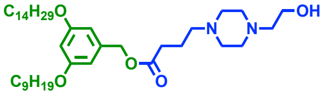

8j (IAJD188)

3-(Nonyloxy)-5-(tetradecyloxy)benzyl 4-(4-(2-hydroxyethyl)piperazin-1-yl)butanoate (8j, IAJD188). Compound **8j** was synthesized from compound **7e** (0.18 g, 0.30 mmol), 1-(2-hydroxyethyl)piperazine (58 mg, 0.44 mmol) and K_2CO_3 (49 mg, 0.36 mmol) following a procedure similar to that used for the synthesis of compound **8a**. The title compound was obtained as a colorless oil (0.15 g, 80%). ^{1}H NMR (400 MHz, $CDCl_3$) δ 6.47 (br, 2 H, Ph*H*), 6.41 (t, 1 H, Ph*H*), 5.04 (s, 2 H, $PhCH_2$-), 3.93 (t, 4 H, $PhOCH_2$-), 3.64 (t, 2 H, -CH_2CH_2OH), 2.59–2.37 (m, 14 H, -$N(CH_2CH_2)_2N$-, -$NCH_2CH_2CH_2COO$- and -CH_2CH_2OH), 1.84 (m, 2 H, -$OCOCH_2CH_2CH_2$-), 1.76 (m, 4 H, $PhOCH_2CH_2$-), 1.44 (m, 4 H, $PhOCH_2CH_2CH_2$-), 1.28 (br, 30 H, $PhOCH_2CH_2CH_2(CH_2)_{10}CH_3$ and $PhOCH_2CH_2CH_2(CH_2)_5CH_3$), 0.88 (t, 6 H, -$CH_2CH_2CH_3$). ^{13}C NMR (101 MHz, $CDCl_3$) δ 173.3, 160.4, 138.1, 106.3, 100.9, 68.0, 66.1, 57.4, 54.9, 52.8, 45.7, 32.1, 31.9, 31.7, 29.6, 29.5, 29.4, 29.4, 29.4, 29.2, 26.0, 22.6, 22.0, 14.2. Purity by HPLC: 99+%. MALDI-TOF MS m/z of $[M + H]^+$ calculated for $C_{40}H_{73}N_2O_5$: 661.5; Found: 662.4.

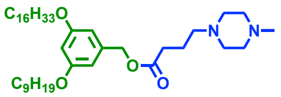

8k (IAJD189)

3-(Hexadecyloxy)-5-(nonyloxy)benzyl 4-(4-methylpiperazin-1-yl)butanoate (8k, IAJD189). Compound **8k** was synthesized from compound **7f** (0.27 g, 0.41 mmol), 1-methylpiperazine (46 mg, 0.46 mmol) and K_2CO_3 (86 mg, 0.62 mmol) following a procedure similar to that used for the synthesis of compound **8a**. The title compound was obtained as a colorless (0.26 g, 95%). ^{1}H NMR (400 MHz, $CDCl_3$) δ 6.45 (br, 2 H, Ph*H*), 6.38 (t, 1 H, Ph*H*), 5.02 (s, 2 H, PhC*H*$_2$-), 3.91 (t, 4 H, PhOC*H*$_2$-), 2.62–2.26 (m, 15 H, -N(C*H*$_2$C*H*$_2$)$_2$N-, -NC*H*$_2$CH$_2$C*H*$_2$COO- and -NC*H*$_3$), 1.84 (m, 2 H, -OCOCH$_2$C*H*$_2$CH$_2$-), 1.75 (m, 4 H, PhOCH$_2$C*H*$_2$-), 1.44 (m, 4 H, PhOCH$_2$CH$_2$C*H*$_2$-), 1.28 (br, 34 H, PhOCH$_2$CH$_2$CH$_2$(C*H*$_2$)$_{12}$CH$_3$ and PhOCH$_2$CH$_2$CH$_2$(C*H*$_2$)$_5$CH$_3$), 0.87 (t, 6 H, -CH$_2$CH$_2$C*H*$_3$). ^{13}C NMR (101 MHz, $CDCl_3$) δ 173.3, 160.5, 138.1, 106.4, 100.9, 68.1, 66.1, 64.6, 57.5, 57.4, 54.9, 54.9, 52.7, 52.6, 45.7, 32.2, 31.9, 31.9, 29.7, 29.7, 29.6, 29.6, 29.5, 29.4, 29.4, 29.3, 28.7, 26.1, 26.0, 22.7, 22.1, 14.1. Purity by HPLC: 99+%. MALDI-TOF MS m/z of $[M + H]^+$ calculated for $C_{41}H_{75}N_2O_4$: 659.6; Found: 660.0.

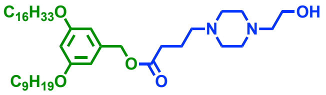

8l (IAJD190)

3-(Hexadecyloxy)-5-(nonyloxy)benzyl 4-(4-(2-hydroxyethyl)piperazin-1-yl)butanoate (8l, IAJD190). Compound **8l** was synthesized from compound **7f** (0.25 g, 0.39 mmol), 1-(2-hydroxyethyl)piperazine (77 mg, 0.59 mmol) and K_2CO_3 (65 mg, 0.47 mmol) following a procedure similar to that used for the synthesis of compound **8a**. The title compound was obtained as a colorless oil (0.24 g, 89%). ^{1}H NMR (400 MHz, $CDCl_3$) δ 6.45 (br, 2 H, Ph*H*), 6.38 (t, 1 H, Ph*H*), 5.01 (s, 2 H, PhC*H*$_2$-), 3.90 (t, 4 H, PhOC*H*$_2$-), 3.60 (t, 2H, -CH$_2$C*H*$_2$OH), 2.63–2.26 (m, 14 H, -N(C*H*$_2$C*H*$_2$)$_2$N-, -NC*H*$_2$CH$_2$C*H*$_2$COO- and -C*H*$_2$CH$_2$OH), 1.83 (m, 2 H, -OCOCH$_2$C*H*$_2$CH$_2$-), 1.74 (m, 4 H, PhOCH$_2$C*H*$_2$-), 1.43 (m, 4 H, PhOCH$_2$CH$_2$C*H*$_2$-), 1.25 (br, 34 H, PhOCH$_2$CH$_2$CH$_2$(C*H*$_2$)$_{12}$CH$_3$ and PhOCH$_2$CH$_2$CH$_2$(C*H*$_2$)$_5$CH$_3$), 0.87 (t, 6 H, -CH$_2$CH$_2$C*H*$_3$). ^{13}C NMR (101 MHz, $CDCl_3$) δ 173.4, 160.4, 138.1, 106.3, 100.8, 68.0, 66.1, 57.4, 54.9, 52.7, 45.7, 32.1, 31.9, 31.8, 29.5, 29.5, 29.5, 29.4, 29.3, 29.2, 26.0, 22.7, 22.1, 14.2. Purity by HPLC: 99+%. MALDI-TOF MS m/z of $[M + H]^+$ calculated for $C_{42}H_{77}N_2O_5$: 689.6; Found: 689.9.

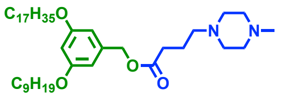

8m (IAJD191)

3-(Heptadecyloxy)-5-(nonyloxy)benzyl 4-(4-methylpiperazin-1-yl)butanoate (8m, IAJD191). Compound **8m** was synthesized from compound **7g** (0.26 g, 0.40 mmol), 1-methylpiperazine (42 mg, 0.42 mmol) and K_2CO_3 (58 mg, 0.42 mmol) following a procedure similar to that used for the synthesis of compound **8a**. The title compound was obtained as a colorless (0.22 g, 82%). ^{1}H NMR (400 MHz, $CDCl_3$) δ 6.44 (br, 2 H, Ph*H*), 6.37 (t, 1 H, Ph*H*), 5.01 (s, 2 H, PhC*H*$_2$-), 3.90 (t, 4 H, PhOC*H*$_2$-), 2.54–2.20 (m, 15 H, -N(C*H*$_2$C*H*$_2$)$_2$N-, -NC*H*$_2$CH$_2$C*H*$_2$COO- and -NC*H*$_3$), 1.82 (m, 2 H, -OCOCH$_2$C*H*$_2$CH$_2$-), 1.73 (m, 4 H, PhOCH$_2$C*H*$_2$-), 1.42 (m, 4 H, PhOCH$_2$CH$_2$C*H*$_2$-), 1.26 (br, 36 H, PhOCH$_2$CH$_2$CH$_2$(C*H*$_2$)$_{13}$CH$_3$ and PhOCH$_2$CH$_2$CH$_2$(C*H*$_2$)$_5$CH$_3$), 0.87 (t, 6 H, -CH$_2$CH$_2$C*H*$_3$). ^{13}C NMR (101 MHz, $CDCl_3$) δ 173.3, 173.3, 160.4, 138.1, 106.4, 100.9, 68.0, 66.1, 57.6, 55.2, 53.1, 46.1, 32.2, 31.9, 31.9, 29.7, 29.7, 29.6, 29.6, 29.5, 29.4, 29.4, 29.3, 26.1, 22.7, 22.7, 22.2, 14.1. Purity by HPLC: 99+%. MALDI-TOF MS m/z of $[M + H]^+$ calculated for $C_{42}H_{77}N_2O_4$: 673.6; Found: 674.1.

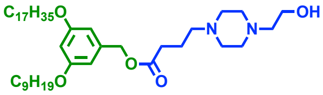

8n (IAJD192)

3-(Heptadecyloxy)-5-(nonyloxy)benzyl 4-(4-(2-hydroxyethyl)piperazin-1-yl)butanoate (8n, IAJD192). Compound **8n** was synthesized from compound **7g** (0.26 g, 0.40 mmol), 1-(2-hydroxyethyl)piperazine (55 mg, 0.42 mmol) and K_2CO_3 (58 mg, 0.42 mmol) following a procedure similar to that used for the synthesis of compound **8a**. The title compound was obtained as a colorless oil (0.24 g, 86%). ^{1}H NMR (400 MHz, $CDCl_3$) δ 6.45 (br, 2 H, Ph*H*), 6.39(t, 1 H, Ph*H*), 5.02 (s, 2 H, PhC*H*$_2$-), 3.91 (t, 4 H, PhOC*H*$_2$-), 3.59 (t, 2H, -CH$_2$C*H*$_2$OH), 2.60–2.28 (m, 14 H, -N(C*H*$_2$C*H*$_2$)$_2$N-, -NC*H*$_2$CH$_2$C*H*$_2$COO- and -C*H*$_2$CH$_2$OH), 1.84 (m, 2 H, -OCOCH$_2$C*H*$_2$CH$_2$-), 1.74 (m, 4 H, PhOCH$_2$C*H*$_2$-), 1.42 (m, 4 H, PhOCH$_2$CH$_2$C*H*$_2$-), 1.28 (br, 36 H, PhOCH$_2$CH$_2$CH$_2$(C*H*$_2$)$_{13}$CH$_3$ and PhOCH$_2$CH$_2$CH$_2$(C*H*$_2$)$_5$CH$_3$), 0.87 (t, 6 H, -CH$_2$CH$_2$C*H*$_3$). ^{13}C NMR (101 MHz, $CDCl_3$) δ 173.3, 160.5, 138.1, 106.4, 100.9, 68.1, 66.1, 59.2, 57.7, 57.6, 53.2, 52.9, 32.2, 31.9, 31.9, 29.7, 29.7, 29.6, 29.6, 29.5, 29.4, 29.4, 29.3, 26.1, 22.7, 22.7, 22.2, 14.1. Purity by HPLC: 99+%. MALDI-TOF MS m/z of $[M + H]^+$ calculated for $C_{43}H_{79}N_2O_5$: 703.6; Found: 703.7.

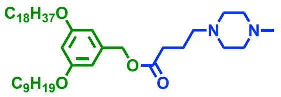

8o (IAJD193)

3-(Nonyloxy)-5-(octadecyloxy)benzyl 4-(4-methylpiperazin-1-yl)butanoate (8o, IAJD193). Compound **8o** was synthesized from compound **7h** (0.23 g, 0.35 mmol), 1-methylpiperazine (42 mg, 0.42 mmol) and K_2CO_3 (58 mg, 0.42 mmol) following a procedure similar to that used for the synthesis of compound **8a**. The title compound was obtained as a colorless (0.20 g, 84%). ^{1}H NMR (400 MHz, $CDCl_3$) δ 6.41 (br, 2 H, Ph*H*), 6.33 (t, 1 H, Ph*H*), 4.97 (s, 2 H, PhCH_2-), 3.85 (t, 4 H, PhOCH_2-), 2.77–2.14 (m, 15 H, -N(CH_2CH_2)$_2$N-, -NCH_2CH$_2$CH_2COO- and -NCH_3), 1.77 (m, 2 H, -OCOCH$_2$CH_2CH$_2$-), 1.69 (m, 4 H, PhOCH$_2$CH_2-), 1.39 (m, 4 H, PhOCH$_2$CH$_2$CH_2-), 1.21 (br, 38 H, PhOCH$_2$CH$_2$CH$_2$(CH_2)$_{14}$CH$_3$ and PhOCH$_2$CH$_2$CH$_2$(CH_2)$_5$CH$_3$), 0.83 (t, 6 H, -CH$_2$CH$_2$CH_3). ^{13}C NMR (101 MHz, $CDCl_3$) δ 173.1, 166.1, 160.4, 138.1, 106.3, 100.8, 77.5, 77.2, 76.9, 68.0, 66.0, 57.4, 55.0, 52.7, 45.8, 32.1, 31.9, 31.9, 29.7, 29.7, 29.6, 29.6, 29.5, 29.4, 29.4, 29.3, 26.0, 22.7, 22.6, 22.1, 14.1. Purity by HPLC: 99+%. MALDI-TOF MS m/z of $[M + H]^+$ calculated for $C_{43}H_{79}N_2O_4$: 687.6; Found: 688.3.

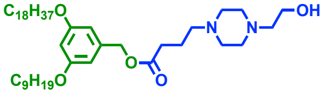

8p (IAJD194)

3-(Nonyloxy)-5-(octadecyloxy)benzyl 4-(4-(2-hydroxyethyl)piperazin-1-yl)butanoate (8p, IAJD194). Compound **8p** was synthesized from compound **7h** (0.23 g, 0.35 mmol), 1-(2-hydroxyethyl)piperazine (55 mg, 0.42 mmol) and K_2CO_3 (58 mg, 0.42 mmol) following a procedure similar to that used for the synthesis of compound **8a**. The title compound was obtained as a colorless oil (0.19 g, 76%). ^{1}H NMR (400 MHz, $CDCl_3$) δ 6.46 (br, 2 H, Ph*H*), 6.38 (t, 1 H, Ph*H*), 5.01 (s, 2 H, PhCH_2-), 3.90 (t, 4 H, PhOCH_2-), 3.61 (t, 2H, -CH$_2$CH_2OH), 2.74–2.12 (m, 14 H, -N(CH_2CH_2)$_2$N-, -NCH_2CH$_2$CH_2COO- and -CH_2CH$_2$OH), 1.82 (m, 2 H, -OCOCH$_2$CH_2CH$_2$-), 1.75 (m, 4 H, PhOCH$_2$CH_2-), 1.43 (m, 4 H, PhOCH$_2$CH$_2$CH_2-), 1.25 (br, 38 H, PhOCH$_2$CH$_2$CH$_2$(CH_2)$_{14}$CH$_3$ and PhOCH$_2$CH$_2$CH$_2$(CH_2)$_5$CH$_3$), 0.86 (t, 6 H, -CH$_2$CH$_2$CH_3). ^{13}C NMR (101 MHz, $CDCl_3$) δ 173.3, 160.5, 138.1, 106.5, 101.0, 68.2, 66.2, 59.5, 57.7, 57.6, 53.0, 53.0, 32.3, 32.0, 32.0, 29.8, 29.8, 29.7, 29.7, 29.6, 29.5, 29.4, 29.4, 26.1, 22.8, 22.8, 22.2, 14.2. Purity by HPLC: 99+%. MALDI-TOF MS m/z of $[M + H]^+$ calculated for $C_{44}H_{81}N_2O_5$: 717.6; Found: 718.7.

3.2 Synthesis of Dodecane-Based IAJDs with 3,5-Nonsymmetric Alkyl Chains

Scheme S2. Synthesis of Dodecane-Based IAJDs with 3,5-Nonsymmetric Alkyl Chains

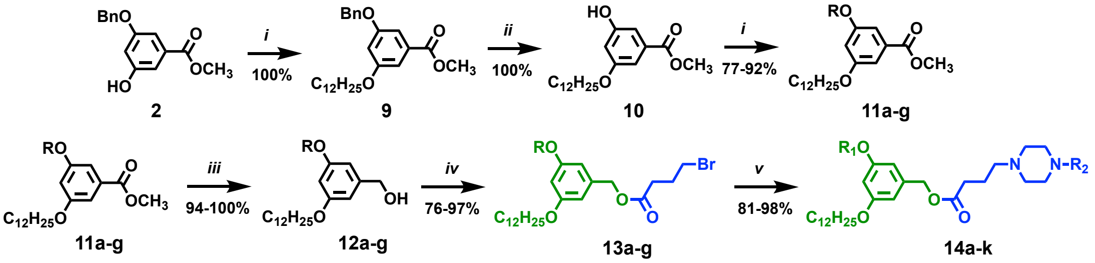

11-13a: R = 2-ethylhexyl
11-13b: R = C_8H_{17}
11-13c: R = $C_{10}H_{21}$
11-13d: R = $C_{14}H_{29}$
11-13e: R = $C_{16}H_{33}$
11-13f: R = $C_{17}H_{35}$
11-13g: R = $C_{18}H_{37}$

14a (IAJD197): R_1 = 2-ethylhexyl; R_2 = CH_3
14b (IAJD198): R_1 = 2-ethylhexyl; R_2 = CH_2CH_2OH
14c (IAJD199): R_1 = C_8H_{17}; R_2 = CH_3
14d (IAJD200): R_1 = C_8H_{17}; R_2 = CH_2CH_2OH
14e (IAJD201): R_1 = $C_{10}H_{21}$; R_2 = CH_2CH_2OH
14f (IAJD202): R_1 = $C_{14}H_{29}$; R_2 = CH_3
14g (IAJD203): R_1 = $C_{14}H_{29}$; R_2 = CH_2CH_2OH
14h (IAJD204): R_1 = $C_{16}H_{33}$; R_2 = CH_2CH_2OH
14i (IAJD205): R_1 = $C_{17}H_{35}$; R_2 = CH_3
14j (IAJD206): R_1 = $C_{17}H_{35}$; R_2 = CH_2CH_2OH
14k (IAJD207): R_1 = $C_{18}H_{37}$; R_2 = CH_2CH_2OH

Reagents and conditions: (*i*) RBr/ROTs, K_2CO_3, DMF, 120 °C, 2 h; (*ii*) H_2, Pd/C, DCM, MeOH, 12 h; (*iii*) $LiAlH_4$, THF, 0–23 °C, 1 h; (*iv*) DCC, DPTS, DCM, 12 h; (*v*) K_2CO_3, MeCN, 95 °C, 3 h.

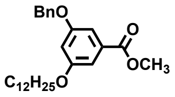

9

Methyl 3-(benzyloxy)-5-(dodecyloxy)benzoate (9). A mixture of compound **2** (5.50 g, 21.30 mmol, 1 equiv), 1-bromododecane (5.84 g, 23.43 mmol, 1.1 equiv), K_2CO_3 (5.89 g, 42.60 mmol, 2 equiv) and DMF (20 mL) was heated to 120 °C and stirred under N_2 atmosphere for 2 h. The reaction mixture was cooled to 23 °C and poured into ice/water (150 mL). The resulted white precipitates in the solution were filtered and collected. Then the precipitates were recrystallized from minimum acetone to afford the title compound as a white solid (6.53 g, 74%). ^{1}H NMR (400 MHz, $CDCl_3$) δ 7.49–7.31 (m, 5 H, Ph*H*), 7.27 (br, 1 H, Ph*H*), 7.20 (br, 1 H, Ph*H*), 6.73 (t, 1 H, Ph*H*), 5.08 (s, 2 H, PhC*H*$_2$O-), 3.97 (t, 2 H, PhOC*H*$_2$-), 3.90 (s, 3 H, PhCOOC*H*$_3$), 1.78 (m, 2 H, $PhOCH_2CH_2CH_2(CH_2)_8CH_3$), 1.44 (m, 2 H, $PhOCH_2CH_2CH_2(CH_2)_8CH_3$), 1.29 (m, 16 H, $PhOCH_2CH_2CH_2(CH_2)_8CH_3$), 0.89 (t, 3 H, $PhO(CH_2)_{11}CH_3$). ^{13}C NMR (101 MHz, $CDCl_3$) δ 167.0, 160.4, 159.9, 136.7, 132.1, 128.8, 128.2, 127.7, 108.3, 108.0, 107.1, 77.4, 70.4, 68.5, 52.3, 32.1, 29.8, 29.8, 29.7, 29.7, 29.6, 29.5, 29.3, 26.1, 22.8, 14.3. Mp = 36 °C.

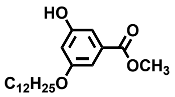

10

Methyl 3-hydroxy-5-(dodecyloxy)benzoate (10). Compound **9** (6.53 g, 15.8 mmol) was dissolved in a mixture of DCM (40 mL) and methanol (20 mL). Then Pd/C (0.34 g, 5 wt%) was added to the solution and the flask was evacuated and filled with hydrogen for three times. Afterwards, the mixture was stirred at 23 °C under hydrogen atmosphere for 12 h. The reaction mixture was filtered through Celite, and the filter cake was washed with DCM. Evaporation of the solvent yielded the title compound as a white solid (5.04 g, 95%). ^{1}H NMR (400 MHz, $CDCl_3$) δ 7.15 (d, 2 H, Ph*H*), 6.62 (t, 1 H, Ph*H*), 5.99 (s, 1 H, PhO*H*), 3.95 (t, 2 H, PhOC*H*$_2$-), 3.90 (s, 3 H, PhCOOC*H*$_3$), 1.74 (m, 2 H, $PhOCH_2CH_2CH_2(CH_2)_8CH_3$), 1.43 (m, 2 H, $PhOCH_2CH_2CH_2(CH_2)_8CH_3$), 1.27 (m, 16 H, $PhOCH_2CH_2CH_2(CH_2)_8CH_3$), 0.88 (t, 3 H, $PhO(CH_2)_{11}CH_3$). ^{13}C NMR (101 MHz, $CDCl_3$) δ 167.4, 160.6, 157.0, 157.0, 132.0, 109.2, 107.9, 107.3, 68.5, 52.5, 34.2, 33.0, 32.1, 29.8, 29.8, 29.7, 29.7, 29.7, 29.6, 29.5, 29.5, 29.3, 28.9, 28.3, 26.1, 22.8, 14.2. Mp = 89 °C.

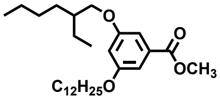

11a

Methyl 3-((2-ethylhexyl)oxy)-5-(dodecyloxy)benzoate (11a). A mixture of compound **10** (0.60 g, 1.78 mmol, 1 equiv), 2-ethylhexyl bromide (0.38 g, 1.96 mmol, 1.1 equiv), K_2CO_3 (0.49 g, 3.56 mmol, 2 equiv) and DMF (15 mL) was heated to 80 °C and stirred under N_2 atmosphere for 12 h. The reaction mixture was cooled to 23 °C and DMF was removed under reduced pressure. Then water (20 mL) was added, and the resulted mixture was extracted with DCM (20 mL) for three times. The organic phases were collected and dried over anhydrous $MgSO_4$. After filtration, the obtained filtrate was concentrated to around 3 mL and purified by column chromatography (SiO_2) with hexane/EtOAc = 20/1 as the eluent to give the title compound as a light-yellow oil (0.55 g, 69%). ^{1}H NMR (400 MHz, $CDCl_3$) δ 7.16 (br, 2 H, Ph*H*), 6.62 (t, 1 H, Ph*H*), 3.97 (t, 2 H, $PhOCH_2(CH_2)_{13}CH_3$), 3.89 (s, 3 H, PhCOOC*H*$_3$), 3.85 (m, 2 H, $PhOCH_2CH(CH_2CH_3)$-), 1.86–1.65 (m, 3 H, $PhOCH_2CH_2(CH_2)_9CH_3$ and $PhOCH_2CH(CH_2CH_3)(CH_2)_3CH_3$), 1.57–1.18 (m, 26 H, $PhOCH_2CH_2(CH_2)_9CH_3$ and $PhOCH_2CH(CH_2CH_3)(CH_2)_3CH_3$), 1.00–0.82 (m, 9 H, $PhO(CH_2)_{11}CH_3$ and $PhOCH_2CH(CH_2CH_3)(CH_2)_3CH_3$). ^{13}C NMR (101 MHz, $CDCl_3$) δ 167.3, 160.6, 160.3, 132.2, 131.9, 107.9, 107.8, 107.6, 106.7, 70.9, 68.5, 52.4, 52.3, 30.7, 29.8, 29.8, 29.7, 29.7, 29.5, 29.3, 29.2, 26.2, 22.8, 14.2, 14.2, 11.2.

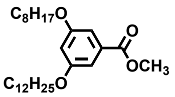

11b

Methyl 3-(octyloxy)-5-(dodectyloxy)benzoate (11b). A mixture of compound **10** (0.60 g, 1.78 mmol, 1 equiv), 1-bromooctane (0.38 g, 1.96 mmol, 1.1 equiv), K_2CO_3 (0.49 g, 3.56 mmol, 2 equiv) and DMF (10 mL) was heated to 120 °C and stirred under N_2 atmosphere for 2 h. The reaction mixture was cooled to 23 °C and poured into ice/water (150 mL). The resulted white precipitates in the solution were filtered and collected. Then the precipitates were recrystallized from minimum acetone to afford the title compound as a white solid (0.43 g, 54%). ^{1}H NMR (400 MHz, $CDCl_3$) δ 7.16 (br, 2 H, Ph*H*), 6.64 (t, 1 H, Ph*H*), 3.96 (t, 4 H, $PhOCH_2CH_2CH_2$-), 3.89 (s, 3 H, $PhCOOCH_3$), 1.77 (m, 4 H, $PhOCH_2CH_2CH_2$-), 1.44 (m, 4 H, $PhOCH_2CH_2CH_2$-), 1.28 (m, 24 H, $PhOCH_2CH_2CH_2(CH_2)_4CH_3$ and $PhOCH_2CH_2CH_2(CH_2)_8CH_3$), 0.88 (t, 6 H, $PhO(CH_2)_7CH_3$ and $PhO(CH_2)_{11}CH_3$). ^{13}C NMR (101 MHz, $CDCl_3$) δ 167.1, 160.3, 132.0, 107.8, 106.7, 68.5, 52.3, 32.1, 32.0, 29.8, 29.8, 29.7, 29.7, 29.5, 29.5, 29.5, 29.4, 29.3, 26.2, 22.8, 22.8, 14.2. Mp = 39 °C.

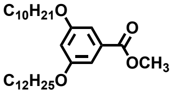

11c

Methyl 3-(decyloxy)-5-(dodecyloxy)benzoate (11c). Compound **11c** was synthesized from compound **10** (0.60 g, 1.78 mmol, 1 equiv), 1-bromodecane (0.43 g, 1.96 mmol, 1.1 equiv) and K_2CO_3 (0.49 g, 3.56 mmol, 2 equiv) following a procedure similar to that used for the synthesis of compound **11b**. The title compound was obtained as a white solid (0.69 g, 81%). ^{1}H NMR (400 MHz, $CDCl_3$) δ 7.16 (br, 2 H, Ph*H*), 6.63 (t, 1 H, Ph*H*), 3.96 (t, 4 H, $PhOCH_2CH_2CH_2$-), 3.89 (s, 3 H, $PhCOOCH_3$), 1.77 (m, 4 H, $PhOCH_2CH_2CH_2$-), 1.45 (m, 4 H, $PhOCH_2CH_2CH_2$-), 1.29 (m, 28 H, $PhOCH_2CH_2CH_2(CH_2)_6CH_3$ and $PhOCH_2CH_2CH_2(CH_2)_8CH_3$), 0.88 (t, 6 H, $PhO(CH_2)_9CH_3$ and $PhO(CH_2)_{11}CH_3$). ^{13}C NMR (101 MHz, $CDCl_3$) δ 167.1, 160.3, 132.0, 107.8, 106.7, 68.5, 52.3, 32.1, 32.0, 29.8, 29.8, 29.7, 29.7, 29.7, 29.5, 29.5, 29.3, 26.2, 22.8, 14.3. Mp = 50 °C.

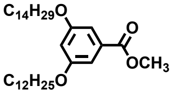

11d

Methyl 3-(dodecyloxy)-5-(tetradecyloxy)benzoate (11d). Compound **11d** was synthesized from Compound **10** (0.60 g, 1.78 mmol, 1 equiv), 1-bromotetradecane (0.54 g, 1.96 mmol, 1.1 equiv) and K_2CO_3 (0.49 g, 3.56 mmol, 2 equiv) following a procedure similar to that used for the synthesis of compound **11b**. The title compound was obtained as a white solid (0.81 g, 85%). ^{1}H NMR (400 MHz,

CDCl3) δ 7.15 (br, 2 H, Ph*H*), 6.63 (t, 1 H, Ph*H*), 3.96 (t, 4 H, PhOCH_2CH$_2$CH$_2$-), 3.89 (s, 3 H, PhCOOCH_3), 1.77 (m, 4 H, PhOCH$_2$CH_2CH$_2$-), 1.45 (m, 4 H, PhOCH$_2$CH$_2$CH_2-), 1.29 (m, 36 H, PhOCH$_2$CH$_2$CH$_2$(CH_2)$_{10}$CH$_3$ and PhOCH$_2$CH$_2$CH$_2$(CH_2)$_8$CH$_3$), 0.89 (t, 6 H, PhO(CH$_2$)$_{13}$CH_3 and PhO(CH$_2$)$_{11}$CH_3). ^{13}C NMR (101 MHz, CDCl$_3$) δ 167.1, 160.3, 132.0, 107.8, 106.7, 77.4, 68.5, 52.3, 32.1, 29.8, 29.8, 29.8, 29.7, 29.7, 29.6, 29.5, 29.3, 26.2, 22.8, 14.2. Mp = 57 °C.

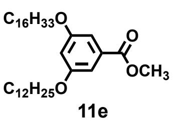

11e

Methyl 3-(dodecyloxy)-5-(hexadecyloxy)benzoate (11e). Compound **11e** was synthesized from compound **10** (0.60 g, 1.78 mmol, 1 equiv), 1-bromohexadecane (0.60 g, 1.96 mmol, 1.1 equiv) and K_2CO_3 (0.49 g, 3.56 mmol, 2 equiv) following a procedure similar to that used for the synthesis of compound **11b**. The title compound was obtained as a white solid (730 g, 73%). ^{1}H NMR (400 MHz, CDCl$_3$) δ 7.16 (br, 2 H, Ph*H*), 6.63 (t, 1 H, Ph*H*), 3.96 (t, 4 H, PhOCH_2CH$_2$CH$_2$-), 3.88 (s, 3 H, PhCOOCH_3), 1.77 (m, 4 H, PhOCH$_2$CH_2CH$_2$-), 1.44 (m, 4 H, PhOCH$_2$CH$_2$CH_2-), 1.25 (m, 40 H, PhOCH$_2$CH$_2$CH$_2$(CH_2)$_{12}$CH$_3$ and PhOCH$_2$CH$_2$CH$_2$(CH_2)$_8$CH$_3$), 0.87 (t, 6 H, PhO(CH$_2$)$_{15}$CH_3 and PhO(CH$_2$)$_{11}$CH_3). ^{13}C NMR (101 MHz, CDCl$_3$) δ 167.0, 160.2, 131.8, 107.6, 106.6, 77.3, 77.0, 76.7, 68.3, 52.2, 31.9, 29.7, 29.7, 29.7, 29.6, 29.6, 29.4, 29.3, 29.2, 26.0, 22.7, 14.1. Mp = 45 °C.

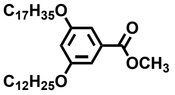

11f

Methyl 3-(dodecyloxy)-5-(heptadecyloxy)benzoate (11f). Compound **11f** was synthesized from compound **10** (0.60 g, 1.78 mmol, 1 equiv), $C_{17}H_{35}$OTs (0.81g, 1.96 mmol, 1.1 equiv) and K_2CO_3 (0.49 g, 3.56 mmol, 2 equiv) following a procedure similar to that used for the synthesis of compound **11b**. The title compound was obtained as a white solid (0.72 g, 70%). ^{1}H NMR (400 MHz, CDCl$_3$) δ 7.16 (br, 2 H, Ph*H*), 6.63 (t, 1 H, Ph*H*), 3.96 (t, 4 H, PhOCH_2CH$_2$CH$_2$-), 3.89 (s, 3 H, PhCOOCH_3), 1.77 (m, 4 H, PhOCH$_2$CH_2CH$_2$-), 1.45 (m, 4 H, PhOCH$_2$CH$_2$CH_2-), 1.29 (m, 42 H, PhOCH$_2$CH$_2$CH$_2$(CH_2)$_{13}$CH$_3$ and PhOCH$_2$CH$_2$CH$_2$(CH_2)$_8$CH$_3$), 0.88 (t, 6 H, PhO(CH$_2$)$_{16}$CH_3 and PhO(CH$_2$)$_{11}$CH_3). ^{13}C NMR (101 MHz, CDCl$_3$) δ 167.1, 160.3, 132.0, 107.8, 106.7, 77.4, 68.5, 68.2, 52.3, 51.9, 32.1, 29.8, 29.8, 29.8, 29.7, 29.7, 29.7, 29.6, 29.5, 29.5, 29.3, 26.2, 22.8, 14.3. Mp = 60 °C.

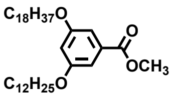

11g

Methyl 3-(dodecyloxy)-5-(octadecyloxy)benzoate (11g). Compound **11g** was synthesized from compound **10** (0.60 g, 1.78 mmol, 1 equiv), 1-bromooctadecane (0.63 g, 1.96 mmol, 1.1 equiv) and K_2CO_3 (0.49 g, 3.56 mmol, 2 equiv) following a procedure similar to that used for the synthesis of compound **11b**. The title compound was obtained as a white solid (0.78 g, 76%). ^1H NMR (400 MHz, $CDCl_3$) δ 7.15 (br, 2 H, Ph*H*), 6.63 (t, 1 H, Ph*H*), 3.97 (t, 4 H, $PhOCH_2$ CH_2CH_2-), 3.89 (s, 3 H, $PhCOOCH_3$), 1.77 (m, 4 H, $PhOCH_2CH_2CH_2$-), 1.45 (m, 4 H, $PhOCH_2CH_2CH_2$-), 1.26 (m, 44 H, $PhOCH_2CH_2CH_2(CH_2)_{14}CH_3$ and $PhOCH_2CH_2CH_2(CH_2)_8CH_3$), 0.88 (t, 6 H, $PhO(CH_2)_{17}CH_3$ and $PhO(CH_2)_{11}CH_3$). ^{13}C NMR (101 MHz, $CDCl_3$) δ 167.1, 160.3, 132.0, 107.8, 106.7, 77.4, 68.5, 68.2, 52.3, 51.9, 32.1, 29.8, 29.8, 29.8, 29.7, 29.7, 29.7, 29.6, 29.5, 29.5, 29.4, 26.2, 22.7, 14.2. Mp = 62 °C.

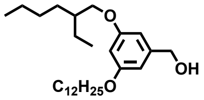

12a

(3-(2-Ethylhexyl)oxy)-5-(dodecyloxy)phenyl)methanol (12a). Compound **11a** (0.55 g, 1.23 mmol, 1 equiv) was dissolved in 5 mL dry THF, which was added dropwise to a slurry of $LiAlH_4$ (47 mg, 1.23 mmol, 1 equiv) in dry THF (5 mL) at 0 °C under N_2 atmosphere. Afterwards, the resulted mixture was stirred at 23 °C for 1 h. Finally, the reaction was quenched by the successive addition of water (0.2 mL), 15% NaOH aqueous solution (0.2 mL) and water (1.0 mL). White precipitates were formed in the mixture and filtered out. The filtrate was dried over anhydrous $MgSO_4$ and filtered. The solvent was removed under reduced pressure to afford the title compound as a colorless oil (0.36 g, 70%). ^1H NMR (400 MHz, $CDCl_3$) δ 6.50 (d, 2 H, Ph*H*), 6.38 (t, 1H, Ph*H*), 4.61 (s, 2 H, $PhCH_2OH$), 3.93 (t, 2 H, $PhOCH_2(CH_2)_{10}CH_3$), 3.82 (m, 2 H, $PhOCH_2CH(CH_2CH_3)$-), 1.86–1.65 (m, 3 H, $PhOCH_2CH_2(CH_2)_9CH_3$ and $PhOCH_2CH(CH_2CH_3)(CH_2)_3CH_3$), 1.55–1.18 (m, 26 H, $PhOCH_2CH_2(CH_2)_9CH_3$ and $PhOCH_2CH(CH_2CH_3)(CH_2)_3CH_3$), 0.90 (m, 9 H, $PhO(CH_2)_{11}CH_3$ and $PhOCH_2CH(CH_2CH_3)(CH_2)_3CH_3$). ^{13}C NMR (101 MHz, $CDCl_3$) δ 161.0, 160.7, 143.3, 105.3, 105.2, 105.1, 100.7, 77.4, 70.7, 68.2, 65.6, 65.6, 39.5, 32.1, 30.7, 29.8, 29.8, 29.7, 29.7, 29.5, 29.5, 29.4, 29.2, 26.2, 24.0, 23.2, 22.8, 14.3, 14.2, 11.2.

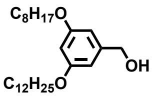

12b

(3-(Dodecyloxy)-5-(octyloxy)phenyl)methanol (12b). Compound **12b** was synthesized from compound **11b** (0.46 g, 1.03 mmol, 1 equiv) and $LiAlH_4$ (39 mg, 1.03 mmol, 1 equiv) following a procedure similar to that used for the synthesis of compound **12a**. The title compound was obtained as a colorless oil (0.43 g, 100%). ^{1}H NMR (400 MHz, $CDCl_3$) δ 6.50 (br, 2 H, Ph*H*), 6.38 (t, 1 H, Ph*H*), 4.61 (s, 2 H, PhCH_2OH), 3.93 (t, 4 H, PhOC$H_2CH_2CH_2$-), 1.77 (m, 4 H, $PhOCH_2CH_2CH_2$-), 1.44 (m, 4 H, $PhOCH_2CH_2CH_2$-), 1.29 (m, 18 H, $PhOCH_2CH_2CH_2(CH_2)_4CH_3$ and $PhOCH_2CH_2CH_2(CH_2)_5CH_3$), 0.89 (t, 6 H, $PhO(CH_2)_7CH_3$ and $PhO(CH_2)_8CH_3$). ^{13}C NMR (101 MHz, $CDCl_3$) δ 160.7, 143.3, 105.2, 100.7, 77.4, 68.2, 65.6, 65.6, 32.1, 32.0, 29.8, 29.8, 29.8, 29.7, 29.5, 29.5, 29.4, 29.4, 26.2, 22.8, 22.8, 14.3, 14.2.

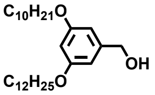

12c

(3-(Decyloxy)-5-(dodecyloxy)phenyl)methanol (12c). Compound **12c** was synthesized from compound **11c** (0.69 g, 1.45 mmol, 1 equiv) and $LiAlH_4$ (55 mg, 1.45 mmol, 1 equiv) following a procedure similar to that used for the synthesis of compound **12a**. The title compound was obtained as a colorless oil (0.52 g, 83%). ^{1}H NMR (400 MHz, $CDCl_3$) δ 6.49 (br, 2 H, Ph*H*), 6.37 (t, 1 H, Ph*H*), 4.60 (s, 2 H, PhCH_2OH), 3.93 (t, 4 H, $PhOCH_2CH_2CH_2$-), 1.76 (m, 4 H, $PhOCH_2CH_2CH_2$-), 1.44 (m, 4 H, $PhOCH_2CH_2CH_2$-), 1.29 (m, 28 H, $PhOCH_2CH_2CH_2(CH_2)_6CH_3$ and $PhOCH_2CH_2CH_2(CH_2)_8CH_3$), 0.88 (t, 6 H, $PhO(CH_2)_9CH_3$ and $PhO(CH_2)_{11}CH_3$). ^{13}C NMR (101 MHz, $CDCl_3$) δ 160.7, 143.4, 105.2, 100.7, 68.2, 65.5, 32.1, 32.0, 29.8, 29.8, 29.7, 29.7, 29.5, 29.5, 29.5, 29.4, 26.2, 22.8, 14.2.

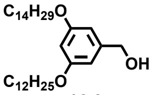

12d

(3-(Tetradecyloxy)-5-(dodecyloxy)phenyl)methanol (12d). Compound **12d** was synthesized from compound **11d** (0.78 g, 1.46 mmol, 1 equiv) and $LiAlH_4$ (56 mg, 1.46 mmol, 1 equiv) following a procedure similar to that used for the synthesis of compound **12a**. The title compound was obtained as a colorless oil (0.63 g, 86%). ^{1}H NMR (400 MHz, $CDCl_3$) δ 6.50 (br, 2 H, Ph*H*), 6.38 (t, 1 H, Ph*H*), 4.61 (s, 2 H, PhCH_2OH), 3.93 (t, 4 H, $PhOCH_2CH_2CH_2$-), 1.76 (m, 4 H, $PhOCH_2CH_2CH_2$-), 1.44 (m, 4 H, $PhOCH_2CH_2CH_2$-), 1.29 (m, 36 H, $PhOCH_2CH_2CH_2(CH_2)_{10}CH_3$ and $PhOCH_2CH_2CH_2(CH_2)_8CH_3$), 0.89 (t, 6 H, $PhO(CH_2)_{13}CH_3$ and $PhO(CH_2)_{11}CH_3$). ^{13}C NMR (101 MHz, $CDCl_3$) δ 160.7, 143.4, 105.2, 100.7, 77.4, 68.2, 65.5, 32.9, 32.1, 29.8, 29.8, 29.8, 29.8, 29.7, 29.6, 29.5, 29.5, 29.4, 26.2, 25.9, 22.8, 14.3.

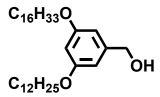

12e

(3-(Dodecyloxy)-5-(hexadecyloxy)phenyl)methanol (12e). Compound **12d** was synthesized from **12f** (580 mg, 1.21 mmol, 1 equiv) and $LiAlH_4$ (46 mg, 1.21 mmol, 1 equiv) following a procedure similar to that used for the synthesis of compound **12a**. The title compound was obtained as a white solid (630 mg, 98%). ^1H NMR (400 MHz, $CDCl_3$) δ 6.50 (br, 2 H, Ph*H*), 6.38 (t, 1 H, Ph*H*), 4.62 (s, 2 H, $PhC\mathit{H_2}OH$), 3.93 (t, 4 H, $PhOC\mathit{H_2}CH_2CH_2$-), 1.75 (m, 4 H, $PhOCH_2C\mathit{H_2}CH_2$-), 1.44 (m, 4 H, $PhOCH_2CH_2C\mathit{H_2}$-), 1.30 (m, 40 H, $PhOCH_2CH_2CH_2(C\mathit{H_2})_{12}CH_3$ and $PhOCH_2CH_2CH_2(C\mathit{H_2})_8CH_3$), 0.88 (t, 6 H, $PhO(CH_2)_{15}C\mathit{H_3}$ and $PhO(CH_2)_{11}C\mathit{H_3}$). ^{13}C NMR (101 MHz, $CDCl_3$) δ 160.6, 160.6, 143.2, 105.1, 100.6, 77.3, 77.0, 76.7, 68.1, 65.5, 31.9, 30.3, 29.7, 29.7, 29.7, 29.6, 29.6, 29.5, 29.4, 29.4, 29.4, 29.3, 26.1, 22.7, 14.1. Mp = 40 °C.

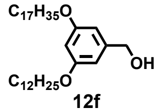

12f

(3-(Heptadecyloxy)-5-(decyloxy)phenyl)methanol (12f). Compound **12f** was synthesized from compound **11f** (0.72 g, 1.25 mmol, 1 equiv) and $LiAlH_4$ (48 mg, 1.25 mmol, 1 equiv) following a procedure similar to that used for the synthesis of compound **12a**. The title compound was obtained as a white solid (0.61 g, 89%). ^1H NMR (400 MHz, $CDCl_3$) δ 6.50 (br, 2 H, Ph*H*), 6.38 (t, 1 H, Ph*H*), 4.61 (s, 2 H, $PhC\mathit{H_2}OH$), 3.93 (t, 4 H, $PhOC\mathit{H_2}CH_2CH_2$-), 1.76 (m, 4 H, $PhOCH_2C\mathit{H_2}CH_2$-), 1.44 (m, 4 H, $PhOCH_2CH_2C\mathit{H_2}$-), 1.29 (m, 42 H, $PhOCH_2CH_2CH_2(C\mathit{H_2})_{13}CH_3$ and $PhOCH_2CH_2CH_2(C\mathit{H_2})_8CH_3$), 0.89 (t, 6 H, $PhO(CH_2)_{16}C\mathit{H_3}$ and $PhO(CH_2)_{11}C\mathit{H_3}$). ^{13}C NMR (101 MHz, $CDCl_3$) δ 160.7, 143.4, 105.2, 100.7, 68.2, 65.6, 63.2, 33.0, 32.1, 29.8, 29.8, 29.8, 29.8, 29.7, 29.6, 29.5, 29.5, 29.4, 26.2, 25.9, 22.8, 14.2. Mp = 45 °C.

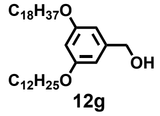

12g

(3-(Dodecyloxy)-5-(octadecyloxy)phenyl)methanol (12g). Compound **12g** was synthesized from compound **11g** (770 mg, 1.31 mmol, 1 equiv) and $LiAlH_4$ (50 mg, 1.31 mmol, 1 equiv) following a procedure similar to that used for the synthesis of compound **12a**. The title compound was obtained as a colorless oil (729 mg, 99%). ^1H NMR (400 MHz, $CDCl_3$) δ 6.49 (br, 2 H, Ph*H*), 6.37 (t, 1 H, Ph*H*), 4.60 (s, 2 H, $PhC\mathit{H_2}OH$), 3.93 (t, 4 H, $PhOC\mathit{H_2}CH_2CH_2$-), 1.75 (m, 4 H, $PhOCH_2C\mathit{H_2}CH_2$-), 1.43 (m, 4 H, $PhOCH_2CH_2C\mathit{H_2}$-), 1.28 (m, 44 H, $PhOCH_2CH_2CH_2(C\mathit{H_2})_{14}CH_3$ and $PhOCH_2CH_2CH_2(C\mathit{H_2})_8CH_3$), 0.89

(t, 6 H, PhO(CH_2)$_{17}$C*H$_3$* and PhO(CH_2)$_{11}$C*H$_3$*). ^{13}C NMR (101 MHz, $CDCl_3$) δ 160.6, 143.2, 105.1, 100.6, 68.1, 65.5, 31.9, 31.8, 30.3, 29.6, 29.6, 29.4, 29.4, 29.3, 29.3, 29.3, 26.1, 22.7, 14.1. Mp = 48 °C.

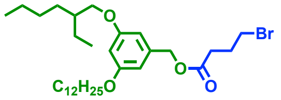

13a

(3-(2-Ethylhexyl)oxy)-5-(dodecyloxy)benzyl 4-bromobutanoate (13a). Compound **12a** (0.36 g, 0.80 mmol, 1 equiv), 4-bromobutyric acid (0.15 g, 0.88 mmol, 1.1 equiv) and DPTS (0.26 g, 0.88 mmol, 1.1 equiv) were dissolved in 10 mL DCM. DCC (0.33 g, 1.60 mmol, 2 equiv) was added into the above mixture. The reaction mixture was stirred at 23 °C for 12 h. Afterwards, the resulted precipitates (urea) were filtered out from the reaction mixture. The filtrate was concentrated to around 3 mL and purified by column chromatography (SiO_2) with hexane/DCM = 1/1 as the mobile phase to give the title compound as a colorless oil (0.38 g, 83%). ^{1}H NMR (400 MHz, $CDCl_3$) δ 6.47 (d, 2 H, Ph*H*), 6.41 (t, 1 H, Ph*H*), 5.05 (s, 2 H, PhC*H$_2$*OCO), 3.93 (t, 2 H, PhOC*H$_2$*(CH_2)$_7$$CH_3$), 3.82 (m, 2 H, PhOC*H$_2$*CH($CH_2$$CH_3$)-), 3.47 (t, 2 H, -OCOC$H_2CH_2$C*H$_2$*Br), 2.57 (t, 2 H, -OCOC*H$_2$*CH_2CH_2Br), 2.20 (m, 2 H, -OCOCH_2C*H$_2$*CH_2Br), 1.83–1.67 (m, 3 H, PhOCH_2C*H$_2$*(CH_2)$_9$$CH_3$ and PhOCH_2C*H*($CH_2$$CH_3$)($CH_2$)$_3$$CH_3$), 1.57–1.21 (m, 26 H, PhOCH_2CH_2(C*H$_2$*)$_9$$CH_3$ and PhOCH_2CH(C*H$_2$*CH_3)(C*H$_2$*)$_3$$CH_3$), 0.91 (m, 9 H, PhO($CH_2$)$_{11}$C*H$_3$* and PhOC$H_2$CH($CH_2$C*H$_3$*)($CH_2$)$_3$C*H$_3$*). ^{13}C NMR (101 MHz, $CDCl_3$) δ 172.5, 172.4, 160.9, 160.6, 137.9, 106.6, 106.5, 106.4, 101.2, 77.5, 77.2, 76.9, 70.7, 68.2, 66.6, 39.5, 32.8, 32.7, 32.6, 32.1, 30.7, 29.8, 29.8, 29.8, 29.7, 29.6, 29.5, 29.5, 29.4, 29.2, 28.0, 27.9, 26.2, 24.0, 23.2, 22.8, 14.3, 14.2, 11.3.

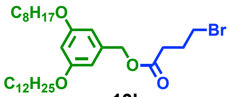

13b

3-(Octyloxy)-5-(dodecyloxy)benzyl 4-bromobutanoate (13b). Compound **13b** was synthesized from compound **12b** (0.43 g, 0.96 mmol, 1 equiv), 4-bromobutyric acid (0.18 g, 1.05 mmol, 1.1 equiv), DPTS (0.31 g, 1.05 mmol, 1.1 equiv) and DCC (0.40 g, 1.92 mmol, 2 equiv) following a procedure similar to that used for the synthesis of compound **13a**. The title compound was obtained as a colorless oil (0.43 g, 79%). ^{1}H NMR (400 MHz, $CDCl_3$) δ 6.47 (br, 2 H, Ph*H*), 6.41 (t, 1 H, Ph*H*), 5.05 (s, 2 H, PhC*H$_2$*OCO), 3.93 (t, 4 H, PhOC*H$_2$*CH_2CH_2-), 3.46 (t, 2 H, -OCOCH_2CH_2C*H$_2$*Br), 2.56 (t, 2 H, -OCOC*H$_2$*CH_2CH_2Br), 2.20 (m, 2 H, -OCOCH_2C*H$_2$*CH_2Br), 1.77 (m, 4 H, PhOCH_2C*H$_2$*CH_2-), 1.44 (m, 4 H, PhOCH_2CH_2C*H$_2$*-), 1.28 (m, 24 H, PhOCH_2CH_2CH_2(C*H$_2$*)$_4$$CH_3$ and PhOCH_2CH_2CH_2(C*H$_2$*)$_8$$CH_3$), 0.89 (t, 6 H, PhO($CH_2$)$_7$C*H$_3$* and PhO($CH_2$)$_{11}$C*H$_3$*). ^{13}C NMR (101 MHz, $CDCl_3$) δ 172.4, 160.6, 138.0, 106.5, 101.2,

77.5, 77.2, 76.9, 68.3, 68.2, 66.5, 32.6, 32.1, 32.0, 29.8, 29.8, 29.8, 29.6, 29.5, 29.4, 27.9, 26.2, 22.8, 22.8, 14.3, 14.2.

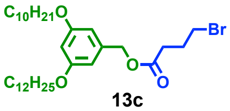

13c

3-(Decyloxy)-5-(dodecyloxy)benzyl 4-bromobutanoate (13c). Compound **13c** was synthesized from compound **12c** (0.20 g, 0.45 mmol, 1 equiv), 4-bromobutyric acid (0.08 g, 0.49 mmol, 1.1 equiv), DPTS (0.14 g, 0.49 mmol, 1.1 equiv) and DCC (0.18 g, 0.89 mmol, 2 equiv) following a procedure similar to that used for the synthesis of compound **13a**. The title compound was obtained as a colorless oil (0.24 g, 90%). ^{1}H NMR (400 MHz, $CDCl_3$) δ 6.46 (br, 2 H, Ph*H*), 6.40 (t, 1 H, Ph*H*), 5.05 (s, 2 H, PhCH_2OCO), 3.93 (t, 4 H, PhOC$H_2$$CH_2$$CH_2$-), 3.47 (t, 2 H, -OCO$CH_2$$CH_2CH_2$Br), 2.57 (t, 2 H, -OCOC$H_2$$CH_2$$CH_2$Br), 2.20 (m, 2 H, -OCO$CH_2CH_2$$CH_2$Br), 1.76 (m, 4 H, PhO$CH_2CH_2$$CH_2$-), 1.44 (m, 4 H, PhO$CH_2$$CH_2CH_2$-), 1.28 (m, 28 H, PhO$CH_2$$CH_2$$CH_2$(C$H_2$)$_6$$CH_3$ and PhO$CH_2$$CH_2$$CH_2$(C$H_2$)$_8$$CH_3$), 0.89 (t, 6 H, PhO($CH_2$)$_9CH_3$ and PhO(CH_2)$_{11}$CH_3). ^{13}C NMR (101 MHz, $CDCl_3$) δ 172.4, 160.6, 137.9, 106.5, 101.2, 68.2, 66.5, 32.8, 32.6, 32.1, 32.0, 29.8, 29.8, 29.8, 29.7, 29.7, 29.7, 29.5, 29.5, 29.5, 29.4, 27.9, 26.2, 22.8, 14.3.

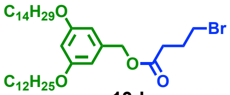

13d

3-(Dodecyloxy)-5-(tetradecyloxy)benzyl 4-bromobutanoate (13d). Compound **13d** was synthesized from compound **12d** (0.63 g, 1.24 mmol, 1 equiv), 4-bromobutyric acid (0.22 g, 1.36 mmol, 1.1 equiv), DPTS (0.40 g, 1.36 mmol, 1.1 equiv) and DCC (0.57 g, 2.48 mmol, 2 equiv) following a procedure similar to that used for the synthesis of compound **13a**. The title compound was obtained as a colorless oil (0.75 g, 97%). ^{1}H NMR (400 MHz, $CDCl_3$) δ 6.46 (br, 2 H, Ph*H*), 6.40 (t, 1 H, Ph*H*), 5.04 (s, 2 H, PhCH_2OCO), 3.93 (t, 4 H, PhOC$H_2$$CH_2$$CH_2$-), 3.47 (t, 2 H, -OCO$CH_2$$CH_2CH_2$Br), 2.56 (t, 2 H, -OCOC$H_2$$CH_2$$CH_2$Br), 2.20 (m, 2 H, -OCO$CH_2CH_2$$CH_2$Br), 1.76 (m, 4 H, PhO$CH_2CH_2$$CH_2$-), 1.45 (m, 4 H, PhO$CH_2$$CH_2CH_2$-), 1.28 (m, 36 H, PhO$CH_2$$CH_2$$CH_2$(C$H_2$)$_{10}$$CH_3$ and PhO$CH_2$$CH_2$$CH_2$(C$H_2$)$_8$$CH_3$), 0.89 (t, 6 H, PhO($CH_2$)$_{13}CH_3$ and PhO(CH_2)$_{11}$CH_3). ^{13}C NMR (101 MHz, $CDCl_3$) δ 172.4, 160.6, 137.9, 106.5, 101.2, 68.2, 66.5, 64.9, 32.7, 32.6, 32.1, 29.8, 29.8, 29.8, 29.8, 29.7, 29.7, 29.5, 29.5, 29.4, 27.9, 26.2, 22.8, 14.3.

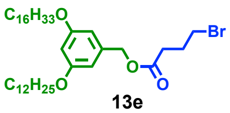

13e

3-(Dodecyloxy)-5-(hexadecyloxy)benzyl 4-bromobutanoate (13e). Compound **13e** was synthesized from compound **12e** (0.20 g, 0.38 mmol, 1 equiv), 4-bromobutyric acid (0.07 g, 0.41 mmol, 1.1 equiv), DPTS (0.12 g, 0.41 mmol, 1.1 equiv) and DCC (0.16 mg, 0.75 mmol, 2 equiv) following a procedure similar to that used for the synthesis of compound **13a**. The title compound was obtained as a colorless oil (0.25 g, 97%). ^{1}H NMR (400 MHz, $CDCl_3$) δ 6.47 (br, 2 H, Ph*H*), 6.40 (t, 1 H, Ph*H*), 5.05 (s, 2 H, PhCH_2OCO), 3.93 (t, 4 H, PhOC$H_2$$CH_2$$CH_2$-), 3.46 (t, 2 H, -OCO$CH_2$$CH_2CH_2$Br), 2.56 (t, 2 H, -OCOC$H_2$$CH_2$$CH_2$Br), 2.21 (m, 2 H, -OCO$CH_2CH_2$$CH_2$Br), 1.77 (m, 4 H, PhO$CH_2CH_2$$CH_2$-), 1.43 (m, 4 H, PhO$CH_2$$CH_2CH_2$-), 1.27 (m, 40 H, PhO$CH_2$$CH_2$$CH_2$(C$H_2$)$_{12}$$CH_3$ and PhO$CH_2$$CH_2$$CH_2$(C$H_2$)$_8$$CH_3$), 0.89 (t, 6 H, PhO($CH_2$)$_9CH_3$ and PhO(CH_2)$_7$CH_3). ^{13}C NMR (101 MHz, $CDCl_3$) δ 172.3, 160.5, 137.8, 106.4, 101.1, 77.4, 77.03 76.7, 76.7, 68.1, 66.4, 32.6, 32.5, 31.22, 29.7, 29.7, 29.7, 29.7, 29.64, 29.61, 29.4, 29.4, 29.3, 27.7, 26.1, 22.7, 14.1.

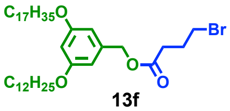

13f

3-(Dodecyloxy)-5-(heptadecyloxy)benzyl 4-bromobutanoate (13f). Compound **13f** was synthesized from compound **12f** (0.58 g, 1.06 mmol, 1 equiv), 4-bromobutyric acid (0.20 g, 1.17 mmol, 1.1 equiv), DPTS (0.34 g, 1.17 mmol, 1.1 equiv) and DCC (0.45 g, 2.12 mmol, 2 equiv) following a procedure similar to that used for the synthesis of compound **13a**. The title compound was obtained as a colorless oil (0.71 g, 96%). ^{1}H NMR (400 MHz, $CDCl_3$) δ 6.46 (br, 2 H, Ph*H*), 6.40 (t, 1 H, Ph*H*), 5.04 (s, 2 H, PhCH_2OCO), 3.93 (t, 4 H, PhOC$H_2$$CH_2$$CH_2$-), 3.46 (t, 2 H, -OCO$CH_2$$CH_2CH_2$Br), 2.56 (t, 2 H, -OCOC$H_2$$CH_2$$CH_2$Br), 2.21 (m, 2 H, -OCO$CH_2CH_2$$CH_2$Br), 1.76 (m, 4 H, PhO$CH_2CH_2$$CH_2$-), 1.44 (m, 4 H, PhO$CH_2$$CH_2CH_2$-), 1.28 (m, 42 H, PhO$CH_2$$CH_2$$CH_2$(C$H_2$)$_{13}$$CH_3$ and PhO$CH_2$$CH_2$$CH_2$(C$H_2$)$_8$$CH_3$), 0.89 (t, 6 H, PhO($CH_2$)$_{16}CH_3$ and PhO(CH_2)$_{11}$CH_3). ^{13}C NMR (101 MHz, $CDCl_3$) δ 172.4, 160.6, 137.9, 106.5, 101.2, 68.2, 66.5, 64.9, 32.8, 32.7, 32.6, 32.6, 32.1, 29.8, 29.8, 29.8, 29.7, 29.7, 29.6, 29.5, 29.5, 29.4, 28.7, 27.9, 27.9, 26.2, 26.0, 22.8, 14.2.

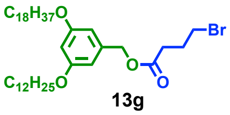

13g

3-(Dodecyloxy)-5-(octadecyloxy)benzyl 4-bromobutanoate (13g). Compound **13g** was synthesized from compound **12g** (0.20 g, 0.34 mmol, 1 equiv), 4-bromobutyric acid (0.06 g, 0.37 mmol, 1.1 equiv),

DPTS (0.11 g, 0.37 mmol, 1 equiv) and DCC (0.14 g, 0.68 mmol, 2 equiv) following a procedure similar to that used for the synthesis of compound 30a. The title compound was obtained as a colorless oil (0.24 g, 100%). ^{1}H NMR (400 MHz, $CDCl_3$) δ 6.46 (br, 2 H, Ph*H*), 6.40 (t, 1 H, Ph*H*), 5.04 (s, 2 H, PhC*H*$_2$OCO), 3.93 (t, 4 H, PhOC*H*$_2$CH$_2$CH$_2$-), 3.47 (t, 2 H, -OCOCH$_2$CH$_2$C*H*$_2$Br), 2.57 (t, 2 H, -OCOC*H*$_2$CH$_2$CH$_2$Br), 2.20 (m, 2 H, -OCOCH$_2$C*H*$_2$CH$_2$Br), 1.76 (m, 4 H, PhOCH$_2$C*H*$_2$CH$_2$-), 1.43 (m, 4 H, PhOCH$_2$CH$_2$C*H*$_2$-), 1.27 (m, 44 H, PhOCH$_2$CH$_2$CH$_2$(C*H*$_2$)$_{14}$CH$_3$ and PhOCH$_2$CH$_2$CH$_2$(C*H*$_2$)$_8$CH$_3$), 0.88 (t, 6 H, PhO(CH$_2$)$_{17}$C*H*$_3$ and PhO(CH$_2$)$_{11}$C*H*$_3$). ^{13}C NMR (101 MHz, $CDCl_3$) δ 172.3, 160.5, 137.8, 106.4, 101.2, 68.1, 66.4, 32.6, 32.5, 31.9, 31.8, 29.6, 29.6, 29.4, 29.4, 29.4, 29.3, 29.3, 27.8, 26.1, 22.7, 22.7, 14.1.

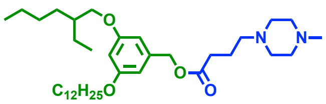

14a (IAJD197)

3-((2-Ethylhexyl)oxy)-5-(dodecyloxy)benzyl 4-(4-methylpiperazin-1-yl)butanoate (14a, IAJD197). A mixture of compound **13a** (0.19 g, 0.33 mmol, 1.0 equiv), 1-methylpiperazine (36 mg, 0.36 mmol, 1.1 equiv), K_2CO_3 (56 mg, 0.40 mmol, 1.2 equiv) and MeCN (15 mL) was heated to 95 °C and stirred for 3 h. The reaction mixture was cooled to 23 °C and MeCN was removed under reduced pressure. Then water (20 mL) was added, and the resulted mixture was extracted with DCM (20 mL) for three times. The organic phases were collected and dried over anhydrous $MgSO_4$. After filtration, the obtained filtrate was concentrated to around 3 mL and purified by column chromatography (SiO_2) with DCM/MeOH = 30/1 and 15/1 as the eluent. Then the obtained product was dissolved in DCM (20 mL), which was washed by $NaHCO_3$ solution (2%, 20 mL). The aqueous phase was extracted by DCM (20 mL) for another two times. The organic phases were combined and dried over anhydrous $MgSO_4$. Filtration and evaporation of the solvent yielded the title compound as a colorless oil (0.16 g, 81%). ^{1}H NMR (400 MHz, $CDCl_3$) δ 6.45 (br, 2 H, Ph*H*), 6.39 (t, 1 H, Ph*H*), 5.01 (s, 2 H, PhC*H*$_2$-), 3.91 (t, 4 H, PhOC*H*$_2$-), 3.79 (m, 2 H, PhOC*H*$_2$CH(CH$_2$CH$_3$)-), 2.64–2.26 (m, 15 H, -N(C*H*$_2$C*H*$_2$)$_2$N-, -NC*H*$_2$CH$_2$C*H*$_2$COO- and -NC*H*$_3$), 1.83 (m, 2 H, -OCOCH$_2$C*H*$_2$CH$_2$-), 1.72 (m, 3 H, PhOCH$_2$C*H*$_2$(CH$_2$)$_9$CH$_3$ and PhOCH$_2$C*H*(CH$_2$CH$_3$)(CH$_2$)$_3$CH$_3$), 1.45–1.16 (m, 26 H, PhOCH$_2$CH$_2$(C*H*$_2$)$_9$CH$_3$ and PhOCH$_2$CH(C*H*$_2$CH$_3$)(C*H*$_2$)$_3$CH$_3$), 0.91 (m, 9 H, PhO(CH$_2$)$_{11}$C*H*$_3$ and PhOCH$_2$CH(CH$_2$C*H*$_3$)(CH$_2$)$_3$C*H*$_3$). ^{13}C NMR (101 MHz, $CDCl_3$) δ 173.4, 160.8, 160.5, 138.1, 106.6, 106.5, 106.4, 101.0, 70.6, 68.2, 66.3, 66.2, 57.6, 55.1, 52.8, 45.9, 39.5, 32.3, 32.0, 30.6, 29.8, 29.7, 29.7, 29.7, 29.5, 29.4, 29.4, 29.2, 26.2, 24.0, 23.1, 22.8, 22.2, 14.2, 14.2, 11.2. Purity by HPLC: 99+%. MALDI-TOF MS m/z of $[M + H]^+$ calculated for $C_{36}H_{65}N_2O_4$: 589.5; Found: 590.2.

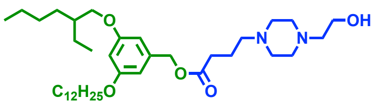

14b (IAJD198)

3-((2-Ethylhexyl)oxy)-5-(dodecyloxy)benzyl 4-(4-(2-hydroxyethyl)piperazin-1-yl)butanoate (14b, IAJD198). Compound **14b** was synthesized from compound **13a** (0.19 g, 0.33 mmol), 1-(2-hydroxyethyl)piperazine (66 mg, 0.50 mmol) and K_2CO_3 (56 mg, 0.40 mmol) following a procedure similar to that used for the synthesis of compound **14a**. The title compound was obtained as a colorless oil (0.16 g, 77%). ^1H NMR (400 MHz, $CDCl_3$) δ 6.45 (br, 2 H, Ph*H*), 6.39 (t, 1 H, Ph*H*), 5.02 (s, 2 H, $PhC\mathit{H}_2$-), 3.91 (t, 4 H, $PhOC\mathit{H}_2$-), 3.80 (m, 2 H, $PhOC\mathit{H}_2CH(CH_2CH_3)$-), 3.61 (t, 2 H, -$CH_2C\mathit{H}_2OH$), 2.70–2.29 (m, 14 H, -$N(C\mathit{H}_2C\mathit{H}_2)_2N$-, -$NC\mathit{H}_2CH_2C\mathit{H}_2COO$- and -$NC\mathit{H}_2$-), 1.83 (m, 2 H, -$OCOCH_2C\mathit{H}_2CH_2$-), 1.73 (m, 3 H, $PhOCH_2C\mathit{H}_2(CH_2)_9CH_3$ and $PhOCH_2C\mathit{H}(CH_2CH_3)(CH_2)_3CH_3$), 1.52–1.15 (m, 26 H, $PhOCH_2CH_2(C\mathit{H}_2)_9CH_3$ and $PhOCH_2CH(C\mathit{H}_2CH_3)(C\mathit{H}_2)_3CH_3$), 0.89 (m, 9 H, $PhO(CH_2)_{11}C\mathit{H}_3$ and $PhOCH_2CH(CH_2C\mathit{H}_3)(CH_2)_3C\mathit{H}_3$). ^{13}C NMR (101 MHz, $CDCl_3$) δ 173.4, 160.8, 160.6, 138.2, 138.1, 106.6, 106.5, 106.4, 101.0, 70.6, 68.2, 66.3, 59.4, 57.8, 57.6, 53.1, 53.0, 39.5, 32.3, 32.0, 30.6, 29.8, 29.7, 29.7, 29.7, 29.5, 29.5, 29.4, 29.2, 26.2, 24.0, 23.1, 22.8, 22.2, 14.2, 14.2, 11.2. Purity by HPLC: 99+%. MALDI-TOF MS m/z of $[M + H]^+$ calculated for $C_{37}H_{67}N_2O_5$: 619.5; Found: 620.5.

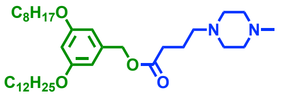

14c (IAJD199)

3-(Dodecyloxy)-5-(octyloxy)benzyl 4-(4-methylpiperazin-1-yl)butanoate (14c, IAJD199). Compound **14c** was synthesized from compound **13b** (0.22 g, 0.38 mmol), 1-methylpiperazine (46 mg, 0.46 mmol) and K_2CO_3 (64 mg, 0.46 mmol) following a procedure similar to that used for the synthesis of compound **14a**. The title compound was obtained as a colorless oil (0.19 g, 84%). ^1H NMR (400 MHz, $CDCl_3$) δ 6.46 (br, 2 H, Ph*H*), 6.39 (t, 1 H, Ph*H*), 5.02 (s, 2 H, $PhC\mathit{H}_2$-), 3.92 (t, 4 H, $PhOC\mathit{H}_2$-), 2.77–2.14 (m, 15 H, -$N(C\mathit{H}_2C\mathit{H}_2)_2N$-, -$NC\mathit{H}_2CH_2C\mathit{H}_2COO$- and -$NC\mathit{H}_3$), 1.83 (m, 2 H, -$OCOCH_2C\mathit{H}_2CH_2$-), 1.76 (m, 4 H, $PhOCH_2C\mathit{H}_2$-), 1.44 (m, 4 H, $PhOCH_2CH_2C\mathit{H}_2$-), 1.27 (br, 24 H, $PhOCH_2CH_2CH_2(C\mathit{H}_2)_4CH_3$ and $PhOCH_2CH_2CH_2(C\mathit{H}_2)_8CH_3$), 0.88 (t, 6 H, -$CH_2CH_2C\mathit{H}_3$). ^{13}C NMR (101 MHz, $CDCl_3$) δ 173.5, 160.6, 138.2, 106.5, 101.1, 68.2, 66.3, 57.7, 55.3, 53.2, 46.1, 32.4, 32.1, 32.0, 29.8, 29.8, 29.8, 29.7, 29.5, 29.5, 29.4, 29.4, 26.2, 22.8, 22.8, 22.3, 14.3, 14.2. Purity by HPLC: 99+%. MALDI-TOF MS m/z of $[M + H]^+$ calculated for $C_{36}H_{65}N_2O_4$: 589.5; Found: 590.0.

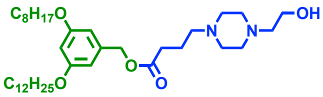

14d (IAJD200)

3-(Dodecyloxy)-5-(octyloxy)benzyl 4-(4-(2-hydroxyethyl)piperazin-1-yl)butanoate (14d, IAJD200). Compound **14d** was synthesized from compound **13b** (0.22 g, 0.38 mmol), 1-(2-hydroxyethyl)piperazine (60 mg, 0.46 mmol) and K_2CO_3 (64 mg, 0.46 mmol) following a procedure similar to that used for the synthesis of compound **14a**. The title compound was obtained as a colorless oil (0.18 g, 75%). ^{1}H NMR (400 MHz, $CDCl_3$) δ 6.46 (br, 2 H, Ph*H*), 6.39 (t, 1 H, Ph*H*), 5.02 (s, 2 H, PhCH_2-), 3.92 (t, 4 H, PhOCH_2-), 3.60 (t, 2 H, -CH_2CH_2OH), 2.74–2.12 (m, 14 H, -N(CH_2CH_2)$_2$N-, -NC$H_2$$CH_2CH_2$COO- and -C$H_2$$CH_2$OH), 1.81 (m, 2 H, -OCO$CH_2CH_2$$CH_2$-), 1.76 (m, 4 H, PhO$CH_2CH_2$-), 1.44 (m, 4 H, PhO$CH_2$$CH_2CH_2$-), 1.26 (br, 24 H, PhO$CH_2$$CH_2$$CH_2$(C$H_2$)$_4$$CH_3$ and PhO$CH_2$$CH_2$$CH_2$(C$H_2$)$_8$$CH_3$), 0.88 (t, 6 H, -$CH_2$$CH_2CH_3$). ^{13}C NMR (101 MHz, $CDCl_3$) δ 173.5, 160.6, 138.2, 106.6, 101.0, 68.2, 66.3, 59.3, 57.8, 57.7, 53.3, 53.0, 32.4, 32.1, 32.0, 29.9, 29.8, 29.8, 29.8, 29.7, 29.5, 29.5, 29.4, 29.4, 26.2, 22.8, 22.8, 22.3, 14.3, 14.2. Purity by HPLC: 99+%. MALDI-TOF MS m/z of $[M + H]^+$ calculated for $C_{37}H_{67}N_2O_5$: 619.5; Found: 620.3.

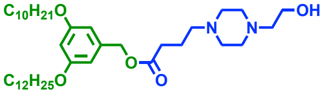

14e (IAJD201)

3-(Decyloxy)-5-(dodecyloxy)benzyl 4-(4-(2-hydroxyethyl)piperazin-1-yl)butanoate (14e, IAJD201). Compound **14e** was synthesized from compound **13c** (0.24 g, 0.40 mmol), 1-(2-hydroxyethyl)piperazine (78 mg, 0.60 mmol) and K_2CO_3 (67 mg, 0.48 mmol) following a procedure similar to that used for the synthesis of compound **14a**. The title compound was obtained as a colorless oil (0.24 g, 92%). ^{1}H NMR (400 MHz, $CDCl_3$) δ 6.45 (br, 2 H, Ph*H*), 6.38 (t, 1 H, Ph*H*), 5.01 (s, 2 H, PhCH_2-), 3.91 (t, 4 H, PhOCH_2-), 3.60 (t, 2 H, -CH_2CH_2OH), 2.65–2.29 (m, 14 H, -N(CH_2CH_2)$_2$N-, -NC$H_2$$CH_2CH_2$COO- and -C$H_2$$CH_2$OH), 1.83 (m, 2 H, -OCO$CH_2CH_2$$CH_2$-), 1.74 (m, 4 H, PhO$CH_2CH_2$-), 1.43 (m, 4 H, PhO$CH_2$$CH_2CH_2$-), 1.27 (br, 28 H, PhO$CH_2$$CH_2$$CH_2$(C$H_2$)$_6$$CH_3$ and PhO$CH_2$$CH_2$$CH_2$(C$H_2$)$_8$$CH_3$), 0.87 (t, 6 H, -$CH_2$$CH_2CH_3$). ^{13}C NMR (101 MHz, $CDCl_3$) δ 173.4, 160.5, 138.2, 106.5, 101.0, 77.4, 68.2, 66.2, 59.4, 57.8, 57.6, 53.1, 53.0, 32.3, 32.0, 32.0, 29.8, 29.7, 29.7, 29.7, 29.7, 29.5, 29.4, 29.4, 29.4, 26.2, 22.8, 22.2, 14.2. Purity by HPLC: 99+%. MALDI-TOF MS m/z of $[M + H]^+$ calculated for $C_{39}H_{71}N_2O_5$: 647.5; Found: 647.4.

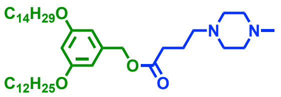

14f (IAJD202)

3-(Dodecyloxy)-5-(tetradecyloxy)benzyl 4-(4-methylpiperazin-1-yl)butanoate (14f, IAJD202). Compound **14f** was synthesized from compound **13d** (0.35 g, 0.54 mmol), 1-methylpiperazine (81 mg, 0.80 mmol) and K_2CO_3 (89 mg, 0.64 mmol) following a procedure similar to that used for the synthesis of compound **14a**. The title compound was obtained as a colorless oil (0.30 g, 86%). ^{1}H NMR (400 MHz, $CDCl_3$) δ 6.45 (br, 2 H, Ph*H*), 6.38 (t, 1 H, Ph*H*), 5.02 (s, 2 H, PhC*H*2-), 3.90 (t, 4 H, PhOC*H*2-), 2.63–2.21 (m, 15 H, -N(C*H*2C*H*2)2N-, -NC*H*2CH2C*H*2COO- and -NC*H*3), 1.83 (m, 2 H, -OCOCH2C*H*2CH2-), 1.74 (m, 4 H, PhOCH2C*H*2-), 1.42 (m, 4 H, PhOCH2CH2C*H*2-), 1.27 (br, 36 H, PhOCH2CH2CH2(C*H*2)10CH3 and PhOCH2CH2CH2(C*H*2)8CH3), 0.86 (t, 6 H, -CH2CH2C*H*3). ^{13}C NMR (101 MHz, $CDCl_3$) δ 173.2, 160.4, 138.0, 106.4, 100.9, 77.4, 77.1, 76.8, 68.0, 66.1, 59.5, 57.7, 57.5, 52.9, 32.1, 31.9, 31.9, 29.6, 29.5, 29.5, 29.4, 29.3, 29.2, 26.0, 22.7, 22.1, 14.1. Purity by HPLC: 99+%. MALDI-TOF MS m/z of $[M + H]^+$ calculated for $C_{42}H_{77}N_2O_4$: 673.6; Found: 673.7.

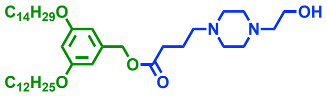

14g (IAJD203)

3-(Dodecyloxy)-5-(tetradecyloxy)benzyl 4-(4-(2-hydroxyethyl)piperazin-1-yl)butanoate (14g, IAJD203). Compound **14g** was synthesized from compound **13d** (0.35 g, 0.54 mmol), 1-(2-hydroxyethyl)piperazine (105 mg, 0.80 mmol) and K_2CO_3 (90 mg, 0.64 mmol) following a procedure similar to that used for the synthesis of compound **14a**. The title compound was obtained as a colorless oil (0.32 g, 84%). ^{1}H NMR (400 MHz, $CDCl_3$) δ 6.45 (br, 2 H, Ph*H*), 6.39 (t, 1 H, Ph*H*), 5.02 (s, 2 H, PhC*H*2-), 3.91 (t, 4 H, PhOC*H*2-), 3.60 (t, 2 H, -CH2C*H*2OH), 2.64–2.25 (m, 14 H, -N(C*H*2C*H*2)2N-, -NC*H*2CH2C*H*2COO- and -C*H*2CH2OH), 1.83 (m, 2 H, -OCOCH2C*H*2CH2-), 1.75 (m, 4 H, PhOCH2C*H*2-), 1.43 (m, 4 H, PhOCH2CH2C*H*2-), 1.27 (br, 40 H, PhOCH2CH2CH2(C*H*2)10CH3 and PhOCH2CH2CH2(C*H*2)8CH3), 0.87 (t, 6 H, -CH2CH2C*H*3). ^{13}C NMR (101 MHz, $CDCl_3$) δ 173.4, 160.6, 138.2, 106.5, 101.0, 68.2, 66.3, 59.4, 57.8, 57.6, 53.1, 53.0, 32.3, 32.0, 29.8, 29.8, 29.7, 29.7, 29.7, 29.5, 29.5, 29.4, 26.2, 22.8, 22.2, 14.2. Purity by HPLC: 99+%. MALDI-TOF MS m/z of $[M + H]^+$ calculated for $C_{43}H_{79}N_2O_5$: 703.6; Found: 703.3.

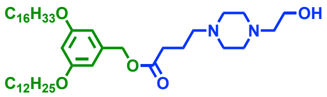

14h (IAJD204)

3-(Dodecyloxy)-5-(hexadecyloxy)benzyl 4-(4-(2-hydroxyethyl)piperazin-1-yl)butanoate (14h, IAJD204). Compound **14h** was synthesized from compound **13e** (250 mg, 0.38 mmol), 1-(2-hydroxyethyl)piperazine (74 mg, 0.57 mmol) and K_2CO_3 (64 mg, 0.46 mmol) following a procedure similar to that used for the synthesis of compound **14a**. The title compound was obtained as a colorless oil (200 mg, 74%). ^{1}H NMR (400 MHz, $CDCl_3$) δ 6.45 (s, 2 H, Ph*H*), 6.38 (t, 1 H, Ph*H*), 5.01 (s, 2 H, PhC*H*$_2$-), 3.90 (t, 4 H, PhOC*H*$_2$-), 3.61 (t, 2H, -CH_2C*H*$_2$OH), 2.57–2.32 (m, 14 H, -N(C*H*$_2$C*H*$_2$)$_2$N-, -NC*H*$_2$$CH_2$C*H*$_2$COO- and -C*H*$_2$$CH_2$OH), 1.83 (m, 2 H, -OCO$CH_2$C*H*$_2$$CH_2$-), 1.75 (m, 4 H, PhO$CH_2$C*H*$_2$-), 1.43 (m, 4 H, PhO$CH_2CH_2$C*H*$_2$-), 1.26 (br, 40 H, PhO$CH_2CH_2CH_2$(C*H*$_2$)$_{12}$$CH_3$ and PhO$CH_2CH_2CH_2$(C*H*$_2$)$_8$$CH_3$), 0.87 (t, 6 H, -$CH_2CH_2$C*H*$_3$). ^{13}C NMR (101 MHz, $CDCl_3$) δ 173.19, 173.16, 160.43, 138.05, 106.37, 100.85, 77.45, 77.13, 76.82, 68.02, 66.11, 66.10, 59.47, 57.72, 57.48, 52.94, 52.92, 52.91, 32.16, 31.92, 31.91, 29.70, 29.68, 29.66, 29.63, 29.60, 29.58, 29.40, 29.36, 29.34, 29.25, 26.05, 22.68, 22.07, 14.11. Purity by HPLC: 99+%. MALDI-TOF MS m/z of $[M + H]^+$ calculated for $C_{45}H_{83}N_2O_5$: 731.6; Found: 732.5.

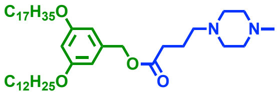

14i (IAJD205)

3-(Dodecyloxy)-5-(heptadecyloxy)benzyl 4-(4-methylpiperazin-1-yl)butanoate (14i, IAJD205). Compound **14i** was synthesized from compound **13f** (0.35 g, 0.50 mmol), 1-methylpiperazine (55 mg, 0.55 mmol) and K_2CO_3 (0.10 g, 0.72 mmol) following a procedure similar to that used for the synthesis of compound **14a**. The title compound was obtained as a colorless oil (0.31 g, 86%). ^{1}H NMR (400 MHz, $CDCl_3$) δ 6.46 (br, 2 H, Ph*H*), 6.39 (t, 1 H, Ph*H*), 5.02 (s, 2 H, PhC*H*$_2$-), 3.91 (t, 4 H, PhOC*H*$_2$-), 2.58–2.25 (m, 15 H, -N(C*H*$_2$C*H*$_2$)$_2$N-, -NC*H*$_2$$CH_2$C*H*$_2$COO- and -NC*H*$_3$), 1.84 (m, 2 H, -OCO$CH_2$C*H*$_2$$CH_2$-), 1.77 (m, 4 H, PhO$CH_2$C*H*$_2$-), 1.43 (m, 4 H, PhO$CH_2CH_2$C*H*$_2$-), 1.25 (br, 42 H, PhO$CH_2CH_2CH_2$(C*H*$_2$)$_{13}$$CH_3$ and PhO$CH_2CH_2CH_2$(C*H*$_2$)$_8$$CH_3$), 0.88 (t, 6 H, -$CH_2CH_2$C*H*$_3$). ^{13}C NMR (101 MHz, $CDCl_3$) δ 173.3, 160.5, 138.1, 106.4, 100.9, 68.1, 66.1, 59.3, 57.7, 57.5, 53.4, 53.0, 52.9, 32.2, 31.9, 29.7, 29.7, 29.6, 29.6, 29.6, 29.4, 29.4, 29.3, 26.1, 22.7, 22.1, 14.1. Purity by HPLC: 99+%. MALDI-TOF MS m/z of $[M + H]^+$ calculated for $C_{45}H_{83}N_2O_4$: 715.6; Found: 716.2.

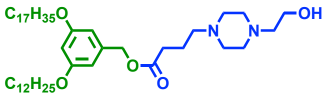

14j (IAJD206)

3-(Dodecyloxy)-5-(heptadecyloxy)benzyl 4-(4-(2-hydroxyethyl)piperazin-1-yl)butanoate (14j, IAJD206). Compound **14j** was synthesized from compound **13g** (0.35 g, 0.50 mmol), 1-(2-hydroxyethyl)piperazine (72 mg, 0.55 mmol) and K_2CO_3 (0.10 g, 0.72 mmol) following a procedure similar to that used for the synthesis of compound **14a**. The title compound was obtained as a colorless oil (0.33 g, 89%). ^{1}H NMR (400 MHz, $CDCl_3$) δ 6.45 (br, 2 H, Ph*H*), 6.38 (t, 1 H, Ph*H*), 5.01 (s, 2 H, PhC*H*$_2$-), 3.91 (t, 4 H, PhOC*H*$_2$-), 3.61 (t, 2 H, -CH$_2$C*H*$_2$OH), 2.58–2.31 (m, 14 H, -N(C*H*$_2$C*H*$_2$)$_2$N-, -NC*H*$_2$CH$_2$C*H*$_2$COO- and -C*H*$_2$CH$_2$OH), 1.82 (m, 2 H, -OCOCH$_2$C*H*$_2$CH$_2$-), 1.73 (m, 4 H, PhOCH$_2$C*H*$_2$-), 1.44 (m, 4 H, PhOCH$_2$CH$_2$C*H*$_2$-), 1.27 (br, 42 H, PhOCH$_2$CH$_2$CH$_2$(C*H*$_2$)$_{13}$CH$_3$ and PhOCH$_2$CH$_2$CH$_2$(C*H*$_2$)$_8$CH$_3$), 0.89 (t, 6 H, -CH$_2$CH$_2$C*H*$_3$). ^{13}C NMR (101 MHz, $CDCl_3$) δ 173.3, 160.5, 138.1, 106.4, 100.9, 77.2, 68.1, 66.2, 64.6, 57.5, 57.5, 55.0, 52.8, 52.7, 45.8, 45.8, 32.3, 32.2, 31.9, 29.7, 29.7, 29.6, 29.6, 29.6, 29.5, 29.4, 26.1, 26.0, 22.7, 22.1, 22.1, 14.1. Purity by HPLC: 99+%. MALDI-TOF MS m/z of $[M + H]^+$ calculated for $C_{46}H_{85}N_2O_5$: 745.6; Found: 746.7.

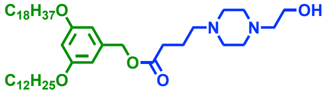

14k (IAJD207)

3-(Dodecyloxy)-5-(octadecyloxy)benzyl 4-(4-(2-hydroxyethyl)piperazin-1-yl)butanoate (14k, IAJD207). Compound **14k** was synthesized from compound **13g** (240 mg, 0.34 mmol), 1-(2-hydroxyethyl)piperazine (66 mg, 0.51 mmol) and K_2CO_3 (57 mg, 0.41 mmol) following a procedure similar to that used for the synthesis of compound 31a. The title compound was obtained as a colorless oil (100 mg, 40%). ^{1}H NMR (400 MHz, $CDCl_3$) δ 6.48 (s, 2 H, Ph*H*), 6.42 (t, 1 H, Ph*H*), 5.05 (s, 2 H, PhC*H*$_2$-), 3.94 (t, 4 H, PhOC*H*$_2$-), 3.64 (t, 2H, -CH$_2$C*H*$_2$OH), 2.63–2.36 (m, 14 H, -N(C*H*$_2$C*H*$_2$)$_2$N-, -NC*H*$_2$CH$_2$C*H*$_2$COO- and -C*H*$_2$CH$_2$OH), 1.86 (m, 2 H, -OCOCH$_2$C*H*$_2$CH$_2$-), 1.78 (m, 4 H, PhOCH$_2$C*H*$_2$-), 1.43 (m, 4 H, PhOCH$_2$CH$_2$C*H*$_2$-), 1.29 (br, 44 H, PhOCH$_2$CH$_2$CH$_2$(C*H*$_2$)$_8$CH$_3$ and PhOCH$_2$CH$_2$CH$_2$(C*H*$_2$)$_{14}$CH$_3$), 0.90 (t, 6 H, -CH$_2$CH$_2$C*H*$_3$). ^{13}C NMR (101 MHz, $CDCl_3$) δ 173.28, 160.46, 138.06, 106.43, 100.89, 77.35, 77.03, 76.72, 68.10, 66.16, 59.29, 57.62, 57.50, 52.95, 52.83, 32.21, 31.93, 29.71, 29.67, 29.64, 29.63, 29.61, 29.59, 29.41, 29.37, 29.36, 29.27, 26.07, 22.69, 22.09, 14.12. Purity by HPLC: 99+%. MALDI-TOF MS m/z of $[M + H]^+$ calculated for $C_{47}H_{87}N_2O_5$: 759.7; Found: 760.1.

3.3 Synthesis of Tetradecane-Based IAJDs with 3,5-Nonsymmetric Alkyl Chains

Scheme S3. Synthesis of Tetradecane-Based IAJDs with 3,5-Nonsymmetric Alkyl Chains

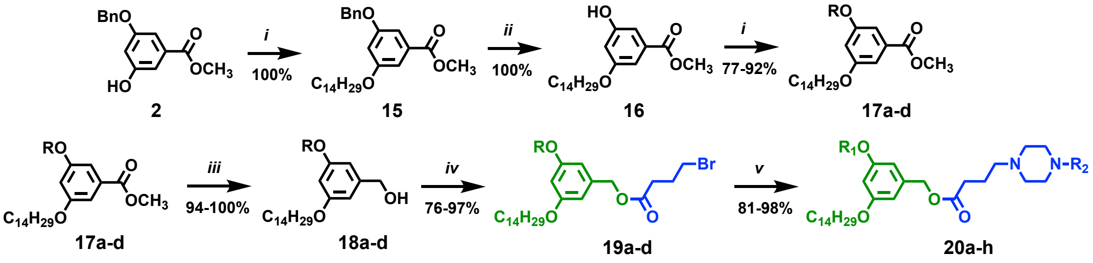

17-19a: R = 2-ethylhexyl
17-19b: R = C_8H_{17}
17-19c: R = $C_{10}H_{21}$
17-19d: R = $C_{18}H_{37}$

20a (IAJD208): R_1 = 2-ethylhexyl; R_2 = CH_3
20b (IAJD209): R_1 = 2-ethylhexyl; R_2 = CH_2CH_2OH
20c (IAJD210): R_1 = C_8H_{17}; R_2 = CH_3
20d (IAJD211): R_1 = C_8H_{17}; R_2 = CH_2CH_2OH
20e (IAJD212): R_1 = $C_{10}H_{21}$; R_2 = CH_3
20f (IAJD213): R_1 = $C_{10}H_{21}$; R_2 = CH_2CH_2OH
20g (IAJD214): R_1 = $C_{18}H_{37}$; R_2 = CH_3
20h (IAJD215): R_1 = $C_{18}H_{37}$; R_2 = CH_2CH_2OH

Reagents and conditions: (*i*) RBr, K_2CO_3, DMF, 120 °C, 2 h; (*ii*) H_2, Pd/C, DCM, MeOH, 12 h; (*iii*) $LiAlH_4$, THF, 0–23 °C, 1 h; (*iv*) DCC, DPTS, DCM, 12 h; (*v*) K_2CO_3, MeCN, 95 °C, 3 h.

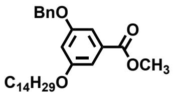

15

Methyl 3-(benzyloxy)-5-(tetradecyloxy)benzoate (15). A mixture of compound **2** (4.00 g, 15.49 mmol, 1 equiv), 1-bromotetradecane (4.72 g, 17.04 mmol, 1.1 equiv), K_2CO_3 (4.28 g, 30.98 mmol, 2 equiv) and DMF (20 mL) was heated to 120 °C and stirred under N_2 atmosphere for 2 h. The reaction mixture was cooled to 23 °C and poured into ice/water (150 mL). The resulted white precipitates in the solution were filtered and collected. Then the precipitates were recrystallized from minimum acetone to afford the title compound as a white solid (5.77 g, 82%). ^{1}H NMR (400 MHz, $CDCl_3$) δ 7.49–7.31 (m, 5 H, Ph*H*), 7.27 (br, 1 H, Ph*H*), 7.20 (br, 1 H, Ph*H*), 6.73 (t, 1 H, Ph*H*), 5.08 (s, 2 H, PhC*H*$_2$O-), 3.97 (t, 2 H, PhOC*H*$_2$-), 3.90 (s, 3 H, PhCOOC*H*$_3$), 1.77 (m, 2 H, $PhOCH_2CH_2CH_2(CH_2)_{10}CH_3$), 1.45 (m, 2 H, $PhOCH_2CH_2CH_2(CH_2)_{10}CH_3$), 1.29 (m, 20 H, $PhOCH_2CH_2CH_2(CH_2)_{10}CH_3$), 0.89 (t, 3 H, $PhO(CH_2)_{13}CH_3$). ^{13}C NMR (101 MHz, $CDCl_3$) δ 167.0, 160.4, 159.9, 136.7, 132.1, 128.7, 128.2, 127.7, 108.3, 108.0, 107.1, 70.4, 68.5, 52.3, 33.0, 32.1, 29.8, 29.7, 29.6, 29.5, 29.3, 28.9, 28.3, 26.1, 22.8, 14.2. Mp = 55 °C.

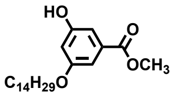

16

Methyl 3-hydroxy-5-(tetradecyloxy)benzoate (16). Compound **15** (5.77 g, 12.70 mmol) was dissolved in a mixture of DCM (30 mL) and methanol (15 mL). Then Pd/C (0.30 g, 5 wt%) was added to the solution

and the flask was evacuated and filled with hydrogen for three times. Afterwards, the mixture was stirred at 23 °C under hydrogen atmosphere for 12 h. The reaction mixture was filtered through Celite, and the filter cake was washed with DCM. Evaporation of the solvent yielded the title compound as a white solid (4.56 g, 98%). ^{1}H NMR (400 MHz, $CDCl_3$) δ 7.14 (d, 2 H, Ph*H*), 6.61 (t, 1 H, Ph*H*), 5.59 (s, 1 H, PhO*H*), 3.95 (t, 2 H, PhOCH_2-), 3.90 (s, 3 H, PhCOOCH_3), 1.75 (m, 2 H, $PhOCH_2CH_2CH_2(CH_2)_{10}CH_3$), 1.45 (m, 2 H, $PhOCH_2CH_2CH_2(CH_2)_{10}CH_3$), 1.27 (m, 20 H, $PhOCH_2CH_2CH_2(CH_2)_{10}CH_3$), 0.88 (t, 3 H, $PhO(CH_2)_{13}CH_3$). ^{13}C NMR (101 MHz, $CDCl_3$) δ 167.2, 160.6, 156.9, 132.1, 109.1, 108.0, 107.2, 77.4, 68.5, 52.5, 34.2, 33.0, 32.1, 29.8, 29.8, 29.7, 29.7, 29.7, 29.6, 29.5, 29.3, 28.9, 28.3, 26.1, 22.8, 14.3. Mp = 94 °C.

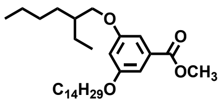

17a

Methyl 3-((2-ethylhexyl)oxy)-5-(tetradecyloxy)benzoate (17a). A mixture of compound **16** (0.70 g, 1.92 mmol, 1 equiv), 2-ethylhexyl bromide (0.41 g, 2.11 mmol, 1.1 equiv), K_2CO_3 (0.53 g, 3.84 mmol, 2 equiv) and DMF (15 mL) was heated to 80 °C and stirred under N_2 atmosphere for 12 h. The reaction mixture was cooled to 23 °C and DMF was removed under reduced pressure. Then water (20 mL) was added, and the resulted mixture was extracted with DCM (20 mL) for three times. The organic phases were collected and dried over anhydrous $MgSO_4$. After filtration, the obtained filtrate was concentrated to around 3 mL and purified by column chromatography (SiO_2) with hexane/EtOAc = 20/1 as the eluent to give the title compound as a light-yellow oil (0.58 g, 64%). ^{1}H NMR (400 MHz, $CDCl_3$) δ 7.16 (br, 2 H, Ph*H*), 6.64 (t, 1 H, Ph*H*), 3.97 (t, 2 H, $PhOCH_2(CH_2)_{12}CH_3$), 3.90 (s, 3 H, PhCOOCH_3), 3.85 (m, 2 H, $PhOCH_2CH(CH_2CH_3)$-), 1.84–1.68 (m, 3 H, $PhOCH_2CH_2(CH_2)_{11}CH_3$ and $PhOCH_2CH(CH_2CH_3)(CH_2)_3CH_3$), 1.55–1.22 (m, 30 H, $PhOCH_2CH_2(CH_2)_{11}CH_3$ and $PhOCH_2CH(CH_2CH_3)(CH_2)_3CH_3$), 1.00–0.76 (m, 9 H, $PhO(CH_2)_{13}CH_3$ and $PhOCH_2CH(CH_2CH_3)(CH_2)_3CH_3$). ^{13}C NMR (101 MHz, $CDCl_3$) δ 167.3, 167.1, 160.6, 160.3, 157.0, 132.1, 131.9, 109.1, 107.9, 107.8, 107.8, 107.6, 107.2, 106.8, 70.9, 68.5, 52.4, 52.3, 30.7, 29.8, 29.8, 29.7, 29.7, 29.5, 29.5, 29.3, 29.3, 29.3, 29.2, 26.2, 26.1, 24.0, 23.2, 22.8, 14.2, 14.2, 11.2.

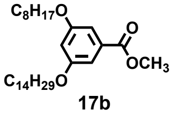

17b

Methyl 3-(octyloxy)-5-(tetradecyloxy)benzoate (17b). A mixture of compound **16** (0.70 g, 1.92 mmol, 1 equiv), 1-bromooctane (0.41 g, 2.11 mmol, 1.1 equiv), K_2CO_3 (0.53 g, 3.84 mmol, 2 equiv) and DMF

(10 mL) was heated to 120 °C and stirred under N_2 atmosphere for 2 h. The reaction mixture was cooled to 23 °C and poured into ice/water (150 mL). The resulted white precipitates in the solution were filtered and collected. Then the precipitates were recrystallized from minimum acetone to afford the title compound as a white solid (0.58 g, 64%). ^{1}H NMR (400 MHz, $CDCl_3$) δ 7.16 (br, 2 H, Ph*H*), 6.64 (t, 1 H, Ph*H*), 3.96 (t, 4 H, $PhOC\mathit{H}_2CH_2CH_2$-), 3.89 (s, 3 H, $PhCOOC\mathit{H}_3$), 1.77 (m, 4 H, $PhOCH_2C\mathit{H}_2CH_2$-), 1.44 (m, 4 H, $PhOCH_2CH_2C\mathit{H}_2$-), 1.28 (m, 28 H, $PhOCH_2CH_2CH_2(C\mathit{H}_2)_4CH_3$ and $PhOCH_2CH_2CH_2(C\mathit{H}_2)_{10}CH_3$), 0.88 (t, 6 H, $PhO(CH_2)_7C\mathit{H}_3$ and $PhO(CH_2)_{13}C\mathit{H}_3$). ^{13}C NMR (101 MHz, $CDCl_3$) δ 167.1, 160.3, 132.0, 107.8, 106.7, 68.5, 52.3, 32.1, 32.0, 29.8, 29.8, 29.7, 29.7, 29.5, 29.5, 29.4, 29.3, 26.2, 22.8, 22.8, 14.3, 14.2. Mp = 42 °C.

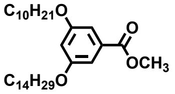

17c

Methyl 3-(decyloxy)-5-(tetradecyloxy)benzoate (17c). Compound **17c** was synthesized from compound **16** (0.70 g, 1.92 mmol, 1 equiv), 1-bromodecane (0.47 g, 2.11 mmol, 1.1 equiv) and K_2CO_3 (0.53 g, 3.84 mmol, 2 equiv) following a procedure similar to that used for the synthesis of compound **17b**. The title compound was obtained as a white solid (0.58 g, 60%). ^{1}H NMR (400 MHz, $CDCl_3$) δ 7.16 (br, 2 H, Ph*H*), 6.64 (t, 1 H, Ph*H*), 3.96 (t, 4 H, $PhOC\mathit{H}_2CH_2CH_2$-), 3.89 (s, 3 H, $PhCOOC\mathit{H}_3$), 1.77 (m, 4 H, $PhOCH_2C\mathit{H}_2CH_2$-), 1.45 (m, 4 H, $PhOCH_2CH_2C\mathit{H}_2$-), 1.28 (m, 32 H, $PhOCH_2CH_2CH_2(C\mathit{H}_2)_6CH_3$ and $PhOCH_2CH_2CH_2(C\mathit{H}_2)_{10}CH_3$), 0.88 (t, 6 H, $PhO(CH_2)_9C\mathit{H}_3$ and $PhO(CH_2)_{13}C\mathit{H}_3$). ^{13}C NMR (101 MHz, $CDCl_3$) δ 167.1, 160.3, 132.0, 107.8, 106.7, 77.4, 68.5, 52.3, 32.1, 32.0, 29.8, 29.8, 29.7, 29.7, 29.7, 29.5, 29.5, 29.3, 26.2, 22.8, 14.2. Mp = 52 °C.

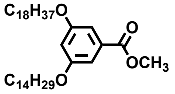

17d

Methyl 3-(octadecyloxy)-5-(tetradecyloxy)benzoate (17d). Compound **17d** was synthesized from compound **16** (0.70 g, 1.92 mmol, 1 equiv), 1-bromoctadecanene (0.70 g, 2.11 mmol, 1.1 equiv) and K_2CO_3 (0.53 g, 3.84 mmol, 2 equiv) following a procedure similar to that used for the synthesis of compound **17b**. The title compound was obtained as a white solid (0.73 g, 62%). ^{1}H NMR (400 MHz, $CDCl_3$) δ 7.16 (br, 2 H, Ph*H*), 6.64 (t, 1 H, Ph*H*), 3.96 (t, 4 H, $PhOC\mathit{H}_2CH_2CH_2$-), 3.89 (s, 3 H, $PhCOOC\mathit{H}_3$), 1.77 (m, 4 H, $PhOCH_2C\mathit{H}_2CH_2$-), 1.45 (m, 4 H, $PhOCH_2CH_2C\mathit{H}_2$-), 1.27 (m, 48 H, $PhOCH_2CH_2CH_2(C\mathit{H}_2)_{14}CH_3$ and $PhOCH_2CH_2CH_2(C\mathit{H}_2)_{10}CH_3$), 0.89 (t, 6 H, $PhO(CH_2)_{17}C\mathit{H}_3$ and

PhO(CH_2)$_{13}$CH_3). ^{13}C NMR (101 MHz, $CDCl_3$) δ 167.1, 160.3, 132.0, 107.8, 106.7, 68.5, 52.3, 34.1, 33.0, 32.1, 29.9, 29.8, 29.8, 29.8, 29.7, 29.7, 29.6, 29.5, 29.3, 28.9, 28.3, 26.2, 22.8, 14.3. Mp = 66 °C.

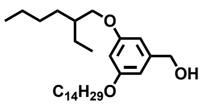

18a

(3-((2-Ethylhexyl)oxy)-5-(tetradecyloxy)phenyl)methanol (18a). Compound **17a** (0.55 g, 1.15 mmol, 1 equiv) was dissolved in 5 mL dry THF, which was added dropwise to a slurry of $LiAlH_4$ (44 mg, 1.23 mmol, 1 equiv) in dry THF (5 mL) at 0 °C under N_2 atmosphere. Afterwards, the resulted mixture was stirred at 23 °C for 1 h. Finally, the reaction was quenched by the successive addition of water (0.2 mL), 15% NaOH aqueous solution (0.2 mL) and water (1.0 mL). White precipitates were formed in the mixture and filtered out. The filtrate was dried over anhydrous $MgSO_4$ and filtered. The solvent was removed under reduced pressure to afford the title compound as a colorless oil (0.38 g, 74%). ^{1}H NMR (400 MHz, $CDCl_3$) δ 6.50 (d, 2 H, Ph*H*), 6.38 (t, 1 H, Ph*H*), 4.61 (s, 2 H, PhCH$_2$O*H*), 3.93 (t, 2 H, PhOCH_2(CH$_2$)$_{12}$CH$_3$), 3.82 (m, 2 H, PhOCH_2CH(CH$_2$CH$_3$)-), 1.72 (m, 3 H, PhOCH$_2$CH_2(CH$_2$)$_{11}$CH$_3$ and PhOCH$_2$C*H*(CH$_2$CH$_3$)(CH$_2$)$_3$CH$_3$), 1.53–1.24 (m, 30 H, PhOCH$_2$CH$_2$(CH_2)$_{11}$CH$_3$ and PhOCH$_2$CH(CH_2CH$_3$)(CH_2)$_3$CH$_3$), 0.90 (m, 9 H, PhO(CH$_2$)$_{13}$CH_3 and PhOCH$_2$CH(CH$_2$CH_3)(CH$_2$)$_3$CH_3). ^{13}C NMR (101 MHz, $CDCl_3$) δ 160.7, 160.7, 143.3, 105.3, 105.2, 105.1, 100.7, 77.4, 70.7, 68.2, 65.5, 62.9, 39.5, 32.1, 30.7, 30.0, 29.8, 29.8, 29.8, 29.7, 29.7, 29.5, 29.5, 29.4, 29.2, 26.2, 24.0, 23.2, 22.8, 14.2, 14.2, 11.2.

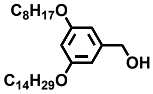

18b

(3-(Octyloxy)-5-(tetradecyloxy)phenyl)methanol (18b). Compound **18b** was synthesized from compound **17b** (0.51 g, 1.07 mmol, 1 equiv) and $LiAlH_4$ (41 mg, 1.07 mmol, 1 equiv) following a procedure similar to that used for the synthesis of compound **18a**. The title compound was obtained as a colorless oil (0.46 g, 96%). ^{1}H NMR (400 MHz, $CDCl_3$) δ 6.49 (br, 2 H, Ph*H*), 6.37 (t, 1 H, Ph*H*), 4.60 (s, 2 H, PhCH_2OH), 3.93 (t, 4 H, PhOCH_2CH$_2$CH$_2$-), 1.75 (m, 4 H, PhOCH$_2$CH_2CH$_2$-), 1.43 (m, 4 H, PhOCH$_2$CH$_2$CH_2-), 1.29 (m, 28 H, PhOCH$_2$CH$_2$CH$_2$(CH_2)$_4$CH$_3$ and PhOCH$_2$CH$_2$CH$_2$(CH_2)$_{10}$CH$_3$), 0.88 (t, 6 H, PhO(CH$_2$)$_7$CH_3 and PhO(CH$_2$)$_{13}$CH_3). ^{13}C NMR (101 MHz, $CDCl_3$) δ 160.7, 160.7, 143.3, 143.3, 105.2, 100.7, 68.2, 65.6, 65.5, 32.1, 32.0, 29.8, 29.8, 29.8, 29.7, 29.7, 29.5, 29.5, 29.5, 29.4, 26.2, 22.8, 14.2.

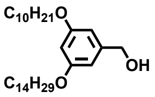

18c

(3-(Decyloxy)-5-(tetradecyloxy)phenyl)methanol (18c). Compound **18c** was synthesized from compound **17c** (0.69 g, 1.45 mmol, 1 equiv) and $LiAlH_4$ (55 mg, 1.45 mmol, 1 equiv) following a procedure similar to that used for the synthesis of compound **18a**. The title compound was obtained as a colorless oil (0.52 g, 83%). ^{1}H NMR (400 MHz, $CDCl_3$) δ 6.49 (br, 2 H, Ph*H*), 6.37 (t, 1 H, Ph*H*), 4.60 (s, 2 H, $PhC\mathit{H}_2OH$), 3.93 (t, 4 H, $PhOC\mathit{H}_2CH_2CH_2$-), 1.76 (m, 4 H, $PhOCH_2C\mathit{H}_2CH_2$-), 1.44 (m, 4 H, $PhOCH_2CH_2C\mathit{H}_2$-), 1.29 (m, 32 H, $PhOCH_2CH_2CH_2(C\mathit{H}_2)_6CH_3$ and $PhOCH_2CH_2CH_2(C\mathit{H}_2)_{10}CH_3$), 0.88 (t, 6 H, $PhO(CH_2)_9C\mathit{H}_3$ and $PhO(CH_2)_{13}C\mathit{H}_3$). ^{13}C NMR (101 MHz, $CDCl_3$) δ 160.6, 160.6, 143.4, 105.2, 100.7, 77.5, 77.2, 76.8, 68.2, 65.5, 65.4, 32.1, 31.9, 29.8, 29.8, 29.7, 29.7, 29.5, 29.5, 29.4, 26.2, 22.8, 22.8, 22.8, 14.2, 14.2.

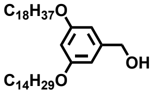

18d

(3-(Octadecyloxy)-5-(tetradecyloxy)phenyl)methanol (18d). Compound **18d** was synthesized from compound **17d** (0.83 g, 1.35 mmol, 1 equiv) and $LiAlH_4$ (51 mg, 1.35 mmol, 1 equiv) following a procedure similar to that used for the synthesis of compound **18a**. The title compound was obtained as a white solid (0.76 g, 95%). ^{1}H NMR (400 MHz, $CDCl_3$) δ 6.49 (br, 2 H, Ph*H*), 6.37 (t, 1 H, Ph*H*), 4.60 (s, 2 H, $PhC\mathit{H}_2OH$), 3.93 (t, 4 H, $PhOC\mathit{H}_2CH_2CH_2$-), 1.76 (m, 4 H, $PhOCH_2C\mathit{H}_2CH_2$-), 1.44 (m, 4 H, $PhOCH_2CH_2C\mathit{H}_2$-), 1.34 (m, 48 H, $PhOCH_2CH_2CH_2(C\mathit{H}_2)_{14}CH_3$ and $PhOCH_2CH_2CH_2(C\mathit{H}_2)_{10}CH_3$), 0.88 (t, 6 H, $PhO(CH_2)_{17}C\mathit{H}_3$ and $PhO(CH_2)_{13}C\mathit{H}_3$). ^{13}C NMR (101 MHz, $CDCl_3$) δ 160.7, 143.4, 105.2, 100.7, 68.2, 65.6, 32.1, 29.9, 29.8, 29.8, 29.7, 29.6, 29.5, 29.5, 29.4, 26.2, 22.8, 14.3. Mp = 52 °C.

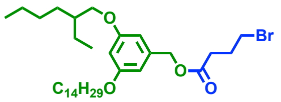

19a

3-((2-Ethylhexyl)oxy)-5-(tetradecyloxy)benzyl 4-bromobutanoate (19a). Compound **18a** (0.52 g, 1.16 mmol, 1 equiv), 4-bromobutyric acid (0.21 g, 1.27 mmol, 1.1 equiv) and DPTS (0.38 g, 1.27 mmol, 1.1 equiv) were dissolved in 10 mL DCM. DCC (0.48 g, 2.32 mmol, 2 equiv) was added into the above mixture. The reaction mixture was stirred at 23 °C for 12 h. Afterwards, the resulted precipitates (urea) were filtered out from the reaction mixture. The filtrate was concentrated to around 3 mL and purified by column chromatography (SiO_2) with hexane/DCM = 1/1 as the mobile phase to give the title compound as a colorless oil (0.66 g, 100%). ^{1}H NMR (400 MHz, $CDCl_3$) δ 6.47 (d, 2 H, Ph*H*), 6.42 (t, 1 H, Ph*H*),

5.05 (s, 2 H, $PhCH_2OCO$), 3.93 (t, 2 H, $PhOCH_2(CH_2)_{12}CH_3$), 3.82 (m, 2 H,$PhOCH_2CH(CH_2CH_3)$-), 3.47 (t, 2 H, -$OCOCH_2CH_2CH_2Br$), 2.57 (t, 2 H, -$OCOCH_2CH_2CH_2Br$), 2.21 (m, 2 H, -$OCOCH_2CH_2CH_2Br$), 1.83–1.67 (m, 3 H, $PhOCH_2CH_2(CH_2)_{11}CH_3$ and $PhOCH_2CH(CH_2CH_3)(CH_2)_3CH_3$), 1.57–1.21 (m, 30 H, $PhOCH_2CH_2(CH_2)_{11}CH_3$ and $PhOCH_2CH(CH_2CH_3)(CH_2)_3CH_3$), 0.91 (m, 9 H, $PhO(CH_2)_{13}CH_3$ and $PhOCH_2CH(CH_2CH_3)(CH_2)_3CH_3$). ^{13}C NMR (101 MHz, $CDCl_3$) δ 172.4, 160.9, 160.6, 137.9, 106.6, 106.5, 106.4, 101.2, 70.7, 68.2, 66.6, 39.5, 32.7, 32.6, 32.1, 30.7, 29.8, 29.8, 29.7, 29.7, 29.6, 29.5, 29.5, 29.4, 29.2, 27.9, 26.2, 24.0, 23.2, 22.8, 14.2, 14.2, 11.2.

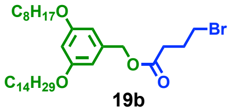

19b

3-(Octyloxy)-5-(tetradecyloxy)benzyl 4-bromobutanoate (19b). Compound **19b** was synthesized from compound **18b** (0.35 g, 0.78 mmol, 1 equiv), 4-bromobutyric acid (0.14 g, 0.86 mmol, 1.1 equiv), DPTS (0.25 g, 0.86 mmol, 1.1 equiv) and DCC (0.32 g, 1.56 mmol, 2 equiv) following a procedure similar to that used for the synthesis of compound **19a**. The title compound was obtained as a colorless oil (0.35 g, 79%). ^{1}H NMR (400 MHz, $CDCl_3$) δ 6.47 (br, 2 H, Ph*H*), 6.41 (t, 1 H, Ph*H*), 5.05 (s, 2 H, $PhCH_2OCO$), 3.93 (t, 4 H, $PhOCH_2CH_2CH_2$-), 3.46 (t, 2 H, -$OCOCH_2CH_2CH_2Br$), 2.56 (t, 2 H, -$OCOCH_2CH_2CH_2Br$), 2.20 (m, 2 H, -$OCOCH_2CH_2CH_2Br$), 1.77 (m, 4 H, $PhOCH_2CH_2CH_2$-), 1.44 (m, 4 H, $PhOCH_2CH_2CH_2$-), 1.28 (m, 28 H, $PhOCH_2CH_2CH_2(CH_2)_4CH_3$ and $PhOCH_2CH_2CH_2(CH_2)_{10}CH_3$), 0.89 (t, 6 H, $PhO(CH_2)_7CH_3$ and $PhO(CH_2)_{13}CH_3$). ^{13}C NMR (101 MHz, $CDCl_3$) δ 172.4, 160.6, 137.9, 106.5, 101.2, 77.4, 68.2, 66.6, 32.8, 32.6, 32.1, 32.0, 30.5, 29.8, 29.8, 29.8, 29.7, 29.7, 29.7, 29.5, 29.4, 27.9, 26.2, 22.8, 14.3.

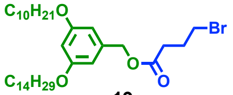

19c

3-(Decyloxy)-5-(tetradecyloxy)benzyl 4-bromobutanoate (19c). Compound **19c** was synthesized from compound **18c** (0.35 g, 0.74 mmol, 1 equiv), 4-bromobutyric acid (0.135 g, 0.81 mmol, 1.1 equiv), DPTS (0.24 g, 0.81 mmol, 1.1 equiv) and DCC (0.30 g, 1.47 mmol, 2 equiv) following a procedure similar to that used for the synthesis of compound **19a**. The title compound was obtained as a colorless oil (0.35 g, 80%). ^{1}H NMR (400 MHz, $CDCl_3$) δ 6.46 (br, 2 H, Ph*H*), 6.40 (t, 1 H, Ph*H*), 5.05 (s, 2 H, $PhCH_2OCO$), 3.93 (t, 4 H, $PhOCH_2CH_2CH_2$-), 3.47 (t, 2 H, -$OCOCH_2CH_2CH_2Br$), 2.57 (t, 2 H, -$OCOCH_2CH_2CH_2Br$), 2.20 (m, 2 H, -$OCOCH_2CH_2CH_2Br$), 1.76 (m, 4 H, $PhOCH_2CH_2CH_2$-), 1.44 (m, 4 H, $PhOCH_2CH_2CH_2$-), 1.27 (m, 32 H, $PhOCH_2CH_2CH_2(CH_2)_6CH_3$ and $PhOCH_2CH_2CH_2(CH_2)_{10}CH_3$), 0.88 (t, 6 H,

$PhO(CH_2)_9C\mathit{H}_3$ and $PhO(CH_2)_{13}C\mathit{H}_3$). ^{13}C NMR (101 MHz, $CDCl_3$) δ 172.4, 160.6, 137.9, 106.5, 101.2, 68.2, 66.5, 32.8, 32.6, 32.1, 32.0, 29.8, 29.8, 29.8, 29.7, 29.7, 29.7, 29.5, 29.5, 29.5, 29.4, 27.9, 26.2, 22.8, 14.3.

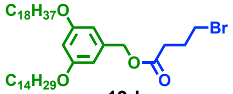

19d

3-(Octadecyloxy)-5-(tetradecyloxy)benzyl 4-bromobutanoate (19d). Compound **19d** was synthesized from compound **18d** (0.35 g, 0.59 mmol, 1 equiv), 4-bromobutyric acid (0.11 g, 0.65 mmol, 1.1 equiv), DPTS (0.19 g, 0.65 mmol, 1.1 equiv) and DCC (0.25 g, 1.19 mmol, 2 equiv) following a procedure similar to that used for the synthesis of compound **19a**. The title compound was obtained as a colorless oil (0.42 g, 100%). ^{1}H NMR (400 MHz, $CDCl_3$) δ 6.46 (br, 2 H, Ph*H*), 6.40 (t, 1 H, Ph*H*), 5.05 (s, 2 H, $PhC\mathit{H}_2OCO$), 3.93 (t, 4 H, $PhOC\mathit{H}_2CH_2CH_2$-), 3.47 (t, 2 H, $-OCOCH_2CH_2C\mathit{H}_2Br$), 2.57 (t, 2 H, $-OCOC\mathit{H}_2CH_2CH_2Br$), 2.20 (m, 2 H, $-OCOCH_2C\mathit{H}_2CH_2Br$), 1.76 (m, 4 H, $PhOCH_2C\mathit{H}_2CH_2$-), 1.44 (m, 4 H, $PhOCH_2CH_2C\mathit{H}_2$-), 1.28 (m, 48 H, $PhOCH_2CH_2CH_2(C\mathit{H}_2)_{14}CH_3$ and $PhOCH_2CH_2CH_2(C\mathit{H}_2)_{10}CH_3$), 0.89 (t, 6 H, $PhO(CH_2)_{17}C\mathit{H}_3$ and $PhO(CH_2)_{13}C\mathit{H}_3$). ^{13}C NMR (101 MHz, $CDCl_3$) δ 172.4, 160.6, 137.9, 106.5, 101.2, 77.4, 68.2, 66.6, 32.8, 32.6, 32.1, 32.0, 30.5, 29.8, 29.8, 29.8, 29.7, 29.7, 29.7, 29.5, 29.5, 29.5, 29.4, 27.9, 26.2, 22.8, 14.3.

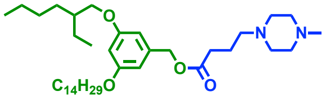

20a (IAJD208)

3-((2-Ethylhexyl)oxy)-5-(tetradecyloxy)benzyl 4-(4-methylpiperazin-1-yl)butanoate (20a, IAJD208). A mixture of compound **19a** (0.33 g, 0.55 mmol, 1.0 equiv), 1-methylpiperazine (61 mg, 0.61 mmol, 1.1 equiv), K_2CO_3 (92 mg, 0.66 mmol, 1.2 equiv) and MeCN (20 mL) was heated to 95 °C and stirred for 3 h. The reaction mixture was cooled to 23 °C and MeCN was removed under reduced pressure. Then water (20 mL) was added, and the resulted mixture was extracted with DCM (20 mL) for three times. The organic phases were collected and dried over anhydrous $MgSO_4$. After filtration, the obtained filtrate was concentrated to around 3 mL and purified by column chromatography (SiO_2) with DCM/MeOH = 30/1 and 15/1 as the eluent. Then the obtained product was dissolved in DCM (20 mL), which was washed by $NaHCO_3$ solution (2%, 20 mL). The aqueous phase was extracted by DCM (20 mL) for another two times. The organic phases were combined and dried over anhydrous $MgSO_4$. Filtration and evaporation of the solvent yielded the title compound as a colorless oil (0.28 g, 82%). ^{1}H NMR (400 MHz, $CDCl_3$) δ 6.44 (br, 2 H, Ph*H*), 6.37 (t, 1 H, Ph*H*), 5.00 (s, 2 H, $PhC\mathit{H}_2$-), 3.89 (t, 4 H, $PhOC\mathit{H}_2$-), 3.77 (m, 2 H,

PhOCH_2CH(CH_2CH_3)-), 2.61–2.22 (m, 15 H, -N(CH_2CH_2)$_2$N-, -NCH_2CH$_2$CH_2COO- and -NCH_3), 1.81 (m, 2 H, -OCOCH$_2$CH_2CH$_2$-), 1.72 (m, 3 H, PhOCH$_2$CH_2(CH$_2$)$_{11}$CH$_3$ and PhOCH$_2$CH(CH$_2$CH$_3$)(CH$_2$)$_3$CH$_3$), 1.49–1.19 (m, 30 H, PhOCH$_2$CH$_2$(CH_2)$_{11}$CH$_3$ and PhOCH$_2$CH(CH_2CH$_3$)(CH_2)$_3$CH$_3$), 0.87 (m, 9 H, PhO(CH$_2$)$_{13}$CH_3 and PhOCH$_2$CH(CH$_2$CH_3)(CH$_2$)$_3$CH_3). ^{13}C NMR (101 MHz, CDCl$_3$) δ 173.3, 173.2, 160.7, 160.5, 138.1, 106.5, 106.4, 106.3, 100.9, 70.5, 68.1, 66.2, 57.5, 55.0, 52.7, 45.8, 39.4, 32.2, 31.9, 30.6, 29.7, 29.7, 29.7, 29.6, 29.6, 29.4, 29.4, 29.3, 29.1, 26.1, 23.9, 23.1, 22.7, 22.1, 14.1, 14.1, 11.1. Purity by HPLC: 99+%. MALDI-TOF MS m/z of [M + H]$^+$ calculated for $C_{38}H_{69}N_2O_4$: 617.5; Found: 617.8.

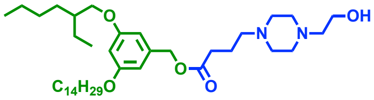

20b (IAJD209)

3-((2-Ethylhexyl)oxy)-5-(tetradecyloxy)benzyl 4-(4-(2-hydroxyethyl)piperazin-1-yl)butanoate (20b, IAJD209). Compound **20b** was synthesized from compound **19a** (0.19 g, 0.32 mmol), 1-(2-hydroxyethyl)piperazine (46 mg, 0.35 mmol) and K_2CO_3 (53 mg, 0.38 mmol) following a procedure similar to that used for the synthesis of compound **20a**. The title compound was obtained as a colorless oil (0.20 g, 95%). ^{1}H NMR (400 MHz, CDCl$_3$) δ 6.44 (br, 2 H, PhH), 6.37 (t, 1 H, PhH), 5.00 (s, 2 H, PhCH_2-), 3.90 (t, 4 H, PhOCH_2-), 3.78 (m, 2 H, PhOCH_2CH(CH$_2$CH$_3$)-), 3.61 (t, 2 H, -CH$_2$CH_2OH), 2.84–2.22 (m, 14 H, -N(CH_2CH_2)$_2$N-, -NCH_2CH$_2$CH_2COO- and -NCH_2-), 1.81 (m, 2 H, -OCOCH$_2$CH_2CH$_2$-), 1.72 (m, 3 H, PhOCH$_2$CH_2(CH$_2$)$_{11}$CH$_3$ and PhOCH$_2$CH(CH$_2$CH$_3$)(CH$_2$)$_3$CH$_3$), 1.52–1.10 (m, 30 H, PhOCH$_2$CH$_2$(CH_2)$_{11}$CH$_3$ and PhOCH$_2$CH(CH_2CH$_3$)(CH_2)$_3$CH$_3$), 0.87 (m, 9 H, PhO(CH$_2$)$_{13}$CH_3 and PhOCH$_2$CH(CH$_2$CH_3)(CH$_2$)$_3$CH_3). ^{13}C NMR (101 MHz, CDCl$_3$) δ 173.3, 173.2, 160.7, 160.5, 138.1, 106.5, 106.4, 106.3, 100.9, 70.5, 68.1, 66.2, 57.5, 55.0, 52.7, 45.8, 39.4, 32.2, 31.9, 30.6, 29.7, 29.7, 29.7, 29.6, 29.6, 29.4, 29.4, 29.3, 29.1, 26.1, 23.9, 23.1, 22.7, 22.1, 14.1, 14.1, 11.1. Purity by HPLC: 99+%. MALDI-TOF MS m/z of [M + H]$^+$ calculated for $C_{39}H_{71}N_2O_5$: 647.5; Found: 647.9.

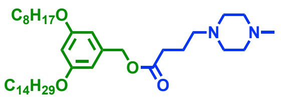

20c (IAJD210)

3-(Octyloxy)-5-(tetradecyloxy)benzyl 4-(4-methylpiperazin-1-yl)butanoate (20c, IAJD210). Compound **20c** was synthesized from compound **19b** (0.22 g, 0.35 mmol), 1-methylpiperazine (0.05g, 0.53 mmol) and K_2CO_3 (0.06 g, 0.42 mmol) following a procedure similar to that used for the synthesis of compound 31a. The title compound was obtained as a colorless oil (200 mg, 92%). ^{1}H NMR (400 MHz,

$CDCl_3$) δ 6.46 (p, 2 H, PhH), 6.40 (t, 1 H, PhH), 5.03 (s, 2 H, $PhCH_2$-), 3.92 (t, 4 H, $PhOCH_2$-), 2.73–2.24 (m, 15 H, -$N(CH_2CH_2)_2N$-, -$NCH_2CH_2CH_2COO$- and -NCH_3), 1.84 (m, 2 H, -$OCOCH_2CH_2CH_2$-), 1.76 (m, 4 H, $PhOCH_2CH_2$-), 1.42 (m, 4 H, $PhOCH_2CH_2CH_2$-), 1.26 (br, 28 H, $PhOCH_2CH_2CH_2(CH_2)_4CH_3$ and $PhOCH_2CH_2CH_2(CH_2)_{10}CH_3$), 0.88 (t, 6 H, -$CH_2CH_2CH_3$). ^{13}C NMR (101 MHz, $CDCl_3$) δ 173.2, 160.4, 138.1, 106.4, 100.9, 77.4, 77.1, 76.6, 68.1, 66.1, 57.4, 54.9, 52.6, 45.7, 45.7, 32.2, 32.2, 31.9, 31.8, 29.7, 29.7, 29.67, 29.6, 29.6, 29.6, 29.4, 29.3, 29.2, 29.2, 26.1, 22.7, 22.6, 22.0, 14.1. Purity by HPLC: 99+%. MALDI-TOF MS m/z of $[M + H]^+$ calculated for $C_{38}H_{69}N_2O_4$: 617.5; Found: 618.5.

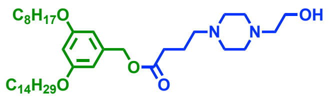

20d (IAJD211)

3-(Octyloxy)-5-(tetradecyloxy)benzyl 4-(4-(2-hydroxyethyl)piperazin-1-yl)butanoate (20d, IAJD211). Compound **20d** was synthesized from compound **19b** (0.18 g, 0.29 mmol), 1-(2-hydroxyethyl)piperazine (0.06 g, 0.53 mmol) and K_2CO_3 (0.05 g, 0.42 mmol) following a procedure similar to that used for the synthesis of compound 31a. The title compound was obtained as a colorless oil (180 mg, 94%). ^1H NMR (400 MHz, $CDCl_3$) δ 6.48 (s, 2 H, PhH), 6.41 (t, 1 H, PhH), 5.05 (s, 2 H, $PhCH_2$-), 3.94 (t, 4 H, $PhOCH_2$-), 3.68 (t, 2H, -CH_2CH_2OH), 2.72–2.35 (m, 14 H, -$N(CH_2CH_2)_2N$-, -$NCH_2CH_2CH_2COO$- and -CH_2CH_2OH), 1.87 (m, 2 H, -$OCOCH_2CH_2CH_2$-), 1.78 (m, 4 H, $PhOCH_2CH_2$-), 1.45 (m, 4 H, $PhOCH_2CH_2CH_2$-), 1.28 (br, 28 H, $PhOCH_2CH_2CH_2(CH_2)_4CH_3$ and $PhOCH_2CH_2CH_2(CH_2)_{10}CH_3$), 0.90 (t, 6 H, -$CH_2CH_2CH_3$). ^{13}C NMR (101 MHz, $CDCl_3$) δ 173.2, 160.5, 138.1, 106.5, 100.9, 77.4, 77.0, 76.7, 68.1, 68.0, 66.2, 59.4, 57.4, 57.4, 52.8, 52.5, 32.1, 31.9, 29.7, 29.7, 29.6, 29.6, 29.4, 29.4, 29.3, 29.2, 26.1, 22.7, 21.9, 14.1. Purity by HPLC: 99+%. MALDI-TOF MS m/z of $[M + H]^+$ calculated for $C_{39}H_{71}N_2O_5$: 648.0; Found: 648.3.

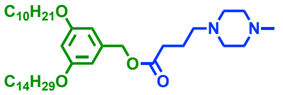

20e (IAJD212)

3-(Decyloxy)-5-(tetradecyloxy)benzyl 4-(4-methylpiperazin-1-yl)butanoate (20e, IAJD212). Compound **20e** was synthesized from compound **19c** (0.18 g, 0.28 mmol), 1-methylpiperazine (31 mg, 0.31 mmol) and K_2CO_3 (58 mg, 0.42 mmol) following a procedure similar to that used for the synthesis of compound **20a**. The title compound was obtained as a colorless oil (0.20 g, 99%). ^1H NMR (400 MHz, $CDCl_3$) δ 6.46 (br, 2 H, PhH), 6.39 (t, 1 H, PhH), 5.03 (s, 2 H, $PhCH_2$-), 3.91 (t, 4 H, $PhOCH_2$-), 2.63–2.30 (m, 15 H, -$N(CH_2CH_2)_2N$-, -$NCH_2CH_2CH_2COO$- and -NCH_3), 1.86 (m, 2 H, -$OCOCH_2CH_2CH_2$-), 1.77 (m, 4 H, $PhOCH_2CH_2$-), 1.45 (m, 4 H, $PhOCH_2CH_2CH_2$-), 1.27 (br, 32 H,

$PhOCH_2CH_2CH_2(CH_2)_6CH_3$ and $PhOCH_2CH_2CH_2(CH_2)_{10}CH_3$), 0.88 (t, 6 H, $-CH_2CH_2CH_3$). ^{13}C NMR (101 MHz, $CDCl_3$) δ 173.3, 160.5, 138.1, 106.4, 100.9, 68.1, 66.2, 57.4, 54.9, 52.4, 45.7, 32.2, 31.9, 29.7, 29.7, 29.7, 29.6, 29.6, 29.4, 29.4, 29.3, 26.1, 22.7, 22.1, 14.1. Purity by HPLC: 99+%. MALDI-TOF MS m/z of $[M + H]^+$ calculated for $C_{40}H_{73}N_2O_4$: 645.6; Found: 646.3.

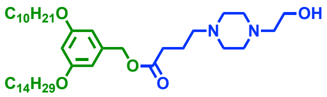

20f (IAJD213)

3-(Decyloxy)-5-(tetradecyloxy)benzyl 4-(4-(2-hydroxyethyl)piperazin-1-yl)butanoate (20f, IAJD213). Compound **20f** was synthesized from compound **19c** (0.18 g, 0.28 mmol), 1-(2-hydroxyethyl)piperazine (41 mg, 0.31 mmol) and K_2CO_3 (58 mg, 0.42 mmol) following a procedure similar to that used for the synthesis of compound **20a**. The title compound was obtained as a colorless oil (0.17 g, 89%). ^1H NMR (400 MHz, $CDCl_3$) δ 6.46 (br, 2 H, Ph*H*), 6.39 (t, 1 H, Ph*H*), 5.02 (s, 2 H, $PhCH_2$-), 3.91 (t, 4 H, $PhOCH_2$-), 3.62 (t, 2 H, $-CH_2CH_2OH$), 2.61–2.30 (m, 14 H, $-N(CH_2CH_2)_2N$-, -$NCH_2CH_2CH_2COO$- and $-NCH_2$-), 1.84 (m, 2 H, $-OCOCH_2CH_2CH_2$-), 1.73 (m, 4 H, $PhOCH_2CH_2$-), 1.43 (m, 4 H, $PhOCH_2CH_2CH_2$-), 1.28 (br, 32 H, $PhOCH_2CH_2CH_2(CH_2)_6CH_3$ and $PhOCH_2CH_2CH_2(CH_2)_{10}CH_3$), 0.89 (t, 6 H, $-CH_2CH_2CH_3$). ^{13}C NMR (101 MHz, $CDCl_3$) δ 173.3, 160.5, 138.1, 106.4, 100.9, 77.2, 68.1, 66.2, 59.3, 57.6, 57.5, 53.0, 52.5, 32.2, 31.9, 29.7, 29.7, 29.6, 29.6, 29.4, 29.4, 29.3, 26.1, 22.7, 22.1, 14.1. Purity by HPLC: 99+%. MALDI-TOF MS m/z of $[M + H]^+$ calculated for $C_{41}H_{75}N_2O_5$: 675.6; Found: 676.5.

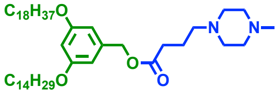

20g (IAJD214)

3-(Octadecyloxy)-5-(tetradecyloxy)benzyl 4-(4-methylpiperazin-1-yl)butanoate (20g, IAJD214). Compound **20g** was synthesized from compound **19d** (0.22 g, 0.31 mmol), 1-methylpiperazine (34 mg, 0.34 mmol) and K_2CO_3 (52 mg, 0.37 mmol) following a procedure similar to that used for the synthesis of compound **20a**. The title compound was obtained as a colorless oil (0.23 g, 98%). ^1H NMR (400 MHz, $CDCl_3$) δ 6.44 (br, 2 H, Ph*H*), 6.37 (t, 1 H, Ph*H*), 5.01 (s, 2 H, $PhCH_2$-), 3.90 (t, 4 H, $PhOCH_2$-), 2.59–2.26 (m, 15 H, $-N(CH_2CH_2)_2N$-, $-NCH_2CH_2CH_2COO$- and $-NCH_3$), 1.82 (m, 2 H, $-OCOCH_2CH_2CH_2$-), 1.73 (m, 4 H, $PhOCH_2CH_2$-), 1.42 (m, 4 H, $PhOCH_2CH_2CH_2$-), 1.26 (br, 48 H, $PhOCH_2CH_2CH_2(CH_2)_{14}CH_3$ and $PhOCH_2CH_2CH_2(CH_2)_{10}CH_3$), 0.86 (t, 6 H, $-CH_2CH_2CH_3$). ^{13}C NMR (101 MHz, $CDCl_3$) δ 173.2, 160.4, 138.1, 106.4, 100.9, 68.1, 66.1, 57.4, 54.9, 52.7, 45.7, 32.2, 31.9, 29.7,

29.7, 29.7, 29.6, 29.6, 29.4, 29.4, 29.3, 26.1, 22.7, 22.1, 14.1. Purity by HPLC: 99+%. MALDI-TOF MS m/z of $[M + H]^+$ calculated for $C_{48}H_{89}N_2O_4$: 757.7; Found: 757.5.

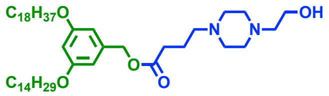

20h (IAJD215)

3-(Octadecyloxy)-5-(tetradecyloxy)benzyl 4-(4-(2-hydroxyethyl)piperazin-1-yl)butanoate (20h, IAJD215). Compound **20h** was synthesized from compound **19d** (0.22 g, 0.31 mmol), 1-(2-hydroxyethyl)piperazine (44 mg, 0.34 mmol) and K_2CO_3 (52 mg, 0.37 mmol) following a procedure similar to that used for the synthesis of compound **20a**. The title compound was obtained as a colorless oil (0.24 g, 100%). ^1H NMR (400 MHz, $CDCl_3$) δ 6.46 (br, 2 H, Ph*H*), 6.39 (t, 1 H, Ph*H*), 5.02 (s, 2 H, PhC*H*$_2$-), 3.91 (t, 4 H, PhOC*H*$_2$-), 3.62 (t, 2 H, -CH$_2$C*H*$_2$OH), 2.61–2.28 (m, 14 H, -N(C*H*$_2$C*H*$_2$)$_2$N-, -NC*H*$_2$CH$_2$C*H*$_2$COO- and -NC*H*$_2$-), 1.84 (m, 2 H, -OCOCH$_2$C*H*$_2$CH$_2$-), 1.74 (m, 4 H, PhOCH$_2$C*H*$_2$-), 1.43 (m, 4 H, PhOCH$_2$CH$_2$C*H*$_2$-), 1.28 (br, 48 H, PhOCH$_2$CH$_2$CH$_2$(C*H*$_2$)$_{14}$CH$_3$ and PhOCH$_2$CH$_2$CH$_2$(C*H*$_2$)$_{10}$CH$_3$), 0.87 (t, 6 H, -CH$_2$CH$_2$C*H*$_3$). ^{13}C NMR (101 MHz, $CDCl_3$) δ 173.3, 160.5, 138.1, 106.4, 100.9, 77.2, 68.1, 66.2, 59.3, 57.6, 57.5, 53.0, 52.8, 32.2, 31.9, 29.7, 29.7, 29.6, 29.6, 29.4, 29.4, 29.3, 26.1, 22.7, 22.1, 14.1. Purity by HPLC: 99+%. MALDI-TOF MS m/z of $[M + H]^+$ calculated for $C_{49}H_{91}N_2O_5$: 787.7; Found: 788.5.

3.4 Synthesis of Hexadecane-Based IAJDs with 3,5-Nonsymmetric Alkyl Chains

Scheme S4. Synthesis of Hexadecane-Based IAJDs with 3,5-Nonsymmetric Alkyl Chains

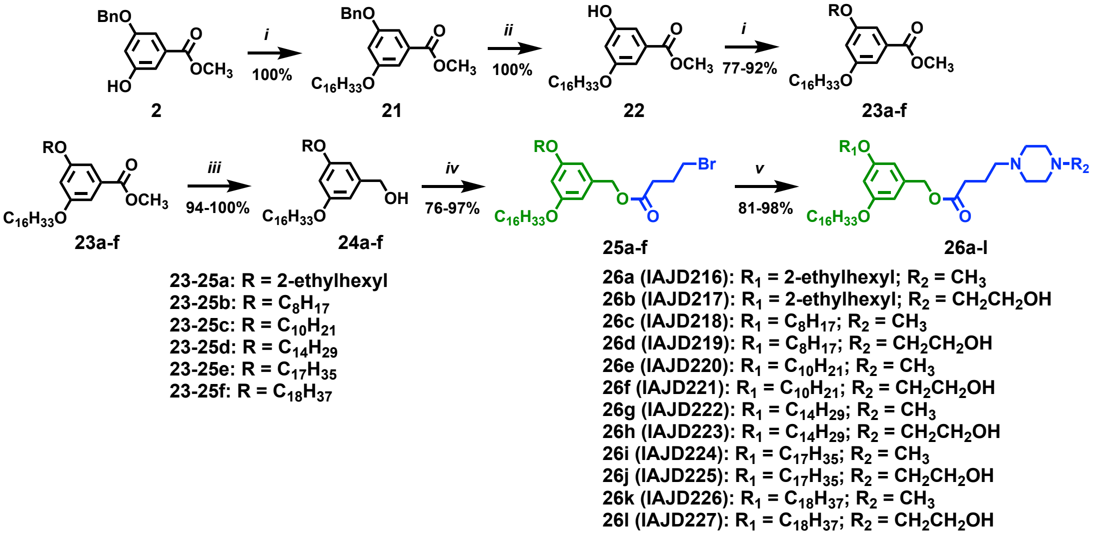

Reagents and conditions: (*i*) RBr/ROTs, K_2CO_3, DMF, 120 °C, 2 h; (*ii*) H_2, Pd/C, DCM, MeOH, 12 h; (*iii*) $LiAlH_4$, THF, 0–23 °C, 1 h; (*iv*) DCC, DPTS, DCM, 12 h; (*v*) K_2CO_3, MeCN, 95 °C, 3 h.

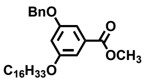

21

Methyl 3-(benzyloxy)-5-(hexadecyloxy)benzoate (21). A mixture of compound **2** (2.40 g, 9.29 mmol, 1 equiv), 1-bromohexadecane (3.10 g, 10.22 mmol, 1.1 equiv), K_2CO_3 (2.57 g, 18.58 mmol, 2 equiv) and DMF (15 mL) was heated to 120 °C and stirred under N_2 atmosphere for 2 h. The reaction mixture was cooled to 23 °C and poured into ice/water (150 mL). The resulted white precipitates in the solution were filtered and collected. Then the precipitates were recrystallized from minimum acetone to afford the title compound as a white solid (4.36 g, 97%). ^{1}H NMR (400 MHz, $CDCl_3$) δ 7.39 (m, 5 H, Ph*H*), 7.27 (br, 1 H, Ph*H*), 7.20 (br, 1 H, Ph*H*), 6.73 (t, 1 H, Ph*H*), 5.08 (s, 2 H, PhC*H*$_2$O-), 3.97 (t, 2 H, PhOC*H*$_2$-), 3.91 (s, 3 H, PhCOOC*H*$_3$), 1.78 (m, 2 H, PhOCH$_2$C*H*$_2$CH$_2$(CH$_2$)$_{12}$CH$_3$), 1.44 (m, 2 H, PhOCH$_2$CH$_2$C*H*$_2$(CH$_2$)$_{12}$CH$_3$), 1.29 (m, 24 H, PhOCH$_2$CH$_2$CH$_2$(C*H*$_2$)$_{12}$CH$_3$), 0.89 (t, 3 H, PhO(CH$_2$)$_{15}$C*H*$_3$). ^{13}C NMR (101 MHz, $CDCl_3$) δ 167.0, 160.3, 159.9, 136.7, 132.1, 128.7, 128.2, 127.7, 108.3, 108.0, 107.1, 77.4, 70.4, 68.5, 52.3, 32.1, 29.8, 29.8, 29.7, 29.7, 29.5, 29.3, 26.4, 26.1, 22.8, 14.2. Mp = 61 °C.

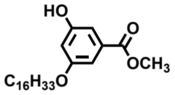

22

Methyl 3-(hexadecyloxy)-5-hydroxybenzoate (22). Compound **21** (3.28 g, 6.42 mmol) was dissolved in a mixture of DCM (20 mL) and methanol (10 mL). Then Pd/C (0.16 g, 5 wt%) was added to the solution and the flask was evacuated and filled with hydrogen for three times. Afterwards, the mixture was stirred at 23 °C under hydrogen atmosphere for 12 h. The reaction mixture was filtered through Celite, and the filter cake was washed with DCM. Evaporation of the solvent yielded the title compound as a white solid (2.70 g, 100%). ^{1}H NMR (400 MHz, $CDCl_3$) δ 7.13 (d, 2 H, Ph*H*), 6.63 (t, 1 H, Ph*H*), 3.95 (t, 2 H, PhOC*H*$_2$-), 3.89 (s, 3 H, PhCOOC*H*$_3$), 1.76 (m, 2 H, PhOCH$_2$C*H*$_2$CH$_2$(CH$_2$)$_{12}$CH$_3$), 1.46 (m, 2 H, PhOCH$_2$CH$_2$C*H*$_2$(CH$_2$)$_{12}$CH$_3$), 1.28 (m, 24 H, PhOCH$_2$CH$_2$CH$_2$(C*H*$_2$)$_{12}$CH$_3$), 0.88 (t, 3 H, PhO(CH$_2$)$_{15}$C*H*$_3$). ^{13}C NMR (101 MHz, $CDCl_3$) δ 167.0, 160.4, 157.0, 131.9, 109.0, 107.7, 107.1, 68.4, 52.2, 31.9, 29.7, 29.7, 29.6, 29.6, 29.4, 29.4, 29.2, 26.0, 22.7, 14.2. Mp = 97 °C.

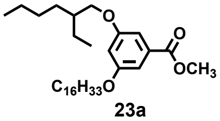

23a

Methyl 3-((2-ethylhexyl)oxy)-5-(hexadecyloxy)benzoate (23a). A mixture of compound **22** (0.50 g, 1.27 mmol, 1 equiv), 2-ethylhexyl bromide (0.27 g, 1.40 mmol, 1.1 equiv), K_2CO_3 (0.35 g, 2.54 mmol, 2

equiv) and DMF (15 mL) was heated to 80 °C and stirred under N_2 atmosphere for 12 h. The reaction mixture was cooled to 23 °C and DMF was removed under reduced pressure. Then water (20 mL) was added, and the resulted mixture was extracted with DCM (20 mL) for three times. The organic phases were collected and dried over anhydrous $MgSO_4$. After filtration, the obtained filtrate was concentrated to around 3 mL and purified by column chromatography (SiO_2) with hexane/EtOAc = 20/1 as the eluent to give the title compound as a light-yellow oil (0.30 g, 47%). ^{1}H NMR (400 MHz, $CDCl_3$) δ 7.16 (br, 2 H, Ph*H*), 6.64 (t, 1 H, Ph*H*), 3.97 (t, 2 H, $PhOC\mathit{H}_2(CH_2)_{14}CH_3$), 3.90 (s, 3 H, $PhCOOC\mathit{H}_3$), 3.85 (m, 2 H, $PhOC\mathit{H}_2CH(CH_2CH_3)$-), 1.86–1.68 (m, 3 H, $PhOCH_2C\mathit{H}_2(CH_2)_{13}CH_3$ and $PhOCH_2C\mathit{H}(CH_2CH_3)(CH_2)_3CH_3$), 1.55–1.18 (m, 34 H, $PhOCH_2CH_2(C\mathit{H}_2)_{13}CH_3$ and $PhOCH_2CH(C\mathit{H}_2CH_3)(C\mathit{H}_2)_3CH_3$), 0.90 (m, 9 H, $PhO(CH_2)_{15}C\mathit{H}_3$ and $PhOCH_2CH(CH_2C\mathit{H}_3)(CH_2)_3C\mathit{H}_3$). ^{13}C NMR (101 MHz, $CDCl_3$) δ 167.2, 160.6, 160.3, 131.9, 107.9, 107.6, 106.7, 70.9, 68.5, 52.3, 52.3, 39.5, 32.1, 30.7, 29.9, 29.8, 29.8, 29.7, 29.5, 29.4, 29.2, 26.2, 24.0, 23.2, 22.8, 14.3, 14.2, 11.2.

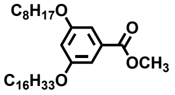

23b

Methyl 3-(hexadecyloxy)-5-(octyloxy)benzoate (23b). A mixture of compound **22** (0.50 g, 1.27 mmol, 1 equiv), 1-bromooctane (0.31 g, 1.40 mmol, 1.1 equiv), K_2CO_3 (0.35 g, 2.54 mmol, 2 equiv) and DMF (15 mL) was heated to 120 °C and stirred under N_2 atmosphere for 2 h. The reaction mixture was cooled to 23 °C and poured into ice/water (150 mL). The resulted white precipitates in the solution were filtered and collected. Then the precipitates were recrystallized from minimum acetone to afford the title compound as a white solid (0.45 g, 66%). ^{1}H NMR (400 MHz, $CDCl_3$) δ 7.16 (br, 2 H, Ph*H*), 6.63 (t, 1 H, Ph*H*), 3.96 (t, 4 H, $PhOC\mathit{H}_2CH_2CH_2$-), 3.89 (s, 3 H, $PhCOOC\mathit{H}_3$), 1.77 (m, 4 H, $PhOCH_2C\mathit{H}_2CH_2$-), 1.44 (m, 4 H, $PhOCH_2CH_2C\mathit{H}_2$-), 1.27 (m, 32 H, $PhOCH_2CH_2CH_2(C\mathit{H}_2)_4CH_3$ and $PhOCH_2CH_2CH_2(C\mathit{H}_2)_{12}CH_3$), 0.88 (t, 6 H, $PhO(CH_2)_7C\mathit{H}_3$ and $PhO(CH_2)_{15}C\mathit{H}_3$). ^{13}C NMR (101 MHz, $CDCl_3$) δ 167.1, 160.3, 132.0, 111.0, 107.8, 106.7, 77.4, 68.5, 68.1, 52.3, 32.1, 31.9, 29.9, 29.8, 29.8, 29.7, 29.7, 29.6, 29.5, 29.5, 29.4, 29.3, 26.3, 26.2, 22.8, 14.2. Mp = 52 °C.

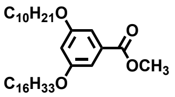

23c

Methyl 3-(decyloxy)-5-(hexadecyloxy)benzoate (23c). Compound **23c** was synthesized from compound **22** (0.50 g, 1.27 mmol, 1 equiv), 1-bromodecane (0.31 g, 1.40 mmol, 1.1 equiv) and K_2CO_3 (0.35 g, 2.54 mmol, 2 equiv) following a procedure similar to that used for the synthesis of compound **23b**. The title

compound was obtained as a white solid (0.55 g, 81%). ¹H NMR (400 MHz, $CDCl_3$) δ 7.16 (br, 2 H, Ph*H*), 6.63 (t, 1 H, Ph*H*), 3.96 (t, 4 H, PhOC$H_2$$CH_2$$CH_2$-), 3.89 (s, 3 H, PhCOOC$H_3$), 1.77 (m, 4 H, PhO$CH_2CH_2$$CH_2$-), 1.44 (m, 4 H, PhO$CH_2$$CH_2CH_2$-), 1.27 (m, 36 H, PhO$CH_2$$CH_2$$CH_2$(C$H_2$)$_6$$CH_3$ and PhO$CH_2$$CH_2$$CH_2$(C$H_2$)$_{12}$$CH_3$), 0.89 (t, 6 H, PhO($CH_2$)$_9CH_3$ and PhO(CH_2)$_{15}$CH_3). ¹³C NMR (101 MHz, $CDCl_3$) δ 167.1, 160.3, 131.9, 107.8, 106.7, 77.4, 68.4, 68.1, 52.3, 32.1, 32.0, 29.9, 29.8, 29.8, 29.7, 29.7, 29.7, 29.6, 29.5, 29.5, 29.3, 26.1, 22.8, 14.2. Mp = 60 °C.

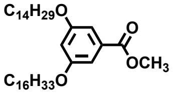

23d

Methyl 3-(hexadecyloxy)-5-(tetradecyloxy)benzoate (23d). Compound **23d** was synthesized from compound **22** (0.50 g, 1.27 mmol, 1 equiv), 1-bromotetradecanene (0.39 g, 1.40 mmol, 1.1 equiv) and K_2CO_3 (0.35 g, 2.54 mmol, 2 equiv) following a procedure similar to that used for the synthesis of compound **23b**. The title compound was obtained as a white solid (0.68 g, 91%). ¹H NMR (400 MHz, $CDCl_3$) δ 7.15 (br, 2 H, Ph*H*), 6.63 (t, 1 H, Ph*H*), 3.96 (t, 4 H, PhOC$H_2$$CH_2$$CH_2$-), 3.89 (s, 3 H, PhCOOC$H_3$), 1.77 (m, 4 H, PhO$CH_2CH_2$$CH_2$-), 1.46 (m, 4 H, PhO$CH_2$$CH_2CH_2$-), 1.27 (m, 44 H, PhO$CH_2$$CH_2$$CH_2$(C$H_2$)$_{10}$$CH_3$ and PhO$CH_2$$CH_2$$CH_2$(C$H_2$)$_{12}$$CH_3$), 0.89 (t, 6 H, PhO($CH_2$)$_{13}CH_3$ and PhO(CH_2)$_{15}$CH_3). ¹³C NMR (101 MHz, $CDCl_3$) δ 167.1, 160.3, 132.0, 107.8, 106.7, 77.4, 68.5, 52.3, 32.1, 29.8, 29.8, 29.7, 29.7, 29.5, 29.3, 26.2, 22.8, 14.3. Mp = 64 °C.

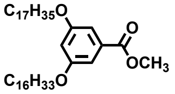

23e

Methyl 3-(heptadecyloxy)-5-(hexadecyloxy)benzoate (23e). Compound **23e** was synthesized from compound **22** (0.50 g, 1.27 mmol, 1 equiv), $C_{17}H_{35}OTs$ (0.58 g, 1.40 mmol, 1.1 equiv) and K_2CO_3 (0.35 g, 2.54 mmol, 2 equiv) following a procedure similar to that used for the synthesis of compound **23b**. The title compound was obtained as a white solid (0.75 g, 94%). ¹H NMR (400 MHz, $CDCl_3$) δ 7.15 (br, 2 H, Ph*H*), 6.64 (t, 1 H, Ph*H*), 3.96 (t, 4 H, PhOC$H_2$$CH_2$$CH_2$-), 3.89 (s, 3 H, PhCOOC$H_3$), 1.77 (m, 4 H, PhO$CH_2CH_2$$CH_2$-), 1.46 (m, 4 H, PhO$CH_2$$CH_2CH_2$-), 1.27 (m, 50 H, PhO$CH_2$$CH_2$$CH_2$(C$H_2$)$_{13}$$CH_3$ and PhO$CH_2$$CH_2$$CH_2$(C$H_2$)$_{12}$$CH_3$), 0.89 (t, 6 H, PhO($CH_2$)$_{16}CH_3$ and PhO(CH_2)$_{15}$CH_3). ¹³C NMR (101 MHz, $CDCl_3$) δ 167.1, 160.3, 132.0, 107.8, 106.7, 77.4, 68.5, 52.2, 32.1, 29.9, 29.8, 29.7, 29.7, 29.5, 29.3, 26.2, 22.8, 14.3. Mp = 69 °C.

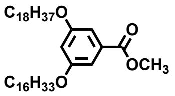

23f

Methyl 3-(hexadecyloxy)-5-(octadecyloxy)benzoate (23f). Compound **23f** was synthesized from compound **22** (0.50 g, 1.27 mmol, 1 equiv), 1-bromoctadecanene (0.47 g, 1.40 mmol, 1.1 equiv) and K_2CO_3 (0.35 g, 2.54 mmol, 2 equiv) following a procedure similar to that used for the synthesis of compound **23b**. The title compound was obtained as a white solid (0.71 g, 87%). ^{1}H NMR (400 MHz, $CDCl_3$) δ 7.15 (br, 2 H, Ph*H*), 6.63 (t, 1 H, Ph*H*), 3.96 (t, 4 H, $PhOCH_2CH_2CH_2$-), 3.89 (s, 3 H, $PhCOOCH_3$), 1.77 (m, 4 H, $PhOCH_2CH_2CH_2$-), 1.43 (m, 4 H, $PhOCH_2CH_2CH_2$-), 1.26 (m, 52 H, $PhOCH_2CH_2CH_2(CH_2)_{14}CH_3$ and $PhOCH_2CH_2CH_2(CH_2)_{12}CH_3$), 0.89 (t, 6 H, $PhO(CH_2)_{17}CH_3$ and $PhO(CH_2)_{15}CH_3$). ^{13}C NMR (101 MHz, $CDCl_3$) δ 167.1, 160.3, 131.9, 107.8, 106.7, 77.4, 68.5, 68.1, 52.3, 32.1, 29.9, 29.8, 29.8, 29.7, 29.7, 29.5, 29.3, 26.2, 25.7, 22.8, 14.2. Mp = 70 °C.

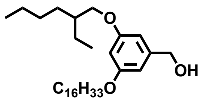

24a

(3-((2-Ethylhexyl)oxy)-5-(hexadecyloxy)phenyl)methanol (24a). Compound **23a** (0.30 g, 0.59 mmol, 1 equiv) was dissolved in 5 mL dry THF, which was added dropwise to a slurry of $LiAlH_4$ (23 mg, 0.59 mmol, 1 equiv) in dry THF (5 mL) at 0 °C under N_2 atmosphere. Afterwards, the resulted mixture was stirred at 23 °C for 1 h. Finally, the reaction was quenched by the successive addition of water (0.2 mL), 15% NaOH aqueous solution (0.2 mL) and water (1.0 mL). White precipitates were formed in the mixture and filtered out. The filtrate was dried over anhydrous $MgSO_4$ and filtered. The solvent was removed under reduced pressure to afford the title compound as a colorless oil (0.22 g, 78%). ^{1}H NMR (400 MHz, $CDCl_3$) δ 6.50 (d, 2 H, Ph*H*), 6.38 (t, 1 H, Ph*H*), 4.61 (s, 2 H, $PhCH_2OH$), 3.94 (t, 2 H, $PhOCH_2(CH_2)_{14}CH_3$), 3.83 (m, 2 H, $PhOCH_2CH(CH_2CH_3)$-), 1.72 (m, 3 H, $PhOCH_2CH_2(CH_2)_{13}CH_3$ and $PhOCH_2CH(CH_2CH_3)(CH_2)_3CH_3$), 1.51–1.17 (m, 34 H, $PhOCH_2CH_2(CH_2)_{13}CH_3$ and $PhOCH_2CH(CH_2CH_3)(CH_2)_3CH_3$), 0.91 (m, 9 H, $PhO(CH_2)_{15}CH_3$ and $PhOCH_2CH(CH_2CH_3)(CH_2)_3CH_3$). ^{13}C NMR (101 MHz, $CDCl_3$) δ 160.9, 160.7, 143.3, 105.3, 105.1, 100.7, 70.7, 68.2, 65.6, 62.9, 39.5, 32.1, 30.7, 29.8, 29.8, 29.8, 29.7, 29.5, 29.5, 29.4, 29.2, 26.2, 24.0, 23.2, 22.8, 14.2, 14.2, 11.2.

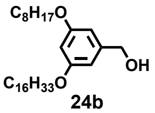

24b

(3-(Hexadecyloxy)-5-(octyloxy)phenyl)methanol (24b). Compound **24b** was synthesized from compound **23b** (0.45 g, 0.89 mmol, 1 equiv) and $LiAlH_4$ (34 mg, 0.89 mmol, 1 equiv) following a procedure similar to that used for the synthesis of compound **24a**. The title compound was obtained as a colorless oil (0.39 g, 92%). ^{1}H NMR (400 MHz, $CDCl_3$) δ 6.49 (br, 2 H, Ph*H*), 6.37 (t, 1 H, Ph*H*), 4.60 (s, 2 H, $PhCH_2OH$), 3.93 (t, 4 H, $PhOCH_2CH_2CH_2$-), 1.76 (m, 4 H, $PhOCH_2CH_2CH_2$-), 1.43 (m, 4 H, $PhOCH_2CH_2CH_2$-), 1.28 (m, 32 H, $PhOCH_2CH_2CH_2(CH_2)_4CH_3$ and $PhOCH_2CH_2CH_2(CH_2)_{12}CH_3$), 0.88 (t, 6 H, $PhO(CH_2)_7CH_3$ and $PhO(CH_2)_{15}CH_3$). ^{13}C NMR (101 MHz, $CDCl_3$) δ 160.7, 143.4, 105.2, 100.7, 77.4, 68.2, 65.5, 32.1, 32.0, 29.8, 29.8, 29.8, 29.7, 29.5, 29.5, 29.4, 29.4, 26.2, 22.8, 22.8, 14.2, 14.2.

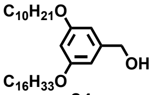

24c

(3-(Decyloxy)-5-(hexadecyloxy)phenyl)methanol (24c). Compound **24c** was synthesized from compound **23c** (0.45 g, 0.85 mmol, 1 equiv) and $LiAlH_4$ (32 mg, 0.85 mmol, 1 equiv) following a procedure similar to that used for the synthesis of compound **24a**. The title compound was obtained as a white solid (0.41 g, 96%). ^{1}H NMR (400 MHz, $CDCl_3$) δ 6.50 (br, 2 H, Ph*H*), 6.37 (t, 1 H, Ph*H*), 4.60 (s, 2 H, $PhCH_2OH$), 3.93 (t, 4 H, $PhOCH_2CH_2CH_2$-), 1.76 (m, 4 H, $PhOCH_2CH_2CH_2$-), 1.44 (m, 4 H, $PhOCH_2CH_2CH_2$-), 1.29 (m, 36 H, $PhOCH_2CH_2CH_2(CH_2)_6CH_3$ and $PhOCH_2CH_2CH_2(CH_2)_{12}CH_3$), 0.89 (t, 6 H, $PhO(CH_2)_9CH_3$ and $PhO(CH_2)_{15}CH_3$). ^{13}C NMR (101 MHz, $CDCl_3$) δ 160.7, 143.4, 105.2, 100.7, 77.4, 68.2, 65.5, 32.1, 32.0, 29.8, 29.8, 29.8, 29.7, 29.7, 29.5, 29.5, 29.5, 29.4, 26.2, 22.8, 14.2. Mp = 40 °C.

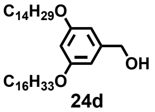

24d

(3-(Hexadecyloxy)-5-(tetradecyloxy)phenyl)methanol (24d). Compound **24d** was synthesized from compound **23d** (0.55 g, 0.93 mmol, 1 equiv) and $LiAlH_4$ (35 mg, 0.93 mmol, 1 equiv) following a procedure similar to that used for the synthesis of compound **24a**. The title compound was obtained as a white solid (0.50 g, 95%). ^{1}H NMR (400 MHz, $CDCl_3$) δ 6.49 (br, 2 H, Ph*H*), 6.37 (t, 1 H, Ph*H*), 4.60 (s, 2 H, $PhCH_2OH$), 3.93 (t, 4 H, $PhOCH_2CH_2CH_2$-), 1.76 (m, 4 H, $PhOCH_2CH_2CH_2$-), 1.44 (m, 4 H, $PhOCH_2CH_2CH_2$-), 1.29 (m, 44 H, $PhOCH_2CH_2CH_2(CH_2)_{10}CH_3$ and $PhOCH_2CH_2CH_2(CH_2)_{12}CH_3$), 0.88

(t, 6 H, $PhO(CH_2)_{13}C\mathit{H}_3$ and $PhO(CH_2)_{15}C\mathit{H}_3$). ^{13}C NMR (101 MHz, $CDCl_3$) δ 160.6, 143.4, 105.2, 100.7, 77.4, 68.2, 65.4, 32.1, 29.8, 29.8, 29.8, 29.7, 29.7, 29.5, 29.5, 29.4, 26.2, 22.8, 14.2. Mp = 47 °C.

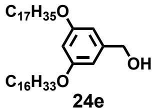

24e

(3-(Heptadecyloxy)-5-(hexadecyloxy)phenyl)methanol (24e). Compound **24e** was synthesized from compound **23e** (0.72 g, 1.14 mmol, 1 equiv) and $LiAlH_4$ (43 mg, 1.14 mmol, 1 equiv) following a procedure similar to that used for the synthesis of compound **24a**. The title compound was obtained as a white solid (0.67 g, 97%). ^{1}H NMR (400 MHz, $CDCl_3$) δ 6.49 (br, 2 H, Ph*H*), 6.37 (t, 1 H, Ph*H*), 4.59 (s, 2 H, $PhC\mathit{H}_2OH$), 3.93 (t, 4 H, $PhOC\mathit{H}_2CH_2CH_2$-), 1.76 (m, 4 H, $PhOCH_2C\mathit{H}_2CH_2$-), 1.44 (m, 4 H, $PhOCH_2CH_2C\mathit{H}_2$-), 1.27 (m, 50 H, $PhOCH_2CH_2CH_2(C\mathit{H}_2)_{13}CH_3$ and $PhOCH_2CH_2CH_2(C\mathit{H}_2)_{12}CH_3$), 0.89 (t, 6 H, $PhO(CH_2)_{16}C\mathit{H}_3$ and $PhO(CH_2)_{15}C\mathit{H}_3$). ^{13}C NMR (101 MHz, $CDCl_3$) δ 160.7, 143.4, 105.2, 100.7, 77.4, 68.2, 65.4, 32.1, 29.8, 29.8, 29.8, 29.7, 29.6, 29.5, 29.4, 26.2, 22.8, 14.3. Mp = 56 °C.

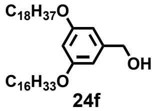

24f

(3-(Hexadecyloxy)-5-(octadecyloxy)phenyl)methanol (24f). Compound **24f** was synthesized from compound **23f** (0.70 g, 1.09 mmol, 1 equiv) and $LiAlH_4$ (41 mg, 1.09 mmol, 1 equiv) following a procedure similar to that used for the synthesis of compound **24a**. The title compound was obtained as a white solid (0.56 g, 83%). ^{1}H NMR (400 MHz, $CDCl_3$) δ 6.50 (br, 2 H, Ph*H*), 6.38 (t, 1 H, Ph*H*), 4.61 (s, 2 H, $PhC\mathit{H}_2OH$), 3.93 (t, 4 H, $PhOC\mathit{H}_2CH_2CH_2$-), 1.76 (m, 4 H, $PhOCH_2C\mathit{H}_2CH_2$-), 1.44 (m, 4 H, $PhOCH_2CH_2C\mathit{H}_2$-), 1.27 (m, 52 H, $PhOCH_2CH_2CH_2(C\mathit{H}_2)_{14}CH_3$ and $PhOCH_2CH_2CH_2(C\mathit{H}_2)_{12}CH_3$), 0.89 (t, 6 H, $PhO(CH_2)_{17}C\mathit{H}_3$ and $PhO(CH_2)_{13}C\mathit{H}_3$). ^{13}C NMR (101 MHz, $CDCl_3$) δ 160.7, 143.4, 125.6, 105.2, 100.7, 68.2, 65.5, 32.1, 30.5, 29.9, 29.8, 29.8, 29.7, 29.6, 29.5, 29.4, 26.2, 22.8, 14.3. Mp = 58 °C.

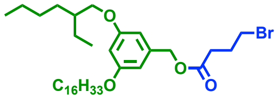

25a

3-((2-Ethylhexyl)oxy)-5-(hexadecyloxy)benzyl 4-bromobutanoate (25a). Compound **24a** (0.22 g, 0.46 mmol, 1 equiv), 4-bromobutyric acid (0.09 g, 0.51 mmol, 1.1 equiv) and DPTS (0.15 g, 0.51 mmol, 1.1 equiv) were dissolved in 8 mL DCM. DCC (0.19 g, 0.92 mmol, 2 equiv) was added into the above mixture. The reaction mixture was stirred at 23 °C for 12 h. Afterwards, the resulted precipitates (urea) were filtered out from the reaction mixture. The filtrate was concentrated to around 3 mL and purified by column chromatography (SiO_2) with hexane/DCM = 1/1 as the mobile phase to give the title compound as a

colorless oil (0.30 g, 100%). ^{1}H NMR (400 MHz, CDCl$_3$) δ 6.47 (d, 2 H, Ph*H*), 6.41 (t, 1 H, Ph*H*), 5.05 (s, 2 H, PhC*H*$_2$OCO), 3.93 (t, 2 H, PhOC*H*$_2$(CH$_2$)$_{14}$CH$_3$), 3.82 (m, 2 H, PhOC*H*$_2$CH(CH$_2$CH$_3$)-), 3.47 (t, 2 H, -OCOCH$_2$CH$_2$C*H*$_2$Br), 2.57 (t, 2 H, -OCOC*H*$_2$CH$_2$CH$_2$Br), 2.21 (m, 2 H, -OCOCH$_2$C*H*$_2$CH$_2$Br), 1.81–1.71 (m, 3 H, PhOCH$_2$C*H*$_2$(CH$_2$)$_{13}$CH$_3$ and PhOCH$_2$C*H*(CH$_2$CH$_3$)(CH$_2$)$_3$CH$_3$), 1.55–1.20 (m, 34 H, PhOCH$_2$CH$_2$(C*H*$_2$)$_{13}$CH$_3$ and PhOCH$_2$CH(C*H*$_2$CH$_3$)(C*H*$_2$)$_3$CH$_3$), 0.91 (m, 9 H, PhO(CH$_2$)$_{15}$C*H*$_3$ and PhOCH$_2$CH(CH$_2$C*H*$_3$)(CH$_2$)$_3$C*H*$_3$). ^{13}C NMR (101 MHz, CDCl$_3$) δ 172.4, 160.9, 160.6, 137.9, 106.6, 106.4, 101.2, 70.7, 68.2, 66.6, 39.5, 32.7, 32.6, 32.1, 30.7, 30.5, 29.8, 29.8, 29.8, 29.7, 29.7, 29.6, 29.5, 29.4, 29.2, 27.9, 26.2, 24.0, 23.2, 22.8, 14.3, 14.2, 11.3.

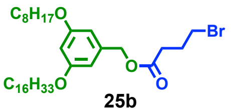

25b

3-(Hexadecyloxy)-5-(octyloxy)benzyl 4-bromobutanoate (25b). Compound **25b** was synthesized from compound **24b** (0.39 g, 0.82 mmol, 1 equiv), 4-bromobutyric acid (0.15 g, 0.90 mmol, 1.1 equiv), DPTS (0.27 g, 0.90 mmol, 1.1 equiv) and DCC (0.34 g, 1.64 mmol, 2 equiv) following a procedure similar to that used for the synthesis of compound **25a**. The title compound was obtained as a colorless oil (0.42 g, 80%). ^{1}H NMR (400 MHz, CDCl$_3$) δ 6.46 (br, 2 H, Ph*H*), 6.40 (t, 1 H, Ph*H*), 5.04 (s, 2 H, PhC*H*$_2$OCO), 3.93 (t, 4 H, PhOC*H*$_2$CH$_2$CH$_2$-), 3.47 (t, 2 H, -OCOCH$_2$CH$_2$C*H*$_2$Br), 2.57 (t, 2 H, -OCOC*H*$_2$CH$_2$CH$_2$Br), 2.20 (m, 2 H, -OCOCH$_2$C*H*$_2$CH$_2$Br), 1.77 (m, 4 H, PhOCH$_2$C*H*$_2$CH$_2$-), 1.44 (m, 4 H, PhOCH$_2$CH$_2$C*H*$_2$-), 1.28 (m, 32 H, PhOCH$_2$CH$_2$CH$_2$(C*H*$_2$)$_4$CH$_3$ and PhOCH$_2$CH$_2$CH$_2$(C*H*$_2$)$_{12}$CH$_3$), 0.89 (t, 6 H, PhO(CH$_2$)$_7$C*H*$_3$ and PhO(CH$_2$)$_{15}$C*H*$_3$). ^{13}C NMR (101 MHz, CDCl$_3$) δ 172.5, 160.6, 137.9, 106.5, 101.2, 77.4, 68.3, 66.6, 32.8, 32.6, 32.1, 32.0, 29.8, 29.8, 29.8, 29.7, 29.5, 29.5, 29.4, 27.9, 26.2, 22.8, 22.8, 14.3, 14.2.

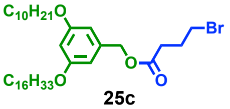

25c

3-(Decyloxy)-5-(hexadecyloxy)benzyl 4-bromobutanoate (25c). Compound **25c** was synthesized from compound **24c** (0.44 g, 0.87 mmol, 1 equiv), 4-bromobutyric acid (0.16 g, 0.96 mmol, 1.1 equiv), DPTS (0.28 g, 0.96 mmol, 1.1 equiv) and DCC (0.36 g, 1.74 mmol, 2 equiv) following a procedure similar to that used for the synthesis of compound **25a**. The title compound was obtained as a colorless oil (0.46 g, 81%). ^{1}H NMR (400 MHz, CDCl$_3$) δ 6.46 (br, 2 H, Ph*H*), 6.40 (t, 1 H, Ph*H*), 5.05 (s, 2 H, PhC*H*$_2$OCO), 3.93 (t, 4 H, PhOC*H*$_2$CH$_2$CH$_2$-), 3.47 (t, 2 H, -OCOCH$_2$CH$_2$C*H*$_2$Br), 2.57 (t, 2 H, -OCOC*H*$_2$CH$_2$CH$_2$Br), 2.20 (m, 2 H, -OCOCH$_2$C*H*$_2$CH$_2$Br), 1.76 (m, 4 H, PhOCH$_2$C*H*$_2$CH$_2$-), 1.44 (m, 4 H, PhOCH$_2$CH$_2$C*H*$_2$-),

1.27 (m, 36 H, $PhOCH_2CH_2CH_2(CH_2)_6CH_3$ and $PhOCH_2CH_2CH_2(CH_2)_{12}CH_3$), 0.88 (t, 6 H, $PhO(CH_2)_9CH_3$ and $PhO(CH_2)_{15}CH_3$). ^{13}C NMR (101 MHz, $CDCl_3$) δ 172.4, 160.6, 138.0, 106.5, 101.2, 77.4, 68.3, 66.6, 32.8, 32.6, 32.1, 32.0, 29.8, 29.8, 29.8, 29.7, 29.7, 29.5, 29.5, 29.5, 29.4, 27.9, 26.2, 22.8, 14.3.

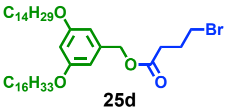

25d

3-(Hexadecyloxy)-5-(tetradecyloxy)benzyl 4-bromobutanoate (25d). Compound **25d** was synthesized from compound **24d** (0.50 g, 0.89 mmol, 1 equiv), 4-bromobutyric acid (0.16 g, 0.98 mmol, 1.1 equiv), DPTS (0.29 g, 0.98 mmol, 1.1 equiv) and DCC (0.37 g, 1.78 mmol, 2 equiv) following a procedure similar to that used for the synthesis of compound **25a**. The title compound was obtained as a colorless oil (0.38 g, 60%). ^{1}H NMR (400 MHz, $CDCl_3$) δ 6.46 (br, 2 H, Ph*H*), 6.40 (t, 1 H, Ph*H*), 5.05 (s, 2 H, $PhCH_2OCO$), 3.93 (t, 4 H, $PhOCH_2CH_2CH_2$-), 3.47 (t, 2 H, -$OCOCH_2CH_2CH_2Br$), 2.57 (t, 2 H, -$OCOCH_2CH_2CH_2Br$), 2.20 (m, 2 H, -$OCOCH_2CH_2CH_2Br$), 1.76 (m, 4 H, $PhOCH_2CH_2CH_2$-), 1.44 (m, 4 H, $PhOCH_2CH_2CH_2$-), 1.26 (m, 44 H, $PhOCH_2CH_2CH_2(CH_2)_{10}CH_3$ and $PhOCH_2CH_2CH_2(CH_2)_{12}CH_3$), 0.88 (t, 6 H, $PhO(CH_2)_{13}CH_3$ and $PhO(CH_2)_{15}CH_3$). ^{13}C NMR (101 MHz, $CDCl_3$) δ 172.4, 160.6, 137.9, 106.5, 101.2, 68.2, 66.6, 32.8, 32.6, 32.1, 29.8, 29.8, 29.8, 29.7, 29.7, 29.6, 29.5, 29.4, 27.9, 26.2, 22.8, 14.3.

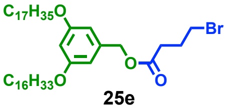

25e

3-(Heptadecyloxy)-5-(hexadecyloxy)benzyl 4-bromobutanoate (25e). Compound **25e** was synthesized from compound **24e** (0.67 g, 1.11 mmol, 1 equiv), 4-bromobutyric acid (0.20 g, 1.22 mmol, 1.1 equiv), DPTS (0.36 g, 1.22 mmol, 1.1 equiv) and DCC (0.46 g, 2.22 mmol, 2 equiv) following a procedure similar to that used for the synthesis of compound **25a**. The title compound was obtained as a colorless oil (0.72 g, 86%). ^{1}H NMR (400 MHz, $CDCl_3$) δ 6.46 (br, 2 H, Ph*H*), 6.40 (t, 1 H, Ph*H*), 5.04 (s, 2 H, $PhCH_2OCO$), 3.93 (t, 4 H, $PhOCH_2CH_2CH_2$-), 3.47 (t, 2 H, -$OCOCH_2CH_2CH_2Br$), 2.57 (t, 2 H, -$OCOCH_2CH_2CH_2Br$), 2.20 (m, 2 H, -$OCOCH_2CH_2CH_2Br$), 1.76 (m, 4 H, $PhOCH_2CH_2CH_2$-), 1.45 (m, 4 H, $PhOCH_2CH_2CH_2$-), 1.27 (m, 50 H, $PhOCH_2CH_2CH_2(CH_2)_{13}CH_3$ and $PhOCH_2CH_2CH_2(CH_2)_{12}CH_3$), 0.88 (t, 6 H, $PhO(CH_2)_{16}CH_3$ and $PhO(CH_2)_{15}CH_3$). ^{13}C NMR (101 MHz, $CDCl_3$) δ 172.4, 160.6, 138.0, 106.5, 101.2, 68.2, 66.6, 32.8, 32.6, 32.1, 29.9, 29.8, 29.8, 29.7, 29.6, 29.5, 29.4, 27.9, 26.2, 22.8, 14.3.

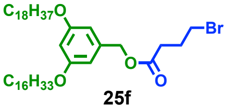

25f

3-(Hexadecyloxy)-5-(octadecyloxy)benzyl 4-bromobutanoate (25f). Compound **25f** was synthesized from compound **24f** (0.56 g, 0.91 mmol, 1 equiv), 4-bromobutyric acid (0.17 g, 1.00 mmol, 1.1 equiv), DPTS (0.29 g, 1.00 mmol, 1.1 equiv) and DCC (0.38 g, 1.82 mmol, 2 equiv) following a procedure similar to that used for the synthesis of compound **25a**. The title compound was obtained as a colorless oil (0.64 g, 92%). ^{1}H NMR (400 MHz, $CDCl_3$) δ 6.46 (br, 2 H, Ph*H*), 6.40 (t, 1 H, Ph*H*), 5.05 (s, 2 H, PhCH_2OCO), 3.93 (t, 4 H, PhOCH_2CH$_2$CH$_2$-), 3.47 (t, 2 H, -OCOCH$_2$CH$_2$CH_2Br), 2.57 (t, 2 H, -OCOCH_2CH$_2$CH$_2$Br), 2.20 (m, 2 H, -OCOCH$_2$CH_2CH$_2$Br), 1.76 (m, 4 H, PhOCH$_2$CH_2CH$_2$-), 1.44 (m, 4 H, PhOCH$_2$CH$_2$CH_2-), 1.27 (m, 52 H, PhOCH$_2$CH$_2$CH$_2$(CH_2)$_{14}$CH$_3$ and PhOCH$_2$CH$_2$CH$_2$(CH_2)$_{12}$CH$_3$), 0.88 (t, 6 H, PhO(CH$_2$)$_{17}$CH_3 and PhO(CH$_2$)$_{15}$CH_3). ^{13}C NMR (101 MHz, $CDCl_3$) δ 172.4, 160.6, 137.9, 125.6, 106.5, 101.2, 68.2, 66.5, 32.7, 32.6, 32.1, 30.5, 29.8, 29.8, 29.8, 29.7, 29.5, 29.5, 29.4, 27.9, 26.2, 22.8, 14.3.

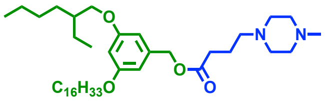

26a (IAJD216)

3-((2-Ethylhexyl)oxy)-5-(hexadecyloxy)benzyl 4-(4-methylpiperazin-1-yl)butanoate (26a, IAJD216). A mixture of compound **25a** (0.12 g, 0.19 mmol, 1.0 equiv), 1-methylpiperazine (21 mg, 0.21 mmol, 1.1 equiv), K_2CO_3 (33 mg, 0.23 mmol, 1.2 equiv) and MeCN (15 mL) was heated to 95 °C and stirred for 3 h. The reaction mixture was cooled to 23 °C and MeCN was removed under reduced pressure. Then water (20 mL) was added, and the resulted mixture was extracted with DCM (20 mL) for three times. The organic phases were collected and dried over anhydrous $MgSO_4$. After filtration, the obtained filtrate was concentrated to around 3 mL and purified by column chromatography (SiO_2) with DCM/MeOH = 30/1 and 15/1 as the eluent. Then the obtained product was dissolved in DCM (20 mL), which was washed by $NaHCO_3$ solution (2%, 20 mL). The aqueous phase was extracted by DCM (20 mL) for another two times. The organic phases were combined and dried over anhydrous $MgSO_4$. Filtration and evaporation of the solvent yielded the title compound as a colorless oil (0.12 g, 97%). ^{1}H NMR (400 MHz, $CDCl_3$) δ 6.45 (br, 2 H, Ph*H*), 6.39 (t, 1 H, Ph*H*), 5.01 (s, 2 H, PhCH_2-), 3.91 (t, 4 H, PhOCH_2-), 3.79 (m, 2 H, PhOCH_2CH(CH$_2$CH$_3$)-), 2.62–2.23 (m, 15 H, -N(CH_2CH_2)$_2$N-, -NCH_2CH$_2$CH_2COO- and -NCH_3), 1.83 (m, 2 H, -OCOCH$_2$CH_2CH$_2$-), 1.72 (m, 3 H, PhOCH$_2$CH_2(CH$_2$)$_{13}$CH$_3$ and PhOCH$_2$C*H*(CH$_2$CH$_3$)(CH$_2$)$_3$CH$_3$), 1.45–1.16 (m, 34 H, PhOCH$_2$CH$_2$(CH_2)$_{13}$CH$_3$ and PhOCH$_2$CH(CH_2CH$_3$)(CH_2)$_3$CH$_3$), 0.90 (m, 9 H, PhO(CH$_2$)$_{15}$CH_3 and PhOCH$_2$CH(CH$_2$CH_3)(CH$_2$)$_3$CH_3). ^{13}C NMR (101 MHz, $CDCl_3$) δ 173.4, 160.8, 160.6, 138.1, 106.6, 106.4, 101.0, 77.4, 70.6, 68.2, 66.3, 57.6, 55.0, 52.8, 50.7, 45.9, 39.5, 32.3, 32.0, 30.6, 29.8, 29.8, 29.8, 29.7, 29.7, 29.5, 29.5, 29.4, 29.2, 26.2,

24.0, 23.1, 22.8, 22.2, 14.2, 14.2, 11.2. Purity by HPLC: 99+%. MALDI-TOF MS m/z of $[M + H]^+$ calculated for $C_{40}H_{73}N_2O_4$: 645.6; Found: 645.6.

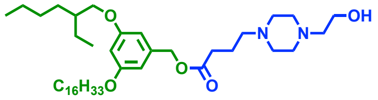

26b (IAJD217)

3-((2-Ethylhexyl)oxy)-5-(hexadecyloxy)benzyl 4-(4-(2-hydroxyethyl)piperazin-1-yl)butanoate (26b, IAJD217). Compound **26b** was synthesized from compound **25a** (0.12 g, 0.19 mmol), 1-(2-hydroxyethyl)piperazine (27 mg, 0.21 mmol) and K_2CO_3 (33 mg, 0.23 mmol) following a procedure similar to that used for the synthesis of compound **26a**. The title compound was obtained as a colorless oil (0.11 g, 85%). ^{1}H NMR (400 MHz, $CDCl_3$) δ 6.45 (br, 2 H, Ph*H*), 6.38 (t, 1 H, Ph*H*), 5.02 (s, 2 H, PhC*H*$_2$-), 3.91 (t, 4 H, PhOC*H*$_2$-), 3.80 (m, 2 H, PhOC*H*$_2$CH(CH_2CH_3)-), 3.62 (t, 2 H, -CH_2C*H*$_2$OH), 2.68–2.28 (m, 14 H, -N(C*H*$_2$C*H*$_2$)$_2$N-, -NC*H*$_2$CH$_2$C*H*$_2$COO- and -NC*H*$_2$-), 1.83 (m, 2 H, -OCOCH$_2$C*H*$_2$CH$_2$-), 1.72 (m, 3 H, PhOCH$_2$C*H*$_2$(CH$_2$)$_{13}$CH$_3$ and PhOCH$_2$C*H*(CH$_2$CH$_3$)(CH$_2$)$_3$CH$_3$), 1.53–1.15 (m, 34 H, PhOCH$_2$CH$_2$(C*H*$_2$)$_{13}$CH$_3$ and PhOCH$_2$CH(C*H*$_2$CH$_3$)(C*H*$_2$)$_3$CH$_3$), 0.89 (m, 9 H, PhO(CH$_2$)$_{15}$C*H*$_3$ and PhOCH$_2$CH(CH$_2$C*H*$_3$)(CH$_2$)$_3$C*H*$_3$). ^{13}C NMR (101 MHz, $CDCl_3$) δ 173.3, 160.8, 160.5, 138.1, 106.6, 106.4, 101.0, 70.6, 68.2, 66.3, 59.5, 57.7, 57.5, 52.9, 52.9, 39.5, 32.3, 32.0, 30.6, 29.8, 29.8, 29.7, 29.7, 29.5, 29.4, 29.4, 29.2, 26.2, 24.0, 23.1, 22.8, 22.1, 14.2, 14.2, 11.2. Purity by HPLC: 99+%. MALDI-TOF MS m/z of $[M + H]^+$ calculated for $C_{41}H_{75}N_2O_5$: 675.6; Found: 675.4.

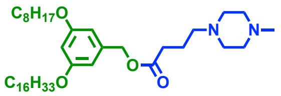

26c (IAJD218)

3-(Hexadecyloxy)-5-(octyloxy)benzyl 4-(4-methylpiperazin-1-yl)butanoate (26c, IAJD218). Compound **26c** was synthesized from compound **25b** (0.20 g, 0.37 mmol), 1-methylpiperazine (41 mg, 0.41 mmol) and K_2CO_3 (77 mg, 0.55 mmol) following a procedure similar to that used for the synthesis of compound **26a**. The title compound was obtained as a colorless oil (0.22 g, 92%). ^{1}H NMR (400 MHz, $CDCl_3$) δ 6.46 (br, 2 H, Ph*H*), 6.39 (t, 1 H, Ph*H*), 5.03 (s, 2 H, PhC*H*$_2$-), 3.92 (t, 4 H, PhOC*H*$_2$-), 2.63–2.28 (m, 15 H, -N(C*H*$_2$C*H*$_2$)$_2$N-, -NC*H*$_2$CH$_2$C*H*$_2$COO- and -NC*H*$_3$), 1.85 (m, 2 H, -OCOCH$_2$C*H*$_2$CH$_2$-), 1.75 (m, 4 H, PhOCH$_2$C*H*$_2$-), 1.44 (m, 4 H, PhOCH$_2$CH$_2$C*H*$_2$-), 1.25 (br, 32 H, PhOCH$_2$CH$_2$CH$_2$(C*H*$_2$)$_4$CH$_3$ and PhOCH$_2$CH$_2$CH$_2$(C*H*$_2$)$_{12}$CH$_3$), 0.88 (t, 6 H, -CH$_2$CH$_2$C*H*$_3$). ^{13}C NMR (101 MHz, $CDCl_3$) δ 173.3, 160.5, 138.1, 106.4, 100.9, 68.1, 66.2, 57.4, 54.9, 52.7, 45.7, 32.2, 31.9, 31.8, 29.7, 29.7, 29.6, 29.6, 29.4, 29.4, 29.3, 29.2, 26.1, 22.7, 22.1, 14.1. Purity by HPLC: 99+%. MALDI-TOF MS m/z of $[M + H]^+$ calculated for $C_{40}H_{73}N_2O_4$: 645.6; Found: 646.3.

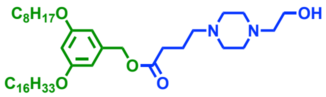

26d (IAJD219)

3-(Hexadecyloxy)-5-(octyloxy)benzyl 4-(4-(2-hydroxyethyl)piperazin-1-yl)butanoate (26d, IAJD219). Compound **26d** was synthesized from compound **25b** (0.20 g, 0.37 mmol), 1-(2-hydroxyethyl)piperazine (53 mg, 0.41 mmol) and K_2CO_3 (77 mg, 0.55 mmol) following a procedure similar to that used for the synthesis of compound **26a**. The title compound was obtained as a colorless oil (0.24 g, 96%). ^{1}H NMR (400 MHz, $CDCl_3$) δ 6.46 (br, 2 H, Ph*H*), 6.39 (t, 1 H, Ph*H*), 5.02 (s, 2 H, PhC*H*$_2$-), 3.92 (t, 4 H, PhOC*H*$_2$-), 3.61 (t, 2 H, -CH$_2$C*H*$_2$OH), 2.64–2.32 (m, 14 H, -N(C*H*$_2$C*H*$_2$)$_2$N-, -NC*H*$_2$CH$_2$C*H*$_2$COO- and -C*H*$_2$CH$_2$OH), 1.84 (m, 2 H, -OCOCH$_2$C*H*$_2$CH$_2$-), 1.75 (m, 4 H, PhOCH$_2$C*H*$_2$-), 1.44 (m, 4 H, PhOCH$_2$CH$_2$C*H*$_2$-), 1.27 (br, 32 H, PhOCH$_2$CH$_2$CH$_2$(C*H*$_2$)$_4$CH$_3$ and PhOCH$_2$CH$_2$CH$_2$(C*H*$_2$)$_{12}$CH$_3$), 0.88 (t, 6 H, -CH$_2$CH$_2$C*H*$_3$). ^{13}C NMR (101 MHz, $CDCl_3$) δ 173.3, 160.5, 138.1, 106.4, 100.9, 77.2, 68.1, 66.2, 59.3, 57.6, 57.5, 53.0, 52.8, 32.2, 31.9, 31.8, 29.7, 29.7, 29.6, 29.6, 29.4, 29.4, 29.3, 29.2, 26.1, 22.7, 22.1, 14.1. Purity by HPLC: 99+%. MALDI-TOF MS m/z of $[M + H]^+$ calculated for $C_{41}H_{75}N_2O_5$: 675.6; Found: 676.7.

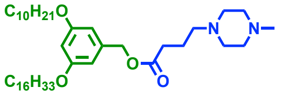

26e (IAJD220)

3-(Decyloxy)-5-(hexadecyloxy)benzyl 4-(4-methylpiperazin-1-yl)butanoate (26e, IAJD220). Compound **26e** was synthesized from compound **25c** (0.20 g, 0.31 mmol), 1-methylpiperazine (34 mg, 0.34 mmol) and K_2CO_3 (51 mg, 0.37 mmol) following a procedure similar to that used for the synthesis of compound **26a**. The title compound was obtained as a colorless oil (0.21 g, 100%). ^{1}H NMR (400 MHz, $CDCl_3$) δ 6.45 (br, 2 H, Ph*H*), 6.38 (t, 1 H, Ph*H*), 5.01 (s, 2 H, PhC*H*$_2$-), 3.91 (t, 4 H, PhOC*H*$_2$-), 2.54–2.22 (m, 15 H, -N(C*H*$_2$C*H*$_2$)$_2$N-, -NC*H*$_2$CH$_2$C*H*$_2$COO- and -NC*H*$_3$), 1.83 (m, 2 H, -OCOCH$_2$C*H*$_2$CH$_2$-), 1.74 (m, 4 H, PhOCH$_2$C*H*$_2$-), 1.44 (m, 4 H, PhOCH$_2$CH$_2$C*H*$_2$-), 1.25 (br, 36 H, PhOCH$_2$CH$_2$CH$_2$(C*H*$_2$)$_6$CH$_3$ and PhOCH$_2$CH$_2$CH$_2$(C*H*$_2$)$_{12}$CH$_3$), 0.88 (t, 6 H, -CH$_2$CH$_2$C*H*$_3$). ^{13}C NMR (101 MHz, $CDCl_3$) δ 173.4, 160.6, 138.2, 106.5, 101.0, 68.2, 66.3, 57.5, 55.0, 52.8, 50.7, 45.8, 32.3, 32.0, 32.0, 29.8, 29.8, 29.7, 29.7, 29.7, 29.5, 29.5, 29.4, 29.4, 26.2, 22.8, 22.1, 14.2. Purity by HPLC: 99+%. MALDI-TOF MS m/z of $[M + H]^+$ calculated for $C_{42}H_{77}N_2O_4$: 673.6; Found: 674.1.

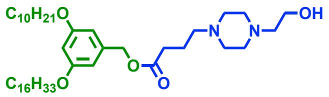

26f (IAJD221)

3-(Decyloxy)-5-(hexadecyloxy)benzyl 4-(4-(2-hydroxyethyl)piperazin-1-yl)butanoate (26f, IAJD221). Compound **26f** was synthesized from compound **25c** (0.15 g, 0.23 mmol), 1-(2-hydroxyethyl)piperazine (32 mg, 0.25 mmol) and K_2CO_3 (37 mg, 0.27 mmol) following a procedure similar to that used for the synthesis of compound **26a**. The title compound was obtained as a colorless oil (0.13 g, 81%). ^{1}H NMR (400 MHz, $CDCl_3$) δ 6.46 (br, 2 H, Ph*H*), 6.39 (t, 1 H, Ph*H*), 5.02 (s, 2 H, PhC*H*$_2$-), 3.91 (t, 4 H, PhOC*H*$_2$-), 3.62 (t, 2 H, -CH$_2$C*H*$_2$OH), 2.64–2.31 (m, 14 H, -N(C*H*$_2$C*H*$_2$)$_2$N-, -NC*H*$_2$CH$_2$C*H*$_2$COO- and -C*H*$_2$CH$_2$OH), 1.84 (m, 2 H, -OCOCH$_2$C*H*$_2$CH$_2$-), 1.75 (m, 4 H, PhOCH$_2$C*H*$_2$-), 1.42 (m, 4 H, PhOCH$_2$CH$_2$C*H*$_2$-), 1.26 (br, 36 H, PhOCH$_2$CH$_2$CH$_2$(C*H*$_2$)$_6$CH$_3$ and PhOCH$_2$CH$_2$CH$_2$(C*H*$_2$)$_{12}$CH$_3$), 0.88 (t, 6 H, -CH$_2$CH$_2$C*H*$_3$). ^{13}C NMR (101 MHz, $CDCl_3$) δ 173.4, 160.6, 138.2, 106.5, 101.0, 77.4, 68.2, 66.3, 59.5, 57.7, 57.6, 53.0, 52.9, 50.8, 32.3, 32.0, 32.0, 29.8, 29.8, 29.7, 29.7, 29.7, 29.7, 29.5, 29.5, 29.4, 29.4, 26.2, 22.8, 22.2, 14.2. Purity by HPLC: 99+%. MALDI-TOF MS m/z of $[M + H]^+$ calculated for $C_{43}H_{79}N_2O_5$: 703.6; Found: 703.8.

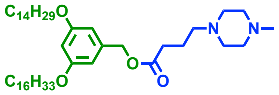

26g (IAJD222)

3-(Hexadecyloxy)-5-(tetradecyloxy)benzyl 4-(4-methylpiperazin-1-yl)butanoate (26g, IAJD222). Compound **26g** was synthesized from compound **25d** (0.18 g, 0.25 mmol), 1-methylpiperazine (28 mg, 0.28 mmol) and K_2CO_3 (38 mg, 0.28 mmol) following a procedure similar to that used for the synthesis of compound **26a**. The title compound was obtained as a light-yellow oil (0.18 g, 98%). ^{1}H NMR (400 MHz, $CDCl_3$) δ 6.46 (br, 2 H, Ph*H*), 6.39 (t, 1 H, Ph*H*), 5.02 (s, 2 H, PhC*H*$_2$-), 3.91 (t, 4 H, PhOC*H*$_2$-), 2.77–2.14 (m, 15 H, -N(C*H*$_2$C*H*$_2$)$_2$N-, -NC*H*$_2$CH$_2$C*H*$_2$COO- and -NC*H*$_3$), 1.83 (m, 2 H, -OCOCH$_2$C*H*$_2$CH$_2$-), 1.76 (m, 4 H, PhOCH$_2$C*H*$_2$-), 1.44 (m, 4 H, PhOCH$_2$CH$_2$C*H*$_2$-), 1.25 (br, 44 H, PhOCH$_2$CH$_2$CH$_2$(C*H*$_2$)$_{10}$CH$_3$ and PhOCH$_2$CH$_2$CH$_2$(C*H*$_2$)$_{12}$CH$_3$), 0.88 (t, 6 H, -CH$_2$CH$_2$C*H*$_3$). ^{13}C NMR (101 MHz, $CDCl_3$) δ 173.5, 160.6, 138.2, 106.5, 101.1, 68.2, 66.3, 57.7, 55.3, 53.2, 46.2, 32.4, 32.1, 29.8, 29.8, 29.8, 29.8, 29.7, 29.6, 29.5, 29.4, 26.2, 22.8, 22.3, 14.3. Purity by HPLC: 99+%. MALDI-TOF MS m/z of $[M + H]^+$ calculated for $C_{46}H_{85}N_2O_4$: 729.6; Found: 730.7.

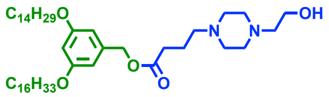

26h (IAJD223)

3-(Hexadecyloxy)-5-(tetradecyloxy)benzyl 4-(4-(2-hydroxyethyl)piperazin-1-yl)butanoate (26h, IAJD223). Compound **26h** was synthesized from compound **25d** (0.18 g, 0.25 mmol), 1-(2-hydroxyethyl)piperazine (35 mg, 0.28 mmol) and K_2CO_3 (38 mg, 0.28 mmol) following a procedure similar to that used for the synthesis of compound **26a**. The title compound was obtained as a light-yellow oil (0.14 g, 76%). ^1H NMR (400 MHz, $CDCl_3$) δ 6.46 (br, 2 H, Ph*H*), 6.39 (t, 1 H, Ph*H*), 5.03 (s, 2 H, $PhCH_2$-), 3.92 (t, 4 H, $PhOCH_2$-), 3.60 (t, 2 H, $-CH_2CH_2OH$), 2.74–2.12 (m, 14 H, $-N(CH_2CH_2)_2N$-, $-NCH_2CH_2CH_2COO$- and $-CH_2CH_2OH$), 1.83 (m, 2 H, $-OCOCH_2CH_2CH_2$-), 1.76 (m, 4 H, $PhOCH_2CH_2$-), 1.43 (m, 4 H, $PhOCH_2CH_2CH_2$-), 1.26 (br, 44 H, $PhOCH_2CH_2CH_2(CH_2)_{10}CH_3$ and $PhOCH_2CH_2CH_2(CH_2)_{12}CH_3$), 0.88 (t, 6 H, $-CH_2CH_2CH_3$). ^{13}C NMR (101 MHz, $CDCl_3$) δ 173.6, 160.6, 138.2, 106.5, 101.0, 68.2, 66.3, 59.3, 57.8, 57.7, 53.3, 53.0, 32.4, 32.1, 29.8, 29.8, 29.7, 29.6, 29.5, 29.5, 29.4, 26.2, 22.8, 22.3, 14.3. Purity by HPLC: 99+%. MALDI-TOF MS m/z of $[M + H]^+$ calculated for $C_{47}H_{87}N_2O_5$: 759.7; Found: 760.8.

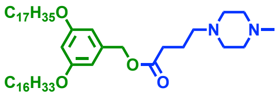

26i (IAJD224)

3-(Heptadecyloxy)-5-(hexadecyloxy)benzyl 4-(4-methylpiperazin-1-yl)butanoate (26i, IAJD224). Compound **26i** was synthesized from compound **25e** (0.30 g, 0.40 mmol), 1-methylpiperazine (44 mg, 0.44 mmol) and K_2CO_3 (66 mg, 0.48 mmol) following a procedure similar to that used for the synthesis of compound **26a**. The title compound was obtained as a colorless oil (0.30 g, 97%). ^1H NMR (400 MHz, $CDCl_3$) δ 6.45 (br, 2 H, Ph*H*), 6.38 (t, 1 H, Ph*H*), 5.02 (s, 2 H, $PhCH_2$-), 3.91 (t, 4 H, $PhOCH_2$-), 2.60–2.22 (m, 15 H, $-N(CH_2CH_2)_2N$-, $-NCH_2CH_2CH_2COO$- and $-NCH_3$), 1.84 (m, 2 H, $-OCOCH_2CH_2CH_2$-), 1.74 (m, 4 H, $PhOCH_2CH_2$-), 1.42 (m, 4 H, $PhOCH_2CH_2CH_2$-), 1.25 (br, 50 H, $PhOCH_2CH_2CH_2(CH_2)_{13}CH_3$ and $PhOCH_2CH_2CH_2(CH_2)_{12}CH_3$), 0.87 (t, 6 H, $-CH_2CH_2CH_3$). ^{13}C NMR (101 MHz, $CDCl_3$) δ 173.4, 160.6, 138.2, 106.5, 101.0, 68.2, 66.3, 57.6, 55.1, 52.9, 45.9, 32.3, 32.0, 29.8, 29.8, 29.7, 29.7, 29.5, 29.5, 29.4, 26.2, 22.8, 22.2, 14.2. Purity by HPLC: 99+%. MALDI-TOF MS m/z of $[M + H]^+$ calculated for $C_{49}H_{91}N_2O_4$: 771.7; Found: 772.7.

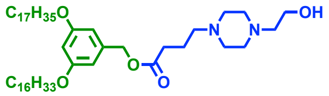

26j (IAJD225)

3-(Heptadecyloxy)-5-(hexadecyloxy)benzyl 4-(4-(2-hydroxyethyl)piperazin-1-yl)butanoate (26j, IAJD225). Compound **26j** was synthesized from compound **25e** (0.15 g, 0.20 mmol), 1-(2-hydroxyethyl)piperazine (29 mg, 0.22 mmol) and K_2CO_3 (33 mg, 0.24 mmol) following a procedure similar to that used for the synthesis of compound **26a**. The title compound was obtained as a colorless oil (0.15 g, 94%). ^{1}H NMR (400 MHz, $CDCl_3$) δ 6.45 (br, 2 H, Ph*H*), 6.38 (t, 1 H, Ph*H*), 5.01 (s, 2 H, PhC*H*$_2$-), 3.90 (t, 4 H, PhOC*H*$_2$-), 3.60 (t, 2 H, -CH_2C*H*$_2$OH), 2.64–2.25 (m, 14 H, -N(C*H*$_2$C*H*$_2$)$_2$N-, -NC*H*$_2$$CH_2$C*H*$_2$COO- and -C*H*$_2$$CH_2$OH), 1.83 (m, 2 H, -OCO$CH_2$C*H*$_2$$CH_2$-), 1.73 (m, 4 H, PhO$CH_2$C*H*$_2$-), 1.43 (m, 4 H, PhO$CH_2$$CH_2$C*H*$_2$-), 1.27 (br, 50 H, PhO$CH_2$$CH_2$$CH_2$(C*H*$_2$)$_{13}$$CH_3$ and PhO$CH_2$$CH_2$$CH_2$(C*H*$_2$)$_{12}$$CH_3$), 0.87 (t, 6 H, -$CH_2$$CH_2$C*H*$_3$). ^{13}C NMR (101 MHz, $CDCl_3$) δ 173.3, 160.5, 138.1, 106.5, 101.0, 68.1, 66.2, 59.4, 57.8, 57.6, 53.1, 53.0, 32.3, 32.0, 29.8, 29.8, 29.7, 29.7, 29.5, 29.5, 29.3, 26.1, 22.8, 22.2, 14.2. Purity by HPLC: 99+%. MALDI-TOF MS m/z of $[M + H]^+$ calculated for $C_{50}H_{93}N_2O_5$: 801.7; Found: 802.1.

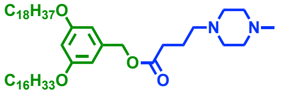

26k (IAJD226)

3-(Hexadecyloxy)-5-(octadecyloxy)benzyl 4-(4-methylpiperazin-1-yl)butanoate (26k, IAJD226). Compound **26k** was synthesized from compound **25f** (0.25 g, 0.33 mmol), 1-methylpiperazine (36 mg, 0.36 mmol) and K_2CO_3 (55 mg, 0.40 mmol) following a procedure similar to that used for the synthesis of compound **26a**. The title compound was obtained as a colorless oil (0.21 g, 81%). ^{1}H NMR (400 MHz, $CDCl_3$) δ 6.44 (br, 2 H, Ph*H*), 6.37 (t, 1 H, Ph*H*), 5.01 (s, 2 H, PhC*H*$_2$-), 3.90 (t, 4 H, PhOC*H*$_2$-), 2.56–2.24 (m, 15 H, -N(C*H*$_2$C*H*$_2$)$_2$N-, -NC*H*$_2$$CH_2$C*H*$_2$COO- and -NC*H*$_3$), 1.83 (m, 2 H, -OCO$CH_2$C*H*$_2$$CH_2$-), 1.74 (m, 4 H, PhO$CH_2$C*H*$_2$-), 1.42 (m, 4 H, PhO$CH_2$$CH_2$C*H*$_2$-), 1.24 (br, 52 H, PhO$CH_2$$CH_2$$CH_2$(C*H*$_2$)$_{14}$$CH_3$ and PhO$CH_2$$CH_2$$CH_2$(C*H*$_2$)$_{12}$$CH_3$), 0.86 (t, 6 H, -$CH_2$$CH_2$C*H*$_3$). ^{13}C NMR (101 MHz, $CDCl_3$) δ 173.3, 160.5, 138.2, 106.5, 101.0, 68.2, 66.2, 57.6, 55.1, 52.9, 45.9, 32.3, 32.0, 29.8, 29.8, 29.7, 29.7, 29.5, 29.5, 29.4, 26.2, 22.8, 22.2, 14.2. Purity by HPLC: 99+%. MALDI-TOF MS m/z of $[M + H]^+$ calculated for $C_{50}H_{93}N_2O_4$: 785.7; Found: 785.9.

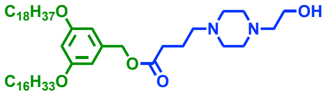

26l (IAJD227)

3-(Hexadecyloxy)-5-(octadecyloxy)benzyl 4-(4-(2-hydroxyethyl)piperazin-1-yl)butanoate (26l, IAJD227). Compound **26l** was synthesized from compound **25f** (0.30 g, 0.39 mmol), 1-(2-hydroxyethyl)piperazine (56 mg, 0.43 mmol) and K_2CO_3 (65 mg, 0.47 mmol) following a procedure

similar to that used for the synthesis of compound **26a**. The title compound was obtained as a colorless oil (0.32 g, 100%). ^{1}H NMR (400 MHz, $CDCl_3$) δ 6.46 (br, 2 H, Ph*H*), 6.39 (t, 1 H, Ph*H*), 5.02 (s, 2 H, PhC*H*$_2$-), 3.91 (t, 4 H, PhOC*H*$_2$-), 3.61 (t, 2 H, -CH_2C*H*$_2$OH), 2.64–2.28 (m, 14 H, -N(C*H*$_2$C*H*$_2$)$_2$N-, -NC*H*$_2$$CH_2$C*H*$_2$COO- and -C*H*$_2$$CH_2$OH), 1.84 (m, 2 H, -OCOC$H_2$C*H*$_2CH_2$-), 1.75 (m, 4 H, PhOC$H_2$C*H*$_2$-), 1.42 (m, 4 H, PhOC$H_2CH_2$C*H*$_2$-), 1.25 (br, 52 H, PhOC$H_2CH_2CH_2$(C*H*$_2$)$_{12}CH_3$ and PhOCH_2CH_2CH_2(C*H*$_2$)$_{14}$CH_3), 0.87 (t, 6 H, -CH_2CH_2C*H*$_3$). ^{13}C NMR (101 MHz, $CDCl_3$) δ 173.4, 160.6, 138.2, 106.5, 101.0, 68.2, 66.3, 59.4, 57.8, 57.6, 53.5, 53.1, 53.0, 32.3, 32.1, 29.8, 29.8, 29.7, 29.7, 29.5, 29.5, 29.4, 26.2, 22.8, 22.2, 14.2. Purity by HPLC: 99+%. MALDI-TOF MS m/z of $[M + H]^+$ calculated for $C_{51}H_{95}N_2O_5$: 815.7; Found: 815.6.

3.5 Synthesis of Heptadecane-Based IAJDs with 3,5-Nonsymmetric Alkyl Chains

Scheme S5. Synthesis of Heptadecane-Based IAJDs with 3,5-Nonsymmetric Alkyl Chains

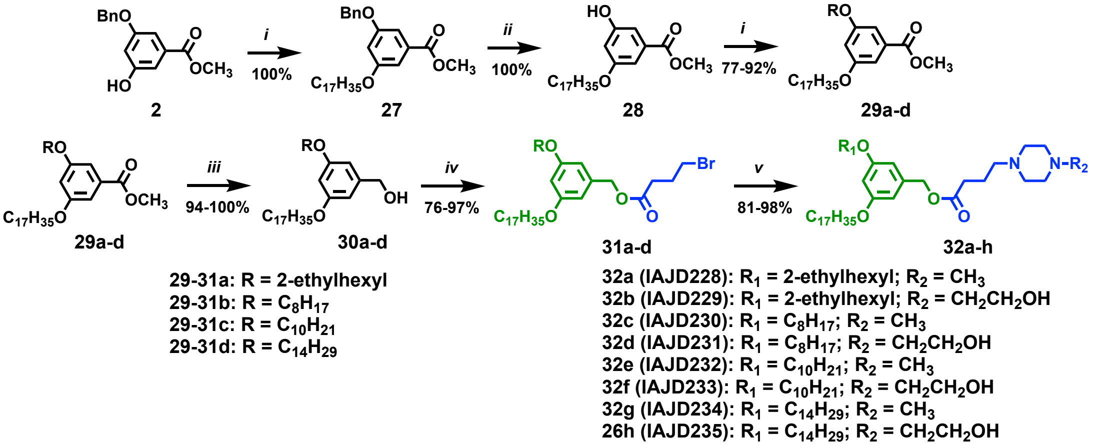

Reagents and conditions: (*i*) RBr/ROTs, K_2CO_3, DMF, 120 °C, 2 h; (*ii*) H_2, Pd/C, DCM, MeOH, 12 h; (*iii*) $LiAlH_4$, THF, 0–23 °C, 1 h; (*iv*) DCC, DPTS, DCM, 12 h; (*v*) K_2CO_3, MeCN, 95 °C, 3 h.

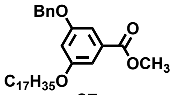

27

Methyl 3-(benzyloxy)-5-(heptadecyloxy)benzoate (27). A mixture of compound **2** (1.20 g, 4.64 mmol, 1 equiv), $C_{17}H_{35}OTs$ (2.11 g, 5.11 mmol, 1.1 equiv), K_2CO_3 (1.28 g, 9.28 mmol, 2 equiv) and DMF (15 mL) was heated to 120 °C and stirred under N_2 atmosphere for 2 h. The reaction mixture was cooled to 23 °C and poured into ice/water (150 mL). The resulted white precipitates in the solution were filtered and collected. Then the precipitates were recrystallized from minimum acetone to afford the title compound as a white solid (2.25 g, 97%). ^{1}H NMR (400 MHz, $CDCl_3$) δ 7.47–7.29 (m, 5 H, Ph*H*), 7.27 (br, 1 H, Ph*H*), 7.20 (br, 1 H, Ph*H*), 6.73 (t, 1 H, Ph*H*), 5.08 (s, 2 H, PhC*H*$_2$O-), 3.96 (t, 2 H, PhOC*H*$_2$-),

3.90 (s, 3 H, $PhCOOCH_3$), 1.77 (m, 2 H, $PhOCH_2CH_2CH_2(CH_2)_{13}CH_3$), 1.45 (m, 2 H, $PhOCH_2CH_2CH_2(CH_2)_{13}CH_3$), 1.27 (m, 26 H, $PhOCH_2CH_2CH_2(CH_2)_{13}CH_3$), 0.89 (t, 3 H, $PhO(CH_2)_{16}CH_3$). ^{13}C NMR (101 MHz, $CDCl_3$) δ 167.0, 160.3, 159.9, 136.7, 132.1, 128.7, 128.2, 127.7, 108.3, 108.0, 107.1, 77.4, 70.4, 68.5, 63.0, 52.3, 32.1, 29.8, 29.8, 29.7, 29.7, 29.5, 29.3, 26.4, 26.1, 25.9, 22.8, 14.2. Mp = 63 °C.

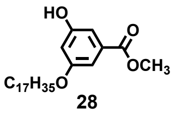

28

Methyl 3-hydroxy-5-(heptadecyloxy)benzoate (28). Compound **27** (2.25 g, 4.49 mmol) was dissolved in a mixture of DCM (20 mL) and methanol (10 mL). Then Pd/C (0.11 g, 5 wt%) was added to the solution and the flask was evacuated and filled with hydrogen for three times. Afterwards, the mixture was stirred at 23 °C under hydrogen atmosphere for 12 h. The reaction mixture was filtered through Celite, and the filter cake was washed with DCM. Evaporation of the solvent yielded the title compound as a white solid (1.92 g, 99%). ^{1}H NMR (400 MHz, $CDCl_3$) δ 7.14 (d, 2 H, Ph*H*), 6.62 (t, 1 H, Ph*H*), 3.95 (t, 2 H, $PhOCH_2$-), 3.90 (s, 3 H, $PhCOOCH_3$), 1.73 (m, 2 H, $PhOCH_2CH_2CH_2(CH_2)_{13}CH_3$), 1.41 (m, 2 H, $PhOCH_2CH_2CH_2(CH_2)_{13}CH_3$), 1.26 (m, 26 H, $PhOCH_2CH_2CH_2(CH_2)_{13}CH_3$), 0.88 (t, 3 H, $PhO(CH_2)_{16}CH_3$). ^{13}C NMR (101 MHz, $CDCl_3$) δ 167.0, 160.4, 157.0, 132.0, 108.2, 107.8, 107.1, 68.4, 52.2, 32.8, 31.9, 29.7, 29.7, 29.6, 29.6, 29.5, 29.4, 29.2, 26.2, 26.0, 25.8, 22.7, 14.1. Mp = 98 °C.

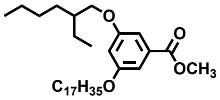

29a

Methyl 3-((2-ethylhexyl)oxy)-5-(heptadecyloxy)benzoate (29a). A mixture of compound **28** (0.60 g, 1.47 mmol, 1 equiv), 2-ethylhexyl bromide (0.31 g, 1.62 mmol, 1.1 equiv), K_2CO_3 (0.41 g, 2.94 mmol, 2 equiv) and DMF (15 mL) was heated to 80 °C and stirred under N_2 atmosphere for 12 h. The reaction mixture was cooled to 23 °C and DMF was removed under reduced pressure. Then water (20 mL) was added, and the resulted mixture was extracted with DCM (20 mL) for three times. The organic phases were collected and dried over anhydrous $MgSO_4$. After filtration, the obtained filtrate was concentrated to around 3 mL and purified by column chromatography (SiO_2) with hexane/EtOAc = 20/1 as the eluent to give the title compound as a light-yellow oil (0.47 g, 61%). ^{1}H NMR (400 MHz, $CDCl_3$) δ 7.16 (br, 2 H, Ph*H*), 6.64 (t, 1 H, Ph*H*), 3.97 (t, 2 H, $PhOCH_2(CH_2)_{15}CH_3$), 3.90 (s, 3 H, $PhCOOCH_3$), 3.85 (m, 2 H, $PhOCH_2CH(CH_2CH_3)$-), 1.83–1.69 (m, 3 H, $PhOCH_2CH_2(CH_2)_{14}CH_3$ and $PhOCH_2CH(CH_2CH_3)(CH_2)_3CH_3$), 1.55–1.18 (m, 36 H, $PhOCH_2CH_2(CH_2)_{14}CH_3$ and

$PhOCH_2CH(CH_2CH_3)(CH_2)_3CH_3)$, 1.01–0.85 (m, 9 H, $PhO(CH_2)_{16}CH_3$ and $PhOCH_2CH(CH_2CH_3)(CH_2)_3CH_3)$. ^{13}C NMR (101 MHz, $CDCl_3$) δ 167.2, 160.6, 160.3, 131.9, 107.9, 107.6, 106.7, 70.9, 68.5, 52.3, 39.5, 32.1, 30.7, 29.8, 29.8, 29.8, 29.7, 29.5, 29.5, 29.4, 29.2, 26.2, 24.0, 23.2, 22.8, 14.2, 14.2, 11.2.

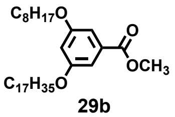

29b

Methyl 3-(heptadecyloxy)-5-(octyloxy)benzoate (29b). A mixture of compound **28** (0.40 g, 0.98 mmol, 1 equiv), 1-bromooctane (0.21 g, 1.08 mmol, 1.1 equiv), K_2CO_3 (0.27 g, 1.97 mmol, 2 equiv) and DMF (15 mL) was heated to 120 °C and stirred under N_2 atmosphere for 2 h. The reaction mixture was cooled to 23 °C and poured into ice/water (150 mL). The resulted white precipitates in the solution were filtered and collected. Then the precipitates were recrystallized from minimum acetone to afford the title compound as a white solid (0.33 g, 66%). ^1H NMR (400 MHz, $CDCl_3$) δ 7.15 (br, 2 H, Ph*H*), 6.63 (t, 1 H, Ph*H*), 3.96 (t, 4 H, $PhOCH_2CH_2CH_2$-), 3.89 (s, 3 H, $PhCOOCH_3$), 1.77 (m, 4 H, $PhOCH_2CH_2CH_2$-), 1.44 (m, 4 H, $PhOCH_2CH_2CH_2$-), 1.27 (m, 34 H, $PhOCH_2CH_2CH_2(CH_2)_4CH_3$ and $PhOCH_2CH_2CH_2(CH_2)_{13}CH_3)$, 0.88 (t, 6 H, $PhO(CH_2)_7CH_3$ and $PhO(CH_2)_{16}CH_3)$. ^{13}C NMR (101 MHz, $CDCl_3$) δ 167.1, 160.3, 131.9, 107.8, 106.7, 77.4, 68.5, 52.3, 32.9, 32.1, 31.9, 29.8, 29.8, 29.7, 29.7, 29.6, 29.5, 29.5, 29.4, 29.3, 26.1, 25.9, 22.8, 22.8, 14.2, 14.2. Mp = 56 °C.

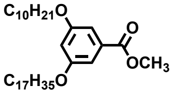

29c

Methyl 3-(decyloxy)-5-(heptadecyloxy)benzoate (29c). Compound **29c** was synthesized from compound **28** (0.40 g, 0.98 mmol, 1 equiv), 1-bromodecane (0.24 g, 1.08 mmol, 1.1 equiv) and K_2CO_3 (0.27 g, 1.97 mmol, 2 equiv) following a procedure similar to that used for the synthesis of compound **29b**. The title compound was obtained as a white solid (0.34 g, 63%). ^1H NMR (400 MHz, $CDCl_3$) δ 7.16 (br, 2 H, Ph*H*), 6.62 (t, 1 H, Ph*H*), 3.96 (t, 4 H, $PhOCH_2CH_2CH_2$-), 3.89 (s, 3 H, $PhCOOCH_3$), 1.77 (m, 4 H, $PhOCH_2CH_2CH_2$-), 1.43 (m, 4 H, $PhOCH_2CH_2CH_2$-), 1.26 (m, 38 H, $PhOCH_2CH_2CH_2(CH_2)_6CH_3$ and $PhOCH_2CH_2CH_2(CH_2)_{13}CH_3)$, 0.88 (t, 6 H, $PhO(CH_2)_9CH_3$ and $PhO(CH_2)_{16}CH_3)$. ^{13}C NMR (101 MHz, $CDCl_3$) δ 167.1, 160.3, 131.9, 107.8, 106.7, 77.4, 68.5, 63.0, 52.3, 32.1, 32.0, 29.8, 29.8, 29.7, 29.7, 29.7, 29.6, 29.5, 29.5, 29.3, 26.1, 25.9, 22.8, 14.2. Mp = 58 °C.

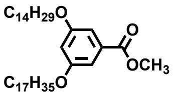

29d

Methyl 3-(heptadecyloxy)-5-(tetradecyloxy)benzoate (29d). Compound **29d** was synthesized from compound **28** (0.40 g, 0.98 mmol, 1 equiv), 1-bromotetradecane (0.30 g, 1.08 mmol, 1.1 equiv) and K_2CO_3 (0.27 g, 1.97 mmol, 2 equiv) following a procedure similar to that used for the synthesis of compound **29b**. The title compound was obtained as a white solid (0.48 g, 81%). ^{1}H NMR (400 MHz, $CDCl_3$) δ 7.15 (br, 2 H, Ph*H*), 6.62 (t, 1 H, Ph*H*), 3.96 (t, 4 H, $PhOCH_2CH_2CH_2$-), 3.88 (s, 3 H, $PhCOOCH_3$), 1.76 (m, 4 H, $PhOCH_2CH_2CH_2$-), 1.43 (m, 4 H, $PhOCH_2CH_2CH_2$-), 1.25 (m, 46 H, $PhOCH_2CH_2CH_2(CH_2)_{10}CH_3$ and $PhOCH_2CH_2CH_2(CH_2)_{13}CH_3$), 0.86 (t, 6 H, $PhO(CH_2)_{13}CH_3$ and $PhO(CH_2)_{16}CH_3$). ^{13}C NMR (101 MHz, $CDCl_3$) δ 167.1, 160.3, 131.9, 107.8, 106.7, 77.4, 68.4, 63.0, 52.3, 32.1, 29.8, 29.8, 29.7, 29.7, 29.6, 29.5, 29.3, 26.4, 26.1, 25.9, 22.8, 14.2. Mp = 65 °C.

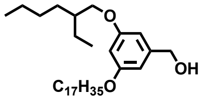

30a

(3-((2-Ethylhexyl)oxy)-5-(heptadecyloxy)phenyl)methanol (30a). Compound **29a** (0.48 g, 0.93 mmol, 1 equiv) was dissolved in 6 mL dry THF, which was added dropwise to a slurry of $LiAlH_4$ (35 mg, 0.93 mmol, 1 equiv) in dry THF (6 mL) at 0 °C under N_2 atmosphere. Afterwards, the resulted mixture was stirred at 23 °C for 1 h. Finally, the reaction was quenched by the successive addition of water (0.3 mL), 15% NaOH aqueous solution (0.3 mL) and water (1.5 mL). White precipitates were formed in the mixture and filtered out. The filtrate was dried over anhydrous $MgSO_4$ and filtered. The solvent was removed under reduced pressure to afford the title compound as a colorless oil (0.40 g, 83%). ^{1}H NMR (400 MHz, $CDCl_3$) δ 6.50 (d, 2 H, Ph*H*), 6.38 (t, 1 H, Ph*H*), 4.62 (s, 2 H, $PhCH_2OH$), 3.94 (t, 2 H, $PhOCH_2(CH_2)_{15}CH_3$), 3.82 (m, 2 H, $PhOCH_2CH(CH_2CH_3)$-), 1.72 (m, 3 H, $PhOCH_2CH_2(CH_2)_{14}CH_3$ and $PhOCH_2CH(CH_2CH_3)(CH_2)_3CH_3$), 1.56–1.17 (m, 36 H, $PhOCH_2CH_2(CH_2)_{14}CH_3$ and $PhOCH_2CH(CH_2CH_3)(CH_2)_3CH_3$), 0.89 (m, 9 H, $PhO(CH_2)_{16}CH_3$ and $PhOCH_2CH(CH_2CH_3)(CH_2)_3CH_3$). ^{13}C NMR (101 MHz, $CDCl_3$) δ 161.0, 160.7, 143.3, 105.3, 105.1, 100.7, 70.7, 68.2, 65.6, 39.5, 32.1, 30.7, 30.5, 29.8, 29.8, 29.8, 29.7, 29.5, 29.5, 29.4, 29.2, 26.2, 24.0, 23.2, 22.8, 14.3, 14.2, 11.2.

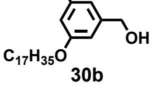

30b

(3-(Heptadecyloxy)-5-(octyloxy)phenyl)methanol (30b). Compound **30b** was synthesized from compound **29b** (0.33 g, 0.64 mmol, 1 equiv) and $LiAlH_4$ (24 mg, 0.64 mmol, 1 equiv) following a

procedure similar to that used for the synthesis of compound **30a**. The title compound was obtained as a colorless oil (0.22 g, 70%). ^{1}H NMR (400 MHz, $CDCl_3$) δ 6.50 (br, 2 H, Ph*H*), 6.37 (t, 1 H, Ph*H*), 4.60 (s, 2 H, $PhCH_2OH$), 3.93 (t, 4 H, $PhOCH_2CH_2CH_2$-), 1.76 (m, 4 H, $PhOCH_2CH_2CH_2$-), 1.43 (m, 4 H, $PhOCH_2CH_2CH_2$-), 1.29 (m, 34 H, $PhOCH_2CH_2CH_2(CH_2)_4CH_3$ and $PhOCH_2CH_2CH_2(CH_2)_{13}CH_3$), 0.88 (t, 6 H, $PhO(CH_2)_7CH_3$ and $PhO(CH_2)_{16}CH_3$). ^{13}C NMR (101 MHz, $CDCl_3$) δ 160.7, 143.4, 105.2, 100.7, 77.4, 68.2, 65.4, 63.1, 32.9, 32.1, 32.0, 29.8, 29.8, 29.8, 29.7, 29.6, 29.5, 29.5, 29.4, 29.4, 26.2, 25.9, 22.8, 22.8, 14.3, 14.2.

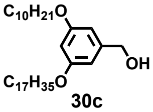

30c

(3-(Decyloxy)-5-(heptadecyloxy)phenyl)methanol (30c). Compound **30c** was synthesized from compound **29c** (0.34 g, 0.62 mmol, 1 equiv) and $LiAlH_4$ (23 mg, 0.62 mmol, 1 equiv) following a procedure similar to that used for the synthesis of compound **30a**. The title compound was obtained as a white solid (0.21 g, 65%). ^{1}H NMR (400 MHz, $CDCl_3$) δ 6.49 (br, 2 H, Ph*H*), 6.37 (t, 1 H, Ph*H*), 4.60 (s, 2 H, $PhCH_2OH$), 3.93 (t, 4 H, $PhOCH_2CH_2CH_2$-), 1.76 (m, 4 H, $PhOCH_2CH_2CH_2$-), 1.44 (m, 4 H, $PhOCH_2CH_2CH_2$-), 1.29 (m, 38 H, $PhOCH_2CH_2CH_2(CH_2)_6CH_3$ and $PhOCH_2CH_2CH_2(CH_2)_{13}CH_3$), 0.89 (t, 6 H, $PhO(CH_2)_9CH_3$ and $PhO(CH_2)_{16}CH_3$). ^{13}C NMR (101 MHz, $CDCl_3$) δ 160.7, 143.5, 105.2, 100.7, 77.4, 68.2, 65.3, 64.0, 63.0, 32.9, 32.1, 29.8, 29.8, 29.8, 29.7, 29.6, 29.6, 29.5, 29.5, 29.4, 29.3, 26.4, 26.2, 25.9, 22.9, 22.8, 14.2. Mp = 42 °C.

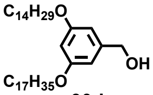

30d

(3-(Heptadecyloxy)-5-(tetradecyloxy)phenyl)methanol (30d). Compound **30d** was synthesized from compound **29d** (0.47 g, 0.78 mmol, 1 equiv) and $LiAlH_4$ (30 mg, 0.78 mmol, 1 equiv) following a procedure similar to that used for the synthesis of compound **30a**. The title compound was obtained as a white solid (0.52 g, 83%). ^{1}H NMR (400 MHz, $CDCl_3$) δ 6.49 (br, 2 H, Ph*H*), 6.36 (t, 1 H, Ph*H*), 4.60 (s, 2 H, $PhCH_2OH$), 3.92 (t, 4 H, $PhOCH_2CH_2CH_2$-), 1.76 (m, 4 H, $PhOCH_2CH_2CH_2$-), 1.44 (m, 4 H, $PhOCH_2CH_2CH_2$-), 1.26 (m, 46 H, $PhOCH_2CH_2CH_2(CH_2)_{10}CH_3$ and $PhOCH_2CH_2CH_2(CH_2)_{13}CH_3$), 0.88 (t, 6 H, $PhO(CH_2)_{13}CH_3$ and $PhO(CH_2)_{16}CH_3$). ^{13}C NMR (101 MHz, $CDCl_3$) δ 160.6, 160.6, 143.4, 105.2, 100.7, 77.5, 77.2, 76.8, 68.2, 65.5, 65.4, 32.1, 31.9, 29.8, 29.8, 29.7, 29.7, 29.5, 29.5, 29.4, 26.2, 22.8, 22.8, 14.2, 14.2. Mp = 48 °C.

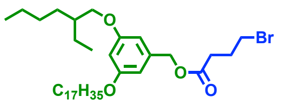

31a

3-((2-Ethylhexyl)oxy)-5-(heptadecyloxy)benzyl 4-bromobutanoate (31a). Compound **30a** (0.40 g, 0.82 mmol, 1 equiv), 4-bromobutyric acid (0.15 g, 0.90 mmol, 1.1 equiv) and DPTS (0.26 g, 0.90 mmol, 1.1 equiv) were dissolved in 8 mL DCM. DCC (0.34 g, 1.64 mmol, 2 equiv) was added into the above mixture. The reaction mixture was stirred at 23 °C for 12 h. Afterwards, the resulted precipitates (urea) were filtered out from the reaction mixture. The filtrate was concentrated to around 3 mL and purified by column chromatography (SiO_2) with hexane/DCM = 1/1 as the mobile phase to give the title compound as a colorless oil (0.41 g, 79%). ^{1}H NMR (400 MHz, $CDCl_3$) δ 6.47 (d, 2 H, PhH), 6.42 (t, 1 H, PhH), 5.05 (s, 2 H, $PhC\mathit{H}_2OCO$), 3.92 (t, 2 H, $PhOC\mathit{H}_2(CH_2)_{15}CH_3$), 3.82 (m, 2 H, $PhOC\mathit{H}_2CH(CH_2CH_3)$-), 3.47 (t, 2 H, -$OCOCH_2CH_2C\mathit{H}_2Br$), 2.57 (t, 2 H, -$OCOC\mathit{H}_2CH_2CH_2Br$), 2.20 (m, 2 H, -$OCOCH_2C\mathit{H}_2CH_2Br$), 1.82–1.65 (m, 3 H, $PhOCH_2C\mathit{H}_2(CH_2)_{14}CH_3$ and $PhOCH_2C\mathit{H}(CH_2CH_3)(CH_2)_3CH_3$), 1.56–1.25 (m, 36 H, $PhOCH_2CH_2(C\mathit{H}_2)_{14}CH_3$ and $PhOCH_2CH(C\mathit{H}_2CH_3)(C\mathit{H}_2)_3CH_3$), 0.90 (m, 9 H, $PhO(CH_2)_{16}C\mathit{H}_3$ and $PhOCH_2CH(CH_2C\mathit{H}_3)(CH_2)_3C\mathit{H}_3$). ^{13}C NMR (101 MHz, $CDCl_3$) δ 172.5, 160.9, 160.6, 137.9, 106.6, 106.4, 101.2, 77.4, 70.7, 68.2, 66.6, 39.5, 32.8, 32.6, 32.1, 30.7, 29.8, 29.8, 29.8, 29.7, 29.6, 29.5, 29.4, 29.2, 27.9, 26.2, 24.0, 23.2, 22.8, 14.3, 14.2, 11.3.

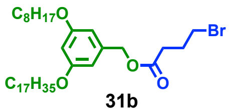

31b

3-(Heptadecyloxy)-5-(octyloxy)benzyl 4-bromobutanoate (31b). Compound **31b** was synthesized from compound **30b** (0.22 g, 0.45 mmol, 1 equiv), 4-bromobutyric acid (0.08 g, 0.49 mmol, 1.1 equiv), DPTS (0.15 g, 0.49 mmol, 1.1 equiv) and DCC (0.19 g, 0.90 mmol, 2 equiv) following a procedure similar to that used for the synthesis of compound **31a**. The title compound was obtained as a colorless oil (0.26 g, 91%). ^{1}H NMR (400 MHz, $CDCl_3$) δ 6.47 (br, 2 H, PhH), 6.41 (t, 1 H, PhH), 5.05 (s, 2 H, $PhC\mathit{H}_2OCO$), 3.93 (t, 4 H, $PhOC\mathit{H}_2CH_2CH_2$-), 3.46 (t, 2 H, -$OCOCH_2CH_2C\mathit{H}_2Br$), 2.56 (t, 2 H, -$OCOC\mathit{H}_2CH_2CH_2Br$), 2.20 (m, 2 H, -$OCOCH_2C\mathit{H}_2CH_2Br$), 1.77 (m, 4 H, $PhOCH_2C\mathit{H}_2CH_2$-), 1.44 (m, 4 H, $PhOCH_2CH_2C\mathit{H}_2$-), 1.27 (m, 34 H, $PhOCH_2CH_2CH_2(C\mathit{H}_2)_4CH_3$ and $PhOCH_2CH_2CH_2(C\mathit{H}_2)_{13}CH_3$), 0.89 (t, 6 H, $PhO(CH_2)_7C\mathit{H}_3$ and $PhO(CH_2)_{16}C\mathit{H}_3$). ^{13}C NMR (101 MHz, $CDCl_3$) δ 172.4, 160.6, 137.9, 106.5, 101.2, 77.4, 68.2, 66.5, 64.9, 32.8, 32.7, 32.7, 32.6, 32.1, 32.0, 29.8, 29.8, 29.8, 29.7, 29.7, 29.7, 29.5, 29.5, 29.4, 28.7, 28.0, 27.9, 26.2, 26.1, 22.8, 22.8, 14.3, 14.2.

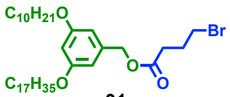

31c

3-(Decyloxy)-5-(heptadecyloxy)benzyl 4-bromobutanoate (31c). Compound **31c** was synthesized from compound **30c** (0.22 g, 0.42 mmol, 1 equiv), 4-bromobutyric acid (0.08 g, 0.47 mmol, 1.1 equiv), DPTS (0.14 g, 0.47 mmol, 1.1 equiv) and DCC (0.18 g, 0.85 mmol, 2 equiv) following a procedure similar to that used for the synthesis of compound **31a**. The title compound was obtained as a colorless oil (0.28 g, 99%). ^{1}H NMR (400 MHz, $CDCl_3$) δ 6.46 (br, 2 H, Ph*H*), 6.40 (t, 1 H, Ph*H*), 5.05 (s, 2 H, $PhCH_2OCO$), 3.93 (t, 4 H, $PhOCH_2CH_2CH_2$-), 3.47 (t, 2 H, -$OCOCH_2CH_2CH_2Br$), 2.53 (t, 2 H, -$OCOCH_2CH_2CH_2Br$), 2.20 (m, 2 H, -$OCOCH_2CH_2CH_2Br$), 1.76 (m, 4 H, $PhOCH_2CH_2CH_2$-), 1.44 (m, 4 H, $PhOCH_2CH_2CH_2$-), 1.27 (m, 38 H, $PhOCH_2CH_2CH_2(CH_2)_6CH_3$ and $PhOCH_2CH_2CH_2(CH_2)_{13}CH_3$), 0.89 (t, 6 H, $PhO(CH_2)_9CH_3$ and $PhO(CH_2)_{16}CH_3$). ^{13}C NMR (101 MHz, $CDCl_3$) δ 172.4, 160.6, 137.9, 106.5, 101.2, 68.2, 66.5, 64.9, 32.8, 32.7, 32.6, 32.1, 32.0, 29.8, 29.8, 29.8, 29.7, 29.7, 29.7, 29.5, 29.5, 29.5, 29.4, 28.8, 28.0, 27.9, 26.2, 26.1, 22.8, 14.3.

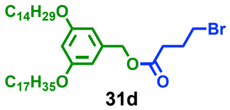

31d

3-(Heptadecyloxy)-5-(tetradecyloxy)benzyl 4-bromobutanoate (31d). Compound **31d** was synthesized from compound **30d** (0.40 g, 0.70 mmol, 1 equiv), 4-bromobutyric acid (0.13 g, 0.77 mmol, 1.1 equiv), DPTS (0.24 g, 0.77 mmol, 1.1 equiv) and DCC (0.29 g, 1.40 mmol, 2 equiv) following a procedure similar to that used for the synthesis of compound **31a**. The title compound was obtained as a colorless oil (0.41 g, 81%). ^{1}H NMR (400 MHz, $CDCl_3$) δ 6.46 (br, 2 H, Ph*H*), 6.40 (t, 1 H, Ph*H*), 5.05 (s, 2 H, $PhCH_2OCO$), 3.93 (t, 4 H, $PhOCH_2CH_2CH_2$-), 3.47 (t, 2 H, -$OCOCH_2CH_2CH_2Br$), 2.53 (t, 2 H, -$OCOCH_2CH_2CH_2Br$), 2.20 (m, 2 H, -$OCOCH_2CH_2CH_2Br$), 1.76 (m, 4 H, $PhOCH_2CH_2CH_2$-), 1.44 (m, 4 H, $PhOCH_2CH_2CH_2$-), 1.27 (m, 46 H, $PhOCH_2CH_2CH_2(CH_2)_{10}CH_3$ and $PhOCH_2CH_2CH_2(CH_2)_{13}CH_3$), 0.88 (t, 6 H, $PhO(CH_2)_{13}CH_3$ and $PhO(CH_2)_{16}CH_3$). ^{13}C NMR (101 MHz, $CDCl_3$) δ 172.7, 172.4, 160.6, 137.9, 106.5, 101.2, 77.5, 77.2, 76.9, 68.2, 66.5, 64.9, 32.8, 32.8, 32.7, 32.7, 32.6, 32.1, 29.9, 29.8, 29.8, 29.7, 29.7, 29.6, 29.5, 29.4, 28.8, 28.0, 27.9, 26.2, 26.1, 22.8, 14.3.

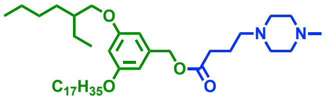

32a (IAJD228)

3-((2-Ethylhexyl)oxy)-5-(heptadecyloxy)benzyl 4-(4-methylpiperazin-1-yl)butanoate (32a, IAJD228). A mixture of compound **31a** (0.21 g, 0.32 mmol, 1.0 equiv), 1-methylpiperazine (98 mg, 0.98 mmol), K_2CO_3 (89 mg, 0.64 mmol) and MeCN (15 mL) was heated to 95 °C and stirred for 3 h. The reaction mixture was cooled to 23 °C and MeCN was removed under reduced pressure. Then water (20 mL) was added, and the resulted mixture was extracted with DCM (20 mL) for three times. The organic phases were collected and dried over anhydrous $MgSO_4$. After filtration, the obtained filtrate was concentrated to around 3 mL and purified by column chromatography (SiO_2) with DCM/MeOH = 30/1 and 15/1 as the eluent. Then the obtained product was dissolved in DCM (20 mL), which was washed by $NaHCO_3$ solution (2%, 20 mL). The aqueous phase was extracted by DCM (20 mL) for another two times. The organic phases were combined and dried over anhydrous $MgSO_4$. Filtration and evaporation of the solvent yielded the title compound as a colorless oil (0.18 g, 85%). ^1H NMR (400 MHz, $CDCl_3$) δ 6.45 (br, 2 H, Ph*H*), 6.39 (t, 1 H, Ph*H*), 5.02 (s, 2 H, PhCH_2-), 3.91 (t, 4 H, PhOCH_2-), 3.80 (m, 2 H, PhOCH_2CH(CH_2CH_3)-), 2.59–2.23 (m, 15 H, -N(CH_2CH_2)$_2$N-, -NCH_2CH$_2$CH_2COO- and -NCH_3), 1.83 (m, 2 H, -OCOCH$_2$CH_2CH$_2$-), 1.73 (m, 3 H, PhOCH$_2$CH_2(CH_2)$_{14}$CH$_3$ and PhOCH$_2$C*H*(CH_2CH_3)(CH_2)$_3$CH$_3$), 1.34–1.16 (m, 36 H, PhOCH$_2$CH$_2$(CH_2)$_{14}$CH$_3$ and PhOCH$_2$CH(CH_2CH$_3$)(CH_2)$_3$CH$_3$), 0.89 (m, 9 H, PhO(CH_2)$_{16}$CH_3 and PhOCH$_2$CH(CH$_2$CH_3)(CH_2)$_3$CH_3). ^{13}C NMR (101 MHz, $CDCl_3$) δ 173.3, 160.7, 160.5, 138.0, 106.5, 106.3, 100.9, 70.5, 68.1, 66.2, 57.5, 55.0, 52.9, 45.9, 39.4, 32.2, 31.9, 30.5, 29.7, 29.7, 29.6, 29.6, 29.4, 29.4, 29.3, 29.1, 26.1, 23.9, 23.0, 22.7, 22.1, 14.1, 14.1, 11.1. Purity by HPLC: 99+%. MALDI-TOF MS m/z of $[M + H]^+$ calculated for $C_{41}H_{75}N_2O_4$: 659.6; Found: 660.1.

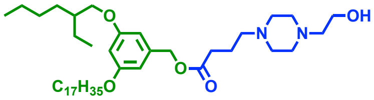

32b (IAJD229)

3-((2-Ethylhexyl)oxy)-5-(heptadecyloxy)benzyl 4-(4-(2-hydroxyethyl)piperazin-1-yl)butanoate (32b, IAJD229). Compound **32b** was synthesized from compound **31a** (0.21 g, 0.32 mmol), 11-(2-hydroxyethyl)piperazine (98 mg, 0.98 mmol), K_2CO_3 (89 mg, 0.64 mmol) following a procedure similar to that used for the synthesis of compound **32a**. The title compound was obtained as a colorless oil (0.18 g, 80%). ^1H NMR (400 MHz, $CDCl_3$) δ 6.45 (br, 2 H, Ph*H*), 6.38 (t, 1 H, Ph*H*), 5.02 (s, 2 H, PhCH_2-), 3.91 (t, 4 H, PhOCH_2-), 3.80 (m, 2 H, PhOCH_2CH(CH_2CH_3)-), 3.62 (t, 2 H, -CH$_2$CH_2OH), 2.71–2.27 (m,

14 H, -N(CH_2CH_2)$_2$N-, -NCH_2CH$_2$CH_2COO- and -NCH_2-), 1.83 (m, 2 H, -OCOCH$_2$CH_2CH$_2$-), 1.72 (m, 3 H, PhOCH$_2$CH_2(CH$_2$)$_{14}$CH$_3$ and PhOCH$_2$CH(CH$_2$CH$_3$)(CH$_2$)$_3$CH$_3$), 1.53–1.15 (m, 36 H, PhOCH$_2$CH$_2$(CH_2)$_{14}$CH$_3$ and PhOCH$_2$CH(CH_2CH$_3$)(CH_2)$_3$CH$_3$), 0.89 (m, 9 H, PhO(CH$_2$)$_{16}$CH_3 and PhOCH$_2$CH(CH$_2$CH_3)(CH$_2$)$_3$CH_3). ^{13}C NMR (101 MHz, CDCl$_3$) δ 173.3, 160.7, 160.5, 138.1, 106.5, 106.4, 101.0, 70.6, 68.2, 66.3, 59.5, 57.7, 57.4, 52.9, 52.9, 39.5, 32.3, 32.0, 30.5, 29.8, 29.8, 29.7, 29.6, 29.5, 29.4, 29.4, 29.2, 26.2, 24.0, 23.1, 22.8, 22.1, 14.2, 14.1, 11.2. Purity by HPLC: 99+%. MALDI-TOF MS m/z of $[M + H]^+$ calculated for $C_{42}H_{77}N_2O_5$: 689.6; Found: 690.3.

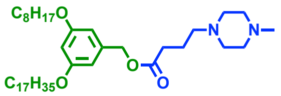

32c (IAJD230)

3-(Heptadecyloxy)-5-(octyloxy)benzyl 4-(4-methylpiperazin-1-yl)butanoate (32c, IAJD230). Compound **32c** was synthesized from compound **31b** (0.12 g, 0.18 mmol), 1-methylpiperazine (20 mg, 0.20 mmol) and K_2CO_3 (38 mg, 0.27 mmol) following a procedure similar to that used for the synthesis of compound **32a**. The title compound was obtained as a colorless oil (0.12 g, 98%). ^{1}H NMR (400 MHz, CDCl$_3$) δ 6.46 (br, 2 H, PhH), 6.39 (t, 1 H, PhH), 5.02 (s, 2 H, PhCH_2-), 3.92 (t, 4 H, PhOCH_2-), 2.61–2.22 (m, 15 H, -N(CH_2CH_2)$_2$N-, -NCH_2CH$_2$CH_2COO- and -NCH_3), 1.84 (m, 2 H, -OCOCH$_2$CH_2CH$_2$-), 1.74 (m, 4 H, PhOCH$_2$CH_2-), 1.43 (m, 4 H, PhOCH$_2$CH$_2$CH_2-), 1.26 (br, 34 H, PhOCH$_2$CH$_2$CH$_2$(CH_2)$_4$CH$_3$ and PhOCH$_2$CH$_2$CH$_2$(CH_2)$_{13}$CH$_3$), 0.88 (t, 6 H, -CH$_2$CH$_2$CH_3). ^{13}C NMR (101 MHz, CDCl$_3$) δ 173.3, 160.5, 138.1, 106.4, 100.9, 77.2, 68.1, 66.2, 57.5, 54.9, 52.7, 45.8, 32.2, 31.9, 31.8, 29.7, 29.7, 29.6, 29.6, 29.4, 29.4, 29.3, 29.2, 26.1, 26.0, 22.7, 22.1, 14.1. Purity by HPLC: 99+%. MALDI-TOF MS m/z of $[M + H]^+$ calculated for $C_{41}H_{75}N_2O_4$: 659.6; Found: 660.1.

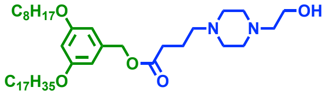

32d (IAJD231)

3-(Heptadecyloxy)-5-(octyloxy)benzyl 4-(4-(2-hydroxyethyl)piperazin-1-yl)butanoate (32d, IAJD231). Compound **32d** was synthesized from compound **31b** (0.12 g, 0.18 mmol), 1-(2-hydroxyethyl)piperazine (26 mg, 0.20 mmol) and K_2CO_3 (38 mg, 0.27 mmol) following a procedure similar to that used for the synthesis of compound **32a**. The title compound was obtained as a colorless oil (0.10 g, 90%). ^{1}H NMR (400 MHz, CDCl$_3$) δ 6.46 (br, 2 H, PhH), 6.39 (t, 1 H, PhH), 5.03 (s, 2 H, PhCH_2-), 3.92 (t, 4 H, PhOCH_2-), 3.65 (t, 2 H, -CH$_2$CH_2OH), 2.70–2.36 (m, 14 H, -N(CH_2CH_2)$_2$N-, -NCH_2CH$_2$CH_2COO- and -CH_2CH$_2$OH), 1.84 (m, 2 H, -OCOCH$_2$CH_2CH$_2$-), 1.76 (m, 4 H, PhOCH$_2$CH_2-), 1.43 (m, 4 H, PhOCH$_2$CH$_2$CH_2-), 1.26 (br, 34 H, PhOCH$_2$CH$_2$CH$_2$(CH_2)$_4$CH$_3$ and

PhOCH$_2$CH$_2$CH$_2$(C*H*$_2$)$_{13}$CH$_3$), 0.88 (t, 6 H, -CH$_2$CH$_2$C*H*$_3$). ^{13}C NMR (101 MHz, CDCl$_3$) δ 173.3, 160.5, 138.1, 106.4, 100.9, 77.2, 68.1, 66.2, 59.3, 57.6, 57.5, 53.0, 52.8, 32.2, 31.9, 29.7, 29.7, 29.6, 29.6, 29.4, 29.4, 29.3, 26.1, 22.7, 22.1, 14.1. Purity by HPLC: 99+%. MALDI-TOF MS m/z of $[M + H]^+$ calculated for $C_{42}H_{77}N_2O_5$: 689.6; Found: 690.4.

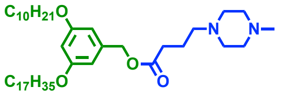

32e (IAJD232)

3-(Decyloxy)-5-(heptadecyloxy)benzyl 4-(4-methylpiperazin-1-yl)butanoate (32e, IAJD232). Compound **32e** was synthesized from compound **31c** (0.19 g, 0.21 mmol), 1-methylpiperazine (32 mg, 0.32 mmol) and K_2CO_3 (35 mg, 0.25 mmol) following a procedure similar to that used for the synthesis of compound **32a**. The title compound was obtained as a light-yellow oil (0.17 g, 80%). ^{1}H NMR (400 MHz, CDCl$_3$) δ 6.43 (br, 2 H, Ph*H*), 6.36 (t, 1 H, Ph*H*), 5.00 (s, 2 H, PhC*H*$_2$-), 3.89 (t, 4 H, PhOC*H*$_2$-), 2.59–2.24 (m, 15 H, -N(C*H*$_2$C*H*$_2$)$_2$N-, -NC*H*$_2$CH$_2$C*H*$_2$COO- and -NC*H*$_3$), 1.81 (m, 2 H, -OCOCH$_2$C*H*$_2$CH$_2$-), 1.73 (m, 4 H, PhOCH$_2$C*H*$_2$-), 1.43 (m, 4 H, PhOCH$_2$CH$_2$C*H*$_2$-), 1.24 (br, 38 H, PhOCH$_2$CH$_2$CH$_2$(C*H*$_2$)$_6$CH$_3$ and PhOCH$_2$CH$_2$CH$_2$(C*H*$_2$)$_{13}$CH$_3$), 0.88 (t, 6 H, -CH$_2$CH$_2$C*H*$_3$). ^{13}C NMR (101 MHz, CDCl$_3$) δ 173.2, 160.4, 138.1, 106.3, 100.9, 68.0, 66.1, 57.5, 55.0, 52.8, 45.8, 32.2, 31.9, 31.9, 29.7, 29.7, 29.6, 29.6, 29.6, 29.4, 29.4, 29.3, 29.3, 26.1, 22.7, 22.1, 14.1. Purity by HPLC: 99+%. MALDI-TOF MS m/z of $[M + H]^+$ calculated for $C_{43}H_{79}N_2O_4$: 687.6; Found: 688.3.

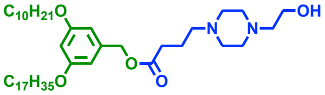

32f (IAJD233)

3-(Decyloxy)-5-(heptadecyloxy)benzyl 4-(4-(2-hydroxyethyl)piperazin-1-yl)butanoate (32f, IAJD233). Compound **32f** was synthesized from compound **31c** (0.19 g, 0.21 mmol), 1-(2-hydroxyethyl)piperazine (42 mg, 0.32 mmol) and K_2CO_3 (35 mg, 0.25 mmol) following a procedure similar to that used for the synthesis of compound **32a**. The title compound was obtained as a colorless oil (0.10 g, 90%). ^{1}H NMR (400 MHz, CDCl$_3$) δ 6.46 (br, 2 H, Ph*H*), 6.39 (t, 1 H, Ph*H*), 5.03 (s, 2 H, PhC*H*$_2$-), 3.91 (t, 4 H, PhOC*H*$_2$-), 3.64 (t, 2 H, -CH$_2$C*H*$_2$OH), 2.62–2.30 (m, 14 H, -N(C*H*$_2$C*H*$_2$)$_2$N-, -NC*H*$_2$CH$_2$C*H*$_2$COO- and -C*H*$_2$CH$_2$OH), 1.84 (m, 2 H, -OCOCH$_2$C*H*$_2$CH$_2$-), 1.76 (m, 4 H, PhOCH$_2$C*H*$_2$-), 1.43 (m, 4 H, PhOCH$_2$CH$_2$C*H*$_2$-), 1.25 (br, 38 H, PhOCH$_2$CH$_2$CH$_2$(C*H*$_2$)$_6$CH$_3$ and PhOCH$_2$CH$_2$CH$_2$(C*H*$_2$)$_{13}$CH$_3$), 0.88 (t, 6 H, -CH$_2$CH$_2$C*H*$_3$). ^{13}C NMR (101 MHz, CDCl$_3$) δ 173.3, 160.5, 138.1, 106.4, 100.9, 77.2, 68.1, 66.2, 59.3, 57.6, 57.5, 52.9, 52.8, 32.2, 31.9, 31.9, 29.7, 29.7, 29.6, 29.6,

29.6, 29.4, 29.4, 29.3, 29.3, 26.1, 22.7, 22.1, 14.1. Purity by HPLC: 99+%. MALDI-TOF MS m/z of [M + H]$^+$ calculated for $C_{44}H_{81}N_2O_5$: 717.6; Found: 717.6.

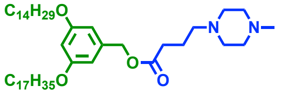

32g (IAJD234)

3-(Heptadecyloxy)-5-(tetradecyloxy)benzyl 4-(4-methylpiperazin-1-yl)butanoate (32g, IAJD234). Compound **32g** was synthesized from compound **31d** (0.20 g, 0.28 mmol), 1-methylpiperazine (30 mg, 0.30 mmol) and K_2CO_3 (42 mg, 0.30 mmol) following a procedure similar to that used for the synthesis of compound **32a**. The title compound was obtained as a light-yellow oil (0.17 g, 80%). ^{1}H NMR (400 MHz, $CDCl_3$) δ 6.46 (br, 2 H, Ph*H*), 6.39 (t, 1 H, Ph*H*), 5.02 (s, 2 H, PhC*H*$_2$-), 3.92 (t, 4 H, PhOC*H*$_2$-), 2.77–2.14 (m, 15 H, -N(C*H*$_2$C*H*$_2$)$_2$N-, -NC*H*$_2$CH$_2$C*H*$_2$COO- and -NC*H*$_3$), 1.83 (m, 2 H, -OCOCH$_2$C*H*$_2$CH$_2$-), 1.75 (m, 4 H, PhOCH$_2$C*H*$_2$-), 1.42 (m, 4 H, PhOCH$_2$CH$_2$C*H*$_2$-), 1.25 (br, 46 H, PhOCH$_2$CH$_2$CH$_2$(C*H*$_2$)$_{10}$CH$_3$ and PhOCH$_2$CH$_2$CH$_2$(C*H*$_2$)$_{13}$CH$_3$), 0.88 (t, 6 H, -CH$_2$CH$_2$C*H*$_3$). ^{13}C NMR (101 MHz, $CDCl_3$) δ 173.5, 160.6, 138.2, 106.5, 101.1, 68.2, 66.3, 57.7, 55.3, 53.2, 46.2, 32.4, 32.1, 29.8, 29.8, 29.8, 29.8, 29.5, 29.5, 29.4, 26.2, 22.8, 22.3, 14.3. Purity by HPLC: 99+%. MALDI-TOF MS m/z of [M + H]$^+$ calculated for $C_{47}H_{87}N_2O_4$: 743.7; Found: 745.5.

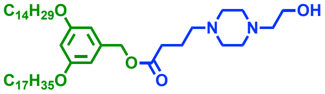

32h (IAJD235)

3-(Heptadecyloxy)-5-(tetradecyloxy)benzyl 4-(4-(2-hydroxyethyl)piperazin-1-yl)butanoate (32h, IAJD235). Compound **32h** was synthesized from compound **31d** (0.20 g, 0.28 mmol), 1-(2-hydroxyethyl)piperazine (39 mg, 0.30 mmol) and K_2CO_3 (42 mg, 0.30 mmol) following a procedure similar to that used for the synthesis of compound **32a**. The title compound was obtained as a light-yellow oil (0.16 g, 73%). ^{1}H NMR (400 MHz, $CDCl_3$) δ 6.45 (br, 2 H, Ph*H*), 6.38 (t, 1 H, Ph*H*), 5.02 (s, 2 H, PhC*H*$_2$-), 3.91 (t, 4 H, PhOC*H*$_2$-), 3.61 (t, 2 H, -CH$_2$C*H*$_2$OH), 2.74–2.12 (m, 14 H, -N(C*H*$_2$C*H*$_2$)$_2$N-, -NC*H*$_2$CH$_2$C*H*$_2$COO- and -C*H*$_2$CH$_2$OH), 1.83 (m, 2 H, -OCOCH$_2$C*H*$_2$CH$_2$-), 1.75 (m, 4 H, PhOCH$_2$C*H*$_2$-), 1.43 (m, 4 H, PhOCH$_2$CH$_2$C*H*$_2$-), 1.25 (br, 46 H, PhOCH$_2$CH$_2$CH$_2$(C*H*$_2$)$_{10}$CH$_3$ and PhOCH$_2$CH$_2$CH$_2$(C*H*$_2$)$_{13}$CH$_3$), 0.87 (t, 6 H, -CH$_2$CH$_2$C*H*$_3$). ^{13}C NMR (101 MHz, $CDCl_3$) δ 173.4, 160.6, 138.2, 106.5, 101.0, 68.2, 66.2, 59.4, 57.8, 57.6, 53.0, 53.0, 32.3, 32.0, 29.8, 29.8, 29.7, 29.7, 29.5, 29.5, 29.4, 26.2, 22.8, 22.2, 14.2. Purity by HPLC: 99+%. MALDI-TOF MS m/z of [M + H]$^+$ calculated for $C_{48}H_{89}N_2O_5$: 773.7; Found: 774.3.

3.6 Synthesis of Octadecane-Based IAJDs with 3,5-Nonsymmetric Alkyl Chains

Scheme S6. Synthesis of Octadecane-Based IAJDs with 3,5-Nonsymmetric Alkyl Chains

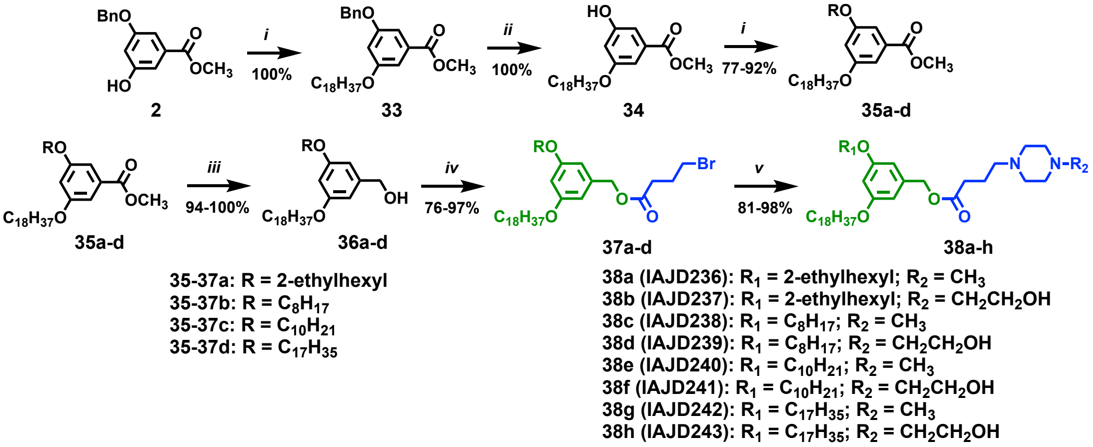

Reagents and conditions: (*i*) RBr/ROTs, K_2CO_3, DMF, 120 °C, 2 h; (*ii*) H_2, Pd/C, DCM, MeOH, 12 h; (*iii*) $LiAlH_4$, THF, 0–23 °C, 1 h; (*iv*) DCC, DPTS, DCM, 12 h; (*v*) K_2CO_3, MeCN, 95 °C, 3 h.

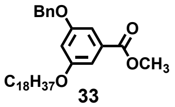

Methyl 3-(benzyloxy)-5-(octadecyloxy)benzoate (33). A mixture of compound **2** (2.00 g, 7.74 mmol, 1 equiv), 1-bromooctadecane (2.84 g, 8.52 mmol, 1.1 equiv), K_2CO_3 (2.14 g, 15.48 mmol, 2 equiv) and DMF (15 mL) was heated to 120 °C and stirred under N_2 atmosphere for 2 h. The reaction mixture was cooled to 23 °C and poured into ice/water (150 mL). The resulted white precipitates in the solution were filtered and collected. Then the precipitates were recrystallized from minimum acetone to afford the title compound as a white solid (3.28 g, 84%). ^{1}H NMR (400 MHz, $CDCl_3$) δ 7.48–7.29 (m, 5 H, Ph*H*), 7.27 (br, 1 H, Ph*H*), 7.20 (br, 1 H, Ph*H*), 6.73 (t, 1 H, Ph*H*), 5.08 (s, 2 H, $PhCH_2O$-), 3.97 (t, 2 H, $PhOCH_2$-), 3.90 (s, 3 H, $PhCOOCH_3$), 1.78 (m, 2 H, $PhOCH_2CH_2CH_2(CH_2)_{14}CH_3$), 1.44 (m, 2 H, $PhOCH_2CH_2CH_2(CH_2)_{14}CH_3$), 1.28 (m, 28 H, $PhOCH_2CH_2CH_2(CH_2)_{14}CH_3$), 0.89 (t, 3 H, $PhO(CH_2)_{17}CH_3$). ^{13}C NMR (101 MHz, $CDCl_3$) δ 167.0, 160.3, 159.9, 136.7, 132.1, 128.7, 128.2, 127.7, 108.3, 108.0, 107.1, 77.4, 70.4, 68.5, 52.3, 32.1, 29.8, 29.7, 29.7, 29.5, 29.3, 26.4, 26.1, 22.9, 22.8, 14.2. Mp = 65 °C.

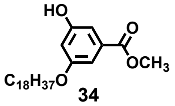

Methyl 3-hydroxy-5-(octadecyloxy)benzoate (34). Compound **33** (3.28 g, 6.42 mmol) was dissolved in a mixture of DCM (20 mL) and methanol (10 mL). Then Pd/C (0.16 g, 5 wt%) was added to the solution

and the flask was evacuated and filled with hydrogen for three times. Afterwards, the mixture was stirred at 23 °C under hydrogen atmosphere for 12 h. The reaction mixture was filtered through Celite, and the filter cake was washed with DCM. Evaporation of the solvent yielded the title compound as a white solid (2.70 g, 100%). ^{1}H NMR (400 MHz, $CDCl_3$) δ 7.13 (d, 2 H, Ph*H*), 6.64 (t, 1 H, Ph*H*), 3.95 (t, 2 H, PhOC*H*$_2$-), 3.89 (s, 3 H, PhCOOC*H*$_3$), 1.75 (m, 2 H, $PhOCH_2CH_2CH_2(CH_2)_{14}CH_3$), 1.44 (m, 2 H, $PhOCH_2CH_2CH_2(CH_2)_{14}CH_3$), 1.26 (m, 28 H, $PhOCH_2CH_2CH_2(CH_2)_{14}CH_3$), 0.88 (t, 3 H, $PhO(CH_2)_{17}CH_3$). ^{13}C NMR (101 MHz, $CDCl_3$) δ 167.0, 160.4, 157.0, 131.9, 109.0, 107.7, 107.1, 68.4, 52.2, 31.9, 29.7, 29.7, 29.6, 29.6, 29.4, 29.4, 29.2, 26.0, 22.7, 14.2. Mp = 99 °C.

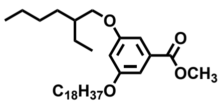

35a

Methyl 3-((2-ethylhexyl)oxy)-5-(octadecyloxy)benzoate (35a). A mixture of compound **34** (0.60 g, 1.43 mmol, 1 equiv), 2-ethylhexyl bromide (0.30 g, 1.57 mmol, 1.1 equiv), K_2CO_3 (0.39 g, 2.86 mmol, 2 equiv) and DMF (15 mL) was heated to 80 °C and stirred under N_2 atmosphere for 12 h. The reaction mixture was cooled to 23 °C and DMF was removed under reduced pressure. Then water (20 mL) was added, and the resulted mixture was extracted with DCM (20 mL) for three times. The organic phases were collected and dried over anhydrous $MgSO_4$. After filtration, the obtained filtrate was concentrated to around 3 mL and purified by column chromatography (SiO_2) with hexane/EtOAc = 20/1 as the eluent to give the title compound as a light-yellow oil (0.63 g, 83%). ^{1}H NMR (400 MHz, $CDCl_3$) δ 7.16 (br, 2 H, Ph*H*), 6.64 (t, 1 H, Ph*H*), 3.97 (t, 2 H, $PhOCH_2(CH_2)_{16}CH_3$), 3.90 (s, 3 H, PhCOOC*H*$_3$), 3.86 (m, 2 H, $PhOCH_2CH(CH_2CH_3)$-), 1.85–1.69 (m, 3 H, $PhOCH_2CH_2(CH_2)_{15}CH_3$ and $PhOCH_2CH(CH_2CH_3)(CH_2)_3CH_3$), 1.52–1.17 (m, 38 H, $PhOCH_2CH_2(CH_2)_{15}CH_3$ and $PhOCH_2CH(CH_2CH_3)(CH_2)_3CH_3$), 0.90 (m, 9 H, $PhO(CH_2)_{17}CH_3$ and $PhOCH_2CH(CH_2CH_3)(CH_2)_3CH_3$). ^{13}C NMR (101 MHz, $CDCl_3$) δ 167.0, 160.4, 160.2, 131.8, 107.7, 107.5, 106.6, 70.8, 68.3, 52.1, 39.4, 31.9, 30.5, 29.7, 29.7, 29.6, 29.6, 29.4, 29.2, 29.1, 26.0, 23.9, 23.0, 22.7, 14.1, 14.1, 11.1.

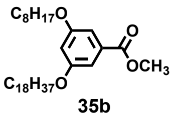

35b

Methyl 3-(octadecyloxy)-5-(octyloxy)benzoate (35b). A mixture of compound **34** (0.50 g, 1.19 mmol, 1 equiv), 1-bromooctane (0.25 g, 1.31 mmol, 1.1 equiv), K_2CO_3 (0.33 g, 2.38 mmol, 2 equiv) and DMF (15 mL) was heated to 120 °C and stirred under N_2 atmosphere for 2 h. The reaction mixture was cooled to 23 °C and poured into ice/water (150 mL). The resulted white precipitates in the solution were filtered

and collected. Then the precipitates were recrystallized from minimum acetone to afford the title compound as a white solid (0.48 g, 76%). ^{1}H NMR (400 MHz, $CDCl_3$) δ 7.15 (br, 2 H, Ph*H*), 6.63 (t, 1 H, Ph*H*), 3.96 (t, 4 H, PhOC*H*$_2$CH$_2$CH$_2$-), 3.89 (s, 3 H, PhCOOC*H*$_3$), 1.77 (m, 4 H, PhOCH$_2$C*H*$_2$CH$_2$-), 1.44 (m, 4 H, PhOCH$_2$CH$_2$C*H*$_2$-), 1.26 (m, 36 H, PhOCH$_2$CH$_2$CH$_2$(C*H*$_2$)$_4$CH$_3$ and PhOCH$_2$CH$_2$CH$_2$(C*H*$_2$)$_{14}$CH$_3$), 0.88 (t, 6 H, PhO(CH$_2$)$_7$C*H*$_3$ and PhO(CH$_2$)$_{17}$C*H*$_3$). ^{13}C NMR (101 MHz, $CDCl_3$) δ 167.1, 160.3, 132.0, 107.8, 106.7, 77.4, 68.5, 52.3, 32.1, 31.9, 29.8, 29.8, 29.7, 29.7, 29.6, 29.5, 29.5, 29.4, 29.3, 26.2, 22.8, 22.8, 14.2, 14.2. Mp = 58 °C.

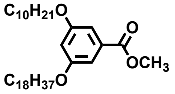

35c

Methyl 3-(decyloxy)-5-(octadecyloxy)benzoate (35c). Compound **35c** was synthesized from compound **34** (0.50 g, 1.19 mmol, 1 equiv), 1-bromodecane (0.29 g, 1.31 mmol, 1.1 equiv) and K_2CO_3 (0.33 g, 2.38 mmol, 2 equiv) following a procedure similar to that used for the synthesis of compound **35b**. The title compound was obtained as a white solid (0.48 g, 72%). ^{1}H NMR (400 MHz, $CDCl_3$) δ 7.15 (br, 2 H, Ph*H*), 6.64 (t, 1 H, Ph*H*), 3.96 (t, 4 H, PhOC*H*$_2$CH$_2$CH$_2$-), 3.89 (s, 3 H, PhCOOC*H*$_3$), 1.77 (m, 4 H, PhOCH$_2$C*H*$_2$CH$_2$-), 1.43 (m, 4 H, PhOCH$_2$CH$_2$C*H*$_2$-), 1.26 (m, 40 H, PhOCH$_2$CH$_2$CH$_2$(C*H*$_2$)$_6$CH$_3$ and PhOCH$_2$CH$_2$CH$_2$(C*H*$_2$)$_{14}$CH$_3$), 0.88 (t, 6 H, PhO(CH$_2$)$_9$C*H*$_3$ and PhO(CH$_2$)$_{17}$C*H*$_3$). ^{13}C NMR (101 MHz, $CDCl_3$) δ 167.1, 160.3, 132.0, 107.8, 106.7, 77.4, 68.5, 68.1, 52.3, 32.1, 32.0, 29.9, 29.8, 29.8, 29.7, 29.7, 29.7, 29.7, 29.6, 29.5, 29.5, 29.3, 26.3, 26.2, 22.8, 14.2. Mp = 60 °C.

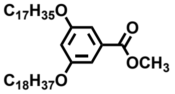

35d

Methyl 3-(heptadecyloxy)-5-(octadecyloxy)benzoate (35d). Compound **35d** was synthesized from compound **34** (0.50 g, 1.19 mmol, 1 equiv), $C_{17}H_{35}OTs$ (0.54 g, 1.31 mmol, 1.1 equiv) and K_2CO_3 (0.33 g, 2.38 mmol, 2 equiv) following a procedure similar to that used for the synthesis of compound **35b**. The title compound was obtained as a white solid (0.70 g, 89%). ^{1}H NMR (400 MHz, $CDCl_3$) δ 7.15 (br, 2 H, Ph*H*), 6.63 (t, 1 H, Ph*H*), 3.96 (t, 4 H, PhOC*H*$_2$CH$_2$CH$_2$-), 3.89 (s, 3 H, PhCOOC*H*$_3$), 1.77 (m, 4 H, PhOCH$_2$C*H*$_2$CH$_2$-), 1.43 (m, 4 H, PhOCH$_2$CH$_2$C*H*$_2$-), 1.26 (m, 54 H, PhOCH$_2$CH$_2$CH$_2$(C*H*$_2$)$_{13}$CH$_3$ and PhOCH$_2$CH$_2$CH$_2$(C*H*$_2$)$_{14}$CH$_3$), 0.88 (t, 6 H, PhO(CH$_2$)$_{16}$C*H*$_3$ and PhO(CH$_2$)$_{17}$C*H*$_3$). ^{13}C NMR (101 MHz, $CDCl_3$) δ 167.1, 160.3, 132.0, 107.8, 106.7, 68.5, 52.3, 34.1, 33.0, 32.1, 29.9, 29.8, 29.8, 29.8, 29.7, 29.7, 29.6, 29.5, 29.3, 28.9, 28.3, 26.2, 22.8, 14.3. Mp = 72 °C.

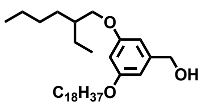

36a

(3-((2-Ethylhexyl)oxy)-5-(octadecyloxy)phenyl)methanol (36a). Compound **35a** (0.30 g, 0.56 mmol, 1 equiv) was dissolved in 5 mL dry THF, which was added dropwise to a slurry of $LiAlH_4$ (22 mg, 0.56 mmol, 1 equiv) in dry THF (5 mL) at 0 °C under N_2 atmosphere. Afterwards, the resulted mixture was stirred at 23 °C for 1 h. Finally, the reaction was quenched by the successive addition of water (0.2 mL), 15% NaOH aqueous solution (0.2 mL) and water (1.0 mL). White precipitates were formed in the mixture and filtered out. The filtrate was dried over anhydrous $MgSO_4$ and filtered. The solvent was removed under reduced pressure to afford the title compound as a colorless oil (0.24 g, 84%). ^{1}H NMR (400 MHz, $CDCl_3$) δ 6.50 (d, 2 H, PhH), 6.38 (t, 1 H, PhH), 4.61 (s, 2 H, $PhCH_2OH$), 3.94 (t, 2 H, $PhOCH_2(CH_2)_{16}CH_3$), 3.82 (m, 2 H, $PhOCH_2CH(CH_2CH_3)$-), 1.72 (m, 3 H, $PhOCH_2CH_2(CH_2)_{15}CH_3$ and $PhOCH_2CH(CH_2CH_3)(CH_2)_3CH_3$), 1.52–1.17 (m, 38 H, $PhOCH_2CH_2(CH_2)_{15}CH_3$ and $PhOCH_2CH(CH_2CH_3)(CH_2)_3CH_3$), 0.91 (m, 9 H, $PhO(CH_2)_{17}CH_3$ and $PhOCH_2CH(CH_2CH_3)(CH_2)_3CH_3$). ^{13}C NMR (101 MHz, $CDCl_3$) δ 161.0, 160.7, 143.3, 105.3, 105.1, 100.7, 70.7, 68.2, 65.6, 62.9, 39.5, 32.1, 30.7, 29.8, 29.8, 29.8, 29.7, 29.5, 29.5, 29.4, 29.2, 26.2, 24.0, 23.2, 22.8, 14.3, 14.2, 11.2.

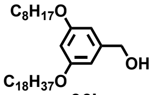

36b

(3-(Octadecyloxy)-5-(octyloxy)phenyl)methanol (36b). Compound **36b** was synthesized from compound **35b** (0.50 g, 0.94 mmol, 1 equiv) and $LiAlH_4$ (36 mg, 0.94 mmol, 1 equiv) following a procedure similar to that used for the synthesis of compound **36a**. The title compound was obtained as a white solid (0.46 g, 97%). ^{1}H NMR (400 MHz, $CDCl_3$) δ 6.49 (br, 2 H, PhH), 6.37 (t, 1 H, PhH), 4.59 (s, 2 H, $PhCH_2OH$), 3.93 (t, 4 H, $PhOCH_2CH_2CH_2$-), 1.77 (m, 4 H, $PhOCH_2CH_2CH_2$-), 1.44 (m, 4 H, $PhOCH_2CH_2CH_2$-), 1.29 (m, 36 H, $PhOCH_2CH_2CH_2(CH_2)_4CH_3$ and $PhOCH_2CH_2CH_2(CH_2)_{14}CH_3$), 0.89 (t, 6 H, $PhO(CH_2)_7CH_3$ and $PhO(CH_2)_{17}CH_3$). ^{13}C NMR (101 MHz, $CDCl_3$) δ 160.6, 143.4, 105.2, 100.7, 77.4, 68.2, 65.4, 32.1, 32.0, 29.8, 29.8, 29.8, 29.7, 29.5, 29.5, 29.4, 29.4, 26.2, 22.8, 22.8, 14.2, 14.2. Mp = 37 °C.

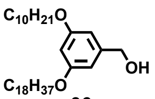

36c

(3-(Decyloxy)-5-(octadecyloxy)phenyl)methanol (36c). Compound **36c** was synthesized from compound **35c** (0.44 g, 0.78 mmol, 1 equiv) and $LiAlH_4$ (30 mg, 0.78 mmol, 1 equiv) following a

procedure similar to that used for the synthesis of compound **36a**. The title compound was obtained as a white solid (0.40 g, 96%). ^{1}H NMR (400 MHz, CDCl$_3$) δ 6.49 (br, 2 H, Ph*H*), 6.37 (t, 1 H, Ph*H*), 4.60 (s, 2 H, PhC*H*$_2$OH), 3.93 (t, 4 H, PhOC*H*$_2$CH$_2$CH$_2$-), 1.76 (m, 4 H, PhOCH$_2$C*H*$_2$CH$_2$-), 1.44 (m, 4 H, PhOCH$_2$CH$_2$C*H*$_2$-), 1.28 (m, 40 H, PhOCH$_2$CH$_2$CH$_2$(C*H*$_2$)$_6$CH$_3$ and PhOCH$_2$CH$_2$CH$_2$(C*H*$_2$)$_{14}$CH$_3$), 0.88 (t, 6 H, PhO(CH$_2$)$_9$C*H*$_3$ and PhO(CH$_2$)$_{17}$C*H*$_3$). ^{13}C NMR (101 MHz, CDCl$_3$) δ 160.7, 160.7, 143.4, 105.2, 100.7, 77.4, 68.2, 65.5, 32.1, 32.1, 29.9, 29.8, 29.8, 29.7, 29.7, 29.6, 29.5, 29.5, 29.5, 29.4, 26.2, 22.8, 14.3. Mp = 42 °C.

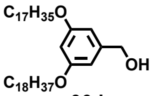

36d

(3-(Heptadecyloxy)-5-(octadecyloxy)phenyl)methanol (36d). Compound **36d** was synthesized from compound **35d** (0.70 g, 1.06 mmol, 1 equiv) and LiAlH$_4$ (40 mg, 1.06 mmol, 1 equiv) following a procedure similar to that used for the synthesis of compound **36a**. The title compound was obtained as a white solid (0.46 g, 69%). ^{1}H NMR (400 MHz, CDCl$_3$) δ 6.49 (br, 2 H, Ph*H*), 6.37 (t, 1 H, Ph*H*), 4.61 (s, 2 H, PhC*H*$_2$OH), 3.93 (t, 4 H, PhOC*H*$_2$CH$_2$CH$_2$-), 1.76 (m, 4 H, PhOCH$_2$C*H*$_2$CH$_2$-), 1.44 (m, 4 H, PhOCH$_2$CH$_2$C*H*$_2$-), 1.29 (m, 54 H, PhOCH$_2$CH$_2$CH$_2$(C*H*$_2$)$_{13}$CH$_3$ and PhOCH$_2$CH$_2$CH$_2$(C*H*$_2$)$_{14}$CH$_3$), 0.88 (t, 6 H, PhO(CH$_2$)$_{16}$C*H*$_3$ and PhO(CH$_2$)$_{17}$C*H*$_3$). ^{13}C NMR (101 MHz, CDCl$_3$) δ 160.7, 160.6, 143.4, 125.7, 105.2, 100.7, 68.2, 65.6, 32.1, 30.5, 29.9, 29.8, 29.8, 29.7, 29.6, 29.5, 29.5, 29.4, 26.2, 25.9, 22.8, 14.3. Mp = 62 °C.

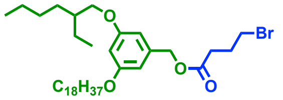

37a

3-((2-Ethylhexyl)oxy)-5-(octadecyloxy)benzyl 4-bromobutanoate (37a). Compound **36a** (0.24 g, 0.48 mmol, 1 equiv), 4-bromobutyric acid (0.09 g, 0.52 mmol, 1.1 equiv) and DPTS (0.15 g, 0.52 mmol, 1.1 equiv) were dissolved in 8 mL DCM. DCC (0.20 g, 0.95 mmol, 2 equiv) was added into the above mixture. The reaction mixture was stirred at 23 °C for 12 h. Afterwards, the resulted precipitates (urea) were filtered out from the reaction mixture. The filtrate was concentrated to around 3 mL and purified by column chromatography (SiO$_2$) with hexane/DCM = 1/1 as the mobile phase to give the title compound as a colorless oil (0.29 g, 93%). ^{1}H NMR (400 MHz, CDCl$_3$) δ 6.47 (d, 2 H, Ph*H*), 6.42 (t, 1 H, Ph*H*), 5.05 (s, 2 H, PhC*H*$_2$OCO), 3.93 (t, 2 H, PhOC*H*$_2$(CH$_2$)$_{16}$CH$_3$), 3.82 (m, 2 H,PhOC*H*$_2$CH(CH$_2$CH$_3$)-), 3.47 (t, 2 H, -OCOCH$_2$CH$_2$C*H*$_2$Br), 2.57 (t, 2 H, -OCOC*H*$_2$CH$_2$CH$_2$Br), 2.21 (m, 2 H, -OCOCH$_2$C*H*$_2$CH$_2$Br), 1.83–1.67 (m, 3 H, PhOCH$_2$C*H*$_2$(CH$_2$)$_{15}$CH$_3$ and PhOCH$_2$C*H*(CH$_2$CH$_3$)(CH$_2$)$_3$CH$_3$), 1.45–1.21 (m, 38 H,

$PhOCH_2CH_2(CH_2)_{15}CH_3$ and $PhOCH_2CH(CH_2CH_3)(CH_2)_3CH_3$), 0.91 (m, 9 H, $PhO(CH_2)_{17}CH_3$ and $PhOCH_2CH(CH_2CH_3)(CH_2)_3CH_3$). ^{13}C NMR (101 MHz, $CDCl_3$) δ 172.4, 160.9, 160.6, 137.9, 106.6, 106.4, 101.2, 70.7, 68.2, 66.6, 39.5, 32.8, 32.6, 32.1, 30.7, 29.8, 29.8, 29.8, 29.7, 29.5, 29.5, 29.4, 29.2, 27.9, 26.2, 24.0, 23.2, 22.8, 14.3, 14.2, 11.3.

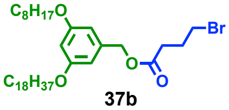

37b

3-(Octadecyloxy)-5-(octyloxy)benzyl 4-bromobutanoate (37b). Compound **37b** was synthesized from compound **36b** (0.46 g, 0.91 mmol, 1 equiv), 4-bromobutyric acid (0.17 g, 1.00 mmol, 1.1 equiv), DPTS (0.30 g, 1.00 mmol, 1.1 equiv) and DCC (0.38 g, 1.82 mmol, 2 equiv) following a procedure similar to that used for the synthesis of compound **37a**. The title compound was obtained as a colorless oil (0.37 g, 62%). ^1H NMR (400 MHz, $CDCl_3$) δ 6.47 (br, 2 H, Ph*H*), 6.41 (t, 1 H, Ph*H*), 5.05 (s, 2 H, $PhCH_2OCO$), 3.93 (t, 4 H, $PhOCH_2CH_2CH_2$-), 3.46 (t, 2 H, -$OCOCH_2CH_2CH_2Br$), 2.56 (t, 2 H, -$OCOCH_2CH_2CH_2Br$), 2.20 (m, 2 H, -$OCOCH_2CH_2CH_2Br$), 1.77 (m, 4 H, $PhOCH_2CH_2CH_2$-), 1.44 (m, 4 H, $PhOCH_2CH_2CH_2$-), 1.27 (m, 36 H, $PhOCH_2CH_2CH_2(CH_2)_4CH_3$ and $PhOCH_2CH_2CH_2(CH_2)_{14}CH_3$), 0.89 (t, 6 H, $PhO(CH_2)_7CH_3$ and $PhO(CH_2)_{17}CH_3$). ^{13}C NMR (101 MHz, $CDCl_3$) δ 172.4, 160.6, 137.9, 106.5, 101.2, 68.2, 66.5, 32.7, 32.6, 32.1, 32.0, 30.5, 29.8, 29.8, 29.8, 29.7, 29.5, 29.5, 29.4, 27.9, 26.2, 22.8, 22.8, 14.3, 14.2.

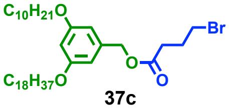

37c

3-(Decyloxy)-5-(octadecyloxy)benzyl 4-bromobutanoate (37c). Compound **37c** was synthesized from compound **36c** (0.40 g, 0.75 mmol, 1 equiv), 4-bromobutyric acid (0.14 g, 0.83 mmol, 1.1 equiv), DPTS (0.24 g, 0.83 mmol, 1.1 equiv) and DCC (0.31 g, 1.50 mmol, 2 equiv) following a procedure similar to that used for the synthesis of compound **37a**. The title compound was obtained as a colorless oil (0.35 g, 68%). ^1H NMR (400 MHz, $CDCl_3$) δ 6.46 (br, 2 H, Ph*H*), 6.40 (t, 1 H, Ph*H*), 5.05 (s, 2 H, $PhCH_2OCO$), 3.93 (t, 4 H, $PhOCH_2CH_2CH_2$-), 3.47 (t, 2 H, -$OCOCH_2CH_2CH_2Br$), 2.57 (t, 2 H, -$OCOCH_2CH_2CH_2Br$), 2.20 (m, 2 H, -$OCOCH_2CH_2CH_2Br$), 1.76 (m, 4 H, $PhOCH_2CH_2CH_2$-), 1.44 (m, 4 H, $PhOCH_2CH_2CH_2$-), 1.27 (m, 40 H, $PhOCH_2CH_2CH_2(CH_2)_6CH_3$ and $PhOCH_2CH_2CH_2(CH_2)_{14}CH_3$), 0.89 (t, 6 H, $PhO(CH_2)_9CH_3$ and $PhO(CH_2)_{17}CH_3$). ^{13}C NMR (101 MHz, $CDCl_3$) δ 172.4, 160.6, 137.9, 106.5, 101.2, 68.2, 66.5, 32.7, 32.6, 32.1, 32.0, 29.8, 29.8, 29.8, 29.7, 29.7, 29.5, 29.5, 29.5, 29.4, 27.9, 26.2, 22.8, 14.3.

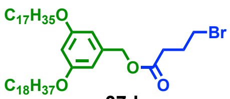

37d

3-(Heptadecyloxy)-5-(octadecyloxy)benzyl 4-bromobutanoate (37d). Compound **37d** was synthesized from compound **36d** (0.46 g, 0.73 mmol, 1 equiv), 4-bromobutyric acid (0.13 g, 0.80 mmol, 1.1 equiv), DPTS (0.24 g, 0.80 mmol, 1.1 equiv) and DCC (0.30 g, 1.46 mmol, 2 equiv) following a procedure similar to that used for the synthesis of compound **37a**. The title compound was obtained as a white solid (0.45 g, 79%). ^{1}H NMR (400 MHz, $CDCl_3$) δ 6.46 (br, 2 H, Ph*H*), 6.40 (t, 1 H, Ph*H*), 5.05 (s, 2 H, PhCH_2OCO), 3.93 (t, 4 H, PhOC$H_2$$CH_2CH_2$-), 3.47 (t, 2 H, -OCO$CH_2CH_2CH_2$Br), 2.54 (t, 2 H, -OCOC$H_2$$CH_2CH_2$Br), 2.20 (m, 2 H, -OCO$CH_2CH_2$$CH_2$Br), 1.76 (m, 4 H, PhO$CH_2CH_2$$CH_2$-), 1.45 (m, 4 H, PhO$CH_2CH_2CH_2$-), 1.27 (m, 54 H, PhO$CH_2CH_2CH_2$(C$H_2$)$_{13}$$CH_3$ and PhO$CH_2CH_2CH_2$(CH_2)$_{14}$$CH_3$), 0.88 (t, 6 H, PhO($CH_2$)$_{16}CH_3$ and PhO(CH_2)$_{17}$CH_3). ^{13}C NMR (101 MHz, $CDCl_3$) δ 172.4, 160.6, 137.9, 106.5, 101.2, 68.2, 66.5, 32.8, 32.6, 32.1, 32.0, 29.8, 29.8, 29.8, 29.7, 29.7, 29.7, 29.5, 29.5, 29.5, 29.4, 27.9, 26.2, 22.8, 14.3. Mp = 45 °C.

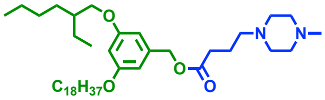

38a (IAJD236)

3-((2-Ethylhexyl)oxy)-5-(octadecyloxy)benzyl 4-(4-methylpiperazin-1-yl)butanoate (38a, IAJD236). A mixture of compound **37a** (0.14 g, 0.21 mmol, 1.0 equiv), 1-methylpiperazine (23 mg, 0.23 mmol, 1.1 equiv), K_2CO_3 (35 mg, 0.25 mmol, 1.2 equiv) and MeCN (10 mL) was heated to 95 °C and stirred for 3 h. The reaction mixture was cooled to 23 °C and MeCN was removed under reduced pressure. Then water (20 mL) was added, and the resulted mixture was extracted with DCM (20 mL) for three times. The organic phases were collected and dried over anhydrous $MgSO_4$. After filtration, the obtained filtrate was concentrated to around 3 mL and purified by column chromatography (SiO_2) with DCM/MeOH = 30/1 and 15/1 as the eluent. Then the obtained product was dissolved in DCM (20 mL), which was washed by $NaHCO_3$ solution (2%, 20 mL). The aqueous phase was extracted by DCM (20 mL) for another two times. The organic phases were combined and dried over anhydrous $MgSO_4$. Filtration and evaporation of the solvent yielded the title compound as a colorless oil (0.14 g, 99%). ^{1}H NMR (400 MHz, $CDCl_3$) δ 6.44 (br, 2 H, Ph*H*), 6.38 (t, 1 H, Ph*H*), 5.01 (s, 2 H, PhCH_2-), 3.90 (t, 4 H, PhOCH_2-), 3.79 (m, 2 H, PhOCH_2CH(CH_2CH_3)-), 2.63–2.25 (m, 15 H, -N(CH_2CH_2)$_2$N-, -NC$H_2$$CH_2CH_2$COO- and -NC$H_3$), 1.82 (m, 2 H, -OCO$CH_2CH_2$$CH_2$-), 1.71 (m, 3 H, PhO$CH_2CH_2$($CH_2$)$_{15}$$CH_3$ and PhOCH_2C*H*(CH_2CH_3)(CH_2)$_3$$CH_3$), 1.48–1.17 (m, 38 H, PhOCH_2CH_2(CH_2)$_{15}$$CH_3$ and

PhOCH$_2$CH(CH_2CH$_3$)(CH_2)$_3$CH$_3$), 0.88 (m, 9 H, PhO(CH$_2$)$_{17}$CH_3 and PhOCH$_2$CH(CH$_2$CH_3)(CH$_2$)$_3$CH_3). ^{13}C NMR (101 MHz, CDCl$_3$) δ 173.3, 160.8, 160.5, 138.1, 106.5, 106.4, 101.0, 70.6, 68.1, 66.2, 57.5, 55.1, 52.8, 45.9, 39.5, 32.3, 32.0, 30.6, 29.8, 29.8, 29.7, 29.7, 29.5, 29.4, 29.4, 29.2, 26.2, 24.0, 23.1, 22.8, 22.2, 14.2, 14.2, 11.2. Purity by HPLC: 99+%. MALDI-TOF MS m/z of $[M + H]^+$ calculated for $C_{42}H_{77}N_2O_4$: 673.6; Found: 674.1.

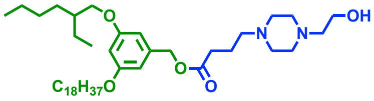

38b (IAJD237)

3-((2-Ethylhexyl)oxy)-5-(octadecyloxy)benzyl 4-(4-(2-hydroxyethyl)piperazin-1-yl)butanoate (38b, IAJD237). Compound **38b** was synthesized from compound **37a** (0.14 g, 0.21 mmol), 1-(2-hydroxyethyl)piperazine (30 mg, 0.23 mmol) and K_2CO_3 (35 mg, 0.25 mmol) following a procedure similar to that used for the synthesis of compound **38a**. The title compound was obtained as a colorless oil (0.14 g, 98%). ^{1}H NMR (400 MHz, CDCl$_3$) δ 6.45 (br, 2 H, Ph*H*), 6.39 (t, 1 H, Ph*H*), 5.02 (s, 2 H, PhCH_2-), 3.91 (t, 4 H, PhOCH_2-), 3.80 (m, 2 H, PhOCH_2CH(CH$_2$CH$_3$)-), 3.61 (t, 2 H, -CH$_2$CH_2OH), 2.73–2.32 (m, 14 H, -N(CH_2CH_2)$_2$N-, -NCH_2CH$_2$CH_2COO- and -NCH_2-), 1.83 (m, 2 H, -OCOCH$_2$CH_2CH$_2$-), 1.73 (m, 3 H, PhOCH$_2$CH_2(CH$_2$)$_{15}$CH$_3$ and PhOCH$_2$C*H*(CH$_2$CH$_3$)(CH$_2$)$_3$CH$_3$), 1.62–1.17 (m, 38 H, PhOCH$_2$CH$_2$(CH_2)$_{15}$CH$_3$ and PhOCH$_2$CH(CH_2CH$_3$)(CH_2)$_3$CH$_3$), 0.88 (m, 9 H, PhO(CH$_2$)$_{17}$CH_3 and PhOCH$_2$CH(CH$_2$CH_3)(CH$_2$)$_3$CH_3). ^{13}C NMR (101 MHz, CDCl$_3$) δ 173.4, 160.8, 160.6, 138.1, 106.6, 106.6, 106.5, 101.0, 77.4, 70.7, 68.2, 66.3, 59.4, 57.7, 57.6, 53.0, 52.9, 39.5, 32.3, 32.1, 30.7, 29.8, 29.8, 29.8, 29.8, 29.7, 29.5, 29.5, 29.4, 29.2, 26.2, 24.0, 23.2, 22.8, 22.2, 14.2, 14.2, 11.2. Purity by HPLC: 99+%. MALDI-TOF MS m/z of $[M + H]^+$ calculated for $C_{43}H_{79}N_2O_5$: 703.6; Found: 703.8.

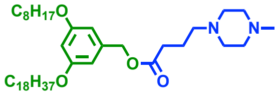

38c (IAJD238)

3-(Octadecyloxy)-5-(octyloxy)benzyl 4-(4-methylpiperazin-1-yl)butanoate (38c, IAJD238). Compound **38c** was synthesized from compound **37b** (0.17 g, 0.26 mmol), 1-methylpiperazine (29 mg, 0.29 mmol) and K_2CO_3 (43 mg, 0.31 mmol) following a procedure similar to that used for the synthesis of compound **38a**. The title compound was obtained as a colorless oil (0.18 g, 100%). ^{1}H NMR (400 MHz, CDCl$_3$) δ 6.45 (br, 2 H, Ph*H*), 6.38 (t, 1 H, Ph*H*), 5.01 (s, 2 H, PhCH_2-), 3.91 (t, 4 H, PhOCH_2-), 2.65–2.29 (m, 15 H, -N(CH_2CH_2)$_2$N-, -NCH_2CH$_2$CH_2COO- and -NCH_3), 1.83 (m, 2 H, -OCOCH$_2$CH_2CH$_2$-), 1.74 (m, 4 H, PhOCH$_2$CH_2-), 1.43 (m, 4 H, PhOCH$_2$CH$_2$CH_2-), 1.27 (br, 36 H, PhOCH$_2$CH$_2$CH$_2$(CH_2)$_4$CH$_3$ and PhOCH$_2$CH$_2$CH$_2$(CH_2)$_{14}$CH$_3$), 0.87 (t, 6 H, -CH$_2$CH$_2$CH_3). ^{13}C NMR

(101 MHz, $CDCl_3$) δ 173.3, 160.5, 138.1, 106.4, 100.9, 68.1, 66.2, 57.5, 54.9, 53.5, 52.7, 45.8, 32.2, 32.0, 31.9, 29.7, 29.7, 29.7, 29.6, 29.4, 29.4, 29.3, 29.3, 26.1, 22.7, 22.7, 22.1, 14.1, 14.1. Purity by HPLC: 99+%. MALDI-TOF MS m/z of $[M + H]^+$ calculated for $C_{42}H_{77}N_2O_4$: 673.6; Found: 674.1.

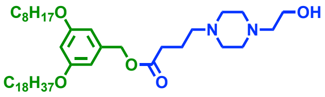

38d (IAJD239)

3-(Octadecyloxy)-5-(octyloxy)benzyl 4-(4-(2-hydroxyethyl)piperazin-1-yl)butanoate (38d, IAJD239). Compound **38d** was synthesized from compound **37b** (0.17 g, 0.26 mmol), 1-(2-hydroxyethyl)piperazine (38 mg, 0.29 mmol) and K_2CO_3 (43 mg, 0.31 mmol) following a procedure similar to that used for the synthesis of compound **38a**. The title compound was obtained as a colorless oil (0.17 g, 98%). ^1H NMR (400 MHz, $CDCl_3$) δ 6.45 (br, 2 H, Ph*H*), 6.39 (t, 1 H, Ph*H*), 5.02 (s, 2 H, PhCH_2-), 3.91 (t, 4 H, PhOCH_2-), 3.60 (t, 2 H, -CH_2CH_2OH), 2.64–2.25 (m, 14 H, -N(CH_2CH_2)$_2$N-, -N$CH_2CH_2CH_2$COO- and -CH_2CH_2OH), 1.83 (m, 2 H, -OCO$CH_2CH_2CH_2$-), 1.75 (m, 4 H, PhOCH_2CH_2-), 1.43 (m, 4 H, PhO$CH_2CH_2CH_2$-), 1.27 (br, 36 H, PhO$CH_2CH_2CH_2(CH_2)_4CH_3$ and PhO$CH_2CH_2CH_2(CH_2)_{14}CH_3$), 0.87 (t, 6 H, -$CH_2CH_2CH_3$). ^{13}C NMR (101 MHz, $CDCl_3$) δ 173.4, 160.6, 138.2, 106.5, 101.0, 68.2, 66.3, 59.4, 57.8, 57.6, 53.1, 53.0, 32.3, 32.0, 29.8, 29.8, 29.7, 29.7, 29.7, 29.5, 29.5, 29.4, 26.2, 22.8, 22.2, 14.2. Purity by HPLC: 99+%. MALDI-TOF MS m/z of $[M + H]^+$ calculated for $C_{43}H_{79}N_2O_5$: 703.6; Found: 703.9.

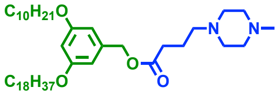

38e (IAJD240)

3-(Decyloxy)-5-(octadecyloxy)benzyl 4-(4-methylpiperazin-1-yl)butanoate (38e, IAJD240). Compound **38e** was synthesized from compound **37c** (0.17 g, 0.25 mmol), 1-methylpiperazine (28 mg, 0.28 mmol) and K_2CO_3 (42 mg, 0.30 mmol) following a procedure similar to that used for the synthesis of compound **38a**. The title compound was obtained as a colorless oil (0.18 g, 100%). ^1H NMR (400 MHz, $CDCl_3$) δ 6.45 (br, 2 H, Ph*H*), 6.38 (t, 1 H, Ph*H*), 5.02 (s, 2 H, PhCH_2-), 3.91 (t, 4 H, PhOCH_2-), 2.59–2.26 (m, 15 H, -N(CH_2CH_2)$_2$N-, -N$CH_2CH_2CH_2$COO- and -NCH_3), 1.84 (m, 2 H, -OCO$CH_2CH_2CH_2$-), 1.75 (m, 4 H, PhOCH_2CH_2-), 1.42 (m, 4 H, PhO$CH_2CH_2CH_2$-), 1.26 (br, 40 H, PhO$CH_2CH_2CH_2(CH_2)_6CH_3$ and PhO$CH_2CH_2CH_2(CH_2)_{14}CH_3$), 0.88 (t, 6 H, -$CH_2CH_2CH_3$). ^{13}C NMR (101 MHz, $CDCl_3$) δ 173.4, 160.6, 138.2, 106.5, 101.0, 77.4, 68.2, 66.3, 57.6, 55.2, 53.0, 46.0, 32.4, 32.1, 32.0, 29.8, 29.8, 29.7, 29.7, 29.7, 29.5, 29.5, 29.4, 29.4, 26.2, 22.8, 22.3, 14.2. Purity by HPLC: 99+%. MALDI-TOF MS m/z of $[M + H]^+$ calculated for $C_{44}H_{81}N_2O_4$: 701.6; Found: 701.4.

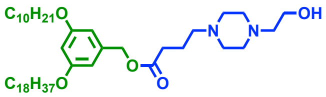

38f (IAJD241)

3-(Decyloxy)-5-(octadecyloxy)benzyl 4-(4-(2-hydroxyethyl)piperazin-1-yl)butanoate (38f, IAJD241). Compound **38f** was synthesized from compound **37c** (0.17 g, 0.25 mmol), 1-(2-hydroxyethyl)piperazine (36 mg, 0.28 mmol) and K_2CO_3 (42 mg, 0.30 mmol) following a procedure similar to that used for the synthesis of compound **38a**. The title compound was obtained as a colorless oil (0.18 g, 98%). ^1H NMR (400 MHz, $CDCl_3$) δ 6.44 (br, 2 H, Ph*H*), 6.37 (t, 1 H, Ph*H*), 5.01 (s, 2 H, PhCH_2-), 3.90 (t, 4 H, PhOCH_2-), 3.60 (t, 2 H, -CH_2CH_2OH), 2.62–2.30 (m, 14 H, -N(CH_2CH_2)$_2$N-, -NCH_2CH$_2$CH_2COO- and -CH_2CH$_2$OH), 1.83 (m, 2 H, -OCOCH$_2$CH_2CH$_2$-), 1.74 (m, 4 H, PhOCH$_2$CH_2-), 1.43 (m, 4 H, PhOCH$_2$CH$_2$CH_2-), 1.27 (br, 40 H, PhOCH$_2$CH$_2$CH$_2$(CH_2)$_6$CH$_3$ and PhOCH$_2$CH$_2$CH$_2$(CH_2)$_{14}$CH$_3$), 0.86 (t, 6 H, -CH$_2$CH$_2$CH_3). ^{13}C NMR (101 MHz, $CDCl_3$) δ 173.3, 160.5, 138.1, 106.5, 100.9, 68.1, 66.2, 59.4, 57.8, 57.6, 53.0, 52.9, 32.3, 32.0, 32.0, 29.8, 29.7, 29.7, 29.7, 29.6, 29.5, 29.4, 29.4, 29.3, 26.1, 22.8, 22.2, 14.2. Purity by HPLC: 99+%. MALDI-TOF MS m/z of $[M + H]^+$ calculated for $C_{45}H_{83}N_2O_5$: 731.6; Found: 731.6.

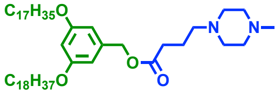

38g (IAJD242)

3-(Heptadecyloxy)-5-(octadecyloxy)benzyl 4-(4-methylpiperazin-1-yl)butanoate (38g, IAJD242). Compound **38g** was synthesized from compound **37d** (0.18 g, 0.23 mmol), 1-methylpiperazine (25 mg, 0.25 mmol) and K_2CO_3 (39 mg, 0.28 mmol) following a procedure similar to that used for the synthesis of compound **38a**. The title compound was obtained as a colorless oil (0.18 g, 99%). ^1H NMR (400 MHz, $CDCl_3$) δ 6.44 (br, 2 H, Ph*H*), 6.37 (t, 1 H, Ph*H*), 5.01 (s, 2 H, PhCH_2-), 3.90 (t, 4 H, PhOCH_2-), 2.59–2.25 (m, 15 H, -N(CH_2CH_2)$_2$N-, -NCH_2CH$_2$CH_2COO- and -NCH_3), 1.83 (m, 2 H, -OCOCH$_2$CH_2CH$_2$-), 1.77 (m, 4 H, PhOCH$_2$CH_2-), 1.42 (m, 4 H, PhOCH$_2$CH$_2$CH_2-), 1.27 (br, 54 H, PhOCH$_2$CH$_2$CH$_2$(CH_2)$_{13}$CH$_3$ and PhOCH$_2$CH$_2$CH$_2$(CH_2)$_{14}$CH$_3$), 0.86 (t, 6 H, -CH$_2$CH$_2$CH_3). ^{13}C NMR (101 MHz, $CDCl_3$) δ 173.3, 160.5, 138.2, 106.5, 101.0, 68.2, 66.2, 57.6, 55.1, 52.9, 45.9, 32.3, 32.0, 29.8, 29.8, 29.7, 29.7, 29.5, 29.5, 29.4, 26.2, 22.8, 22.2, 14.2. Purity by HPLC: 99+%. MALDI-TOF MS m/z of $[M + H]^+$ calculated for $C_{51}H_{95}N_2O_4$: 799.7; Found: 799.8.

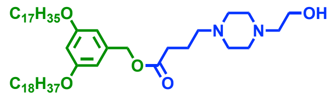

38h (IAJD243)

3-(Heptadecyloxy)-5-(octadecyloxy)benzyl 4-(4-(2-hydroxyethyl)piperazin-1-yl)butanoate (38h, IAJD243). Compound **38h** was synthesized from compound **37d** (0.18 g, 0.23 mmol), 1-(2-hydroxyethyl)piperazine (33 mg, 0.25 mmol) and K_2CO_3 (39 mg, 0.28 mmol) following a procedure similar to that used for the synthesis of compound **38a**. The title compound was obtained as a colorless oil (0.18 g, 95%). ^{1}H NMR (400 MHz, $CDCl_3$) δ 6.45 (br, 2 H, Ph*H*), 6.38 (t, 1 H, Ph*H*), 5.02 (s, 2 H, PhC*H*$_2$-), 3.91 (t, 4 H, PhOC*H*$_2$-), 3.60 (t, 2 H, -CH$_2$C*H*$_2$OH), 2.63–2.31 (m, 14 H, -N(C*H*$_2$C*H*$_2$)$_2$N-, -NC*H*$_2$CH$_2$C*H*$_2$COO- and -C*H*$_2$CH$_2$OH), 1.84 (m, 2 H, -OCOCH$_2$C*H*$_2$CH$_2$-), 1.74 (m, 4 H, PhOCH$_2$C*H*$_2$-), 1.43 (m, 4 H, PhOCH$_2$CH$_2$C*H*$_2$-), 1.25 (br, 54 H, PhOCH$_2$CH$_2$CH$_2$(C*H*$_2$)$_{13}$CH$_3$ and PhOCH$_2$CH$_2$CH$_2$(C*H*$_2$)$_{14}$CH$_3$), 0.87 (t, 6 H, -CH$_2$CH$_2$C*H*$_3$). ^{13}C NMR (101 MHz, $CDCl_3$) δ 173.4, 160.6, 138.2, 106.5, 101.0, 68.2, 66.3, 59.5, 57.8, 57.6, 53.0, 53.0, 32.3, 32.0, 29.8, 29.8, 29.7, 29.7, 29.5, 29.5, 29.4, 26.2, 22.8, 22.2, 14.2. Purity by HPLC: 99+%. MALDI-TOF MS m/z of $[M + H]^+$ calculated for $C_{52}H_{97}N_2O_5$: 829.7; Found: 829.9.

3.7 Synthesis of Undecane and Pentadecane Based IAJDs with 3,5-Nonsymmetric Alkyl Chains

Scheme S7. Synthesis of Undecane and Pentadecane Based IAJDs with 3,5-Nonsymmetric Alkyl Chains

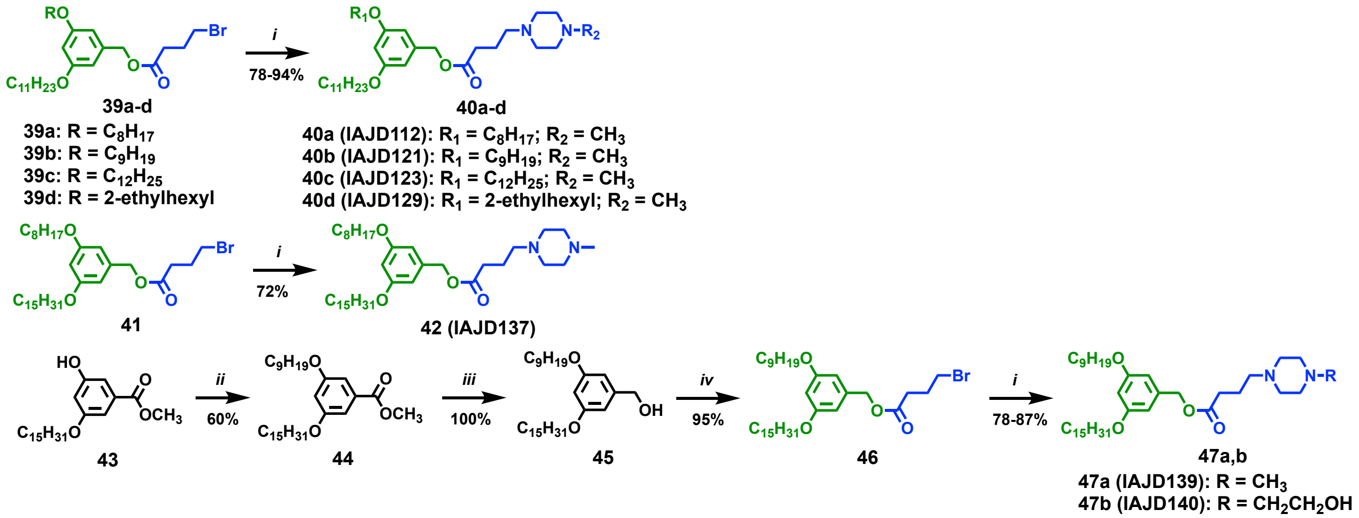

Reagents and conditions: (*i*) K_2CO_3, MeCN, 95 °C, 3 h; (*ii*) $C_9H_{19}Br$, K_2CO_3, DMF, 120 °C, 2 h; (*iii*) $LiAlH_4$, THF, 0–23 °C, 1 h; (*iv*) DCC, DPTS, DCM, 12 h.

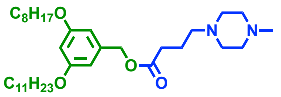

40a (IAJD112)

3-(Octyloxy)-5-(undecyloxy)benzyl 4-(4-methylpiperazin-1-yl)butanoate (40a, IAJD112). Compound **39a** was synthesized according to a literature procedure reported by our laboratory.[5] A mixture of compound **39a** (0.20 g, 0.36 mmol, 1.0 equiv), 1-methylpiperazine (40 mg, 0.40 mmol, 1.1 equiv), K_2CO_3 (59 mg, 0.43 mmol, 1.2 equiv) and MeCN (10 mL) was heated to 95 °C and stirred for 3 h. The reaction mixture was cooled to 23 °C and MeCN was removed under reduced pressure. Then water (20 mL) was added, and the resulted mixture was extracted with DCM (20 mL) for three times. The organic phases were collected and dried over anhydrous $MgSO_4$. After filtration, the obtained filtrate was concentrated to around 3 mL and purified by column chromatography (SiO_2) with DCM/MeOH = 30/1 and 15/1 as the eluent. Then the obtained product was dissolved in DCM (20 mL), which was washed by $NaHCO_3$ solution (2%, 20 mL). The aqueous phase was extracted by DCM (20 mL) for another two times. The organic phases were combined and dried over anhydrous $MgSO_4$. Filtration and evaporation of the solvent yielded the title compound as a colorless oil (0.18 g, 83%). ^{1}H NMR (400 MHz, $CDCl_3$) δ 6.45 (br, 2 H, Ph*H*), 6.38 (t, 1 H, Ph*H*), 5.01 (s, 2 H, PhCH_2-), 3.90 (t, 4 H, PhOCH_2-), 2.64–2.17 (m, 15 H, -N(CH_2CH_2)$_2$N-, -NCH_2CH$_2$CH_2COO- and -NCH_3), 1.83 (m, 2 H, -OCOCH$_2$CH_2CH$_2$-), 1.75 (m, 4 H, PhOCH$_2$CH_2-), 1.44 (m, 4 H, PhOCH$_2$CH$_2$CH_2-), 1.27 (br, 22 H, PhOCH$_2$CH$_2$CH$_2$(CH_2)$_4$CH$_3$ and PhOCH$_2$CH$_2$CH$_2$(CH_2)$_7$CH$_3$), 0.87 (t, 6 H, -CH$_2$CH$_2$CH_3). ^{13}C NMR (101 MHz, $CDCl_3$) δ 173.3, 160.4, 138.1, 106.3, 100.8, 68.0, 66.1, 57.6, 55.2, 53.1, 46.0, 32.2, 31.9, 31.8, 29.6, 29.6, 29.4, 29.3, 29.3, 29.2, 26.0, 22.7, 22.6, 22.2, 14.1, 14.1. Purity by HPLC: 99+%. MALDI-TOF MS m/z of $[M + H]^+$ calculated for $C_{35}H_{63}N_2O_4$: 575.5; Found: 576.9.

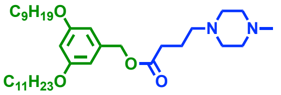

40b (IAJD121)

3-(Nonyloxy)-5-(undecyloxy)benzyl 4-(4-methylpiperazin-1-yl)butanoate (40b, IAJD121). Compound **39b** was synthesized according to a literature procedure reported by our laboratory.[5] Compound **40b** was synthesized from compound **39b** (0.15 g, 0.26 mmol), 1-methylpiperazine (29 mg, 0.29 mmol) and K_2CO_3 (43 mg, 0.31 mmol) following a procedure similar to that used for the synthesis of compound **40a**. The title compound was obtained as a colorless oil (0.14 g, 93%). ^{1}H NMR (400 MHz, $CDCl_3$) δ 6.45 (br, 2 H, Ph*H*), 6.38 (t, 1 H, Ph*H*), 5.01 (s, 2 H, PhCH_2-), 3.90 (t, 4 H, PhOCH_2-), 2.68–2.18 (m, 15 H, -N(CH_2CH_2)$_2$N-, -NCH_2CH$_2$CH_2COO- and -NCH_3), 1.83 (m, 2 H, -OCOCH$_2$CH_2CH$_2$-),

1.74 (m, 4 H, PhOCH$_2$CH_2-), 1.43 (m, 4 H, PhOCH$_2$CH$_2$CH_2-), 1.29 (br, 24 H, PhOCH$_2$CH$_2$CH$_2$(CH_2)$_5$CH$_3$ and PhOCH$_2$CH$_2$CH$_2$(CH_2)$_7$CH$_3$), 0.87 (t, 6 H, -CH$_2$CH$_2$CH_3). ^{13}C NMR (101 MHz, CDCl$_3$) δ 173.3, 160.4, 138.1, 106.3, 100.8, 68.0, 66.1, 57.5, 55.2, 53.1, 46.0, 32.2, 31.9, 31.9, 29.6, 29.6, 29.5, 29.4, 29.3, 29.2, 26.0, 22.7, 22.7, 22.2, 14.1. Purity by HPLC: 99+%. MALDI-TOF MS m/z of [M + H]$^+$ calculated for $C_{36}H_{65}N_2O_4$: 589.5; Found: 591.2.

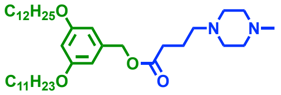

40c (IAJD123)

3-(Dodecyloxy)-5-(undecyloxy)benzyl 4-(4-methylpiperazin-1-yl)butanoate (40c, IAJD123). Compound **39c** was synthesized according to a literature procedure reported by our laboratory.[5] Compound **40c** was synthesized from compound **39c** (0.25 g, 0.41 mmol), 1-methylpiperazine (45 mg, 0.45 mmol) and K_2CO_3 (68 mg, 0.49 mmol) following a procedure similar to that used for the synthesis of compound **40a**. The title compound was obtained as a colorless oil (0.20 g, 78%). ^{1}H NMR (400 MHz, CDCl$_3$) δ 6.45 (br, 2 H, PhH), 6.38 (t, 1 H, PhH), 5.02 (s, 2 H, PhCH_2-), 3.91 (t, 4 H, PhOCH_2-), 2.63–2.20 (m, 15 H, -N(CH_2CH_2)$_2$N-, -NCH_2CH$_2$CH_2COO- and -NCH_3), 1.83 (m, 2 H, -OCOCH$_2$CH_2CH$_2$-), 1.76 (m, 4 H, PhOCH$_2$CH_2-), 1.42 (m, 4 H, PhOCH$_2$CH$_2$CH_2-), 1.28 (br, 30 H, PhOCH$_2$CH$_2$CH$_2$(CH_2)$_8$CH$_3$ and PhOCH$_2$CH$_2$CH$_2$(CH_2)$_7$CH$_3$), 0.87 (t, 6 H, -CH$_2$CH$_2$CH_3). ^{13}C NMR (101 MHz, CDCl$_3$) δ 173.5, 160.6, 138.2, 106.5, 101.0, 77.4, 68.2, 66.3, 60.5, 57.7, 55.3, 53.2, 46.2, 32.4, 32.0, 29.8, 29.8, 29.7, 29.7, 29.5, 29.5, 29.4, 26.2, 22.8, 22.3, 21.2, 14.3, 14.2. Purity by HPLC: 99+%. MALDI-TOF MS m/z of [M + H]$^+$ calculated for $C_{39}H_{71}N_2O_4$: 631.5; Found: 632.8.

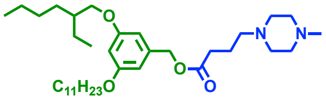

40d (IAJD129)

3-((2-Ethylhexyl)oxy)-5-(undecyloxy)benzyl 4-(4-methylpiperazin-1-yl)butanoate (40d, IAJD129). Compound **39d** was synthesized according to a literature procedure reported by our laboratory.[5] Compound **40d** was synthesized from compound **39d** (0.28 g, 0.49 mmol), 1-methylpiperazine (55 mg, 0.54 mmol) and K_2CO_3 (81 mg, 0.59 mmol) following a procedure similar to that used for the synthesis of compound **40a**. The title compound was obtained as a colorless oil (0.26 g, 94%). ^{1}H NMR (400 MHz, CDCl$_3$) δ 6.46 (br, 2 H, PhH), 6.39 (t, 1 H, PhH), 5.02 (s, 2 H, PhCH_2-), 3.92 (t, 4 H, PhOCH_2-), 3.80 (m, 2 H, PhOCH_2CH(CH$_2$CH$_3$)-), 2.62–2.18 (m, 15 H, -N(CH_2CH_2)$_2$N-, -NCH_2CH$_2$CH_2COO- and -NCH_3), 1.84 (m, 2 H, -OCOCH$_2$CH_2CH$_2$-), 1.71 (m, 3 H, PhOCH$_2$CH_2(CH$_2$)$_8$CH$_3$ and PhOCH$_2$CH(CH$_2$CH$_3$)(CH$_2$)$_3$CH$_3$), 1.52–1.19 (m, 24 H, PhOCH$_2$CH$_2$(CH_2)$_8$CH$_3$ and

PhOCH$_2$CH(CH_2CH$_3$)(CH_2)$_3$CH$_3$), 0.89 (m, 9 H, PhO(CH$_2$)$_{10}$CH_3 and PhOCH$_2$CH(CH$_2$CH_3)(CH$_2$)$_3$CH_3). ^{13}C NMR (101 MHz, CDCl$_3$) δ 173.5, 160.8, 160.6, 138.1, 106.6, 106.4, 101.0, 77.4, 70.6, 68.2, 66.3, 57.7, 55.3, 53.2, 46.2, 39.5, 32.4, 32.0, 30.6, 29.7, 29.7, 29.5, 29.5, 29.4, 29.2, 26.2, 24.0, 23.2, 22.8, 22.3, 14.2, 14.2, 11.2. Purity by HPLC: 99+%. MALDI-TOF MS m/z of $[M + H]^+$ calculated for $C_{35}H_{63}N_2O_4$: 575.5; Found: 576.6.

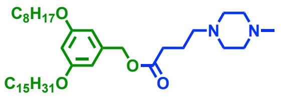

42 (IAJD137)

3-(Octyloxy)-5-(pentadecyloxy)benzyl 4-(4-methylpiperazin-1-yl)butanoate (42, IAJD137). Compound **41** was synthesized according to a literature procedure reported by our laboratory.[5] Compound **42** was synthesized from compound **41** (0.25 g, 0.41 mmol), 1-methylpiperazine (45 mg, 0.45 mmol) and K_2CO_3 (68 mg, 0.49 mmol) following a procedure similar to that used for the synthesis of compound **40a**. The title compound was obtained as a colorless oil (0.18 g, 72%). ^{1}H NMR (400 MHz, CDCl$_3$) δ 6.46 (br, 2 H, PhH), 6.39 (t, 1 H, PhH), 5.02 (s, 2 H, PhCH_2-), 3.91 (t, 4 H, PhOCH_2-), 2.62–2.24 (m, 15 H, -N(CH_2CH_2)$_2$N-, -NCH_2CH$_2$CH_2COO- and -NCH_3), 1.83 (m, 2 H, -OCOCH$_2$CH_2CH$_2$-), 1.75 (m, 4 H, PhOCH$_2$CH_2-), 1.43 (m, 4 H, PhOCH$_2$CH$_2$CH_2-), 1.27 (br, 30 H, PhOCH$_2$CH$_2$CH$_2$(CH_2)$_4$CH$_3$ and PhOCH$_2$CH$_2$CH$_2$(CH_2)$_{11}$CH$_3$), 0.88 (t, 6 H, -CH$_2$CH$_2$CH_3). ^{13}C NMR (101 MHz, CDCl$_3$) δ 173.3, 160.5, 138.1, 106.4, 100.9, 68.1, 66.2, 57.5, 55.0, 52.8, 45.8, 32.2, 31.9, 31.8, 29.7, 29.7, 29.6, 29.6, 29.4, 29.4, 29.3, 26.1, 22.7, 22.7, 22.1, 14.1. Purity by HPLC: 99+%. MALDI-TOF MS m/z of $[M + H]^+$ calculated for $C_{39}H_{71}N_2O_4$: 631.5; Found: 632.8.

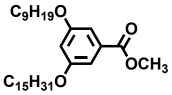

44

Methyl 3-(nonyloxy)-5-(pentadecyloxy)benzoate (44). Compound **43** was synthesized according to a literature procedure reported by our laboratory.[5] A mixture of compound **43** (0.50 g, 1.32 mmol, 1 equiv), 1-bromononane (0.30 g, 1.45 mmol, 1.1 equiv), K_2CO_3 (0.36 g, 2.64 mmol, 2 equiv) and DMF (15 mL) was heated to 120 °C and stirred under N_2 atmosphere for 2 h. The reaction mixture was cooled to 23 °C and poured into ice/water (150 mL). The resulted white precipitates in the solution were filtered and collected. Then the precipitates were recrystallized from minimum acetone to afford the title compound as a white solid (0.39 g, 60%). ^{1}H NMR (400 MHz, CDCl$_3$) δ 7.16 (br, 2 H, PhH), 6.63 (t, 1 H, PhH), 3.96 (t, 4 H, PhOCH_2CH$_2$CH$_2$-), 3.89 (s, 3 H, PhCOOCH_3), 1.77 (m, 4 H, PhOCH$_2$CH_2CH$_2$-), 1.45 (m, 4 H, PhOCH$_2$CH$_2$CH_2-), 1.26 (m, 32 H, PhOCH$_2$CH$_2$CH$_2$(CH_2)$_5$CH$_3$ and PhOCH$_2$CH$_2$CH$_2$(CH_2)$_{11}$CH$_3$), 0.88

(t, 6 H, PhO$(CH_2)_8CH_3$ and PhO$(CH_2)_{14}CH_3$). ^{13}C NMR (101 MHz, $CDCl_3$) δ 167.0, 160.2, 131.8, 107.6, 106.6, 68.3, 52.2, 31.9, 31.9, 29.7, 29.7, 29.6, 29.6, 29.5, 29.4, 29.3, 29.2, 26.0, 22.7, 22.7, 14.1. Mp = 52°C.

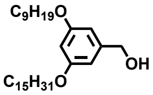

45

(3-(Nonyloxy)-5-(pentadecyloxy)phenyl)methanol (45). Compound **44** (0.35 g, 0.69 mmol, 1 equiv) was dissolved in 5 mL dry THF, which was added dropwise to a slurry of $LiAlH_4$ (26 mg, 0.69 mmol, 1 equiv) in dry THF (5 mL) at 0 °C under N_2 atmosphere. Afterwards, the resulted mixture was stirred at 23 °C for 1 h. Finally, the reaction was quenched by the successive addition of water (0.2 mL), 15% NaOH aqueous solution (0.2 mL) and water (1.0 mL). White precipitates were formed in the mixture and filtered out. The filtrate was dried over anhydrous $MgSO_4$ and filtered. The solvent was removed under reduced pressure to afford the title compound as a colorless oil (0.25 g, 100%). ^{1}H NMR (400 MHz, $CDCl_3$) δ 6.50 (br, 2 H, PhH), 6.37 (t, 1 H, PhH), 4.61 (s, 2 H, PhCH_2OH), 3.93 (t, 4 H, PhO$CH_2CH_2CH_2$-), 1.76 (m, 4 H, PhO$CH_2CH_2CH_2$-), 1.43 (m, 4 H, PhO$CH_2CH_2CH_2$-), 1.29 (m, 32 H, PhO$CH_2CH_2CH_2(CH_2)_5CH_3$ and PhO$CH_2CH_2CH_2(CH_2)_{11}CH_3$), 0.88 (t, 6 H, PhO$(CH_2)_8CH_3$ and PhO$(CH_2)_{14}CH_3$). ^{13}C NMR (101 MHz, $CDCl_3$) δ 160.5, 143.2, 105.1, 100.6, 68.1, 65.5, 31.9, 31.8, 29.7, 29.7, 29.6, 29.6, 29.4, 29.4, 29.3, 26.1, 22.7, 22.7, 14.1, 14.1.

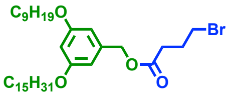

46

3-(Nonyloxy)-5-(pentadecyloxy)benzyl 4-bromobutanoate (46). Compound **45** (0.29 g, 0.61 mmol, 1 equiv), 4-bromobutyric acid (0.11 g, 0.67 mmol, 1.1 equiv) and DPTS (0.19 g, 0.67 mmol, 1.1 equiv) were dissolved in 8 mL DCM. DCC (0.26 g, 1.22 mmol, 2 equiv) was added into the above mixture. The reaction mixture was stirred at 23 °C for 12 h. Afterwards, the resulted precipitates (urea) were filtered out from the reaction mixture. The filtrate was concentrated to around 3 mL and purified by column chromatography (SiO_2) with hexane/DCM = 1/1 as the mobile phase to give the title compound as a colorless oil (0.32 g, 95%). ^{1}H NMR (400 MHz, $CDCl_3$) δ 6.46 (br, 2 H, PhH), 6.41 (t, 1 H, PhH), 5.05 (s, 2 H, PhCH_2OCO), 3.93 (t, 4 H, PhO$CH_2CH_2CH_2$-), 3.47 (t, 2 H, -OCO$CH_2CH_2CH_2$Br), 2.57 (t, 2 H, -OCO$CH_2CH_2CH_2$Br), 2.20 (m, 2 H, -OCO$CH_2CH_2CH_2$Br), 1.76 (m, 4 H, PhO$CH_2CH_2CH_2$-), 1.44 (m, 4 H, PhO$CH_2CH_2CH_2$-), 1.27 (m, 32 H, PhO$CH_2CH_2CH_2(CH_2)_5CH_3$ and PhO$CH_2CH_2CH_2(CH_2)_{11}CH_3$), 0.89 (t, 6 H, PhO$(CH_2)_8CH_3$ and PhO$(CH_2)_{14}CH_3$). ^{13}C NMR (101 MHz, $CDCl_3$) δ 172.3, 160.5, 137.8,

106.4, 101.0, 77.2, 68.1, 66.4, 32.7, 32.5, 31.9, 31.9, 29.7, 29.7, 29.6, 29.6, 29.6, 29.5, 29.4, 29.4, 29.3, 29.3, 27.7, 26.1, 22.7, 14.1.

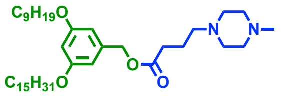

47a (IAJD139)

3-(Nonyloxy)-5-(pentadecyloxy)benzyl 4-(4-methylpiperazin-1-yl)butanoate (47a, IAJD139). Compound **47a** was synthesized from compound **46** (0.20 g, 0.31 mmol), 1-methylpiperazine (34 mg, 0.34 mmol) and K_2CO_3 (51 mg, 0.37 mmol) following a procedure similar to that used for the synthesis of compound **40a**. The title compound was obtained as a colorless oil (0.15 g, 78%). ^{1}H NMR (400 MHz, $CDCl_3$) δ 6.45 (br, 2 H, Ph*H*), 6.38 (t, 1 H, Ph*H*), 5.02 (s, 2 H, PhCH_2-), 3.91 (t, 4 H, PhOCH_2-), 2.62–2.26 (m, 15 H, -N(CH_2CH_2)$_2$N-, -NCH_2CH$_2$CH_2COO- and -NCH_3), 1.84 (m, 2 H, -OCOCH$_2$CH_2CH$_2$-), 1.74 (m, 4 H, PhOCH$_2$CH_2-), 1.43 (m, 4 H, PhOCH$_2$CH$_2$CH_2-), 1.27 (br, 32 H, PhOCH$_2$CH$_2$CH$_2$(CH_2)$_5$CH$_3$ and PhOCH$_2$CH$_2$CH$_2$(CH_2)$_{11}$CH$_3$), 0.87 (t, 6 H, -CH$_2$CH$_2$CH_3). ^{13}C NMR (101 MHz, $CDCl_3$) δ 173.3, 160.5, 138.1, 106.4, 100.9, 77.2, 68.1, 66.2, 57.5, 55.0, 52.8, 45.8, 32.2, 31.9, 31.9, 29.7, 29.7, 29.6, 29.6, 29.5, 29.4, 29.4, 29.3, 26.1, 22.7, 22.7, 22.1, 14.1. Purity by HPLC: 99+%. MALDI-TOF MS m/z of $[M + H]^+$ calculated for $C_{40}H_{73}N_2O_4$: 645.6; Found: 647.7.

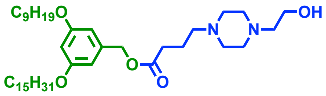

47b (IAJD140)

3-(Nonyloxy)-5-(pentadecyloxy)benzyl 4-(4-(2-hydroxyethyl)piperazin-1-yl)butanoate (47b, IAJD140). Compound **47b** was synthesized from compound **46** (0.20 g, 0.31 mmol), 1-(2-hydroxyethyl)piperazine (44 mg, 0.34 mmol) and K_2CO_3 (51 mg, 0.37 mmol) following a procedure similar to that used for the synthesis of compound **40a**. The title compound was obtained as a colorless oil (0.20 g, 87%). ^{1}H NMR (400 MHz, $CDCl_3$) δ 6.46 (br, 2 H, Ph*H*), 6.39 (t, 1 H, Ph*H*), 5.02 (s, 2 H, PhCH_2-), 3.91 (t, 4 H, PhOCH_2-), 3.61 (t, 2 H, -CH$_2$CH_2OH), 2.63–2.27 (m, 14 H, -N(CH_2CH_2)$_2$N-, -NCH_2CH$_2$CH_2COO- and -CH_2CH$_2$OH), 1.83 (m, 2 H, -OCOCH$_2$CH_2CH$_2$-), 1.74 (m, 4 H, PhOCH$_2$CH_2-), 1.43 (m, 4 H, PhOCH$_2$CH$_2$CH_2-), 1.25 (br, 32 H, PhOCH$_2$CH$_2$CH$_2$(CH_2)$_5$CH$_3$ and PhOCH$_2$CH$_2$CH$_2$(CH_2)$_{11}$CH$_3$), 0.88 (t, 6 H, -CH$_2$CH$_2$CH_3). ^{13}C NMR (101 MHz, $CDCl_3$) δ 173.3, 160.5, 138.1, 106.4, 100.9, 68.1, 66.2, 59.3, 59.3, 57.6, 57.5, 53.4, 53.0, 52.8, 32.2, 31.9, 31.9, 29.7, 29.7, 29.6, 29.6, 29.5, 29.4, 29.4, 29.3, 26.1, 22.7, 22.7, 22.1, 14.1. Purity by HPLC: 99+%. MALDI-TOF MS m/z of $[M + H]^+$ calculated for $C_{41}H_{75}N_2O_5$: 675.6; Found: 676.9.

3.8 Synthesis of Tridecane Based IAJDs with 3,5-Nonsymmetric Alkyl Chains

Scheme S8. Synthesis of Tridecane Based IAJDs with 3,5-Nonsymmetric Alkyl Chains

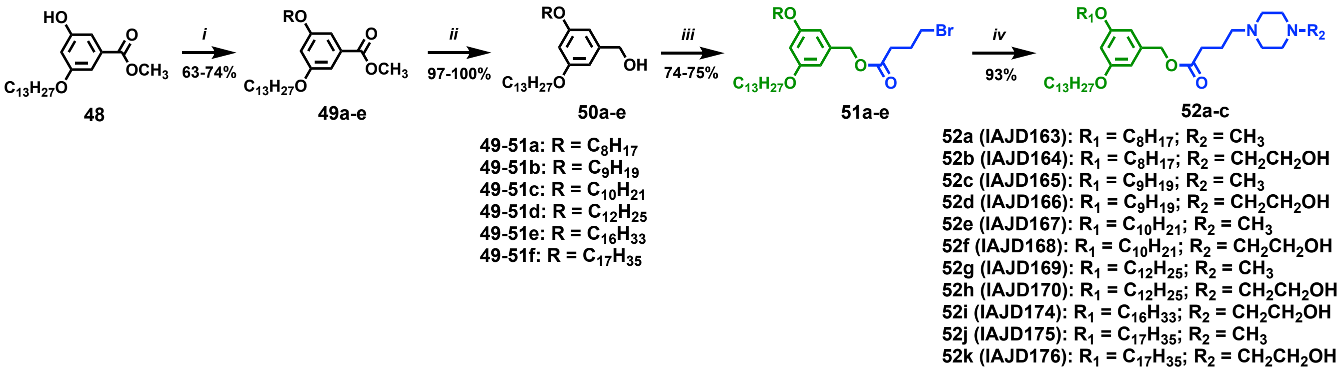

Reagents and conditions: (*i*) RBr, K_2CO_3, DMF, 120 °C, 2 h; (*ii*) $LiAlH_4$, THF, 0–23 °C, 1 h; (*iii*) DCC, DPTS, DCM, 12 h; (*iv*) K_2CO_3, MeCN, 95 °C, 3 h.

General Synthetic Procedure for Compounds 51e. Compounds **49e** were synthesized according to a procedure elaborated and optimized by our laboratory.[3] Generally, compound **48** (1 equiv) and K_2CO_3 (2 equiv) were stirred in dry DMF. RBr (2.2 equiv) was added, and the mixture was stirred at 120 °C under N_2 atmosphere for 2 h. The reaction mixture was cooled to 23 °C. The reaction mixture was poured into ice/water and the white precipitates were filtered and collected. Then the precipitates were purified by recrystallization from acetone to give the title compound as a white solid. The synthesis and characterizations of compounds **50e** were reported in the literature.[5]

General Synthetic Procedure for Compounds 50e. Compounds **50e** were synthesized according to a procedure reported by our group.[4] Generally, compounds **49e** (1 equiv) were dissolved in dry THF, which was added dropwise to a slurry of $LiAlH_4$ (1 equiv) in dry THF at 0 °C under N_2 atmosphere. The resulted mixture was stirred at 23 °C for 1 h. The reaction was quenched by the successive addition of water, 15% NaOH aqueous solution and water. Then the mixture was filtered and dried over anhydrous $MgSO_4$. Filtration and evaporation of the solvent yielded the title compound as a white solid or light-yellow oil. The synthesis and characterizations of compounds **50e** were reported in the literature.[5]

General Synthetic Procedure for Compounds 51e. Compounds **51e** were synthesized according to a procedure reported by our group.[4] Generally, compounds **50e** (1 equiv), 4-bromobutyric acid (1.1 equiv) and DPTS (1.1 equiv) were dissolved in dry DCM. DCC (2 equiv) was added into the above mixture and stirred for 12h. Hexane was added to the reaction mixture, which was then vaccum filtered. The filtrate was concentrated and further purified by column chromatography on silica gel with a mobile phase of

hexane/DCM = 1/1 (v/v) to afford the title compound as a transparent oil. The synthesis and characterizations of compounds **51e** were reported in the literature.[5]

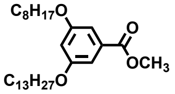

49a

Methyl 3-(octyloxy)-5-(tridecyloxy)benzoate (49a). Compound **48** was synthesized according to a literature procedure reported by our laboratory.[5] A mixture of compound **48** (0.60 g, 1.71 mmol, 1 equiv), 1-bromooctane (0.37 g, 1.88 mmol, 1.1 equiv), K_2CO_3 (0.47 g, 3.42 mmol, 2 equiv) and DMF (15 mL) was heated to 120 °C and stirred under N_2 atmosphere for 2 h. The reaction mixture was cooled to 23 °C and poured into ice/water (150 mL). The resulted white precipitates in the solution were filtered and collected. Then the precipitates were recrystallized from minimum acetone to afford the title compound as a white solid (0.51 g, 64%). ^{1}H NMR (400 MHz, $CDCl_3$) δ 7.16 (br, 2 H, Ph*H*), 6.63 (t, 1 H, Ph*H*), 3.96 (t, 4 H, PhOCH_2CH$_2$CH$_2$-), 3.90 (s, 3 H, PhCOOCH_3), 1.77 (m, 4 H, PhOCH$_2$CH_2CH$_2$-), 1.45 (m, 4 H, PhOCH$_2$CH$_2$CH_2-), 1.28 (m, 26 H, PhOCH$_2$CH$_2$CH$_2$(CH_2)$_4$CH$_3$ and PhOCH$_2$CH$_2$CH$_2$(CH_2)$_9$CH$_3$), 0.88 (t, 6 H, PhO(CH$_2$)$_7$CH_3 and PhO(CH$_2$)$_{12}$CH_3). ^{13}C NMR (101 MHz, $CDCl_3$) δ 167.2, 160.3, 132.0, 107.8, 106.7, 68.5, 52.3, 32.1, 29.8, 29.8, 29.7, 29.5, 29.3, 26.2, 22.8, 14.3. Mp = 47 °C.

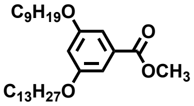

49b

Methyl 3-(nonyloxy)-5-(tridecyloxy)benzoate (**49b**). Compound **49b** was synthesized from Compound 27 (0.60 g, 1.71 mmol, 1 equiv), 1-bromononane (0.39 g, 1.88 mmol, 1.1 equiv) and K_2CO_3 (0.47 g, 3.42 mmol, 2 equiv) following a procedure similar to that used for the synthesis of compound **49a**. The title compound was obtained as a white solid (0.51 g, 63%). ^{1}H NMR (400 MHz, $CDCl_3$) δ 7.16 (br, 2 H, Ph*H*), 6.63 (t, 1 H, Ph*H*), 3.96 (t, 4 H, PhOCH_2CH$_2$CH$_2$-), 3.89 (s, 3 H, PhCOOCH_3), 1.77 (m, 4 H, PhOCH$_2$CH_2CH$_2$-), 1.45 (m, 4 H, PhOCH$_2$CH$_2$CH_2-), 1.29 (m, 28 H, PhOCH$_2$CH$_2$CH$_2$(CH_2)$_5$CH$_3$ and PhOCH$_2$CH$_2$CH$_2$(CH_2)$_9$CH$_3$), 0.88 (t, 6 H, PhO(CH$_2$)$_8$CH_3 and PhO(CH$_2$)$_{12}$CH_3). ^{13}C NMR (101 MHz, $CDCl_3$) δ 167.1, 160.3, 132.0, 107.8, 106.7, 77.4, 68.5, 52.3, 32.1, 32.0, 29.8, 29.8, 29.7, 29.7, 29.5, 29.3, 26.2, 22.8, 14.2. Mp = 48 °C.

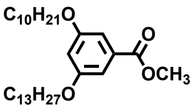

49c

Methyl 3-(decyloxy)-5-(tridecyloxy)benzoate (49c). Compound **49c** was synthesized from compound **48** (0.60 g, 1.71 mmol, 1 equiv), 1-bromodecane (0.39 g, 1.88 mmol, 1.1 equiv) and K_2CO_3 (0.47 g, 3.42 mmol, 2 equiv) following a procedure similar to that used for the synthesis of compound **49a**. The title compound was obtained as a white solid (0.62 g, 74%). ^{1}H NMR (400 MHz, $CDCl_3$) δ 7.16 (br, 2 H, Ph*H*), 6.63 (t, 1 H, Ph*H*), 3.96 (t, 4 H, PhOCH_2CH$_2$CH$_2$-), 3.89 (s, 3 H, PhCOOCH_3), 1.77 (m, 4 H, PhOCH$_2$CH_2CH$_2$-), 1.44 (m, 4 H, PhOCH$_2$CH$_2$CH_2-), 1.28 (m, 30 H, PhOCH$_2$CH$_2$CH$_2$(CH_2)$_6$CH$_3$ and PhOCH$_2$CH$_2$CH$_2$(CH_2)$_9$CH$_3$), 0.88 (t, 6 H, PhO(CH$_2$)$_9$CH_3 and PhO(CH$_2$)$_{12}$CH_3). ^{13}C NMR (101 MHz, $CDCl_3$) δ 167.1, 160.3, 132.0, 107.8, 106.7, 77.4, 68.5, 52.3, 32.1, 32.0, 29.8, 29.8, 29.7, 29.7, 29.7, 29.5, 29.5, 29.3, 26.2, 22.8, 14.3. Mp = 50 °C.

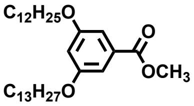

49d

Methyl 3-(Dodecyloxy)-5-(tridecyloxy)benzoate (**49d**). Compound **49d** was synthesized from Compound **48** (0.60 g, 1.71 mmol, 1 equiv), 1-bromononane (0.39 g, 1.88 mmol, 1.1 equiv) and K_2CO_3 (0.47 g, 3.42 mmol, 2 equiv) following a procedure similar to that used for the synthesis of compound **49a**. The title compound was obtained as a white solid (0.71 g, 80%). ^{1}H NMR (400 MHz, $CDCl_3$) δ 7.16 (br, 2 H, Ph*H*), 6.64 (t, 1 H, Ph*H*), 3.96 (t, 4 H, PhOCH_2CH$_2$CH$_2$-), 3.89 (s, 3 H, PhCOOCH_3), 1.77 (m, 4 H, PhOCH$_2$CH_2CH$_2$-), 1.45 (m, 4 H, PhOCH$_2$CH$_2$CH_2-), 1.26 (m, 34 H, PhOCH$_2$CH$_2$CH$_2$(CH_2)$_8$CH$_3$ and PhOCH$_2$CH$_2$CH$_2$(CH_2)$_9$CH$_3$), 0.88 (t, 6 H, PhO(CH$_2$)$_{11}$CH_3 and PhO(CH$_2$)$_{12}$CH_3). ^{13}C NMR (101 MHz, $CDCl_3$) δ 167.1, 160.3, 132.0, 107.8, 106.7, 77.4, 68.5, 52.3, 32.1, 29.8, 29.7, 29.7, 29.5, 29.3, 26.2, 22.8, 14.3. Mp = 51 °C.

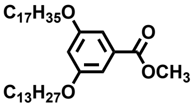

49f

Methyl 3-(Heptadecyloxy)-5-(tridecyloxy)benzoate (**49f**). Compound **49f** was synthesized from Compound **48** (0.60 g, 1.71 mmol, 1 equiv), 1-bromononane (0.39 g, 1.88 mmol, 1.1 equiv) and K_2CO_3 (0.47 g, 3.42 mmol, 2 equiv) following a procedure similar to that used for the synthesis of compound **49a**. The title compound was obtained as a white solid (0.75 g, 75%). ^{1}H NMR (400 MHz, $CDCl_3$) δ 7.16

(br, 2 H, Ph*H*), 6.63 (t, 1 H, Ph*H*), 3.96 (t, 4 H, $PhOCH_2CH_2CH_2$-), 3.89 (s, 3 H, $PhCOOCH_3$), 1.77 (m, 4 H, $PhOCH_2CH_2CH_2$-), 1.45 (m, 4 H, $PhOCH_2CH_2CH_2$-), 1.28 (m, 44 H, $PhOCH_2CH_2CH_2(CH_2)_{13}CH_3$ and $PhOCH_2CH_2CH_2(CH_2)_9CH_3$), 0.89 (t, 6 H, $PhO(CH_2)_{16}CH_3$ and $PhO(CH_2)_{12}CH_3$). ^{13}C NMR (101 MHz, $CDCl_3$) δ 167.1, 160.3, 132.0, 107.8, 106.7, 68.5, 52.3, 32.1, 29.8, 29.8, 29.8, 29.7, 29.5, 29.5, 29.3, 26.2, 22.8, 14.3. Mp = 54 °C.

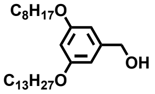

50a

(3-(Octyloxy)-5-(tridecyloxy)phenyl)methanol (50a). Compound **49a** (0.47 g, 1.02 mmol, 1 equiv) was dissolved in 5 mL dry THF, which was added dropwise to a slurry of $LiAlH_4$ (39 mg, 1.02 mmol, 1 equiv) in dry THF (5 mL) at 0 °C under N_2 atmosphere. Afterwards, the resulted mixture was stirred at 23 °C for 1 h. Finally, the reaction was quenched by the successive addition of water (0.2 mL), 15% NaOH aqueous solution (0.2 mL) and water (1.0 mL). White precipitates were formed in the mixture and filtered out. The filtrate was dried over anhydrous $MgSO_4$ and filtered. The solvent was removed under reduced pressure to afford the title compound as a colorless oil (0.45 g, 100%). ^{1}H NMR (400 MHz, $CDCl_3$) δ 6.49 (br, 2 H, Ph*H*), 6.38 (t, 1 H, Ph*H*), 4.61 (s, 2 H, $PhCH_2OH$), 3.93 (t, 4 H, $PhOCH_2CH_2CH_2$-), 1.77 (m, 4 H, $PhOCH_2CH_2CH_2$-), 1.44 (m, 4 H, $PhOCH_2CH_2CH_2$-), 1.28 (m, 26 H, $PhOCH_2CH_2CH_2(CH_2)_4CH_3$ and $PhOCH_2CH_2CH_2(CH_2)_9CH_3$), 0.89 (t, 6 H, $PhO(CH_2)_7CH_3$ and $PhO(CH_2)_{12}CH_3$). ^{13}C NMR (101 MHz, $CDCl_3$) δ 160.7, 143.4, 143.3, 105.2, 100.7, 68.2, 65.5, 32.1, 32.0, 29.8, 29.8, 29.8, 29.7, 29.5, 29.5, 29.4, 29.4, 26.2, 22.8, 22.8, 14.2, 14.2.

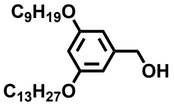

50b

(3-(Nonyloxy)-5-(tridecyloxy)phenyl)methanol (50b). Compound **49b** (0.45 g, 0.94 mmol, 1 equiv) and $LiAlH_4$ (36 mg, 0.94 mmol, 1 equiv) following a procedure similar to that used for the synthesis of compound 23a. The title compound was obtained as a colorless oil (0.41 g, 97%). ^{1}H NMR (400 MHz, $CDCl_3$) δ 6.49 (br, 2 H, Ph*H*), 6.38 (t, 1 H, Ph*H*), 4.61 (s, 2 H, $PhCH_2OH$), 3.93 (t, 4 H, $PhOCH_2CH_2CH_2$-), 1.76 (m, 4 H, $PhOCH_2CH_2CH_2$-), 1.44 (m, 4 H, $PhOCH_2CH_2CH_2$-), 1.29 (m, 28 H, $PhOCH_2CH_2CH_2(CH_2)_5CH_3$ and $PhOCH_2CH_2CH_2(CH_2)_9CH_3$), 0.89 (t, 6 H, $PhO(CH_2)_8CH_3$ and $PhO(CH_2)_{12}CH_3$). ^{13}C NMR (101 MHz, $CDCl_3$) δ 160.7, 160.6, 143.4, 143.4, 105.2, 100.7, 77.5, 77.2, 76.9, 68.2, 65.6, 65.4, 32.1, 32.0, 29.8, 29.8, 29.8, 29.7, 29.7, 29.6, 29.5, 29.5, 29.4, 26.2, 22.8, 22.8, 14.2.

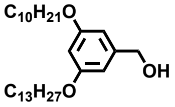

50c

(3-(Decyloxy)-5-(tridecyloxy)phenyl)methanol (50c). Compound **50c** was synthesized from compound **49b** (0.56 g, 1.14 mmol, 1 equiv) and $LiAlH_4$ (44 mg, 1.14 mmol, 1 equiv) following a procedure similar to that used for the synthesis of compound **50a**. The title compound was obtained as a colorless oil (0.51 g, 97%). ^{1}H NMR (400 MHz, $CDCl_3$) δ 6.50 (br, 2 H, Ph*H*), 6.38 (t, 1 H, Ph*H*), 4.61 (s, 2 H, $PhC\mathit{H}_2OH$), 3.93 (t, 4 H, $PhOC\mathit{H}_2CH_2CH_2$-), 1.76 (m, 4 H, $PhOCH_2C\mathit{H}_2CH_2$-), 1.44 (m, 4 H, $PhOCH_2CH_2C\mathit{H}_2$-), 1.29 (m, 30 H, $PhOCH_2CH_2CH_2(C\mathit{H}_2)_6CH_3$ and $PhOCH_2CH_2CH_2(C\mathit{H}_2)_9CH_3$), 0.89 (t, 6 H, $PhO(CH_2)_9C\mathit{H}_3$ and $PhO(CH_2)_{12}C\mathit{H}_3$). ^{13}C NMR (101 MHz, $CDCl_3$) δ 160.7, 160.7, 143.4, 105.2, 100.7, 100.7, 68.2, 65.6, 65.5, 32.1, 32.0, 29.8, 29.8, 29.8, 29.7, 29.5, 29.5, 29.5, 29.4, 26.2, 22.8, 14.3.

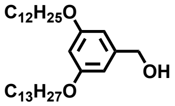

50d

(3-(Dodecyloxy)-5-(tridecyloxy)phenyl)methanol (**50d**). Compound **49d** (0.68 g, 1.31 mmol, 1 equiv) and $LiAlH_4$ (50 mg, 1.31 mmol, 1 equiv) following a procedure similar to that used for the synthesis of compound 23a. The title compound was obtained as a colorless oil (0.60 g, 93%). ^{1}H NMR (400 MHz, $CDCl_3$) δ 6.49 (br, 2 H, Ph*H*), 6.37 (t, 1 H, Ph*H*), 4.60 (s, 2 H, $PhC\mathit{H}_2OH$), 3.93 (t, 4 H, $PhOC\mathit{H}_2CH_2CH_2$-), 1.76 (m, 4 H, $PhOCH_2C\mathit{H}_2CH_2$-), 1.43 (m, 4 H, $PhOCH_2CH_2C\mathit{H}_2$-), 1.29 (m, 34 H, $PhOCH_2CH_2CH_2(C\mathit{H}_2)_8CH_3$ and $PhOCH_2CH_2CH_2(C\mathit{H}_2)_9CH_3$), 0.89 (t, 6 H, $PhO(CH_2)_{11}C\mathit{H}_3$ and $PhO(CH_2)_{12}C\mathit{H}_3$). ^{13}C NMR (101 MHz, $CDCl_3$) δ 160.7, 160.7, 143.4, 143.3, 105.2, 100.7, 100.7, 68.2, 65.6, 65.5, 32.1, 29.8, 29.8, 29.7, 29.5, 29.5, 29.4, 26.2, 22.8, 14.3.

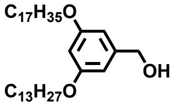

50d

(3-(Heptadecyloxy)-5-(tridecyloxy)phenyl)methanol (**50f**). Compound **49f** (0.73 g, 1.24 mmol, 1 equiv) and $LiAlH_4$ (47 mg, 1.24 mmol, 1 equiv) following a procedure similar to that used for the synthesis of compound 23a. The title compound was obtained as a colorless oil (0.65 g, 93%). ^{1}H NMR (400 MHz, $CDCl_3$) δ 6.50 (br, 2 H, Ph*H*), 6.38 (t, 1 H, Ph*H*), 4.61 (s, 2 H, $PhC\mathit{H}_2OH$), 3.93 (t, 4 H, $PhOC\mathit{H}_2CH_2CH_2$-), 1.77 (m, 4 H, $PhOCH_2C\mathit{H}_2CH_2$-), 1.44 (m, 4 H, $PhOCH_2CH_2C\mathit{H}_2$-), 1.28 (m, 44 H,

PhOCH$_2$CH$_2$CH$_2$(C*H$_2$*)$_{13}$CH$_3$ and PhOCH$_2$CH$_2$CH$_2$(C*H$_2$*)$_9$CH$_3$), 0.88 (t, 6 H, PhO(CH$_2$)$_{16}$C*H$_3$* and PhO(CH$_2$)$_{12}$C*H$_3$*). ^{13}C NMR (101 MHz, CDCl$_3$) δ 160.7, 160.7, 143.4, 143.3, 105.2, 100.7, 100.7, 68.2, 65.6, 65.5, 32.1, 29.8, 29.8, 29.7, 29.5, 29.5, 29.4, 26.2, 22.8, 14.3. Mp = 46 °C.

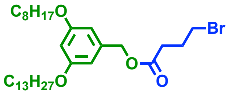

51a

3-(Octyloxy)-5-(tridecyloxy)benzyl 4-bromobutanoate (51a). Compound **50a** (0.47 g, 1.08 mmol, 1 equiv), 4-bromobutyric acid (0.20 g, 1.19 mmol, 1.1 equiv) and DPTS (0.34 g, 1.19 mmol, 1.1 equiv) were dissolved in 8 mL DCM. DCC (0.45 g, 2.16 mmol, 2 equiv) was added into the above mixture. The reaction mixture was stirred at 23 °C for 12 h. Afterwards, the resulted precipitates (urea) were filtered out from the reaction mixture. The filtrate was concentrated to around 3 mL and purified by column chromatography (SiO$_2$) with hexane/DCM = 1/1 as the mobile phase to give the title compound as a colorless oil (0.46 g, 74%). ^{1}H NMR (400 MHz, CDCl$_3$) δ 6.47 (br, 2 H, Ph*H*), 6.41 (t, 1 H, Ph*H*), 5.05 (s, 2 H, PhC*H$_2$*OCO), 3.93 (t, 4 H, PhOC*H$_2$*CH$_2$CH$_2$-), 3.46 (t, 2 H, -OCOCH$_2$CH$_2$C*H$_2$*Br), 2.56 (t, 2 H, -OCOC*H$_2$*CH$_2$CH$_2$Br), 2.20 (m, 2 H, -OCOCH$_2$C*H$_2$*CH$_2$Br), 1.77 (m, 4 H, PhOCH$_2$C*H$_2$*CH$_2$-), 1.44 (m, 4 H, PhOCH$_2$CH$_2$C*H$_2$*-), 1.28 (m, 26 H, PhOCH$_2$CH$_2$CH$_2$(C*H$_2$*)$_4$CH$_3$ and PhOCH$_2$CH$_2$CH$_2$(C*H$_2$*)$_9$CH$_3$), 0.89 (t, 6 H, PhO(CH$_2$)$_7$C*H$_3$* and PhO(CH$_2$)$_{12}$C*H$_3$*). ^{13}C NMR (101 MHz, CDCl$_3$) δ 172.4, 160.6, 137.9, 106.5, 101.2, 77.4, 68.2, 66.5, 32.8, 32.6, 32.1, 32.0, 29.8, 29.8, 29.7, 29.7, 29.5, 29.5, 29.4, 27.9, 26.2, 22.8, 22.8, 14.3, 14.2.

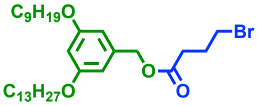

51b

3-(Nonyloxy)-5-(tridecyloxy)benzyl 4-bromobutanoate (51b). Compound **51b** was synthesized from compound **50b** (0.45 g, 1.00 mmol, 1 equiv), 4-bromobutyric acid (0.25 g, 1.50 mmol, 1.5 equiv), DPTS (0.32 g, 1.50 mmol, 1.5 equiv) and DCC (0.62 g, 3.00 mmol, 3 equiv) following a procedure similar to that used for the synthesis of compound **51b**. The title compound was obtained as a colorless oil (0.60 g, 75%). ^{1}H NMR (400 MHz, CDCl$_3$) δ 6.46 (br, 2 H, Ph*H*), 6.40 (t, 1 H, Ph*H*), 5.04 (s, 2 H, PhC*H$_2$*OCO), 3.93 (t, 4 H, PhOC*H$_2$*CH$_2$CH$_2$-), 3.46 (t, 2 H, -OCOCH$_2$CH$_2$C*H$_2$*Br), 2.56 (t, 2 H, -OCOC*H$_2$*CH$_2$CH$_2$Br), 2.20 (m, 2 H, -OCOCH$_2$C*H$_2$*CH$_2$Br), 1.76 (m, 4 H, PhOCH$_2$C*H$_2$*CH$_2$-), 1.44 (m, 4 H, PhOCH$_2$CH$_2$C*H$_2$*-),

1.28 (m, 28 H, $PhOCH_2CH_2CH_2(C\mathit{H}_2)_5CH_3$ and $PhOCH_2CH_2CH_2(C\mathit{H}_2)_9CH_3$), 0.88 (t, 6 H, $PhO(CH_2)_9C\mathit{H}_3$ and $PhO(CH_2)_{12}C\mathit{H}_3$). ^{13}C NMR (101 MHz, $CDCl_3$) δ 172.4, 172.4, 160.6, 138.0, 106.5, 101.2, 77.5, 77.2, 76.9, 68.3, 68.2, 66.6, 66.5, 32.8, 32.7, 32.6, 32.1, 29.8, 29.8, 29.7, 29.6, 29.5, 29.5, 29.4, 27.9, 26.2, 22.8, 14.3.

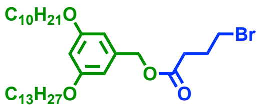

51c

3-(Decyloxy)-5-(tridecyloxy)benzyl 4-bromobutanoate (51c). Compound **51c** was synthesized from compound **50c** (0.60 g, 1.30 mmol, 1 equiv), 4-bromobutyric acid (0.33 g, 1.94 mmol, 1.5 equiv), DPTS (0.46 g, 1.94 mmol, 1.5 equiv) and DCC (0.81 g, 3.90 mmol, 3 equiv) following a procedure similar to that used for the synthesis of compound **51c**. The title compound was obtained as a colorless oil (0.60 g, 75%). ^1H NMR (400 MHz, $CDCl_3$) δ 6.46 (br, 2 H, Ph*H*), 6.40 (t, 1 H, Ph*H*), 5.04 (s, 2 H, $PhC\mathit{H}_2OCO$), 3.93 (t, 4 H, $PhOC\mathit{H}_2CH_2CH_2$-), 3.46 (t, 2 H, -$OCOCH_2CH_2C\mathit{H}_2Br$), 2.56 (t, 2 H, -$OCOC\mathit{H}_2CH_2CH_2Br$), 2.20 (m, 2 H, -$OCOCH_2C\mathit{H}_2CH_2Br$), 1.76 (m, 4 H, $PhOCH_2C\mathit{H}_2CH_2$-), 1.44 (m, 4 H, $PhOCH_2CH_2C\mathit{H}_2$-), 1.28 (m, 30 H, $PhOCH_2CH_2CH_2(C\mathit{H}_2)_6CH_3$ and $PhOCH_2CH_2CH_2(C\mathit{H}_2)_9CH_3$), 0.88 (t, 6 H, $PhO(CH_2)_9C\mathit{H}_3$ and $PhO(CH_2)_{12}C\mathit{H}_3$). ^{13}C NMR (101 MHz, $CDCl_3$) δ 172.4, 172.4, 160.6, 138.0, 106.5, 101.2, 77.5, 77.2, 76.9, 68.3, 68.2, 66.6, 66.5, 32.8, 32.7, 32.6, 32.1, 29.8, 29.8, 29.7, 29.6, 29.5, 29.5, 29.4, 27.9, 26.2, 22.8, 14.3.

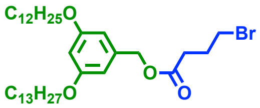

51d

3-(Dodectyloxy)-5-(tridecyloxy)benzyl 4-bromobutanoate (51d). Compound **51d** was synthesized from compound 29c (0.51 g, 1.04 mmol, 1 equiv), 4-bromobutyric acid (0.26 g, 1.56 mmol, 1.5 equiv), DPTS (0.46 g, 1.56 mmol, 1.5 equiv) and DCC (0.64 g, 3.12 mmol, 3 equiv) following a procedure similar to that used for the synthesis of compound **51d**. The title compound was obtained as a colorless oil (0.58 g, 87%). ^1H NMR (400 MHz, $CDCl_3$) δ 6.47 (br, 2 H, Ph*H*), 6.41 (t, 1 H, Ph*H*), 5.05 (s, 2 H, $PhC\mathit{H}_2OCO$), 3.93 (t, 4 H, $PhOC\mathit{H}_2CH_2CH_2$-), 3.47 (t, 2 H, -$OCOCH_2CH_2C\mathit{H}_2Br$), 2.57 (t, 2 H, -$OCOC\mathit{H}_2CH_2CH_2Br$), 2.20 (m, 2 H, -$OCOCH_2C\mathit{H}_2CH_2Br$), 1.77 (m, 4 H, $PhOCH_2C\mathit{H}_2CH_2$-), 1.45 (m, 4 H, $PhOCH_2CH_2C\mathit{H}_2$-), 1.27 (m, 34 H, $PhOCH_2CH_2CH_2(C\mathit{H}_2)_8CH_3$ and $PhOCH_2CH_2CH_2(C\mathit{H}_2)_9CH_3$), 0.88 (t, 6 H,

PhO(CH2)7CH3 and PhO(CH2)12CH3). ^{13}C NMR (101 MHz, CDCl3) δ 172.4, 160.6, 137.9, 106.5, 101.2, 77.4, 68.2, 66.5, 32.7, 32.6, 32.1, 29.8, 29.8, 29.7, 29.7, 29.6, 29.5, 29.5, 29.4, 27.9, 26.2, 22.8, 14.3.

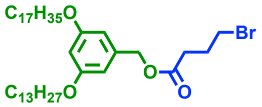

51f

3-(Heptectyloxy)-5-(tridecyloxy)benzyl 4-bromobutanoate (51f). Compound **51f** was synthesized from compound 29c (0.30 g, 0.54 mmol, 1 equiv), 4-bromobutyric acid (0.13 g, 0.80 mmol, 1.5 equiv), DPTS (0.24 g, 0.80 mmol, 1.5 equiv) and DCC (0.33 g, 0.60 mmol, 3 equiv) following a procedure similar to that used for the synthesis of compound **51f**. The title compound was obtained as a colorless oil (0.28 g, 74%). ^{1}H NMR (400 MHz, CDCl3) δ 6.47 (br, 2 H, Ph*H*), 6.40 (t, 1 H, Ph*H*), 5.05 (s, 2 H, PhC*H2*OCO), 3.93 (t, 4 H, PhOC*H2*CH2CH2-), 3.47 (t, 2 H, -OCOCH2CH2C*H2*Br), 2.57 (t, 2 H, -OCOC*H2*CH2CH2Br), 2.20 (m, 2 H, -OCOCH2C*H2*CH2Br), 1.76 (m, 4 H, PhOCH2C*H2*CH2-), 1.44 (m, 4 H, PhOCH2CH2C*H2*-), 1.28 (m, 44 H, PhOCH2CH2CH2(C*H2*)13CH3 and PhOCH2CH2CH2(C*H2*)9CH3), 0.88 (t, 6 H, PhO(CH2)7C*H3* and PhO(CH2)12C*H3*). ^{13}C NMR (101 MHz, CDCl3) δ 172.4, 160.6, 137.9, 106.5, 101.2, 68.2, 66.6, 32.8, 32.6, 32.1, 29.8, 29.8, 29.8, 29.7, 29.7, 29.6, 29.5, 29.4, 27.9, 26.2, 22.8, 14.3.

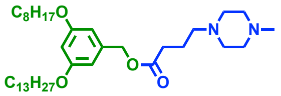

52a (IAJD163)

3-(Octyloxy)-5-(tridecyloxy)benzyl 4-(4-methylpiperazin-1-yl)butanoate (52a, IAJD163). Compound **52a** was synthesized from compound **51a** (280 mg, 0.48 mmol, 1 equiv), 1-methylpiperazine (72 mg, 0.72 mmol, 1.5 equiv) and K_2CO_3 (80 mg, 0.58 mmol) in 10ml MeCN was heated to 95 °C and stirred for 3 h. The reaction mixture was cooled to 23 °C and MeCN was removed under reduced pressure. Then water (20 mL) was added, and the resulted mixture was extracted with DCM (20 mL) for three times. The organic phases were collected and dried over anhydrous $MgSO_4$. After filtration, the obtained filtrate was concentrated to around 3 mL and purified by column chromatography (SiO_2) with DCM/MeOH = 30/1 and 15/1 as the eluent. Then the obtained product was dissolved in DCM (20 mL), which was washed by $NaHCO_3$ solution (2%, 20 mL). The aqueous phase was extracted by DCM (20 mL) for another two times. The organic phases were combined and dried over anhydrous $MgSO_4$. Filtration and evaporation of the solvent yielded the title compound as a colorless oil (0.20 g, 87%). ^{1}H NMR (400 MHz, CDCl3) δ 6.45 (br, 2 H, Ph*H*), 6.38 (t, 1 H, Ph*H*), 5.02 (s, 2 H, PhC*H2*-), 3.91 (t, 4 H, PhOC*H2*-), 2.62–2.26 (m, 14 H, -N(C*H2*C*H2*)2N-, -NC*H2*CH2C*H2*COO- and -C*H2*CH2OH), 1.83 (m, 2 H, -OCOCH2C*H2*CH2-), 1.74 (m, 4

H, PhOCH$_2$C*H*$_2$-), 1.43 (m, 4 H, PhOCH$_2$CH$_2$C*H*$_2$-), 1.27 (br, 28 H, PhOCH$_2$CH$_2$CH$_2$(C*H*$_2$)$_5$CH$_3$ and PhOCH$_2$CH$_2$CH$_2$(C*H*$_2$)$_9$CH$_3$), 0.87 (t, 6 H, -CH$_2$CH$_2$C*H*$_3$). ^{13}C NMR (101 MHz, CDCl$_3$) δ 173.1, 160.4, 138.1, 106.3, 100.8, 77.5, 77.2, 76.9, 68.0, 66.0, 57.4, 55.0, 52.8, 45.8, 32.1, 31.9, 31.8, 29.7, 29.6, 29.6, 29.6, 29.6, 29.4, 29.3, 29.2, 29.2, 26.0, 22.7, 22.6, 22.1, 14.1, 14.1. Purity by HPLC: 99+%. MALDI-TOF MS m/z of $[M + H]^+$ calculated for $C_{37}H_{67}N_2O_4$: 603.6; Found: 604.3.

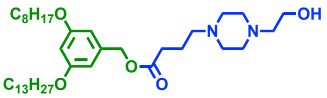

52b (IAJD164)

3-(Octyloxy)-5-(tridecyloxy)benzyl 4-(4-(2-hydroxyethyl)piperazin-1-yl)butanoate (52b, IAJD164). Compound 52b was synthesized from compound 51b (278 mg, 0.48 mmol, 1equiv), 1-(2-hydroxyethyl)piperazine (98 mg, 0.72 mmol, 1.5 equiv) and K_2CO_3 (80 mg, 0.58 mmol, 1.2 equiv) following a procedure similar to that used for the synthesis of compound **52a**. The title compound was obtained as a colorless oil (230 mg, 76%). ^{1}H NMR (400 MHz, CDCl$_3$) δ 6.47 (s, 2 H, Ph*H*), 6.39 (t, 1 H, Ph*H*), 5.03 (s, 2 H, PhC*H*$_2$-), 3.92 (t, 4 H, PhOC*H*$_2$-), 3.62 (t, 2 H, -CH$_2$C*H*$_2$OH), 2.63–2.27 (m, 14 H, -N(C*H*$_2$C*H*$_2$)$_2$N-, -NC*H*$_2$CH$_2$C*H*$_2$COO- and -C*H*$_2$CH$_2$OH), 1.86 (m, 2 H, -OCOCH$_2$C*H*$_2$CH$_2$-), 1.74 (m, 4 H, PhOCH$_2$C*H*$_2$-), 1.43 (m, 4 H, PhOCH$_2$CH$_2$C*H*$_2$-), 1.27 (br, 28 H, PhOCH$_2$CH$_2$CH$_2$(C*H*$_2$)$_5$CH$_3$ and PhOCH$_2$CH$_2$CH$_2$(C*H*$_2$)$_9$CH$_3$), 0.89 (t, 6 H, -CH$_2$CH$_2$C*H*$_3$). ^{13}C NMR (101 MHz, CDCl$_3$) δ 173.3, 160.4, 138.1, 106.4, 100.9, 68.1, 66.2, 59.3, 59.3, 57.6, 57.5, 53.4, 53.0, 52.8, 32.2, 31.9, 31.9, 29.7, 29.7, 29.6, 29.6, 29.5, 29.4, 29.4, 29.3, 26.1, 22.7, 22.7, 22.1, 14.1. Purity by HPLC: 99+%. MALDI-TOF MS m/z of [M + H]+ calculated for $C_{38}H_{68}N_2O_5$: 634.3; Found: 634.0.

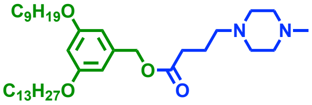

52c (IAJD165)

3-(nonyloxy)-5-(tridecyloxy)benzyl 4-(4-methylpiperazin-1-yl)butanoate (52c, IAJD165). Compound **52c** was synthesized from compound **51b** (0.27 g, 0.44 mmol), 1-methylpiperazine (53 mg, 0.53 mmol) and K_2CO_3 (74 mg, 0.53 mmol) following a procedure similar to that used for the synthesis of compound **52a**. The title compound was obtained as a light-yellow oil (0.23 g, 85 %). ^{1}H NMR (400 MHz, CDCl$_3$) δ 6.45 (d, 2 H, Ph*H*), 6.39 (t, 1 H, Ph*H*), 5.02 (s, 2 H, -C*H*$_2$Ph), 3.92 (t, 4 H, 2×PhOC*H*$_2$-), 2.77–2.14 (m, 15 H, -N(C*H*$_2$C*H*$_2$)$_2$N-, -NC*H*$_2$CH$_2$C*H*$_2$COO- and -NC*H*$_3$), 1.83 (m, 2 H, - OCOCH$_2$C*H*$_2$CH$_2$-), 1.75 (m, 4 H, PhOCH$_2$C*H*$_2$-), 1.43 (m, 4 H, PhOCH$_2$CH$_2$C*H*$_2$-), 1.25 (br, 28 H, PhOCH$_2$CH$_2$CH$_2$(C*H*$_2$)$_9$CH$_3$ and

$PhOCH_2CH_2CH_2(CH_2)_5CH_3$), 0.87 (t, 6 H, $-CH_2CH_2CH_3$). ^{13}C NMR (101 MHz, $CDCl_3$) δ 173.5, 160.6, 138.2, 106.5, 101.5, 68.2, 66.3, 57.7, 55.3, 53.2, 46.2, 32.4, 32.1, 32.0, 29.8, 29.8, 29.8, 29.7, 29.7, 29.5, 29.5, 29.4, 26.2, 22.8, 22.8, 22.3, 14.2. Purity by HPLC: 99+%. MALDI-TOF MS *m*/*z* of $[M + H]^+$ calculated for $C_{38}H_{69}N_2O_4$: 617.5; Found: 618.4.

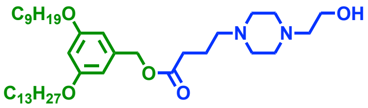

52d (IAJD166)

3-(nonyloxy)-5-(tridecyloxy)benzyl 4-(4-(2-hydroxyethyl)piperazin-1-yl)butanoate (52d, IAJD166). Compound **52d** was synthesized from compound **51b** (0.26 g, 0.44 mmol), 1-(2- hydroxyethyl)piperazine (69 mg, 0.53 mmol) and K_2CO_3 (74 mg, 0.53 mmol) following a procedure similar to that used for the synthesis of compound **52a**. The title compound was obtained as a light-yellow oil (0.18 g, 62 %). ^1H NMR (400 MHz, $CDCl_3$) δ 6.46 (d, 2 H, Ph*H*), 6.39 (t, 1 H, Ph*H*), 5.03 (s, 2 H, - CH_2Ph), 3.92 (t, 4 H, $2\times PhOCH_2$-), 3.60 (t, 2 H, $-CH_2CH_2OH$), 2.74–2.12 (m, 15 H, $-CH_2CH_2OH$, - $N(CH_2CH_2)_2N$-, -$OCOCH_2CH_2CH_2$- and $-CH_2CH_2OH$), 1.83 (m, 2 H, $-OCOCH_2CH_2CH_2$-), 1.75 (m, 4 H, $PhOCH_2CH_2$-), 1.43 (m, 4 H, $PhOCH_2CH_2CH_2$-), 1.26 (br, 28 H, $PhOCH_2CH_2CH_2(CH_2)_9CH_3$ and $PhOCH_2CH_2CH_2(CH_2)_5CH_3$), 0.88 (t, 6 H, $-CH_2CH_2CH_3$). ^{13}C NMR (101 MHz, $CDCl_3$) δ 173.5, 160.6, 138.2, 106.5, 101.0, 68.2, 66.3, 59.3, 57.8, 57.7, 53.3, 53.0, 32.4, 32.0, 32.0, 29.8, 29.8, 29.8, 29.7, 29.7, 29.5, 29.5, 29.4, 26.2, 22.8, 22.8, 22.3, 14.3. Purity by HPLC: 99+%. MALDI-TOF MS *m*/*z* of $[M + H]^+$ calculated for $C_{39}H_{71}N_2O_5$: 647.5; Found: 647.2.

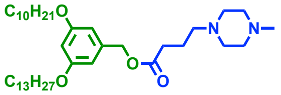

52c (IAJD167)

3-(Decyloxy)-5-(tridecyloxy)benzyl 4-(4-methylpiperazin-1-yl)butanoate (52c, IAJD167). Compound **52c** was synthesized from compound **51b** (0.25 g, 0.41 mmol), 1-methylpiperazine (45 mg, 0.45 mmol) and K_2CO_3 (68 mg, 0.49 mmol) following a procedure similar to that used for the synthesis of compound **52a**. The title compound was obtained as a colorless oil (0.24 g, 93%). ^1H NMR (400 MHz, $CDCl_3$) δ 6.45 (br, 2 H, Ph*H*), 6.38 (t, 1 H, Ph*H*), 5.01 (s, 2 H, $PhCH_2$-), 3.91 (t, 4 H, $PhOCH_2$-), 2.73–2.19 (m, 15 H, -$N(CH_2CH_2)_2N$-, $-NCH_2CH_2CH_2COO$- and $-NCH_3$), 1.81 (m, 2 H, $-OCOCH_2CH_2CH_2$-), 1.75 (m, 4 H, $PhOCH_2CH_2$-), 1.43 (m, 4 H, $PhOCH_2CH_2CH_2$-), 1.27 (br, 30 H, $PhOCH_2CH_2CH_2(CH_2)_6CH_3$ and

$PhOCH_2CH_2CH_2(CH_2)_9CH_3$), 0.87 (t, 6 H, $-CH_2CH_2CH_3$). ^{13}C NMR (101 MHz, $CDCl_3$) δ 173.2, 160.5, 138.1, 106.4, 100.9, 68.1, 66.1, 57.5, 55.0, 52.8, 45.8, 32.2, 31.9, 31.9, 29.7, 29.7, 29.7, 29.6, 29.6, 29.6, 29.4, 29.4, 29.3, 29.3, 26.1, 22.7, 22.1, 14.1. Purity by HPLC: 99+%. MALDI-TOF MS m/z of $[M + H]^+$ calculated for $C_{39}H_{71}N_2O_4$: 631.5; Found: 631.4.

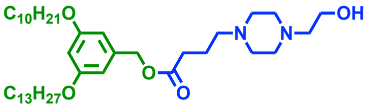

52f (IAJD168)

3-(Dectyloxy)-5-(tridecyloxy)benzyl 4-(4-(2-hydroxyethyl)piperazin-1-yl)butanoate (52f, IAJD168). Compound **52f** was synthesized from compound **51c** (0.14 g, 0.21 mmol), 1-(2-hydroxyethyl)piperazine (29 mg, 0.22 mmol) and K_2CO_3 (31 mg, 0.22 mmol) following a procedure similar to that used for the synthesis of compound **52a**. The title compound was obtained as a colorless a colorless oil (0.24 g, 93%). ^1H NMR (400 MHz, $CDCl_3$) δ 6.45 (br, 2 H, Ph*H*), 6.38 (t, 1 H, Ph*H*), 5.02 (s, 2 H, $PhCH_2$-), 3.91 (t, 4 H, $PhOCH_2$-), 3.60 (t, 2H, $-CH_2CH_2OH$), 2.65–2.24 (m, 14 H, $-N(CH_2CH_2)_2N$-, $-NCH_2CH_2CH_2COO$- and $-CH_2CH_2OH$), 1.84 (m, 2 H, $-OCOCH_2CH_2CH_2$-), 1.75 (m, 4 H, $PhOCH_2CH_2$-), 1.42 (m, 4 H, $PhOCH_2CH_2CH_2$-), 1.28 (br, 30 H, $PhOCH_2CH_2CH_2(CH_2)_6CH_3$ and $PhOCH_2CH_2CH_2(CH_2)_9CH_3$), 0.87 (t, 6 H, $-CH_2CH_2CH_3$). ^{13}C NMR (101 MHz, $CDCl_3$) δ 173.4, 160.6, 138.2, 106.5, 101.0, 77.4, 68.2, 66.3, 59.4, 57.8, 57.6, 53.1, 53.0, 32.3, 32.0, 32.0, 29.8, 29.8, 29.8, 29.7, 29.7, 29.7, 29.5, 29.5, 29.4, 29.4, 26.2, 22.8, 22.2, 14.2. Purity by HPLC: 99+%. MALDI-TOF MS m/z of $[M + H]^+$ calculated for $C_{40}H_{73}N_2O_5$: 661.55; Found: 662.6.

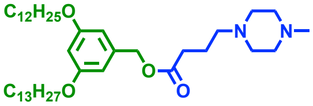

52g (IAJD169)

3-(dodecyloxy)-5-(tridecyloxy)benzyl 4-(4-methylpiperazin-1-yl)butanoate (52g, IAJD169). Compound **52g** was synthesized from compound **51d** (0.3 g, 0.47 mmol), 1-methylpiperazine (56 mg, 0.56 mmol) and K_2CO_3 (78 mg, 0.56 mmol) following a procedure similar to that used for the synthesis of compound **52a**. The title compound was obtained as a light-yellow oil (0.26 g, 85 %). ^1H NMR (400 MHz, $CDCl_3$) δ 6.46 (d, 2 H, Ph*H*), 6.39 (t, 1 H, Ph*H*), 5.02 (s, 2 H, $-CH_2Ph$), 3.92 (t, 4 H, $2\times PhOCH_2$-), 2.77–2.14 (m, 15 H, $-N(CH_2CH_2)_2N$-, $-NCH_2CH_2CH_2COO$- and $-NCH_3$), 1.83 (m, 2 H, $-OCOCH_2CH_2CH_2$-), 1.75 (m, 4 H, $PhOCH_2CH_2$-), 1.44 (m, 4 H, $PhOCH_2CH_2CH_2$-), 1.26 (br, 34 H, $PhOCH_2CH_2CH_2(CH_2)_9CH_3$ and $PhOCH_2CH_2CH_2(CH_2)_8CH_3$), 0.88 (t, 6 H, $-CH_2CH_2CH_3$). ^{13}C NMR

(101 MHz, $CDCl_3$) δ 173.5, 160.6, 138.2, 106.5, 101.1, 68.2, 66.3, 57.7, 55.3, 53.3, 46.2, 32.4, 32.6, 29.8, 29.8, 29.8, 29.7, 29.6, 29.5, 29.4, 26.2, 22.8, 22.4, 14.3. Purity by HPLC: 99+%. MALDI-TOF MS *m*/*z* of $[M + H]^+$ calculated for $C_{41}H_{75}N_2O_4$: 659.6 ; Found: 659.0 .

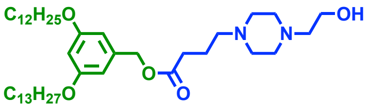

52h (IAJD170)

3-(dodecyloxy)-5-(tridecyloxy)benzyl 4-(4-(2-hydroxyethyl)piperazin-1-yl)butanoate (52h, IAJD170). Compound **52h** was synthesized from compound **51d** (0.3 g, 0.47 mmol), 1-(2-hydroxyethyl)piperazine (73 mg, 0.56 mmol) and K_2CO_3 (77 mg, 0.56 mmol) following a procedure similar to that used for the synthesis of compound **52a**. The title compound was obtained as a light-yellow oil (0.28 g, 87 %). ^1H NMR (400 MHz, $CDCl_3$) δ 6.46 (d, 2 H, Ph*H*), 6.39 (t, 1 H, Ph*H*), 5.02 (s, 2 H, -C*H*$_2$Ph), 3.92 (t, 4 H, 2×PhOC*H*$_2$-), 3.59 (t, 2 H, -CH$_2$C*H*$_2$OH), 2.75–2.24 (m, 15 H, -CH$_2$CH$_2$O*H*, -N(C*H*$_2$C*H*$_2$)$_2$N-, -OCOC*H*$_2$CH$_2$C*H*$_2$- and -C*H*$_2$CH$_2$OH), 1.83 (m, 2 H, -OCOCH$_2$C*H*$_2$CH$_2$-), 1.76 (m, 4 H, PhOCH$_2$C*H*$_2$-), 1.43 (m, 4 H, PhOCH$_2$CH$_2$C*H*$_2$-), 1.26 (br, 34 H, PhOCH$_2$CH$_2$CH$_2$(C*H*$_2$)$_9$CH$_3$ and PhOCH$_2$CH$_2$CH$_2$(C*H*$_2$)$_8$CH$_3$), 0.88 (t, 6 H, -CH$_2$CH$_2$C*H*$_3$). ^{13}C NMR (101 MHz, $CDCl_3$) δ 173.5, 160.6, 138.2, 106.6, 101.0, 68.2, 66.3, 59.3, 57.8, 57.7, 53.3, 53.0, 32.4, 32.6, 29.8, 29.8, 29.8, 29.8, 29.7, 29.5, 29.5, 29.4, 26.2, 22.8, 22.3, 14.3. Purity by HPLC: 99+%. MALDI-TOF MS *m*/*z* of $[M + H]^+$ calculated for $C_{42}H_{77}N_2O_5$: 689.6; Found: 689.5.

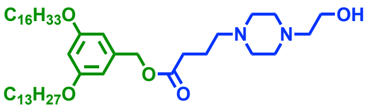

52i (IAJD174)

3-(Hexadecyloxy)-5-(tridecyloxy)benzyl 4-(4-(2-hydroxyethyl)piperazin-1-yl)butanoate (52i, IAJD174). Compound **52i** was synthesized from compound **51e** (0.14 g, 0.20 mmol), 1-(2-hydroxyethyl)piperazine (34 mg, 0.24 mmol) and K_2CO_3 (40 mg, 0.30 mmol) following a procedure similar to that used for the synthesis of compound **52a**. The title compound was obtained as a colorless a colorless oil (0.14 g, 90%). ^1H NMR (400 MHz, $CDCl_3$) δ 6.45 (br, 2 H, Ph*H*), 6.38 (t, 1 H, Ph*H*), 5.02 (s, 2 H, PhC*H*$_2$-), 3.91 (t, 4 H, PhOC*H*$_2$-), 3.61 (t, 2H, -CH$_2$C*H*$_2$OH), 2.64–2.31 (m, 14 H, -N(C*H*$_2$C*H*$_2$)$_2$N-,

-NCH_2CH$_2$CH_2COO- and -CH_2CH$_2$OH), 1.84 (m, 2 H, -OCOCH$_2$CH_2CH$_2$-), 1.75 (m, 4 H, PhOCH$_2$CH_2-), 1.43 (m, 4 H, PhOCH$_2$CH$_2$CH_2-), 1.25 (br, 42 H, PhOCH$_2$CH$_2$CH$_2$(CH_2)$_{12}$CH$_3$ and PhOCH$_2$CH$_2$CH$_2$(CH_2)$_9$CH$_3$), 0.87 (t, 6 H, -CH$_2$CH$_2$CH_3). ^{13}C NMR (101 MHz, CDCl$_3$) δ 173.2, 160.5, 138.1, 106.4, 100.9, 77.3, 68.1, 66.1, 59.3, 57.6, 57.5, 52.9, 52.8, 32.2, 31.9, 29.7, 29.7, 29.6, 29.6, 29.6, 29.5, 29.4, 29.4, 29.3, 26.1, 22.7, 22.1, 14.1. Purity by HPLC: 99+%. MALDI-TOF MS m/z of [M + H]$^+$ calculated for $C_{46}H_{85}N_2O_5$: 745.65; Found: 747.9.

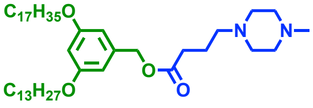

52j (IAJD175)

3-(heptadecyloxy)-5-(tridecyloxy)benzyl 4-(4-methylpiperazin-1-yl)butanoate (52j, IAJD175). Compound **52j** was synthesized from compound **51f** (0.14 g, 0.20 mmol), 1-methylpiperazine (25 mg, 0.25 mmol) and K_2CO_3 (35 mg, 0.25 mmol) following a procedure similar to that used for the synthesis of compound **52a**. The title compound was obtained as a light-yellow oil (0.13 g, 90 %). ^{1}H NMR (400 MHz, CDCl$_3$) δ 6.46 (d, 2 H, Ph*H*), 6.39 (t, 1 H, Ph*H*), 5.03 (s, 2 H, -CH_2Ph), 3.92 (t, 4 H, 2×PhOCH_2-), 2.77–2.14 (m, 15 H, -N(CH_2CH_2)$_2$N-, -NCH_2CH$_2$CH_2COO- and -NCH_3), 1.83 (m, 2 H, -OCOCH$_2$CH_2CH$_2$-), 1.76 (m, 4 H, PhOCH$_2$CH_2-), 1.43 (m, 4 H, PhOCH$_2$CH$_2$CH_2-), 1.26 (br, 44 H, PhOCH$_2$CH$_2$CH$_2$(CH_2)$_9$CH$_3$ and PhOCH$_2$CH$_2$CH$_2$(CH_2)$_{13}$CH$_3$), 0.88 (t, 6 H, -CH$_2$CH$_2$CH_3). ^{13}C NMR (101 MHz, CDCl$_3$) δ 173.5, 160.6, 138.2, 106.5, 101.1, 68.2, 66.3, 57.8, 55.3, 53.3, 46.2, 32.4, 32.1, 29.8, 29.8, 29.8, 29.7, 29.6, 29.5, 29.4, 26.2, 22.8, 22.4, 14.3. Purity by HPLC: 99+%. MALDI-TOF MS *m/z* of [M + H]$^+$ calculated for $C_{46}H_{85}N_2O_4$: 729.6; Found: 729.6.

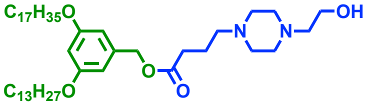

52k (IAJD176)

3-(heptadecyloxy)-5-(tridecyloxy)benzyl 4-(4-(2-hydroxyethyl)piperazin-1-yl)butanoate (52k, IAJD176). Compound **52k** was synthesized from compound **51f** (0.14 g, 0.20 mmol), 1-(2-hydroxyethyl)piperazine (33 mg, 0.25 mmol) and K_2CO_3 (35 mg, 0.25 mmol) following a procedure similar to that used for the synthesis of compound **52a**. The title compound was obtained as a light-yellow oil (0.10 g, 67 %). ^{1}H NMR (400 MHz, CDCl$_3$) δ 6.46 (d, 2 H, Ph*H*), 6.39 (t, 1 H, Ph*H*), 5.03 (s, 2 H, -CH_2Ph), 3.92 (t, 4 H, 2×PhOCH_2-), 3.60 (t, 2 H, -CH$_2$CH_2OH), 2.63–2.23 (m, 15 H, -CH$_2$CH$_2$O*H*, -

$N(C\mathit{H}_2C\mathit{H}_2)_2N$-, -$OCOC\mathit{H}_2CH_2C\mathit{H}_2$- and -$C\mathit{H}_2CH_2OH$), 1.82 (m, 2 H, -$OCOCH_2C\mathit{H}_2CH_2$-), 1.76 (m, 4 H, $PhOCH_2C\mathit{H}_2$-), 1.43 (m, 4 H, $PhOCH_2CH_2C\mathit{H}_2$-), 1.26 (br, 44 H, $PhOCH_2CH_2CH_2(C\mathit{H}_2)_9CH_3$ and $PhOCH_2CH_2CH_2(C\mathit{H}_2)_{13}CH_3$), 0.88 (t, 6 H, -$CH_2CH_2C\mathit{H}_3$). ^{13}C NMR (101 MHz, $CDCl_3$) δ 173.5, 160.6, 138.2, 106.6, 101.0, 68.3, 66.3, 59.3, 57.8, 57.7, 53.2, 53.0, 32.4, 32.0, 29.9, 29.8, 29.8, 29.7, 29.6, 29.5, 29.4, 26.2, 22.8, 22.3, 14.3. Purity by HPLC: 99+%. MALDI-TOF MS *m*/*z* of $[M + H]^+$ calculated for $C_{47}H_{86}N_2O_5$: 759.7; Found: 759.6 .

3.9 Synthesis of IAJD131

Scheme S9. Synthesis of IAJD131

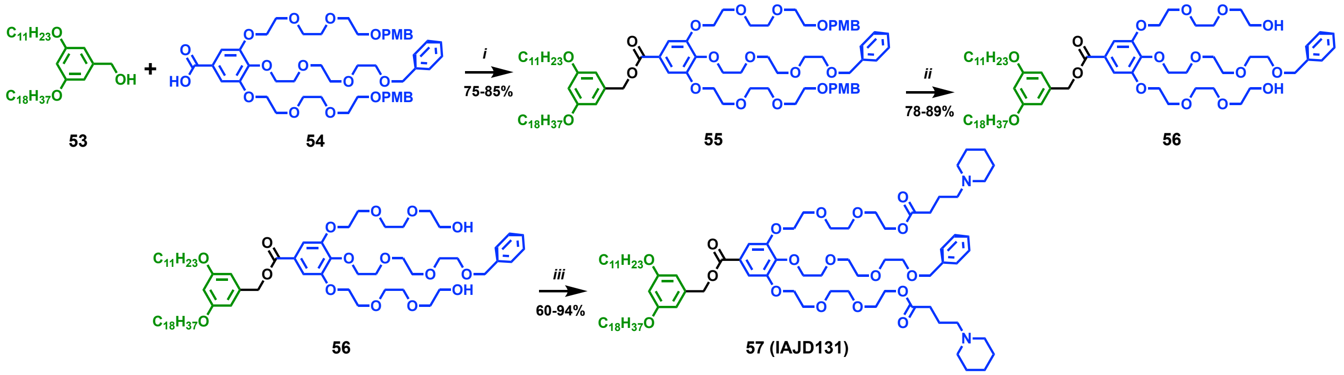

Reagents and conditions: (*i*) EDC.HCl, DMAP, DCM, 23 °C, 12 h; (*ii*) DDQ, DCM/H_2O, 23 °C, 1 h; (*iii*) 4-(piperidin-1-yl)butanoic acid hydrochloride, DCC, DCM, 23 °C, 12 h.

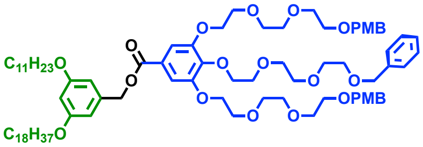

55

Compound 55. Compound **53** was synthesized according to a literature procedure reported by our laboratory.[5] Compound **54** was synthesized according to a literature procedure elaborated and optimized by our laboratory.[4] Compound **53** (0.28 g, 0.51 mmol, 1.1 equiv), compound **54** (0.41 g, 0.46 mmol, 1.0 equiv), EDC.HCl (0.98 g, 0.51 mmol, 1.1 equiv) and DMAP (17 mg, 0.14 mmol, 0.3 equiv) were dissolved in dry DCM (6 mL). The reaction mixture was stirred at 23 °C for 12 h. Brine (20 mL) was then added, and the mixture was extracted by DCM (20 mL) for three times. The organic phases were collected and dried over anhydrous $MgSO_4$. After filtration, the obtained filtrate was concentrated to around 3 mL and purified by column chromatography (SiO_2) with EtOAc/hexane = 1/1 as the mobile phase to give the title

compound as a colorless oil (0.53 g, 100%). ^{1}H NMR (400 MHz, $CDCl_3$) δ 7.25 (m, 11 H, Ph*H*), 6.86–6.78 (m, 4 H, Ph*H*), 6.51 (d, 2 H, Ph*H*), 6.39 (t, 1 H, Ph*H*), 5.21 (s, 2 H, $-OC\mathit{H}_2Ph$), 4.51 (s, 2 H, $-C\mathit{H}_2Ph$), 4.44 (s, 4 H, $-OC\mathit{H}_2Ph$), 4.17 (m, 6 H, $PhOC\mathit{H}_2CH_2O$-), 3.89 (t, 4 H, $PhOC\mathit{H}_2CH_2CH_2$-), 3.78 (m, 4 H, $PhOCH_2C\mathit{H}_2O$-), 3.74 (m, 8 H, $PhOCH_2C\mathit{H}_2O$- and $PhOC\mathit{H}_3$), 3.68 (m, 6 H, $PhOCH_2CH_2OC\mathit{H}_2$-), 3.61–3.55 (m, 18 H, $PhOCH_2CH_2OCH_2C\mathit{H}_2OC\mathit{H}_2C\mathit{H}_2$-), 1.73 (m, 4 H, $PhOCH_2C\mathit{H}_2CH_2$-), 1.41 (m, 4 H, $PhOCH_2CH_2C\mathit{H}_2$-), 1.23 (br, 42 H, $PhOCH_2CH_2CH_2(C\mathit{H}_2)_7CH_3$ and $PhOCH_2CH_2CH_2(C\mathit{H}_2)_{14}CH_3$), 0.85 (t, 6 H, $-CH_2CH_2C\mathit{H}_3$). ^{13}C NMR (101 MHz, $CDCl_3$) δ 165.9, 160.5, 159.2, 152.3, 142.8, 138.3, 138.1, 130.4, 129.4, 128.3, 127.7, 127.6, 124.9, 113.7, 109.3, 106.6, 101.0, 73.2, 72.9, 72.5, 70.9, 70.7, 70.7, 70.7, 70.6, 70.6, 69.7, 69.5, 69.1, 69.0, 68.1, 66.8, 55.2, 31.9, 31.9, 29.7, 29.7, 29.6, 29.6, 29.4, 29.4, 29.4, 29.3, 26.1, 22.7, 14.2.

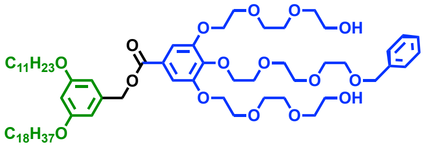

56

Compound 56. Compound **55** (0.45 g, 0.30 mmol, 1 equiv) was dissolved in 6 mL DCM and 0.42 mL water (7 v%) was added. To this solution was added 2,3-dichloro-5,6-dicyano-1,4-benzoquinone (DDQ, 0.15 g, 0.66 mmol, 2.2 equiv). The reaction mixture was stirred at 23 °C for 1 h. Then the precipitates were filtered out and DCM (20 mL) was added. The mixture was washed by $NaHCO_3$ aqueous solution (saturated), $NaHSO_3$ aqueous solution (2%) and $NaHCO_3$ aqueous solution (saturated) successively. The organic phases were collected and dried over anhydrous $MgSO_4$. After filtration, the obtained filtrate was concentrated and purified by column chromatography (SiO_2) with EtOAc/MeOH = 20/1 as the mobile phase to give the title compound as a light-yellow oil (0.45 g, 78%). ^{1}H NMR (400 MHz, $CDCl_3$) δ 7.36–7.16 (m, 7 H, Ph*H*), 6.50 (d, 2 H, Ph*H*), 6.38 (t, 1 H, Ph*H*), 5.20 (s, 2 H, $-OC\mathit{H}_2Ph$), 4.51 (s, 2 H, $-OC\mathit{H}_2Ph$), 4.18 (m, 6 H, $PhOC\mathit{H}_2CH_2O$-), 3.92 (t, 4 H, $PhOC\mathit{H}_2CH_2CH_2$-), 3.84 (t, 6 H, $PhOCH_2C\mathit{H}_2O$-), 3.77–3.47 (m, 24 H, $PhOCH_2CH_2OC\mathit{H}_2C\mathit{H}_2OC\mathit{H}_2C\mathit{H}_2$-), 3.04 (br, 2H, $-OCH_2CH_2O\mathit{H}$), 1.76 (m, 4 H, $PhOCH_2C\mathit{H}_2CH_2$-), 1.40 (m, 4 H, $PhOCH_2CH_2C\mathit{H}_2$-), 1.24 (br, 42 H, $PhOCH_2CH_2CH_2(C\mathit{H}_2)_7CH_3$ and $PhOCH_2CH_2CH_2(C\mathit{H}_2)_{14}CH_3$), 0.84 (t, 6 H, $-CH_2CH_2C\mathit{H}_3$). ^{13}C NMR (101 MHz, $CDCl_3$) δ 165.9, 160.4, 152.2, 142.7, 138.2, 138.0, 128.3, 127.7, 127.6, 124.9, 109.2, 107.8, 106.6, 100.9, 73.2, 72.6, 72.4, 70.8, 70.6, 70.6, 70.5, 70.5, 70.4, 69.6, 69.4, 68.9, 68.1, 67.5, 66.8, 61.6, 60.4, 31.9, 31.9, 29.7, 29.6, 29.6, 29.6, 29.4, 29.3, 29.3, 29.2, 29.2, 26.0, 23.9, 22.7, 14.1.

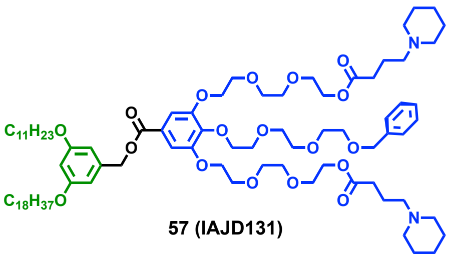

Compound 57 (IAJD131). Compound **56** (0.21 g, 0.18 mmol, 1 equiv) and 4-(piperidin-1-yl)butanoic acid hydrochloride (83 mg, 0.40 mmol, 2.2 equiv) were dissolved in 6 mL dry DCM. DCC (0.11 g, 0.54 mmol, 3 equiv) was added in one portion into the above mixture. The reaction mixture was stirred at 23 °C for 12 h. Afterwards, the resulted precipitates (urea) were filtered out from the reaction mixture. The filtrate was concentrated to around 3 mL and purified by column chromatography (SiO_2) with DCM/MeOH = 30/1, 10/1, 8/1 and 8/1 with 0.1% NEt_3 as the mobile phase. Then the product was dissolved in DCM (20 mL), which was washed by $NaHCO_3$ aqueous solution (2%, 20 mL). The aqueous phase was extracted by DCM (20 mL) for another two times. The organic phases were combined and dried over anhydrous $MgSO_4$. Filtration and evaporation of the solvent yielded the title compound as a colorless oil (0.23 g, 85%). ^{1}H NMR (400 MHz, $CDCl_3$) δ 7.29 (m, 7 H, Ph*H*), 6.50 (d, 2 H, Ph*H*), 6.38 (t, 1 H, Ph*H*), 5.20 (s, 2 H, -OCH_2Ph), 4.51 (s, 2 H, -OCH_2Ph), 4.16 (m, 10 H, PhOCH_2CH$_2$O- and -OCH$_2$CH_2OCO(CH$_2$)$_3$-), 3.89 (t, 4 H, PhOCH_2CH$_2$CH$_2$-), 3.80 (t, 6 H, PhOCH$_2$CH_2O-), 3.63 (m, 20 H, PhOCH$_2$CH$_2$OCH_2CH_2OCH_2CH_2-), 2.27 (m, 16 H, N(CH_2-)$_2$CH_2CH$_2$CH_2COO-), 1.84–1.73 (m, 4 H, PhOCH$_2$CH_2(CH$_2$)$_8$CH$_3$, PhOCH$_2$CH_2(CH$_2$)$_{15}$CH$_3$ and N(CH$_2$-)$_2$CH$_2$CH_2CH$_2$COO-), 1.58–1.53 (m, 8 H, N(CH$_2$CH_2)$_2$CH$_2$), 1.44 (m, 8 H, PhOCH$_2$CH$_2$CH_2(CH$_2$)$_7$CH$_3$, PhOCH$_2$CH$_2$CH_2(CH$_2$)$_{14}$CH$_3$ and N(CH$_2$CH$_2$)$_2$CH_2), 1.26 (br, 42 H, PhOCH$_2$CH$_2$CH$_2$(CH_2)$_7$CH$_3$ and PhOCH$_2$CH$_2$CH$_2$(CH_2)$_{14}$CH$_3$), 0.84 (t, 6 H, -CH$_2$CH$_2$CH_3). ^{13}C NMR (101 MHz, $CDCl_3$) δ 173.5, 165.8, 160.4, 152.3, 142.8, 138.3, 138.0, 128.3, 127.7, 127.5, 124.9, 109.3, 106.5, 73.2, 72.4, 70.8, 70.7, 70.6, 70.6, 70.6, 69.7, 69.4, 69.2, 68.9, 68.0, 66.8, 63.4, 58.4, 54.5, 50.0, 32.2, 31.9, 31.9, 29.7, 29.6, 29.6, 29.6, 29.4, 29.3, 29.3, 29.2, 26.2, 26.0, 26.0, 24.5, 24.4, 22.7, 22.3, 22.2, 14.1. Purity by HPLC: 99+%. MALDI-TOF MS *m*/*z* of $[M + H]^+$ calculated for $C_{86}H_{143}N_2O_{18}$: 1492.0; Found: 1492.5.

3.10 Synthesis of Decane-Based IAJDs with 3,5-Nonsymmetric Alkyl Chains

Scheme S10. Synthesis of Decane-Based IAJDs with 3,5-Nonsymmetric Alkyl Chains

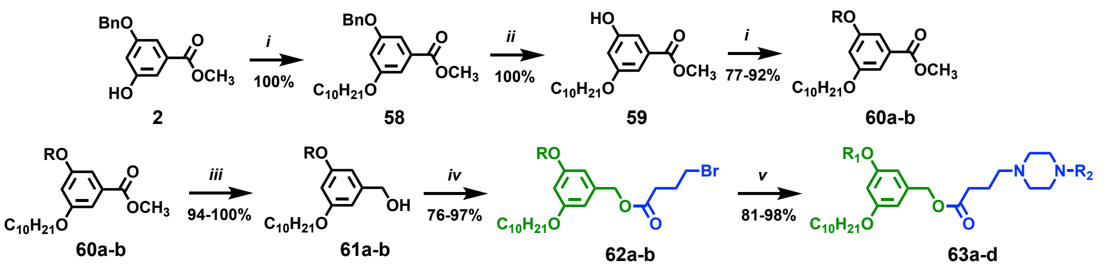

60-62a: R = C_8H_{17}
60-62b: R = R = 2-ethylhexyl

63a (IAJD244): R_1 = C_8H_{17}; R_2 = CH_3
63b (IAJD245): R_1 = C_8H_{17}; R_2 = CH_2CH_2OH
63c (IAJD246): R_1 = 2-ethylhexyl; R_2 = CH_3
63d (IAJD247): R_1 = 2-ethylhexyl; R_2 = CH_2CH_2OH

Reagents and conditions: (*i*) RBr/ROTs, K_2CO_3, DMF, 120 °C, 2 h; (*ii*) H_2, Pd/C, DCM, MeOH, 12 h; (*iii*) $LiAlH_4$, THF, 0–23 °C, 1 h; (*iv*) DCC, DPTS, DCM, 12 h; (*v*) K_2CO_3, MeCN, 95 °C, 3 h.

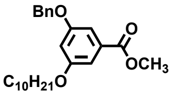

58

Methyl 3-(benzyloxy)-5-(decyloxy)benzoate (58). A mixture of compound **2** (4.00 g, 15.49 mmol, 1 equiv), 1-Bromodecane (1.41 g, 6.39 mmol, 1.1 equiv), K_2CO_3 (1.6 g, 11.6 mmol, 2 equiv) and DMF (10 mL) was heated to 120 °C and stirred under N_2 atmosphere for 2 h. The reaction mixture was cooled to 23 °C and poured into ice/water (150 mL). The resulted white precipitates in the solution were filtered and collected. Then the precipitates were recrystallized from minimum acetone to afford the title compound as a white solid (1.26 g, 70%). ^{1}H NMR (400 MHz, $CDCl_3$) δ 7.49–7.33 (m, 5 H, Ph*H*), 7.29 (br, 1 H, Ph*H*), 7.21 (br, 1 H, Ph*H*), 6.73 (t, 1 H, Ph*H*), 5.08 (s, 2 H, PhC*H*$_2$O-), 3.97 (t, 2 H, PhOC*H*$_2$-), 3.90 (s, 3 H, PhCOOC*H*$_3$), 1.78 (m, 2 H, $PhOCH_2CH_2CH_2(CH_2)_6CH_3$ with C*H*$_2$ at position 2), 1.43 (m, 2 H, PhOCH$_2$CH$_2$C*H*$_2$(CH$_2$)$_6$CH$_3$), 1.28 (m, 12 H, PhOCH$_2$CH$_2$CH$_2$(C*H*$_2$)$_6$CH$_3$), 0.89 (t, 3 H, PhO(CH$_2$)$_{10}$C*H*$_3$). ^{13}C NMR (101 MHz, $CDCl_3$) δ 167.9, 160.2, 159.8, 136.6, 132.0, 128.6, 128.1, 127.6, 108.2, 107.8, 107.0, 70.3, 68.4, 52.2, 32.0, 29.6, 29.5, 29.4, 29.3, 29.2, 26.0, 22.7, 14.1. Mp = 51 °C.

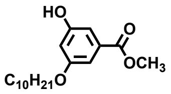

59

Methyl 3-(decyloxy)-5-hydroxybenzoate (59). Compound **59** (1.26 g, 3.16 mmol) was dissolved in a mixture of DCM (30 mL) and methanol (15 mL). Then Pd/C (0.06 g, 5 wt%) was added to the solution and the flask was evacuated and filled with hydrogen for three times. Afterwards, the mixture was stirred at 23 °C under hydrogen atmosphere for 12 h. The reaction mixture was filtered through Celite, and the filter cake was washed with DCM. Evaporation of the solvent yielded the title compound as a white solid (2.70 g, 100%). ^{1}H NMR (400 MHz, $CDCl_3$) δ 7.13 (d, 2 H, Ph*H*), 6.64 (t, 1 H, Ph*H*), 5.32 (s, 1 H, PhO*H*),

3.95 (t, 2 H, PhOCH_2-), 3.89 (s, 3 H, PhCOOCH_3), 1.75 (m, 2 H, PhOCH$_2$CH_2CH$_2$(CH$_2$)$_6$CH$_3$), 1.44 (m, 2 H, PhOCH$_2$CH$_2$CH_2(CH$_2$)$_6$CH$_3$), 1.26 (m, 12 H, PhOCH$_2$CH$_2$CH$_2$(CH_2)$_6$CH$_3$), 0.88 (t, 3 H, PhO(CH$_2$)$_{17}$CH_3). ^{13}C NMR (101 MHz, CDCl$_3$) δ 167.2, 160.4, 156.9, 131.9, 109.1, 107.8, 107.1, 68.4, 52.4, 31.9, 29.6, 29.6, 29.4, 29.3, 29.2, 26.0, 22.7, 14.1. Mp = 99 °C.

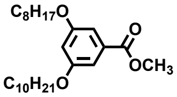

60a

Methyl 3-(decyloxy)-5-(octyloxy)benzoate (60a). A mixture of compound **59** (0360 mg, 1.17 mmol, 1 equiv), 1-bromodecane (250 mg, 1.29 mmol, 1.1 equiv), K_2CO_3 (320 mg, 2.34 mmol, 2 equiv) and DMF (15 mL) was heated to 120 °C and stirred under N_2 atmosphere for 2 h. The reaction mixture was cooled to 23 °C and poured into ice/water (150 mL). The resulted white precipitates in the solution were filtered and collected. Then the precipitates were recrystallized from minimum acetone to afford the title compound as a white solid (0.28 g, 57%). ^{1}H NMR (400 MHz, CDCl$_3$) δ 7.15 (br, 2 H, PhH), 6.63 (t, 1 H, PhH), 3.95 (t, 4 H, PhOCH_2CH$_2$CH$_2$-), 3.89 (s, 3 H, PhCOOCH_3), 1.77 (m, 4 H, PhOCH$_2$CH_2CH$_2$-), 1.44 (m, 4 H, PhOCH$_2$CH$_2$CH_2-), 1.26 (m, 20 H, PhOCH$_2$CH$_2$CH$_2$(CH_2)$_4$CH$_3$ and PhOCH$_2$CH$_2$CH$_2$(CH_2)$_6$CH$_3$), 0.88 (t, 6 H, PhO(CH$_2$)$_7$CH_3 and PhO(CH$_2$)$_9$CH_3). ^{13}C NMR (101 MHz, CDCl$_3$) δ 167.0, 160.2, 131.8, 107.7, 106.6, 68.3, 52.2, 31.9, 31.8, 29.6, 29.6, 29.4, 29.3, 29.3, 29.2, 29.2, 26.0, 22.7, 22.7, 14.1, 14.1. Mp = 33 °C.

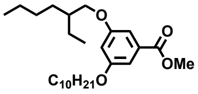

60b

Methyl 3-(decyloxy)-5-((2-ethylhexyl)oxy)benzoate (60b). A mixture of compound **59** (0.36 g, .17 mmol, 1 equiv), 2-ethylhexyl bromide (250 mg, 1.29 mmol, 1.1 equiv), K_2CO_3 (320 mg, 2.34 mmol, 2 equiv) and DMF (15 mL) was heated to 80 °C and stirred under N_2 atmosphere for 12 h. The reaction mixture was cooled to 23 °C and DMF was removed under reduced pressure. Then water (20 mL) was added, and the resulted mixture was extracted with DCM (20 mL) for three times. The organic phases were collected and dried over anhydrous $MgSO_4$. After filtration, the obtained filtrate was concentrated to around 3 mL and purified by column chromatography (SiO_2) with hexane/EtOAc = 20/1 as the eluent to give the title compound as a light-yellow oil (0.34 g, 69%). ^{1}H NMR (400 MHz, CDCl$_3$) δ 7.17 (br, 2 H, PhH), 6.68 (t, 1 H, PhH), 3.97 (t, 2 H, PhOCH_2(CH$_2$)$_{16}$CH$_3$), 3.90 (s, 3 H, PhCOOCH_3), 3.86 (m, 2 H, PhOCH_2CH(CH$_2$CH$_3$)-), 1.85–1.69 (m, 3 H, PhOCH$_2$CH_2(CH$_2$)$_7$CH$_3$ and PhOCH$_2$CH(CH$_2$CH$_3$)(CH$_2$)$_3$CH$_3$), 1.52–1.17 (m, 22 H, PhOCH$_2$CH$_2$(CH_2)$_7$CH$_3$ and

PhOCH$_2$CH(C*H*$_2$CH$_3$)(C*H*$_2$)$_3$CH$_3$), 0.90 (m, 9 H, PhO(CH$_2$)$_9$C*H*$_3$ and PhOCH$_2$CH(CH$_2$C*H*$_3$)(CH$_2$)$_3$C*H*$_3$). ^{13}C NMR (101 MHz, CDCl$_3$) δ 167.0, 160.4, 160.1, 131.8, 107.7, 107.5, 106.6, 70.8, 68.3, 52.1, 39.4, 31.9, 30.5, 29.6, 29.6, 29.4, 29.3, 29.2, 29.1, 26.0, 25.2, 23.9, 23.0, 22.7, 14.1, 14.1, 11.1.

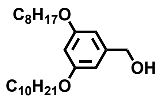

61a

(3-(Decyloxy)-5-(octyloxy)phenyl)methanol (61a). Compound **61a** (0.28 g, 0.67 mmol, 1 equiv) was dissolved in 5 mL dry THF, which was added dropwise to a slurry of LiAlH$_4$ (25 mg, 0.67 mmol, 1 equiv) in dry THF (5 mL) at 0 °C under N$_2$ atmosphere. Afterwards, the resulted mixture was stirred at 23 °C for 1 h. Finally, the reaction was quenched by the successive addition of water (0.2 mL), 15% NaOH aqueous solution (0.2 mL) and water (1.0 mL). White precipitates were formed in the mixture and filtered out. The filtrate was dried over anhydrous MgSO$_4$ and filtered. The solvent was removed under reduced pressure to afford the title compound as a colorless oil (0.52 g, 81%). ^{1}H NMR (400 MHz, CDCl$_3$) δ 6.49 (br, 2 H, Ph*H*), 6.37 (t, 1 H, Ph*H*), 4.59 (s, 2 H, PhC*H*$_2$OH), 3.93 (t, 4 H, PhOC*H*$_2$CH$_2$CH$_2$-), 1.77 (m, 4 H, PhOCH$_2$C*H*$_2$CH$_2$-), 1.44 (m, 4 H, PhOCH$_2$CH$_2$C*H*$_2$-), 1.29 (m, 20 H, PhOCH$_2$CH$_2$CH$_2$(C*H*$_2$)$_4$CH$_3$ and PhOCH$_2$CH$_2$CH$_2$(C*H*$_2$)$_6$CH$_3$), 0.91 (t, 6 H, PhO(CH$_2$)$_7$C*H*$_3$ and PhO(CH$_2$)$_9$C*H*$_3$). ^{13}C NMR (101 MHz, CDCl$_3$) δ 160.6, 143.2, 105.1, 100.6, 68.1, 65.5, 31.9, 31.8, 30.3, 29.6, 29.6, 29.4, 29.4, 29.3, 29.3, 29.3, 26.1, 22.7, 22.7, 14.1, 14.1.

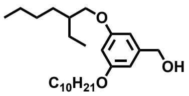

61b

(3-(Decyloxy)-5-((2-ethylhexyl)oxy)phenyl)methanol (61b). Compound **61b** was synthesized from compound **60b** (0.34 g, 0.81 mmol, 1 equiv) and LiAlH$_4$ (31 mg, 0.81 mmol, 1 equiv) following a procedure similar to that used for the synthesis of compound **61a**. The title compound was obtained as a colorless oil (240 mg, 73%). ^{1}H NMR (400 MHz, CDCl$_3$) δ 6.50 (br, 2 H, Ph*H*), 6.38 (t, 1 H, Ph*H*), 4.60 (s, 2 H, PhC*H*$_2$OH), 3.93 (t, 2 H, PhOC*H*$_2$CH$_2$CH$_2$-), 3.81 (m, 2 H, PhOC*H*$_2$CH(CH$_2$CH$_3$)(CH$_2$)$_3$CH$_3$), 1.73 (m, 4 H, PhOCH$_2$C*H*$_2$CH$_2$-), 1.43 (m, 6 H, PhOCH$_2$CH$_2$C*H*$_2$- and PhOCH$_2$CH(C*H*$_2$CH$_3$)C*H*$_2$(CH$_2$)$_2$CH$_3$), 1.27 (m, 16 H, PhOCH$_2$CH$_2$CH$_2$(C*H*$_2$)$_6$CH$_3$ and PhOCH$_2$CH(CH$_2$CH$_3$)CH$_2$(C*H*$_2$)$_2$CH$_3$), 0.93 (m, 9 H, PhO(CH$_2$)$_9$C*H*$_3$ and PhOCH$_2$CH(CH$_2$C*H*$_3$)CH$_2$(CH$_2$)$_2$C*H*$_3$). ^{13}C NMR (101 MHz, CDCl$_3$) δ 160.8, 160.6, 143.2, 105.1, 105.0, 100.6, 70.6, 68.1, 65.5, 39.4, 31.9, 30.6, 30.3, 29.6, 29.6, 29.4, 29.3, 29.3, 29.1, 26.1, 23.9, 23.1, 22.7, 14.1, 14.1, 11.1.

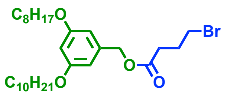

62a

3-(Decyloxy)-5-(octyloxy)benzyl 4-bromobutanoate (62a). Compound **61a** (0.22 g, 0.54 mmol, 1 equiv), 4-bromobutyric acid (0.11 g, 0.60 mmol, 1.1 equiv), and DPTS (0.16 g, 0.54 mmol, 1 equiv) were dissolved in 8 mL DCM. DCC (0.23 g, 1.08 mmol, 2 equiv) was added into the above mixture. The reaction mixture was stirred at 23 °C for 12 h. Afterwards, the resulted precipitates (urea) were filtered out from the reaction mixture. The filtrate was concentrated to around 3 mL and purified by column chromatography (SiO_2) with hexane/DCM = 1/1 as the mobile phase to give the title compound as a colorless oil (0.28 g, 63%). ^{1}H NMR (400 MHz, $CDCl_3$) δ 6.47 (br, 2 H, Ph*H*), 6.41 (t, 1 H, Ph*H*), 5.05 (s, 2 H, PhC*H*$_2$OCO), 3.93 (t, 4 H, PhOC*H*$_2$CH$_2$CH$_2$-), 3.46 (t, 2 H, -OCOCH$_2$CH$_2$C*H*$_2$Br), 2.56 (t, 2 H, -OCOC*H*$_2$CH$_2$CH$_2$Br), 2.20 (m, 2 H, -OCOCH$_2$C*H*$_2$CH$_2$Br), 1.77 (m, 4 H, PhOCH$_2$C*H*$_2$CH$_2$-), 1.44 (m, 4 H, PhOCH$_2$CH$_2$C*H*$_2$-), 1.28 (m, 20 H, PhOCH$_2$CH$_2$CH$_2$(C*H*$_2$)$_6$CH$_3$ and PhOCH$_2$CH$_2$CH$_2$(C*H*$_2$)$_4$CH$_3$), 0.89 (t, 6 H, PhO(CH$_2$)$_7$C*H*$_3$ and PhO(CH$_2$)$_9$C*H*$_3$). ^{13}C NMR (101 MHz, $CDCl_3$) δ 172.3, 160.5, 137.8, 106.4, 101.2, 68.1, 66.4, 32.6, 32.5, 31.9, 31.8, 29.6, 29.6, 29.4, 29.4, 29.4, 29.3, 29.3, 27.8, 26.1, 22.7, 22.7, 14.1.

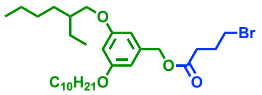

62b

3-(Decyloxy)-5-(3-ethylheptyl)benzyl 4-bromobutanoate (62b). Compound **62b** was synthesized from compound **61b** (0.22 g, 0.54 mmol, 1 equiv), 4-bromobutyric acid (0.11 g, 0.60 mmol, 1.1 equiv), DPTS (0.16 g, 0.54 mmol, 1 equiv) and DCC (0.22 g, 1.08 mmol, 2 equiv) following a procedure similar to that used for the synthesis of compound **62a**. The title compound was obtained as a colorless oil (200 mg, 82%). ^{1}H NMR (400 MHz, $CDCl_3$) δ 6.47 (br, 2 H, Ph*H*), 6.40 (t, 1 H, Ph*H*), 5.02 (s, 2 H, PhC*H*$_2$OCO), 3.93 (t, 2 H, PhOC*H*$_2$CH$_2$CH$_2$-), 3.81 (m, 2 H, PhOC*H*$_2$CH(CH$_2$CH$_3$)(CH$_2$)$_3$CH$_3$), 3.46 (t, 2 H, -OCOCH$_2$CH$_2$C*H*$_2$Br), 2.58 (t, 2 H, -OCOC*H*$_2$CH$_2$CH$_2$Br), 2.19 (m, 2 H, -OCOCH$_2$C*H*$_2$CH$_2$Br), 1.73 (m, 3 H, PhOCH$_2$C*H*$_2$CH$_2$- and PhOCH$_2$C*H*(CH$_2$CH$_3$)(CH$_2$)$_3$CH$_3$), 1.47 (m, 6 H, PhOCH$_2$CH$_2$C*H*$_2$- and PhOCH$_2$CH(C*H*$_2$CH$_3$)C*H*$_2$(CH$_2$)$_2$CH$_3$), 1.27 (m, 16 H, PhOCH$_2$CH$_2$CH$_2$(C*H*$_2$)$_6$CH$_3$ PhOCH$_2$CH(CH$_2$CH$_3$)CH$_2$(C*H*$_2$)$_2$CH$_3$), 0.90 (m, 9 H, PhO(CH$_2$)$_9$C*H*$_3$ and PhOCH$_2$CH(CH$_2$C*H*$_3$)(CH$_2$)$_3$C*H*$_3$). ^{13}C NMR (101 MHz, $CDCl_3$) δ 172.3, 160.8, 160.5, 137.8, 106.5, 106.3, 101.1, 70.5, 68.1, 66.4, 39.4, 32.6, 31.9, 30.6, 29.6, 29.6, 29.4, 29.3, 29.3, 29.1, 27.8, 26.1, 23.9, 23.1, 22.7, 14.1, 14.1, 11.1.

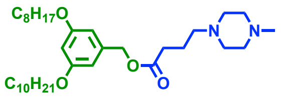

63a (IAJD244)

3-(Decyloxy)-5-(octyloxy)benzyl 4-(4-methylpiperazin-1-yl)butanoate (63a, IAJD244). A mixture of compound **62a** (0.14 g, 0.17 mmol, 1 equiv), 1-methylpiperazine (26 mg, 0.26 mmol, 1.5 equiv), K_2CO_3 (28 mg, 0.20 mmol, 1.2 equiv) and MeCN (10 mL) was heated to 95 °C and stirred for 3 h. The reaction mixture was cooled to 23 °C and MeCN was removed under reduced pressure. Then water (20 mL) was added, and the resulted mixture was extracted with DCM (20 mL) for three times. The organic phases were collected and dried over anhydrous MgSO4. After filtration, the obtained filtrate was concentrated to around 3 mL and purified by column chromatography (SiO2) with DCM/MeOH = 30/1 and 15/1 as the eluent. Then the obtained product was dissolved in DCM (20 mL), which was washed by $NaHCO_3$ solution (2%, 20 mL). The aqueous phase was extracted by DCM (20 mL) for another two times. The organic phases were combined and dried over anhydrous $MgSO_4$. Filtration and evaporation of the solvent yielded the title compound as a colorless oil (60 mg, 63%). ^{1}H NMR (400 MHz, $CDCl_3$) δ 6.45 (p, 2 H, Ph*H*), 6.37 (t, 1 H, Ph*H*), 5.01 (s, 2 H, PhCH_2-), 3.89 (t, 4 H, PhOCH_2-), 2.78–2.24 (m, 15 H, -N(CH_2CH_2)$_2$N-, -NCH_2CH$_2$CH_2COO- and -NCH_3), 1.81 (m, 2 H, -OCOCH$_2$CH_2CH$_2$-), 1.73 (m, 4 H, PhOCH$_2$CH_2-), 1.44 (m, 4 H, PhOCH$_2$CH$_2$CH_2-), 1.28 (br, 20 H, PhOCH$_2$CH$_2$CH$_2$(CH_2)$_6$CH$_3$ and PhOCH$_2$CH$_2$CH$_2$(CH_2)$_4$CH$_3$), 0.88 (t, 6 H, -CH$_2$CH$_2$CH_3). ^{13}C NMR (101 MHz, $CDCl_3$) δ 173.3, 160.5, 138.1, 106.4, 100.9, 68.1, 66.2, 57.4, 54.9, 52.6, 45.7, 32.2, 31.9, 31.9, 29.6, 29.6, 29.4, 29.4, 29.3, 29.3, 29.2, 26.1, 22.7, 22.7, 22.1, 14.1, 14.1. Purity by HPLC: 99+%. MALDI-TOF MS m/z of $[M + H]^+$ calculated for $C_{34}H_{61}N_2O_4$: 561.5; Found: 562.5.

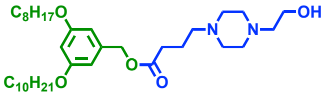

63b (IAJD245)

3-(Decyloxy)-5-(octyloxy)benzyl 4-(4-(2-hydroxyethyl)piperazin-1-yl)butanoate (63b, IAJD245). Compound **63b** was synthesized from compound **62a** (90 mg, 0.17 mmol, 1 equiv), 1-(2-hydroxyethyl)piperazine (34 mg, 0.26 mmol, 1.5 equiv) and K_2CO_3 (28 mg, 0.20 mmol, 1.2 equiv) following a procedure similar to that used for the synthesis of compound **63a**. The title compound was obtained as a colorless oil (80 mg, 84%). ^{1}H NMR (400 MHz, $CDCl_3$) δ 6.44 (s, 2 H, Ph*H*), 6.39 (t, 1 H, Ph*H*), 5.01 (s, 2 H, PhCH_2-), 3.90 (t, 4 H, PhOCH_2-), 3.61 (t, 2H, -CH$_2$CH_2OH), 2.54–2.33 (m, 14 H, -N(CH_2CH_2)$_2$N-, -NCH_2CH$_2$CH_2COO- and -CH_2CH$_2$OH), 1.83 (m, 2 H, -OCOCH$_2$CH_2CH$_2$-), 1.73 (m, 4 H, PhOCH$_2$CH_2-), 1.41 (m, 4 H, PhOCH$_2$CH$_2$CH_2-), 1.26 (br, 20 H, PhOCH$_2$CH$_2$CH$_2$(CH_2)$_4$CH$_3$ and

PhOCH$_2$CH$_2$CH$_2$(C*H$_2$*)$_6$CH$_3$), 0.89 (t, 6 H, -CH$_2$CH$_2$C*H$_3$*). ^{13}C NMR (101 MHz, CDCl$_3$) δ 173.3, 160.5, 138.1, 106.4, 100.1, 68.1, 66.1, 66.2, 59.4, 57.6, 57.5, 52.8, 52.8, 32.2, 31.9, 31.8, 29.6, 29.6, 29.4, 29.4, 29.3, 29.3, 29.3, 29.2, 26.1, 22.7, 22.7, 22.0, 14.1, 14.1. Purity by HPLC: 99+%. MALDI-TOF MS m/z of [M + H]$^+$ calculated for C$_{35}$H$_{63}$N$_2$O$_5$: 591.5; Found: 591.9.

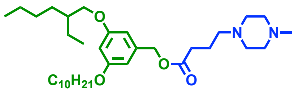

63c (IAJD246)

3-(Decyloxy)-5-((2-ethylhexyl)oxy)benzyl 4-(4-methylpiperazin-1-yl)butanoate (63c, IAJD246). Compound **63c** was synthesized from compound **62b** (100 mg, 0.19 mmol, 1 equiv), 1-methylpiperazine (29 mg, 0.29 mmol, 1.5 equiv) and K$_2$CO$_3$ (32 mg, 0.23 mmol, 1.2 equiv) following a procedure similar to that used for the synthesis of compound **63a**. The title compound was obtained as a colorless oil (60 mg, 56%). ^{1}H NMR (400 MHz, CDCl$_3$) δ 6.44 (p, 2 H, Ph*H*), 6.37 (t, 1 H, Ph*H*), 5.00 (s, 2 H, PhC*H$_2$*-), 3.90 (t, 4 H, PhOC*H$_2$*-), 2.78–2.15 (m, 15 H, -N(C*H$_2$*C*H$_2$*)$_2$N-, -NC*H$_2$*CH$_2$C*H$_2$*COO- and -NC*H$_3$*), 1.83 (m, 2 H, -OCOCH$_2$C*H$_2$*CH$_2$-), 1.70 (m, 4 H, PhOCH$_2$C*H$_2$*-), 1.44 (m, 4 H, PhOCH$_2$CH$_2$C*H$_2$*-), 1.42-1.27 (br, 18 H, PhOCH$_2$CH$_2$CH$_2$(C*H$_2$*)$_6$CH$_3$ and PhOCH$_2$CH(C*H$_2$*CH$_3$)(CH$_2$)$_3$CH$_3$), 0.89 (t, 9 H, PhO(CH$_2$)$_9$C*H$_3$* and PhOCH$_2$CH(CH$_2$C*H$_3$*)(CH$_2$)$_3$C*H$_3$*). ^{13}C NMR (101 MHz, CDCl$_3$) δ 173.3, 160.7, 160.5, 138.0, 106.5, 106.3, 100.9, 77.5, 68.1, 66.2, 57.5, 55.0, 52.3, 45.8, 39.4, 32.0, 31.9, 29.6, 29.6, 29.4, 29.3, 29.3, 29.1, 26.1, 23.9, 23.0, 22.7, 22.1, 14.1, 14.1, 11.1. Purity by HPLC: 99+%. MALDI-TOF MS m/z of [M + H]$^+$ calculated for C$_{34}$H$_{61}$N$_2$O$_4$: 561.5; Found: 562.3.

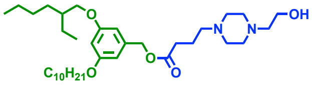

63d (IAJD247)

3-(Decyloxy)-5-((2-ethylhexyl)oxy)benzyl 4-(4-(2-hydroxyethyl)piperazin-1-yl)butanoate (63d, IAJD247). Compound **63d** was synthesized from compound **62b** (100 mg, 0.19 mmol, 1 equiv), 1-(2-hydroxyethyl)piperazine (38 mg, 0.29 mmol, 1.5 equiv) and K$_2$CO$_3$ (32 mg, 0.23 mmol, 1.2 equiv) following a procedure similar to that used for the synthesis of compound **63a**. The title compound was obtained as a colorless oil (80 mg, 75%). ^{1}H NMR (400 MHz, CDCl$_3$) δ 6.44 (br, 2 H, Ph*H*), 6.38 (t, 1 H, Ph*H*), 5.01 (s, 2 H, PhC*H$_2$*-), 3.88 (t, 4 H, PhOC*H$_2$*-), 3.60 (t, 2H, -CH$_2$C*H$_2$*OH), 2.65–2.29 (m, 14 H, -N(C*H$_2$*C*H$_2$*)$_2$N-, -NC*H$_2$*CH$_2$C*H$_2$*COO- and -C*H$_2$*CH$_2$OH), 1.81 (m, 2 H, -OCOCH$_2$C*H$_2$*CH$_2$-), 1.71 (m, 4 H, PhOCH$_2$C*H$_2$*-), 1.47 (m, 4 H, PhOCH$_2$CH$_2$C*H$_2$*-), 1.27 (br, 18 H, PhOCH$_2$CH$_2$CH$_2$(C*H$_2$*)$_6$CH$_3$ and PhOCH$_2$CH(CH$_2$CH$_3$)(C*H$_2$*)$_3$CH$_3$), 0.92 (t, 9 H, PhO(CH$_2$)$_9$C*H$_3$* and PhOCH$_2$CH(CH$_2$C*H$_3$*)(CH$_2$)$_3$C*H$_3$*). ^{13}C NMR (101 MHz, CDCl$_3$) δ 173.3, 160.7, 160.5, 138.0, 106.5, 106.3, 100.9, 70.5, 68.1, 66.2, 59.3,

57.6, 57.5, 52.9, 52.8, 39.4, 32.2, 31.9, 31.9, 30.5, 29.7, 29.6, 29.6, 29.4, 29.3, 29.3, 29.1, 26.1, 23.9, 23.0, 22.7, 22.0, 14.1, 14.1, 11.1. Purity by HPLC: 99+%. MALDI-TOF MS m/z of $[M + H]^+$ calculated for $C_{35}H_{63}N_2O_5$: 591.5; Found: 591.8.

3.11 Synthesis of IAJDs with 3,5-symmetric Alkyl Chains

Scheme S11. Synthesis of IAJDs with 3,5-symmetric Alkyl Chains

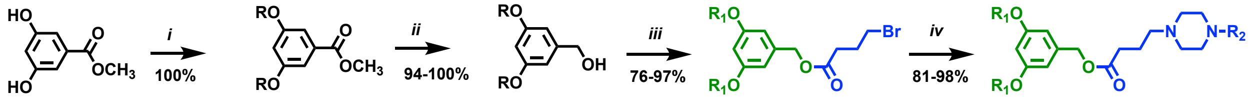

1 64a-h 65a-h 66a-h 67a-l

64-66a: R = $C_{11}H_{23}$
64-66b: R = $C_{10}H_{21}$
64-66c: R = $C_{15}H_{31}$
64-66d: R = $C_{17}H_{35}$
64-66e: R = C_9H_{19}
64-66f: R = $C_{13}H_{27}$
64-66g: R = $C_{14}H_{29}$
64-66h: R = $C_{16}H_{33}$

67a (IAJD73): $R_1 = C_{11}H_{21}$; $R_2 = CH_3$
67b (IAJD90): $R_1 = C_{10}H_{19}$; $R_2 = CH_3$
67c (IAJD263): $R_1 = C_{15}H_{31}$; $R_2 = CH_3$
67d (IAJD264): $R_1 = C_{15}H_{31}$; $R_2 = CH_2CH_2OH$
67e (IAJD265): $R_1 = C_{17}H_{35}$; $R_2 = CH_3$
67f (IAJD266): $R_1 = C_{17}H_{35}$; $R_2 = CH_2CH_2OH$
67g (IAJD267): $R_1 = C_9H_{19}$; $R_2 = CH_2CH_2OH$
67h (IAJD268): $R_1 = C_{11}H_{23}$; $R_2 = CH_2CH_2OH$
67i (IAJD269): $R_1 = C_{13}H_{27}$; $R_2 = CH_3$
67j (IAJD270): $R_1 = C_{13}H_{27}$; $R_2 = CH_2CH_2OH$
67k (IAJD271): $R_1 = C_{14}H_{29}$; $R_2 = CH_2CH_2OH$
67l (IAJD272): $R_1 = C_{16}H_{33}$; $R_2 = CH_2CH_2OH$

Reagents and conditions: (*i*) RBr/ROTs, K_2CO_3, DMF, 120 °C, 2 h; (*ii*) $LiAlH_4$, THF, 0–23 °C, 1 h; (*iii*) DCC, DPTS, DCM, 12 h; (*iv*) K_2CO_3, MeCN, 95 °C, 3 h.

General Synthetic Procedure for Compounds 64b, 64e, and 64g-h. Compounds **64b, 64e, and 64g-h** were synthesized according to a procedure elaborated and optimized by our laboratory.[4] Generally, compound **64b, 64e, and 64g-h** (1 equiv) and K_2CO_3 (2 equiv) were stirred in dry DMF. RBr (2.2 equiv) was added, and the mixture was stirred at 120 °C under N_2 atmosphere for 2 h. The reaction mixture was cooled to 23 °C. The reaction mixture was poured into ice/water and the white precipitates were filtered and collected. Then the precipitates were purified by recrystallization from acetone to give the title compound as a white solid. The synthesis and characterizations of compounds **64b, 64e, and 64g-h** were reported in the literature.[4]

General Synthetic Procedure for Compounds 65b, 65e, and 65g-h. Compounds **65b, 65e, and 65g-h** were synthesized according to a procedure reported by our group.[4] Generally, compounds **65b, 65e, and 65g-h** (1 equiv) were dissolved in dry THF, which was added dropwise to a slurry of $LiAlH_4$ (1 equiv) in dry THF at 0 °C under N_2 atmosphere. The resulted mixture was stirred at 23 °C for 1 h. The reaction was quenched by the successive addition of water, 15% NaOH aqueous solution and water. Then the mixture was filtered and dried over anhydrous $MgSO_4$. Filtration and evaporation of the solvent yielded the title

compound as a white solid or light-yellow oil. The synthesis and characterizations of compounds **65b, 65e, and 65g-h** were reported in the literature.[4]

General Synthetic Procedure for Compounds 66b and 66e. Compounds **66b and 66e** were synthesized according to a procedure reported by our group.[4] Generally, compounds **66b and 66e** (1 equiv), 4-bromobutyric acid (1.2 equiv) and DPTS (1.2 equiv) were dissolved in dry DCM. DCC (2 equiv) was added into the above mixture and stirred for 12h. Hexane was added to the reaction mixture, which was then vaccum filtered. The filtrate was concentrated and further purified by column chromatography on silica gel with a mobile phase of hexane/DCM = 1/1 (v/v) to afford the title compound as a transparent oil. The synthesis and characterizations of compounds **66b and 66e** were reported in the literature.[4]

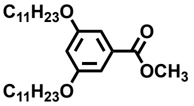

64a

Methyl 3,5-bis(undecyloxy)benzoate) (64a). Compound **64a** was synthesized from compound **1** (0.40 g, 2.38 mmol, 1 equiv), 1-Bromoundecane (1.23 g, 5.23 mmol, 2.2 equiv), K_2CO_3 (0.82 g, 5.95 mmol, 2.5 equiv) and DMF (20 mL) was heated to 120 °C and stirred under N_2 atmosphere for 2 h. The reaction mixture was cooled to 23 °C and poured into ice/water (150 mL). The resulted white precipitates in the solution were filtered and collected. Then the precipitates were recrystallized from minimum acetone to afford the title compound as a white solid (0.89 g, 78%). ^1H NMR (400 MHz, $CDCl_3$) δ 7.16 (br, 2 H, Ph*H*), 6.63 (t, 1 H, Ph*H*), 3.96 (t, 4 H, $PhOCH_2CH_2CH_2$-), 3.89 (s, 3 H, $PhCOOCH_3$), 1.78 (m, 4 H, $PhOCH_2CH_2CH_2$-), 1.46 (m, 4 H, $PhOCH_2CH_2CH_2$-), 1.26 (m, 28 H, $PhOCH_2CH_2CH_2(CH_2)_7CH_3$), 0.87 (t, 6 H, $PhO(CH_2)_{10}CH_3$). ^{13}C NMR (101 MHz, $CDCl_3$) δ 167.0, 160.2, 131.8, 107.6, 106.6, 77.2, 68.3, 64.1, 52.2, 31.9, 29.7, 29.7, 29.6, 29.6, 29.4, 29.2, 26.0, 25.8, 22.7, 14.1. Mp = 45 °C.

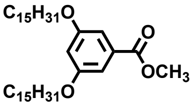

64c

Methyl 3,5-bis(pentadecyloxy)benzoate) (64c). A mixture of compound **1** (0.40 g, 2.38 mmol, 1 equiv), 1-Bromopentadecane (1.52 g, 5.23 mmol, 2.2 equiv), K_2CO_3 (0.82 g, 5.95 mmol, 2.5 equiv) following a procedure similar to that used for the synthesis of compound **64a**. The title compound was obtained as a white solid (1.30 g, 95%). ^1H NMR (400 MHz, $CDCl_3$) δ 7.16 (br, 2 H, Ph*H*), 6.63 (t, 1 H, Ph*H*), 3.96 (t, 4 H, $PhOCH_2CH_2CH_2$-), 3.89 (s, 3 H, $PhCOOCH_3$), 1.78 (m, 4 H, $PhOCH_2CH_2CH_2$-), 1.45 (m, 4 H, $PhOCH_2CH_2CH_2$-), 1.28 (m, 44 H, $PhOCH_2CH_2CH_2(CH_2)_{11}CH_3$), 0.88 (t, 6 H, $PhO(CH_2)_{14}CH_3$). ^{13}C

NMR (101 MHz, $CDCl_3$) δ 167.0, 160.2, 131.8, 107.6, 106.6, 77.2, 68.3, 52.2, 31.9, 29.7, 29.7, 29.7, 29.6, 29.6, 29.4, 29.2, 26.0, 22.7, 14.1. Mp = 65 °C.

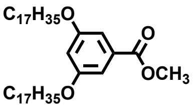

64d

Methyl 3,5-bis(heptadecyloxy)benzoate) (64d). Heptadecyl 4-methylbenzenesulfonate ($C_{17}H_{35}OTs$) was synthesized according to a literature procedure reported by our laboratory.[5] Compound **64d** was synthesized from compound **1** (0.40 g, 2.38 mmol, 1 equiv), $C_{17}H_{35}OTs$ (2.30 g, 5.23 mmol, 2.2 equiv), K_2CO_3 (0.82 g, 5.95 mmol, 2.5 equiv) following a procedure similar to that used for the synthesis of compound **64a**. The title compound was obtained as a white solid (1.33 g, 87%). ^1H NMR (400 MHz, $CDCl_3$) δ 7.16 (br, 2 H, Ph*H*), 6.63 (t, 1 H, Ph*H*), 3.96 (t, 4 H, $PhOCH_2CH_2CH_2$-), 3.89 (s, 3 H, $PhCOOCH_3$), 1.77 (m, 4 H, $PhOCH_2CH_2CH_2$-), 1.45 (m, 4 H, $PhOCH_2CH_2CH_2$-), 1.26 (m, 52 H, $PhOCH_2CH_2CH_2(CH_2)_{13}CH_3$), 0.88 (t, 6 H, $PhO(CH_2)_{16}CH_3$). ^{13}C NMR (101 MHz, $CDCl_3$) δ 167.0, 160.2, 131.8, 107.6, 106.6, 77.2, 68.3, 64.1, 52.2, 31.9, 29.7, 29.7, 29.6, 29.6, 29.4, 29.2, 26.0, 25.8, 22.7, 14.1. Mp = 70 °C.

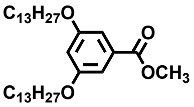

64f

Methyl 3,5-bis(tridecyloxy)benzoate) (64f). Tridecyl 4-methylbenzenesulfonate ($C_{13}H_{23}OTs$) was synthesized according to a literature procedure reported by our laboratory.[5] Compound **64f** was synthesized from compound **1** (1.28 g, 7.6 mmol, 1 equiv), $C_{13}H_{23}OTs$ (6.01 g, 16.9 mmol, 2.2 equiv), K_2CO_3 (3.14 g, 22.8 mmol, 3 equiv) following a procedure similar to that used for the synthesis of compound **64a**. The title compound was obtained as a white solid (2.83 g, 88%). ^1H NMR (400 MHz, $CDCl_3$) δ 7.16 (br, 2 H, Ph*H*), 6.63 (t, 1 H, Ph*H*), 3.96 (t, 4 H, $PhOCH_2CH_2CH_2$-), 3.89 (s, 3 H, $PhCOOCH_3$), 1.77 (m, 4 H, $PhOCH_2CH_2CH_2$-), 1.45 (m, 4 H, $PhOCH_2CH_2CH_2$-), 1.26 (m, 36 H, $PhOCH_2CH_2CH_2(CH_2)_9CH_3$), 0.88 (t, 6 H, $PhO(CH_2)_{12}CH_3$). ^{13}C NMR (101 MHz, $CDCl_3$) δ 167.1, 160.2,

131.8, 107.6, 106.6, 77.2, 68.3, 64.1, 52.2, 31.9, 29.7, 29.7, 29.6, 29.6, 29.4, 29.2, 26.1, 25.8, 22.7, 14.1. Mp = ? °C.

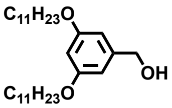

65a

(3,5-Bis(undecyloxy)phenyl)methanol (65a). Compound **64a** (0.83 g, 1.41 mmol, 1 equiv) was dissolved in 10 mL dry THF, which was added dropwise to a slurry of $LiAlH_4$ (54 mg, 1.41 mmol, 1 equiv) in dry THF (5 mL) at 0 °C under N_2 atmosphere. Afterwards, the resulted mixture was stirred at 23 °C for 1 h. Finally, the reaction was quenched by the successive addition of water (0.2 mL), 15% NaOH aqueous solution (0.2 mL) and water (1.0 mL). White precipitates were formed in the mixture and filtered out. The filtrate was dried over anhydrous $MgSO_4$ and filtered. The solvent was removed under reduced pressure to afford the title compound as a colorless oil (0.83 g, 99%). ^{1}H NMR (400 MHz, $CDCl_3$) δ 6.50 (br, 2 H, Ph*H*), 6.37 (t, 1 H, Ph*H*), 4.61 (s, 2 H, $PhCH_2OH$), 3.93 (t, 4 H, $PhOCH_2CH_2CH_2$-), 1.76 (m, 4 H, $PhOCH_2CH_2CH_2$-), 1.44 (m, 4 H, $PhOCH_2CH_2CH_2$-), 1.26 (m, 28 H, $PhOCH_2CH_2CH_2(CH_2)_7CH_3$), 0.88 (t, 6 H, $PhO(CH_2)_{10}CH_3$). ^{13}C NMR (101 MHz, $CDCl_3$) δ 160.6, 143.2, 105.1, 100.6, 77.2, 68.1, 65.5, 31.9, 30.3, 29.6, 29.6, 29.5, 29.4, 29.4, 29.3, 26.1, 22.7, 14.1.

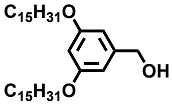

65c

(3,5-Bis(pentadecyloxy)phenyl)methanol (65c). Compound **65c** was synthesized from compound **64c** (0.89 g, 1.87 mmol, 1 equiv) and $LiAlH_4$ (71 mg, 1.87 mmol, 1 equiv) following a procedure similar to that used for the synthesis of compound **65a**. The title compound was obtained as a white solid (0.80 g, 100%). δ 6.50 (br, 2 H, Ph*H*), 6.38 (t, 1 H, Ph*H*), 4.61 (s, 2 H, $PhCH_2OH$), 3.93 (t, 4 H, $PhOCH_2CH_2CH_2$-), 1.76 (m, 4 H, $PhOCH_2CH_2CH_2$-), 1.43 (m, 4 H, $PhOCH_2CH_2CH_2$-), 1.26 (m, 44 H, $PhOCH_2CH_2CH_2(CH_2)_{11}CH_3$), 0.88 (t, 6 H, $PhO(CH_2)_{14}CH_3$). ^{13}C NMR (101 MHz, $CDCl_3$) δ 160.6, 143.2, 105.1, 100.6, 77.2, 68.1, 65.5, 31.9, 29.7, 29.7, 29.6, 29.6, 29.4, 29.4, 29.3, 26.1, 22.7, 14.1. Mp = 55 °C.

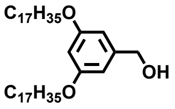

65d

(3,5-Bis(heptadecyloxy)phenyl)methanol (65d). Compound **65d** was synthesized from compound **64d** (0.66 g, 1.02 mmol, 1 equiv) and $LiAlH_4$ (38 mg, 1.02 mmol, 1 equiv) following a procedure similar to that used for the synthesis of compound **65a**. The title compound was obtained as a white solid (0.62 g, 100%). ^{1}H NMR (400 MHz, $CDCl_3$) δ 6.50 (br, 2 H, Ph*H*), 6.37 (t, 1 H, Ph*H*), 4.61 (s, 2 H, PhC*H*$_2$OH), 3.93 (t, 4 H, PhOC*H*$_2$CH$_2$CH$_2$-), 1.76 (m, 4 H, PhOCH$_2$C*H*$_2$CH$_2$-), 1.45 (m, 4 H, PhOCH$_2$CH$_2$C*H*$_2$-), 1.26 (m, 52 H, PhOCH$_2$CH$_2$CH$_2$(C*H*$_2$)$_{13}$CH$_3$), 0.88 (t, 6 H, PhO(CH$_2$)$_{16}$C*H*$_3$). ^{13}C NMR (101 MHz, $CDCl_3$) δ 160.6, 143.2, 105.1, 100.6, 77.2, 68.1, 65.5, 34.0, 32.9, 31.9, 29.7, 29.7, 29.6, 29.6, 29.6, 29.5, 29.4, 29.4, 29.3, 28.8, 28.2, 26.1, 22.7, 14.1. Mp = 64 °C.

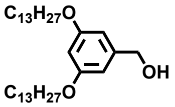

65f

(3,5-Bis(tridecyloxy)phenyl)methanol (65f). Compound **65f** was synthesized from compound **64f** (2.8 g, 5.02 mmol, 1 equiv) and $LiAlH_4$ (239 mg, 6.30 mmol, 1.2 equiv) following a procedure similar to that used for the synthesis of compound **65a**. The title compound was obtained as a colorless oil (2.46 g, 93%). ^{1}H NMR (400 MHz, $CDCl_3$) δ 6.50 (br, 2 H, Ph*H*), 6.38 (t, 1 H, Ph*H*), 4.61 (s, 2 H, PhC*H*$_2$OH), 3.93 (t, 4 H, PhOC*H*$_2$CH$_2$CH$_2$-), 1.76 (m, 4 H, PhOCH$_2$C*H*$_2$CH$_2$-), 1.44 (m, 4 H, PhOCH$_2$CH$_2$C*H*$_2$-), 1.26 (m, 36 H, PhOCH$_2$CH$_2$CH$_2$(C*H*$_2$)$_9$CH$_3$), 0.89 (t, 6 H, PhO(CH$_2$)$_{12}$C*H*$_3$). ^{13}C NMR (101 MHz, $CDCl_3$) δ 160.6, 143.2, 125.5, 105.1, 100.6, 77.2, 68.1, 65.5, 34.2, 31.9, 30.3, 29.7, 29.7, 29.7, 29.6, 29.6, 29.4, 29.4, 29.3, 26.1, 22.7, 21.2, 14.1.

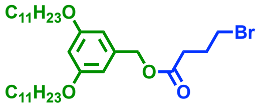

66a

3,5-Bis(undecyloxy)benzyl 4-bromobutanoate (66a). Compound **66a** was synthesized from compound **65a** (0.20 g, 0.45 mmol, 1 equiv), 4-bromobutyric acid (0.09 g, 0.54 mmol, 1.2 equiv), DPTS (0.16 g, 0.54 mmol, 1.2 equiv) were dissolved in 10 mL DCM. *N,N'*-Dicyclohexylcarbodiimide (DCC 0.18 g, 0.89 mmol, 2 equiv) was added into the above mixture. The reaction mixture was stirred at 23 °C for 12 h.

Afterwards, the resulted precipitates (urea) were filtered out from the reaction mixture. The filtrate was concentrated to around 3 mL and purified by column chromatography (SiO_2) with hexane/DCM = 1/1 as the mobile phase to give the title compound as a colorless oil (0.24 g, 90%). ^{1}H NMR (400 MHz, $CDCl_3$) δ 6.47 (br, 2 H, Ph*H*), 6.41 (t, 1 H, Ph*H*), 5.05 (s, 2 H, PhC*H*$_2$OCO), 3.93 (t, 4 H, PhOC*H*$_2$CH$_2$CH$_2$-), 3.46 (t, 2 H, -OCOCH$_2$CH$_2$C*H*$_2$Br), 2.56 (t, 2 H, -OCOC*H*$_2$CH$_2$CH$_2$Br), 2.20 (m, 2 H, -OCOCH$_2$C*H*$_2$CH$_2$Br), 1.77 (m, 4 H, PhOCH$_2$C*H*$_2$CH$_2$-), 1.45 (m, 4 H, PhOCH$_2$CH$_2$C*H*$_2$-), 1.28 (m, 28 H, PhOCH$_2$CH$_2$CH$_2$(C*H*$_2$)$_7$CH$_3$), 0.89 (t, 6 H, PhO(CH$_2$)$_{10}$C*H*$_3$). ^{13}C NMR (101 MHz, $CDCl_3$) δ 172.3, 160.5, 137.8, 106.4, 101.1, 77.2, 68.1, 66.4, 64.8, 32.7, 32.6, 32.5, 32.5, 31.9, 30.3, 29.7, 29.7, 29.6, 29.6, 29.6, 29.5, 29.4, 29.4, 29.3, 27.8, 27.8, 26.1, 25.9, 22.7, 14.1.

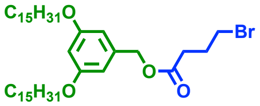

66c

3,5-Bis(pentadecyloxy)benzyl 4-bromobutanoate (66c). Compound **65c** (0.80 g, 1.41 mmol, 1 equiv), 4-bromobutyric acid (0.28 g, 1.69 mmol, 1.2 equiv), DPTS (0.50 g, 1.69 mmol, 1.2 equiv), and DCC (0.58 g, 2.82 mmol, 2 equiv) following a procedure similar to that used for the synthesis of compound **66a**. The title compound was obtained as a white solid (1.00 g, 100%). ^{1}H NMR (400 MHz, $CDCl_3$) δ 6.47 (br, 2 H, Ph*H*), 6.40 (t, 1 H, Ph*H*), 5.05 (s, 2 H, PhC*H*$_2$OCO), 3.93 (t, 4 H, PhOC*H*$_2$CH$_2$CH$_2$-), 3.47 (t, 2 H, -OCOCH$_2$CH$_2$C*H*$_2$Br), 2.57 (t, 2 H, -OCOC*H*$_2$CH$_2$CH$_2$Br), 2.20 (m, 2 H, -OCOCH$_2$C*H*$_2$CH$_2$Br), 1.77 (m, 4 H, PhOCH$_2$C*H*$_2$CH$_2$-), 1.45 (m, 4 H, PhOCH$_2$CH$_2$C*H*$_2$-), 1.28 (m, 44 H, PhOCH$_2$CH$_2$CH$_2$(C*H*$_2$)$_{11}$CH$_3$), 0.88 (t, 6 H, PhO(CH$_2$)$_{14}$C*H*$_3$). ^{13}C NMR (101 MHz, $CDCl_3$) δ 172.3, 160.5, 137.8, 106.4, 101.1, 68.1, 66.4, 32.6, 32.5, 31.9, 29.7, 29.7, 29.6, 29.6, 29.5, 29.4, 29.4, 29.3, 27.8, 26.1, 22.7, 14.1. Mp = 40 °C.

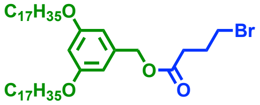

66d

3,5-Bis(heptadecyloxy)benzyl 4-bromobutanoate (66d). Compound **66d** was synthesized from compound **65d** (0.62 g, 1.02 mmol, 1 equiv), 4-bromobutyric acid (0.20 g, 1.22 mmol, 1.2 equiv), DPTS (0.36 g, 1.22 mmol, 1.2 equiv) and DCC (0.42 g, 2.04 mmol, 2 equiv) following a procedure similar to that used for the synthesis of compound **66a**. The title compound was obtained as a white solid (0.75 g, 96%). ^{1}H NMR (400 MHz, $CDCl_3$) δ 6.47 (br, 2 H, Ph*H*), 6.40 (t, 1 H, Ph*H*), 5.05 (s, 2 H, PhC*H*$_2$OCO),

3.93 (t, 4 H, PhOC*H*$_2$CH$_2$CH$_2$-), 3.47 (t, 2 H, -OCOCH$_2$CH$_2$C*H*$_2$Br), 2.57 (t, 2 H, -OCOC*H*$_2$CH$_2$CH$_2$Br), 2.20 (m, 2 H, -OCOCH$_2$C*H*$_2$CH$_2$Br), 1.77 (m, 4 H, PhOCH$_2$C*H*$_2$CH$_2$-), 1.45 (m, 4 H, PhOCH$_2$CH$_2$C*H*$_2$-), 1.28 (m, 52 H, PhOCH$_2$CH$_2$CH$_2$(C*H*$_2$)$_{13}$CH$_3$), 0.88 (t, 6 H, PhO(CH$_2$)$_{16}$C*H*$_3$). ^{13}C NMR (101 MHz, CDCl$_3$) δ 172.3, 160.5, 137.8, 106.4, 101.1, 77.2, 68.1, 66.4, 64.8, 32.7, 32.6, 32.5, 32.5, 31.9, 30.3, 29.7, 29.7, 29.6, 29.6, 29.6, 29.5, 29.4, 29.4, 29.3, 27.8, 27.8, 26.1, 25.9, 22.7, 14.1. Mp = 55 °C.

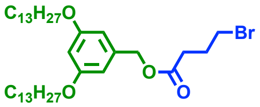

66f

3,5-Bis(tridecyloxy)benzyl 4-bromobutanoate (66f). Compound **66f** was synthesized from compound **65f** (1.00 g, 1.98 mmol, 1 equiv), 4-bromobutyric acid (0.36 g, 2.18 mmol, 1.1 equiv), DPTS (0.64 g, 2.18 mmol, 1.1 equiv) and DCC (0.82 g, 3.96 mmol, 2 equiv) following a procedure similar to that used for the synthesis of compound **66a**. The title compound was obtained as a colorless oil (1.2 g, 93%). ^{1}H NMR (400 MHz, CDCl$_3$) δ 6.46 (br, 2 H, Ph*H*), 6.40 (t, 1 H, Ph*H*), 5.04 (s, 2 H, PhC*H*$_2$OCO), 3.93 (t, 4 H, PhOC*H*$_2$CH$_2$CH$_2$-), 3.47 (t, 2 H, -OCOCH$_2$CH$_2$C*H*$_2$Br), 2.57 (t, 2 H, -OCOC*H*$_2$CH$_2$CH$_2$Br), 2.20 (m, 2 H, -OCOCH$_2$C*H*$_2$CH$_2$Br), 1.75 (m, 4 H, PhOCH$_2$C*H*$_2$CH$_2$-), 1.44 (m, 4 H, PhOCH$_2$CH$_2$C*H*$_2$-), 1.28 (m, 36 H, PhOCH$_2$CH$_2$CH$_2$(C*H*$_2$)$_9$CH$_3$), 0.88 (t, 6 H, PhO(CH$_2$)$_{12}$C*H*$_3$). ^{13}C NMR (101 MHz, CDCl$_3$) δ 172.3, 153.5, 138.1, 130.6, 106.3, 75.9, 71.2, 66.9, 40.6, 39.7, 32.6, 32.5, 30.5, 29.4, 29.3, 29.3, 29.1, 27.8, 23.8, 23.8, 23.2, 23.1, 14.2, 14.1, 11.1.

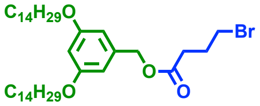

66g

3,5-Bis(tetradecyloxy)benzyl 4-bromobutanoate (66g). Compound **66g** was synthesized from compound **65g** (0.20 g, 0.36 mmol, 1 equiv), 4-bromobutyric acid (0.07 g, 0.43 mmol, 1.2 equiv), DPTS (0.13 g, 0.43 mmol, 1.2 equiv) and DCC (0.15 g, 0.71 mmol, 2 equiv) following a procedure similar to that used for the synthesis of compound **66a**. The title compound was obtained as a colorless oil (240 mg, 99%). ^{1}H NMR (400 MHz, CDCl$_3$) δ 6.46 (br, 2 H, Ph*H*), 6.40 (t, 1 H, Ph*H*), 5.04 (s, 2 H, PhC*H*$_2$OCO), 3.93 (t, 4 H, PhOC*H*$_2$CH$_2$CH$_2$-), 3.48 (t, 2 H, -OCOCH$_2$CH$_2$C*H*$_2$Br), 2.57 (t, 2 H, -OCOC*H*$_2$CH$_2$CH$_2$Br), 2.20 (m, 2 H, -OCOCH$_2$C*H*$_2$CH$_2$Br), 1.75 (m, 4 H, PhOCH$_2$C*H*$_2$CH$_2$-), 1.43 (m, 4 H, PhOCH$_2$CH$_2$C*H*$_2$-), 1.28 (m, 40 H, PhOCH$_2$CH$_2$CH$_2$(C*H*$_2$)$_{10}$CH$_3$), 0.88 (t, 6 H, PhO(CH$_2$)$_{13}$C*H*$_3$). ^{13}C NMR (101 MHz, CDCl$_3$)

δ 172.3, 160.5, 138.1, 130.6, 106.3, 75.9, 71.2, 66.9, 40.6, 39.7, 32.6, 32.5, 30.5, 29.4, 29.3, 29.3, 29.1, 27.8, 23.8, 23.8, 23.2, 23.1, 22.7, 14.2, 14.1.

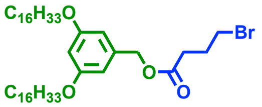

66h

3,5-Bis(hexadecyloxy)benzyl 4-bromobutanoate (66h). Compound **66h** was synthesized from compound **65h** (0.20 g, 0.34 mmol, 1 equiv), 4-bromobutyric acid (0.07 g, 0.41 mmol, 1.2 equiv), DPTS (0.12 g, 0.41 mmol, 1.2 equiv) and DCC (0.15 g, 0.68 mmol, 2 equiv) following a procedure similar to that used for the synthesis of compound **66a**. The title compound was obtained as a white solid (240 mg, 96%). ^{1}H NMR (400 MHz, $CDCl_3$) δ 6.46 (br, 2 H, Ph*H*), 6.41 (t, 1 H, Ph*H*), 5.05 (s, 2 H, PhC*H*$_2$OCO), 3.93 (t, 4 H, PhOC*H*$_2$CH$_2$CH$_2$-), 3.47 (t, 2 H, -OCOCH$_2$CH$_2$C*H*$_2$Br), 2.57 (t, 2 H, -OCOC*H*$_2$CH$_2$CH$_2$Br), 2.20 (m, 2 H, -OCOCH$_2$C*H*$_2$CH$_2$Br), 1.76 (m, 4 H, PhOCH$_2$C*H*$_2$CH$_2$-), 1.44 (m, 4 H, PhOCH$_2$CH$_2$C*H*$_2$-), 1.27 (m, 48 H, PhOCH$_2$CH$_2$CH$_2$(C*H*$_2$)$_{12}$CH$_3$), 0.88 (t, 6 H, PhO(CH$_2$)$_{15}$C*H*$_3$). ^{13}C NMR (101 MHz, $CDCl_3$) δ 172.3, 160.5, 137.8, 106.4, 101.1, 77.2, 68.1, 66.4, 32.6, 32.5, 31.9, 29.7, 29.7, 29.6, 29.6, 29.4, 29.4, 29.3, 27.8, 26.1, 22.7, 14.1. Mp = 43 °C.

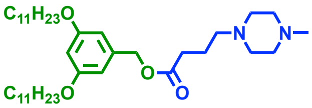

67a (IAJD73)

3,5-Bis(undecyloxy)benzyl 4-(4-methylpiperazin-1-yl)butanoate (67a, IAJD73). Compound **67a** was synthesized from compound **66a** (390 mg, 0.65 mmol, 1 equiv), 1-methylpiperazine (68 mg, 0.68 mmol, 1.05 equiv) and K_2CO_3 (94 mg, 0.68 mmol, 1.05 equiv) in 20ml MeCN was heated to 95 °C and stirred for 3 h. The reaction mixture was cooled to 23 °C and MeCN was removed under reduced pressure. Then water (20 mL) was added, and the resulted mixture was extracted with DCM (20 mL) for three times. The organic phases were collected and dried over anhydrous $MgSO_4$. After filtration, the obtained filtrate was concentrated to around 3 mL and purified by column chromatography (SiO_2) with DCM/MeOH = 30/1 and 15/1 as the eluent. Then the obtained product was dissolved in DCM (20 mL), which was washed by $NaHCO_3$ solution (2%, 20 mL). The aqueous phase was extracted by DCM (20 mL) for another two times. The organic phases were combined and dried over anhydrous $MgSO_4$. Filtration and evaporation of the solvent yielded the title compound as a colorless oil (0.36 g, 90%). ^{1}H NMR (400 MHz, $CDCl_3$) δ 6.44

(br, 2 H, Ph*H*), 6.38 (t, 1 H, Ph*H*), 5.01 (s, 2 H, PhC*H*$_2$-), 3.90 (t, 4 H, PhOC*H*$_2$-), 2.58–2.21 (m, 14 H, -N(C*H*$_2$C*H*$_2$)$_2$N-, -NC*H*$_2$CH$_2$C*H*$_2$COO- and -C*H*$_2$CH$_2$OH), 1.82 (m, 2 H, -OCOCH$_2$C*H*$_2$CH$_2$-), 1.74 (m, 4 H, PhOCH$_2$C*H*$_2$-), 1.43 (m, 4 H, PhOCH$_2$CH$_2$C*H*$_2$-), 1.27 (br, 28 H, PhOCH$_2$CH$_2$CH$_2$(C*H*$_2$)$_7$CH$_3$), 0.86 (t, 6 H, -CH$_2$CH$_2$C*H*$_3$). ^{13}C NMR (101 MHz, CDCl$_3$) δ 173.2, 160.4, 138.1, 106.4, 100.9, 77.3, 68.0, 66.1, 57.6, 55.2, 53.1, 46.1, 32.2, 31.9, 29.6, 29.6, 29.4, 29.3, 29.3, 26.1, 22.7, 22.2, 14.1. Purity by HPLC: 99+%. MALDI-TOF MS m/z of $[M + H]^+$ calculated for $C_{38}H_{69}N_2O_4$: 617.6; Found: 617.2.

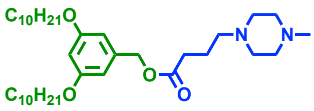

67b (IAJD90)

3,5-Bis(decyloxy)benzyl 4-(4-methylpiperazin-1-yl)butanoate (67b, IAJD90). Compound **67b** was synthesized from compound **66b** (300 mg, 0.53 mmol, 1 equiv), 1-methylpiperazine (73 mg, 0.56 mmol, 1.05 equiv) and K_2CO_3 (77 mg, 0.56 mmol, 1.05 equiv) following a procedure similar to that used for the synthesis of compound **67a**. The title compound was obtained as a colorless oil (0.28 g, 90%). ^{1}H NMR (400 MHz, CDCl$_3$) δ 6.45 (br, 2 H, Ph*H*), 6.38 (t, 1 H, Ph*H*), 5.01 (s, 2 H, PhC*H*$_2$-), 3.91 (t, 4 H, PhOC*H*$_2$-), 2.65–2.21 (m, 15 H, -N(C*H*$_2$C*H*$_2$)$_2$N-, -NC*H*$_2$CH$_2$C*H*$_2$COO- and -NC*H*$_3$), 1.83 (m, 2 H, -OCOCH$_2$C*H*$_2$CH$_2$-), 1.74 (m, 4 H, PhOCH$_2$C*H*$_2$-), 1.42 (m, 4 H, PhOCH$_2$CH$_2$C*H*$_2$-), 1.28 (br, 24 H, PhOCH$_2$CH$_2$CH$_2$(C*H*$_2$)$_6$CH$_3$), 0.87 (t, 6 H, -CH$_2$CH$_2$C*H*$_3$). ^{13}C NMR (101 MHz, CDCl$_3$) δ 173.3, 160.4, 138.1, 106.4, 100.9, 77.3, 68.1, 66.1, 57.6, 55.2, 53.1, 46.1, 32.3, 31.9, 29.6, 29.6, 29.4, 29.3, 29.3, 26.1, 22.7, 22.2, 14.1. Purity by HPLC: 99+%. MALDI-TOF MS m/z of $[M + H]^+$ calculated for $C_{36}H_{65}N_2O_4$: 589.6; Found: 590.4.

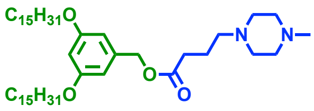

67c (IAJD263)

3,5-Bis(pentadecyloxy)benzyl 4-(4-methylpiperazin-1-yl)butanoate (67c, IAJD263). Compound **67c** was synthesized from compound **66c** (500 mg, 0.70 mmol, 1 equiv), 1-methylpiperazine (85 mg, 0.85 mmol, 1.2 equiv) and K_2CO_3 (146 mg, 1.06 mmol, 1.5 equiv) following a procedure similar to that used for the synthesis of compound **67a**. The title compound was obtained as a colorless oil (0.50 g, 97%). ^{1}H NMR (400 MHz, CDCl$_3$) δ 6.45 (br, 2 H, Ph*H*), 6.39 (t, 1 H, Ph*H*), 5.02 (s, 2 H, PhC*H*$_2$-), 3.91 (t, 4 H, PhOC*H*$_2$-), 2.59–2.25 (m, 15 H, -N(C*H*$_2$C*H*$_2$)$_2$N-, -NC*H*$_2$CH$_2$C*H*$_2$COO- and -NC*H*$_3$), 1.83 (m, 2 H, -OCOCH$_2$C*H*$_2$CH$_2$-), 1.75 (m, 4 H, PhOCH$_2$C*H*$_2$-), 1.42 (m, 4 H, PhOCH$_2$CH$_2$C*H*$_2$-), 1.28 (br, 44 H,

PhOCH$_2$CH$_2$CH$_2$(C*H*$_2$)$_{11}$CH$_3$), 0.87 (t, 6 H, -CH$_2$CH$_2$C*H*$_3$). ^{13}C NMR (101 MHz, CDCl$_3$) δ 173.3, 160.5, 138.1, 106.4, 100.9, 77.2, 68.1, 66.1, 57.5, 54.9, 52.7, 45.8, 32.2, 31.9, 29.7, 29.7, 29.6, 29.6, 29.4, 29.4, 29.3, 26.1, 22.7, 22.1, 14.1.. Purity by HPLC: 99+%. MALDI-TOF MS m/z of [M + H]$^+$ calculated for C$_{46}$H$_{85}$N$_2$O$_4$: 729.7; Found: 730.3.

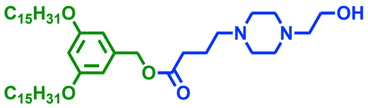

67d (IAJD264)

3,5-Bis(pentadecyloxy)benzyl 4-(4-(2-hydroxyethyl)piperazin-1-yl)butanoate (67d, IAJD264). Compound **67d** was synthesized from compound **66c** (500 mg, 0.70 mmol, 1 equiv), 1-(2-hydroxyethyl)piperazine (110 mg, 0.85 mmol, 1.2 equiv) and K$_2$CO$_3$ (146 mg, 1.06 mmol, 1.5 equiv) following a procedure similar to that used for the synthesis of compound **67a**. The title compound was obtained as a colorless oil (0.50 g, 94%). ^{1}H NMR (400 MHz, CDCl$_3$) δ 6.46 (br, 2 H, Ph*H*), 6.39 (t, 1 H, Ph*H*), 5.02 (s, 2 H, PhC*H*$_2$-), 3.91 (t, 4 H, PhOC*H*$_2$-), 3.61 (t, 2H, -CH$_2$C*H*$_2$OH), 2.60–2.30 (m, 14 H, -N(C*H*$_2$C*H*$_2$)$_2$N-, -NC*H*$_2$CH$_2$C*H*$_2$COO- and -C*H*$_2$CH$_2$OH), 1.85 (m, 2 H, -OCOCH$_2$C*H*$_2$CH$_2$-), 1.74 (m, 4 H, PhOCH$_2$C*H*$_2$-), 1.43 (m, 4 H, PhOCH$_2$CH$_2$C*H*$_2$-), 1.28 (br, 44 H, PhOCH$_2$CH$_2$CH$_2$(C*H*$_2$)$_{11}$CH$_3$), 0.87 (t, 6 H, -CH$_2$CH$_2$C*H*$_3$). ^{13}C NMR (101 MHz, CDCl$_3$) δ 173.3, 160.5, 138.1, 106.4, 100.9, 77.2, 68.1, 66.2, 59.3, 57.6, 57.5, 52.9, 52.8, 32.2, 31.9, 29.7, 29.7, 29.6, 29.6, 29.4, 29.4, 29.3, 26.1, 22.7, 22.1, 14.1. Purity by HPLC: 99+%. MALDI-TOF MS m/z of [M + H]$^+$ calculated for C$_{47}$H$_{87}$N$_2$O$_5$: 759.9; Found: 759.6.

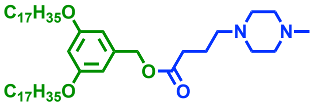

67e (IAJD265)

3,5-Bis(pentadecyloxy)benzyl 4-(4-methylpiperazin-1-yl)butanoate (67e, IAJD265). Compound **67e** was synthesized from compound **66d** (300 mg, 0.39 mmol, 1 equiv), 1-methylpiperazine (47 mg, 0.47 mmol, 1.2 equiv) and K$_2$CO$_3$ (81 mg, 0.59 mmol, 1.5 equiv) following a procedure similar to that used for the synthesis of compound **67a**. The title compound was obtained as a colorless oil (0.50 g, 97%). ^{1}H NMR (400 MHz, CDCl$_3$) δ 6.46 (br, 2 H, Ph*H*), 6.39 (t, 1 H, Ph*H*), 5.02 (s, 2 H, PhC*H*$_2$-), 3.92 (t, 4 H, PhOC*H*$_2$-), 2.63–2.25 (m, 15 H, -N(C*H*$_2$C*H*$_2$)$_2$N-, -NC*H*$_2$CH$_2$C*H*$_2$COO- and -NC*H*$_3$), 1.83 (m, 2 H, -OCOCH$_2$C*H*$_2$CH$_2$-), 1.73 (m, 4 H, PhOCH$_2$C*H*$_2$-), 1.42 (m, 4 H, PhOCH$_2$CH$_2$C*H*$_2$-), 1.26 (br, 52 H, PhOCH$_2$CH$_2$CH$_2$(C*H*$_2$)$_{13}$CH$_3$), 0.88 (t, 6 H, -CH$_2$CH$_2$C*H*$_3$). ^{13}C NMR (101 MHz, CDCl$_3$) δ 173.3, 160.5,

138.1, 106.4, 100.9, 77.2, 68.1, 66.2, 57.6, 55.2, 53.1, 46.1, 32.3, 31.9, 29.7, 29.7, 29.6, 29.6, 29.4, 29.4, 29.3, 26.1, 22.7, 22.2, 14.1. Purity by HPLC: 99+%. MALDI-TOF MS m/z of $[M + H]^+$ calculated for $C_{50}H_{93}N_2O_4$: 785.8; Found: 786.0.

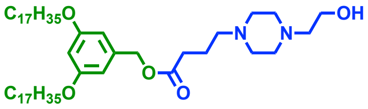

67f (IAJD266)

3,5-Bis(pentadecyloxy)benzyl 4-(4-(2-hydroxyethyl)piperazin-1-yl)butanoate (67f, IAJD266). Compound **67f** was synthesized from compound **66d** (300 mg, 0.39 mmol, 1 equiv), 1-(2-hydroxyethyl)piperazine (62 mg, 0.47 mmol, 1.2 equiv) and K_2CO_3 (81 mg, 0.59 mmol, 1.5 equiv) following a procedure similar to that used for the synthesis of compound **67a**. The title compound was obtained as a colorless oil (0.50 g, 94%). ^{1}H NMR (400 MHz, $CDCl_3$) δ 6.46 (br, 2 H, Ph*H*), 6.39 (t, 1 H, Ph*H*), 5.01 (s, 2 H, PhC*H*2-), 3.89 (t, 4 H, PhOC*H*2-), 3.61 (t, 2H, -CH2C*H*2OH), 2.62–2.31 (m, 14 H, -N(C*H*2C*H*2)2N-, -NC*H*2CH2C*H*2COO- and -C*H*2CH2OH), 1.82 (m, 2 H, -OCOCH2C*H*2CH2-), 1.72 (m, 4 H, PhOCH2C*H*2-), 1.40 (m, 4 H, PhOCH2CH2C*H*2-), 1.28 (br, 52 H, PhOCH2CH2CH2(C*H*2)13CH3), 0.86 (t, 6 H, -CH2CH2C*H*3). ^{13}C NMR (101 MHz, $CDCl_3$) δ 173.3, 160.5, 138.1, 106.4, 100.9, 77.2, 68.1, 66.2, 59.3, 57.6, 57.5, 53.0, 52.8, 32.2, 31.9, 31.8, 29.5, 29.4, 29.4, 29.3, 29.2, 26.1, 22.7, 22.1, 14.1. Purity by HPLC: 99+%. MALDI-TOF MS m/z of $[M + H]^+$ calculated for $C_{51}H_{95}N_2O_5$: 815.8; Found: 815.7.

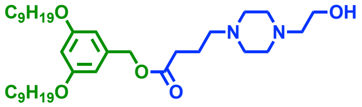

67g (IAJD267)

3,5-Bis(noncyloxy)benzyl 4-(4-(2-hydroxyethyl)piperazin-1-yl)butanoate (67g, IAJD267). Compound **67g** was synthesized from compound **66e** (280 mg, 0.52 mmol, 1 equiv), 1-(2-hydroxyethyl)piperazine (74 mg, 0.57 mmol, 1.1 equiv) and K_2CO_3 (84 mg, 0.57 mmol, 1.1 equiv) following a procedure similar to that used for the synthesis of compound **67a**. The title compound was obtained as a colorless oil (0.27 g, 88%). ^{1}H NMR (400 MHz, $CDCl_3$) δ 6.46 (br, 2 H, Ph*H*), 6.39 (t, 1 H, Ph*H*), 5.03 (s, 2 H, PhC*H*2-), 3.92 (t, 4 H, PhOC*H*2-), 3.61 (t, 2H, -CH2C*H*2OH), 2.62–2.31 (m, 14 H, -N(C*H*2C*H*2)2N-, -NC*H*2CH2C*H*2COO- and -C*H*2CH2OH), 1.84 (m, 2 H, -OCOCH2C*H*2CH2-), 1.75 (m, 4 H, PhOCH2C*H*2-), 1.43 (m, 4 H, PhOCH2CH2C*H*2-), 1.28 (br, 20 H, PhOCH2CH2CH2(C*H*2)5CH3), 0.89 (t, 6 H, -CH2CH2C*H*3). ^{13}C NMR (101 MHz, $CDCl_3$) δ 173.3, 160.5, 138.1, 106.4, 100.9, 68.1, 66.2, 59.2,

57.7, 57.6, 53.2, 52.8, 32.3, 31.9, 29.5, 29.4, 29.3, 26.1, 22.7, 22.2, 14.1. Purity by HPLC: 99+%. MALDI-TOF MS m/z of $[M + H]^+$ calculated for $C_{35}H_{63}N_2O_5$: 591.5; Found: 592.5.

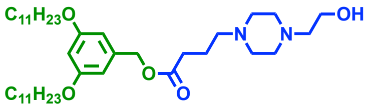

67h (IAJD268)

3,5-Bis(undecyloxy)benzyl 4-(4-(2-hydroxyethyl)piperazin-1-yl)butanoate (67h, IAJD268). Compound **67h** was synthesized from compound **66a** (240 mg, 0.40 mmol, 1 equiv), 1-(2-hydroxyethyl)piperazine (79 mg, 0.60 mmol, 1.5 equiv) and K_2CO_3 (67 mg, 0.48 mmol, 1.2 equiv) following a procedure similar to that used for the synthesis of compound **67a**. The title compound was obtained as a colorless oil (0.25 g, 96%). ^{1}H NMR (400 MHz, $CDCl_3$) δ 6.44 (br, 2 H, Ph*H*), 6.37 (t, 1 H, Ph*H*), 5.01 (s, 2 H, $PhC\mathit{H}_2$-), 3.90 (t, 4 H, $PhOC\mathit{H}_2$-), 3.60 (t, 2H, $-CH_2C\mathit{H}_2OH$), 2.67 – 2.26 (m, 14 H, $-N(C\mathit{H}_2C\mathit{H}_2)_2N$-, $-NC\mathit{H}_2CH_2C\mathit{H}_2COO$- and $-C\mathit{H}_2CH_2OH$), 1.86 (m, 2 H, $-OCOCH_2C\mathit{H}_2CH_2$-), 1.73 (m, 4 H, $PhOCH_2C\mathit{H}_2$-), 1.44 (m, 4 H, $PhOCH_2CH_2C\mathit{H}_2$-), 1.27 (br, 28 H, $PhOCH_2CH_2CH_2(C\mathit{H}_2)_7CH_3$), 0.86 (t, 6 H, $-CH_2CH_2C\mathit{H}_3$). ^{13}C NMR (101 MHz, $CDCl_3$) δ 173.3, 160.5, 138.1, 106.4, 100.9, 77.2, 68.1, 66.2, 59.3, 57.6, 57.5, 53.0, 52.8, 32.2, 31.9, 31.8, 29.5, 29.4, 29.4, 29.3, 29.2, 26.1, 22.7, 22.1, 14.1. Purity by HPLC: 99+%. MALDI-TOF MS m/z of $[M + H]^+$ calculated for $C_{39}H_{71}N_2O_5$: 647.6; Found: 648.1.

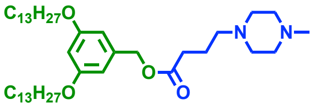

67i (IAJD269)

3,5-Bis(tridecyloxy)benzyl 4-(4-methylpiperazin-1-yl)butanoate (67i, IAJD269). Compound **67i** was synthesized from compound **66f** (200 mg, 0.31 mmol, 1 equiv), 1-methylpiperazine (46 mg, 0.46 mmol, 1.5 equiv) and K_2CO_3 (51 mg, 0.37 mmol, 1.2 equiv) following a procedure similar to that used for the synthesis of compound **67a**. The title compound was obtained as a colorless oil (0.50 g, 97%). ^{1}H NMR (400 MHz, $CDCl_3$) δ 6.44 (br, 2 H, Ph*H*), 6.37 (t, 1 H, Ph*H*), 5.01 (s, 2 H, $PhC\mathit{H}_2$-), 3.90 (t, 4 H, $PhOC\mathit{H}_2$-), 2.61–2.24 (m, 15 H, $-N(C\mathit{H}_2C\mathit{H}_2)_2N$-, $-NC\mathit{H}_2CH_2C\mathit{H}_2COO$- and $-NC\mathit{H}_3$), 1.82 (m, 2 H, $-OCOCH_2C\mathit{H}_2CH_2$-), 1.78 (m, 4 H, $PhOCH_2C\mathit{H}_2$-), 1.42 (m, 4 H, $PhOCH_2CH_2C\mathit{H}_2$-), 1.28 (br, 36 H, $PhOCH_2CH_2CH_2(C\mathit{H}_2)_9CH_3$), 0.86 (t, 6 H, $-CH_2CH_2C\mathit{H}_3$). ^{13}C NMR (101 MHz, $CDCl_3$) δ 173.2, 160.4, 138.1, 106.4, 100.9, 68.1, 66.1, 57.4, 55.0, 53.4, 52.7, 45.8, 32.2, 31.9, 29.7, 29.7, 29.6, 29.6, 29.6, 29.4, 29.4, 29.3, 26.1, 22.7, 22.1, 14.1. Purity by HPLC: 99+%. MALDI-TOF MS m/z of $[M + H]^+$ calculated for $C_{42}H_{77}N_2O_4$: 673.7; Found: 672.8.

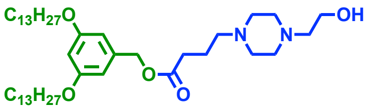

67j (IAJD270)

3,5-Bis(tridecyloxy)benzyl 4-(4-(2-hydroxyethyl)piperazin-1-yl)butanoate (67j, IAJD270). Compound **67j** was synthesized from compound **66f** (200 mg, 0.31 mmol, 1 equiv), 1-(2-hydroxyethyl)piperazine (60 mg, 0.46 mmol, 1.5 equiv) and K_2CO_3 (51 mg, 0.37 mmol, 1.2 equiv) following a procedure similar to that used for the synthesis of compound **67a**. The title compound was obtained as a colorless oil (0.50 g, 94%). ^1H NMR (400 MHz, $CDCl_3$) δ 6.46 (br, 2 H, Ph*H*), 6.39 (t, 1 H, Ph*H*), 5.02 (s, 2 H, PhC*H*$_2$-), 3.91 (t, 4 H, PhOC*H*$_2$-), 3.62 (t, 2H, -CH$_2$C*H*$_2$OH), 2.68–2.31 (m, 14 H, -N(C*H*$_2$C*H*$_2$)$_2$N-, -NC*H*$_2$CH$_2$C*H*$_2$COO- and -C*H*$_2$CH$_2$OH), 1.84 (m, 2 H, -OCOCH$_2$C*H*$_2$CH$_2$-), 1.75 (m, 4 H, PhOCH$_2$C*H*$_2$-), 1.43 (m, 4 H, PhOCH$_2$CH$_2$C*H*$_2$-), 1.27 (br, 36 H, PhOCH$_2$CH$_2$CH$_2$(C*H*$_2$)$_9$CH$_3$), 0.88 (t, 6 H, -CH$_2$CH$_2$C*H*$_3$). ^{13}C NMR (101 MHz, $CDCl_3$) δ 173.3, 160.5, 138.1, 106.4, 105.1, 100.9, 77.2, 68.1, 66.2, 59.3, 57.5, 57.4, 53.4, 52.7, 52.7, 32.2, 31.9, 29.7, 29.7, 29.6, 29.6, 29.4, 29.4, 29.3, 26.1, 22.7, 22.0, 14.1. Purity by HPLC: 99+%. MALDI-TOF MS m/z of $[M + H]^+$ calculated for $C_{43}H_{79}N_2O_5$: 703.7; Found: 703.8.

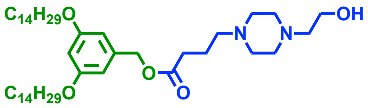

67k (IAJD271)

3,5-Bis(tetradecyloxy)benzyl 4-(4-(2-hydroxyethyl)piperazin-1-yl)butanoate (67k, IAJD271). Compound **67k** was synthesized from compound **66g** (280 mg, 0.52 mmol, 1 equiv), 1-(2-hydroxyethyl)piperazine (69 mg, 0.53 mmol, 1.5 equiv) and K_2CO_3 (59 mg, 0.42 mmol, 1.2 equiv) following a procedure similar to that used for the synthesis of compound **67a**. The title compound was obtained as a colorless oil (0.47 g, 93%). ^1H NMR (400 MHz, $CDCl_3$) δ 6.44 (br, 2 H, Ph*H*), 6.37 (t, 1 H, Ph*H*), 5.01 (s, 2 H, PhC*H*$_2$-), 3.90 (t, 4 H, PhOC*H*$_2$-), 3.60 (t, 2H, -CH$_2$C*H*$_2$OH), 2.61–2.24 (m, 14 H, -N(C*H*$_2$C*H*$_2$)$_2$N-, -NC*H*$_2$CH$_2$C*H*$_2$COO- and -C*H*$_2$CH$_2$OH), 1.83 (m, 2 H, -OCOCH$_2$C*H*$_2$CH$_2$-), 1.75 (m, 4 H, PhOCH$_2$C*H*$_2$-), 1.42 (m, 4 H, PhOCH$_2$CH$_2$C*H*$_2$-), 1.28 (br, 40 H, PhOCH$_2$CH$_2$CH$_2$(C*H*$_2$)$_5$CH$_3$), 0.86 (t, 6 H, -CH$_2$CH$_2$C*H*$_3$). ^{13}C NMR (101 MHz, $CDCl_3$) δ 173.2, 160.4, 138.1, 106.4, 100.9, 68.1, 66.1, 57.5, 53.4, 52.9, 52.9, 32.2, 31.9, 29.7, 29.7, 29.7, 29.6, 29.6, 29.4, 29.4, 29.3, 26.1, 22.7, 22.1, 14.1. Purity by HPLC: 99+%. MALDI-TOF MS m/z of $[M + H]^+$ calculated for $C_{45}H_{83}N_2O_5$: 731.7; Found: 732.1.

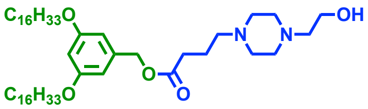

67l (IAJD272)

3,5-Bis(hexadecyloxy)benzyl 4-(4-(2-hydroxyethyl)piperazin-1-yl)butanoate (67l, IAJD272). Compound **67l** was synthesized from compound **66h** (240 mg, 0.33 mmol, 1 equiv), 1-(2-hydroxyethyl)piperazine (64 mg, 0.49 mmol, 1.5 equiv) and K_2CO_3 (53 mg, 0.39 mmol, 1.2 equiv) following a procedure similar to that used for the synthesis of compound **67a**. The title compound was obtained as a colorless oil (0.24 g, 94%). ^{1}H NMR (400 MHz, $CDCl_3$) δ 6.45 (br, 2 H, Ph*H*), 6.38 (t, 1 H, Ph*H*), 5.02 (s, 2 H, PhC*H*$_2$-), 3.91 (t, 4 H, PhOC*H*$_2$-), 3.61 (t, 2H, -CH$_2$C*H*$_2$OH), 2.67–2.29 (m, 14 H, -N(C*H*$_2$C*H*$_2$)$_2$N-, -NC*H*$_2$CH$_2$C*H*$_2$COO- and -C*H*$_2$CH$_2$OH), 1.84 (m, 2 H, -OCOCH$_2$C*H*$_2$CH$_2$-), 1.75 (m, 4 H, PhOCH$_2$C*H*$_2$-), 1.43 (m, 4 H, PhOCH$_2$CH$_2$C*H*$_2$-), 1.26 (br, 44 H, PhOCH$_2$CH$_2$CH$_2$(C*H*$_2$)$_{11}$CH$_3$), 0.87 (t, 6 H, -CH$_2$CH$_2$C*H*$_3$). ^{13}C NMR (101 MHz, $CDCl_3$) δ 173.2, 160.5, 138.1, 106.4, 100.9, 68.1, 66.2, 59.4, 57.6, 57.5, 52.8, 32.2, 31.9, 29.7, 29.7, 29.6, 29.6, 29.4, 29.4, 29.3, 26.1, 22.7, 22.0, 14.1. Purity by HPLC: 99+%. MALDI-TOF MS m/z of $[M + H]^+$ calculated for $C_{49}H_{91}N_2O_5$: 787.8; Found: 787.2.

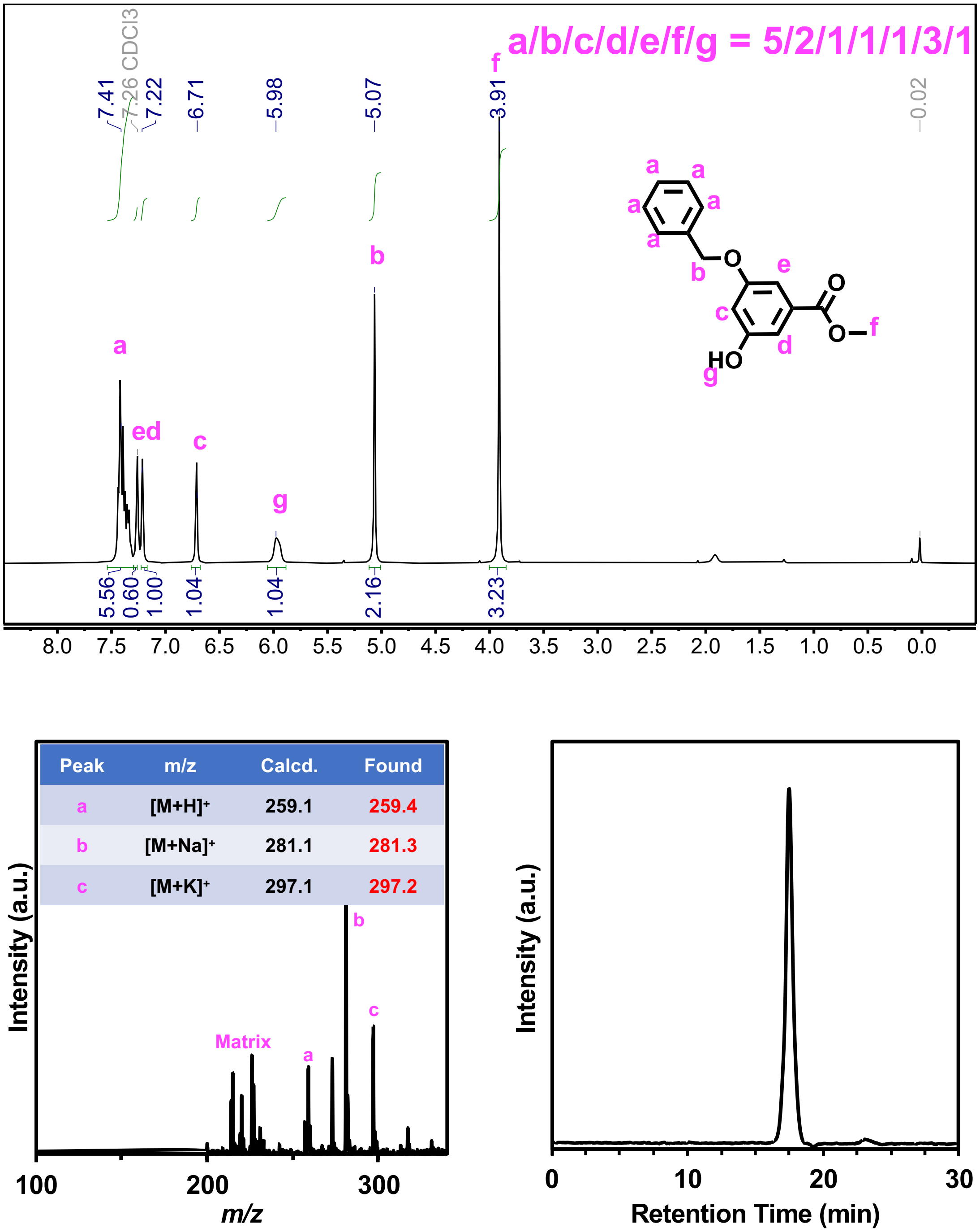

Peak	m/z	Calcd.	Found
a	[M+H]+	259.1	259.4
b	[M+Na]+	281.1	281.3
c	[M+K]+	297.1	297.2

Figure S1. ^{1}H NMR spectrum (top), MALDI-TOF MS spectrum (bottom left) and HPLC trace (bottom right) of Monoprotected Benzyl Ether of Methyl 3,5-Dihydroxybenzoate **2**.

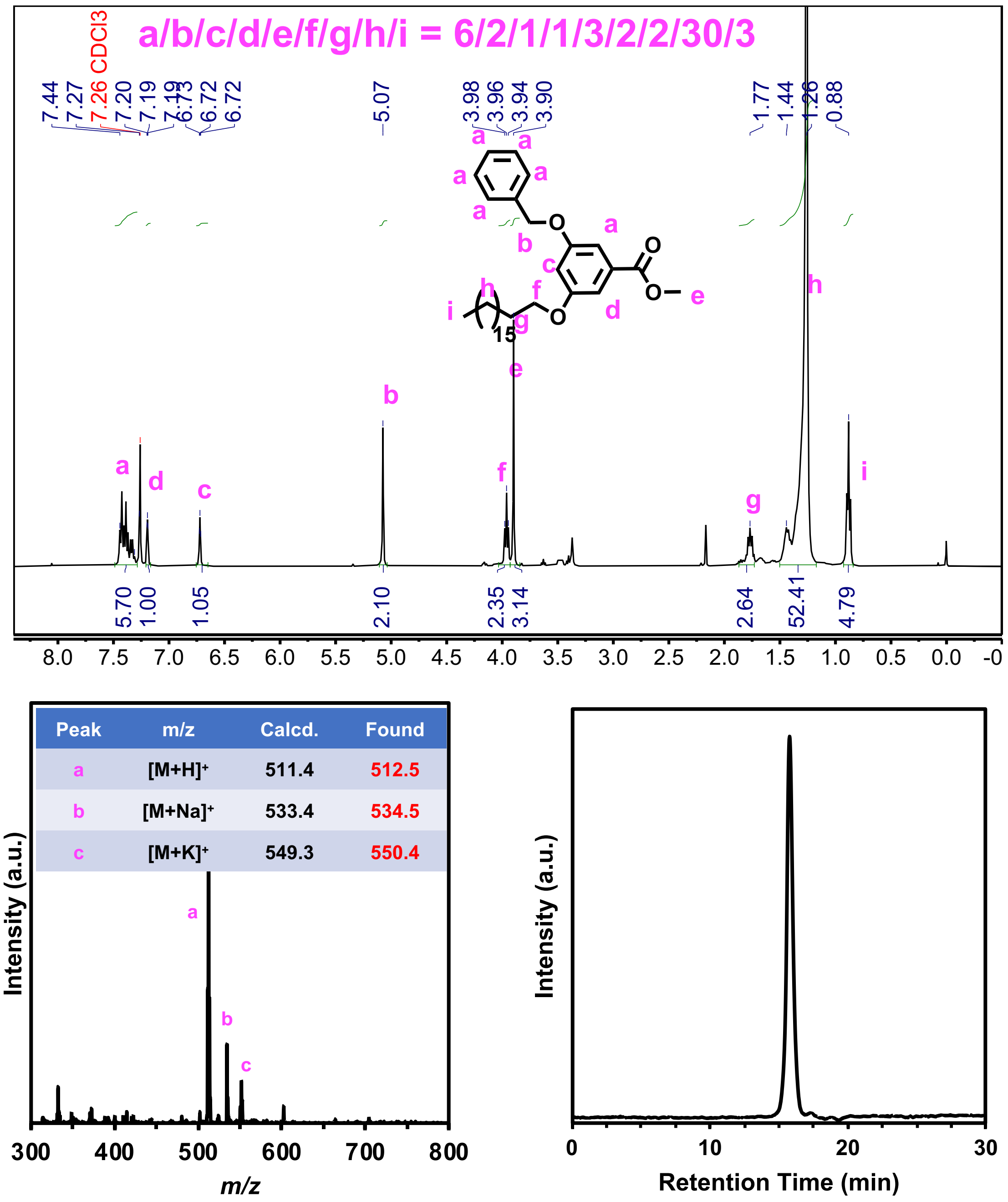

Peak	m/z	Calcd.	Found
a	$[M+H]^+$	511.4	512.5
b	$[M+Na]^+$	533.4	534.5
c	$[M+K]^+$	549.3	550.4

Figure S2. ^{1}H NMR spectrum (top), MALDI-TOF MS spectrum (bottom left) and HPLC trace (bottom right) of Methyl 3-(benzyloxy)-5-(octadecyloxy)benzoate, **33**.

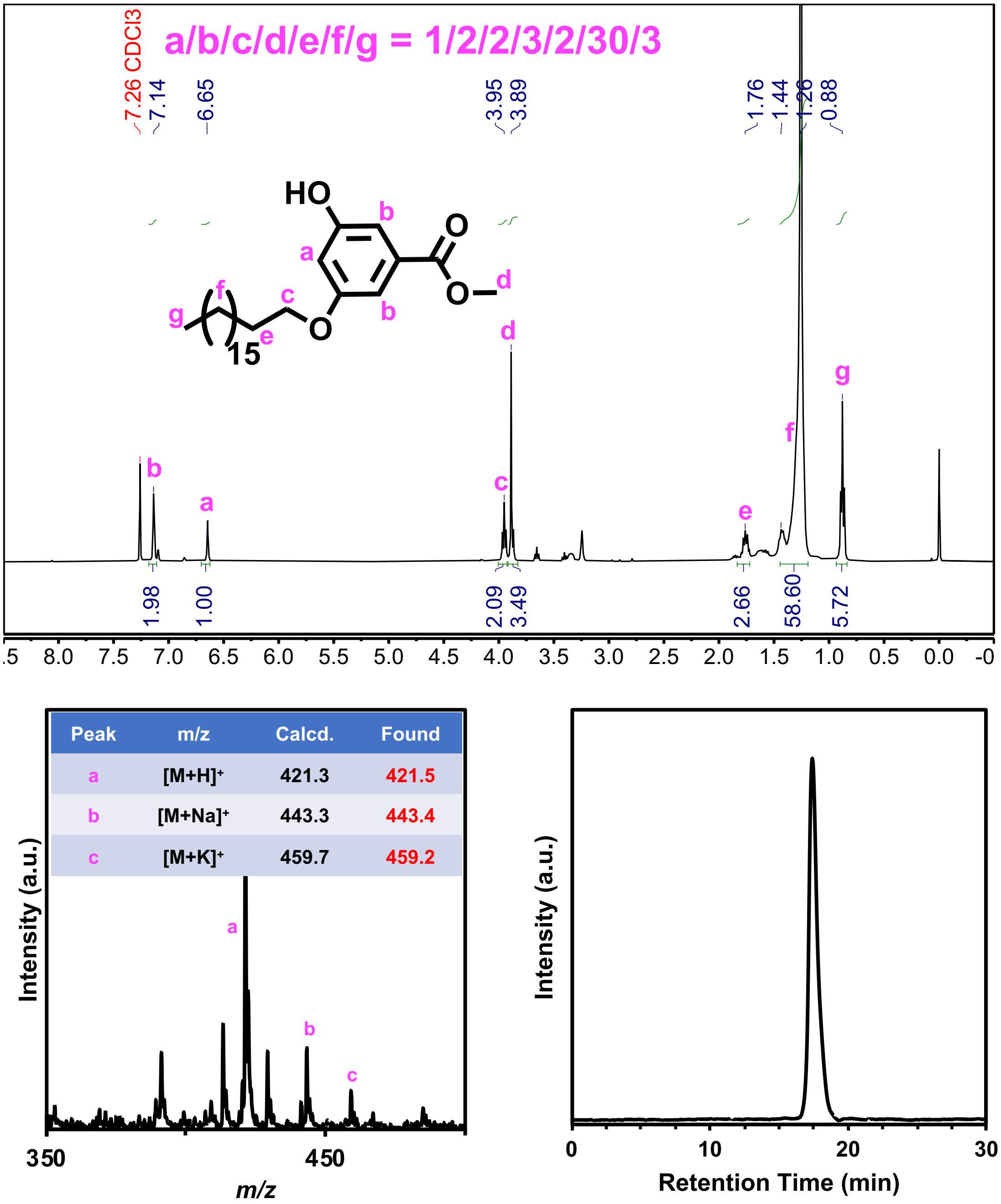

Peak	m/z	Calcd.	Found
a	$[M+H]^+$	421.3	421.5
b	$[M+Na]^+$	443.3	443.4
c	$[M+K]^+$	459.7	459.2

Figure S3. ^{1}H NMR spectrum (top), MALDI-TOF MS spectrum (bottom left) and HPLC trace (bottom right) of Methyl 3-hydroxy-5-(octadecyloxy)benzoate, **34**.

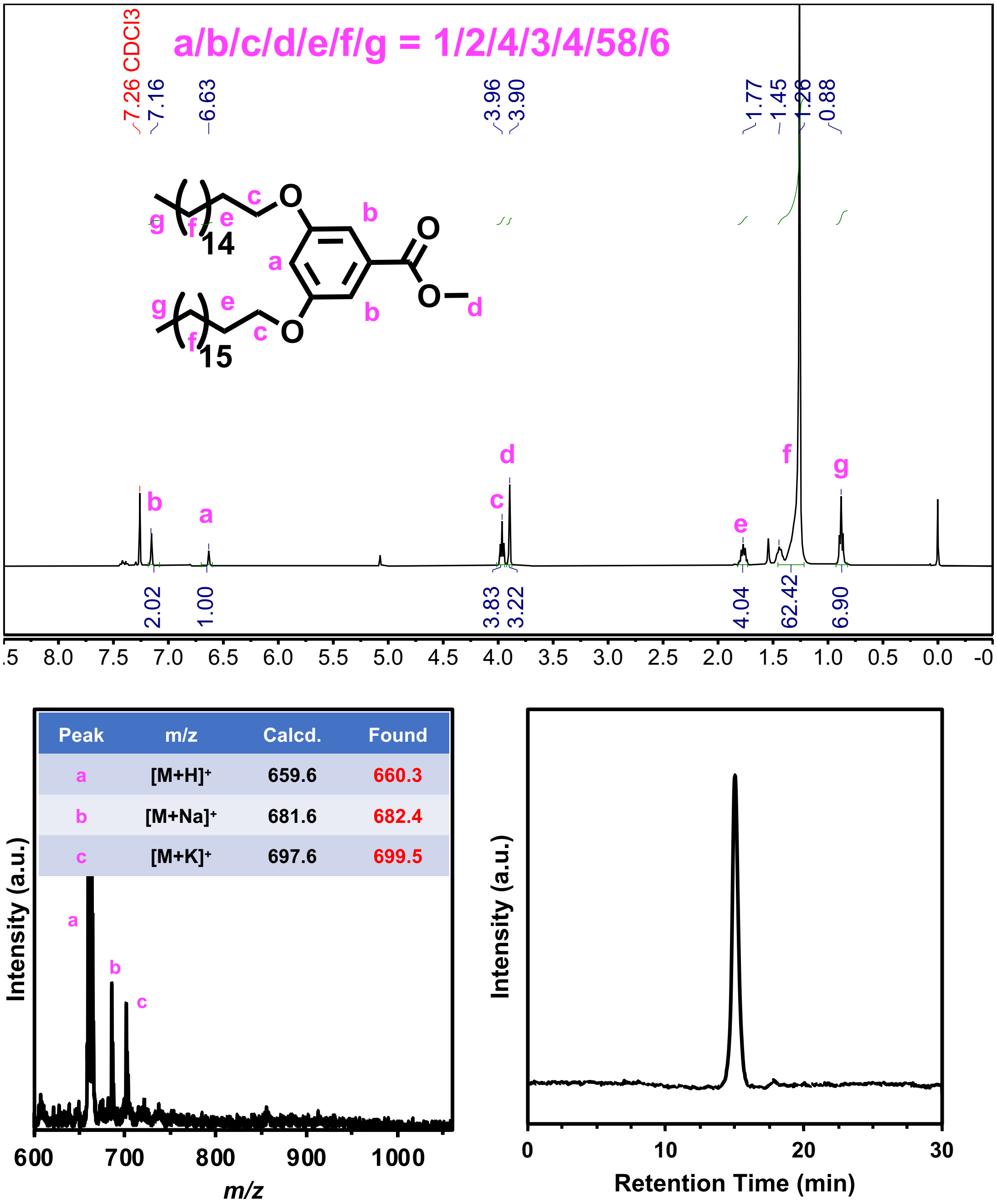

Peak	m/z	Calcd.	Found
a	[M+H]+	659.6	660.3
b	[M+Na]+	681.6	682.4
c	[M+K]+	697.6	699.5

Figure S4. ^{1}H NMR spectra (top), MALDI-TOF MS spectra (bottom left) and HPLC trace (bottom right) of Methyl 3-(heptadecyloxy)-5-(octadecyloxy)benzoate, **35d**.

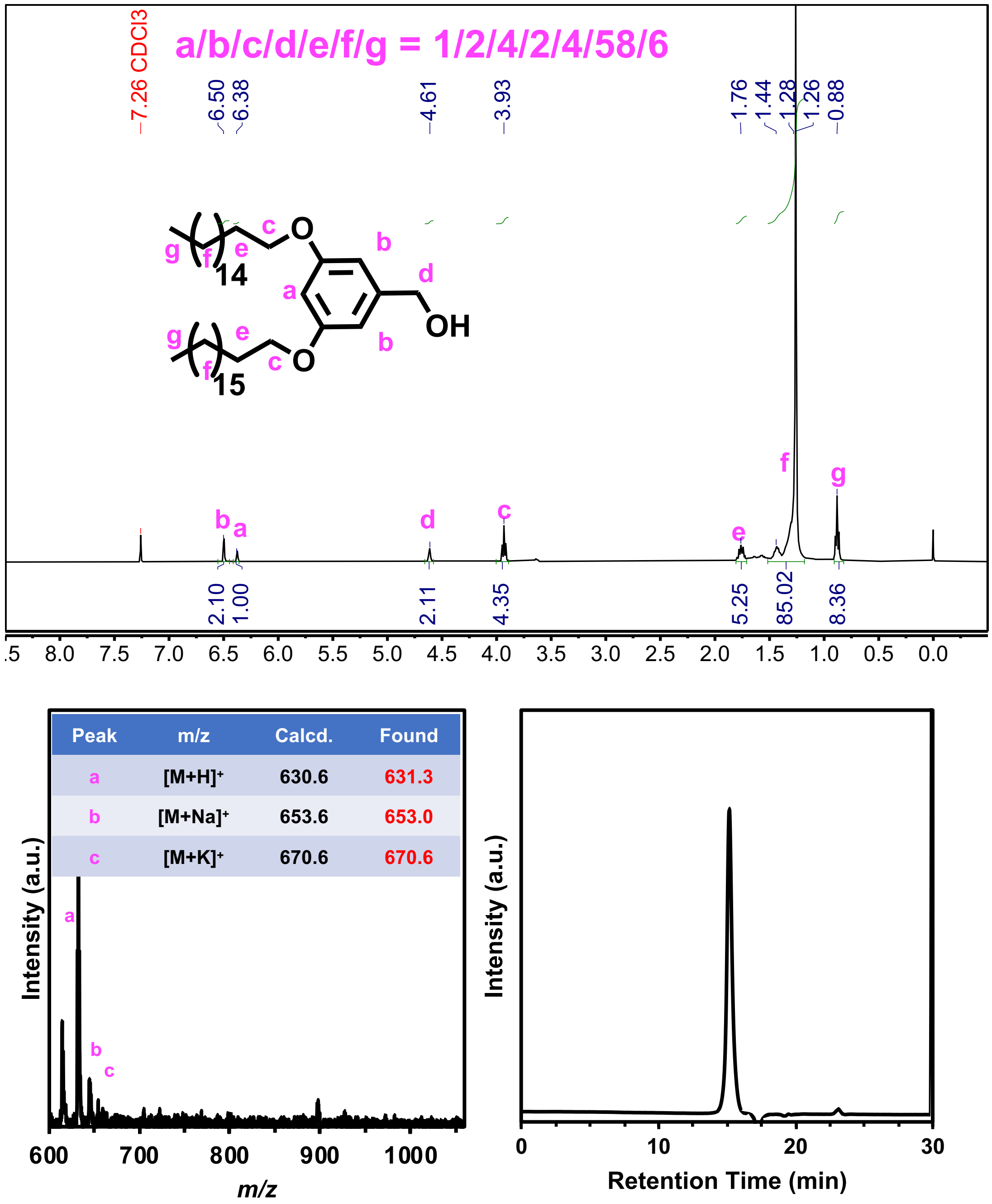

Peak	m/z	Calcd.	Found
a	$[M+H]^+$	630.6	631.3
b	$[M+Na]^+$	653.6	653.0
c	$[M+K]^+$	670.6	670.6

Figure S5. ^{1}H NMR spectrum (top), MALDI-TOF MS spectrum (bottom left) and HPLC trace (bottom right) of (3-(Heptadecyloxy)-5-(octadecyloxy)phenyl)methanol, **36d**.

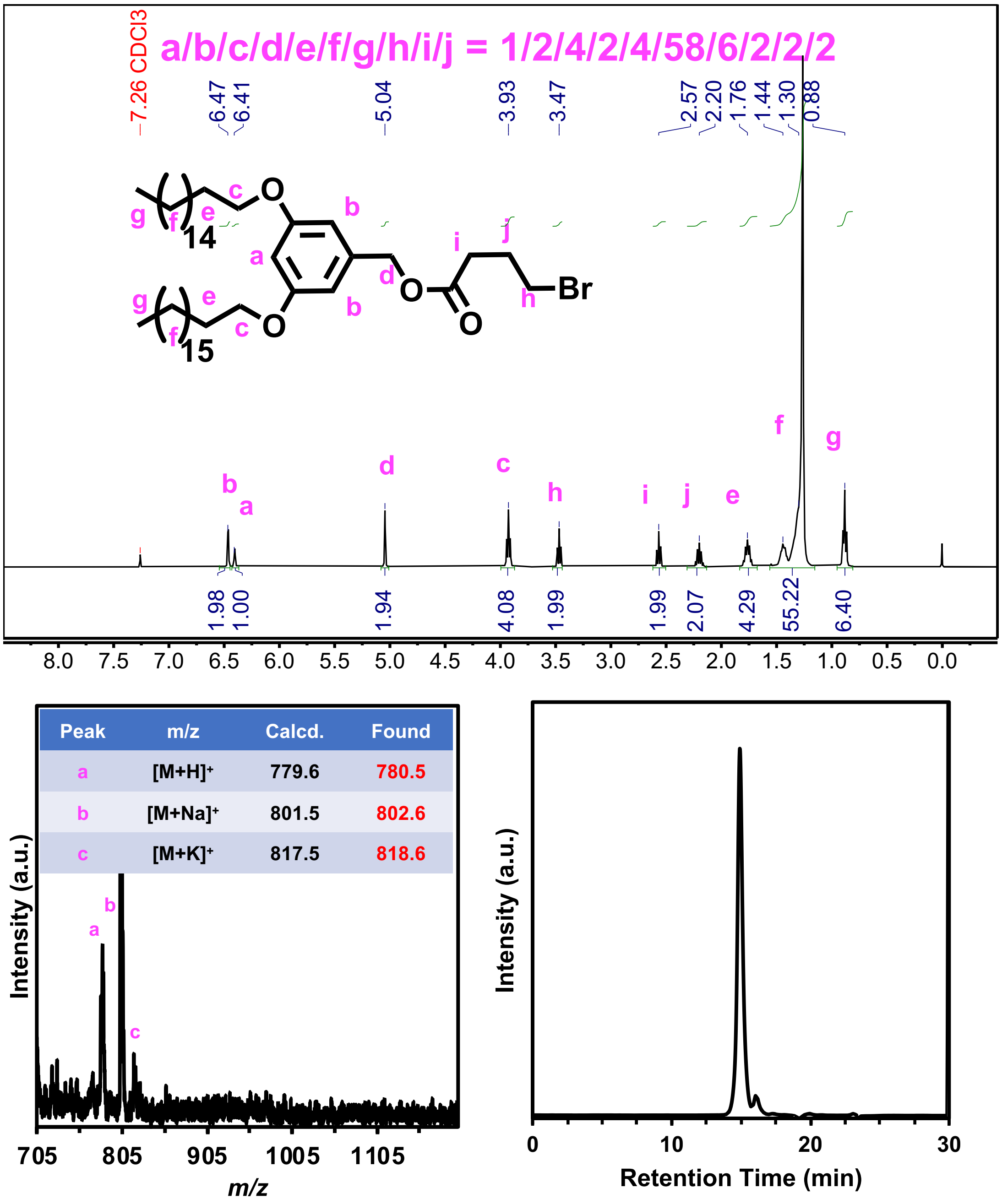

Peak	m/z	Calcd.	Found
a	$[M+H]^+$	779.6	780.5
b	$[M+Na]^+$	801.5	802.6
c	$[M+K]^+$	817.5	818.6

Figure S6. ^{1}H NMR spectrum (top), MALDI-TOF MS spectrum (bottom left) and HPLC trace (bottom right) of 3-(Heptadecyloxy)-5-(octadecyloxy)benzyl 4-bromobutanoate, **37d**.

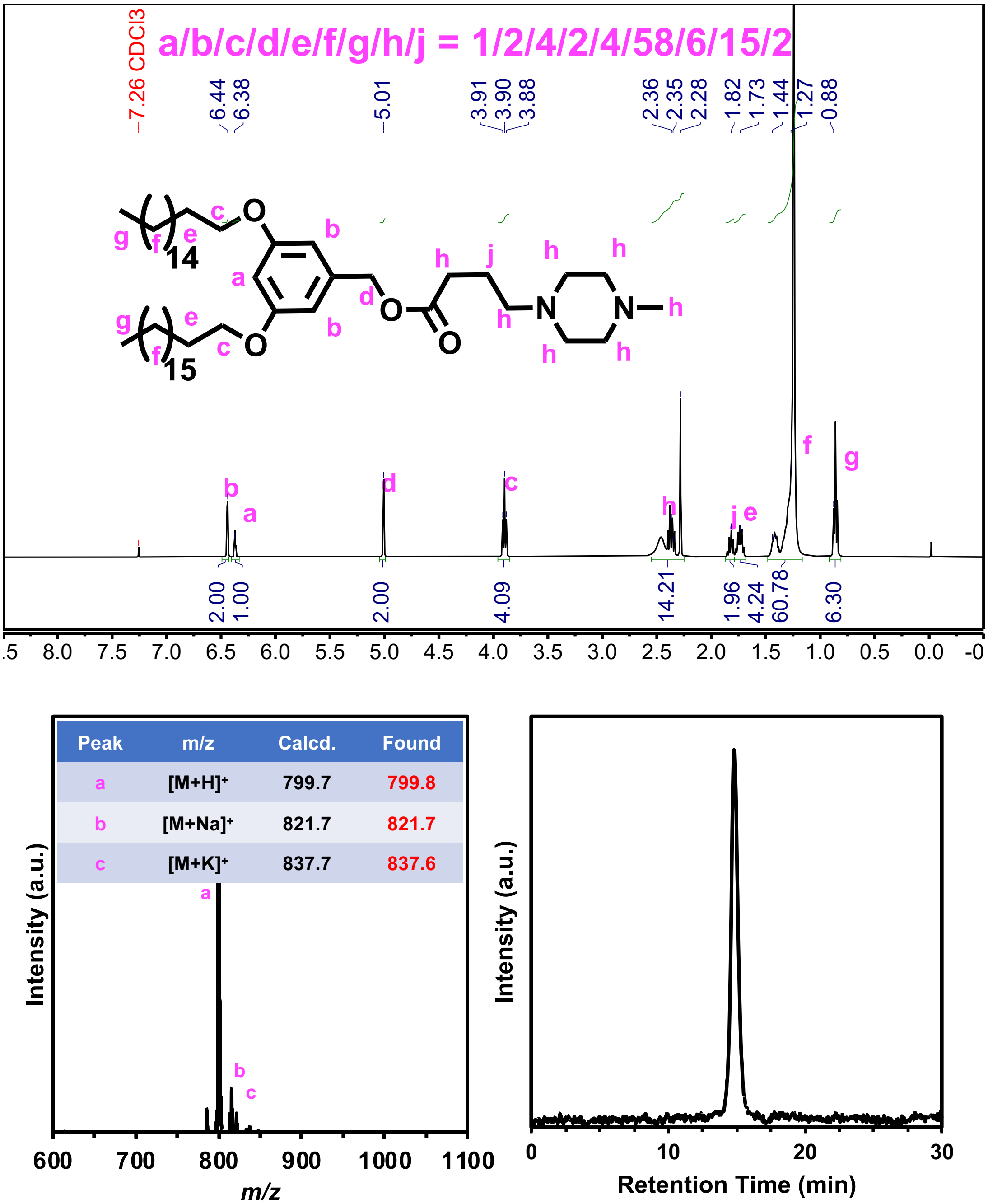

Peak	m/z	Calcd.	Found
a	[M+H]+	799.7	799.8
b	[M+Na]+	821.7	821.7
c	[M+K]+	837.7	837.6

Figure S7. ^{1}H NMR spectrum (top), MALDI-TOF MS spectrum (bottom left) and HPLC trace (bottom right) of 3-(heptadecyloxy)-5-(octadecyloxy)benzyl 4-(4-methylpiperazin-1-yl)butanoate, **38g**, IAJD242.

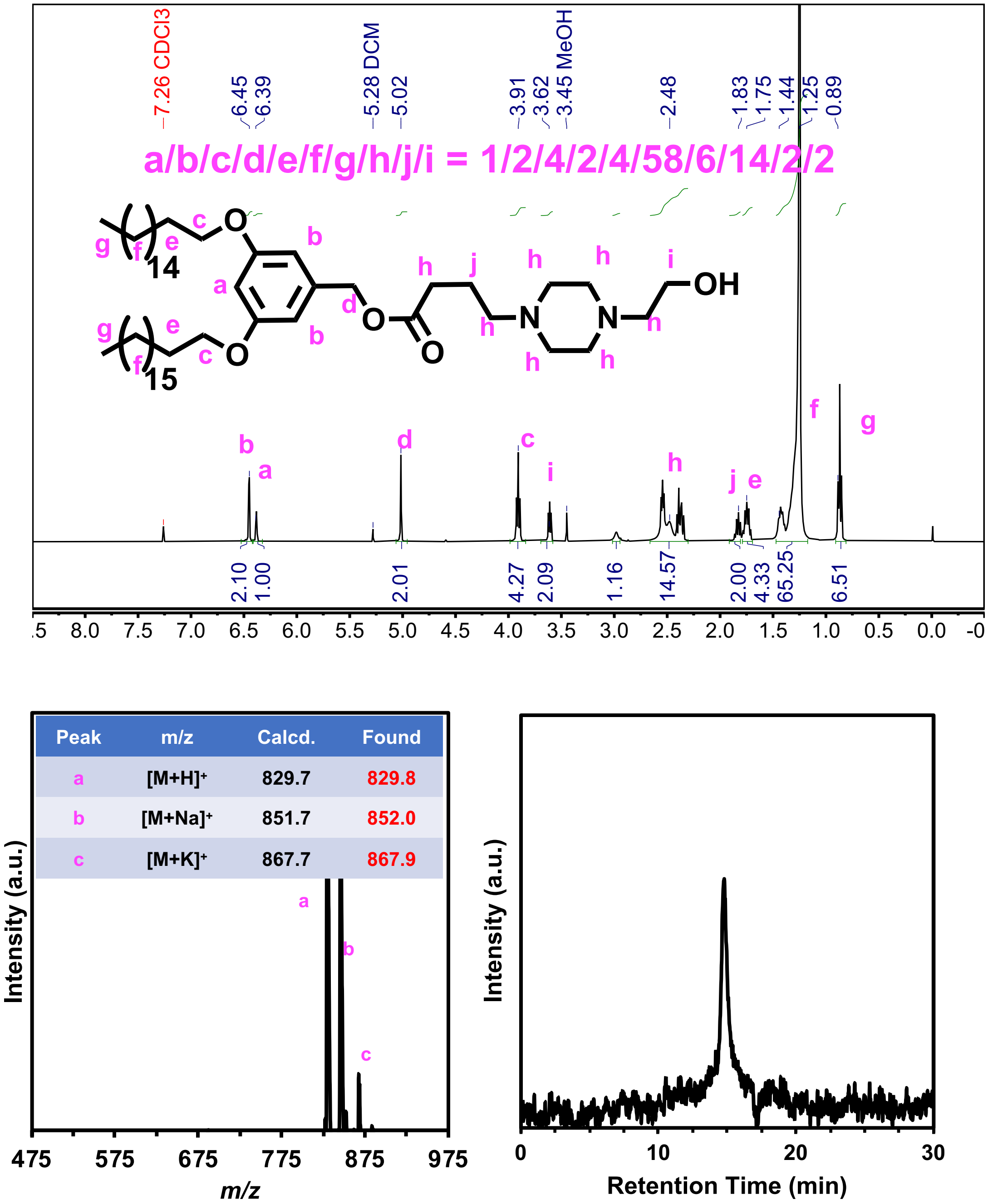

Peak	m/z	Calcd.	Found
a	[M+H]+	829.7	829.8
b	[M+Na]+	851.7	852.0
c	[M+K]+	867.7	867.9

Figure S8. ^{1}H NMR spectrum (top), MALDI-TOF MS spectrum (bottom left) and HPLC trace (bottom right) of 3-(Heptadecyloxy)-5-(octadecyloxy)benzyl 4-(4-(2-hydroxyethyl)piperazin-1-yl)butanoate, **38h**, IAJD243.

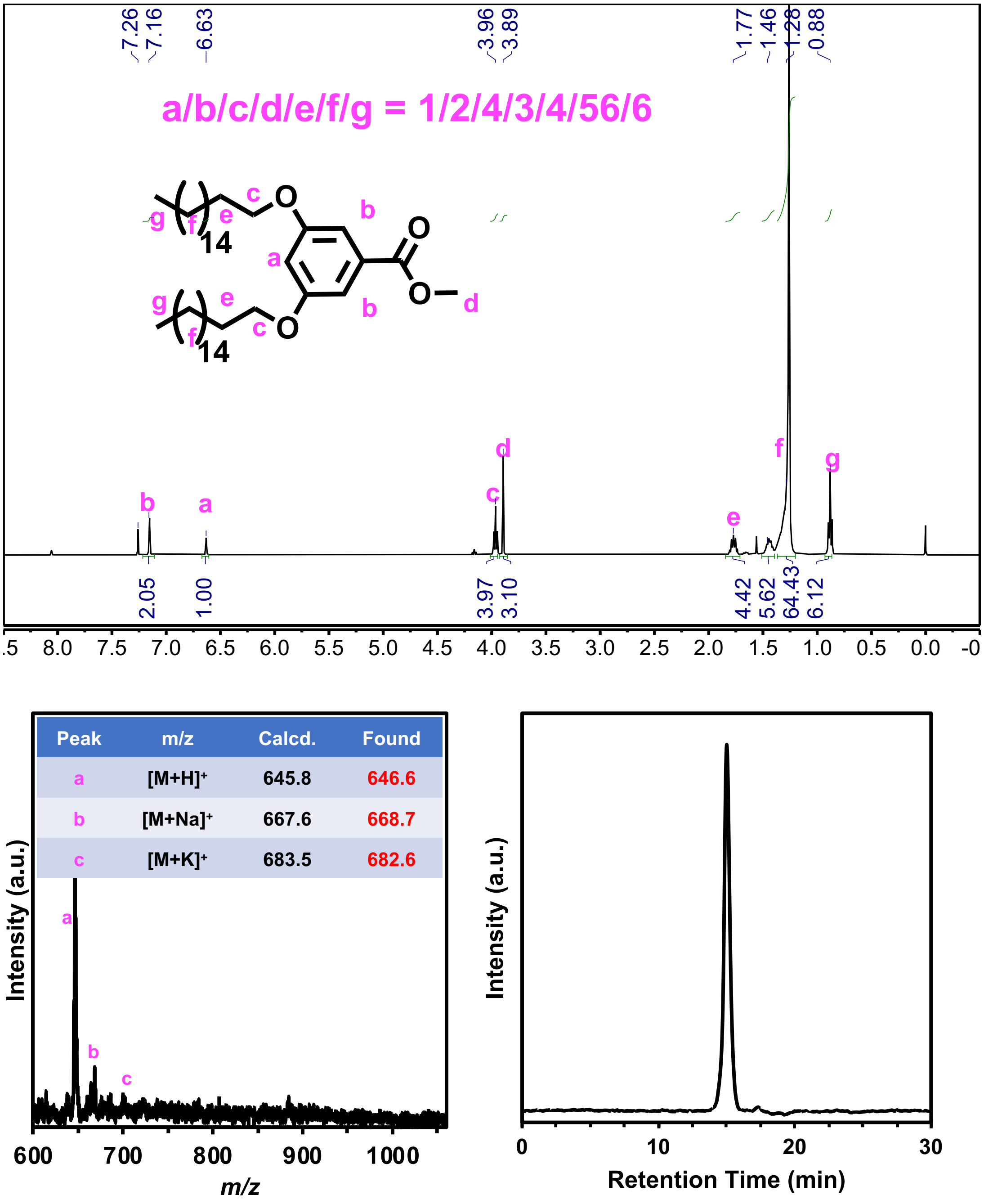

Peak	m/z	Calcd.	Found
a	$[M+H]^+$	645.8	646.6
b	$[M+Na]^+$	667.6	668.7
c	$[M+K]^+$	683.5	682.6

Figure S9. ^{1}H NMR spectrum (top), MALDI-TOF MS spectrum (bottom left) and HPLC trace (bottom right) of methyl 3,5-bis(heptadecyloxy)benzoate), **64d**.

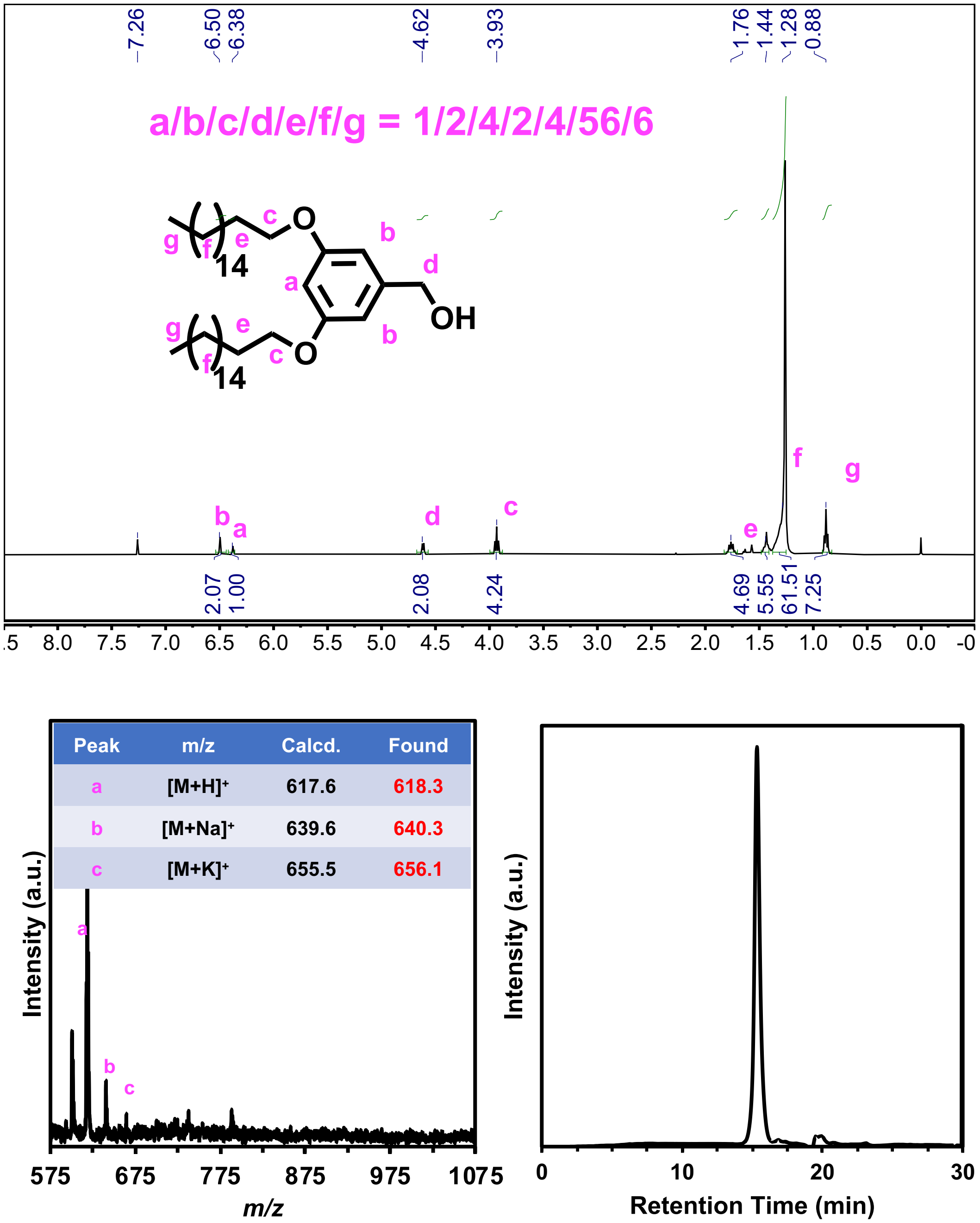

Peak	m/z	Calcd.	Found
a	[M+H]+	617.6	618.3
b	[M+Na]+	639.6	640.3
c	[M+K]+	655.5	656.1

Figure S10. ^{1}H NMR spectrum (top), MALDI-TOF MS spectrum (bottom left) and HPLC trace (bottom right) of (3,5-bis(heptadecyloxy)phenyl)methanol, **65d**.

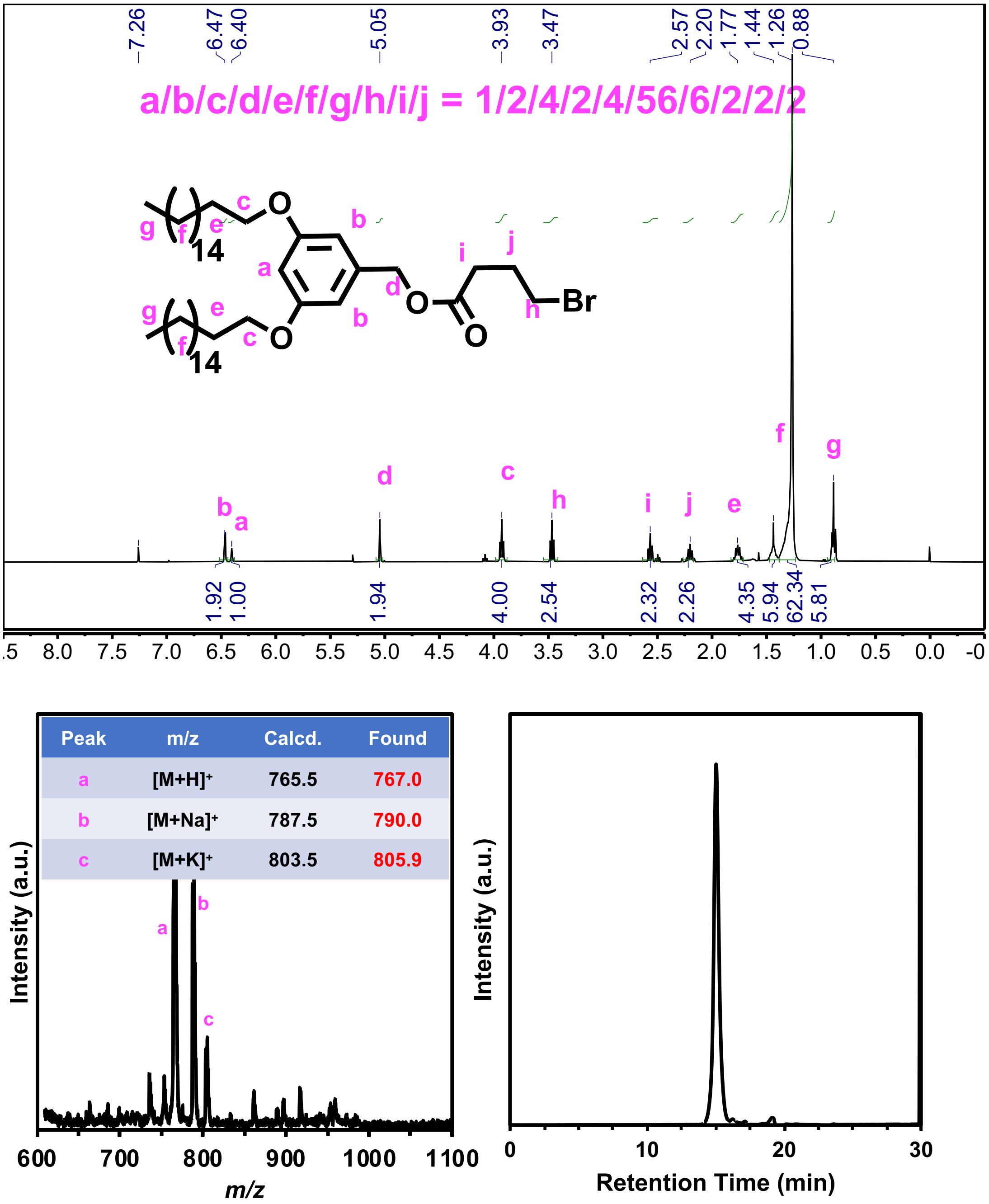

Figure S11. ^{1}H NMR spectrum (top), MALDI-TOF MS spectrum (bottom left) and HPLC trace (bottom right) of 3,5-bis(heptadecyloxy)benzyl 4-bromobutanoate, **66d**.

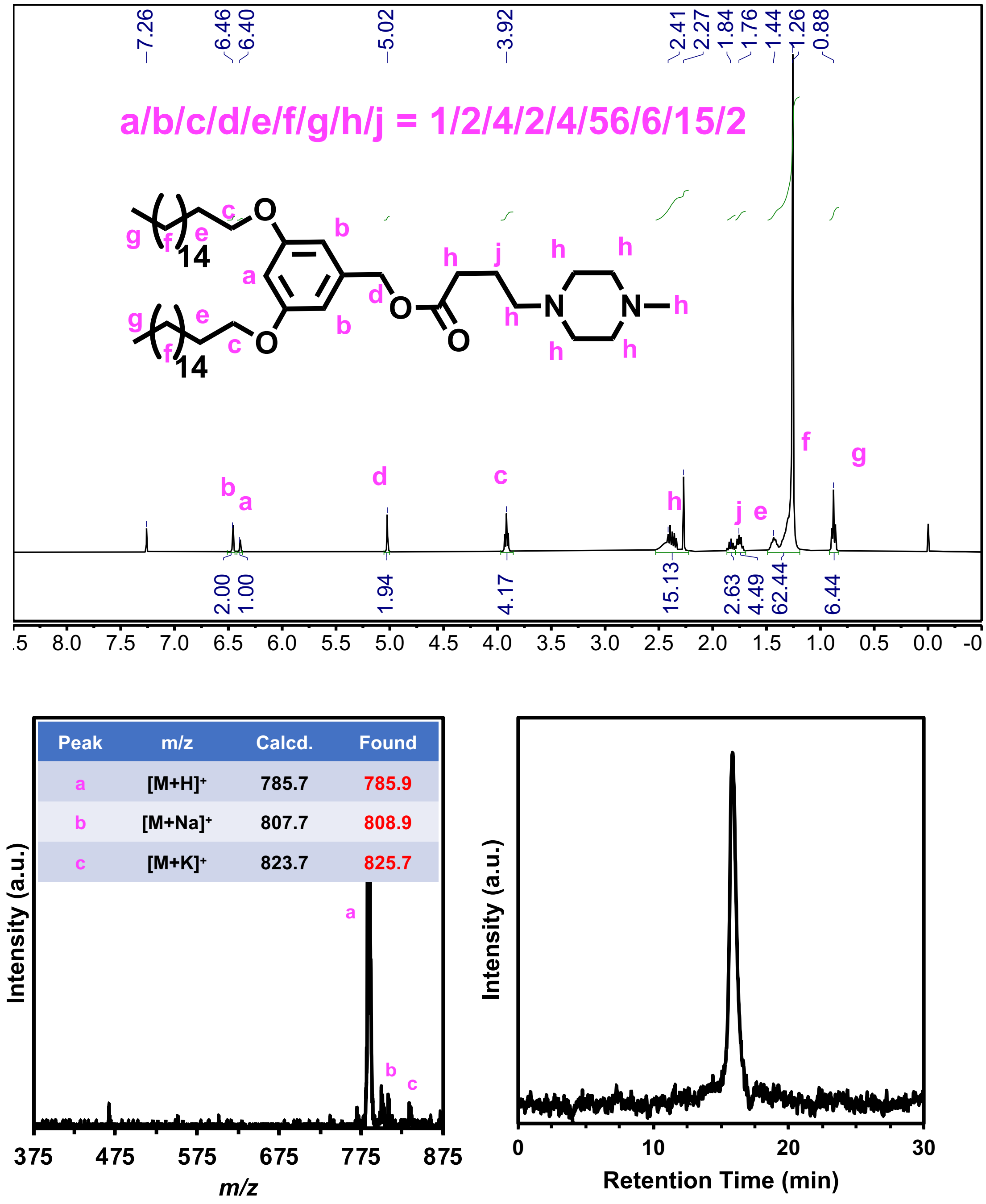

Peak	m/z	Calcd.	Found
a	[M+H]+	785.7	785.9
b	[M+Na]+	807.7	808.9
c	[M+K]+	823.7	825.7

Figure S12. ^{1}H NMR spectrum (top), MALDI-TOF MS spectrum (bottom left) and HPLC trace (bottom right) of 3,5-bis(pentadecyloxy)benzyl 4-(4-methylpiperazin-1-yl)butanoate, **67e**, IAJD265.

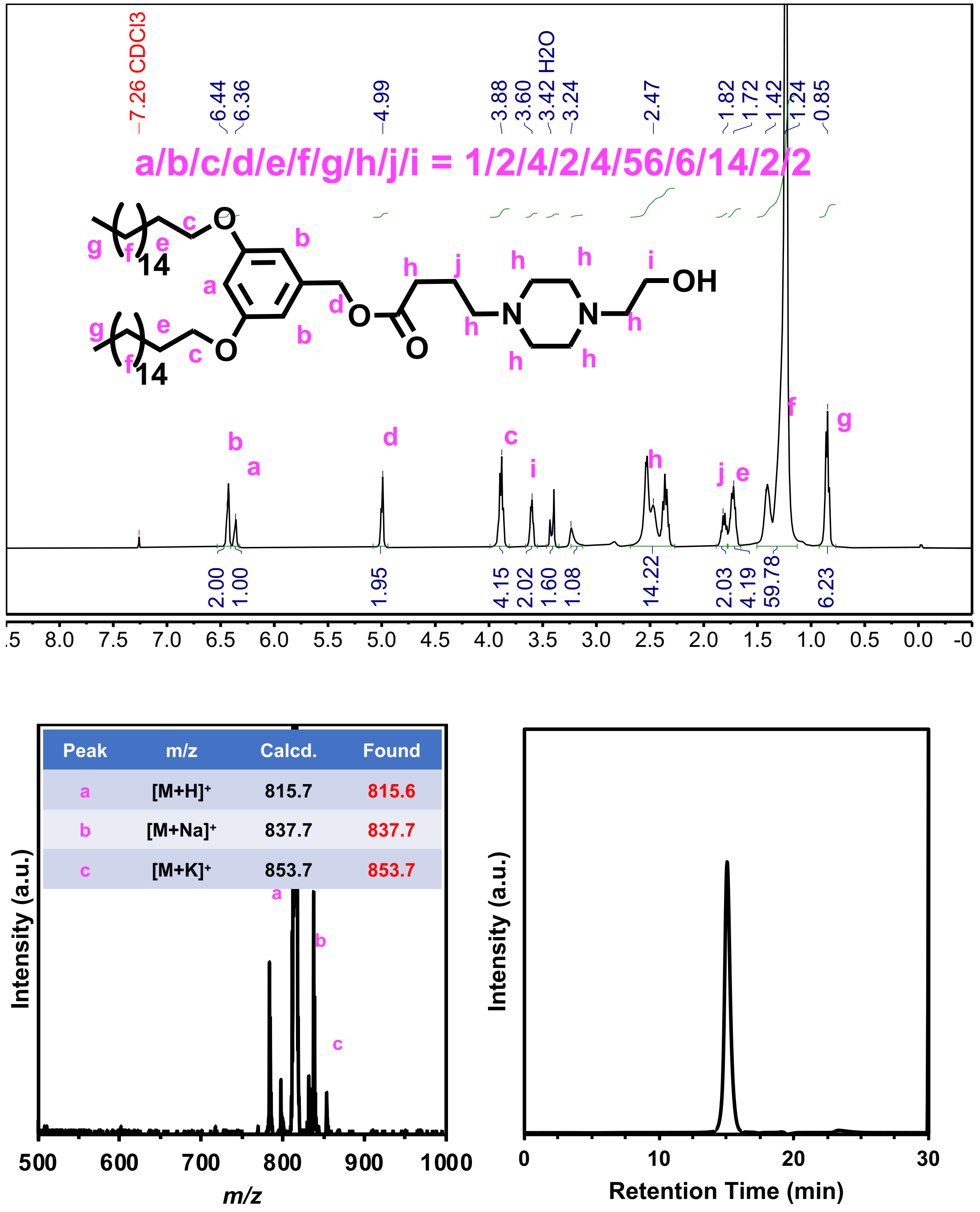

Peak	m/z	Calcd.	Found
a	$[M+H]^+$	815.7	815.6
b	$[M+Na]^+$	837.7	837.7
c	$[M+K]^+$	853.7	853.7

Figure S13. ^{1}H NMR spectrum (top), MALDI-TOF MS spectrum (bottom left) and HPLC trace (bottom right) of 3,5-Bis(pentadecyloxy)benzyl 4-(4-(2-hydroxyethyl)piperazin-1-yl)butanoate, **67f,** IAJD266.

4. DLS Data of DNPs Co-Assembled from IAJDs and Luc-mRNA

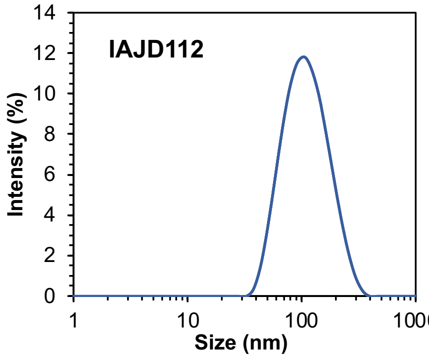

IAJD112 (4.0 mg/mL) + **Luc-mRNA** (0.10 mg/mL) in acetate buffer (pH = 4.0)

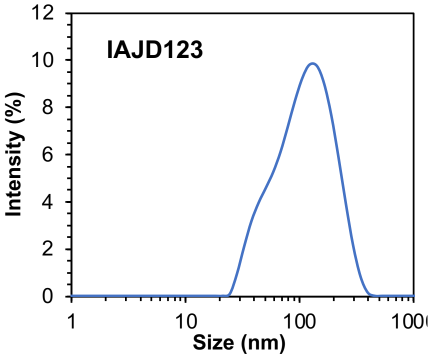

IAJD123 (4.0 mg/mL) + **Luc-mRNA** (0.10 mg/m in acetate buffer (pH = 4.0)

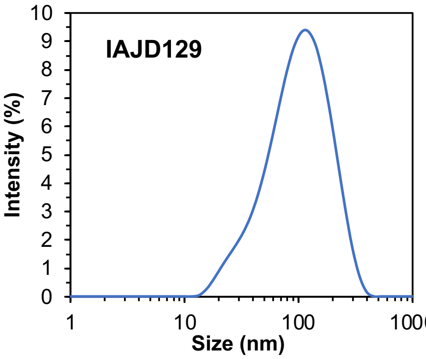

IAJD129 (4.0 mg/mL) + **Luc-mRNA** (0.10 mg/mL) in acetate buffer (pH = 4.0)

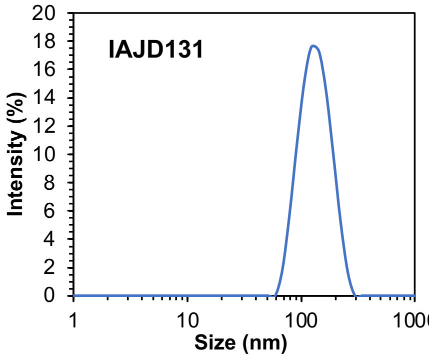

IAJD131 (4.0 mg/mL) + **Luc-mRNA** (0.10 mg/mL) in acetate buffer (pH = 4.0)

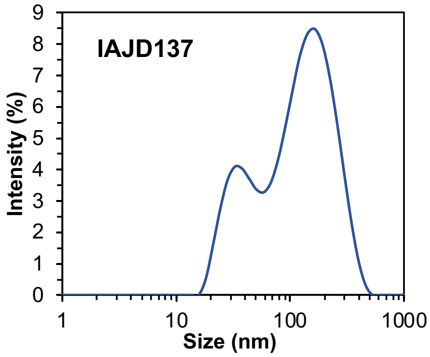

IAJD137 (4.0 mg/mL) + **Luc-mRNA** (0.10 mg/mL in acetate buffer (pH = 4.0)

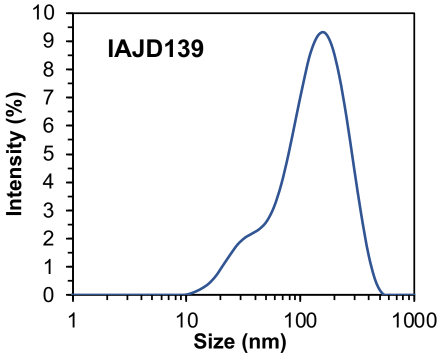

IAJD139 (4.0 mg/mL) + **Luc-mRNA** (0.10 mg/mL) in acetate buffer (pH = 4.0)

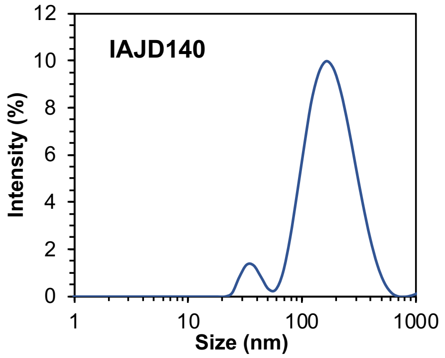

IAJD140 (4.0 mg/mL) + **Luc-mRNA** (0.10 mg/mL) in acetate buffer (pH = 4.0)

Figure S14. DLS data of DNPs assembled from IAJDs 112,123,129131,137,139 and 140.

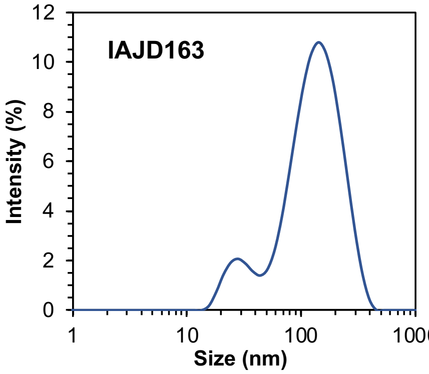

IAJD163 (4.0 mg/mL) + **Luc-mRNA** (0.10 mg/mL) in acetate buffer (pH = 4.0)
Size (D) = 98 nm, PDI = 0.289

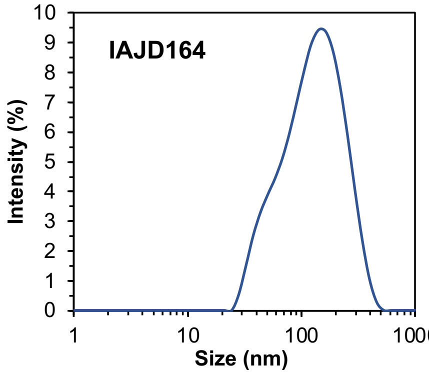

IAJD164 (4.0 mg/mL) + **Luc-mRNA** (0.10 mg/mL) in acetate buffer (pH = 4.0)
Size (D) = 105 nm, PDI = 0.253

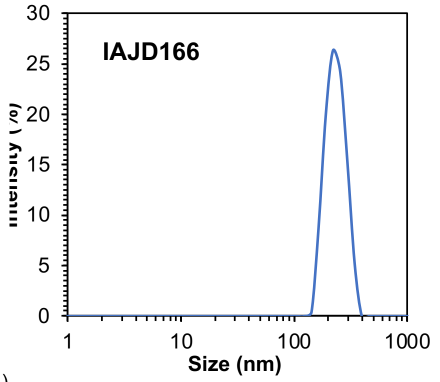

IAJD166 (4.0 mg/mL) + **Luc-mRNA** (0.10 mg/mL) in acetate buffer (pH = 4.0)
Size (D) = 232 nm, PDI = 0.102

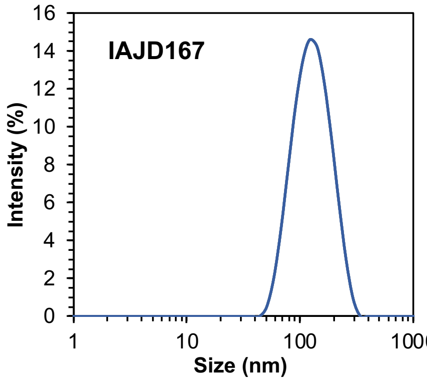

IAJD167 (4.0 mg/mL) + **Luc-mRNA** (0.10 mg/mL in acetate buffer (pH = 4.0)
Size (D) = 105 nm, PDI = 0.208

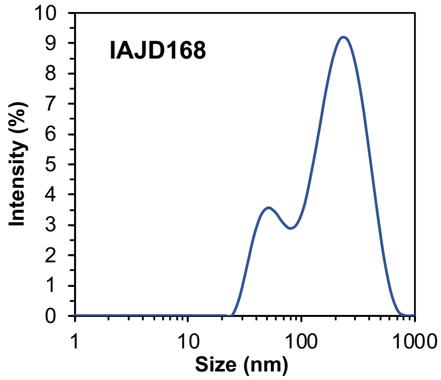

IAJD168 (4.0 mg/mL) + **Luc-mRNA** (0.10 mg/mL) in acetate buffer (pH = 4.0)
Size (D) = 138 nm, PDI = 0.318

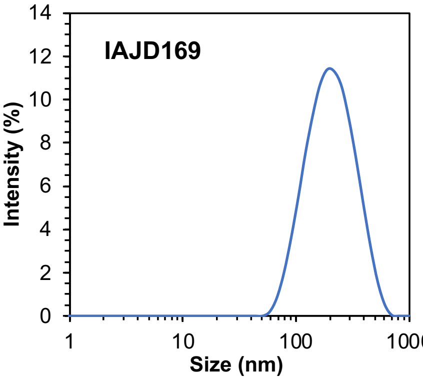

IAJD169 (4.0 mg/mL) + **Luc-mRNA** (0.10 mg/mL) in acetate buffer (pH = 4.0)
Size (D) = 165 nm, PDI = 0.247

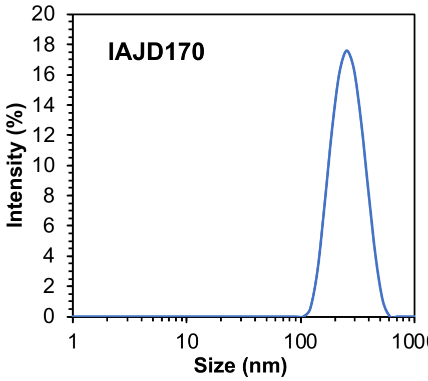

IAJD170 (4.0 mg/mL) + **Luc-mRNA** (0.10 mg/ml in acetate buffer (pH = 4.0)
Size (D) = 233 nm, PDI = 0.251

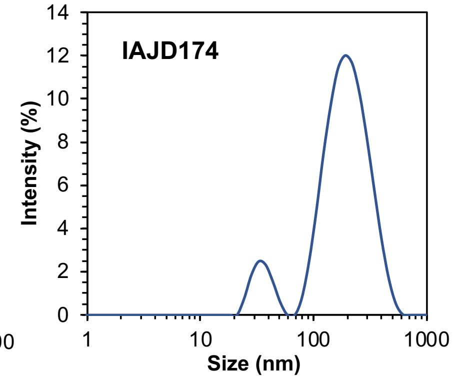

IAJD174 (4.0 mg/mL) + **Luc-mRNA** (0.10 mg/mL) in acetate buffer (pH = 4.0)
Size (D) = 145 nm, PDI = 0.286

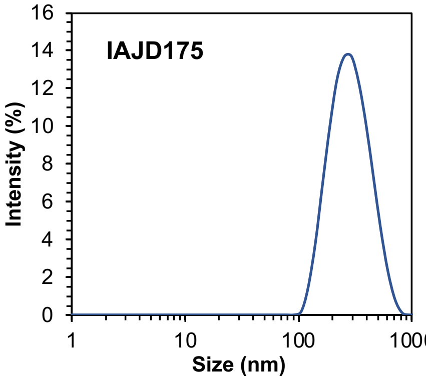

IAJD175 (4.0 mg/mL) + **Luc-mRNA** (0.10 mg/mL) in acetate buffer (pH = 4.0)
Size (D) = 246 nm, PDI = 0.198

Figure S15. DLS data of DNPs assembled from IAJDs 163,164,166 – 170, 174, 175.

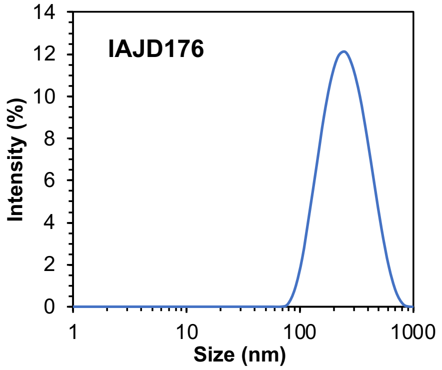

IAJD176 (4.0 mg/mL) + **Luc-mRNA** (0.10 mg/mL) in acetate buffer (pH = 4.0)
Size (D) = 216 nm, PDI = 0.203

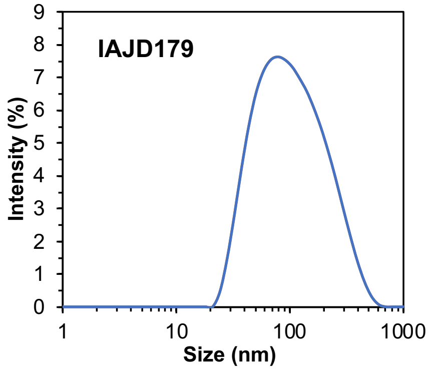

IAJD179 (4.0 mg/mL) + **Luc-mRNA** (0.10 mg/mL) in acetate buffer (pH = 4.0)
Size (D) = 83 nm, PDI = 0.265

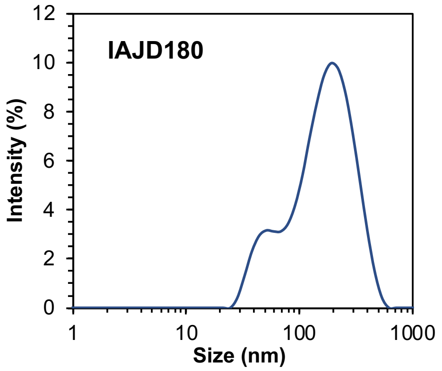

IAJD180 (4.0 mg/mL) + **Luc-mRNA** (0.10 mg/mL) in acetate buffer (pH = 4.0)
Size (D) = 130 nm, PDI = 0.297

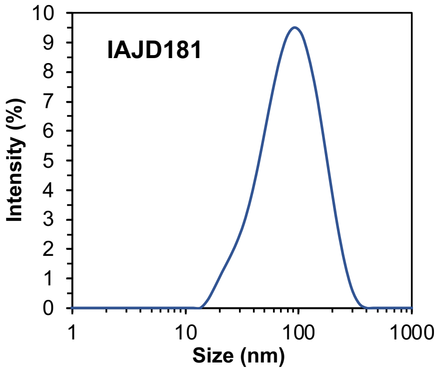

IAJD181 (4.0 mg/mL) + **Luc-mRNA** (0.10 mg/mL) in acetate buffer (pH = 4.0)
Size (D) = 74 nm, PDI = 0.263

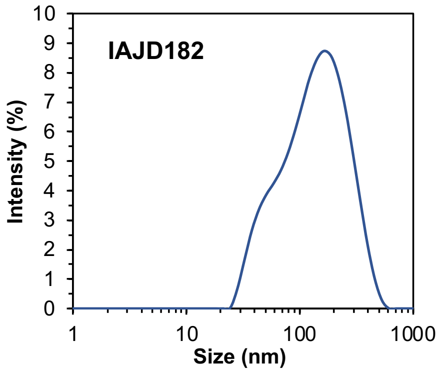

IAJD182 (4.0 mg/mL) + **Luc-mRNA** (0.10 mg/mL) in acetate buffer (pH = 4.0)
Size (D) = 109 nm, PDI = 0.271

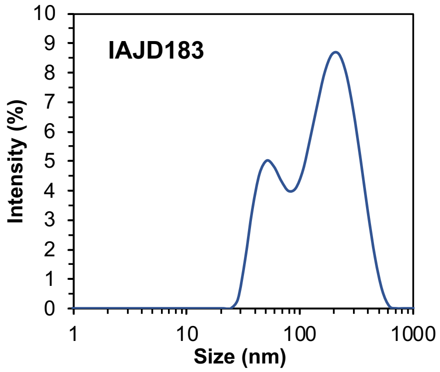

IAJD183 (4.0 mg/mL) + **Luc-mRNA** (0.10 mg/mL) in acetate buffer (pH = 4.0)
Size (D) = 112 nm, PDI = 0.290

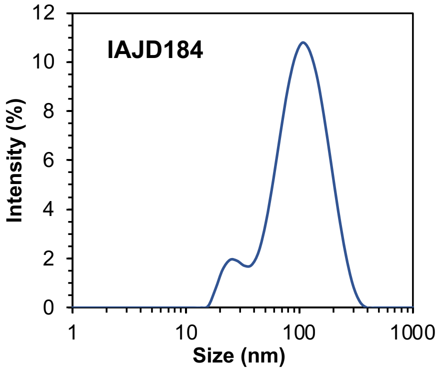

IAJD184 (4.0 mg/mL) + **Luc-mRNA** (0.10 mg/mL) in acetate buffer (pH = 4.0)
Size (D) = 85 nm, PDI = 0.270

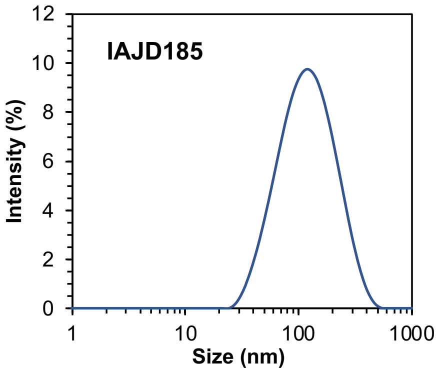

IAJD185 (4.0 mg/mL) + **Luc-mRNA** (0.10 mg/mL) in acetate buffer (pH = 4.0)
Size (D) = 106 nm, PDI = 0.247

Figure S16. DLS data of DNPs assembled from IAJDs 176,179-185.

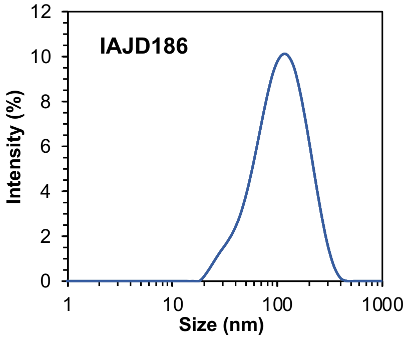

IAJD186 (4.0 mg/mL) + **Luc-mRNA** (0.10 mg/mL) in acetate buffer (pH = 4.0)
Size (D) = 94 nm, PDI = 0.253

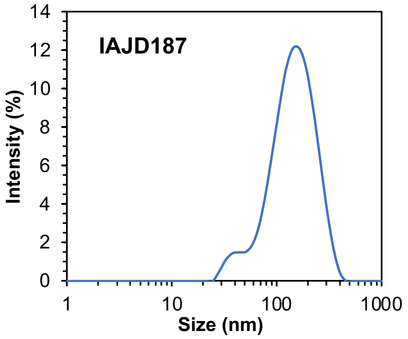

IAJD187 (4.0 mg/mL) + **Luc-mRNA** (0.10 mg/mL) in acetate buffer (pH = 4.0)
Size (D) = 123 nm, PDI = 0.199

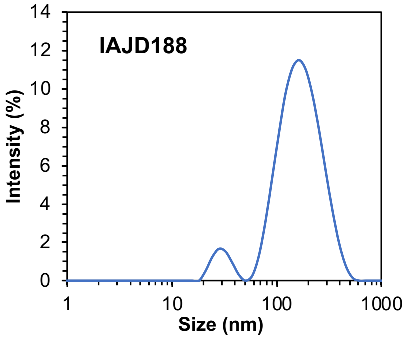

IAJD188 (4.0 mg/mL) + **Luc-mRNA** (0.10 mg/mL) in acetate buffer (pH = 4.0)
Size (D) = 140 nm, PDI = 0.280

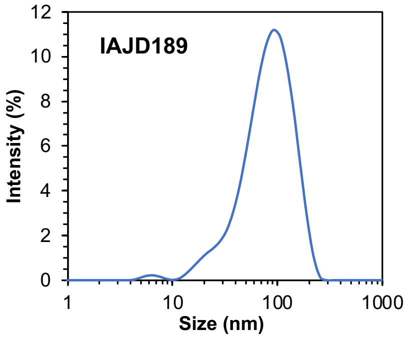

IAJD189 (4.0 mg/mL) + **Luc-mRNA** (0.10 mg/mL) in acetate buffer (pH = 4.0)
Size (D) = 81 nm, PDI = 0.336

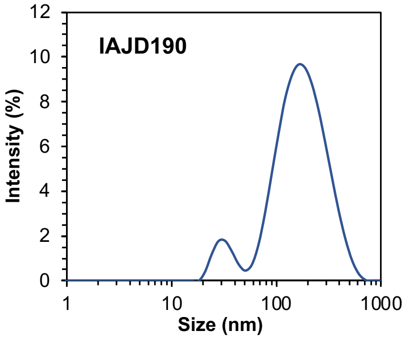

IAJD190 (4.0 mg/mL) + **Luc-mRNA** (0.10 mg/mL) in acetate buffer (pH = 4.0)
Size (D) = 141 nm, PDI = 0.387

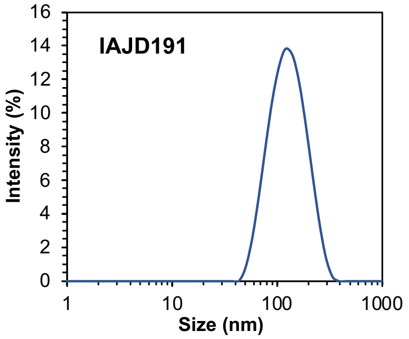

IAJD191 (4.0 mg/mL) + **Luc-mRNA** (0.10 mg/mL) in acetate buffer (pH = 4.0)
Size (D) = 111 nm, PDI = 0.176

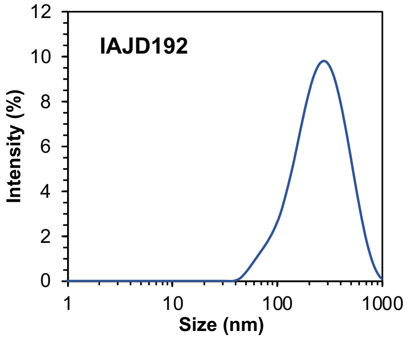

IAJD192 (4.0 mg/mL) + **Luc-mRNA** (0.10 mg/mL) in acetate buffer (pH = 4.0)
Size (D) = 230 nm, PDI = 0.294

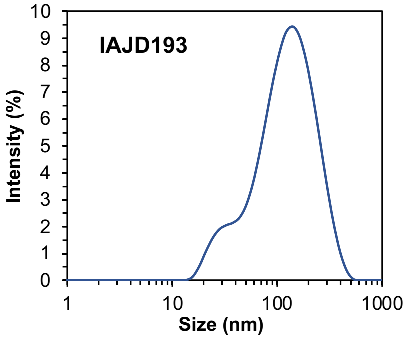

IAJD193 (4.0 mg/mL) + **Luc-mRNA** (0.10 mg/mL) in acetate buffer (pH = 4.0)
Size (D) = 97 nm, PDI = 0.289

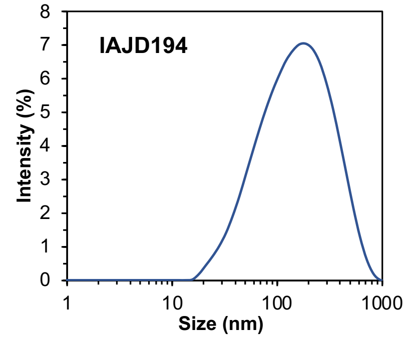

IAJD194 (4.0 mg/mL) + **Luc-mRNA** (0.10 mg/mL) in acetate buffer (pH = 4.0)
Size (D) = 114 nm, PDI = 0.390

Figure S17. DLS data of DNPs assembled from IAJDs 186-194.

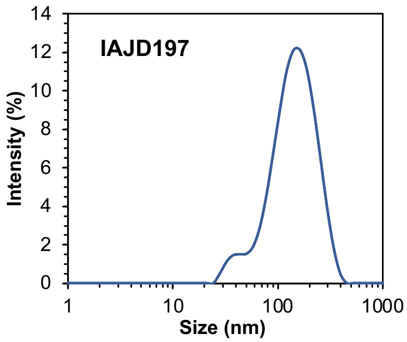

IAJD197 (4.0 mg/mL) + **Luc-mRNA** (0.10 mg/mL) in acetate buffer (pH = 4.0)
Size (D) = 109 nm, PDI = 0.251

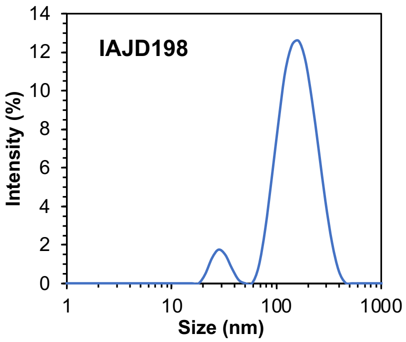

IAJD198 (4.0 mg/mL) + **Luc-mRNA** (0.10 mg/mL) in acetate buffer (pH = 4.0)
Size (D) = 142 nm, PDI = 0.291

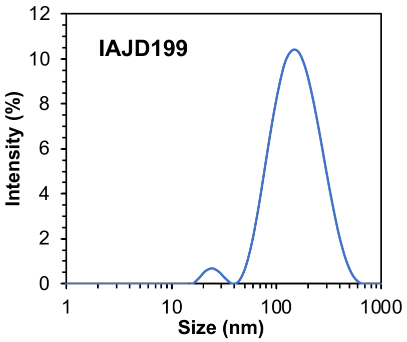

IAJD199 (4.0 mg/mL) + **Luc-mRNA** (0.10 mg/mL) in acetate buffer (pH = 4.0)
Size (D) = 134 nm, PDI = 0.259

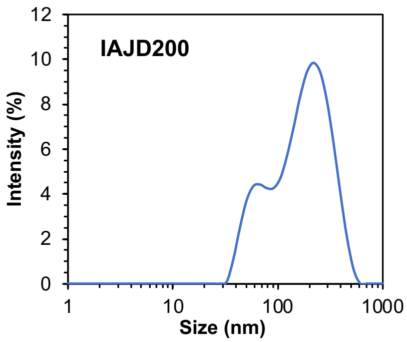

IAJD200 (4.0 mg/mL) + **Luc-mRNA** (0.10 mg/mL) in acetate buffer (pH = 4.0)
Size (D) = 139 nm, PDI = 0.376

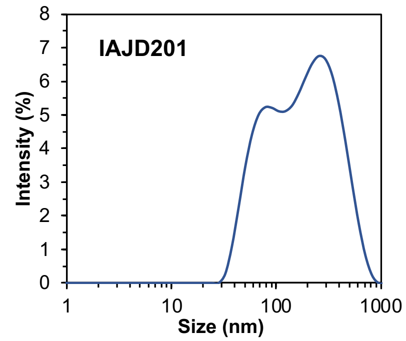

IAJD201 (4.0 mg/mL) + **Luc-mRNA** (0.10 mg/mL) in acetate buffer (pH = 4.0)
Size (D) = 143 nm, PDI = 0.434

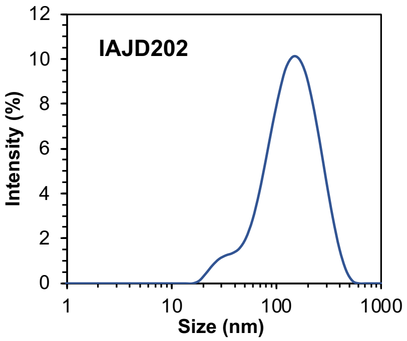

IAJD202 (4.0 mg/mL) + **Luc-mRNA** (0.10 mg/mL) in acetate buffer (pH = 4.0)
Size (D) = 113 nm, PDI = 0.260

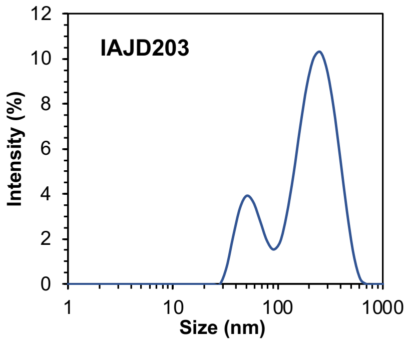

IAJD203 (4.0 mg/mL) + **Luc-mRNA** (0.10 mg/mL) in acetate buffer (pH = 4.0)
Size (D) = 226 nm, PDI = 0.510

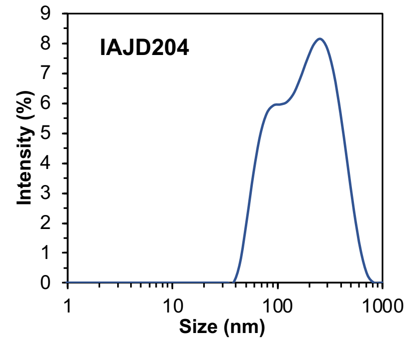

IAJD204 (4.0 mg/mL) + **Luc-mRNA** (0.10 mg/mL) in acetate buffer (pH = 4.0)
Size (D) = 140 nm, PDI = 0.415

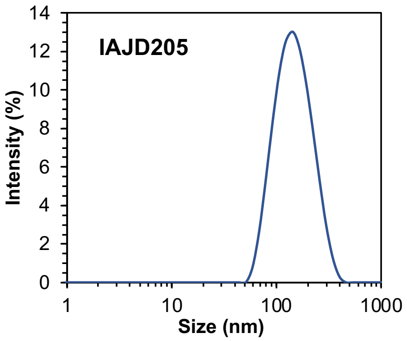

IAJD205 (4.0 mg/mL) + **Luc-mRNA** (0.10 mg/mL) in acetate buffer (pH = 4.0)
Size (D) = 138 nm, PDI = 0.271

Figure S18. DLS data of DNPs assembled from IAJDs 197-205.

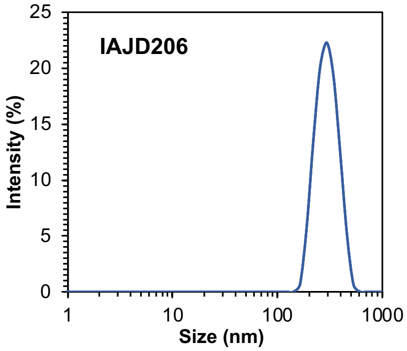

IAJD206 (4.0 mg/mL) + **Luc-mRNA** (0.10 mg/mL) in acetate buffer (pH = 4.0)
Size (D) = 321 nm, PDI = 0.293

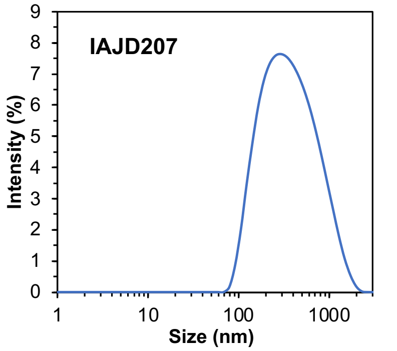

IAJD207 (4.0 mg/mL) + **Luc-mRNA** (0.10 mg/mL) in acetate buffer (pH = 4.0)
Size (D) = 261 nm, PDI = 0.324

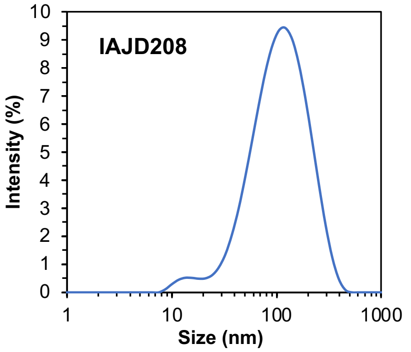

IAJD208 (4.0 mg/mL) + **Luc-mRNA** (0.10 mg/mL) in acetate buffer (pH = 4.0)
Size (D) = 89 nm, PDI = 0.290

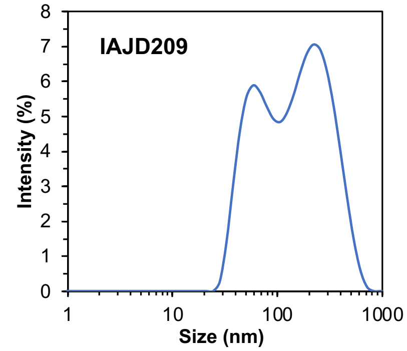

IAJD209 (4.0 mg/mL) + **Luc-mRNA** (0.10 mg/mL) in acetate buffer (pH = 4.0)
Size (D) = 106 nm, PDI = 0.375

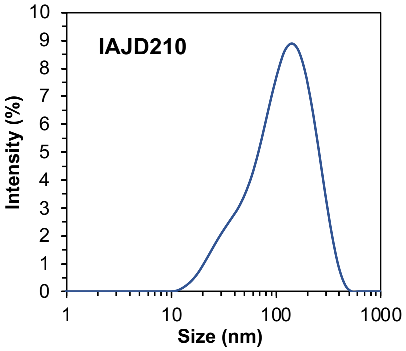

IAJD210 (4.0 mg/mL) + **Luc-mRNA** (0.10 mg/mL) in acetate buffer (pH = 4.0)
Size (D) = 90 nm, PDI = 0.302

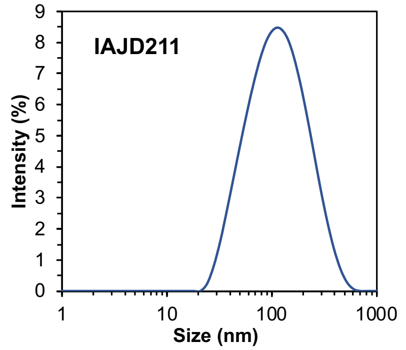

IAJD211 (4.0 mg/mL) + **Luc-mRNA** (0.10 mg/mL) in acetate buffer (pH = 4.0)
Size (D) = 96 nm, PDI = 0.271

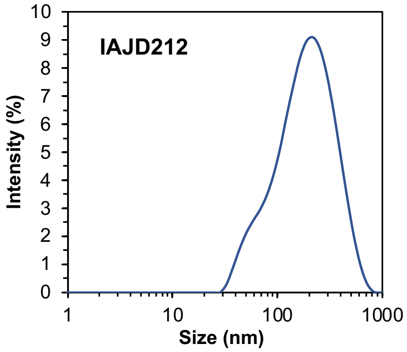

IAJD212 (4.0 mg/mL) + **Luc-mRNA** (0.10 mg/mL) in acetate buffer (pH = 4.0)
Size (D) = 128 nm, PDI = 0.247

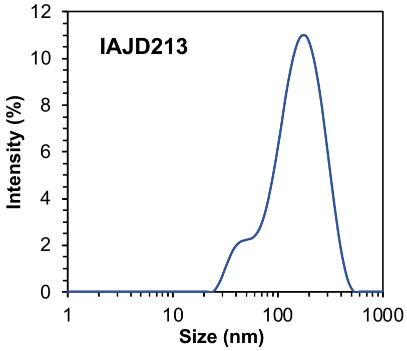

IAJD213 (4.0 mg/mL) + **Luc-mRNA** (0.10 mg/mL) in acetate buffer (pH = 4.0)
Size (D) = 156 nm, PDI = 0.296

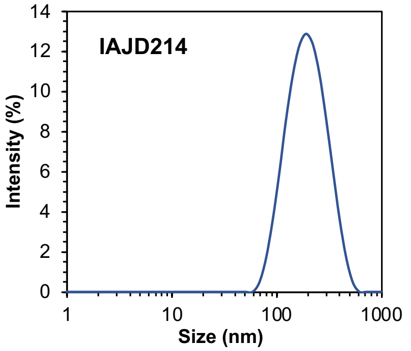

IAJD214 (4.0 mg/mL) + **Luc-mRNA** (0.10 mg/mL) in acetate buffer (pH = 4.0)
Size (D) = 196 nm, PDI = 0.236

Figure S19. DLS data of DNPs assembled from IAJDs 206-214.

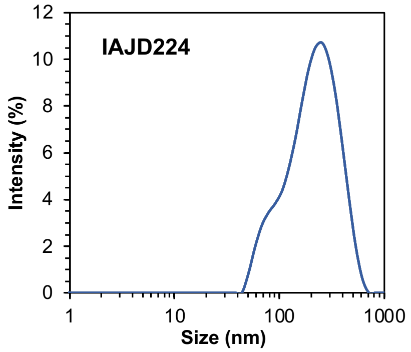

IAJD224 (4.0 mg/mL) + **Luc-mRNA** (0.10 mg/mL) in acetate buffer (pH = 4.0)
Size (D) = 175 nm, PDI = 0.231

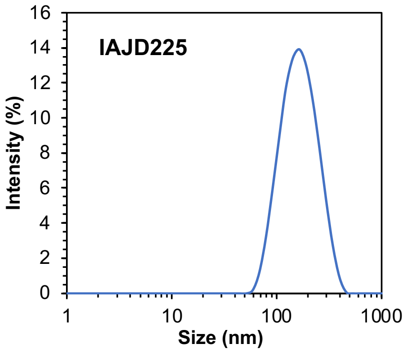

IAJD225 (4.0 mg/mL) + **Luc-mRNA** (0.10 mg/mL) in acetate buffer (pH = 4.0)
Size (D) = 153 nm, PDI = 0.193

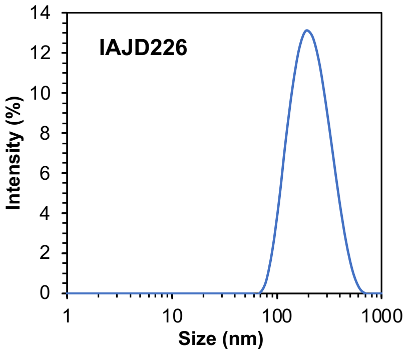

IAJD226 (4.0 mg/mL) + **Luc-mRNA** (0.10 mg/mL) in acetate buffer (pH = 4.0)
Size (D) = 191 nm, PDI = 0.174

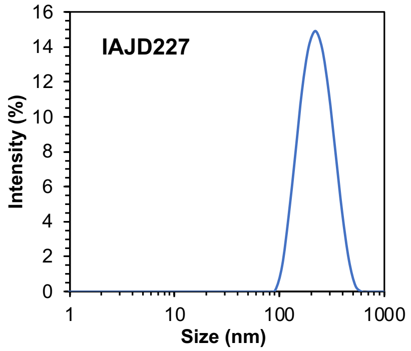

IAJD227 (4.0 mg/mL) + **Luc-mRNA** (0.10 mg/mL) in acetate buffer (pH = 4.0)
Size (D) = 229 nm, PDI = 0.299

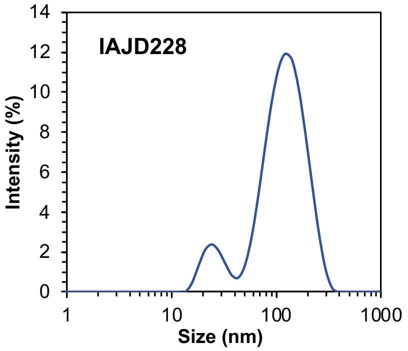

IAJD228 (4.0 mg/mL) + **Luc-mRNA** (0.10 mg/mL) in acetate buffer (pH = 4.0)
Size (D) = 89 nm, PDI = 0.279

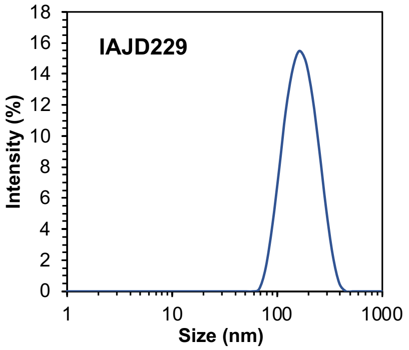

IAJD229 (4.0 mg/mL) + **Luc-mRNA** (0.10 mg/mL) in acetate buffer (pH = 4.0)
Size (D) = 158 nm, PDI = 0.108

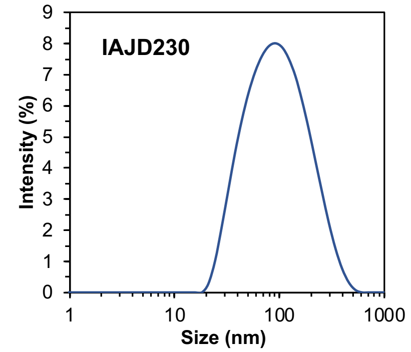

IAJD230 (4.0 mg/mL) + **Luc-mRNA** (0.10 mg/mL) in acetate buffer (pH = 4.0)
Size (D) = 78 nm, PDI = 0.254

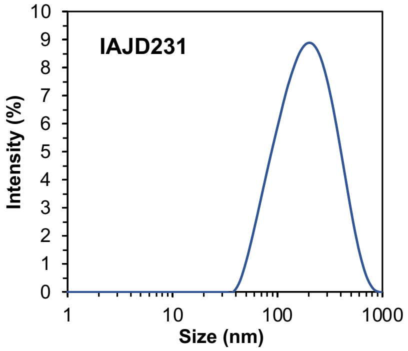

IAJD231 (4.0 mg/mL) + **Luc-mRNA** (0.10 mg/mL) in acetate buffer (pH = 4.0)
Size (D) = 159 nm, PDI = 0.243

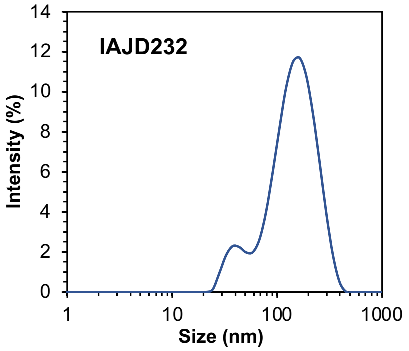

IAJD232 (4.0 mg/mL) + **Luc-mRNA** (0.10 mg/mL) in acetate buffer (pH = 4.0)
Size (D) = 126 nm, PDI = 0.324

Figure S20. DLS data of DNPs assembled from IAJDs 224-232.

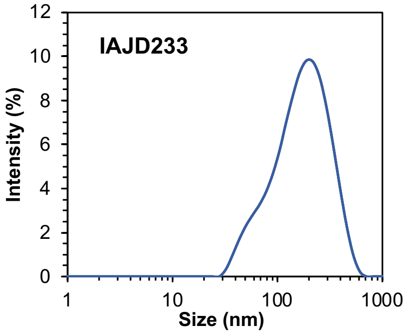

IAJD233 (4.0 mg/mL) + **Luc-mRNA** (0.10 mg/mL) in acetate buffer (pH = 4.0)
Size (D) = 146 nm, PDI = 0.267

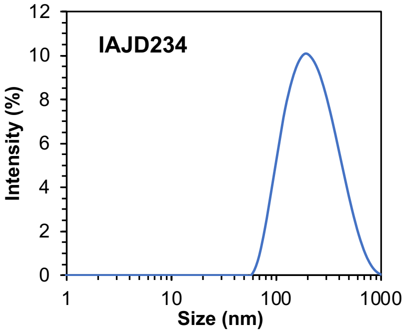

IAJD234 (4.0 mg/mL) + **Luc-mRNA** (0.10 mg/mL) in acetate buffer (pH = 4.0)
Size (D) = 192 nm, PDI = 0.216

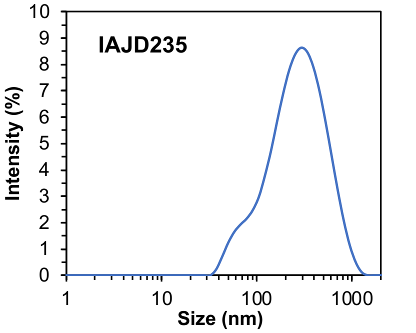

IAJD235 (4.0 mg/mL) + **Luc-mRNA** (0.10 mg/mL) in acetate buffer (pH = 4.0)
Size (D) = 212 nm, PDI = 0.294

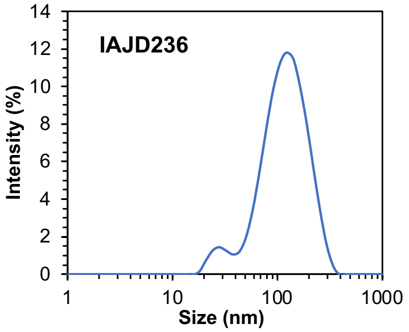

IAJD236 (4.0 mg/mL) + **Luc-mRNA** (0.10 mg/mL) in acetate buffer (pH = 4.0)
Size (D) = 98 nm, PDI = 0.228

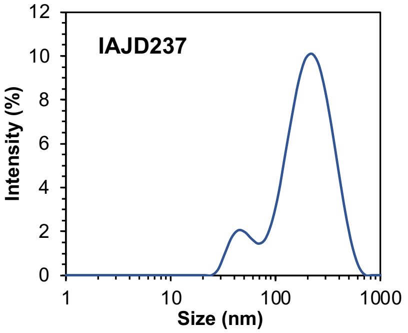

IAJD237 (4.0 mg/mL) + **Luc-mRNA** (0.10 mg/mL) in acetate buffer (pH = 4.0)
Size (D) = 173 nm, PDI = 0.397

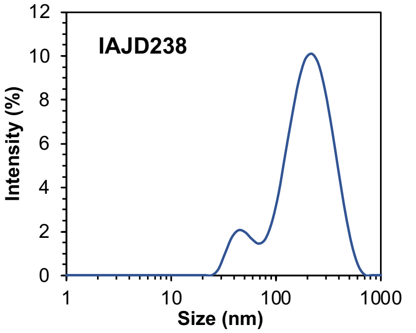

IAJD238 (4.0 mg/mL) + **Luc-mRNA** (0.10 mg/mL) in acetate buffer (pH = 4.0)
Size (D) = 126 nm, PDI = 0.289

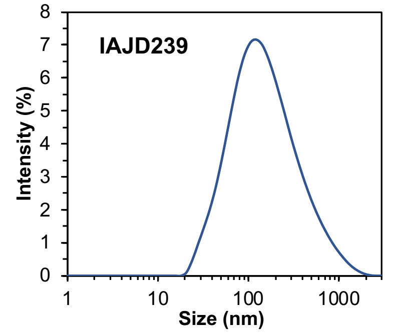

IAJD239 (4.0 mg/mL) + **Luc-mRNA** (0.10 mg/mL) in acetate buffer (pH = 4.0)
Size (D) = 112 nm, PDI = 0.381

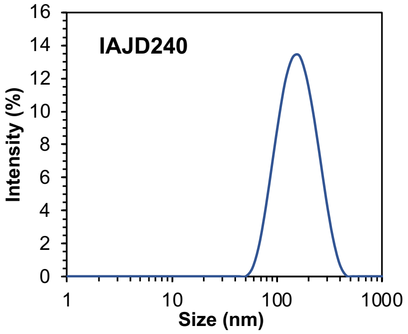

IAJD240 (4.0 mg/mL) + **Luc-mRNA** (0.10 mg/mL) in acetate buffer (pH = 4.0)
Size (D) = 131 nm, PDI = 0.206

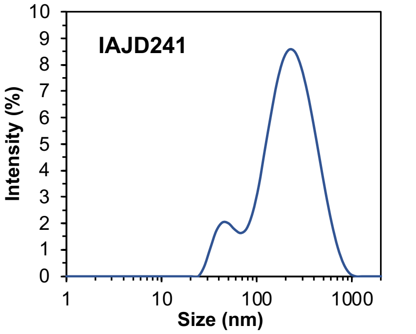

IAJD241 (4.0 mg/mL) + **Luc-mRNA** (0.10 mg/mL) in acetate buffer (pH = 4.0)
Size (D) = 183 nm, PDI = 0.442

Figure S21. DLS data of DNPs assembled from IAJDs 233-241.

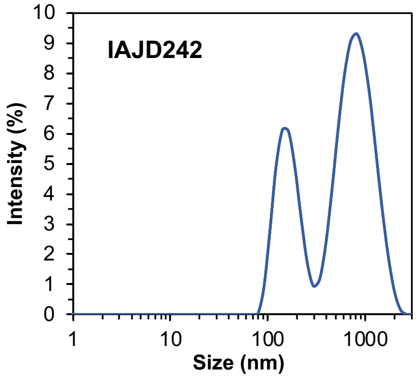

IAJD242 (4.0 mg/mL) + **Luc-mRNA** (0.10 mg/mL) in acetate buffer (pH = 4.0)
Size (D) = 224 nm, PDI = 0.263

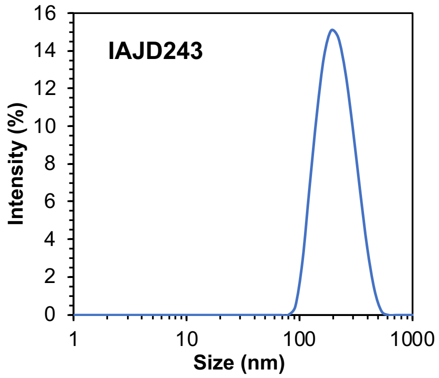

IAJD243 (4.0 mg/mL) + **Luc-mRNA** (0.10 mg/mL) in acetate buffer (pH = 4.0)
Size (D) = 227 nm, PDI = 0.309

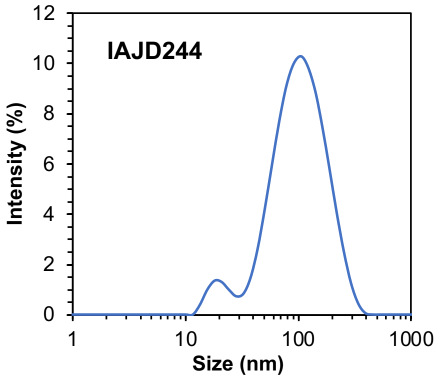

IAJD244 (4.0 mg/mL) + **Luc-mRNA** (0.10 mg/mL) in acetate buffer (pH = 4.0)
Size (D) = 86 nm, PDI = 0.275

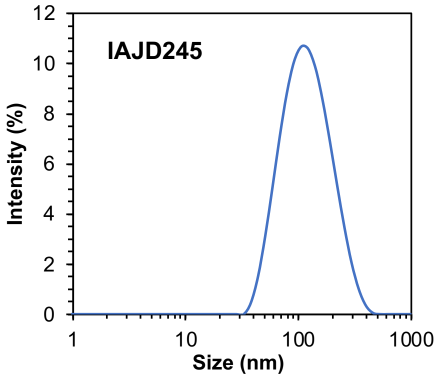

IAJD245 (4.0 mg/mL) + **Luc-mRNA** (0.10 mg/mL) in acetate buffer (pH = 4.0)
Size (D) = 103 nm, PDI = 0.341

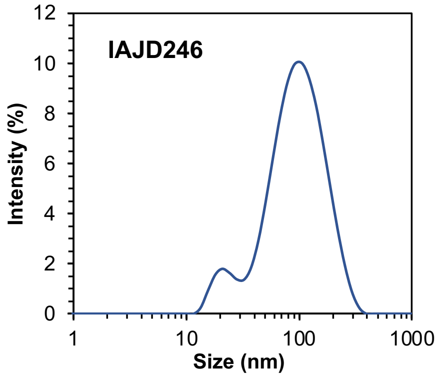

IAJD246 (4.0 mg/mL) + **Luc-mRNA** (0.10 mg/mL) in acetate buffer (pH = 4.0)
Size (D) = 80 nm, PDI = 0.295

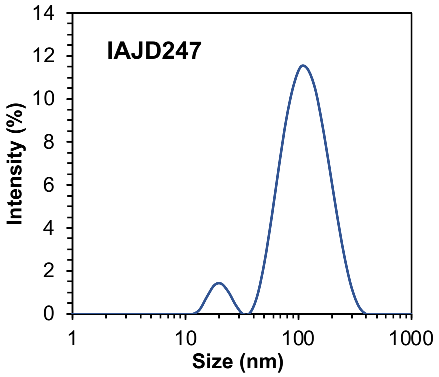

IAJD247 (4.0 mg/mL) + **Luc-mRNA** (0.10 mg/mL) in acetate buffer (pH = 4.0)
Size (D) = 95 nm, PDI = 0.304

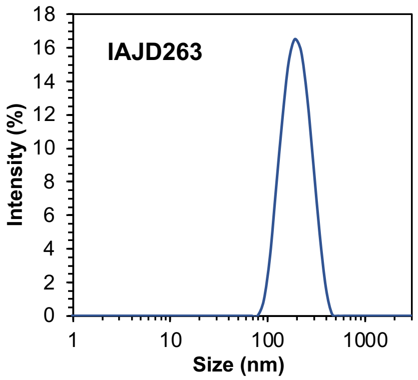

IAJD263 (4.0 mg/mL) + **Luc-mRNA** (0.10 mg/mL) in acetate buffer (pH = 4.0)
Size (D) = 167 nm, PDI = 0.234

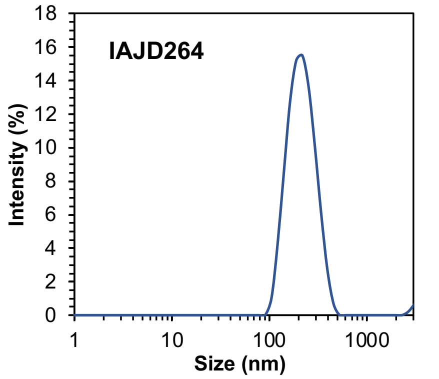

IAJD264 (4.0 mg/mL) + **Luc-mRNA** (0.10 mg/mL) in acetate buffer (pH = 4.0)
Size (D) = 395 nm, PDI = 0.273

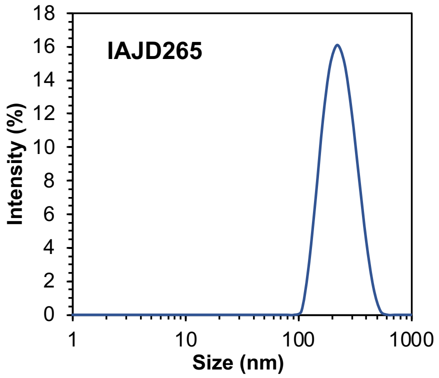

IAJD265 (4.0 mg/mL) + **Luc-mRNA** (0.10 mg/mL) in acetate buffer (pH = 4.0)
Size (D) = 254 nm, PDI = 0.299

Figure S22. DLS data of DNPs assembled from IAJDs 242-247, 263-265.

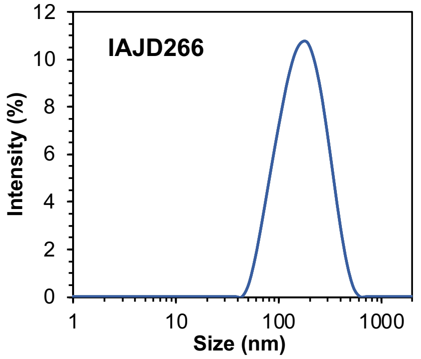

IAJD266 (4.0 mg/mL) + **Luc-mRNA** (0.10 mg/mL)
in acetate buffer (pH = 4.0)
Size (D) = 237 nm, PDI = 0.307

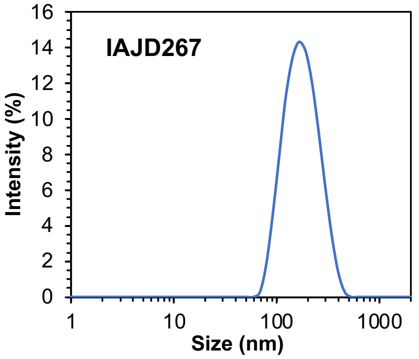

IAJD267 (4.0 mg/mL) + **Luc-mRNA** (0.10 mg/mL)
in acetate buffer (pH = 4.0)
Size (D) = 166 nm, PDI = 0.211

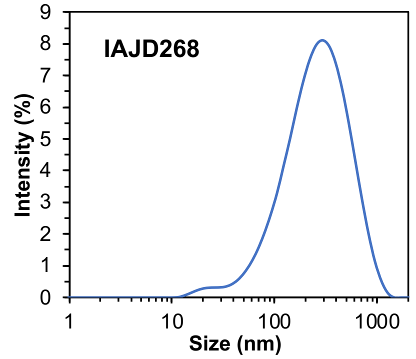

IAJD268 (4.0 mg/mL) + **Luc-mRNA** (0.10 mg/mL)
in acetate buffer (pH = 4.0)
Size (D) = 204 nm, PDI = 0.408

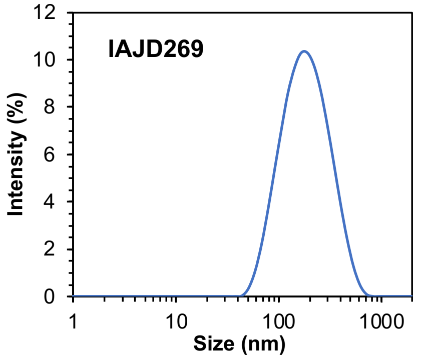

IAJD269 (4.0 mg/mL) + **Luc-mRNA** (0.10 mg/mL)
in acetate buffer (pH = 4.0)
Size (D) = 155 nm, PDI = 0.265

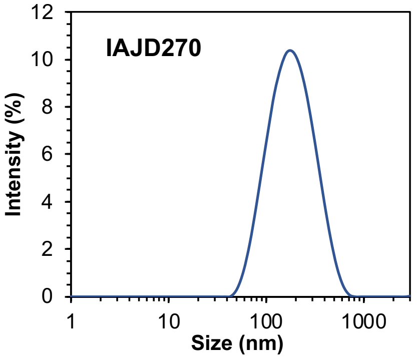

IAJD270 (4.0 mg/mL) + **Luc-mRNA** (0.10 mg/mL)
in acetate buffer (pH = 4.0)
Size (D) = 166 nm, PDI = 0.230

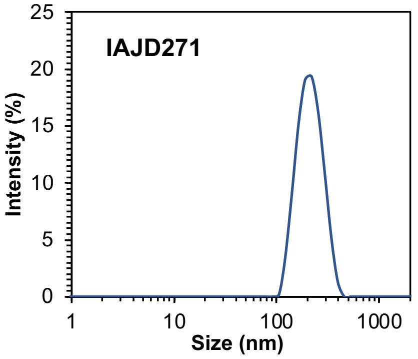

IAJD271 (4.0 mg/mL) + **Luc-mRNA** (0.10 mg/mL)
in acetate buffer (pH = 4.0)
Size (D) = 196 nm, PDI = 0.100

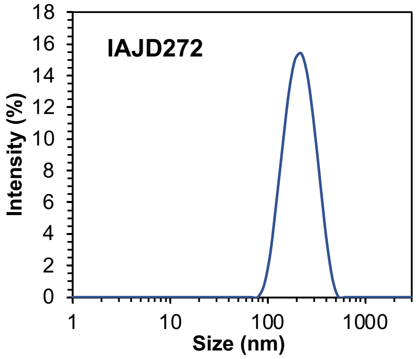

IAJD272 (4.0 mg/mL) + **Luc-mRNA** (0.10 mg/mL)
in acetate buffer (pH = 4.0)
Size (D) = 186 nm, PDI = 0.154

Figure S23. DLS data of DNPs assembled from IAJDs 266-272.

5. pK_a Measurements of Individual IAJDs

Table S1. pK_a of Individual IAJDs

IAJD No.	pKa
IAJD 73	6.30
IAJD 90	6.36
IAJD 112	6.4
IAJD 121	6.64
IAJD 123	6.61
IAJD 129	6.49
IAJD 131	7.34
IAJD 137	6.44
IAJD 139	6.43
IAJD 140	6.43
IAJD 163	6.3
IAJD 164	6.29
IAJD 165	6.41
IAJD 166	6.34
IAJD 167	6.24
IAJD 168	6.56
IAJD 169	6.44
IAJD 170	6.27
IAJD 174	6.31
IAJD 175	6.44
IAJD 176	6.29
IAJD 179	6.51
IAJD 180	6.49
IAJD 181	6.42
IAJD 182	6.41
IAJD 183	6.32
IAJD 184	6.36
IAJD 185	6.3
IAJD 186	6.19
IAJD 187	6.43
IAJD 188	6.46
IAJD 189	6.3
IAJD 190	6.35
IAJD 191	6.54
IAJD 192	6.42
IAJD 193	6.34
IAJD 194	6.37
IAJD 197	6.33
IAJD 198	6.28
IAJD 199	6.41
IAJD 200	6.17
IAJD 201	6.27
IAJD 202	6.24
IAJD 203	6.35
IAJD 204	6.26
IAJD 205	6.27
IAJD 206	6.28
IAJD 207	6.14
IAJD 208	6.2
IAJD 209	6.26
IAJD 210	6.19
IAJD 211	6.1
IAJD 212	6.26
IAJD 213	6.05
IAJD 214	6.23
IAJD 215	6.3
IAJD 216	6.26
IAJD 217	6.2
IAJD 218	6.26
IAJD 219	6.35
IAJD 220	6.26
IAJD 221	6.22
IAJD 222	6.39
IAJD 223	6.46
IAJD 224	6.33
IAJD 225	6.36
IAJD 226	6.46
IAJD 227	6.49
IAJD 228	6.56
IAJD 229	6.3
IAJD 230	6.3
IAJD 231	5.95
IAJD 232	6.28
IAJD 233	6.31
IAJD 234	6.35
IAJD 235	6.24
IAJD 236	6.34
IAJD 237	6.26
IAJD 238	6.25
IAJD 239	6.22
IAJD 240	6.29
IAJD 241	6.33
IAJD 242	6.31
IAJD 243	6.34
IAJD 244	6.3
IAJD 245	6.4
IAJD 246	6.32
IAJD 247	6.39
IAJD 263	6.48
IAJD 264	6.54
IAJD 265	6.54
IAJD 266	6.54
IAJD 267	6.52
IAJD 268	6.45
IAJD 269	6.5
IAJD 270	6.5
IAJD 271	6.45
IAJD 272	6.48

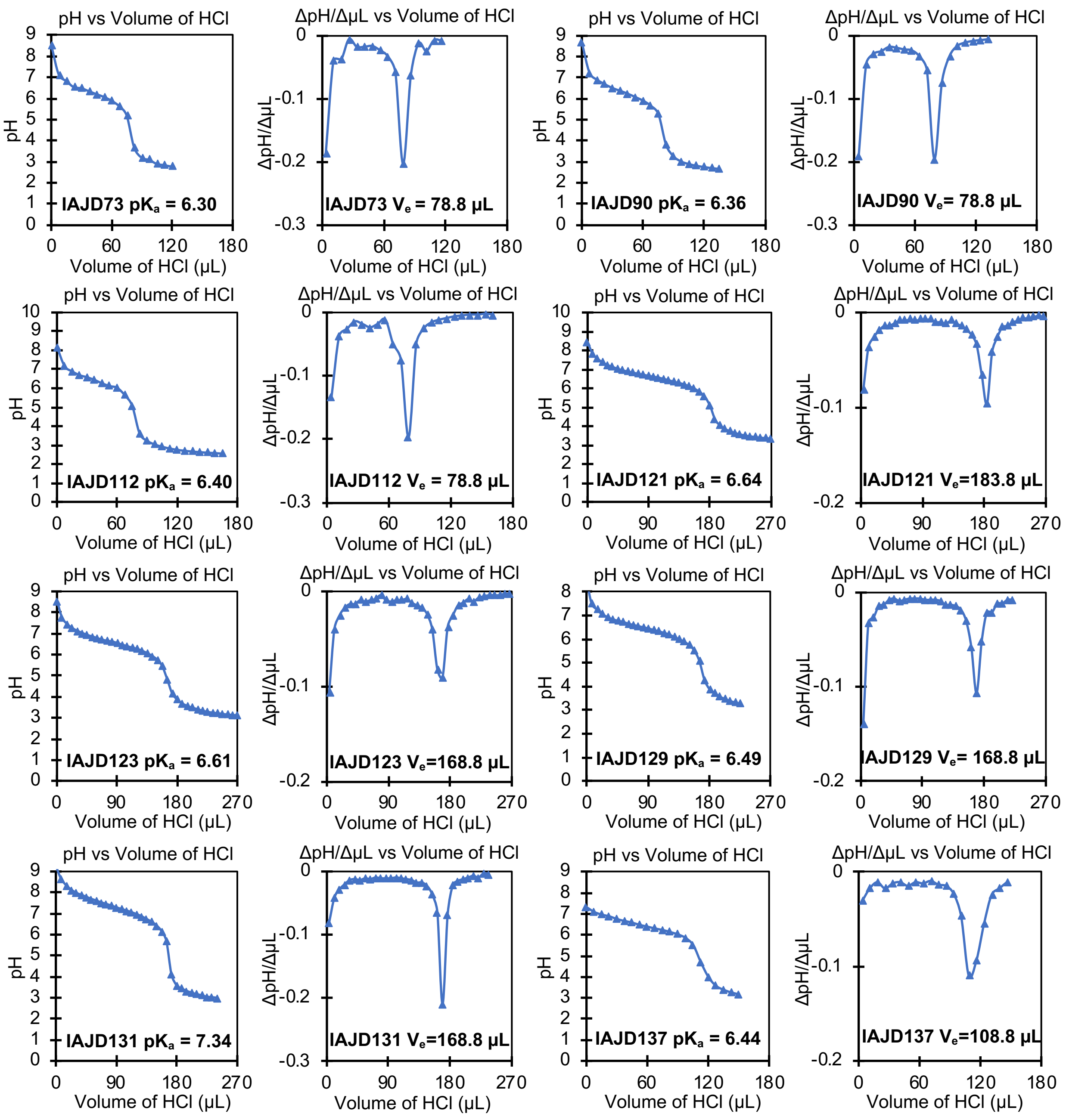

Figure S24. Titration curves showing changes in solution pH in response to addition of a strong acid and calculated pKa for IAJDs 73,90,112,121,123,129,131,137.

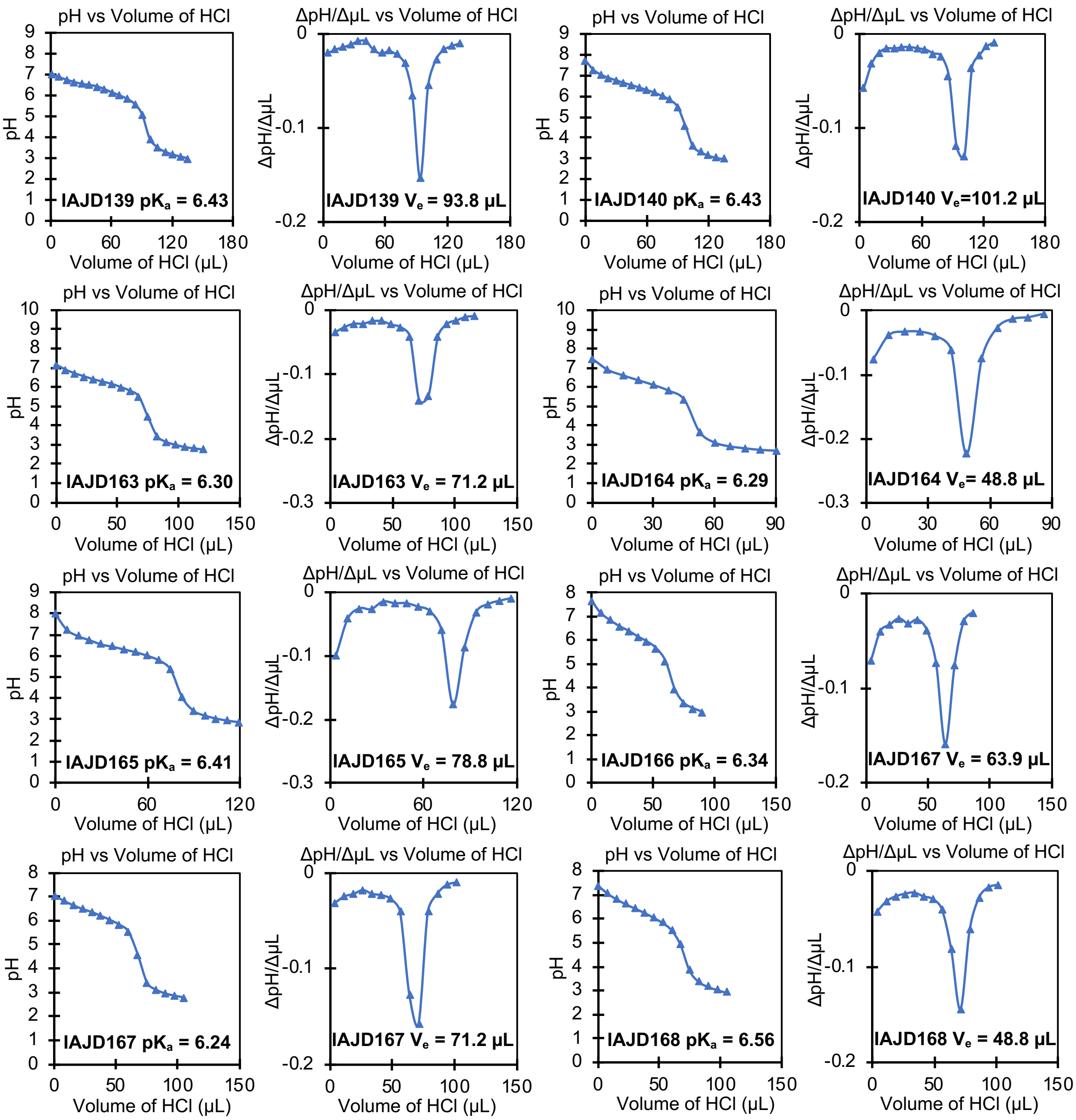

Figure S25. Titration curves showing changes in solution pH in response to addition of a strong acid and calculated pKa for IAJDs 139, 140, 163-168.

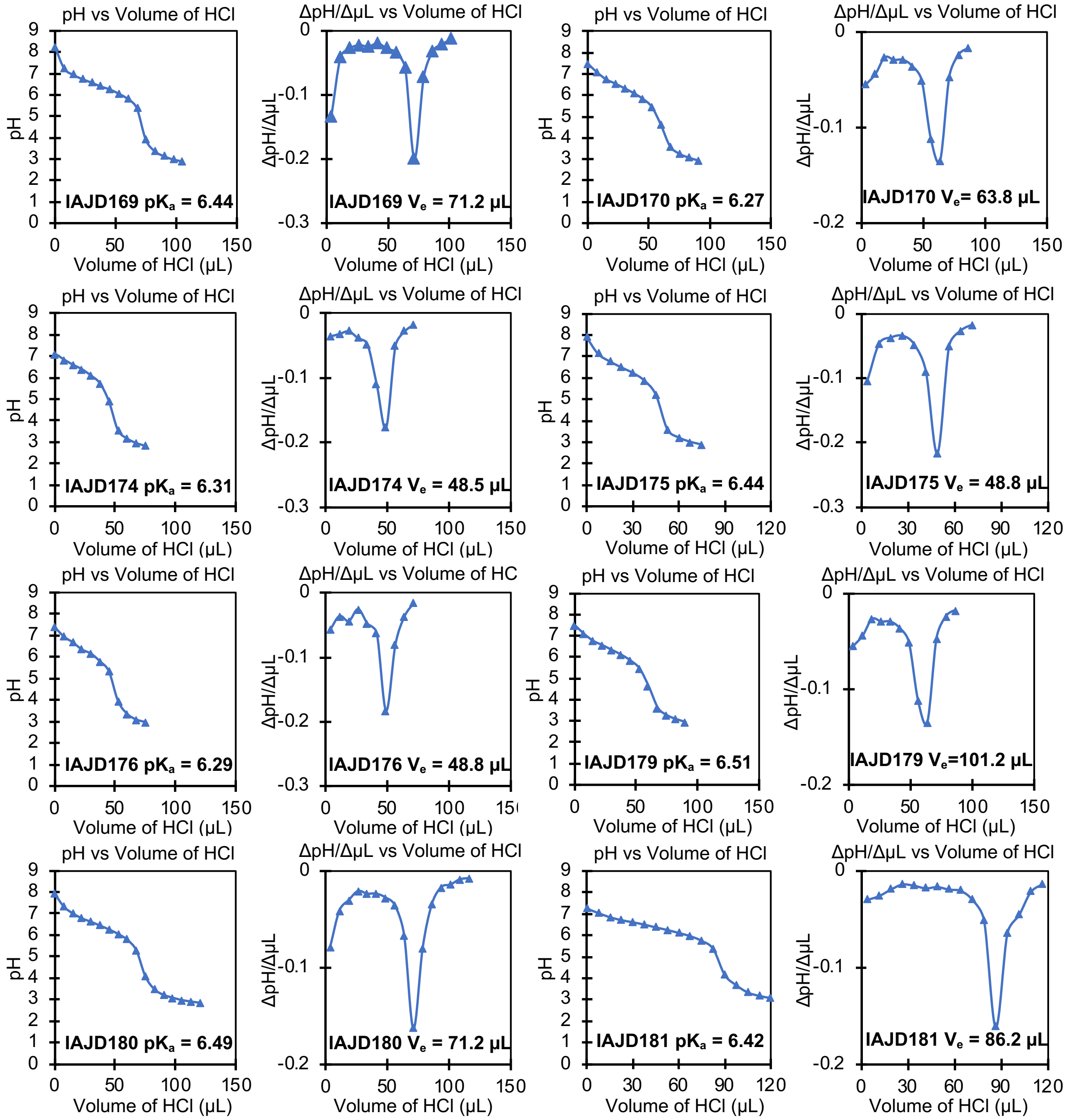

Figure S26. Titration curves showing changes in solution pH in response to addition of a strong acid and calculated pKa for IAJDs 169,170,174-176, 179-181.

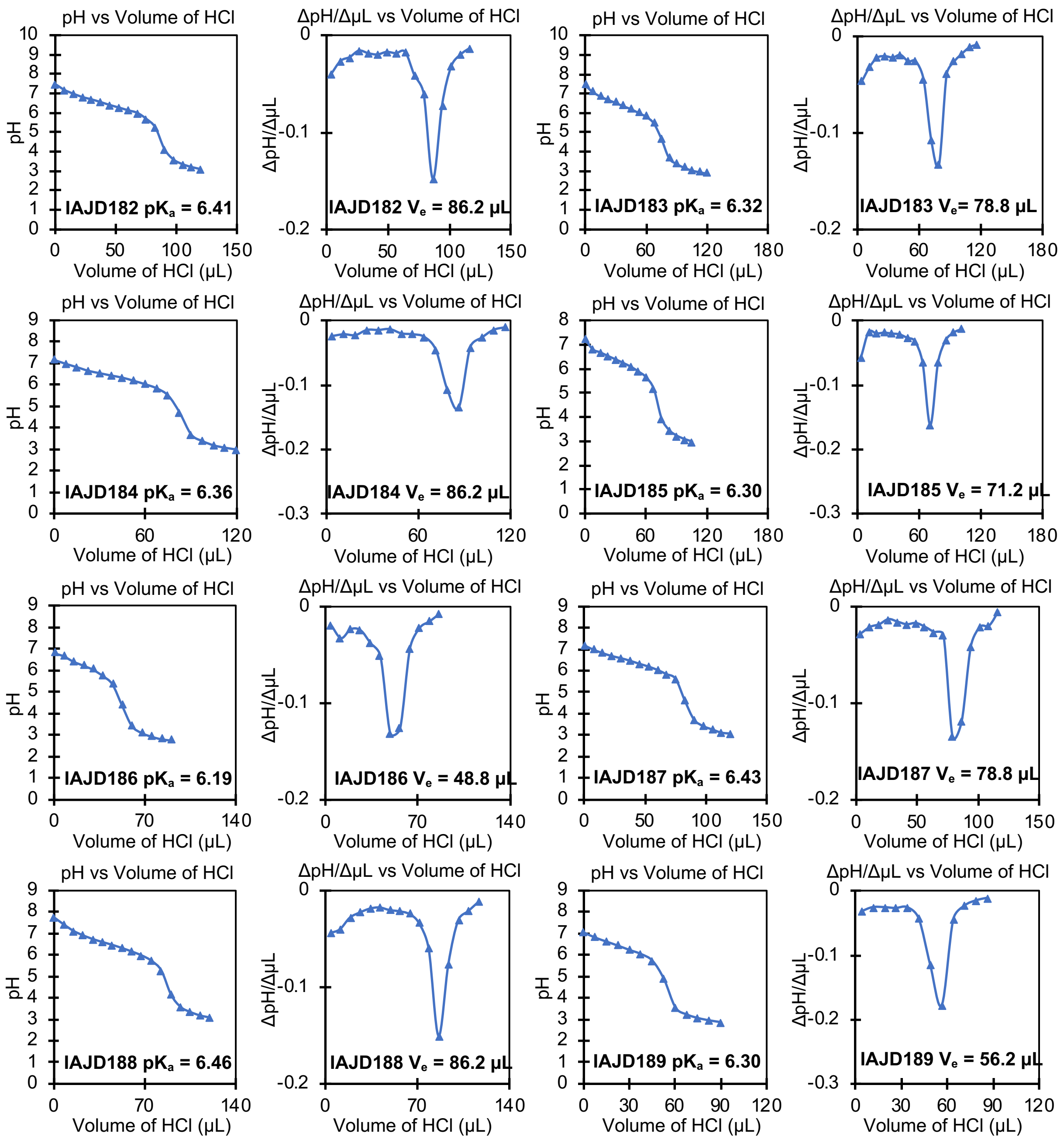

Figure S27. Titration curves showing changes in solution pH in response to addition of a strong acid and calculated pKa for IAJDs 182-189.

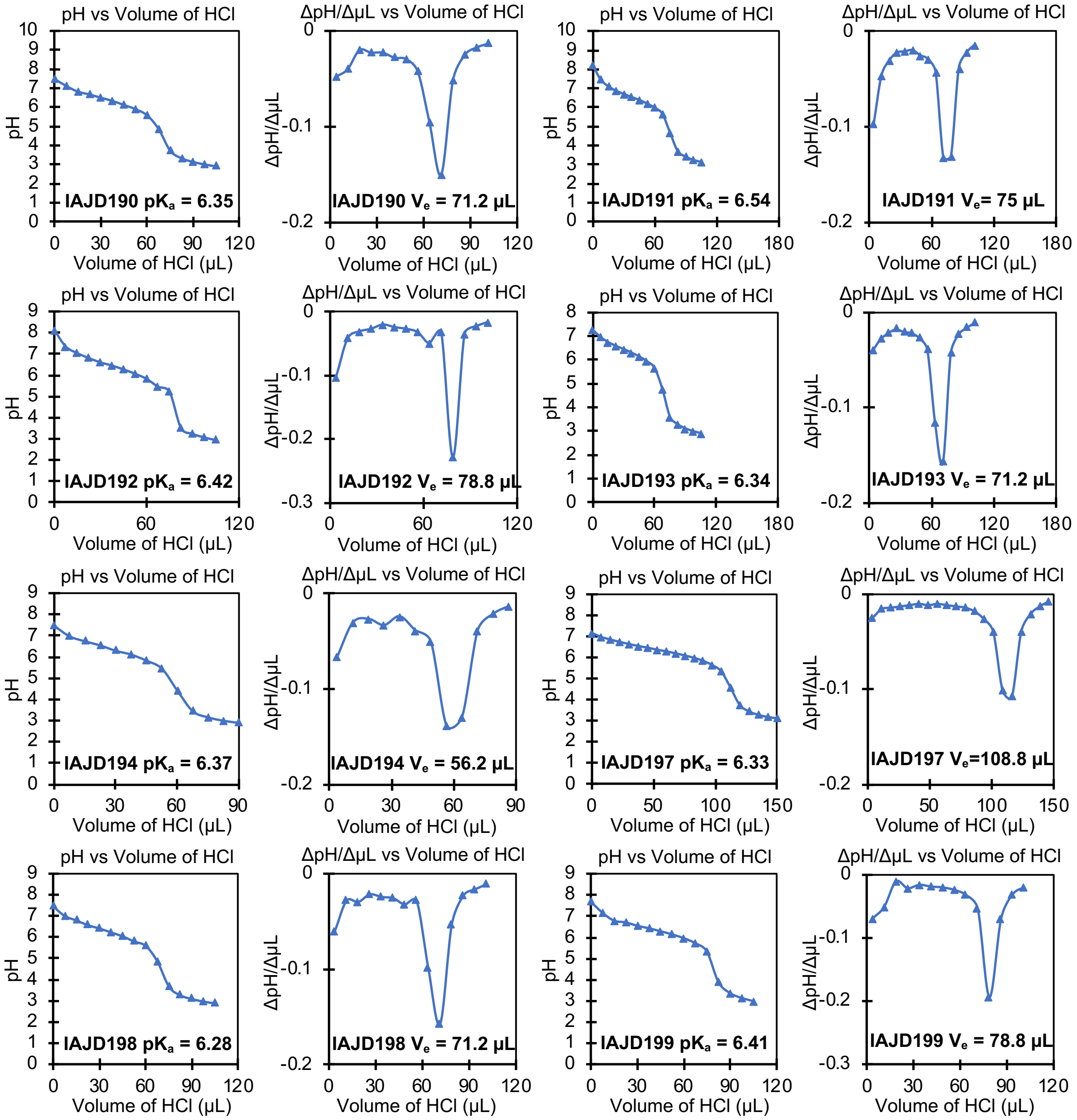

Figure S28. Titration curves showing changes in solution pH in response to addition of a strong acid and calculated pKa for IAJDs 190-194,197-199.

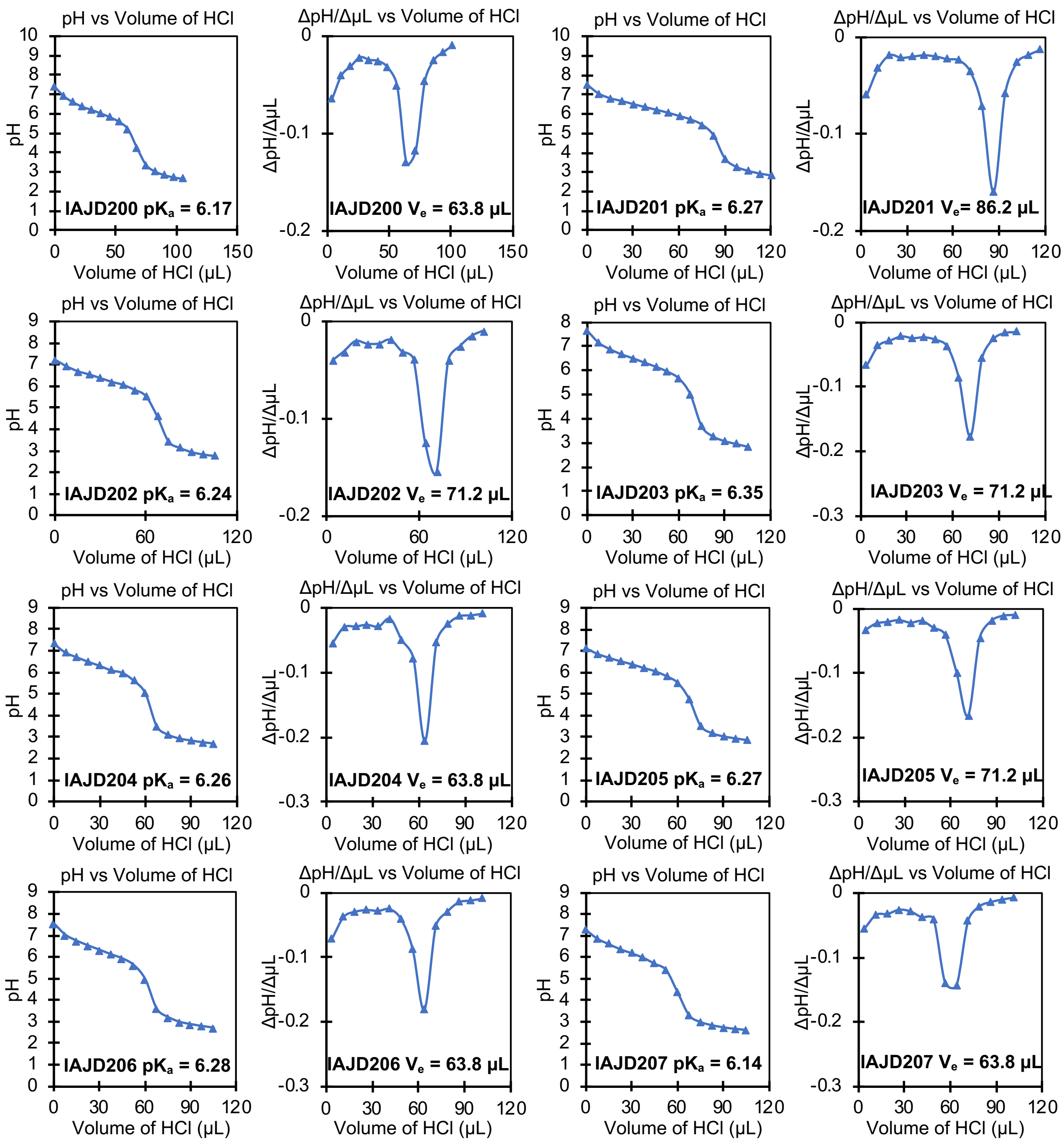

Figure S29. Titration curves showing changes in solution pH in response to addition of a strong acid and calculated pKa for IAJDs 200-207.

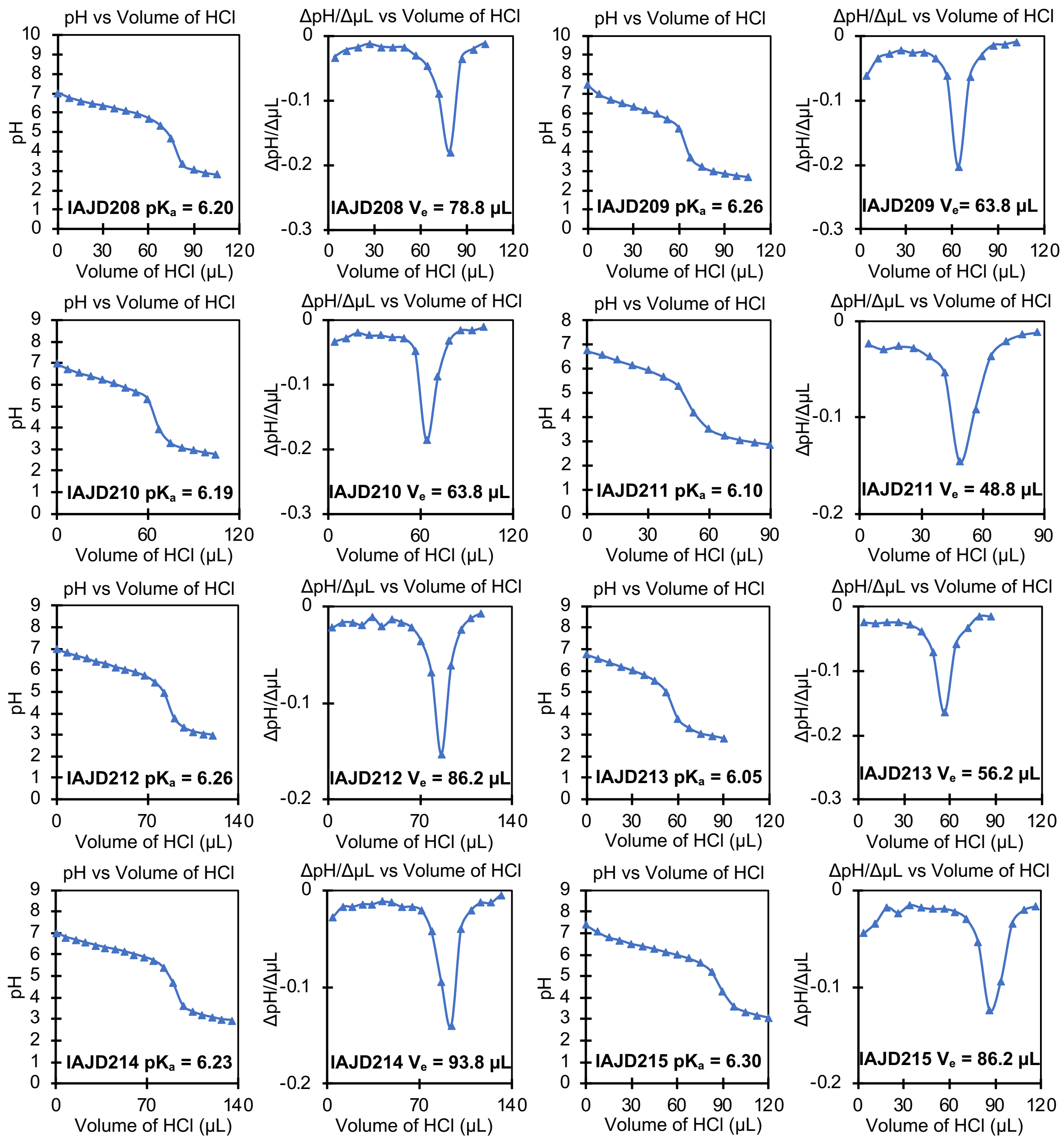

Figure S30. Titration curves showing changes in solution pH in response to addition of a strong acid and calculated pKa for IAJDs 208-215.

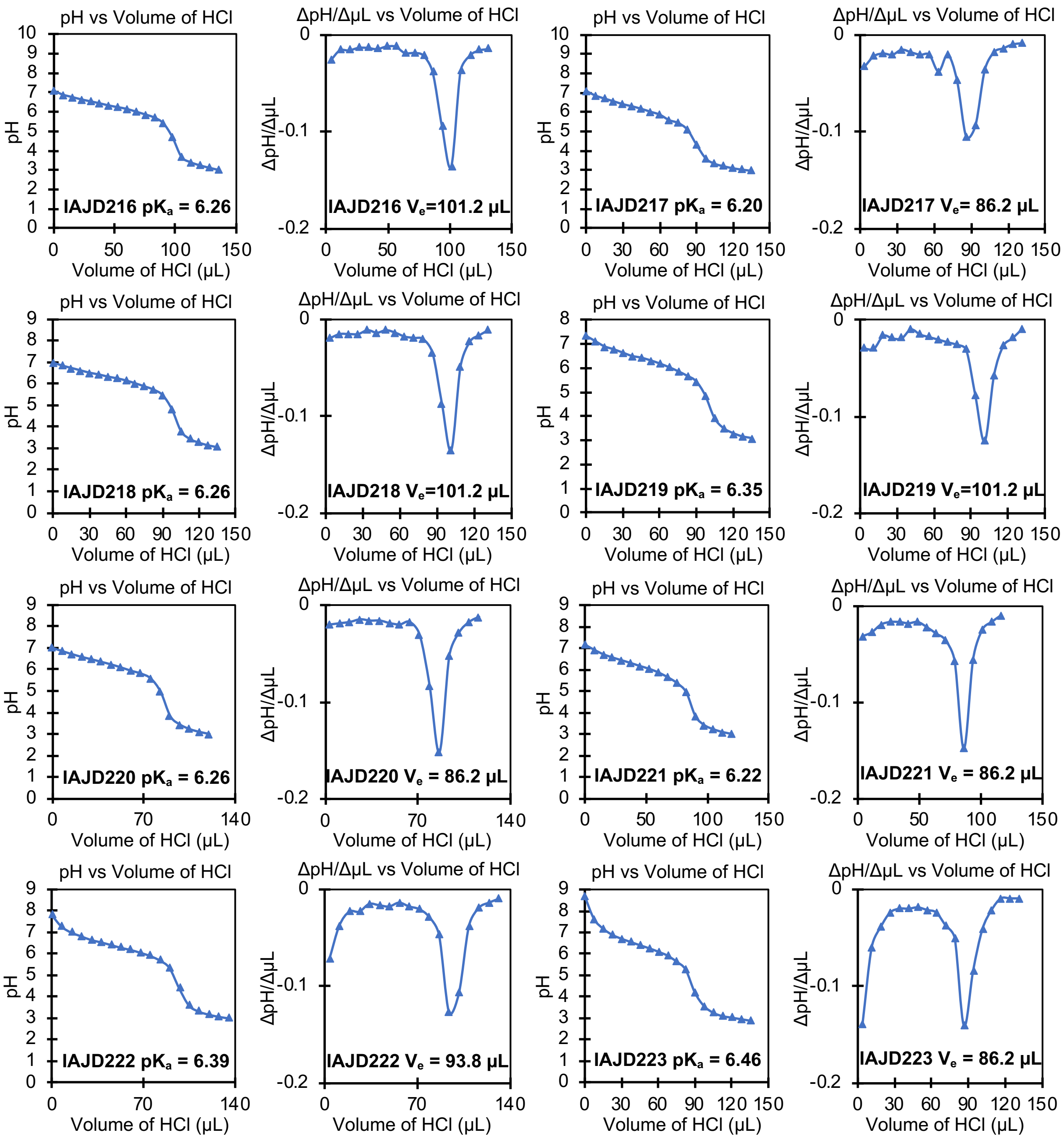

Figure S31. Titration curves showing changes in solution pH in response to addition of a strong acid and calculated pKa for IAJDs 261-223.

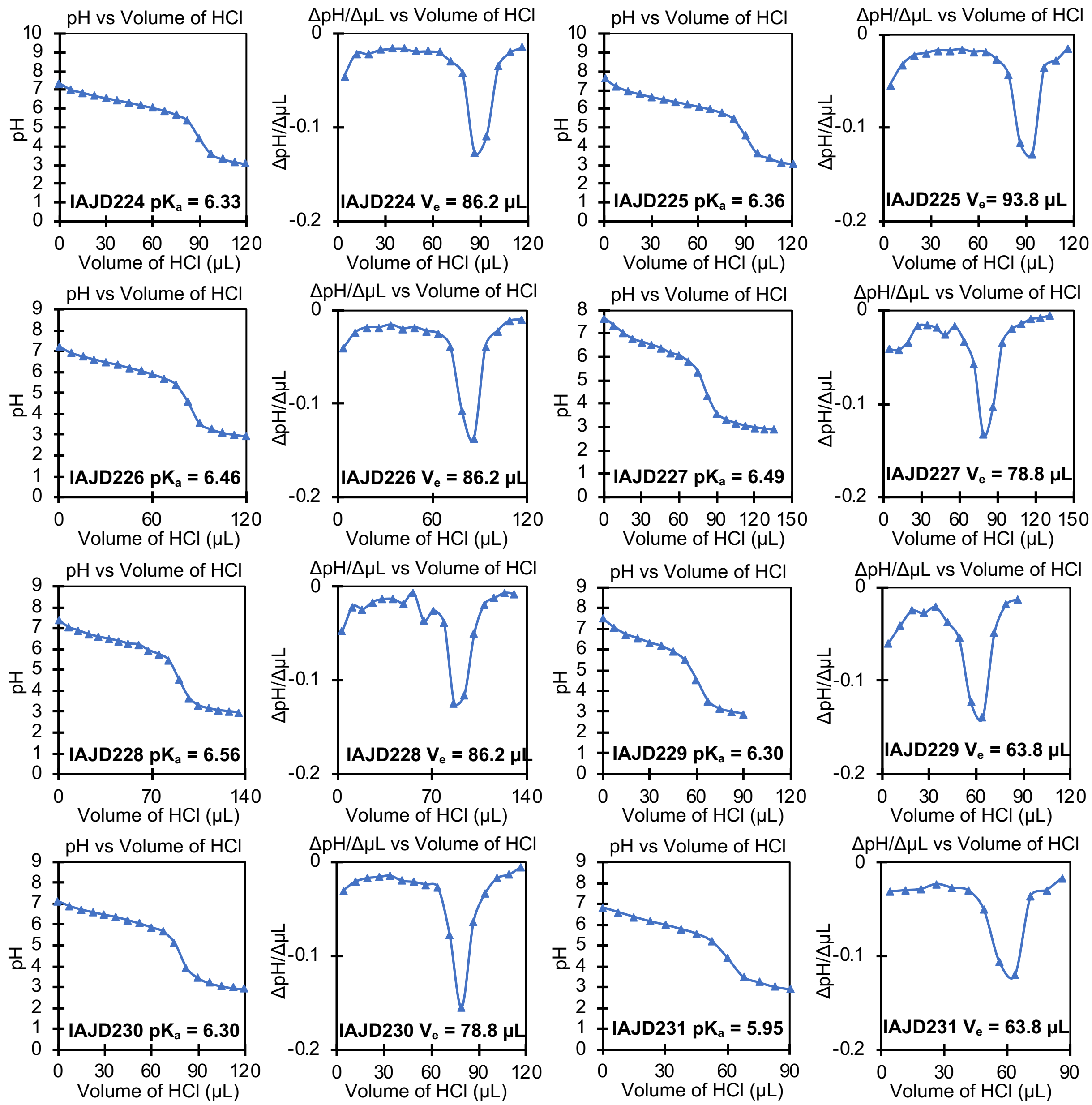

Figure S32. Titration curves showing changes in solution pH in response to addition of a strong acid and calculated pKa for IAJDs 224-231.

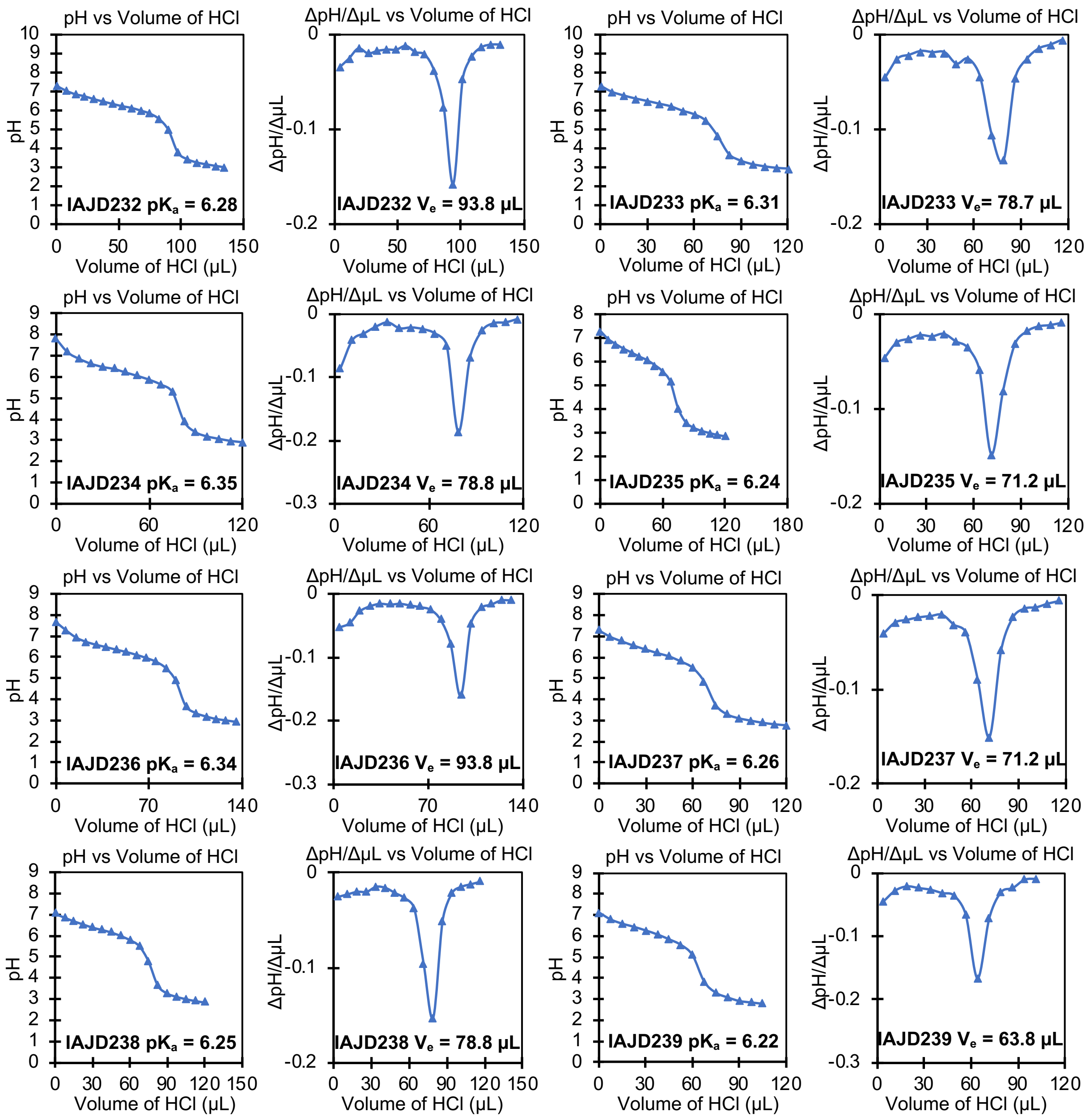

Figure S33. Titration curves showing changes in solution pH in response to addition of a strong acid and calculated pKa for IAJDs 232-239.

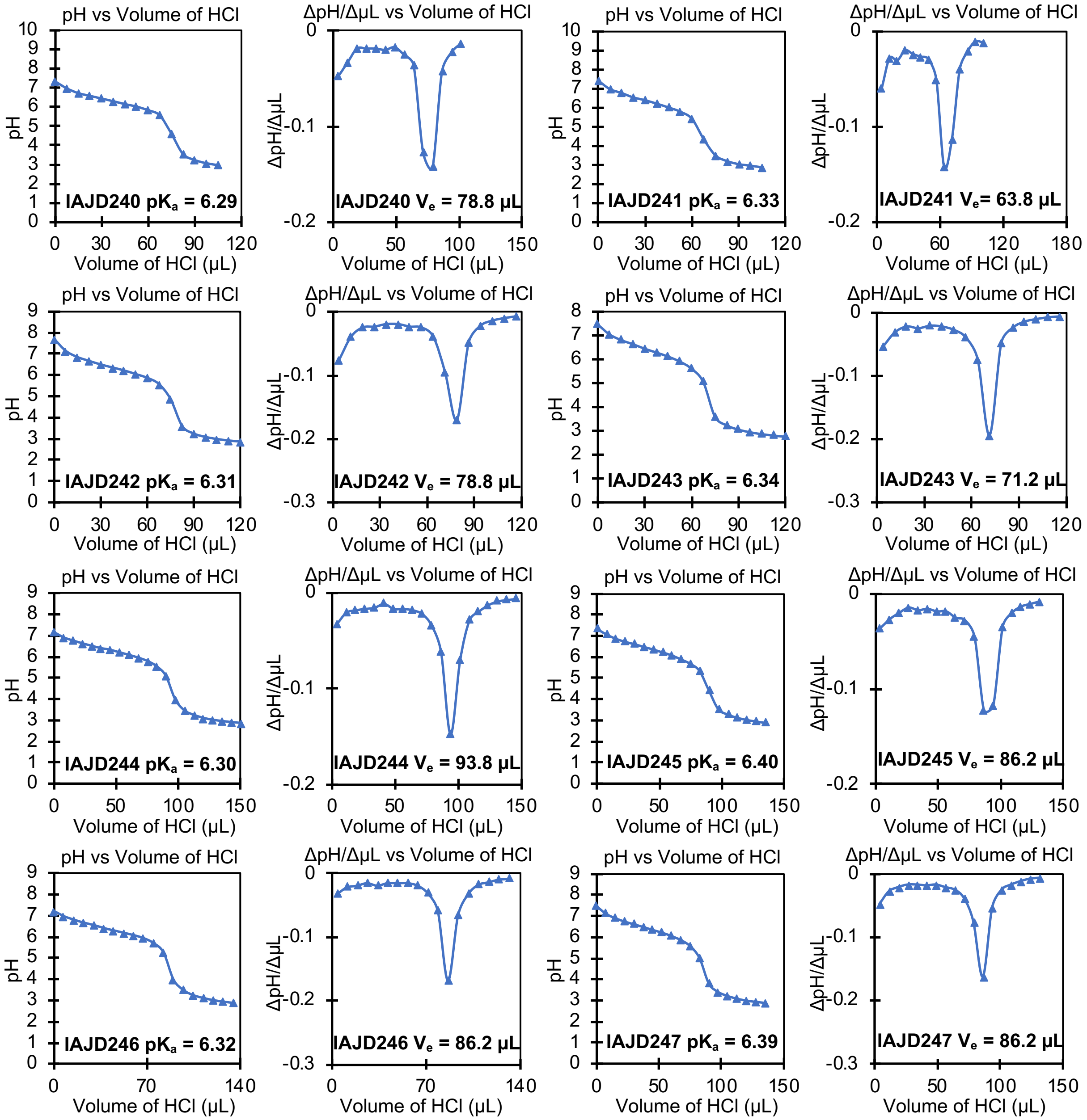

Figure S34. Titration curves showing changes in solution pH in response to addition of a strong acid and calculated pKa for IAJDs 240-247.

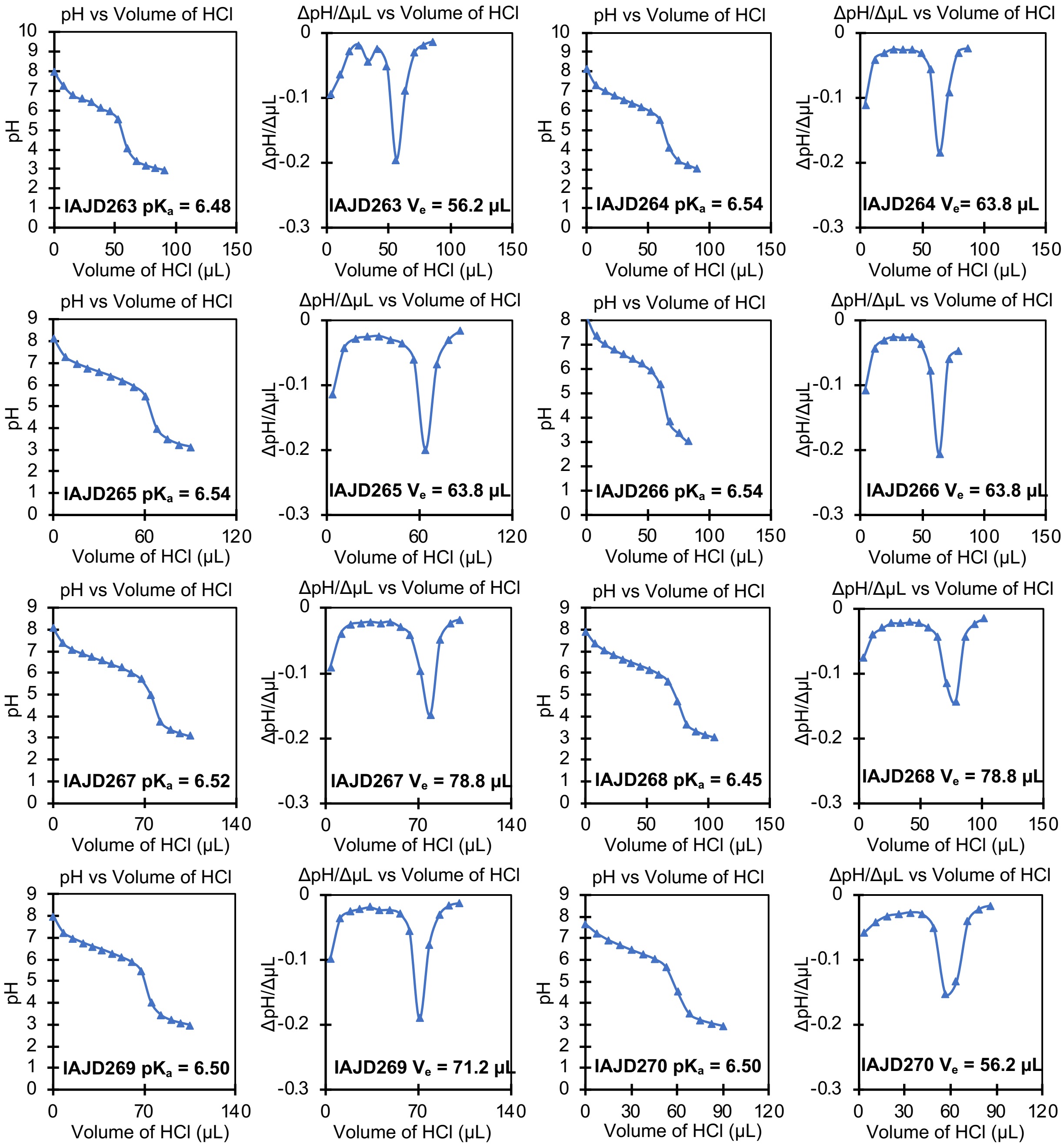

Figure S35. Titration curves showing changes in solution pH in response to addition of a strong acid and calculated pKa for IAJDs 263-270.

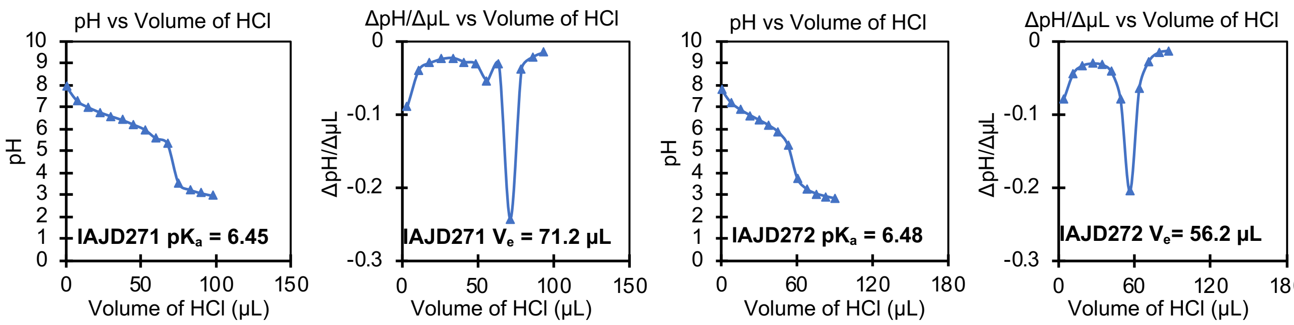

Figure S36. Titration curves showing changes in solution pH in response to addition of a strong acid and calculated pKa for IAJDs 271,272.

6. Supporting References

(1) Moore, J. S.; Stupp, S. I. Room Temperature Polyesterification. *Macromolecules* **1990**, *23*, 65–70. 10.1021/ma00203a013

(2) Pardi, N.; Muramatsu, H.; Weissman, D.; Kariko, K. In Vitro Transcription of Long RNA Containing Modified Nucleosides. *Methods Mol. Biol.* **2013**, *969*, 29–42. 10.1007/978-1-62703-260-5_2

(3) Zhang, D.; Atochina-Vasserman, E. N.; Maurya, D. S.; Liu, M.; Xiao, Q.; Lu, J.; Lauri, G.; Ona, N.; Reagan, E. K.; Ni, H.; Weissman, D.; Percec, V. Targeted Delivery of mRNA with One-Component Ionizable Amphiphilic Janus Dendrimers. *J. Am. Chem. Soc.* **2021**, *143*, 17975−17982. 10.1021/jacs.1c09585

(4) Zhang, D.; Atochina-Vasserman, E. N.; Maurya, D. S.; Huang, N.; Xiao, Q.; Ona, N.; Liu, M.; Shahnawaz, H.; Ni, H.; Kim, K.; Billingsley, M. M.; Pochan, D. J.; Mitchell, M. J.; Weissman, D.; Percec, V. One-Component Multifunctional Sequence-Defined Ionizable Amphiphilic Janus Dendrimer Delivery Systems for mRNA. *J. Am. Chem. Soc.* **2021**, *143*, 12315–12327. 10.1021/jacs.1c05813

(5) Zhang, D.; Atochina-Vasserman, E. N.; Lu, J.; Maurya, D. S.; Xiao, Q.; Liu, M.; Adamson, J.; Ona, N.; Reagan, E. K.; Ni, H.; et al. The Unexpected Importance of the Primary Structure of the Hydrophobic Part of One-Component Ionizable Amphiphilic Janus Dendrimers in Targeted MRNA Delivery Activity. *J. Am. Chem. Soc.* **2022**, *144* (11), 4746–4753. 10.1021/jacs.2c00273

(6) May, J. P.; Undzys, E.; Roy, A.; Li, S.-D. Synthesis of a Gemcitabine Prodrug for Remote Loading into Liposomes and Improved Therapeutic Effect. *Bioconjugate Chem.* **2016**, *27* (1), 226–237. 10.1021/acs.bioconjchem.5b00619
